# Non-bonding 1,5-S···O interactions govern chemo- and enantioselectivity in isothiourea-catalyzed annulations of benzazoles†
†Electronic supplementary information (ESI) available: ^1^H and ^13^C{^1^H} NMR spectra and HPLC traces of all novel compounds. Coordinates, thermal corrections, and energies of all computed structures. See DOI: 10.1039/c6sc00940a



**DOI:** 10.1039/c6sc00940a

**Published:** 2016-07-04

**Authors:** Emily R. T. Robinson, Daniel M. Walden, Charlene Fallan, Mark D. Greenhalgh, Paul Ha-Yeon Cheong, Andrew D. Smith

**Affiliations:** a EaStCHEM , School of Chemistry , University of St Andrews , North Haugh , St Andrews KY16 9ST , UK . Email: ads10@st-andrews.ac.uk; b Department of Chemistry , Oregon State University , 135 Gilbert Hall , Corvallis , OR 97331 , USA . Email: cheongh@oregonstate.edu

## Abstract

Isothiourea-catalyzed annulations between 2-acyl benzazoles and α,β-unsaturated acyl ammonium intermediates are selectively tuned to form either lactam or lactone heterocycles in good yields (up to 95%) and high ee (up to 99%) using benzothiazole or benzoxazole derivatives, respectively. Computation gives insight into the significant role of two 1,5-S···O interactions in controlling the structural preorganization and chemoselectivity observed within the lactam synthesis with benzothiazoles as nucleophiles. When using benzazoles the absence of a second stabilizing non-bonding 1,5-S···O interaction leads to a dominant C–H···O interaction in determining structural preorganization and lactone formation.

## Abstract 2

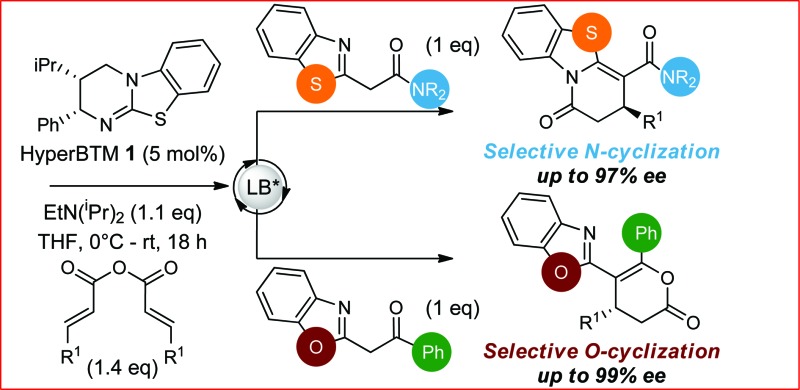
Isothiourea-catalyzed annulations of 2-acyl benzazoles and α,β-unsaturated acyl ammoniums are tuned to form lactams or lactones (up to 99% ee). Computations highlight the governing roles of S···O and CH···O interactions.

## Introduction

Nitrogen-containing heterocycles are of wide-spread importance in pharmaceutical, agrochemical and material science industries.^1^ In particular, benzazoles have found broad-reaching applications as bioactive compounds in medicinal chemistry, with a range of therapeutic treatments exploiting their anti-bacterial, anti-fungal, anti-parasitic and anti-cancer properties.^2^ In addition, they are key components of useful ligands^3^ as well as organic semiconductors and dyes.^4^ The prevalence of the benzazole motif in these applications has led the synthetic community to develop numerous methodologies for the use of benzazole containing nucleophiles for the rapid synthesis of complex heterocycles.^5^

**Scheme 1:**
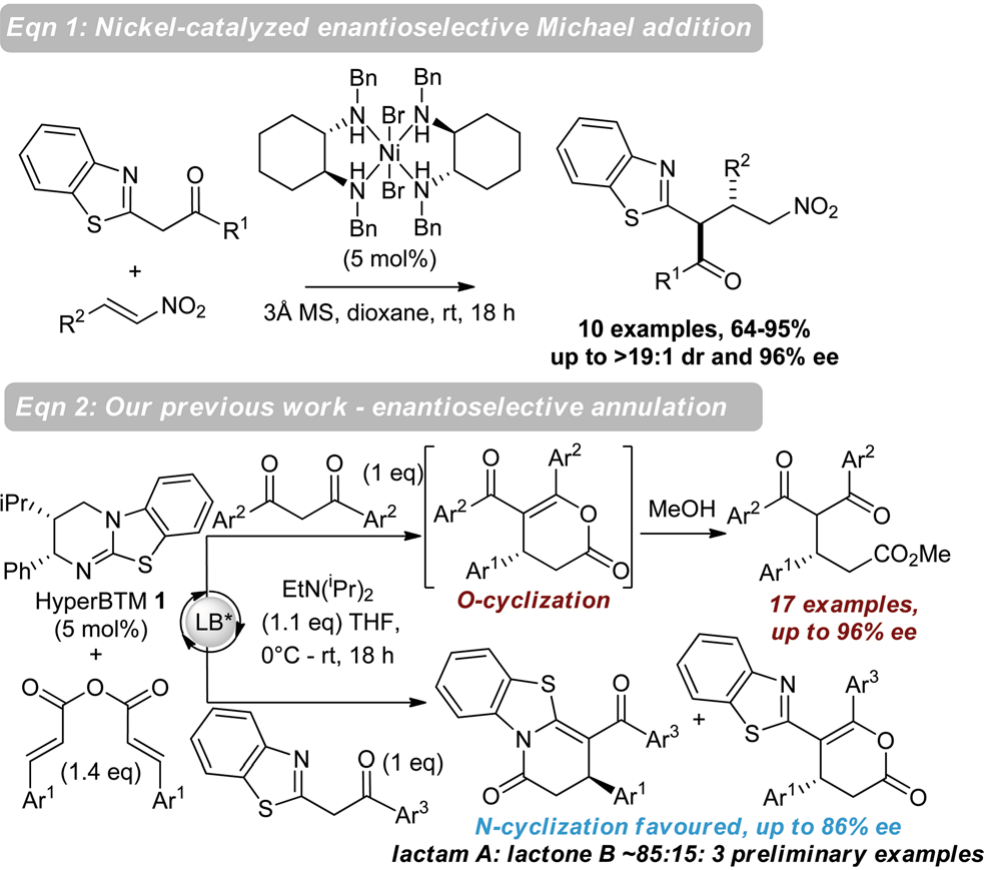
Previous work using benzazoles in enantioselective catalysis. Eqn (1) nickel-catalyzed Michael addition to nitroalkenes; eqn (2) isothiourea-catalyzed enantioselective annulation with α,β-unsaturated acyl ammonium intermediates.

Despite this interest, catalytic enantioselective functionalization of benzazole derivatives has received limited attention, with only a small number of enantioselective protocols developed to date.^6^ As a representative example of such an approach, Lam has shown that benzazoles undergo catalytic enantioselective nickel-catalyzed Michael-additions to nitroalkenes, giving the desired products in high yields, moderate to excellent dr and good to excellent enantioselectivity (Scheme 1, eqn (1)).^7^ As part of our ongoing research employing isothioureas^8^ in catalysis,^9^ we recently developed an enantioselective annulation process utilizing α,β-unsaturated acyl ammonium intermediates.^10,11^ In this annulation process, reaction of this intermediate with symmetrical 1,3-dicarbonyl nucleophiles generates functionalized esters in high ee after ring-opening through a postulated Michael addition-lactonization/ring-opening process (17 examples, up to 96% ee). Notably, preliminary results using unsymmetrical 2-phenacylbenzothiazole as a nucleophile gave functionalized lactams preferentially (∼85 : 15 lactam : lactone), resulting from preferential *N*- rather than *O*-cyclization, through a Michael addition-lactamization process in up to 86% ee in three isolated examples (Scheme 1, eqn (2)).

**Scheme 2:**
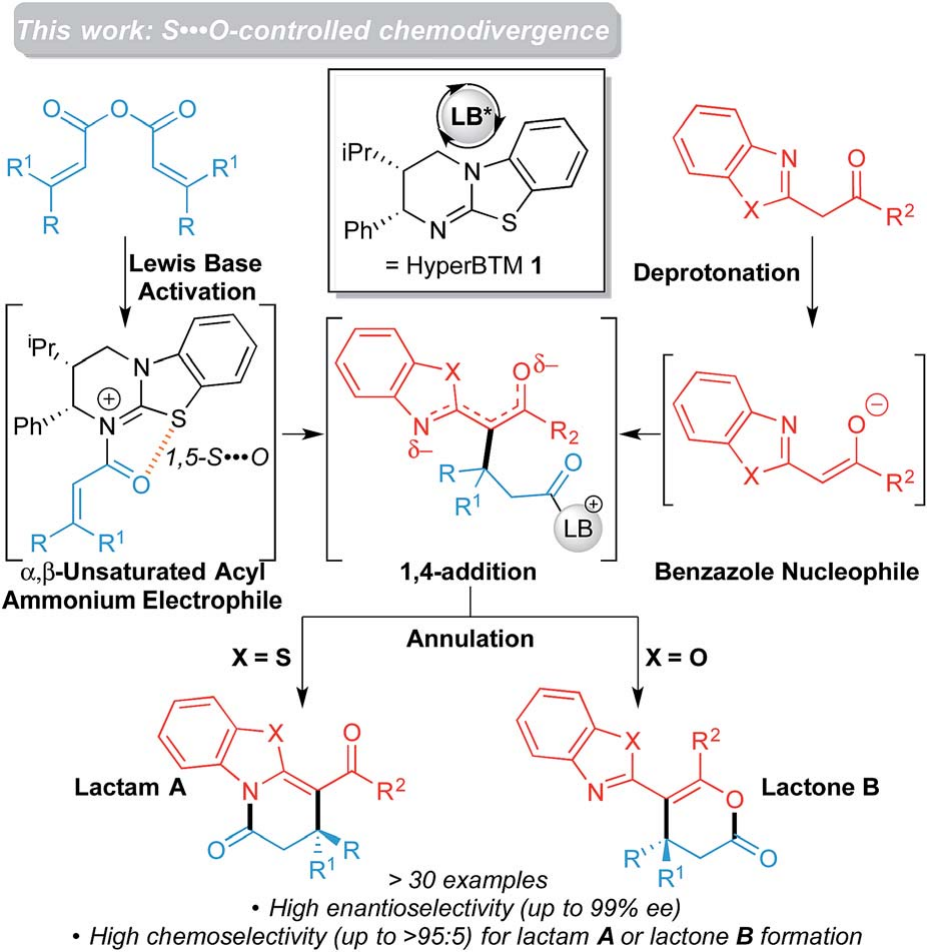
Chemo- and enantioselective isothiourea-catalyzed annulation of acylbenzazoles with α,β-unsaturated acyl ammonium intermediates.

This manuscript builds upon the intriguing chemoselectivity observed in the preferential formation of lactams in this latter process, and subsequently explores the effect of changing both carbonyl substitution and the heteroatom within a series of acylbenzazole nucleophiles. As a result, we have developed a highly chemoselective method to access either lactam **A** or lactone **B** heterocyclic products in excellent enantioselectivity through use of acylbenzothiazole or acylbenzoxazole derivatives respectively (Scheme 2). Furthermore, through computations, the role that non-bonding 1,5-S···O interactions and C–H···O interactions play in governing the unusual regioselectivity of these processes is highlighted. The importance of non-bonding S···O interactions has been widely recognized in structural and medicinal chemistry in the solid state (commonly ascribed to a stabilizing n_O_ to σ* interaction),^12^ and has been used as a key controlling element to rationalize enantioselective isothiourea-catalyzed reactions.^13^ While the origin of this interaction is still under debate,^14^ and is the focus of ongoing work within our research groups, the demonstration of alternative examples of how non-bonding S···O interactions can facilitate selectivity in catalysis could lead to its broader utilization, akin to the current widespread use of hydrogen bonding and other non-bonding interactions in synthesis.^15^ To the best of our knowledge, S···O interactions have not been invoked to describe the origins of chemoselectivity in a catalytic reaction.

## Results and discussion

### Probing the effects of acyl and benzazole substituents on annulation chemo- and enantioselectivity

**Scheme 3:**
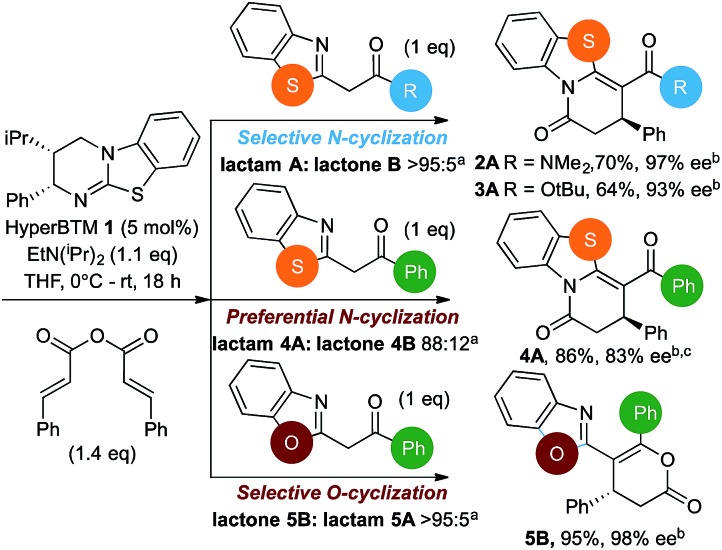
Probing the effects of acyl and benzazole substituents on annulation chemo- and enantioselectivity. ^a^Ratio of constitutional isomers arising from either *N*- or *O*-cyclization calculated from ^1^H NMR spectra of crude reaction product. ^b^ee values obtained *via* chiral HPLC. ^c^Following a single recrystallization ee could be enhanced to 97%.

Initial investigations sequentially probed substituent effects on the chemo- and enantioselectivity of this annulation process within a series of acylbenzazoles, with variation of both the acyl substituent and heterocycle tested (Scheme 3). Consistent with our previous studies, using homoanhydrides as α,β-unsaturated acyl ammonium precursors with isothiourea HyperBTM **1** (5 mol%) in bench-grade THF, 2-phenacylbenzothiazole gave preferentially lactam product **4A** (88 : 12 lactam **4A** : lactone **4B**), with **4A** isolated in 86% yield and 83% ee that was recrystallized to give **4A** in 68% yield and 97% ee. A small amount of the lactone constitutional isomer **4B** was also isolated (9% yield, 86% ee).^16^ The potential for isomerization of lactone **4B** to the lactam **4A** was investigated under a range of conditions. Treatment of the minor lactone product **4B** with base, with base and HyperBTM, or to the reaction conditions, led to no interconversion of lactone to lactam, consistent with the observed product ratios arising from kinetic control (see ESI† for further details). The incorporation of electron donor benzothiazole amides and esters resulted in the exclusive formation of lactams **2A** and **3A** as single constitutional isomers in excellent ee (97% and 93% ee) and in good yields respectively. Further studies probed the effect of variation within the heterocyclic portion of the benzazole. While using 2-phenacylbenzothiazole leads to preferential formation of lactam **4A**, remarkably, 2-phenacylbenzoxazole afforded exclusively lactone product (>95 : 5 **5B** : **5A**) with the lactone **5B** isolated in 95% yield and 98% ee. The seemingly trivial substrate change from benzothiazole to benzoxazole in this system promotes a change in chemoselectivity in the annulation process to selectively facilitate lactone (*O*-cyclization) rather than lactam (*N*-cyclization) product formation.

### Scope and generality

To demonstrate the generality of these chemo- and enantioselective annulation processes, and facilitate direct comparison across a range of substrates, the use of 2-phenacylbenzothiazole, 2-phenacylbenzoxazole and 2-*N*,*N*-dimethylacetamidobenzothiazole as nucleophiles was fully investigated with a range of anhydrides (Table 1).

**Table 1:** Chemo- and enantioselective formation of lactam or lactone products

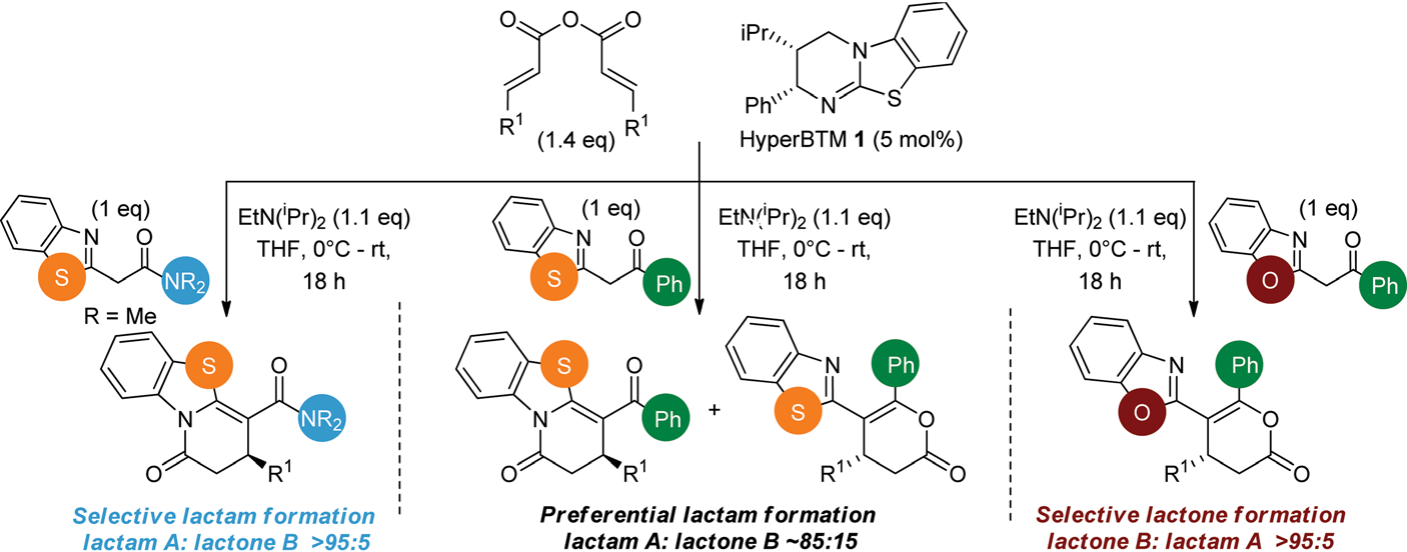		
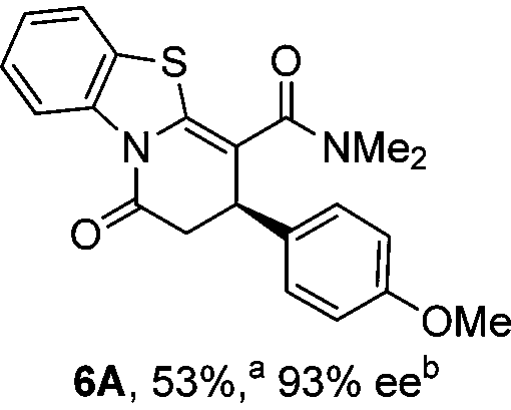	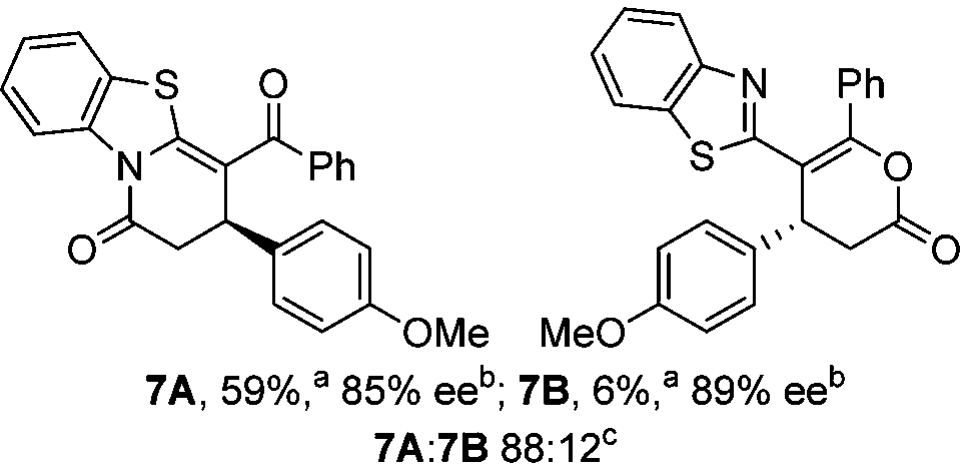	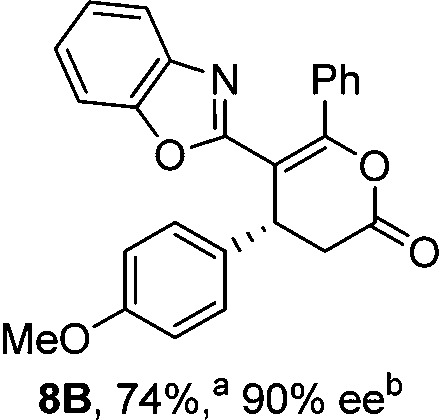
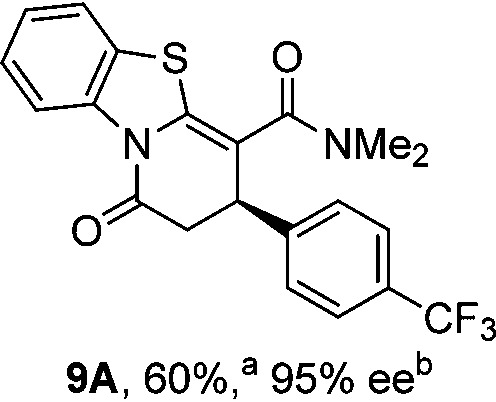	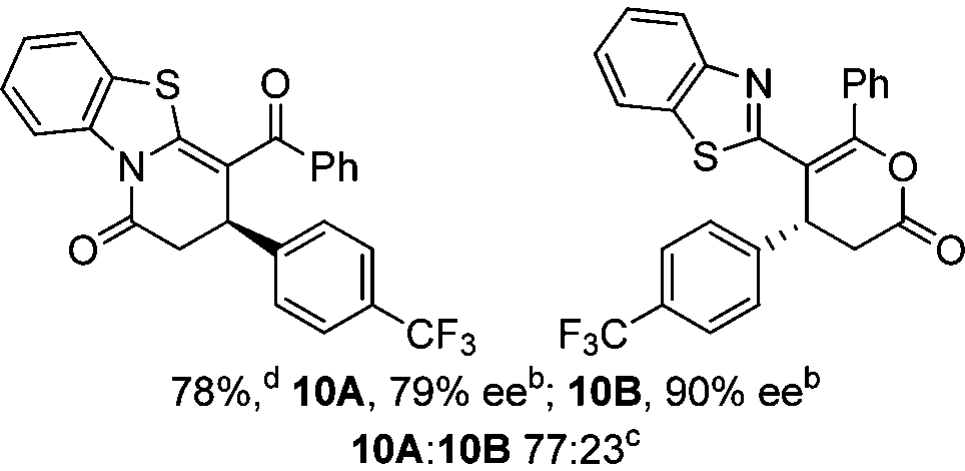	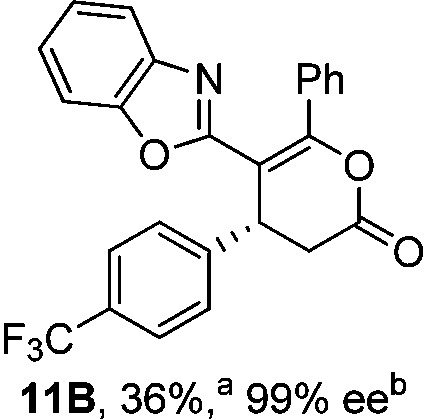
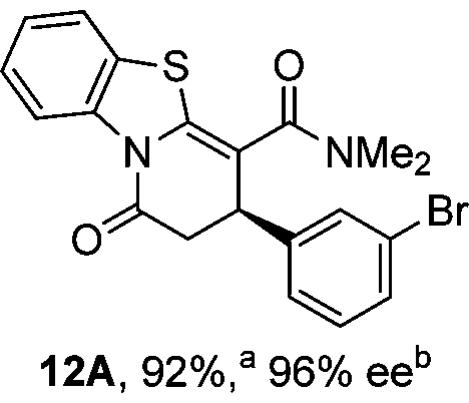	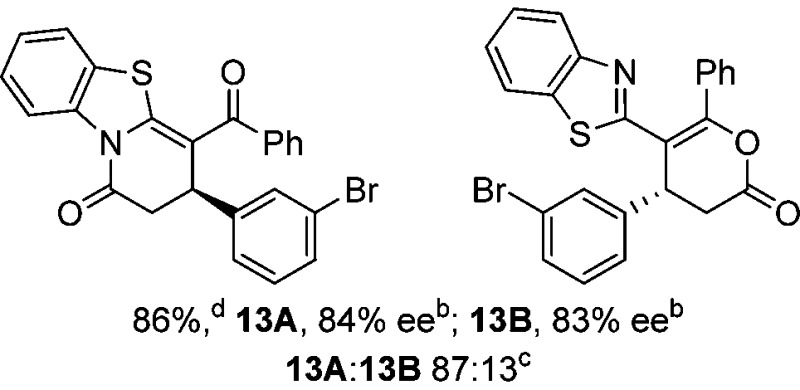	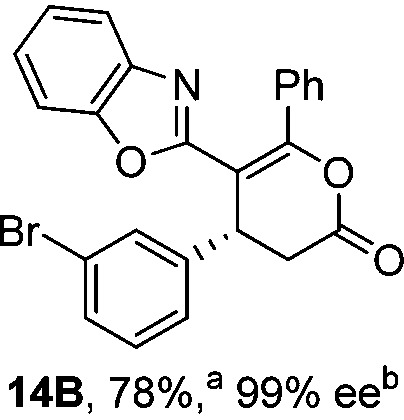
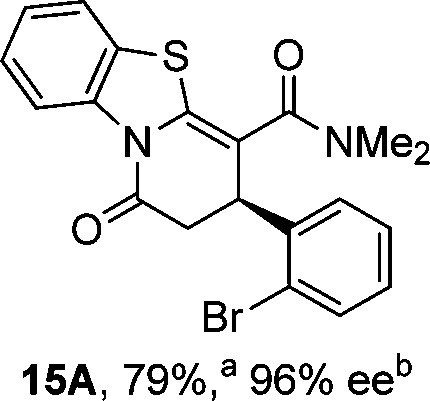	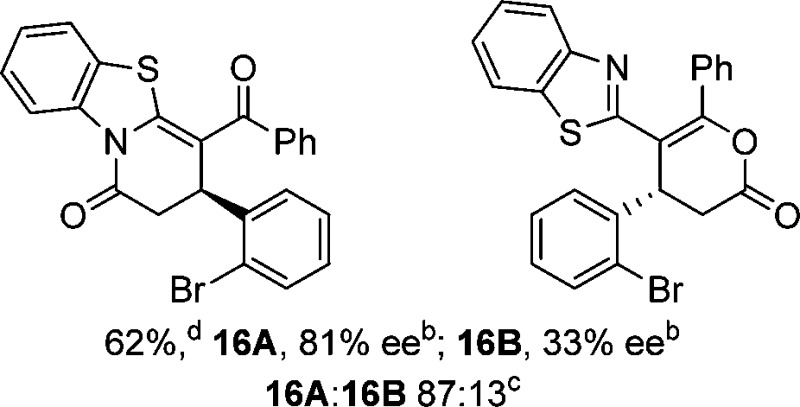	No reaction
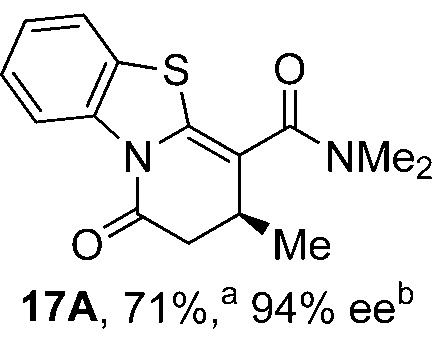	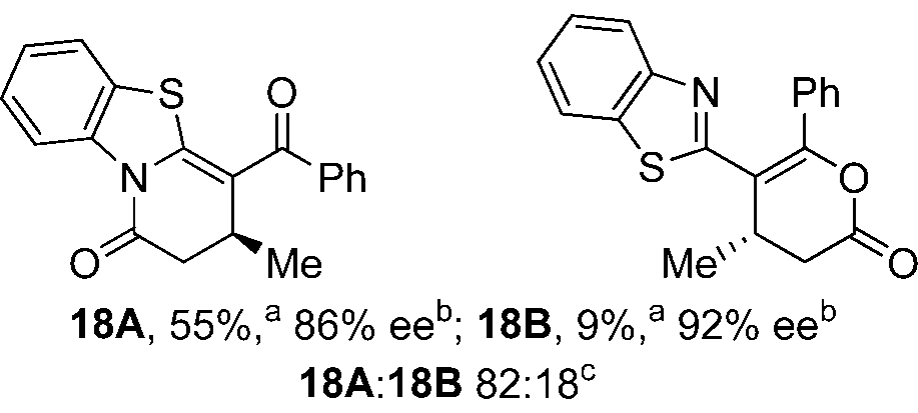	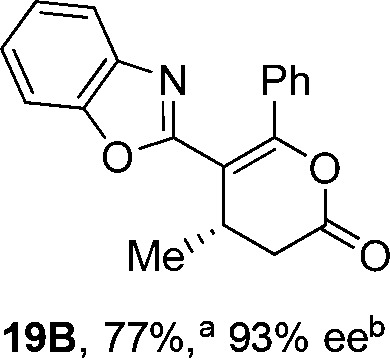
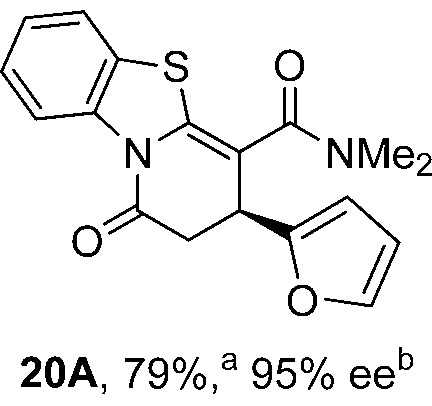	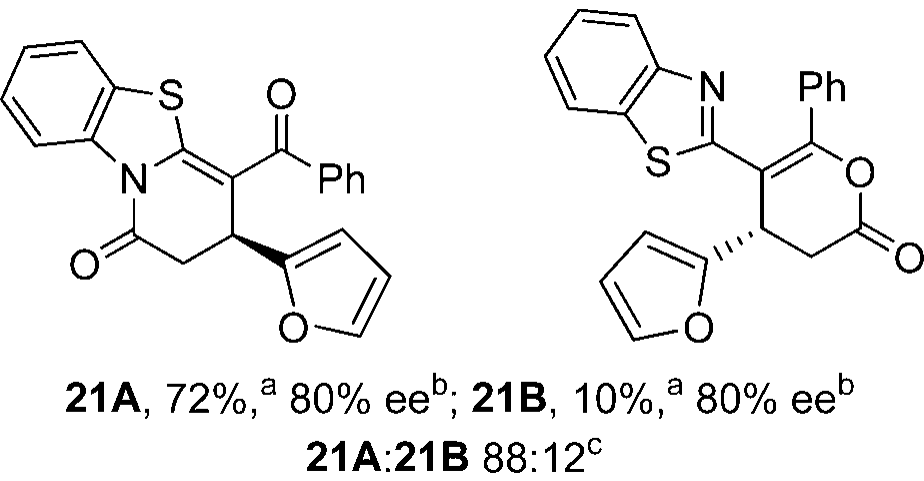	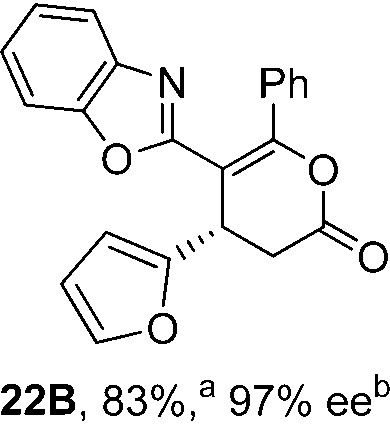
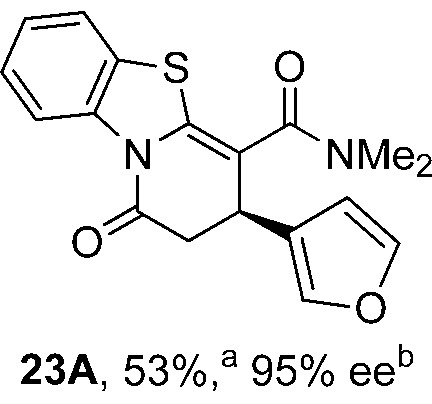	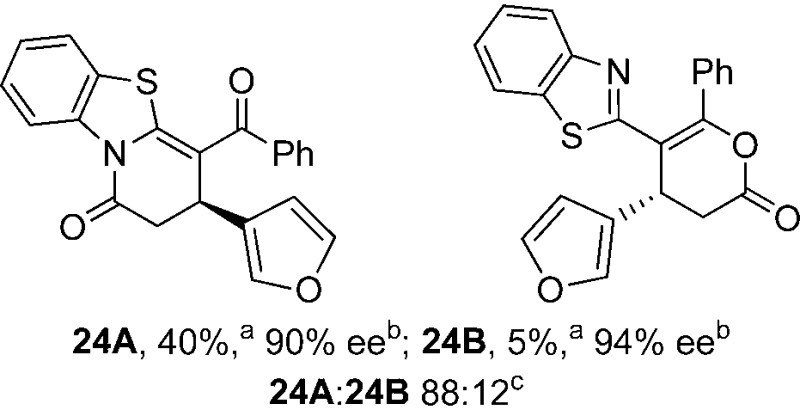	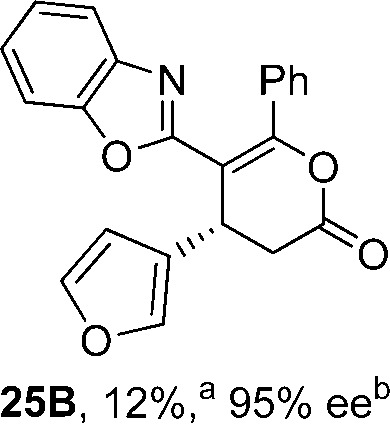
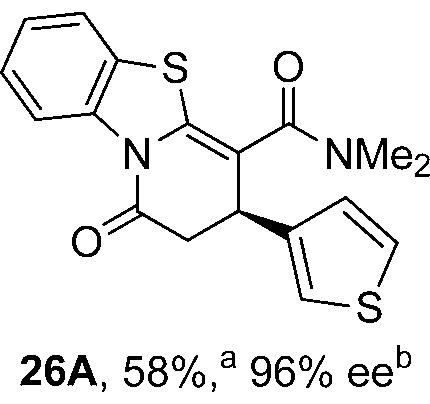	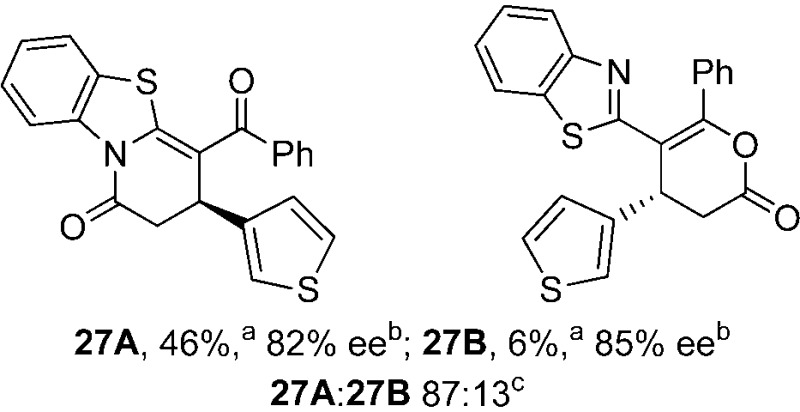	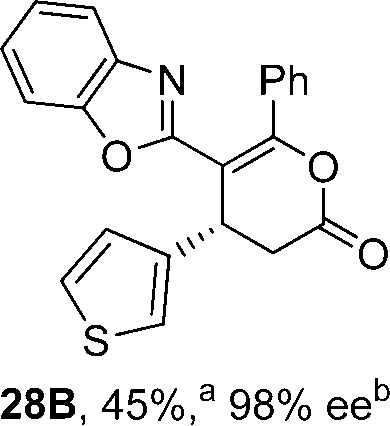
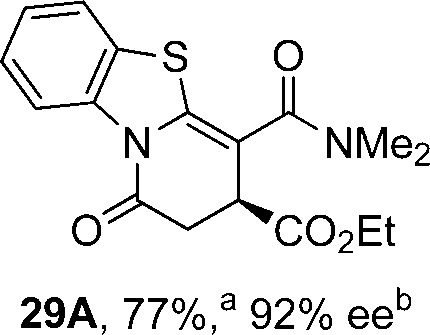	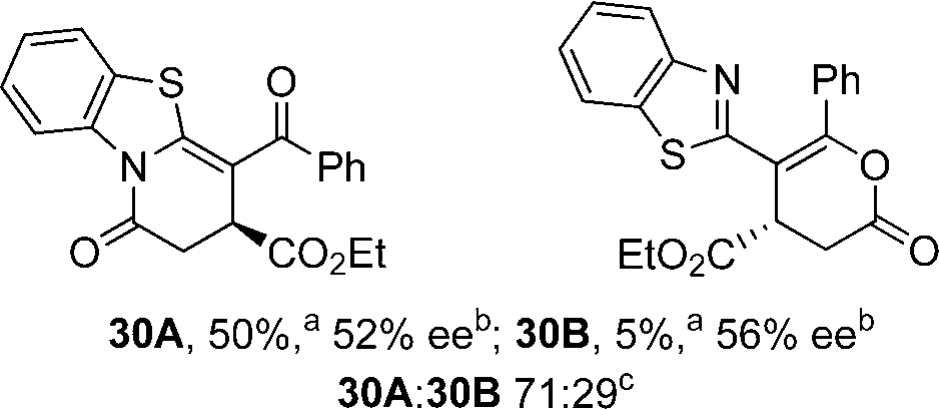	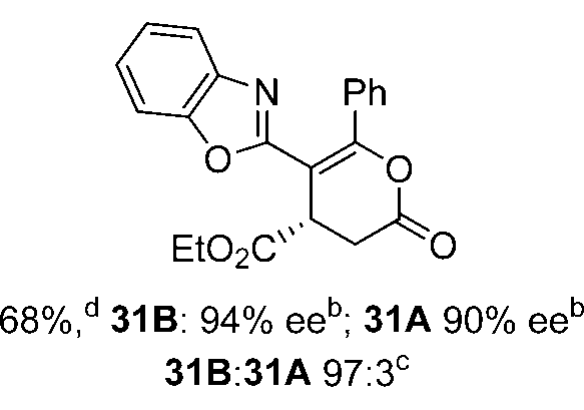

^*a*^Isolated yield of single constitutional isomer.

^*b*^ee values obtained *via* chiral HPLC.

^*c*^Ratio of constitutional isomers calculated from ^1^H NMR spectra of crude reaction product.

^*d*^Isolated yield of inseparable mixture of constitutional isomers.

Consistent with the model studies, chemoselective formation of either lactam or lactone products (>95 : 5 ratio of constitutional isomers) was achieved by using 2-*N*,*N*-dimethylacetamidobenzothiazole or 2-phenacylbenzoxazole, with excellent enantioselectivity (90–99% ee) observed across a range of anhydrides. Using 2-phenacylbenzothiazole led to preferential lactam formation (typically ∼85 : 15 lactam : lactone), albeit with reduced enantioselectivity (typically >80% ee). For all acylbenzazole nucleophiles, variation of aryl substitution within the anhydride was tolerated, including electron donating (4-MeOC_6_H_4_), electron withdrawing (4-CF_3_C_6_H_4_), and 3-BrC_6_H_4_ substitution. Sterically demanding 2-BrC_6_H_4_ substitution led to no reaction with 2-phenacylbenzoxazole, while reactions using 2-*N*,*N*-dimethylacetamidobenzothiazole or 2-phenacylbenzothiazole gave acceptable to good product yields, with excellent enantioselectivity in the amide series. Heteroaryl (2-furyl, 3-furyl, and 3-thiophenyl) substituents were also successfully incorporated (90–99% ee), as were methyl and ester substitution. In the 2-phenacylbenzothiazole derived series, the ee of lactam and lactone products were approximately equivalent, except for **16A**/**16B** (81% and 33% ee respectively) bearing a 2-Br substituent. The origin of this variation in ee is currently unexplained, despite extensive synthetic and computational investigations.^17^

**Scheme 4:**
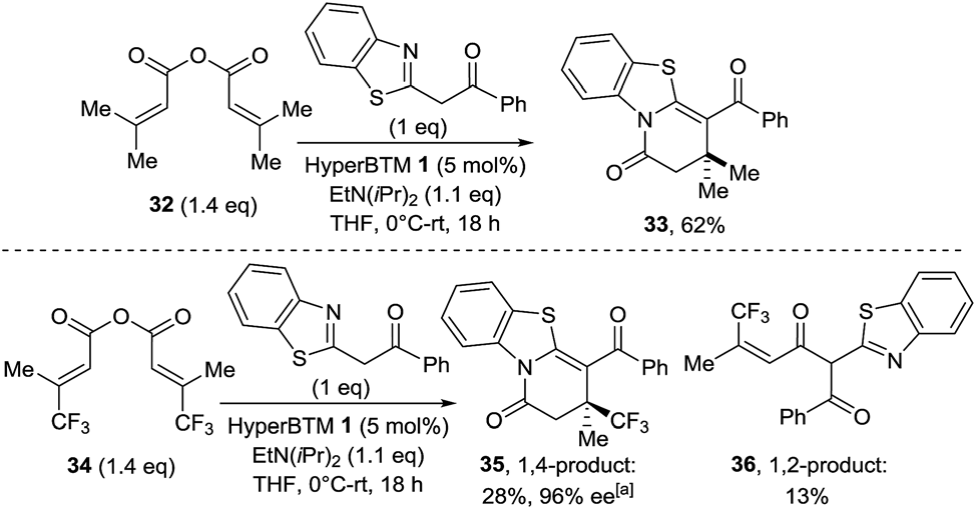
Generation of all-carbon quaternary centres.^a^

Excited by the high chemo- and enantiocontrol observed, the scope of this process was expanded to the synthesis of challenging all-carbon quaternary centers (Scheme 4). Trisubstituted homoanhydrides were used as α,β-unsaturated acyl ammonium precursors, allowing limited access to stereogenic quaternary centers for the first time in this methodology. Initial studies employed 3-methylbut-2-enoic anhydride **32** and gave the expected achiral lactam product **33** in good yield (Scheme 4).

Unfortunately, when (2*E*)-3-phenylbut-2-enoic anhydride was examined under the same conditions, no reaction was observed. The use of (2*E*)-4,4,4-trifluoro-3-methylbut-2-enoic anhydride **34** proved compatible with this methodology,^18^ leading to cyclized lactam product **35** containing a stereogenic quaternary trifluoromethyl group in moderate yield but 96% ee. Notably no lactone products were observed in this annulation process, although 1,2-addition product **36** was isolated in 13% yield as a side-product.

### Scale-up and derivatizations

**Scheme 5:**
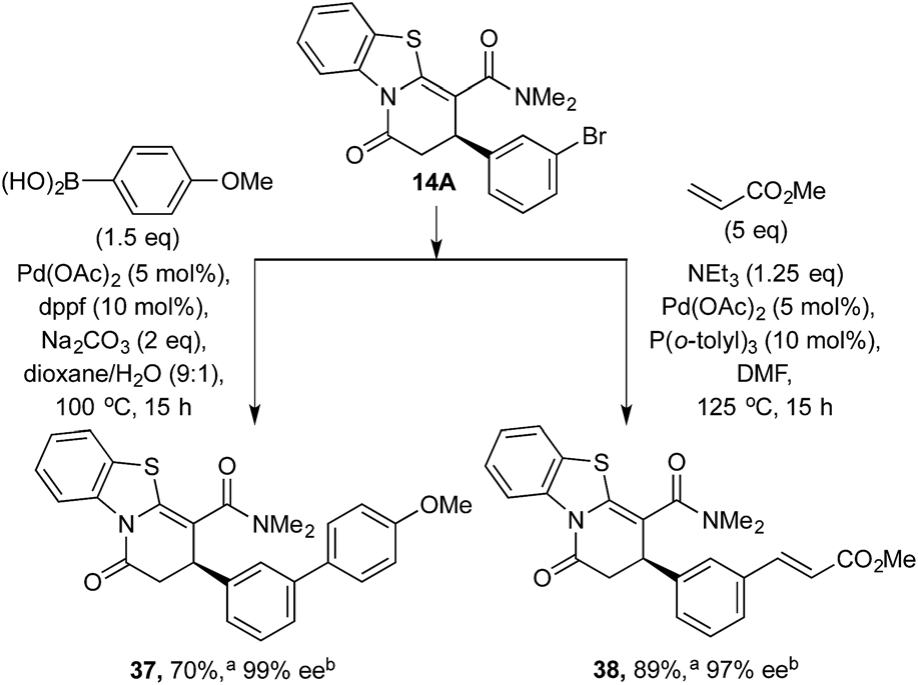
Product derivatization through cross coupling. ^a^ Isolated yield. ^b^ ee values obtained *via* chiral HPLC.

To demonstrate the potential further utility of the heterocyclic products obtained, scale-up and derivatization through palladium-catalyzed cross-coupling reactions was tested. 3-BrC_6_H_4_-substituted lactam **14A** was readily prepared on gram scale in high yield and enantioselectivity (1.15 g, 75%, 96% ee). Subjecting lactam **14A** to Suzuki coupling generated **37** in 70% yield with no erosion of enantioselectivity; similarly, Heck reaction of **14A** with methyl acrylate afforded **38** in 89% yield and 97% ee (Scheme 5).

### Computational details and mechanism

**Fig. 1:**
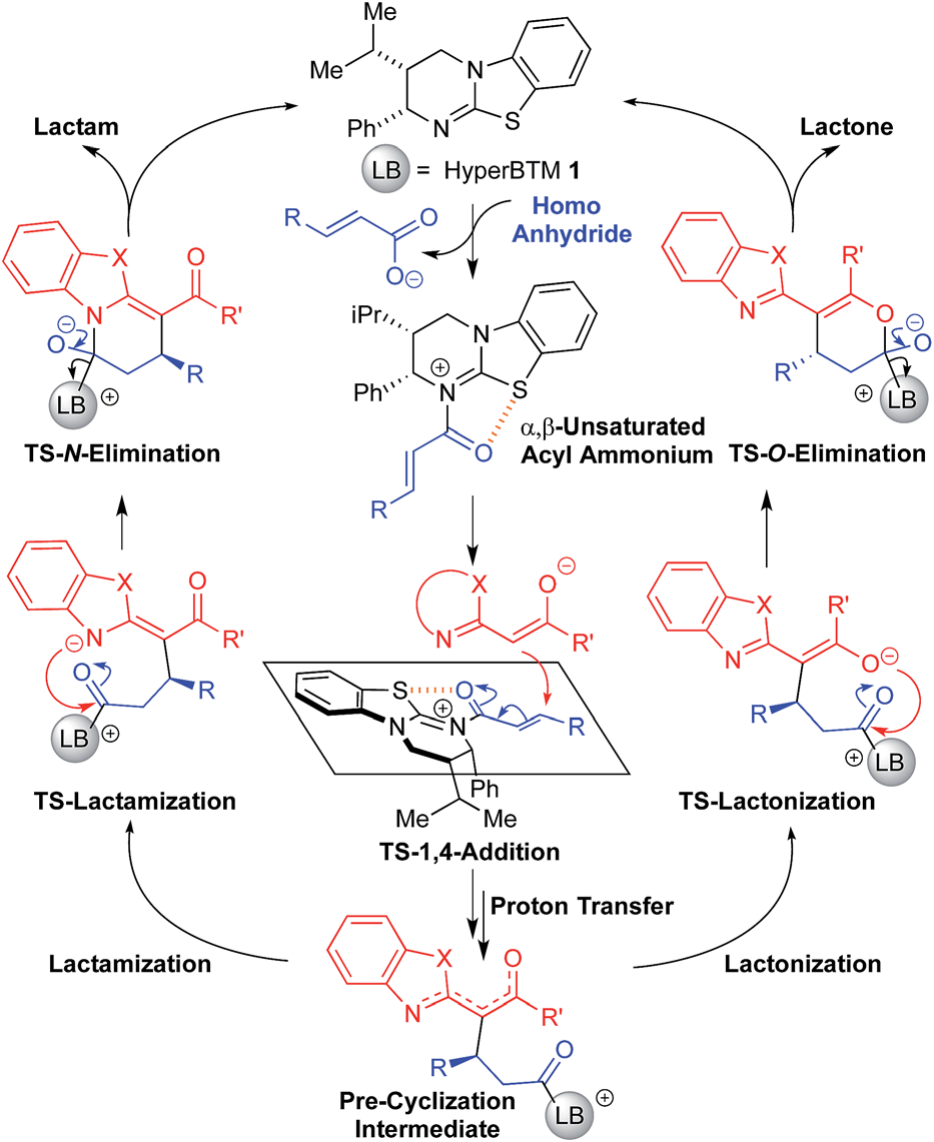
Catalytic cycle of the isothiourea-catalyzed annulations between 2-acyl benzazoles and homoanhydrides to form the lactam (left) or the lactone (right) using benzothiazole (X = S) or benzoxazole (X = O) derivatives, respectively.

Computations were undertaken to provide insight into the observed chemoselectivity when using the benzoxazole and benzothiazole nucleophiles (X = O or S, respectively). For this purpose, we have specifically computed the intermediates and transition structures involved in the formation of products **4A** (lactam) and **4B** (lactone) using 2-phenacylbenzothiazole, and **5B** (lactone) using 2-phenacylbenzoxazole. All energy refinements and geometries were computed in solution using the implicit polarizable continuum model PCM with tetrahydrofuran as solvent (M06-2X/6-31+G(d,p)/PCM(THF)//M06-2X/6-31G(d)/PCM(THF)^19^).^20^ The M06-2X DFT method has been successfully used to rationalize mechanisms and selectivities of synthetic reactions by us and others.^21^ Given the zwitterionic nature of many of the intermediates in the reaction, we also took into account the ability of M06-2X to accurately evaluate dispersion-heavy and ionic systems relative to the less computationally expensive B3LYP method.^22^ The catalytic cycle is shown in Fig. 1.^23^ Stepwise *N*-acylation of HyperBTM leads to the α,β-unsaturated acyl ammonium intermediate. Stereodetermining 1,4-addition of the anionic benzazole nucleophile and proton transfer leads to the pre-cyclization intermediate, which can either lactamize or lactonize. Restoration of the carbonyl π-bond releases the product and regenerates HyperBTM **1**.

#### S···O interaction

Considering this mechanistic scheme, within all key reactive intermediates and transition states where S- and O-atoms contain 1,5-connectivity (such as from the carbonyl C=O and isothiourea S within the acylammonium intermediate, or 2-phenacylbenzothiazole carbonyl-O and benzothiazole-S), these atoms are co-planar. The internuclear distances (within the range of 2.53–2.70 Å) are significantly less than the sum of the van der Waals radii (3.4 Å).^24^ These observations are consistent with an attractive force between the S- and O-atoms and in line with previous computations by Tantillo and Romo^13*b*^ as well as by Houk and Birman.^13*c*^ Unique to this system, however, is how this interaction dominates the structural preorganization of all key reactive intermediates and transition states of this annulation process.

#### S···O interaction in the enantiocontrol of 1,4-addition

**Fig. 2:**
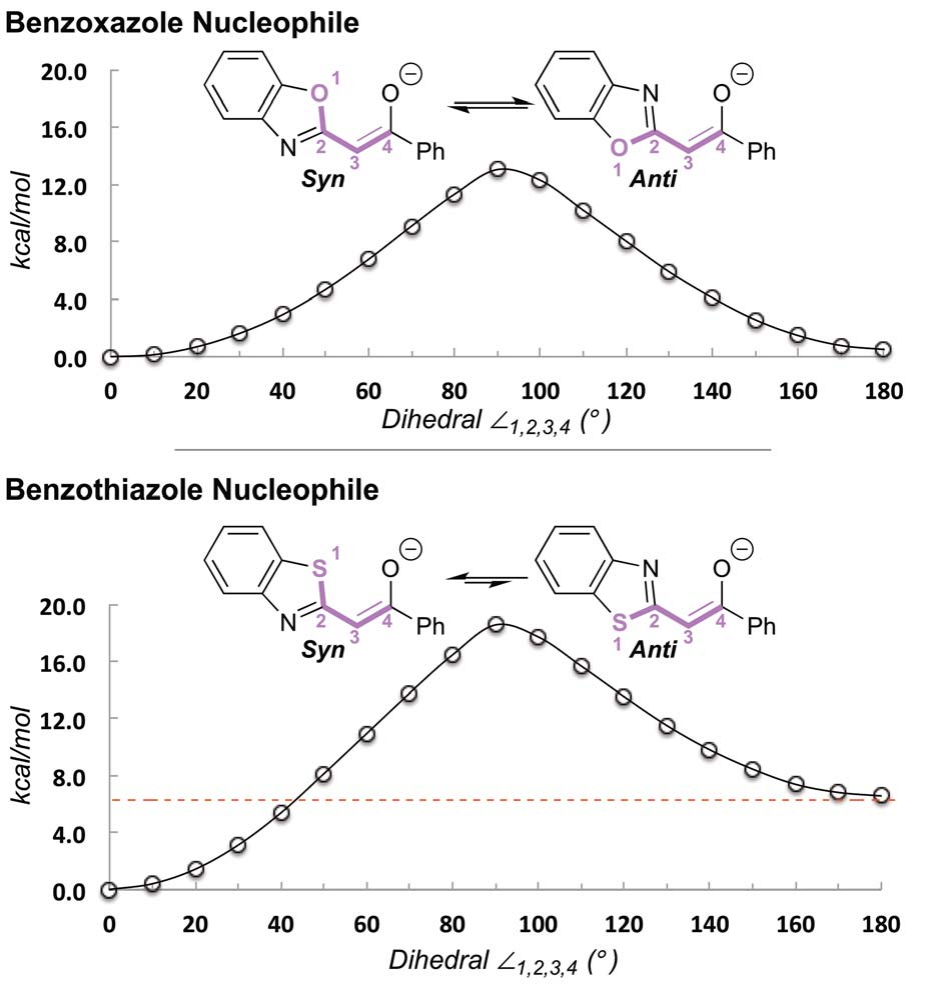
Conformational preferences of anionic benzoxazole and benzothiazole nucleophiles.^25^

All stable conformations of the α,β-unsaturated acyl ammonium intermediate exhibit coplanarity of the 1,5-O and S atoms. This is corroborated by the crystal structure of this intermediate which show the S–O being coplanar at a distance of 2.48 Å.^10*a*^ In addition, both anionic nucleophiles prefer the planar arrangement (Fig. 2), with the 1,5-S–O *syn* conformation favored by ∼7 kcal mol^–1^ in the case of benzothiazole. Taken together, these factors rigidify and planarize both the electrophilic α,β-unsaturated acyl ammonium intermediate and the incoming nucleophile, dramatically simplifying the stereochemical model. Nucleophilic 1,4-addition occurs *anti* to the catalyst stereodirecting groups on the less hindered face. The computed enantioselectivities of 99% in both cases are in reasonable agreement with experiments (83% and 98% ee for **4A** and **5B**, respectively, Scheme 3).

#### Lactamization *vs.* lactonization

**Fig. 3:**
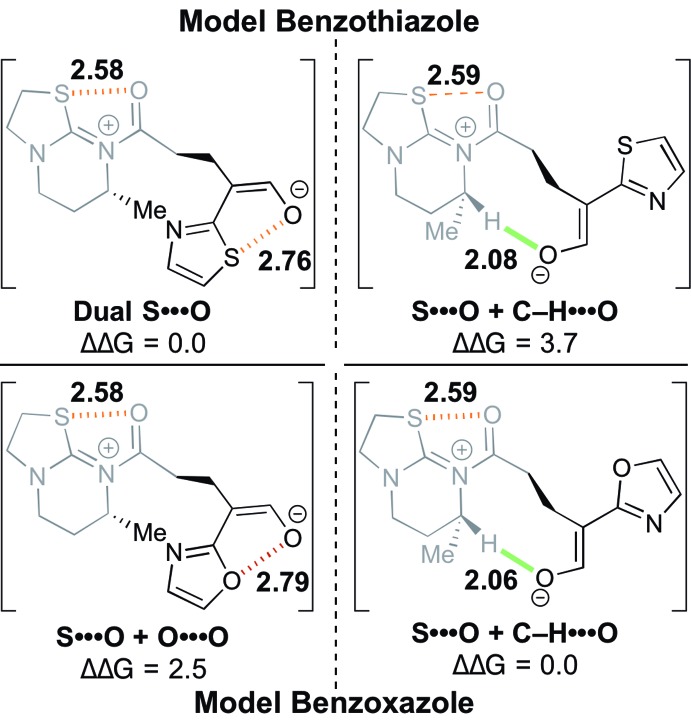
Model systems probing the relative energetic values (in kcal mol^–1^) between S···O and C–H···O nonbonding interactions.^26^

The interplay between S···O and C–H···O interactions^26^ (between the anionic nucleophile atoms and C–H α-to the positively-charged nitrogen of the acylated HyperBTM) governs cyclization chemoselectivity. Fig. 3 shows computed model complexes analogous to the pre-cyclization intermediate, featuring truncated simplified structures of both HyperBTM catalyst and benzazole nucleophiles. In the oxazole model system, the conformation with one S···O and one C–H···O interaction is favored by 2.5 kcal mol^–1^ over the conformation featuring the unfavorable O···O. However, in the thiazole model, the conformation featuring two S···O interactions, rather than one S···O and one C–H···O, is preferred by 3.7 kcal mol^–1^.

**Fig. 4:**
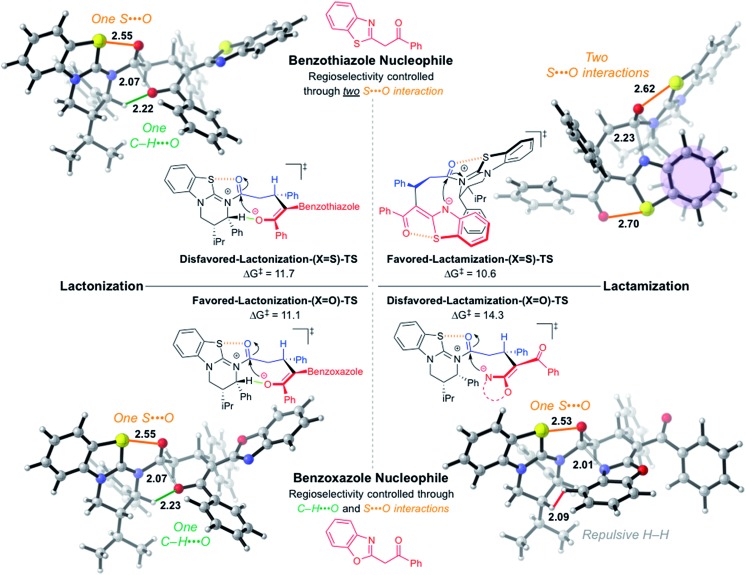
Computed chemoselectivity determining cyclization transition structures for benzoxazole and thiazole nucleophiles. All transition structures are stepwise (tetrahedral intermediate formation followed by HBTM release) except for favored-lactamization-(X = S)-TS (see ESI† for computed reaction coordinates). Forming bonds shown in grey. S···O interactions shown in orange, C–H···O highlighted in green, and van der Waals repulsion shown in red. Aromatic interaction shaded in purple. Relative energy values given in kcal mol^–1^. Structure images rendered using CYLview.^28^

These preferences carry over to the cyclization transition structures (Fig. 4). In the benzoxazole case, both annulations occur *via* a boat-like six-membered transition structure *anti* to the catalyst stereodirecting groups (phenyl and isopropyl) to minimize steric occlusion. The **Favored-Lactonization-(X = O)-TS** is preferred over the **Disfavored-Lactamization-(X = O)-TS** (Δ*G* ^‡^ = 11.1 and 14.3 kcal mol^–1^, respectively) due to a stabilizing C–H···O involving the *ortho* C–H of the catalyst and the incoming oxygen atom. In the latter, a β-C–H is involved in a repulsive interaction with the incoming benzoxazole. The computed selectivity of 99 : 1 matches well with the experimental selectivity of 98 : 2 seen with lactone **5B**.

The benzothiazole lactone closure occurs exactly as the benzoxazole case through the **Disfavored-Lactonization-(X = S)-TS** (Δ*G* ^‡^ = 11.7 kcal mol^–1^). The **Favored-Lactamization-(X = S)-TS** has a lower barrier (Δ*G* ^‡^ = 10.6 kcal mol^–1^), and the computed selectivity of 88 : 12 matches experiments. Interestingly, lactamization occurs on the same face as the catalyst stereodirecting groups, previously thought to be disfavored due to the steric occlusion.

Two key stabilizing interactions are present in benzothiazole lactamization that are not found in lactonization: (1) π-stacking of the catalyst phenyl and the fused benzene of the benzothiazole ring,^27^ and (2) a second 1,5-S···O interaction within the former benzothiazole nucleophile. The switch in chemoselectivity in favor of lactam formation using the benzothiazole is attributed to the penalty of breaking the 1,5-S···O present within the benzothiazole nucleophile for the lactonization process to proceed.

## Conclusions

To conclude, we have demonstrated the scope and limitations of the organocatalytic enantioselective functionalization of a range of benzazole nucleophiles using the isothiourea HyperBTM **1** and α,β-unsaturated homoanhydrides as α,β-unsaturated acyl ammonium precursors. The chemoselectivity observed during the cyclization is influenced by the nature of the benzazole and the carbonyl employed within the acylbenzazole, with benzothiazole preferentially using the ring-nitrogen to extrude the catalyst, whereas the benzoxazole moiety prefers to cyclize through the β-carbonyl substituent. Computations elucidated the importance of non-covalent 1,5-S···O interactions in determining the chemoselectivity within these processes. Specifically, the use of benzothiazole nucleophiles allows two stabilizing 1,5-S···O interactions in the preferred lactamization transition structure, while benzoxazole contains one stabilizing 1,5-S···O and one C–H···O interaction in the lactonization transition structure. Future research within our laboratories is aimed at harnessing the collaboration between theory and experiments towards the development of isothiourea Lewis base catalysts in new enantioselective transformations.
