# Supplementary material for: Non-bonding 1,5-S···O interactions govern chemo- and enantioselectivity in isothiourea-catalyzed annulations of benzazoles
Source: Chem Sci. 2016 Jul 4;7(12):6919–27. doi: 10.1039/c6sc00940a (PMC5450589; doi:10.1039/c6sc00940a)
Supplement: Supplementary file 1 [file SC-007-C6SC00940A-s001.pdf]

Electronic Supplementary Material (ESI) for Chemical Science.
This journal is © The Royal Society of Chemistry 2016

Non-Bonding 1,5-S•••O Interactions Govern Chemo- and Enantioselectivity in Isothiourea-Catalyzed Annulations of Benzazoles

Emily R. T. Robinson, Daniel M. Walden, Charlene Fallan, Mark D. Greenhalgh, Paul Ha-Yeon Cheong, and Andrew D. Smith*,†*

† EaStCHEM, School of Chemistry, University of St Andrews
North Haugh, St Andrews, Fife, UK, KY16 9ST.
E-mail: ads10@st-andrews.ac.uk
Homepage: http://ch-www.st-andrews.ac.uk/staff/ads/group/

Supporting Information: Synthetic Chemistry

Contents **Page**

General Information S2
Preparation of α,β-Unsaturated Carboxylic Acids S3
Preparation of α,β-Unsaturated Homoanhydrides S6
Preparation of Acyl Benzazoles S12
Asymmetric Annulation Products S14
Isomerisation and Epimerisation Experiments S48
HPLC Traces S53
NMR Spectra S97
References S197

General Information

Reactions involving moisture sensitive reagents were carried out under a nitrogen atmosphere using standard vacuum line techniques and anhydrous solvents. All glassware was flame dried and cooled under vacuum. For moisture sensitive reactions, solvents (THF, CH_2Cl_2, toluene, hexane and Et_2O) were obtained anhydrous and purified by an alumina column (Mbraun SPS-800). Petrol is defined as petroleum ether 40-60 °C. All other solvents and commercial reagents were used as supplied without further purification unless stated otherwise. Room temperature (rt) refers to 20-25 °C. Temperatures of 0 °C and −78 °C were obtained using ice/water and CO_2(s)/acetone baths respectively. Temperatures of 0 °C to −50 °C for overnight reactions were obtained using an immersion cooler (HAAKE EK 90). Reflux conditions were obtained using a DrySyn heating mantle equipped with a contact thermometer. *In vacuo* refers to the use of a rotary evaporator with a vacuum controller. Analytical thin layer chromatography was performed on pre-coated aluminium plates (Kieselgel 60 F_{254} silica). TLC visualisation was carried out with ultraviolet light (254 nm). Flash column chromatography was performed on Kieselgel 60 silica in the solvent system stated. ^{1}H, ^{13}C and ^{19}F nuclear magnetic resonance (NMR) spectra were acquired on either a Bruker Avance 300 (300 MHz ^{1}H, 75 MHz ^{13}C{1H}, 282 MHz ^{19}F{1H}), Bruker Avance II 400 (400 MHz ^{1}H, 100 MHz ^{13}C{1H}, 376 MHz ^{19}F{1H}) or a Bruker Avance II 500 (500 MHz ^{1}H, 125 MHz ^{13}C{1H}, 470 MHz ^{19}F{1H}) spectrometer at ambient temperature in the deuterated solvent stated. All chemical shifts are quoted in parts per million (ppm) relative to the residual solvent as the internal standard. All coupling constants, *J*, are quoted in Hz. Multiplicities are indicated by: s (singlet), d (doublet), t (triplet), q (quartet), m (multiplet), dd (doublet of doublets), ddd (doublet of doublet of doublets, dt (doublet of triplets), dq (doublet of quartets) and td (triplet of doublets). The abbreviation Ar is used to denote aromatic, Ph to denote phenyl, Bn to denote benzyl, br to denote broad and *app* to denote apparent. NMR peak assignments were confirmed using 2D 1H correlated spectroscopy (COSY), 2D 1H-13C heteronuclear multiple-bond correlation spectroscopy (HMBC), and 2D 1H-13C heteronuclear single quantum coherence (HSQC) where necessary. Infrared spectra (ν_{max}/cm^{-1}) were recorded on a Shimadzu IRAffinity-1 using a Pike attenuated total reflectance (ATR) accessory. Only the characteristic peaks are quoted. Melting points were recorded on an Electrothermal 9100 melting point apparatus and are uncorrected. *Dec* refers to decomposition. HPLC analyses were obtained on a Shimadzu HPLC consisting of a DGU-20A5 degasser, LC-20AT liquid chromatograph, SIL-20AHT autosampler, CMB-20A communications bus module, SPD-M20A diode array detector and a CTO-20A column oven

which allowed the temperature to be set from 25-40 °C. Separation was achieved using DAICEL CHIRALCEL OD-H and OJ-H columns or DAICEL CHIRALPAK AD-H, AS-H and IC columns. All chiral HPLC traces were compared to the authentic racemic trace prepared in analogous fashion. GC analyses were obtained on a Shimadzu GC consisting of a Shimadzu AOC-20i auto injector and a Shimadzu GC-2025 gas chromatograph. Analysis was performed using Shimadzu GCsolution v2.41 software and separation was achieved using the column described. Mass spectrometry (*m/z*) data were acquired by electrospray ionisation (ESI), chemical ionisation (CI), electron impact (EI), atmospheric solids analysis probe (ASAP), atmospheric pressure chemical ionization (APCI) or nanospray ionisation (NSI) either at the University of St Andrews or the EPSRC National Mass Spectrometry Service Centre, Swansea. At the University of St Andrews, low and high resolution ESI-MS were carried out on a Micromass LCT spectrometer. At the EPSRC National Mass Spectrometry Service Centre, low resolution NSI-MS was carried out on a Micromass Quattro II spectrometer and high resolution NSI-MS on a Thermofisher LTQ Orbitrap XL spectrometer. Optical rotations were measured on a Perkin Elmer Precisely/Model-341 polarimeter or Optical Activity AA-1000 polarimeter, operating at the sodium D line with a 100 mm path cell at rt.

Preparation of α,β-Unsaturated Carboxylic Acids

General Procedure A: Knoevenagel Condensation

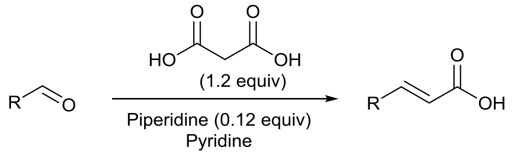

To a solution of the corresponding aldehyde (1 equiv) in pyridine (1.5 M), was added malonic acid (1.2 equiv) and piperidine (0.12 equiv) at room temperature. The reaction mixture was heated at 85 ˚C for 18 h. After cooling to room temperature the solution was acidified using 6 M HCl affording the carboxylic acid as a precipitate which was isolated by filtration. The acid was purified by column chromatography or recrystallisation as required.

(*E*)-3-Bromocinnamic acid (S1)

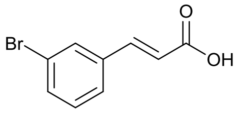

The title compound was prepared according to *General Procedure A* from 3-bromobenzaldehyde (3.49 mL, 30.0 mmol), malonic acid (3.75 g, 36.0 mmol) and piperidine (0.35 mL, 3.60 mmol) in pyridine (20 mL) to give the *carboxylic acid* **S1** as a white solid which was washed with hexane; no further purification needed (6.45 g, 95%); mp 176-178 ˚C {Lit.[1] 175-176.3 °C}; δ_H (300 MHz, DMSO-d_6) 6.61 (1H, d, *J* 16.0, ArCH=C*H*), 7.37 (1H, t, *J* 7.9, C(5)*H*), 7.56 (1H, d, *J* 16.1, ArC*H*=CH), 7.57 – 7.62 (1H, m, C(4)*H*), 7.71 (1H, dt, *J* 7.8, 1.3, ArC(6)*H*), 7.94 (1H, t, *J* 1.8, ArC(2)*H*), 12.52 (1H, s, COO*H*). Data in agreement with the literature.[1]

(*E*)-2-Bromocinnamic acid (S2)

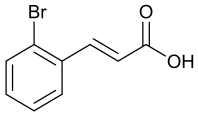

The title compound was prepared according to *General Procedure A* from 2-bromobenzaldehyde (3.49 mL, 30.0 mmol), malonic acid (3.75 g, 36.0 mmol and piperidine (0.35 mL, 3.60 mmol) in pyridine (20 mL) to give the *carboxylic acid* **S2** as a white solid which was washed with hexane; no further purification needed (6.78 g, 99%); mp 217-219 ˚C {Lit.[2] 217.5-218.5 °C}; δ_H (300 MHz, DMSO-d_6) 6.57 (1H, d, *J* 15.9, ArCH=C*H*), 7.32-7.39 (1H, m, *J* 7.6, 1.8, ArC(5)*H*), 7.40 – 7.47 (1H, m, ArC(4)*H*), 7.71 (1H, dd, *J* 7.9, 1.4, ArC(3)*H*), 7.84 (1H, d, *J* 15.9, ArC*H*=CH), 7.90 (1H, dd, *J* 7.8, 1.8ArC(6)*H*), 12.64 (1H, br, s, COO*H*). Data in agreement with the literature.[2]

(2*E*)-3-(Furan-3-yl)prop-2-enoic acid (S3)

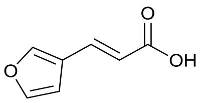

The title compound was prepared according to *General Procedure A* from furan-3-carbaldehyde (2.59 mL, 30.0 mmol), malonic acid (3.75 g, 36.0 mmol) and piperidine (0.35 mL, 3.60 mmol) in pyridine (20 mL) to give the *carboxylic acid* which was recrystallised from EtOAc/hexane to give **S3** as a brown solid (2.51 g, 61%); mp 151-152 ˚C {Lit.[3] 152.5-154}; δ_H (500 MHz, d_4-MeOD) 6.20 (1H, d, *J* 15.8, ArCH=C*H*), 6.75 (1H, d, *J* 1.9,

ArC(4)*H*), 7.52-7.56 (1H, m, ArC(5)*H*), 7.59 (1H, d, *J* 15.8, ArC*H*=CH), 7.80-7.85 (1H, m, ArC(2)*H*). Data in agreement with the literature.[3,4]

(2*E*)-3-(Thiophen-3-yl)prop-2-enoic acid (S4)

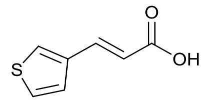

The title compound was prepared according to *General Procedure A* from thiophene-3-carbaldehyde (2.59 mL, 30.0 mmol), malonic acid (3.75 g, 36.0 mmol) and piperidine (0.35 mL, 3.60 mmol) in pyridine (20 mL) to give the *carboxylic acid* **S4** as an off-white solid, no further purification needed (4.33 g, 94%); mp 148-150 ˚C {Lit.[5] 151 ˚C}; δ_H (300 MHz, $CDCl_3$) 6.27 (1H, d, *J* 15.9, ArCH=C*H*), 7.32 (1H, ddd, *J* 5.1, 1.4, 0.4, ArC(4)*H*), 7.36 (1H, ddd, *J* 5.1, 2.9, 0.6, ArC(5)*H*), 7.56 (1H, dd, *J* 2.9, 1.3, ArC(2)*H*), 7.78 (1H, d, *J* 15.9, ArC*H*=CH). Data in agreement with the literature.[5,6]

(2*E*)-4,4,4-Trifluoro-3-methylbut-2-enoic acid (S5)

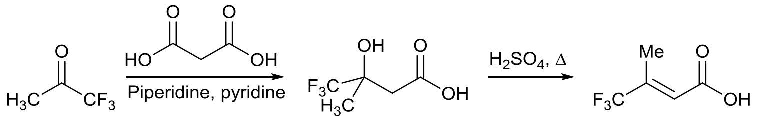

Procedure from Tarrant and Taylor.[7] Malonic acid (12.5 g, 120 mmol) and piperidine (1.19 mL, 12.0 mmol) were added to pyridine (50 mL) and cooled to 0 ˚C. 1,1,1-Trifluoromethylketone (8.96 mL, 100 mmol) was added to the flask by syringe addition directly into the pyridine solution to minimise loss through ketone volatility. After 1 h stirring at 0 ˚C the flask was then warmed to room temperature and stirred for 16 h. The flask was then heated to 90 ˚C for 24 h, followed by 130 ˚C for 4 h. The flask was cooled to room temperature and acidified using a 6 M solution of hydrochloric acid. The aqueous layer was washed with ethyl acetate (50 mL × 3) and the combined organic layers dried over $MgSO_4$, filtered and concentrated *in vacuo* to afford the β-hydroxy acid which was used without further purification. The hydroxy acid was added to a solution of concentrated sulfuric acid (50 mL) in water (50 mL) at room temperature. The flask was attached to distillation apparatus and heated slowly to 200 ˚C under atmospheric pressure, with a mixture of water and crude α,β-unsaturated acid collected in the receiver flask. After heating for 16 h at 200 ˚C the mixture in the receiver flask was washed with CH_2Cl_2 (50 mL × 3), and the combined organic layers dried over $MgSO_4$, filtered and concentrated *in vacuo* to afford the crude acid which was purified by vacuum distillation (<1 mbar) at 70 ˚C to give acid **S5** as a colourless oil (4.18 g, 31%, E:Z 89:11 confirmed by $^1H \rightarrow ^{19}F$ NOE); bp 158-163 ˚C {Lit. 160-166 ˚C};

δ_H (500 MHz, $CDCl_3$) 1.67 (3H, d, *J* 1.1, (*Z*)-C*H*$_3$), 2.28 (3H, d, *J* 1.7, (*E*)-C*H*$_3$), 6.36 (1H, h, *J* 1.5, (*E*)-C*H*), 6.52-6.55 (1H, m, (*Z*)-C*H*), 12.08 (1H, s, CO_2*H*); δ_F (471 MHz, $CDCl_3$) −82.95 (*Z*), −71.45 (*E*). Data in agreement with the literature.[7,8]

(2*E*)-3-Phenylbut-2-enoic acid (S6)

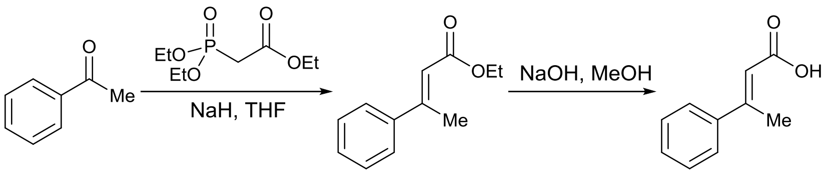

Modified procedure from Peters and Tiseni.[9] Triethylphosphonoacetate (19.8 mL, 100 mmol) was added dropwise to a suspension of sodium hydride (60% in mineral oil, 4.00 g, 100 mmol) in dry THF (100 mL) under nitrogen at 0 ˚C. The flask was stirred at 0 ˚C for 30 min then warmed to room temperature over 30 min. A solution of acetophenone (11.7 mL, 100 mmol) in dry THF (50 mL) was then added dropwise and the flask stirred at room temperature for 18 h. The reaction was quenched by careful addition of water at 0 ˚C then the mixture extracted with Et_2O (3 × 100 mL). The combined organic phase was washed with brine, dried over anhydrous $MgSO_4$ and concentrated *in vacuo* to afford the crude ester which was used without further purification. The crude oil was dissolved in methanol (150 mL) followed by addition of sodium hydroxide (16.8 g, 300.0 mmol) and water (50 mL). The flask was stirred at room temperature for 48 h then quenched by acidification with 6 M HCl. The mixture was concentrated *in vacuo* then extracted with CH_2Cl_2 (3 × 100 mL). The combined organic layers were dried over anhydrous $MgSO_4$, filtered and concentrated *in vacuo* to afford the crude acid. Purification by recrystallisation from ethanol/water afforded acid **S6** as a white solid (5.71 g, 35%); mp 93-94 °C {Lit.[10] 91-92 °C}; δ_H (300 MHz, $CDCl_3$) 2.62 (3H, d, *J* 1.3, C*H*$_3$), 6.19 (1H, q, *J* 1.3, C=C*H*), 7.37-7.43 (3H, m, 2 × PhC(3)*H*, PhC(4)*H*), 7.44-7.57 (2H, m, 2 × PhC(2)*H*). Data in agreement with the literature.[10]

Preparation of α,β-Unsaturated Homoanhydrides

General Procedure B: Anhydride Synthesis

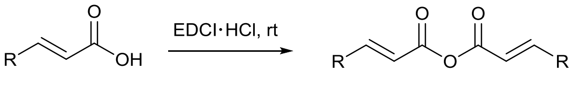

To a solution of carboxylic acid in CH_2Cl_2 or THF as specified, was added 1-ethyl-3-(3-dimethylaminopropyl)carbodiimide·HCl and the solution stirred for 1-2 h at room temperature. The solution was diluted with CH_2Cl_2 (50 mL) and then washed sequentially

with water (2 × 50 mL) and saturated aqueous $NaHCO_3$ solution (50 mL). The organic layer was dried over anhydrous $MgSO_4$, filtered, and concentrated *in vacuo* to afford the *homoanhydride*.

(*E*)-Cinnamic anhydride (S7)

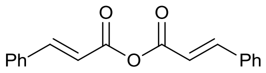

The title compound was prepared according to *General Procedure B* from (*E*)-cinnamic acid (741 mg, 5.00 mmol) and EDCI·HCl (959 mg, 5.00 mmol) in CH_2Cl_2 (6 mL) to give the *homoanhydride* **S7** as a white solid (448 mg, 64%); mp 118-120 °C {Lit.[11] 130 °C}; δ_H (400 MHz, $CDCl_3$) 6.54 (2H, d, *J* 16.0, ArCH=C*H*), 7.40–7.47 (6H, m, Ar*H*), 7.54–7.63 (4H, m, Ar*H*), 7.86 (2H, d, *J* 16.0, ArC*H*=CH). Data in agreement with the literature.[11,12]

(*E*)-3-(4-Methoxyphenyl)acrylic 3-(4-methoxyphenyl)propanoic anhydride (S8)

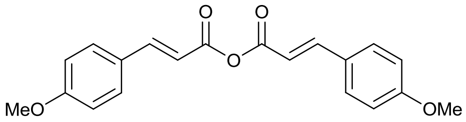

The title compound was prepared according to *General Procedure B* from 4-methoxycinnamic acid (5.34 g, 30.0 mmol) and EDCI·HCl (3.46 mg, 18.0 mmol) in CH_2Cl_2 (50 mL) to give the *homoanydride* **S8** as a white solid (2.89 g, 57%); mp 111-113 °C {Lit.[12] 116-119 °C}; δ_H (500 MHz, $CDCl_3$) 3.86 (6H, s, ArOCH_3), 6.39 (2H, d, *J* 15.8, ArCH=C*H*), 6.91-6.96 (4H, m, Ar*H*), 7.50-7.56 (4H, m, Ar*H*), 7.80 (2H, d, *J* 15.8, ArC*H*=CH). Data in agreement with the literature.[12]

(*E*)-3-(4-(Trifluoromethyl)phenyl)acrylic anhydride (S9)

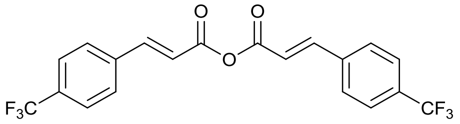

The title compound was prepared according to *General Procedure B* from 4-trifluoromethylcinnamic acid (6.48 g, 30.0 mmol) and EDCI·HCl (3.46 g, 18.0 mmol) in CH_2Cl_2 (100 mL) to give the *homoanhydride* **S9** as a white solid (2.79 g, 45%); mp 127-131 °C {Lit.[13] 127-128 °C}; δ_H (300 MHz, $CDCl_3$) 6.61 (2H, d, *J* 16.0, CH=C*H*CO), 7.70 (8H, s, Ar*H*), 7.88 (2H, d, *J* 16.0, C*H*=CHCO). Data in agreement with the literature.[13]

***(E)*-3-Bromocinnamic anhydride (S10)**

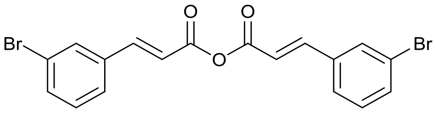

The title compound was prepared according to *General Procedure B* from (*E*)-3-bromocinnamic acid (7.95 g, 35.0 mmol) and EDCI·HCl (4.03 g, 21.0 mmol) in CH_2Cl_2 (50 mL) to give the *homoanhydride* **S10** as a white solid (4.75 g, 62%); mp 126-128 °C; ν_{max} (film)/cm^{-1} 3078 (C-H), 1755 (C=O), 1627 (C=C); δ_H (500 MHz, $CDCl_3$) 6.51 (2H, d, *J* 15.9, ArCH=C*H*), 7.31 (2H, t, *J* 7.9, ArC(5)*H*), 7.50 (2H, dt, *J* 7.8, 1.3 ArC(6)*H*), 7.57 (2H, ddd, *J* 8.0, 2.0, 1.0, ArC(4)*H*), 7.73 (2H, t, *J* 1.8, ArC(2)*H*), 7.77 (2H, d, *J* 15.9, ArC*H*=CH); δ_C (75 MHz, $CDCl_3$) 118.2 (2 × ArCH=*C*H), 123.4 (2 × Ar*C*(1)Br), 127.3 (2 × Ar*C*(4)H), 130.7 (2 × Ar*C*(5)H), 131.3 (2 × Ar*C*(6)H), 134.2 (2 × Ar*C*(2)H), 135.8 (2 × Ar*C*(2)), 147.1 (2 × Ar*C*H=CH), 162.0 (2 × *C*O); *m/z* (APCI$^+$) 211 ([M–$C_9H_6BrO_2$]$^+$, 100%), 437 ([M+H]$^+$, 6055%), 454 ([M+NH_4]$^+$, 75%); HRMS (APCI$^+$) $C_{18}H_{13}Br^{79}{}_2O_3$ ([M+H]$^+$) requires 434.9226, found 434.9223 (–0.7 ppm).

(*E*)-2-Bromocinnamic anhydride (S11)

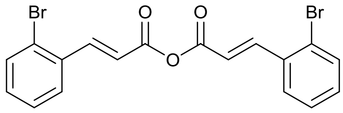

The title compound was prepared according to *General Procedure B* from (*E*)-2-bromocinnamic acid (1.14 g, 5.00 mmol) and EDCI·HCl (576 mg, 3.00 mmol) in CH_2Cl_2 (10 mL) to give the *homoanhydride* **S11** as a white solid (394 mg, 36%); mp 135-138 °C; ν_{max} (film)/cm^{-1} 2970 (C-H), 1759 (C=O), 1701 (C=O), 1624 (C=C); δ_H (500 MHz, $CDCl_3$) 6.49 (2H, d, *J* 15.9, ArCH=C*H*), 7.29 (2H, td, *J* 7.7, 1.7, ArC(3)*H*), 7.35-7.40 (2H, m, ArC(5)*H*), 7.66 (4H, ddd, *J* 9.1, 8.0, 1.5, ArC(4)*H*, ArC(6)*H*), 8.26 (2H, d, *J* 15.9, ArC*H*=CH); δ_C (126 MHz, $CDCl_3$) 119.4 (2 × ArCH=*C*H), 125.9 (2 × Ar*C*(1)Br), 127.9 (2 × Ar*C*(5)H), 128.1 (2 × Ar*C*(3)H), 132.2 (2 × Ar*C*(4)H), 133.7 (2 × Ar*C*(6)H), 133.7 (2 × Ar*C*(2)), 146.8 (2 × Ar*C*H=CH), 161.7 (2 × *C*O); *m/z* (APCI$^+$) 211 ([M–$C_9H_6BrO_2$]$^+$, 100%), 437 ([M+H]$^+$, 60%), 454 ([M+NH_4]$^+$, 80%); HRMS (APCI$^+$) $C_{18}H_{13}Br^{79}{}_2O_3$ ([M+H]$^+$) requires 434.9226, found 434.9221 (–1.1 ppm).

(*E*)-But-2-enoic anhydride (S12)

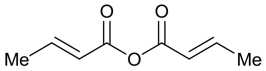

The title compound was prepared according to *General Procedure B* from crotonic acid (258 mg, 3.00 mmol) and EDCI·HCl (576 mg, 3.00 mmol) in CH_2Cl_2 (6 mL) to give the *homoanhydride* **S12** as a colourless oil (136 mg, 59%); δ_H (400 MHz, $CDCl_3$) 1.95 (6H, dd, *J* 7.0, 1.7, C*H*$_3$), 5.91 (2H, dq, *J* 15.5, 1.7, CH_3CH=C*H*), 7.14 (2H, dq, *J* 15.5, 7.0, CH_3C*H*=CH). Data in agreement with the literature.[14]

(*E*)-3-(Furan-2-yl)acrylic anhydride (S13)

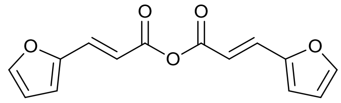

The title compound was prepared according to *General Procedure B* from furylacrylic acid (4.14 g, 30.0 mmol) and EDCI·HCl (3.46 mg, 18.0 mmol) in THF (50 mL) to give the *homoanhydride* **S13** as a brown solid (2.79 g, 72%); mp 70-72 °C{Lit.[13] 68-71 °C}; δ_H (500 MHz, $CDCl_3$) 6.38 (2H, d, *J* 15.6, CH=C*H*CO), 6.52 (2H, dd, *J* 3.5, 1.8, furanylC(4)*H*), 6.74 (2H, d, *J* 3.4, furanylC(3)*H*), 7.53-7.60 (4H, m, furanylC(5)*H*, C*H*=CHCO). Data in agreement with the literature.[13]

(2E)-3-(Furan-3-yl)prop-2-enoic anhydride (S14)

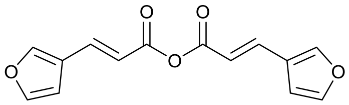

The title compound was prepared according to *General Procedure B* from (2*E*)-3-(furan-3-yl)prop-2-enoic acid (1.38 g, 10.0 mmol) and EDCI·HCl (1.15 g, 6.00 mmol) in THF (10 mL) to give the *homoanhydride* **S14** as a brown solid (0.87 g, 67%); mp 109-110 °C; ν_{max} (film)/cm^{-1} 3125 (C-H), 1775 (C=O), 1632 (C=C); δ_H (500 MHz, $CDCl_3$) 6.22 (2H, d, *J* 15.7, ArCH=C*H*), 6.63 (2H, d, *J* 2.0, ArC(4)*H*), 7.47 (2H, d, *J* 1.8, ArC(5)*H*), 7.73 (2H, s, ArC(2)*H*), 7.74 (2H, d, *J* 15.7, ArC*H*=CH); δ_C (75 MHz, $CDCl_3$) 107.5 (2 × furyl*C*(4)H), 116.6 (2 × ArCH=*C*H), 122.6 (2 × furyl*C*(3)), 138.7 (2 × furyl*C*(2)H), 145.0 (2 × furyl*C*(5)H), 145.9 (2 × Ar*C*H=CH), 162.6 (2 × *C*O); *m/z* (APCI$^+$) 121 ([M–$C_7H_5O_3$]$^+$, 100%), 259 ([M+H]$^+$, 35%), 276 ([M+NH$_4$]$^+$, 30%); HRMS (APCI$^+$) $C_{14}H_{11}O_5$ ([M+H]$^+$) requires 259.0601, found 259.0596 (–1.9 ppm).

(2E)-3-(Thiophen-3-yl)prop-2-enoic anhydride (S15)

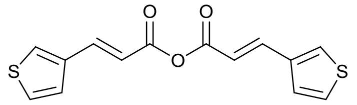

The title compound was prepared according to *General Procedure B* from (2*E*)-3-(thiophen-3-yl)prop-2-enoic acid (1.54 g, 10.0 mmol) and EDCI·HCl (1.15 g, 6.00 mmol) in THF (10 mL) to give the *homoanhydride* **S15** as a brown solid (1.16 g, 80%); mp 102-105 °C; ν_{max} (film)/cm^{-1} 3097 (C-H), 1771 (C=O), 1620 (C=C); δ_H (500 MHz, $CDCl_3$) 6.33 (2H, d, *J* 15.8, ArCH=C*H*), 7.34 (2H, dd, *J* 5.1, 1.3, ArC(4)*H*), 7.38 (2H, dd, *J* 5.2, 2.9, ArC(5)*H*), 7.61 (2H, dd, *J* 3.0, 1.3, ArC(2)*H*), 7.82 (2H, d, *J* 15.8, ArC*H*=CH); δ_C (126 MHz, $CDCl_3$) 116.4 (2 × ArCH=*C*H), 125.3 (2 × thiophene*C*(5)H), 127.6 (2 × thiophene*C*(4)H), 130.2 (2 × thiophene*C*(2)H), 137.1 (2 × thiophene*C*(3)), 142.0 (2 × Ar*C*H=CH), 162.9 (2 × *C*O); *m/z* (APCI$^+$) 137 ([M–$C_7H_5O_2S$]$^+$, 100%), 291 ([M+H]$^+$, 30%), 308 ([M+NH_4]$^+$, 55%); HRMS (APCI$^+$) $C_{14}H_{11}O_3S_2$ ([M+H]$^+$) requires 291.0144, found 291.0140 (–1.4 ppm).

***(E)*-4-Ethoxy-4-oxobut-2-enoic anhydride (S16)**

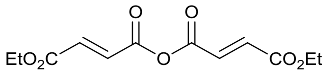

The title compound was prepared according to *General Procedure B* from *(E)*-4-ethoxy-4-oxobut-2-enoic acid (1.44 g, 10.0 mmol) and EDCI·HCl (1.15 g, 6.00 mmol) in CH_2Cl_2 (20 mL) to give the *homoanhydride* **S16** as a brown oil (795 mg, 59%); ν_{max} (film) /cm^{-1} 2986 (C-H), 1798 (C=O), 1721 (C=O); δ_H (500 MHz, $CDCl_3$) 1.33 (6H, t, *J* 7.2, C*H*$_3$), 4.29 (4H, q, *J* 7.2, C*H*$_2$CH$_3$), 6.87 (2H, d, *J* 15.7, CO$_2$EtCH=C*H*), 6.98 (2H, d, *J* 15.8, CO$_2$EtC*H*=CH); δ_C (75 MHz, $CDCl_3$) 14.2 (2 × *C*H$_3$), 62.0 (2 × C*H*$_2$), 131.4 (2 × EtO$_2$C-CH=*C*H), 137.5 (2 × EtO$_2$C-*C*H=CH), 159.7 (2 × =CH*C*O), 164 (2 × *C*O$_2$Et); *m/z* (APCI$^+$) 127 ([M–$C_6H_7O_4$]$^+$, 35%), 271 ([M+H]$^+$, 5%), 288 ([M+NH_4]$^+$, 100%); HRMS (APCI$^+$) $C_{12}H_{18}NO_7$ ([M+NH_4]$^+$) requires 288.1078, found 288.1074 (–1.3 ppm).

3-Methylbut-2-enoic anhydride (32)

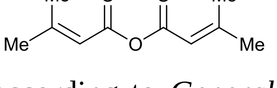

The title compound was prepared according to *General Procedure B* from 3-methylbut-2-enoic acid (2.00 g, 20.0 mmol) and EDCI·HCl (2.30 g, 12.0 mmol) in CH_2Cl_2 (50 mL) to give the *homoanhydride* **S17** as a colourless oil (0.71 g, 39%); δ_H (500 MHz, $CDCl_3$) 1.93

(3H, d, *J* 1.5, C*H*$_3$), 2.19 (3H, d, *J* 1.5, C*H*$_3$), 5.68 (1H, h, *J* 1.4, C=C*H*). Data in agreement with the literature.[15]

(2*E*)-3-Phenylbut-2-enoic anhydride (S17)

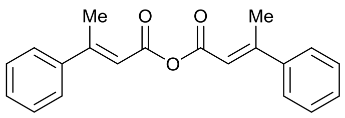

The title compound was prepared according to *General Procedure B* from (2*E*)-3-phenylbut-2-enoic acid (3.24 g, 20.0 mmol) and EDCI·HCl (2.30 g, 12.0 mmol) in CH_2Cl_2 (50 mL) to give the *homoanhydride* **S17** as a pale yellow oil (1.77 g, 58%); ν_{max} (film) /cm^{-1} 2922 (C-H), 2852 (C-H), 1771 (C=O), 1709 (C-O), 1609 (C=C); δ_H (500 MHz, $CDCl_3$) 2.66 (6H, d, *J* 1.3, C*H*$_3$), 6.19 (2H, d, *J* 1.3, C=C*H*), 7.38-7.44 (6H, m, PhC(2)*H*, PhC(4)*H*), 7.49-7.53 (4H, m, PhC(3)*H*); δ_C (126 MHz, $CDCl_3$) 18.8 (2 × *C*H$_3$), 115.9 (2 × C=*C*H), 126.6 (4 × Ar*C*(2)H), 128.8 (4 × Ar*C*(3)H), 129.9 (2 × Ar*C*(4)H), 141.7 (2 × Ar*C*(1)), 161.4 (2 × *C*O), 162.3 (2 × *C*=CH); *m/z* (APCI$^+$) 145 ([M–$C_{10}H_9O_2$]$^+$, 80%), 307 ([M+H]$^+$, 5%), 324 ([M+NH_4]$^+$, 10%), 318 (unknown product, 100%); HRMS (APCI$^+$) $C_{20}H_{19}O_3$ ([M+H]$^+$) requires 307.1329, found 307.1326 (–0.9 ppm).

(*E*)-4,4,4-Trifluoro-3-methylbut-2-enoic anhydride (34)

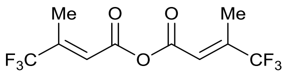

The title compound was prepared according to *General Procedure B* from (*E*)-4,4,4-trifluoro-3-methylbut-2-enoic acid (2.31 g, 15.0 mmol, *E*:*Z* = 89:11) and EDCI·HCl (1.73 g, 9.00 mmol) in CH_2Cl_2 (25 mL) to give the *homoanhydride* **34** as a pale yellow oil (1.33g, 60%). The product was used as a mixture without further purification (based on *E*:*Z* composition of the starting material, the product was assumed to be isolated as a statistical mixture of *E*,*E*:*E*,*Z*:*Z*,*Z* = 79:20:1). ν_{max} (film)/cm^{-1} 2642 (C-H), 1707 (C=O), 1292 (C-F); δ_H (500 MHz, $CDCl_3$) 2.32 (3H, d, *J* 1.6, 2 × C*H*$_3$), 6.33 (1H, dt, *J* 3.0, 1.6, 2 × C=C*H*); δ_C (126 MHz, $CDCl_3$) 13.1 (2 × *C*H$_3$), 119.63 (q, $^3J_{CF}$ 5.8, 2 × C=*C*H), 122.67 (q, $^1J_{CF}$ 274.8, 2 × *C*F$_3$), 147.78 (q, $^2J_{CF}$ 31.0, 2 × *C*=CH), 159.40 (2 × *C*O); δ_F (471 MHz, $CDCl_3$) -71.63; *m/z* (APCI$^+$) 137 ([M–$C_5H_4F_3O_2$]$^+$, 75%), 221 (unknown degradation peak, 100%), 291 ([M+H]$^+$, 75%); HRMS (APCI$^+$) $C_{10}H_9F_6O_3$ ([M+H]$^+$) requires 291.0450 found 291.0445 (–1.9 ppm).

Preparation of Acyl Benzazoles

General Procedure C: Benzazole Synthesis with NaHMDS

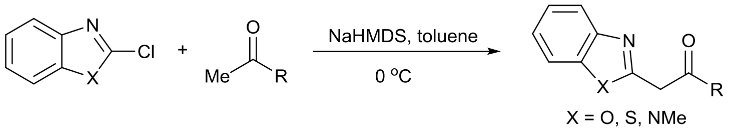

To a degassed solution of chlorobenzazole (1 equiv) and carbonyl nucleophile (3 equiv) in dry toluene was added NaHMDS (3.0 eq, 2 M solution in THF or 0.6 M solution toluene) dropwise at 0 °C, and the solution stirred for 5 h at 0 °C followed by room temperature for 16 h. Excess NaHMDS was quenched by dropwise addition of saturated aqueous NH_4Cl (50 mL) at 0 °C. The organic layer was separated, then the aqueous layer extracted with EtOAc. The combined organic layers were dried over $MgSO_4$, filtered, and concentrated *in vacuo.* The residue was as specified to afford the product.

2-Phenacylbenzothiazole (S18)

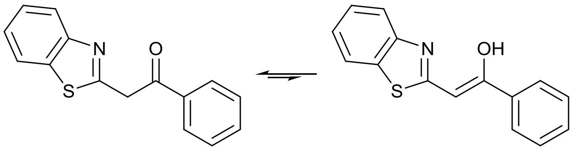

The title compound was prepared according to *General Procedure C* from 2-chlorobenzothiazole (2.62 mL, 20.0 mmol) and acetophenone (6.99 mL, 60.0 mmol) in anhydrous toluene (60 mL), with NaHMDS (30 mL, 2 M in THF, 12.0 mmol) and purified by trituration with cold hexane to give the *azaarylketone* **S18** as a yellow solid (4.76 g, 94%); mp 115-117 °C {Lit.[16] 113-114 °C}; δ_H (400 MHz, $CDCl_3$) 4.85 (2H, s, *keto*-CH_2COAr), 6.38 (1H, s, *enol*-C*H*COHAr), 7.31 (1H, t, *J* 7.6, *enol*-benzothiazoleC(6)*H*), 7.39 (1H, t, *J* 7.5, *enol*-benzothiazoleC(6)*H*), 7.42 – 7.52 (6H, m, Ar*H*), 7.51 (1H, t, *J* 7.7, *enol*-benzothiazoleC(5)*H*), 7.62 (1H, t, *J* 7.4, *keto*-benzothiazoleC(5)*H*), 7.79 (1H, d, *J* 7.9, *enol*-benzothiazoleC(4)*H*), 7.82 (1H, d, *J* 8.2, *enol*-benzothiazoleC(7)*H*)), 7.88 (3H, m, *keto*-benzothiazoleC(4)*H*, *enol*-phenacylC(2')*H*), 8.02 (1H, d, *J* 8.0, *keto*-benzothiazoleC(7)*H*), 8.10 (2H, d, *J* 7.5 *keto*-phenacylC(2')*H*). Data in agreement with the literature.[16]

2-(1,3-Benzothiazol-2-yl)-*N,N*-dimethylacetamide (S19)

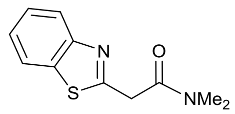

The title compound was prepared according to *General Procedure C* from 2-chlorobenzothiazole (521 μL, 4.00 mmol) and *N,N*-dimethylacetamide (1.12 mL, 12.0 mmol) in dry, degassed toluene (15 mL), with NaHMDS (6 mL, 2 M in THF, 12.0 mmol) to give the *azaarylamide* **S19** after aqueous work up as a white solid, no further purification required (875 mg, 99%); mp 98-100 °C {Lit.[17] 92-93 °C}; δ_H (300 MHz, $CDCl_3$) 2.88 (3H, s, NC*H*$_3$CH$_3$), 2.98 (3H, s, NC*H*$_3$CH$_3$), 4.11 (2H, s, C*H*$_2$CO), 7.24 (1H, ddd, *J* 8.4, 7.3, 1.2, benzothiazoleC(6)*H*), 7.34 (1H, ddd, *J* 8.1, 7.2, 1.2, benzothiazoleC(5)*H*), 7.74 (1H, ddd, *J* 7.9, 1.3, 0.6, benzothiazoleC(4)*H*), 7.88 (1H, ddd, *J* 8.2, 1.1, 0.6, benzothiazoleC(7)*H*). Data in agreement with the literature.[17]

***tert*-Butyl 2-(1,3-benzothiazol-2-yl)acetate (S20)**

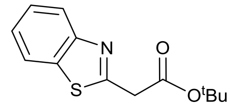

The title compound was prepared according to *General Procedure C* from 2-chlorobenzothiazole (521 μL, 4.00 mmol) and *tert*-butylacetate (1.61 mL, 12.0 mmol) in dry, degassed toluene (15 mL), with NaHMDS (20 mL, 0.6 M in toluene, 12.0 mmol) and purified by chromatography (10% hexane/CH_2Cl_2) to give the *azarylacetate* **S20** as an off-white solid (876 mg, 88%); mp 69-72 °C {Lit.[18] (oil)}; δ_H (300 MHz, $CDCl_3$) 1.50 (9H, s, C(C*H*$_3$)$_3$), 4.10 (2H, s, C*H*$_2$CO), 7.38 (1H, ddd, *J* 7.8, 7.5, 1.2, benzothiazoleC(6)*H*), 7.47 (1H, ddd, *J* 7.8, 7.5, 1.5, benzothiazoleC(5)*H*), 7.87 (1H, ddd, *J* 8.0, 1.2, 0.6, benzothiazoleC(4)*H*), 8.00 (1H, ddd, *J* 8.0, 1.2, 0.6, benzothiazoleC(7)*H*). Spectroscopic data in agreement with the literature.[18]

2-Phenacylbenzoxazole (S21)

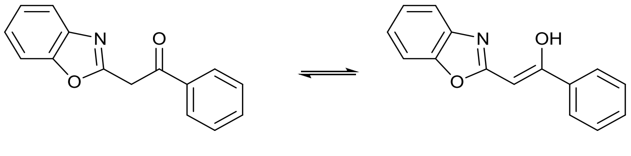

To a solution of 2-methylbenzoxazole (4.76 mL, 40.0 mmol), benzoyl chloride (20.4 mL, 120 mmol) in HPLC grade acetonitrile stored over molecular sieves (200 mL) was added NEt_3 (19.96 mL, 144 mmol) and the solution stirred at reflux for 18 h. The reaction was cooled and the actonitrile removed *in vacuo*. The crude product dissolved in CH_2Cl_2 (100 mL) and the

organic layer washed with saturated aqueous $NaHCO_3$ solution, dried over $MgSO_4$, filtered, and concentrated *in vacuo* to give the intermediate ester as a brown oil that was used without further purification. The crude ester was dissolved in methanol (100 mL) and solid KOH (5.6 g, 100.0 mmol) was added portionwise to the stirred solution. The reaction was then stirred at room temperature for 20 h. The methanol was removed *in vacuo* then the residue dissolved in CH_2Cl_2 and acidified using 2 M HCl. The aqueous layer was separated and the organic layer washed with saturated aqueous $NaHCO_3$, dried over $MgSO_4$, filtered and concentrated *in vacuo*. The crude was purified by column chromatography (5% EtOAc/petrol ether) followed by recrystallisation from ethanol to give the the *azaarylketone* **S21** as an off-white solid (6.24 g, 67%); mp 90-92 °C {Lit.[19] 87-89 °C}; δ_H (300 MHz, $CDCl_3$) 4.66 (2H, s, *keto*-CH_2-CO), 6.22 (1H, s, *enol*-C*H*=COH), 7.27-7.39 (4H, m, br, Ar*H*), 7.41-7.56 (7H, m, br, Ar*H*), 7.62 (2H, s, br, Ar*H*), 7.72 (1H, s, br, Ar*H*), 7.89 (2H, s, br, Ar*H*), 8.01-8.09 (2H, m, br, PhC(2)*H*). Data in agreement with the literature.[19]

Asymmetric Annulation Products

General Procedure D: Lactams and Lactones From Benzazoles

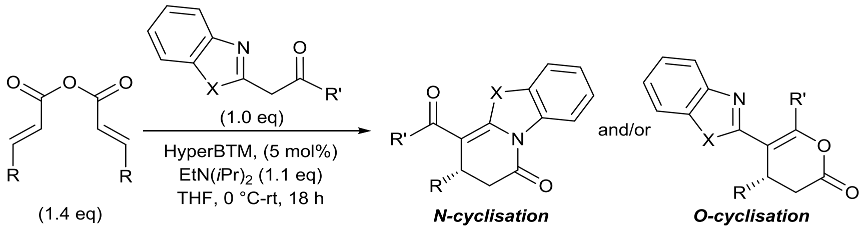

To a solution of the corresponding homoanhydride (1.4 equiv) in bench grade THF (0.36 M), was added benzazole (1.0 equiv), isothiourea (HyperBTM, 0.05 equiv) and EtN(*i*Pr)$_2$ (1.1 equiv) at 0 °C. The reaction mixture was stirred and gradually warmed to room temperature over 18 h. The solution was diluted with EtOAc and washed sequentially with 0.1 M HCl and saturated $NaHCO_3$ solution, dried over anhydrous $MgSO_4$, filtered and concentrated in vacuo. The residue was purified by column chromatography on silica gel to afford the product(s).

(*R*)-*N*,*N*-Dimethyl-1-oxo-3-phenyl-2,3-dihydro-1*H*-benzo[4,5]thiazolo[3,2-*a*]pyridine-4-carboxamide (2A)

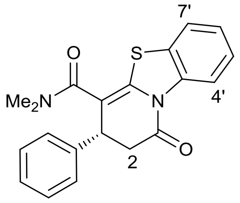

The title compound was prepared according to *General Procedure D* from (*E*)-cinnamic anhydride (70 mg, 0.25 mmol), and 2-(1,3-benzothiazol-2-yl)-*N*,*N*-dimethylacetamide (40 mg, 0.18 mmol), EtN(*i*Pr)$_2$ (35 μL, 0.20 mmol) and HyperBTM (2.8 mg, 0.05 mmol) in THF (0.5 mL) and purified by chromatography on silica gel (10% EtOAc/CH_2Cl_2) to give **2A** as an off-white foamy solid (44 mg, 70%); mp 71-74 °C; $[\alpha]_D^{20}$ –96.4 (*c* 0.5 in CH_2Cl_2); chiral HPLC analysis, ChiralPak AD-H (20% *i*-PrOH:hexane, flow rate 1.0 mL min^{-1}, 211 nm, 30 °C), t_R major: 17.1 min, t_R minor: 27.3 min, 96% ee; ν_{max} (film)/cm^{-1} 3061 (C-H), 2924 (C-H), 1703 (C=O), 1614 (C=C), 1584 (C=C), 1489 (C-N), 1385 (C-S), 1306 (C-O); δ_H (300 MHz, $CDCl_3$) 2.85 (6H, s, N(C*H*$_3$)$_2$), 2.98 (1H, dd, *J* 16.2, 5.9, C(2)*H*H), 3.19 (1H, dd, *J* 16.2, 7.1, C(2)H*H*), 4.21 (1H, t, *J* 6.5, C(3)*H*), 7.14-7.18 (1H, m, Ar*H*), 7.20-7.26 (4H, m, Ar*H*), 7.28-7.32 (3H, m, Ar*H*), 8.36 (1H, dd, *J* 8.1, 1.0, C(4')*H*); δ_C (126 MHz, $CDCl_3$) 37.0 (2 × N(*C*H$_3$)$_2$), 40.2 (*C*(2)H$_2$), 40.9 (*C*(3)H), 106.4 (*C*(4)), 117.6 (*C*(4')H), 121.4 (*C*(7')H), 125.5 (*C*(6')H), 126.1 (*C*(7a')), 126.4 (*C*(5')H), 126.9 (2 × C(3)Ph*C*(2)H), 127.6 (C(3)Ph*C*(4)H), 129.2 (2 × C(3)Ph*C*(3)H), 137.3 (*C*(4a')), 140.9 (C(3)Ph*C*(1)), 142.3 (*C*(5)), 167.8 (*C*(1)O), 169.4 (*C*ONMe$_2$); *m/z* (NSI$^+$) 373 ([M+Na]$^+$, 100%), 389 ([M+K]$^+$, 70%); HRMS (NSI$^+$) $C_{20}H_{18}O_2N_2NaS$ ([M+Na]$^+$) requires 373.0981, found 373.0974 (–1.9 ppm).

***tert*-Butyl (*R*)-1-oxo-3-phenyl-2,3-dihydro-1H-benzo[4,5]thiazolo[3,2-a]pyridine-4-carboxylate (3A)**

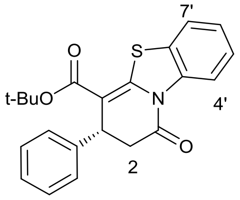

The title compound was prepared according to *General Procedure D* from (*E*)-cinnamic anhydride (278 mg, 1.00 mmol), and *tert*-butyl 2-(1,3-benzothiazol-2-yl)acetate (179 mg, 0.72 mmol), EtN(*i*Pr)$_2$ (0.14 mL, 0.80 mmol) and HyperBTM (11.1 mg, 0.036 mmol) in THF (2 mL) and purified by chromatography on silica gel (1:1→5:1 CH_2Cl_2/hexane) to give **3A** as

a yellow solid (174 mg, 64%); mp 105-110 ˚C; $[\alpha]_D^{20}$ –269.1 (*c* 1 in $CHCl_3$); chiral HPLC analysis, ChiralPak AD-H (2.5% *i*-PrOH:hexane, flow rate 1.0 mL min^{-1}, 254 nm, 30 °C), t_R major: 16.4 min, t_R minor: 11.0 min, 93% ee; ν_{max} (film)/cm^{-1} 2979 (C-H), 2928 (C-H), 1717 (C=O), 1668 (C=O), 1454 (C=C); δ_H (500 MHz, $CDCl_3$) 1.45 (9H, s, OC(CH_3)$_3$), 3.02 (1H, dd, *J* 16.3, 2.0, C(2)*H*H), 3.26 (1H, dd, *J* 16.3, 8.1, C(2)*H*H), 4.28 (1H, dd, *J* 8.3, 2.0, C(3)*H*), 7.18-7.33 (7H, m, Ar*H*), 7.42-7.47 (1H, m, C(7')*H*), 8.46 (1H, d, *J* 8.2, C(4')*H*); δ_C (126 MHz, $CDCl_3$) 28.4 (3 × OC(*C*H$_3$)$_3$), 37.4 (*C*(2)H), 40.3 (*C*(3)H$_2$), 81.3 (O*C*(CH$_3$)$_3$), 102.2 (*C*(4)), 117.5 (*C*(4')H), 121.5 (*C*(7')H), 125.6 (*C*(6')H), 126.5 (*C*(5')H), 126.7 (2 × C(3)Ar*C*(2)H), 127.2 (*C*(7a')), 127.3 (*C*(3)Ar*C*(4)H), 128.9 (2 × C(3)Ar*C*(3)H), 136.9 (*C*(4a')), 142.0 (C(3)Ar*C*(1)), 151.1 (*C*(5)), 166.1 (*C*(1)O), 168.5 (C(4)*C*O; *m/z* (NSI$^+$) 346 ([M–*t*Bu+Na]$^+$, 100%), 380 ([M+H]$^+$,20%), 402 ([M+Na]$^+$, 25%), 418 ([M+K]$^+$, 70%); HRMS (NSI$^+$) $C_{22}H_{21}O_3NS$ ([M+H]$^+$) requires 380.1315, found 380.1311 (–1.0 ppm).

(11*R*)-10-Benzoyl-11-phenyl-8-thia-1-azatricyclo[7.4.0.0^{2,7}]trideca-2,4,6,9-tetraen-13-one (4A) and (4*R*)-5-(1,3-benzothiazol-2-yl)-4,6-diphenyl-3,4-dihydro-2H-pyran-2-one (4B)

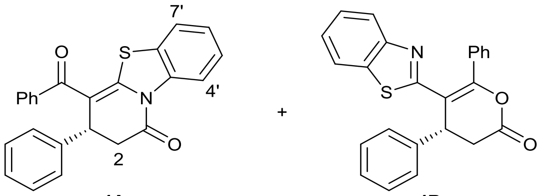

4A **4B**

The title compounds were prepared according to *General Procedure D* from (*E*)-cinnamic anhydride (700 mg, 2.50 mmol) and 2-phenacyl benzothiazole (455 mg, 1.80 mmol), EtN(*i*Pr)$_2$ (0.35 mL, 2.00 mmol) and HyperBTM (5.5 mg, 1 mol %) in THF (5 mL) and purified by chromatography on silica gel (10:2 CH_2Cl_2/hexane) to afford **4A** as a yellow solid (595 mg, 86%) and **4B** as a yellow solid (63 mg, 9%). The major isomer was suspended in Et_2O then recrystallised from EtOAc: crystals were obtained (68 mg, 10% overall yield, 4% ee) plus liquors which were concentrated *in vacuo* to give a yellow solid.

4A (major): (472 mg, 68% yield); mp 168-171 °C; $[\alpha]_D^{20}$ –148.5 (*c* 1.0 in $CHCl_3$); chiral HPLC analysis, ChiralPak AD-H (20% *i*-PrOH:hexane, flow rate 1 mL min^{-1}, 211 nm, 30 °C), t_R minor: 13.2 min, t_R major: 22.5 min, 97% ee; ν_{max} (film)/cm^{-1} 3024 (C-H), 2984 (C-H), 2913 (C-H), 1722 (C=O), 1597 (C=C), 1574 (C=C), 1477 (C-N), 1360 (C-S), 1269 (C-O); δ_H (500 MHz, $CDCl_3$) 3.05 (1H, dd, *J* 15.9, 2.2, C(12)*H*$_2$), 3.29 (1H, dd, *J* 15.9, 6.9, C(12)*H*$_2$), 4.38 (1H, dd, *J* 6.9, 2.2, C(11)*H*), 7.11 (2H, d, *J* 7.2, 2×C(11)PhC(2)*H*), 7.21-7.44 (10H, m, Ar*H*), 7.62 (1H, s, C(6)*H*), 8.47 (1H, d, *J* 7.8, C(3)*H*); δ_C (126 MHz, $CDCl_3$) 38.6

(*C*(11)H), 41.4 (*C*(12)H_2), 107.7 (*C*(10)), 117.5 (*C*(3)H), 122.0 (*C*(6)H), 125.9 (*C*(5)H), 126.8 (2×C(11)Ph*C*(2)H), 127.0 (2×C(10)COPh*C*(3)H, *C*(4)H), 127.6 (C(11)Ph*C*(4)H), 127.8 (*C*(7)), 128.1 (2×C(10)COPh*C*(2)H), 129.3 (2×C(11)Ph*C*(3)H), 130.3 (C(10)COPh*C*(4)H), 136.0 (*C*(2)), 139.4 (C(10)COPh*C*(1)), 140.8 (C(11)Ph*C*(1)), 156.2 (*C*(9)), 167.9 (*C*(13)O), 191.2 (C(10)*C*O); *m/z* (NSI^+) 384 ($[M+H]^+$, 60%); HRMS (NSI^+) $C_{24}H_{18}O_2NS$ ($[M+H]^+$) requires 384.1053, found 384.1052 (–0.2 ppm).

4B (minor): (63 mg, 9%); mp 192-194 °C; $[\alpha]_D^{20}$ –18.4 (*c* 1.0 in $CHCl_3$); chiral HPLC analysis, ChiralPak AD-H (20% *i*-PrOH:hexane, flow rate 1 mL min^{-1}, 211 nm, 30 °C), t_R major: 7.7 min, t_R minor: 10.8 min, 86% ee; ν_{max} (film)/cm^{-1} 2974 (C-H), 2372 (C-H), 1775 (C=O), 1647 (C=N), 1491 (C=C), 1435 (C=C), 1339 (C-S), 1271 (C-O); δ_H (300 MHz, $CDCl_3$) 3.08 (1H, dd, *J* 15.8, 1.6, C(3)H_2), 3.32 (1H, dd, *J* 15.8, 7.6, C(3)H_2), 5.03 (1H, dd, *J* 7.6, 1.6, C(4)*H*), 7.23-7.36 (6H, m, Ar*H*), 7.38-7.52 (3H, m, Ar*H*), 7.55-7.63 (4H, m, Ar*H*), 7.93 (1H, d, *J* 7.8, HetAr*H*); δ_C (75 MHz, $CDCl_3$) 36.9 (*C*(3)H_2), 41.3 (*C*(4)H), 115.1 (*C*(5)), 121.3 (C(5)HetAr*C*H), 123.1 (C(5)HetAr*C*H), 125.5 (C(5)HetAr*C*H), 126.1 (C(5)HetAr*C*H), 127.0 (2×C(4)Ph*C*(2)H), 127.8 (C(4)Ph*C*(4)H), 128.9 (2×C(6)Ph*C*(3)H), 129.3 (2×C(4)Ph*C*(3)H), 130.1 (2×C(6)Ph*C*(2)H), 130.8 (C(6)Ph*C*(4)H), 131.9 (C(6)Ph*C*(1)), 135.7 (C(5)HetAr*C*), 139.6 (C(4)Ph*C*(1)), 152.4 (C(5)HetAr*C*), 154.4 (*C*(6)), 164.2 (C(5)HetAr*C*=N), 166.6 (*C*(2)O); *m/z* (NSI^+) 384 ($[M+H]^+$, 60%); HRMS (NSI^+) $C_{24}H_{18}O_2NS$ ($[M+H]^+$) requires 384.1053, found 384.1052 (–0.2 ppm).

(*R*)-5-(Benzo[*d*]oxazol-2-yl)-4,6-diphenyl-3,4-dihydro-2*H*-pyran-2-one (5B)

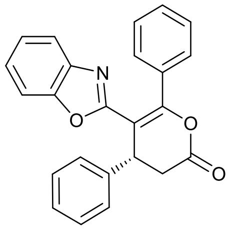

The title compound was prepared according to *General Procedure D* from (*E*)-cinnamic anhydride (70 mg, 0.25 mmol), and 2-phenacylbenzoxazole (43 mg, 0.18 mmol), EtN(*i*Pr)$_2$ (35 μL, 0.20 mmol) and HyperBTM (2.8 mg, 0.05 mmol) in THF (0.5 mL) and purified by chromatography on silica gel (CH_2Cl_2) to give **5B** as an off-white foamy solid (61 mg, 95%); mp 125-128 °C; $[\alpha]_D^{20}$ +28.8 (*c* 1.0 in CH_2Cl_2); chiral HPLC analysis, ChiralPak OJ-H (20% *i*-PrOH:hexane, flow rate 1.0 mL min^{-1}, 211 nm, 30 °C), t_R major: 13.6 min, t_R minor: 17.3 min, 98% ee; ν_{max} (film)/cm^{-1} 3061 (C-H), 1778 (C=O), 1690 (C=O), 1645 (C=N), 1530 (C=C); δ_H (500 MHz, $CDCl_3$) 3.12 (1H, dd, *J* 15.7, 1.4, C(3)*H*H), 3.31 (1H, dd, *J* 15.7, 7.6,

C(3)H*H*), 4.94 (1H, d, *J* 6.6, C(4)*H*), 7.17-7.20 (1H, m, Ar*H*), 7.21-7.27 (3H, m, Ar*H*), 7.28-7.36 (4H, m, Ar*H*), 7.37-7.42 (2H, m, Ar*H*), 7.45-7.49 (1H, m, Ar*H*), 7.51-7.55 (2H, m, Ar*H*), 7.62-7.66 (1H, m, benzoxazoleC(4)*H*); δ_C (126 MHz, $CDCl_3$) 36.6 (*C*(3)H_2), 40.2 (*C*(4)H), 107.5 (*C*(5)), 110.4 (benzoxazole*C*(7')H), 119.9 (benzoxazole*C*(4')H), 124.5 (benzoxazole*C*(6')H), 125.3 (benzoxazole*C*(5')H), 126.9 (2 × Ar*C*H), 127.8 (C(4)Ph*C*(4)H), 128.2 (2 × Ar*C*H), 129.0 (2 × Ar*C*H), 129.3 (2 × Ar*C*H), 130.4 (C(6)Ph*C*(4)H), 132.6 (C(6)Ph*C*(1)), 139.4 (benzoxazole*C*(4a')), 141.5 (C(4)Ph*C*(1)), 150.2 (benzoxazole*C*(7a')), 156.0 (*C*(6)), 160.9 (benzoxazole*C*(2')), 166.2 (*C*(2)O); *m/z* (NSI^+) 390 ($[M+Na]^+$, 100%), 368 ($[M+H]^+$, 40%); HRMS (NSI^+) $C_{24}H_{17}O_3NNa$ ($[M+Na]^+$) requires 390.1101, found 390.1094 (–1.7 ppm).

(*R*)-3-(4-Methoxyphenyl)-*N,N*-dimethyl-1-oxo-2,3-dihydro-1*H*-benzo[4,5]thiazolo[3,2-*a*]pyridine-4-carboxamide (6A)

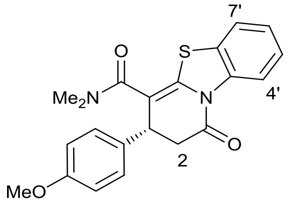

The title compound was prepared according to *General Procedure D* from (*E*)-4-methoxycinnamic anhydride (338 mg, 1.00 mmol), and 2-(1,3-benzothiazol-2-yl)-*N,N*-dimethylacetamide (158 mg, 0.72 mmol), EtN(*i*Pr)$_2$ (140 μL, 0.80 mmol) and HyperBTM (11.1 mg, 0.036 mmol) in THF (2 mL) and purified by chromatography on silica gel (10% EtOAc/CH_2Cl_2) to give **6A** as a yellow solid (145 mg, 53%); mp 157-159 °C; $[\alpha]_D^{20}$ –120.3 (*c* 1 in CH_2Cl_2); chiral HPLC analysis, ChiralPak AD-H (20% *i*-PrOH:hexane, flow rate 1.0 mL min^{-1}, 211 nm, 30 °C), t_R major: 19.9 min, t_R minor: 27.8 min, 93% ee; ν_{max} (film)/cm^{-1} 2929 (C-H), 1711 (C=O), 1688 (C=O), 1609 (C=C); δ_H (500 MHz, $CDCl_3$) 2.87 (6H, s, N(C*H*$_3$)$_2$), 2.95 (1H, dd, *J* 16.1, 5.9, C(2)*H*$_2$), 3.15 (1H, dd, *J* 16.1, 7.0, C(2)*H*$_2$), 3.77 (3H, s, OC*H*$_3$), 4.15 (1H, t, *J* 6.4, C(3)*H*), 6.80-6.86 (2H, m, C(3)ArC(3)*H*), 7.12-7.19 (3H, m, Ar*H*), 7.22 (1H, ddd, *J* 8.3, 7.5, 1.4, Ar*H*), 7.26-7.31 (1H, m, Ar*H*), 8.35 (1H, dd, *J* 8.2, 1.2, C(4')*H*); δ_C (126 MHz, $CDCl_3$) 36.9 (2 × N(*C*H$_3$)$_2$), 39.3 (*C*(3)H), 41.0 (*C*(2)H_2), 55.3 (O*C*H$_3$), 106.6 (*C*(4)), 114.4 (2 × C(3)Ar*C*(3)H), 117.5 (*C*(4')H), 121.2 (*C*(7')H), 125.3 (*C*(6')H), 126.0 (*C*(7a')), 126.2 (*C*(5')H), 127.9 (2 × C(3)Ar*C*(2)H), 132.7 (C(3)Ar*C*(4)), 137.2 (*C*(4a')), 142.1 (C(3)Ar*C*(1)), 158.82 (*C*(5)), 167.8 (*C*O), 169.3 (*C*O); *m/z* (NSI^+) 381 ($[M+H]^+$, 100%), 761 ($[2M+H]^+$, 35%); HRMS (NSI^+) $C_{21}H_{20}N_2O_3S$ ($[M+H]^+$) requires 381.1267, found 381.1267 (–0.1 ppm).

(11*R*)-10-Benzoyl-11-(4-methoxyphenyl)-8-thia-1-azatricyclo[7.4.0.0^{2,7}]trideca-2,4,6,9-tetraen-13-one (7A) and (4*R*)-5-(1,3-benzothiazol-2-yl)-4-(4-methoxyphenyl)-6-phenyl-3,4-dihydro-2H-pyran-2-one (7B)

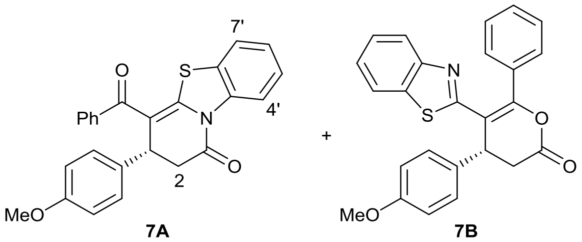

The title compounds were prepared according to *General Procedure D* from (*E*)-3-(4-methoxyphenyl)acrylic 3-(4-methoxyphenyl)propanoic anhydride (314 mg, 1.00 mmol) and 2-phenacyl benzthiazole (182 mg, 0.72 mmol), EtN(*i*Pr)$_2$ (138 μL, 0.79 mmol) and HyperBTM (2 mg, 1 mol %) in THF (2 mL) and purified by chromatography on silica gel (10:2 CH_2Cl_2/hexane) to afford **7A** as a yellow solid and **7B** as a yellow solid

7A (major): (436 mg, 59%); mp 190-191 °C; $[\alpha]_D^{20}$ –135.6 (*c* 1.0 in $CHCl_3$); chiral HPLC analysis, ChiralPak AD-H (20% *i*-PrOH:hexane, flow rate 1 mL min^{-1}, 211 nm, 30 °C), t_R minor: 18.4 min, t_R major: 31.1 min, 85% ee; ν_{max} (film)/cm^{-1} 2972 (C-H), 2843 (C-H), 1721 (C=O), 1603 (C=C), 1510 (C=C), 1474 (C-N), 1358 (C-S), 1298 (C-O); δ_H (300 MHz, $CDCl_3$) 3.00 (1H, dd, *J* 15.8, 2.3, C(12)*H*$_2$), 3.24 (1H, dd, *J* 15.8, 6.6 C(12)*H*$_2$), 3.77 (3H, s, OC*H*$_3$), 4.29 (1H, dd, *J* 6.6, 2.2, C(11)*H*), 6.78-6.84 (2H, m, C(11)ArC(3)*H*), 6.97-7.05 (2H, m, C(11)ArC(2)*H*), 7.23-7.31 (4H, m, Ar*H*), 7.32-7.43 (3H, m, Ar*H*), 7.56-7.65 (1H, m, C(6)*H*), 8.43-8.49 (1H, m, C(3)*H*); δ_C (75 MHz, $CDCl_3$) 38.0 (*C*(11)H), 41.8 (*C*(12)H$_2$), 55.4 (O*C*H$_3$), 108.2 (*C*(10)), 114.8 (2×C(11)Ar*C*(3)H), 117.7 (*C*(3)H), 122.1 (*C*(6)H), 126.0 (*C*(5)H), 127.2 (2×Ar*C*H and *C*(4)H), 127.9 (*C*(7)), 128.0 (2×Ar*C*H), 128.2 (2×Ar*C*H), 130.4 (C(10)COPh*C*(4)H), 132.7 (C(11)Ar*C*(1)), 136.2 (*C*(2)), 139.5 (C(10)COPh*C*(1)), 156.1 (*C*(9)), 159.0 (C(11)Ar*C*(4)), 168.2 (*C*(13)O), 191.4 (C(10)*C*O); *m/z* (NSI$^+$) 436 ([M+Na]$^+$, 100%), 414 ([M+H]$^+$, 60%); HRMS (NSI$^+$) $C_{25}H_{20}O_3NS$ ([M+H]$^+$) requires 414.1158, found 414.1160 (+0.4 ppm).

7B (minor): (41 mg, 6%); mp 148-151 °C; $[\alpha]_D^{20}$ +19.2 (*c* 1.0 in $CHCl_3$); chiral HPLC analysis, ChiralPak AD-H (20% *i*-PrOH:hexane, flow rate 1 mL min^{-1}, 211 nm, 30 °C), t_R minor: 9.7 min, t_R major: 14.6 min, 89% ee; ν_{max} (film)/cm^{-1} 2944 (C-H), 1778 (C=O), 1645 (C=N), 1510 (C=C), 1435 (C=C), 1346 (C-S), 1240 (C-O); δ_H (500 MHz, $CDCl_3$) 3.06 (1H, dd, *J* 15.7, 1.4, C(3)*H*$_2$), 3.30 (1H, dd, *J* 15.7, 7.5, C(3)*H*$_2$), 3.75 (3H, s, OC*H*$_3$), 4.97 (1H, d, *J*

6.5, C(4)*H*), 6.84 (2H, d, *J* 8.7, C(4)ArC(3)*H*), 7.28 (3H, dd, *J* 16.1, 8.3, C(4)ArC(2)*H*, C(5)HetArC(5)*H*), 7.38-7.46 (3H, m, C(5)HetArC(6)*H*, C(6)PhC(3)*H*), 7.50 (1H, m, C(6)PhC(4)*H*), 7.55-7.59 (2H, m, C(6)PhC(2)*H*), 7.62 (1H, d, *J* 8.0, C(5)HetArC(4)*H*), 7.95 (1H, d, *J* 8.2, C(5)HetArC(7)*H*); δ_C (126 MHz, $CDCl_3$) 37.2 (*C*(3)H_2), 40.6 (*C*(4)H), 55.3 (O*C*H_3), 114.6 (2×C(4)Ar*C*(3)H), 115.4 (*C*(5)), 121.3 (C(5)HetAr*C*(7)), 123.1 (C(5)HetAr*C*(4)), 125.5 (C(5)HetAr*C*(6)), 126.2 (C(5)HetAr*C*(5)), 128.2 (2×Ar*C*H), 128.9 (2×Ar*C*H), 130.1 (2×Ar*C*H), 130.8 (C(6)Ph*C*(4)H), 131.6 (C(4)Ar*C*(1)), 131.9 (C(6)Ph*C*(1)), 138.2 (C(5)HetAr*C*), 152.2 (C(5)HetAr*C*), 154.2 (*C*(6)), 159.1 (C(4)Ar*C*(4)), 164.4 (C(5)HetAr*C*=N)), 166.8 (*C*(2)O); *m/z* (NSI^+) 436 ($[M+Na]^+$, 100%), 414 ($[M+H]^+$, 90%); HRMS (NSI^+) $C_{25}H_{20}O_3NS$ ($[M+H]^+$) requires 414.1158, found 414.1156 (–0.6 ppm).

(*R*)-5-(Benzo[*d*]oxazol-2-yl)-4-(4-methoxyphenyl)-6-phenyl-3,4-dihydro-2*H*-pyran-2-one (8B)

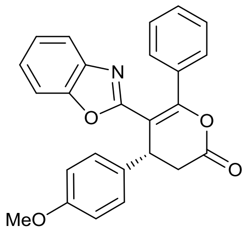

The title compound was prepared according to *General Procedure D* from (*E*)-*p*-methoxycinnamic anhydride (338 mg, 1.00mmol), and 2-phenacylbenzoxazole (171 mg, 0.72 mmol), EtN(*i*Pr)$_2$ (140 µL, 0.80 mmol) and HyperBTM (11.1 mg, 0.036 mmol) in THF (2 mL) and purified by chromatography on silica gel (CH_2Cl_2→10% EtOAC/CH_2Cl_2) to give **8B** as a yellow foamy solid (211 mg, 74%); mp 98-99 °C; $[\alpha]_D^{20}$ +47.0 (*c* 0.5 in CH_2Cl_2); chiral HPLC analysis, ChiralPak AD-H (5% *i*-PrOH:hexane, flow rate 1.0 mL min^{-1}, 211 nm, 30 °C), t_R major: 31.6 min, t_R minor: 26.7 min, 90% ee; ν_{max} (film)/cm^{-1} 2974 (C-H), 2901 (C-H), 1772 (C=O), 1636 (C=C), 1601 (C=C); δ_H (500 MHz, $CDCl_3$) 3.07 (1H, dd, *J* 15.7, 1.8, C(3)*H*H), 3.27 (1H, dd, *J* 15.6, 7.5, C(3)*H*H), 3.76 (3H, s, O*C*H_3), 4.84 (1H, dd, *J* 7.5, 1.7, C(4)*H*), 6.82-6.89 (2H, m, 2 × C(4)ArC(2)*H*), 7.19-7.31 (5H, m, Ar*H*), 7.41 (2H, t, *J* 7.6, 2 × C(6)ArC(3)*H*), 7.48 (1H, t, *J* 7.5, C(5)benzoxazoleC(7)*H*), 7.54 (2H, dd, *J* 7.2, 1.7, 2 × C(6)ArC(2)*H*), 7.66 (1H, dd, *J* 7.6, 1.5, C(5)benzoxazoleC(4)*H*); δ_C (126 MHz, $CDCl_3$) 37.0 (*C*(3)H_2), 39.7 (*C*(4)H), 55.4 (O*C*H_3), 108.0 (*C*(5)), 110.6 (C(5)benzoxazole*C*(7)H), 114.7 (2 × C(4)Ar*C*(2)H), 120.0 (C(5)benzoxazole*C*(4)H), 124.6 (C(5)benzoxazole*C*(6)H), 125.4 (C(5)benzoxazole*C*(5)H), 128.1 (2 × Ar*C*H), 128.3 (2 × Ar*C*H), 129.1 (2 × Ar*C*H), 130.5 (C(6)Ar*C*(4)H), 131.4 (C(6)Ar*C*(1)), 132.7 (C(4)Ar*C*(4)), 141.6 (C(5)benzoxazole*C*(4a)),

150.3 (C(5)benzoxazole*C*(7a)), 155.9 (*C*(6)), 159.2 (C(4)Ar*C*(1)OCH$_3$), 161.1 (C(5)benzoxazole*C*(2)), 166.5 (*C*(2)O); *m/z* (NSI$^+$) 398 ([M+H]$^+$, 25%), 420 ([M+Na]$^+$, 100%), 436 ([M+K]$^+$, 10%); HRMS (NSI$^+$) $C_{25}H_{20}NO_4$ ([M+H]$^+$) requires 398.1387, found 398.1381 (–1.5 ppm).

(*R*)-*N*,*N*-Dimethyl-1-oxo-3-(4-(trifluoromethyl)phenyl)-2,3-dihydro-1H-benzo[4,5]thiazolo[3,2-a]pyridine-4-carboxamide (9A)

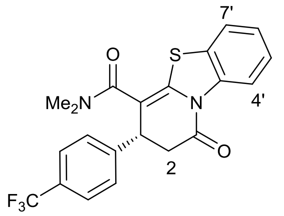

The title compound was prepared according to *General Procedure D* from (*E*)-*p*-trifluoromethylcinnamic anhydride (414 mg, 1.00 mmol), and 2-(1,3-benzothiazol-2-yl)-*N*,*N*-dimethylacetamide (158 mg, 0.72 mmol), EtN(*i*Pr)$_2$ (140 μL, 0.80 mmol) and HyperBTM (11.1 mg, 0.036 mmol) in THF (2 mL) and purified by chromatography on silica gel (10% EtOAc/CH_2Cl_2) to give **9A** as a yellow foamy solid (179 mg, 60%); mp 192-193 °C; $[\alpha]_D^{20}$ –104.1 (*c* 1 in CH_2Cl_2); chiral HPLC analysis, ChiralPak AD-H (20% *i*-PrOH:hexane, flow rate 1.0 mL min^{-1}, 211 nm, 30 °C), t_R major: 14.3 min, t_R minor: 21.2 min, 95% ee; ν_{max} (film)/cm^{-1} 2974 (C-H), 2938 (C-H), 1705 (C=O), 1692 (C=O), 1626 (C=C); δ_H (500 MHz, $CDCl_3$) 2.89 (6H, s, N(C*H*$_3$)$_2$), 2.96 (1H, dd, *J* 16.2, 6.2, C(2)*H*$_2$), 3.19 (1H, dd, *J* 16.3, 7.1, C(2)*H*$_2$), 4.27 (1H, t, *J* 6.7, C(3)*H*), 7.12-7.19 (1H, m, Ar*H*), 7.18-7.25 (1H, m, Ar*H*), 7.26-7.31 (1H, m, Ar*H*), 7.37 (2H, d, *J* 8.1, 2 × C(3)ArC(2)*H*), 7.53-7.60 (2H, m, 2 × C(3)ArC(3)*H*), 8.31-8.36 (1H, m, C(4')*H*); δ_C (126 MHz, $CDCl_3$) 36.9 (2 × N(*C*H$_3$)$_2$), 40.0 (*C*(3)H), 40.5 (*C*(2)H$_2$), 105.0 (*C*(4)), 117.5 (*C*(4')H), 121.3 (*C*(7')H), 124.0 (q, *J* 272.1, *C*F$_3$), 125.5 (*C*(6')H), 125.6 (*C*(7a')), 126.1 (q, *J* 3.8, 2 × C(3)Ar*C*(3)H), 126.4 (*C*(5')H), 127.3 (2 × C(3)Ar*C*(2)H), 129.7 (q, *J* 32.5 C(3)Ar*C*(4)CF$_3$), 137.0 (*C*(4a')), 142.9 (C(3)Ar*C*(1)), 145.1 (*C*(5)), 167.0 (*C*(1)O), 169.0 (*C*ONMe$_2$); δ_F (470 MHz, $CDCl_3$) –62.53; *m/z* (APCI$^+$) 419 ([M+H]$^+$, 100%); HRMS (NSI$^+$) $C_{21}H_{18}F_3N_2O_2S$ ([M+H]$^+$) requires 419.1036, found 419.1040 (+1.1 ppm).

(*R*)-4-Benzoyl-3-(4-(trifluoromethyl)phenyl)-2,3-dihydro-1H-benzo[4,5]thiazolo[3,2-a]pyridin-1-one (10A) and *(R)*-5-(benzo[d]thiazol-2-yl)-6-phenyl-4-(4-(trifluoromethyl)phenyl)-3,4-dihydro-2H-pyran-2-one (10B)

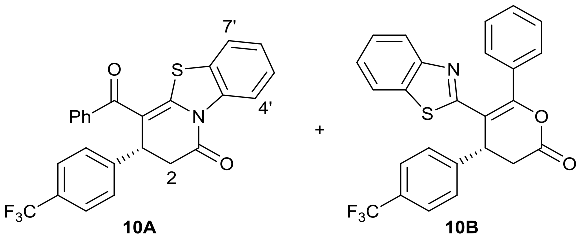

The title compounds were prepared according to *General Procedure D* from (*E*)-*p*-trifluoromethylcinnamic anhydride (275 mg, 0.66 mmol) and 2-phenacylbenzothiazole (120 mg, 0.47 mmol), EtN(*i*Pr)$_2$ (90 μL, 0.52 mmol) and HyperBTM (7.3 mg, 0.024 mmol) in THF (1.5 mL) and purified by chromatography on silica gel (CH_2Cl_2) to give **10A/10B** as a yellow solid (83:17 mixture of constitutional isomers, 252 mg, 78%). Analytical samples were separated by careful chromatography.

10A (major): mp 168-170 °C; $[\alpha]_D^{20}$ −100.0 (*c* 1.0 in CH_2Cl_2); chiral HPLC analysis, ChiralPak AD-H (5% *i*-PrOH:hexane, flow rate 1 mL min^{-1}, 211 nm, 30 °C), t_R major: 56.3 min, t_R minor: 31.1 min, 79% ee; ν_{max} (film)/cm^{-1} 2359 (C-H), 1719 (C=O), 1618 (C=O), 1489 (C=C); δ_H (500 MHz, $CDCl_3$) 3.02 (1H, dd, *J* 16.0, 2.4, C(2)*H*H), 3.33 (1H, dd, *J* 16.0, 7.0, C(2)*H*H), 4.42 (1H, dd, *J* 7.1, 2.4, C(3)*H*), 7.17-7.25 (4H, m, 2 × C(3)ArC(3)*H*, C(5')*H*, C(6')*H*), 7.26-7.33 (2H, m, 2 × C(3)ArC(2)*H*), 7.33-7.44 (3H, m, 2 × C(4)COArC(3)*H*, C(4)COArC(4)*H*), 7.52-7.58 (2H, m, 2 × C(4)COArC(2)*H*), 7.59-7.65 (1H, m, C(7')*H*), 8.46 (1H, dd, *J* 7.9, 1.5, C(4')*H*); δ_C (126 MHz, $CDCl_3$) 38.6 (*C*(3)H), 41.2 (*C*(2)H$_2$), 106.9 (*C*(4)), 117.7 (*C*(4')H), 122.2 (*C*(7')H), 124.0 (q, $^1J_{CF}$ 272.2, *C*F$_3$), 126.3, (*C*(6')H), 126.5 (q, $^3J_{CF}$ 3.7, 2 × C(3)Ar*C*(3)H), 126.9 (2 × C(4)COPh*C*(2)H), 127.3 (*C*(5')H), 127.5 (2 × C(4)COPh*C*(3)H), 127.7 (*C*(7a')), 128.5 (2 × C(3)Ar*C*(2)H), 130.0 (q, $^2J_{CF}$ 32.7, C(3)Ar*C*(4)-CF$_3$), 130.6 (C(4)COPh*C*(4)H), 136.0 (*C*(4a')), 139.5 (C(4)COPh*C*(1)), 145.3 (C(3)Ar*C*(1)), 156.8 (*C*(5)), 167.4 (*C*(1)O), 191.2 (C(4)*C*OPh); δ_F (470 MHz, $CDCl_3$) –62.6; *m/z* (APCI$^+$) 452 ([M+H]$^+$, 100%); HRMS (APCI$^+$) $C_{25}H_{17}NO_2S$ ([M+H]$^+$) requires 452.0927, found 452.0923 (–0.8 ppm).

10B (minor): mp 208-211 °C; $[\alpha]_D^{20}$ +14.4 (*c* 0.125 in CH_2Cl_2); chiral HPLC analysis, ChiralPak AD-H (5% *i*-PrOH:hexane, flow rate 1 mL min^{-1}, 254 nm, 30 °C), t_R major: 15.2 min, t_R minor: 11.5 min, 90% ee; ν_{max} (film)/cm^{-1} 2955 (C-H), 2926 (C-H), 1782 (C=O); δ_H (500 MHz, $CDCl_3$) 3.08 (1H, dd, *J* 15.8, 1.7, C(3)*H*H), 3.35 (1H, dd, *J* 15.9, 7.7, C(3)*H*H),

5.14 (1H, dd, *J* 7.7, 1.7, C(4)*H*), 7.27-7.33 (1H, m, benzothiazoleC(6)*H*), 7.36-7.43 (1H, m, benzothiazoleC(5)*H*), 7.43-7.50 (4H, m, Ar*H*), 7.49-7.65 (6H, m, Ar*H*), 7.91 (1H, d, *J* 8.3, benzothiazoleC(7)*H*); δ_C (126 MHz, $CDCl_3$) 36.6 (*C*(3)H_2), 40.8 (*C*(4)H), 114.8 (*C*(5)), 121.4 (benzothiazole*C*(4)H), 123.2 (benzothiazole*C*(7)H), 125.7 (benzothiazole*C*(6)H), 126.3 (benzothiazole*C*(5)H), 126.3 (q, $^3J_{CF}$ 4.0, 2 × C(4)Ar*C*(3)H), 127.6 (2 × C(6)Ph*C*(2)H), 129.2 (2 × C(4)Ar*C*(2)H), 130.2 (2 × C(6)Ph*C*(3)H), 131.2 (C(6)Ph*C*(4)H), 131.7 (C(6)Ph*C*(1)), 135.7 (benzothiazole*C*(7a)), 143.9 (C(4)Ar*C*(1)), 152.4 (benzothiazole*C*(4a)), 155.0 (*C*(6)), 163.6 (benzothiazole*C*(2)), 166.2 (*C*(2)O) [C(4)Ar*C*(4)-CF_3 and *C*F_3 not seen in ^{13}C NMR due to low sample quantity, visibility could not be improved]; δ_F (470 MHz, $CDCl_3$) –62.7; *m/z* (APCI$^+$) 452 ([M+H]$^+$, 100%); HRMS (APCI$^+$) $C_{25}H_{17}NO_2S$ ([M+H]$^+$) requires 452.0927, found 452.0920 (–1.5 ppm).

(*R*)-5-(Benzo[*d*]oxazol-2-yl)-6-phenyl-4-(4-(trifluoromethyl)phenyl)-3,4-dihydro-2*H*-pyran-2-one (11B)

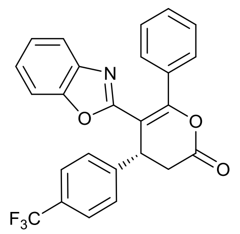

The title compound was prepared according to *General Procedure D* from (*E*)-*p*-trifluoromethylcinnamic anhydride (414 mg, 1.00 mmol), and 2-phenacylbenzoxazole (171 mg, 0.72 mmol), EtN(*i*Pr)$_2$ (140 μL, 0.80 mmol) and HyperBTM (11.1 mg, 0.036 mmol) in THF (2 mL) and purified by chromatography on silica gel (10:2 CH_2Cl_2/hexane) to give **11B** as a light green solid (112 mg, 36%); mp 193-196 °C; $[\alpha]_D^{20}$ +30.4 (*c* 0.5 in CH_2Cl_2); chiral HPLC analysis, ChiralPak AD-H (5% *i*-PrOH:hexane, flow rate 1.0 mL min^{-1}, 211 nm, 30 °C), t_R major: 14.3 min, t_R minor: 15.7 min, 99% ee; ν_{max} (film)/cm^{-1} 3061 (C-H), 2974 (C-H), 1770 (C=O), 1655 (C=C), 168 (C=C); δ_H (500 MHz, $CDCl_3$) 3.10 (1H, dd, *J* 15.8, 1.8, C(3)*H*H), 3.34 (1H, dd, *J* 15.8, 7.6, C(3)*H*H), 4.98 (1H, dd, *J* 7.8, 2.0, C(4)*H*), 7.20-7.24 (1H, m, benzoxazoleC(7)*H*), 7.24-7.33 (2H, m, Ar*H*), 7.44 (2H, t, *J* 7.7 Ar*H*), 7.52 (3H, td, *J* 7.8, 1.8, Ar*H*), 7.54-7.59 (2H, m, Ar*H*), 7.61 (2H, d, *J* 8.2, 2 × C(6)ArC(2)*H*), 7.66 (1H, dd, *J* 7.3, 1.6, benzoxazoleC(4)*H*); δ_C (126 MHz, $CDCl_3$) 36.3 (*C*(3)H_2), 40.1 (*C*(4)H), 106.9 (*C*(5)), 110.6 (benzoxazole*C*(7)H), 120.0 (benzoxazole*C*(4)H), 124.0 (q, *J* 272.2, *C*F_3), 124.7 (benzoxazole*C*(6)H), 125.6 (benzoxazole*C*(5)H), 126.4 (q, *J* 3.8, 2 × C(4)Ar*C*(3)H), 127.5 (2 × C(4)Ar*C*(2)H), 128.4 (2 × C(6)Ar*C*(3)H), 129.1 (2 × C(6)Ar*C*(2)H), 130.28 (q, *J* 32.6,

C(4)Ar*C*(4)), 130.8 (C(6)Ar*C*(4)H), 132.4 (C(6)Ar*C*(1)), 141.5 (benzoxazole*C*(4a)), 143.7 (C(4)Ar*C*(1)), 150.3 (benzoxazole*C*(7a)), 156.6 (*C*(6)), 160.6 (benzoxazole*C*(2)), 165.8 (*C*(2)O); δ_F (470 MHz, $CDCl_3$) –62.68; *m/z* ($APCI^+$) 435 ($[M+H]^+$, 85%), plus decomposition peaks: 198 (100%), 252 (100%), 331 (90%), 359 (90%); HRMS ($APCI^+$) $C_{25}H_{17}F_3NO_3$ ($[M+H]^+$) requires 436.1155, found 436.1154 (–0.2 ppm).

(*R*)-3-(3-Bromophenyl)-*N*,*N*-dimethyl-1-oxo-2,3-dihydro-1*H*-benzo[4,5]thiazolo[3,2-*a*]pyridine-4-carboxamide (12A)

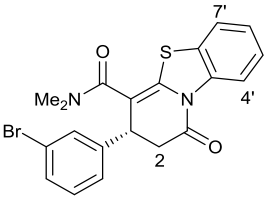

The title compound was prepared according to *General Procedure D* from *(E)*-3-bromocinnamic anhydride (436 mg, 1.00 mmol) and 2-(1,3-benzothiazol-2-yl)-*N*,*N*-dimethylacetamide (158 mg, 0.72 mmol), EtN(*i*Pr)$_2$ (140 µL, 0.80 mmol) and HyperBTM (11.1 mg, 0.036 mmol) in THF (2 mL) and purified by chromatography on silica gel (CH_2Cl_2 → 10% EtOAc/CH_2Cl_2) to give **12A** as a yellow solid (283 mg, 92%); mp 69-72 °C; $[\alpha]_D^{20}$ –103.3 (*c* 1.0, CH_2Cl_2); chiral HPLC analysis, ChiralPak AD-H (20% *i*-PrOH:hexane, flow rate 1 mL min^{-1}, 211 nm, 30 °C), t_R major: 15.4 min, t_R minor: 38.8 min, 96% ee; ν_{max} (film)/cm^{-1} 2922 (C-H), 1701 (C=O), 1613 (C=O), 1587 (C=N), 1452 (C=C); δ_H (500 MHz, $CDCl_3$) 2.89 (6H, s, N(C*H*$_3$)$_2$), 2.94 (1H, dd, *J* 16.3, 6.1, C(2)*H*H), 3.16 (1H, dd, *J* 16.3, 7.2, C(2)*H*H), 4.18 (1H, dd, *J* 7.2, 6.0, C(3)*H*), 7.14-7.19 (3H, m, Ar*H*), 7.20-7.25 (1H, m, Ar*H*), 7.28-7.30 (1H, m, Ar*H*), 7.35-7.40 (2H, m, Ar*H*), 8.35 (1H, dd, *J* 8.3, 0.8, C(4')*H*); δ_C (75 MHz, $CDCl_3$) 37.0 (2 × N(*C*H$_3$)$_2$), 40.0 (*C*(3)H), 40.6 (*C*(2)H$_2$), 105.4 (*C*(5)), 117.7 (*C*(4')H), 121.4 (*C*(7')H), 123.1 (C(3)Ar*C*(1)Br), 125.5, (Ar*C*H), 125.6 (Ar*C*H), 125.8 (*C*(7a')), 126.5 (*C*(5')H), 130.1 (Ar*C*H), 130.8 (Ar*C*H), 130.8 (Ar*C*H), 137.2 (*C*(4a'), 142.7 (C(3)Ar*C*(3)), 143.4 (*C*(5)), 167.3 (*C*(1)O), 169.1 (*C*ONMe$_2$); *m/z* ($APCI^+$) 429 ($[M+H]^+$, 100%); HRMS ($APCI^+$) $C_{20}H_{18}Br^{79}N_2O_2S$ ($[M+H]^+$) requires 429.0267, found 429.0267 (+0.0 ppm).

(*R*)-4-benzoyl-3-(3-bromophenyl)-2,3-dihydro-1*H*-benzo[4,5]thiazolo[3,2-*a*]pyridin-1-one (13A) and (*R*)-5-(benzo[*d*]thiazol-2-yl)-4-(3-bromophenyl)-6-phenyl-3,4-dihydro-2*H*-pyran-2-one (13B)

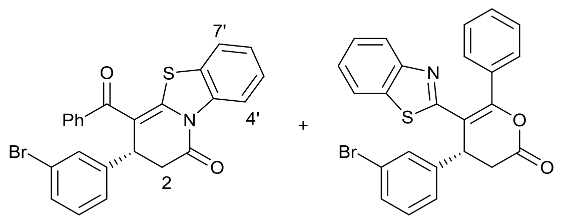

The title compounds were prepared according to *General Procedure D* from (*E*)-3-bromocinnamic anhydride (436 mg, 1.00 mmol) and 2-phenacyl benzothiazole (182 mg, 0.72 mmol), EtN(*i*Pr)$_2$ (140 µL, 0.80 mmol) and HyperBTM (11.1 mg, 0.036 mmol) in THF (2 mL) and purified by chromatography on silica gel (20% EtOAc/petrol) to give **13A/13B** as a yellow solid (86:14 mixture of constitutional isomers, 287 mg, 86%); mp 163-167 °C; $[\alpha]_D^{20}$ –197.8 (*c* 0.5, CH_2Cl_2); ν_{max} (film)/cm^{-1} 3050 (C-H), 1774 (C=O, lactone), 1717 (C=O, ketone), 1601 (C=O, lactam), 1571 (C=N), 1474 (C=C); *m/z* (APCI$^+$) 462 ([M+H]$^+$, 100%); HRMS (APCI$^+$) $C_{24}H_{17}Br^{79}NO_2S$ ([M+H]$^+$) requires 462.0158, found 462.0156 (–0.4 ppm).

13A (major): chiral HPLC analysis, ChiralPak AD-H (20% *i*-PrOH:hexane, flow rate 1 mL min^{-1}, 211 nm, 30 °C), t_R major: 21.4 min, t_R minor: 15.8 min, 84% ee; δ_H (500 MHz, $CDCl_3$) 3.04 (1H, dd, *J* 16.0, 2.4, C(2)*H*H), 3.25-3.33 (1H, m, C(2)*H*H), 4.34 (1H, dd, *J* 7.1, 2.4, C(3)*H*), 7.02-7.07 (1H, m, C(3)Ar*C*(6)H)), 7.16 (2H, t, *J* 7.8, Ar*H*), 7.24-7.45 (9H, m, Ar*H*), 7.62 (1H, dd, *J* 7.4, 1.7, C(7')*H*), 8.48 (1H, dd, *J* 8.1, 1.4, C(4')*H*); δ_C (126 MHz, $CDCl_3$) 38.3 (*C*(3)H), 41.1 (*C*(2)H_2), 106.8 (*C*(4)), 117.6 (*C*(4')H), 122.0 (*C*(7')H), 123.3 (C(3)Ar*C*(1)Br), 125.3 (*C*(6')H), 126.1 (C(3)Ar*C*(6)H), 126.8 (2 × C(4)COAr*C*(2)H), 127.2 (C(3)Ar*C*(2)H), 127.6 (*C*(7a')), 128.2 (2 × C(4)COAr*C*(2)H), 130.1 (Ar*C*H), 130.4 (Ar*C*H), 130.9 (Ar*C*H), 130.9 (Ar*C*H), 136.0 (*C*(4a')), 139.3 (C(4)COAr*C*(1)), 143.4 (C(3)Ar*C*(3)), 156.5 (*C*(5)), 167.5 (*C*(1)O), 191.2 (C(4)*C*OAr).

13B (minor): chiral HPLC analysis, ChiralPak AD-H (20% *i*-PrOH:hexane, flow rate 1 mL min^{-1}, 211 nm, 30 °C), t_R major: 11.7 min, t_R minor: 8.4 min, 83% ee; δ_H (500 MHz, $CDCl_3$, chatacteristic peaks) 3.08 (1H, dd, *J* 15.8, 1.7, C(3)*H*H), 3.34 (1H, dd, *J* 15.8, 7.6, C(3)*H*H), 5.06 (1H, dd, *J* 7.7, 1.7, C(4)*H*), 7.94 (1H, d, *J* 8.2, benzothiazoleC(7)*H*); δ_C (126 MHz, $CDCl_3$, characteristic peaks) 36.7 (*C*(3)H_2), 40.6 (*C*(4)H), 114.5 (*C*(5)), 121.2 (benzothiazole*C*(7)H), 123.0 (benzothiazole*C*(4)H), 123.2 (C(4)Ar*C*(1)Br), 125.4 (Ar*C*H), 125.5 (Ar*C*H), 126.1 (Ar*C*H), 129.0 (2 × C(6)Ar*C*(3)H), 130.0 (2 × C(6)Ar*C*(2)H), 130.8

(Ar*C*H), 131.0 (Ar*C*H), 131.6 (C(6)Ar*C*(1)), 135.6 (benzothiazole*C*(7a)), 142.0 (C(4)Ar*C*(3)), 152.2 (benzothiazole*C*(4a)), 154.9 (*C*(6)), 163.6 (benzothiazole*C*(2)), 166.1 (*C*(2)O).

(*R*)-5-(Benzo[*d*]oxazol-2-yl)-4-(3-bromophenyl)-6-phenyl-3,4-dihydro-2*H*-pyran-2-one (14B)

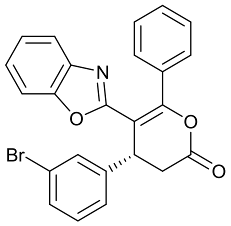

The title compund was prepared according to *General Procedure D* from (*E*)-3-bromocinnamic anhydride (436 mg, 1.00 mmol) and 2-phenacyl benzoxazole (171 mg, 0.72 mmol), $EtN(iPr)_2$ (140 µL, 0.80 mmol) and HyperBTM (11.1 mg, 0.036 mmol) in THF (2 mL) and purified by chromatography on silica gel (20% EtOAc/petrol) to give **14B** as a colourless solid (251 mg, 78%); mp 148-149 °C; $[\alpha]_D^{20}$ +17.7 (*C* 1.0, CH_2Cl_2); chiral HPLC analysis, ChiralPak OD-H (20% *i*-PrOH:hexane, flow rate 1 mL min^{-1}, 211 nm, 30 °C), t_R major: 12.9 min, t_R minor: 9.3 min, 99% ee; ν_{max} (film)/cm^{-1} 3013 (C-H), 1761 (C=O), 1651 (C=N), 1452 (C=C); δ_H (500 MHz, $CDCl_3$) 3.06 (1H, dd, *J* 15.8, 1.8, C(3)*H*H), 3.28 (1H, dd, *J* 15.8, 7.6, C(3)*H*H), 4.85 (1H, dd, *J* 7.6, 1.8, C(4)*H*), 7.16-7.21 (2H, m, Ar*H*), 7.22-7.30 (3H, m, Ar*H*), 7.36-7.43 (3H, m, Ar*H*), 7.46-7.51 (2H, m, Ar*H*), 7.51-7.56 (2H, m, Ar*H*), 7.61-7.67 (1H, m, benzoxazoleC(4)*H*); δ_C (101 MHz, $CDCl_3$) 36.5 (*C*(3)H_2), 40.0 (*C*(4)H), 106.9 (*C*(5)), 110.6 (benzoxazole*C*(7), 120.0 (benzoxazole*C*(4)H), 123.4 (C(4)Ar*C*(1)Br), 124.7 (C(4)Ar*C*(4)H), 125.4 (benzoxazole*C*(5,6)), 125.5 (benzoxazole*C*(5,6)), 128.3 (2 × C(6)Ph*C*(3)H), 129.1 (2 × C(6)Ph*C*(2)H), 130.3 (Ar*C*H), 130.7 (Ar*C*H), 130.9 (Ar*C*H), 131.2 (Ar*C*H), 132.5 (C(6)Ph*C*(1)), 141.5 (Ar*C*), 141.8 (Ar*C*), 150.3 (benzoxazole*C*(7a)), 156.5 (*C*(6)), 160.6 (benzoxazole*C*(2)), 165.8 (*C*(2)O); *m/z* ($APCI^+$) 446 ($[M+H]^+$, 100%); HRMS ($APCI^+$) $C_{24}H_{17}Br^{79}NO_3$ ($[M+H]^+$) requires 446.0386, found 446.0386 (–0.1 ppm).

(*S*)-3-(2-Bromophenyl)-*N*,*N*-dimethyl-1-oxo-2,3-dihydro-1*H*-benzo[4,5]thiazolo[3,2-*a*]pyridine-4-carboxamide (15A)

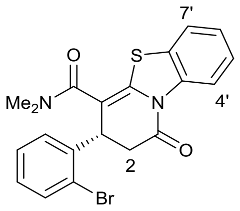

The title compound was prepared according to *General Procedure D* from (*E*)-2-bromocinnamic anhydride (436 mg, 1.00 mmol) and 2-(1,3-benzothiazol-2-yl)-*N*,*N*-dimethylacetamide (158 mg, 0.72 mmol), EtN(*i*Pr)$_2$ (140 μL, 0.80 mmol) and HyperBTM (11.1 mg, 0.036 mmol) in THF (2 mL) and purified by chromatography on silica gel (10% EtOAc/CH_2Cl_2) to give **15A** as a yellow solid (243 mg, 79%); mp 149-152 °C; $[\alpha]_D^{20}$ –20.0 (*c* 1.0, CH_2Cl_2); chiral HPLC analysis, ChiralPak AD-H (20% *i*-PrOH:hexane, flow rate 1 mL min^{-1}, 211 nm, 30 °C), t_R major: 22.8 min, t_R minor: 16.2 min, 96% ee; ν_{max} (film)/cm^{-1} 2924 (C-H), 1710 (C=O), 1604 (C=O), 1585 (C=N), 1454 (C=C); δ_H (500 MHz, $CDCl_3$) 2.87 (6H, s, N(C*H*$_3$)$_3$), 2.95 (1H, dd, *J* 16.2, 5.6, C(2)*H*H), 3.14 (1H, dd, *J* 16.2, 6.9, C(2)*H*H), 4.64 (1H, dd, *J* 6.9, 5.6, C(3)*H*), 7.08-7.15 (1H, m, Ar*H*), 7.16-7.29 (4H, m, Ar*H*), 7.35 (1H, dd, *J* 7.5, 1.5, C(3)ArC(2)*H*), 7.59 (1H, dd, *J* 8.0, 1.3, C(7')*H*), 8.31-8.36 (1H, m, C(4')*H*); δ_C (126 MHz, $CDCl_3$) 37.2 (2 × N(*C*H$_3$)$_2$), 39.2 (*C*(3)H), 39.5 (*C*(2)H$_2$), 104.8 (*C*(4)), 117.5 (*C*(4')H), 121.3 (*C*(7')H), 123.7 (C(3)Ar*C*(1)Br), 125.5 (*C*(6')H), 126.2 (Ar*C*H), 126.8 (*C*(7a')), 128.1 (Ar*C*H), 128.3 (Ar*C*H), 129.2 (Ar*C*H), 133.7 (C(3)Ar*C*(6)), 136.8 (*C*(4a')), 139.2 (C(3)Ar*C*(2)), 147.3 (*C*(5)), 167.3 (*C*(1)O), 169.0 (*C*ONMe$_2$); *m/z* (APCI$^+$) 431 ([M+H]$^+$, 100%); HRMS (APCI$^+$) $C_{20}H_{18}Br^{81}O_2N_2S$ ([M+H]$^+$) requires 429.0267, found 429.0267 (+0.0 ppm).

(*S*)-4-Benzoyl-3-(2-bromophenyl)-2,3-dihydro-1*H*-benzo[4,5]thiazolo[3,2-*a*]pyridin-1-one (16A) and (*S*)-5-(benzo[*d*]thiazol-2-yl)-4-(2-bromophenyl)-6-phenyl-3,4-dihydro-2*H*-pyran-2-one (16B)

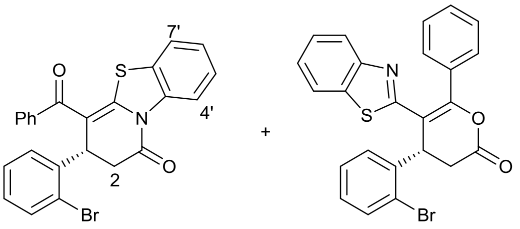

The title compounds were prepared according to *General Procedure D* from *(E)*-2-bromocinnamic anhydride (436 mg, 1.00 mmol) and 2-phenacyl benzothiazole (182 mg, 0.72 mmol), EtN(*i*Pr)$_2$ (140 µL, 0.80 mmol) and HyperBTM (11.1 mg, 0.036 mmol) in THF (2 mL) and purified by chromatography on silica gel (20% EtOAc/petrol) to give **16A/16B** as a yellow solid (92:8 mixture of constitutional isomers, 204 mg, 62%); mp 86-92 °C; $[\alpha]_D^{20}$ –137.6 (*c* 0.5, CH_2Cl_2); ν_{max} (film)/cm^{-1} 3061 (C-H), 3011 (C-H), 2905 (C-H), 1780 (C=O, lactone), 1720 (C=O, ketone), 1606 (C=O, lactam), 1572 (C=N), 1481 (C=C); *m/z* (APCI$^+$) 462 ([M+H]$^+$, 100%); HRMS (APCI$^+$) $C_{24}H_{17}Br^{79}NO_2S$ ([M+H]$^+$) requires 462.0158, found 462.0156 (–0.4 ppm).

16A (major): chiral HPLC analysis, ChiralPak AD-H (20% *i*-PrOH:hexane, flow rate 1 mL min^{-1}, 211 nm, 30 °C), t_R major: 18.7 min, t_R minor: 13.0 min, 81% ee; δ_H (500 MHz, $CDCl_3$) 3.12 (1H, dd, *J* 16.1, 2.3, C(2)*H*H), 3.23 (1H, dd, *J* 16.1, 7.3, C(2)*H*H), 4.73 (1H, dd, *J* 7.3, 2.3, C(3)*H*), 7.13-7.26 (5H, m, Ar*H*), 7.27-7.33 (2H, m, Ar*H*), 7.34-7.43 (3H, m, Ar*H*), 7.61-7.66 (2H, m, 2 × C(4)COArC(2)*H*), 8.40-8.56 (1H, m, C(4')*H*); δ_C (126 MHz, $CDCl_3$) 38.4 (*C*(3)H), 39.1 (*C*(2)H$_2$), 107.2 (*C*(4)), 117.5 (*C*(4')H), 122.1 (*C*(7')), 123.7 (C(3)Ar*C*(1)Br), 126.0 (*C*(6')H), 126.6 (2 × C(4)COAr*C*(2)H), 127.2 (*C*(5')H), 127.8 (*C*(7a')), 128.1 (C(3)Ar*C*H), 128.2 (2 × C(4)COAr*C*(3)H), 128.5 (C(3)Ar*C*H), 129.4 (C(3)Ar*C*H), 130.3 (C(4)COAr*C*(4)H), 134.0 (C(3)Ar*C*(6)H), 136.0 (*C*(4a')), 139.0 (C(4)COAr*C*(1)), 139.0 (C(3)Ar*C*(2)), 157.0 (*C*(5)), 167.6 (*C*(1)O), 191.0 (C(4)*C*OPh).

16B (minor): chiral HPLC analysis, ChiralPak AD-H (20% *i*-PrOH:hexane, flow rate 1 mL min^{-1}, 211 nm, 30 °C), t_R major: 8.2 min, t_R minor: 7.6 min, 33% ee; δ_H (500 MHz, $CDCl_3$, characteristic peaks) 3.18 (1H, dd, *J* 16.1, 1.8, C(3)*H*H), 3.30 (1H, dd, *J* 15.9, 7.8, C(3)*H*H), 5.47 (1H, dd, *J* 7.8, 1.7, C(4)*H*), 7.93 (1H, d, *J* 8.4, benzothiazoleC(7)*H*); δ_C (126 MHz, $CDCl_3$) 35.3 (*C*(3)H$_2$), 41.1 (*C*(4)H), 114.0 (*C*(5)), 121.2 (Ar*C*H), 123.3 (Ar*C*H), 125.5 (Ar*C*H), 126.1 (Ar*C*H), 127.4 (Ar*C*H), 128.9 (2 × C(6)Ar*C*(3)H), 129.5 (Ar*C*H), 129.9 (2 ×

C(6)Ar*C*(2)H), 130.9 (C(6)Ar*C*(4)H), 131.7 (C(6)Ar*C*(1)), 133.9 (Ar*C*H), 135.6 (benzothiazole*C*(7a)), 137.7 (C4)Ar*C*(2)), 152.3 (benzothiazole*C*(4a)), 155.3 (*C*(6)), 163.6 (benzothiazole*C*(2)), 166.1 (*C*(2)O).

(*S*)-*N*,*N*,3-Trimethyl-1-oxo-2,3-dihydro-1*H*-benzo[4,5]thiazolo[3,2-*a*]pyridine-4-carboxamide (17A)

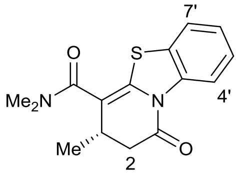

The title compound was prepared according to *General Procedure D* from *(E*)-crotonic anhydride (154 mg, 1.00 mmol), and 2-(1,3-benzothiazol-2-yl)-*N*,*N*-dimethylacetamide (158 mg, 0.72 mmol), EtN(*i*Pr)$_2$ (140 μL, 0.80 mmol) and HyperBTM (11.1 mg, 0.036 mmol) in THF (2 mL) and purified by chromatography on silica gel (10% EtOAc/CH_2Cl_2→10% EtOAc/CH_2Cl_2) to give **17A** as a yellow oil (146 mg, 71%); $[\alpha]_D^{20}$ +9.7 (*c* 1.5 in CH_2Cl_2); chiral HPLC analysis, ChiralPak AD-H (20% *i*-PrOH:hexane, flow rate 1.0 mL min^{-1}, 211 nm, 30 °C), t_R major: 9.6 min, t_R minor: 13.6 min, 94% ee; ν_{max} (film)/cm^{-1} 2963 (C-H), 2926 (C-H), 1699 (br, 2 × C=O), 1622 (C=C); δ_H (500 MHz, $CDCl_3$) 1.19 (3H, d, *J* 6.9, C(3)C*H*$_3$), 2.59 (1H, dd, *J* 16.1, 6.9, C(2)*H*H), 2.90 (1H, dd, *J* 16.1, 6.3, C(2)*H*H), 3.01 (1H, h, *J* 7.0, C(3)*H*), 3.07 (6H, s, N(C*H*$_3$)$_2$), 7.12 (1H, td, *J* 7.6, 1.2, C(5')*H*), 7.18-7.21 (1H, m, C(6')*H*), 7.21-7.24 (1H, m, C(7')*H*), 8.31-8.39 (1H, m, C(4')*H*); δ_c (126 MHz, $CDCl_3$) 18.7 (C(3)*C*H$_3$), 29.6 (*C*(3)H), 36.7 (2 × N(*C*H$_3$)$_2$), 40.4 (*C*(2)H$_2$), 108.5 (*C*(4)), 117.4 (*C*(4')H), 121.2 (*C*(7')H), 125.2 (*C*(6')H), 125.4 (*C*(7a')), 126.2 (*C*(5')H), 137.3 (*C*(4a')), 138.3 (*C*(5)), 168.3 (*C*(1)O), 169.5 (*C*ONMe$_2$); *m/z* (NSI$^+$) 289 ([M+H]$^+$, 20%), 311 ([M+Na]$^+$,40%), 327 ([M+K]$^+$, 90%) plus degradation peaks; HRMS (NSI$^+$) $C_{15}H_{16}N_2O_2S$ ([M+H]$^+$) requires 289.1005, found 289.1005 (–0.1 ppm).

(*S*)-4-Benzoyl-3-methyl-2,3-dihydro-1*H*-benzo[4,5]thiazolo[3,2-*a*]pyridin-1-one (18A) and (*S*)-5-(benzo[*d*]thiazol-2-yl)-4-methyl-6-phenyl-3,4-dihydro-2*H*-pyran-2-one (18B)

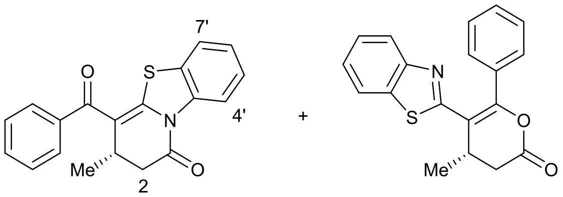

18A **18B**

The title compounds were prepared according to *General Procedure D* from *(E)*-crotonic anhydride (154 mg, 1.00 mmol) and 2-phenacyl benzothiazole (182 mg, 0.72 mmol), EtN(*i*Pr)$_2$ (140 µL, 0.80 mmol) and HyperBTM (11.1 mg, 0.036 mmol) in THF (2 mL) and purified by chromatography on silica gel (10:2 CH_2Cl_2/hexane→CH_2Cl_2) to give **18A** as a yellow solid (126 mg, 55%) and **18B** as a yellow oil (21 mg, 9%).

18A (major): mp 156-157 °C; $[\alpha]_D^{20}$ +93.2 (*c* 0.5 in CH_2Cl_2); chiral HPLC analysis, ChiralPak AD-H (20% *i*-PrOH:hexane, flow rate 1 mL min^{-1}, 211 nm, 30 °C), t_R major: 14.6 min, t_R minor: 11.6 min, 86% ee; ν_{max} (film)/cm^{-1} 3420 (C-H), 2963 (C-H), 1713 (C=O), 1602 (C=O); δ_H (400 MHz, $CDCl_3$) 1.08 (3H, d, *J* 7.0, C*H*$_3$), 2.69 (1H, dd, *J* 16.0, 2.2, C(2)*H*H), 3.04 (1H, dd, *J* 16.0, 6.3, C(2)*H*H), 3.28 (1H, pd, *J* 6.9, 2.2, C(3)*H*), 7.22-7.31 (1H, m, Ar*H*), 7.28-7.38 (1H, m, Ar*H*), 7.39-7.46 (3H, m, Ar*H*), 7.46 – 7.53 (3H, m, Ar*H*), 8.48-8.54 (1H, m, C(4')*H*); δ_C (101 MHz, $CDCl_3$) 19.6 (*C*H$_3$), 27.8 (*C*(3)H), 40.0 (*C*(2)H$_2$), 110.5 (*C*(4)), 117.5 (*C*(4')H), 121.9 (*C*(7')H), 125.8 (*C*(6')H), 126.8 (2 × C(4)COPh*C*(3)H), 126.9 (*C*(5')H), 127.7 (*C*(7a')), 128.4 (2 × C(4)COPh*C*(2)H), 130.0 (C(4)COPh*C*(4)H), 136.1 (*C*(4a')), 140.0 (C(4)COPh*C*(1)), 154.2 (*C*(5)), 168.9 (*C*(1)O), 191.4 (C(4)*C*OPh); *m/z* (NSI$^+$) 322 ([M+H]$^+$, 100%); HRMS (NSI$^+$) $C_{19}H_{16}NO_2S$ ([M+H]$^+$) requires 322.0896, found 322.0901 (+1.5 ppm).

18B (minor): $[\alpha]_D^{20}$ +21.0 (*c* 0.2 in CH_2Cl_2); chiral HPLC analysis, ChiralPak AD-H (5% *i*-PrOH:hexane, flow rate 1 mL min^{-1}, 270 nm, 30 °C), t_R major: 18.9 min, t_R minor: 9.5 min, 92% ee; ν_{max} (film)/cm^{-1} 3057 (C-H), 2963 (C-H), 1775 (C=O); δ_H (500 MHz, $CDCl_3$) 1.30 (3H, d, *J* 7.1, C*H*$_3$), 2.79 (1H, dd, *J* 15.7, 2.0, C(3)*H*$_2$), 3.02 (1H, dd, *J* 15.7, 6.7, C(3)*H*$_2$), 3.82 (1H, pd, *J* 7.1, 2.0, C(4)*H*), 7.28-7.35 (1H, m, benzothiazoleC(6)*H*), 7.37-7.52 (6H, m, Ar*H*), 7.63-7.68 (1H, m, benzothiazoleC(4)*H*), 7.95-8.01 (1H, m, benzothiazoleC(7)*H*); δ_C (126 MHz, $CDCl_3$) 19.0 (*C*H$_3$), 30.8 (*C*(4)H), 36.0 (*C*(3)H$_2$), 117.9 (*C*(5)), 121.3 (benzothiazole*C*(4)H), 123.0 (benzothiazole*C*(7)H), 125.5 (benzothiazole*C*(4)H), 126.2 (benzothiazole*C*(5)H), 129.0 (2 × C(6)Ph*C*(3)H), 130.1 (2 × C(6)Ph*C*(2)H), 130.7

(C(6)Ph*C*(4)H), 132.1 (C(6)Ph*C*(1)), 135.7 (benzothiazole*C*(7a)), 152.6 (benzothiazole*C*(4a)), 153.3 (*C*(6)), 164.2 (benzothiazole*C*(2)), 167.6 (*C*(2)O); *m/z* (NSI^+) 322 ($[M+H]^+$, 100%); HRMS (NSI^+) $C_{19}H_{16}NO_2S$ ($[M+H]^+$) requires 322.0896, found 322.0901 (+1.5 ppm).

(*S*)-5-(Benzo[*d*]oxazol-2-yl)-4-methyl-6-phenyl-3,4-dihydro-2*H*-pyran-2-one (19B)

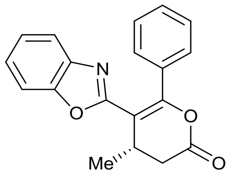

The title compound was prepared according to *General Procedure D* from *(E)*-crotonic anhydride (154 mg, 1.00 mmol), and 2-phenacylbenzoxazole (171 mg, 0.72 mmol), EtN(*i*Pr)$_2$ (140 μL, 0.80 mmol) and HyperBTM (11.1 mg, 0.036 mmol) in THF (2 mL) and purified by chromatography on silica gel (CH_2Cl_2→10% EtOAc/CH_2Cl_2) to give **19B** as a white solid (169 mg, 77%); mp 107-109 °C; $[\alpha]_D^{20}$ +82.8 (*c* 1.0 in CH_2Cl_2); chiral HPLC analysis, ChiralPak AD-H (5% *i*-PrOH:hexane, flow rate 1.0 mL min^{-1}, 270 nm, 30 °C), t_R major: 15.3 min, t_R minor: 9.1 min, 93% ee; ν_{max} (film)/cm^{-1} 2974 (C-H), 2914 (C-H), 1761 (C=O), 1639 (C=C); δ_H (400 MHz, $CDCl_3$) 1.36 (3H, d, *J* 7.1 C*H*$_3$), 2.81 (1H, dd, *J* 15.8, 2.1, C(3)*H*$_2$), 3.01 (1H, dd, *J* 15.7, 6.6, C(3)*H*$_2$), 3.68 (1H, pd, *J* 7.1, 2.1, C(4)*H*), 7.23-7.36 (3H, m, Ar*H*), 7.36-7.42 (2H, m, 2 × C(6)PhC(3)*H*), 7.43-7.50 (3H, m, Ar*H*), 7.70-7.76 (1H, m, benzoxazoleC(7)*H*); δ_C (101 MHz, $CDCl_3$) 19.1 (*C*H$_3$), 29.8 (*C*(4)H), 35.6 (*C*(3)H$_2$), 109.9 (*C*(5)), 110.5 (benzoxazole*C*(7)H), 119.8 (benzoxazole*C*(4)H), 124.6 (benzoxazole*C*(6)H), 125.3 (benzoxazole*C*(5)H), 128.1 (2 × C(6)Ph*C*(3)H), 129.0 (2 × C(6)Ph*C*(2)H), 130.2 (C(6)Ph*C*(4)H), 132.8 (C(6)Ph*C*(1)), 141.5 (benzoxazole*C*(4a)), 150.2 (benzoxazole*C*(7a)), 154.9 (*C*(6)), 161.1 (benzoxazole*C*(2)), 167.0 (*C*(2)O); *m/z* (NSI^+) 346 ($[M+MeCN]^+$, 100%), 603 ($[M+H]^+$, 20%); HRMS (NSI^+) $C_{19}H_{16}NO_3$ ($[M+H]^+$) requires 306.1125, found 306.1124 (–0.2 ppm).

(*S*)-3-(Furan-2-yl)-*N*,*N*-dimethyl-1-oxo-2,3-dihydro-1*H*-benzo[4,5]thiazolo[3,2-*a*]pyridine-4-carboxamide (20A)

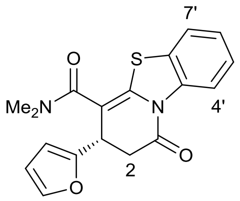

The title compound was prepared according to *General Procedure D* from (*E*)-3-(furan-2-yl)acrylic anhydride (258 mg, 1.00 mmol), and 2-(1,3-benzothiazol-2-yl)-*N*,*N*-dimethylacetamide (158 mg, 0.72 mmol), EtN(*i*Pr)$_2$ (140 μL, 0.80 mmol) and HyperBTM (11.1 mg, 0.036 mmol) in THF (2 mL) and purified by chromatography on silica gel (CH_2Cl_2→10% EtOAc/CH_2Cl_2) to give **20A** as a brown oil (193 mg, 79%); $[\alpha]_D^{20}$ –109.3 (*c* 0.5 in CH_2Cl_2); chiral HPLC analysis, ChiralPak AD-H (20% *i*-PrOH:hexane, flow rate 1.0 mL min^{-1}, 211 nm, 30 °C), t_R major: 14.4 min, t_R minor: 20.3 min, 95% ee; ν_{max} (film)/cm^{-1} 2974 (C-H), 2928 (C-H), 1705 (br, 2 × C=O), 1616 (C=C), 1583 (furan), 1489 (furan); δ_H (500 MHz, $CDCl_3$) 2.97 (6H, s, N(C*H*$_3$)$_2$), 3.10 (1H, dd, *J* 16.2, 3.9, C(2)*H*H), 3.18 (1H, dd, *J* 16.4, 7.0, C(2)*H*H), 4.26 (1H, dd, *J* 7.1, 3.9 C(3)*H*), 6.15 (1H, d, *J* 3.2 (C(3)furylC(3)*H*), 6.28 (1H, dd, *J* 3.3, 1.9, C(3)furylC(4)*H*), 7.14-7.19 (1H, m, C(5')*H*), 7.21-7.27 (1H, m, C(6')*H*), 7.27-7.31 (1H, m C(3)furylC(5)*H*), 7.32 (1H, dd, *J* 1.9, 0.9, C(7')*H*), 8.38 (1H, dd, *J* 8.2, 1.1, C(4')*H*); δ_C (126 MHz, $CDCl_3$) 33.7 (*C*(3)H), 37.0 (2 × N(*C*H$_3$)$_2$), 37.4 (*C*(2)H$_2$), 103.6 (*C*(4)), 106.2 (C(3)furyl*C*(3)H), 110.6 (C(3)furyl*C*(4)H), 117.7 (*C*(4')H), 121.3 (*C*(7')H), 125.4 (*C*(6')H), 125.6 (*C*(7a')), 126.4 (*C*(5')H), 137.4 (*C*(4a')), 141.6 (C(3)furyl*C*(2)), 142.4 (C(3)furyl*C*(5)H), 153.4 (*C*(5)), 167.6 (*C*(1)O), 169.2 (*C*ONMe$_2$); *m/z* (NSI$^+$) 341 ([M+H]$^+$, 15%), 363 ([M+Na]$^+$, 100%), 379 ([M+K]$^+$, 10%); HRMS (NSI$^+$) $C_{18}H_{17}N_2O_3S$ ([M+H]$^+$) requires 341.0954, found 341.0949 (–1.6 ppm).

(11*S*)-10-Benzoyl-11-(furan-2-yl)-8-thia-1-azatricyclo[7.4.0.0^{2,7}]trideca-2,4,6,9-tetraen-13-one (21A) and (4*S*)-5-(1,3-benzothiazol-2-yl)-4-(furan-2-yl)-6-phenyl-3,4-dihydro-2H-pyran-2-one (21B)

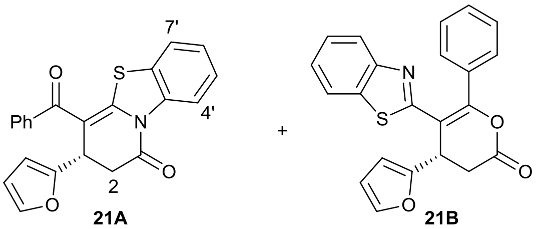

The title compounds were prepared according to *General Procedure D* from (*E*)-3-(furan-2-yl)acrylic anhydride (430 mg, 1.67 mmol) and 2-phenacyl benzothiazole (421 mg, 1.67 mmol), EtN(*i*Pr)$_2$ (319 μL, 1.8 mmol) and HyperBTM (5 mg, 1 mol %) in THF (3 mL) and purified by chromatography (20% hexanes/CH_2Cl_2) to afford the title compounds **21A** as a yellow solid and **21B** as a yellow solid.

21A (major): (446 mg, 72%); mp 127-128 °C; $[\alpha]_D^{20}$ –30.3 (*c* 1.0, $CHCl_3$); HPLC analysis, ChiralPak AD-H (20% *i*-PrOH:hexane, flow rate 1.0 mL min^{-1}, 211 nm, 30 °C), t_R minor: 15.0 min, t_R major: 20.3 min, 80% ee; ν_{max} (film)/cm^{-1} 3142 (C-H), 3136 (C-H), 2363 (C-H), 1732 (C=O), 1626 (C=C), 1605 (C=C), 1474 (C-N), 1362 (C-S), 1275 (C-O); δ_H (400 MHz, $CDCl_3$) 3.16 (1H, dd, *J* 16.1, 6.0, C*H*$_2$), 3.24 (1H, dd, *J* 16.1 2.6, C*H*$_2$), 4.43 (1H, ddd, *J* 6.1, 2.6, 1.0, C(11)*H*), 5.95 (1H, m, furanylC(x)*H*), 6.24 (1H, dd, *J* 3.3, 1.9, furanylC(4)*H*), 7.29-7.51 (8H, m, Ar*H*), 7.54-7.61 (1H, m, Ar*H*), 8.46-8.53 (1H, m, Ar*H*); δ_C (100 MHz, $CDCl_3$) 32.9 (*C*(11)H), 37.9 (*C*12)H$_2$), 105.6 (*C*(10)), 106.8 (furanyl*C*(3)H), 110.3 (furanyl*C*(4)H), 117.6 (Ar*C*H,), 121.9 (Ar*C*H), 125.8 (Ar*C*H), 126.9 (2×Ar*C*H), 127.0 (Ar*C*H), 127.5 (*C*(7)), 128.2 (2×Ar*C*H), 130.4 (Ar*C*H), 136.0 (C(10)Ar*C*(1)), 139.3 (*C*(2)), 142.7 (furanyl*C*(5)H), 153.5 (furanyl*C*(1)), 156.6 (*C*(9)), 167.7 (*C*(13)=O), 190.8 (C(10)*C*=O).

21B (minor): (60 mg, 10%) mp 122-125 °C; $[\alpha]_D^{22}$ –6.2 (*c* 1.0 in CH_2Cl_2); chiral HPLC analysis, ChiralPak AD-H (20% *i*-PrOH:hexane, flow rate 1 mL min^{-1}, 211 nm, 30 °C), t_R minor: 7.6 min, t_R major: 15.1 min, 80% ee; ν_{max} (film)/cm^{-1} 3117 (C-H), 3063 (C-H), 2924 (C-H), 1782 (C=O), 1649 (C=N), 1597 (C=C), 1344 (C-S), 1277 (C-O); δ_H (300 MHz, $CDCl_3$) 3.20 (1H, dd, *J* 15.9, 6.7, C(3)*H*$_2$), 3.26 (1H, dd, *J* 15.9, 1.9, C(3)*H*$_2$), 5.14 (1H, dd, *J* 6.8, 2.0, C(4)*H*), 6.18 (1H, d, *J* 3.3, furanyl(3)*H*), 6.24 (1H, dd, J 3.3, 1.8, furanyl(4)*H*), 7.31 (1H, t, *J* 7.6, Ar*H*), 7.34 (1H, d, *J* 1.8, furanyl(5)*H*), 7.44 (3H, td, *J* 7.3, 1.5, Ar*H*), 7.49-7.57 (3H, m, Ar*H*), 7.61-7.68 (1H, m, Ar*H*), 7.98 (1H, d, *J* 8.2, Ar*H*); δ_C (75 MHz, $CDCl_3$) 33.9 (*C*(3)), 35.1 (*C*(4)), 106.9 (furanyl*C*(3)H), 110.4 (furanyl*C*(4)H), 113.3 (*C*(5)), 121.3

(C(5)HetAr*C*(7)H), 123.1 (C(5)HetAr*C*(4)H), 125.6 (C(5)HetAr*C*(6)H), 126.2 (C(5)HetAr*C*(5)H), 129.0 (2×C(6)Ph*C*(3)H), 130.1 (2×C(6)Ph*C*(2)H), 131.0 (C(6)Ph*C*(4)H), 131.8 (C(6)Ph*C*(1)), 135.7 (C(5)HetAr*C*(7a)), 142.8 (furanyl*C*(5)H), 152.2 (furanyl*C*(2)), 152.4 (C(5)HetAr*C*(3a)), 154.8 (*C*(6)), 164.0 (C(5)HetAr*C*=N), 166.4 (*C*(2)O).

(*S*)-5-(Benzo[d]oxazol-2-yl)-4-(furan-2-yl)-6-phenyl-3,4-dihydro-2H-pyran-2-one (22B)

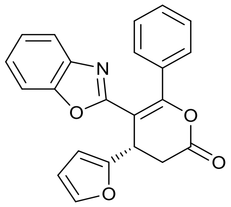

The title compound was prepared according to *General Procedure D* from (*E*)-3-(furan-2-yl)acrylic anhydride (258 mg, 1.00 mmol), and 2-phenacylbenzoxazole (171 mg, 0.72 mmol), EtN(*i*Pr)$_2$ (140 μL, 0.80 mmol) and HyperBTM (11.1 mg, 0.036 mmol) in THF (2 mL) and purified by chromatography on silica gel (CH_2Cl_2→10% EtOAc/CH_2Cl_2) to give **22B** as a brown/green solid (214 mg, 83%); mp 114-116 °C; $[\alpha]_D^{20}$ +85.2 (*c* 1.0 in CH_2Cl_2); chiral HPLC analysis, ChiralPak AD-H (20% *i*-PrOH:hexane, flow rate 1.0 mL min^{-1}, 211 nm, 30 °C), t_R major: 10.9 min, t_R minor: 7.7 min, 97% ee; ν_{max} (film)/cm^{-1} 2972 (C-H), 2926 (C-H), 1782 (C=O), 1645 (C=C), 1531 (furan), 1452 (furan); δ_H (400 MHz, $CDCl_3$) 3.17 (1H, dd, *J* 15.9, 6.9, C(3)*H*$_2$), 3.27 (1H, dd, *J* 15.9, 2.0, C(3)*H*$_2$), 4.96 (1H, dd, *J* 6.9, 1.9, C(4)*H*), 6.23-6.27 (2H, m, furylC(3)*H* and furylC(4)*H*), 7.21-7.36 (4H, m, Ar*H*), 7.37-7.42 (2H, m, 2 × C(6)PhC(3)*H*), 7.44-7.50 (1H, m, benzoxazoleC(7)*H*), 7.50-7.54 (2H, m, 2 × C(6)PhC(2)*H*), 7.69-7.76 (1H, m, benzoxazoleC(4)*H*); δ_C (101 MHz, $CDCl_3$) 33.4 (*C*(3)H$_2$), 34.2 (*C*(4)H), 105.6 (*C*(5)), 106.8 (furyl*C*(4)H), 110.4 (furyl*C*(3)H), 110.5 (benzoxazole*C*(7)H), 119.9 (benzoxazole*C*(4)H), 124.6 (benzoxazole*C*(6)H), 125.4 (benzoxazole*C*(5)H), 128.1 (2 × C(6)Ph*C*(3)H), 129.0 (2 × C(6)Ph*C*(2)H), 130.5 (C(6)Ph*C*(4)H), 132.5 (C(6)Ph*C*(1)), 141.4 (benzoxazole*C*(4a)), 142.8 (furyl*C*(5)H), 150.2 (benzoxazole*C*(7a)), 152.1 (furyl*C*(2)), 156.2 (*C*(6)), 160.8 (benzoxazole*C*(2)), 165.9 (*C*(2)O); *m/z* (NSI$^+$) 358 ([M+H]$^+$, 20%), 375 ([M+NH$_4$]$^+$, 75%), 390 ([M+CH$_3$OH+H]$^+$, 100%); HRMS (NSI$^+$) $C_{22}H_{16}NO_4$ ([M+H]$^+$) requires 358.1074, found 358.1079 (+1.4 ppm).

(*R*)-3-(Furan-3-yl)-*N*,*N*-dimethyl-1-oxo-2,3-dihydro-1*H*-benzo[4,5]thiazolo[3,2-*a*]pyridine-4-carboxamide (23A)

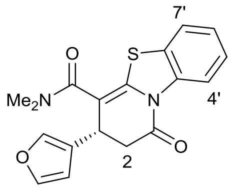

The title compound was prepared according to *General Procedure D* from (2*E*)-3-(furan-3-yl)prop-2-enoic anhydride (258 mg, 1.00 mmol) and 2-(1,3-benzothiazol-2-yl)-*N*,*N*-dimethylacetamide (158 mg, 0.72 mmol), EtN(*i*Pr)$_2$ (140 µL, 0.80 mmol) and HyperBTM (11.1 mg, 0.036 mmol) in THF (2 mL) and purified by chromatography on silica gel (10% EtOAc/CH_2Cl_2) to give **23A** as a yellow oil (130 mg, 53%); $[\alpha]_D^{20}$ –53.7 (*c* 1.0 in CH_2Cl_2); chiral HPLC analysis, ChiralPak OJ-H (20% *i*-PrOH:hexane, flow rate 1 mL min^{-1}, 211 nm, 30 °C), t_R major: 24.5 min, t_R minor: 19.4 min, 96% ee; ν_{max} (film)/cm^{-1} 2926 (C-H), 1701 (C=O), 1614 (C=O), 1583 (C=N), 1462 (C=C); δ_H (300 MHz, $CDCl_3$) 2.85 (1H, dd, *J* 4.9, 16.1, C(2)*H*H), 2.89 (6H, s, N(C*H*$_3$)$_2$), 3.02 (1H, dd, *J* 16.1, 6.6, C(2)*H*H), 3.95 – 4.06 (1H, m, C(3)*H*), 6.22 (1H, dd, *J* 1.9, 1.0, furylC(4)*H*), 7.06 (1H, td, *J* 7.5, 1.4, C(5')*H*), 7.09-7.16 (1H, m, C(6')*H*), 7.16-7.21 (1H, m, C(7')*H*), 7.24 (1H, dt, *J* 1.7, 0.9, furylC(2)*H*), 7.27 (1H, t, *J* 1.7, furylC(5)*H*), 8.20-8.29 (1H, m, C(4')*H*); δ_C (75 MHz, $CDCl_3$) 31.0 (*C*(3)H), 37.1 (2 × N(*C*H$_3$)$_2$), 39.5 (*C*(2)H$_2$), 105.7 (*C*(4)), 109.3 (furyl*C*(4)H), 117.5 (*C*(4')H), 121.2 (*C*(7')H), 124.7 (furyl*C*(3)), 125.3 (*C*(6')H), 125.8 (*C*(7a')), 126.2 (*C*(5')H), 137.1 (*C*(4a')), 139.3 (furyl*C*(2)H), 142.0 (*C*(5)), 143.9 (furyl*C*(5)H), 167.8 (*C*(1)O), 169.2 (*C*ONMe$_2$); *m/z* (APCI$^+$) 341 ([M+H]$^+$, 100%); HRMS (APCI$^+$) $C_{18}H_{17}O_3N_2S$ ([M+H]$^+$) requires 341.0954, found 341.0953 (–0.4 ppm).

(*R*)-4-Benzoyl-3-(furan-3-yl)-2,3-dihydro-1*H*-benzo[4,5]thiazolo[3,2-*a*]pyridin-1-one (24A) and (*R*)-5-(benzo[*d*]thiazol-2-yl)-4-(furan-3-yl)-6-phenyl-3,4-dihydro-2*H*-pyran-2-one (24B)

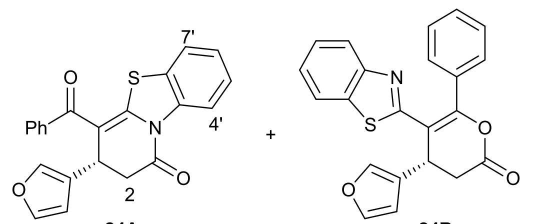

24A 24B

The title compounds were prepared according to *General Procedure D* from (2*E*)-3-(furan-3-yl)prop-2-enoic anhydride (258 mg, 1.00 mmol) and 2-phenacyl benzothiazole (182 mg, 0.72 mmol), EtN(*i*Pr)$_2$ (140 μL, 0.80 mmol) and HyperBTM (11.1 mg, 0.036 mmol) in THF (2 mL) and purified by chromatography on silica gel (10:2 CH_2Cl_2/hexane) to give **24A** as a yellow solid (132 mg, 40%) and **24B** as a yellow oil (15 mg, 5%).

24A (major): mp 92-94 ˚C; $[\alpha]_D^{20}$ +11.2 (*c* 0.5, CH_2Cl_2); chiral HPLC analysis, ChiralPak AD-H (20% *i*-PrOH:hexane, flow rate 1 mL min^{-1}, 211 nm, 30 °C), t_R major: 31.0 min, t_R minor: 17.0 min, 90% ee; ν_{max} (film)/cm^{-1} 3063 (C-H), 1721 (C=O), 1607 (C=O), 1574 (C=N), 1474 (C=C); δ_H (300 MHz, $CDCl_3$) 3.04 (1H, dd, *J* 15.9, 2.4, C(2)*H*H), 3.20 (1H, dd, *J* 16.0, 6.1, C(2)*H*H), 4.32 (1H, ddd, *J* 6.2, 2.4, 1.1, C(3)*H*), 6.21 (1H, dd, *J* 1.9, 1.0, furylC(4)*H*), 7.16 (1H, q, *J* 1.2, furylC(2)*H*), 7.27-7.47 (6H, m, Ar*H*), 7.49-7.59 (3H, m, Ar*H*), 8.39-8.59 (1H, m, C(4'3)*H*); δ_C (75 MHz, $CDCl_3$) 30.3 (*C*(3)H), 40.0 (*C*(2)H_2), 107.9 (*C*(4)), 109.3 (furyl*C*(4)H), 117.6 (*C*(4')H), 122.0 (*C*(7')H), 125.6 (furyl*C*(2)H), 125.9 (*C*(6')H), 127.1 (*C*(5')H), 127.1 (2 × C(4)COPh*C*(2)H), 127.7 (*C*(7a')), 128.3 (2 × C(4)COPh*C*(3)H), 130.5 (C(4)COPh*C*(4)H), 136.1 (*C*(4a')), 139.4 (C(4)COPh*C*(1)), 139.7 (furyl*C*(2)H), 144.2 (furyl*C*(5)H), 156.0 (*C*(5)), 168.1 (*C*(1)O), 190.6 (C(4)*C*O); *m/z* (APCI$^+$) 374 ([M+H]$^+$, 100%); HRMS (APCI$^+$) $C_{22}H_{16}O_3NS$ ([M+H]$^+$) requires 374.0845, found 374.0841 (–1.2 ppm).

24B (minor): $[\alpha]_D^{20}$ +65.5 (*c* 0.2, CH_2Cl_2); chiral HPLC analysis, ChiralPak AD-H (20% *i*-PrOH:hexane, flow rate 1 mL min^{-1}, 211 nm, 30 °C), t_R major: 13.9 min, t_R minor: 7.9 min, 94% ee; ν_{max} (film)/cm^{-1} 3117 (C-H), 3065 (C-H), 2920 (C-H), 1782 (C=O), 1647 (C=N), 1597 (C=C), 1343 (C=N); δ_H (300 MHz, $CDCl_3$) 3.11 (1H, dd, *J* 15.8, 2.2, C(3)*H*H)), 3.21 (1H, dd, *J* 15.8, 6.5, C(3)*H*H), 4.87-5.09 (1H, m, C(4)*H*), 6.32 (1H, dd, *J* 1.9, 1.0, furylC(4)*H*), 7.28-7.37 (3H, m, Ar*H*), 7.39-7.47 (3H, m, Ar*H*), 7.47-7.55 (3H, m, Ar*H*), 7.64

(1H, dt, *J* 7.9, 0.9, benzothiazoleC(4)*H*), 7.97 (1H, dt, *J* 8.1, 0.9, benzothiazoleC(7)*H*); δ_C (126 MHz, $CDCl_3$) 32.3 (*C*(4)H), 35.5 (*C*(3)H_2), 109.5 (furyl*C*(4)H), 115.8 (*C*(5)), 121.4 (benzothiazole*C*(7)H), 123.2 (benzothiazole*C*(4)H), 124.2 (furyl*C*(3)), 125.6 (benzothiazole*C*(6)H), 126.2 (benzothiazole*C*(5)H), 129.1 (2 × C(6)Ph*C*(2)H), 130.1 (2 × C(6)Ph*C*(3)H), 131.0 (C(6)Ph*C*(4)H), 131.8 (C(6)Ph*C*(1)), 135.8 (benzothiazole*C*(4a)), 139.5 (furyl*C*(2)H), 143.9 (furyl*C*(5)H), 152.5 (benzothiazole*C*(7a)), 154.18 (*C*(6)), 164.0 (benzothiazole*C*(2)), 167.0 (*C*(2)O); *m/z* ($APCI^+$) 374 ($[M+H]^+$, 100%); HRMS ($APCI^+$) $C_{22}H_{16}O_3NS$ ($[M+H]^+$) requires 374.0845, found 374.0845 (–0.1 ppm).

(*R*)-5-(Benzo[d]oxazol-2-yl)-4-(furan-3-yl)-6-phenyl-3,4-dihydro-2H-pyran-2-one (25B)

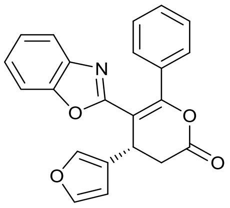

The title compund was prepared according to *General Procedure D* from (2*E*)-3-(furan-3-yl)prop-2-enoic anhydride (258 mg, 1.00 mmol) and 2-phenacyl benzoxazole (171 mg, 0.72 mmol), EtN(*i*Pr)$_2$ (140 μL, 0.80 mmol) and HyperBTM (11.1 mg, 0.036 mmol) in THF (2 mL) and purified by chromatography on silica gel (20% EtOAc/petrol) to give **25B** as a yellow oil (30 mg, 12%); $[\alpha]_D^{20}$ +89.1 (*c* 1.5, CH_2Cl_2); chiral HPLC analysis, ChiralPak AD-H (20% *i*-PrOH:hexane, flow rate 1 mL min^{-1}, 211 nm, 30 °C), t_R major: 11.2 min, t_R minor: 8.4 min, 95% ee; ν_{max} (film)/cm^{-1} 2961 (C-H), 2930 (C-H), 1778 (C=O), 1647 (C=N), 1452 (C=C); δ_H (300 MHz, $CDCl_3$) 3.10 (1H, dd, *J* 15.8, 2.5, C(3)*H*H), 3.18 (1H, dd, *J* 15.7, 6.3, C(3)*H*H), 4.76 (1H, ddd, *J* 6.4, 2.5, 0.9, C(4)*H*), 6.36 (1H, dd, *J* 1.8, 0.9, furylC(4)*H*), 7.18-7.32 (3H, m, Ar*H*), 7.32-7.42 (4H, m, Ar*H*), 7.42-7.50 (3H, m, Ar*H*), 7.66-7.72 (1H, m, benzoxazoleC(4)*H*); δ_C (126 MHz, $CDCl_3$) 31.7 (*C*(4)H), 35.3 (*C*(3)H_2), 107.9 (*C*(5)), 109.3 (furyl*C*(4)H), 110.6 (benzoxazole*C*(7)H), 120.0 (benzoxazole*C*(4)H), 124.1 (furyl*C*(3)), 124.7 (benzoxazole*C*(6)H), 125.5 (benzoxazole*C*(5)H), 128.3 (2 × C(6)Ph*C*(2)H), 129.1 (2 × C(6)Ph*C*(3)H), 130.6 (C(6)Ph*C*(4)H), 132.6 (C(6)Ph*C*(1)), 139.5 (furyl*C*(2)H), 141.6 (benzoxazole*C*(4a)), 144.1 (furyl*C*(5)H), 150.3 (benzoxazole*C*(7a)), 155.7 (*C*(6)), 161.0 (benzoxazole*C*(2)), 166.5 (*C*(2)O); *m/z* ($APCI^+$) 358 ($[M+H]^+$, 100%); HRMS ($APCI^+$) $C_{22}H_{16}O_4N$ ($[M+H]^+$) requires 358.1074, found 358.1071 (–0.8 ppm).

(*R*)-*N*,*N*-Dimethyl-1-oxo-3-(thiophen-3-yl)-2,3-dihydro-1*H*-benzo[4,5]thiazolo[3,2-*a*]pyridine-4-carboxamide (26A)

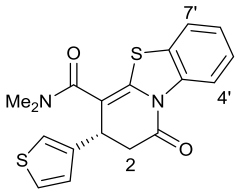

The title compund was prepared according to *General Procedure D* from (2*E*)-3-(thiophen-3-yl)prop-2-enoic anhydride (290 mg, 1.00 mmol) and 2-(1,3-benzothiazol-2-yl)-*N*,*N*-dimethylacetamide (158 mg, 0.72 mmol), EtN(*i*Pr)$_2$ (140 μL, 0.80 mmol) and HyperBTM (11.1 mg, 0.036 mmol) in THF (2 mL) and purified by chromatography on silica gel (CH_2Cl_2→10% EtOAc/CH_2Cl_2) to give **26A** as a yellow oil (148 mg, 58%); $[\alpha]_D^{20}$ –85.6 (*c* 1.0, CH_2Cl_2); chiral HPLC analysis, ChiralPak AD-H (20% *i*-PrOH:hexane, flow rate 1 mL min^{-1}, 211 nm, 30 °C), t_R major: 25.6 min, t_R minor: 22.8 min, 96% ee; ν_{max} (film)/cm^{-1} 3069 (C-H), 2926 (C-H), 1701 (C=O), 1613 (C=O), 1483 (C=N), 1452 (C=C); δ_H (300 MHz, $CDCl_3$) 2.91 (6H, s, N(C*H*$_3$)$_2$), 3.01 (1H, dd, *J* 16.2, 4.7, C(2)*H*H), 3.16 (1H, dd, *J* 16.2, 6.8, C(2)*H*H), 4.27 (1H, dd, *J* 6.7, 4.9, C(3)*H*), 6.95 (1H, dd, *J* 5.0, 1.4, thiopheneC(4)*H*), 7.05-7.11 (1H, m, thiopheneC(2)*H*), 7.12-7.18 (1H, m, Ar*H*), 7.19-7.25 (1H, m, Ar*H*), 7.25-7.32 (2H, m, Ar*H*), 8.31-8.38 (1H, m, C(4')*H*); δ_C (126 MHz, $CDCl_3$) 35.4 (*C*(3)H), 37.1 (2 × N(*C*H$_3$)$_2$), 39.9, (*C*(2)H$_2$), 106.2 (*C*(4)), 117.6 (*C*(4')H), 121.3 (thiophene*C*(2)H), 121.3 (*C*(7')H), 125.4 (*C*(6')H), 126.0 (*C*(7')), 126.4 (*C*(5')H), 126.4 (thiophene*C*(5)H), 127.1 (thiophene*C*(4)H), 137.3 (*C*(4a')), 141.2 (*C*(5)), 142.0 (thiophene*C*(3)), 167.9 (*C*(1)O), 169.4 (*C*ONMe$_2$); *m/z* (APCI$^+$) 357 ([M+H]$^+$, 100%); HRMS (APCI$^+$) $C_{18}H_{17}O_2N_2S_2$ ([M+H]$^+$) requires 357.0726, found 357.0725 (–0.3 ppm).

(*R*)-4-Benzoyl-3-(thiophen-3-yl)-2,3-dihydro-1*H*-benzo[4,5]thiazolo[3,2-*a*]pyridin-1-one (27A) and (*R*)-5-(benzo[*d*]thiazol-2-yl)-6-phenyl-4-(thiophen-3-yl)-3,4-dihydro-2*H*-pyran-2-one (27B)

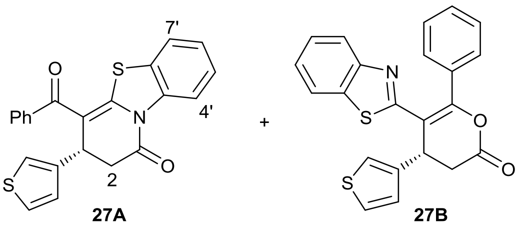

The title compounds were prepared according to *General Procedure D* from (2*E*)-3-(thiophen-3-yl)prop-2-enoic anhydride (290 mg, 1.00 mmol) and 2-phenacyl benzothiazole (182 mg, 0.72 mmol), EtN(*i*Pr)$_2$ (140 μL, 0.80 mmol) and HyperBTM (11.1 mg, 0.036 mmol) in THF (2 mL) and purified by chromatography on silica gel (6:4→10:2 CH_2Cl_2/hexane) to give **27A** as a yellow solid (123 mg, 46%) and **27B** as a yellow solid (16 mg, 6%)

27A (major): mp 154-157 °C; $[\alpha]_D^{20}$ –82.4 (*c* 1.0, CH_2Cl_2); chiral HPLC analysis, ChiralPak AD-H (20% *i*-PrOH:hexane, flow rate 1 mL min^{-1}, 211 nm, 30 °C), t_R major: 31.3 min, t_R minor: 18.2 min, 82% ee; ν_{max} (film)/cm^{-1} 3090 (C-H), 2924 (C-H), 1726 (C=O), 1603 (C=O), 1574 (C=C), 1474 (C=C); δ_H (500 MHz, $CDCl_3$) 3.08 (1H, dd, *J* 16.1, 2.4, C(2)*H*H), 3.22 (1H, dd, *J* 16.0, 6.4, C(2)*H*H), 4.44 (1H, dd, *J* 6.6, 2.4, C(3)*H*), 6.84 (1H, d, *J* 5.0, thiopheneC(4)*H*), 6.92 (1H, d, *J* 3.4, thiopheneC(2)*H*), 7.24-7.46 (8H, m, Ar*H*), 7.57 (1H, d, *J* 7.5, C(7')*H*), 8.46 (1H, d, *J* 8.1, C(4')*H*); δ_C (126 MHz, $CDCl_3$) 34.5 (*C*(3)H), 40.6 (*C*(2)H_2), 108.5 (*C*(4)), 117.7 (*C*(4')H), 121.7 (thiophene*C*(2)H), 122.0 (*C*(7')), 126.0 (*C*(6')H), 126.4 (thiophene*C*(5)H), 127.1 (*C*(5'), 127.2 (2 × C(4)COPh*C*(2)H), 127.5 (thiophene*C*(4)H), 127.8 (*C*(7a')), 128.3 (2 × C(4)COPh*C*(3)H), 130.5 (C(4)COPh*C*(4)H), 136.1 (*C*(4a'), 139.4 (C(4)COPh*C*(1)), 141.8 (thiophene*C*(3)), 156.0 (*C*(5)), 168.1 (*C*(1)O), 191.0 (C(4)*C*OPh); *m/z* (APCI$^+$) 390 ([M+H]$^+$, 100%); HRMS (APCI$^+$) $C_{22}H_{16}O_2NS_2$ ([M+H]$^+$) requires 390.0617, found 390.0617 (+0.0 ppm).

27B (minor): mp 149-152 °C; $[\alpha]_D^{20}$ –52.5 (*c* 0.4, CH_2Cl_2); chiral HPLC analysis, ChiralPak AD-H (20% *i*-PrOH:hexane, flow rate 1 mL min^{-1}, 211 nm, 30 °C), t_R major: 13.7 min, t_R minor: 8.8 min, 85% ee; ν_{max} (film)/cm^{-1} 3102 (C-H), 2920 (C-H), 1780 (C=O), 1647 (C=N), 1595 (C=C), 1431 (C=C); δ_H (500 MHz, $CDCl_3$) 3.18 (1H, dd, *J* 15.8, 2.0, C(3)*H*H), 3.25 (1H, dd, *J* 15.8, 6.9, C(3)*H*H), 5.13 (1H, dd, *J* 6.7, 2.5 (C(4)*H*), 7.01 (1H, dd, *J* 5.0, 1.4, thiopheneC(4)*H*), 7.16-7.18 (1H, m, thiopheneC(2)*H*), 7.25-7.28 (1H, m, Ar*H*), 7.28-7.34 (1H, m, Ar*H*), 7.39-7.45 (3H, m, Ar*H*), 7.48-7.54 (3H, m, Ar*H*), 7.62-7.65 (1H, m,

benzothiazoleC(7)*H*), 7.96 (1H, dt, *J* 8.2, 0.9, benzothiazoleC(4)*H*); δ_C (126 MHz, $CDCl_3$) 36.0 (*C*(3)H_2), 36.6 (*C*(4)H), 116.0 (*C*(5)), 121.4 (benzothiazole*C*(4)H), 121.7 (thiophene*C*(2)H), 123.2 (benzothiazole*C*(7)H), 125.6 (benzothiazole*C*(6)H), 126.2 (benzothiazole*C*(5)H), 126.6 (thiophene*C*(5)H), 127.0 (thiophene*C*(4)H), 129.0 (2 × C(6)Ph*C*(2)H), 130.1 (2 × C(6)Ph*C*(3)H), 130.9 (C(6)Ph*C*(4)H), 131.9 (C(6)Ph*C*(1)), 135.9 (benzothiazole*C*(4a)), 140.2 (thiophene*C*(3)), 152.5 (benzothiazole*C*(7a)), 154.0 (*C*(6)), 164.2 (benzothiazole*C*(2)), 167.0 (*C*(2)O); *m/z* ($APCI^+$) 390 ($[M+H]^+$, 100%); HRMS ($APCI^+$) $C_{22}H_{16}O_2NS_2$ ($[M+H]^+$) requires 390.0617, found 390.0616 (–0.2 ppm).

(*S*)-5-(Benzo[d]oxazol-2-yl)-6-phenyl-4-(thiophen-3-yl)-3,4-dihydro-2H-pyran-2-one (28B)

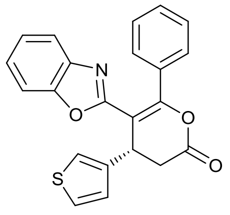

The title compund was prepared according to *General Procedure D* from (2*E*)-3-(thiophen-3-yl)prop-2-enoic anhydride (290 mg, 1.00 mmol) and 2-phenacyl benzoxazole (171 mg, 0.72 mmol), EtN(*i*Pr)$_2$ (140 μL, 0.80 mmol) and HyperBTM (11.1 mg, 0.036 mmol) in THF (2 mL) and purified by chromatography on silica gel (6:4→10:2 CH_2Cl_2/hexane) to give **28B** as a tan solid (120 mg, 45%) ; mp 105-107 °C; $[\alpha]_D^{20}$ +80.4 (*c* 1.0, CH_2Cl_2); chiral HPLC analysis, ChiralPak AD-H (20% *i*-PrOH:hexane, flow rate 1 mL min^{-1}, 211 nm, 30 °C), t_R major: 10.6 min, t_R minor: 8.9 min, 98% ee; ν_{max} (film)/cm^{-1} 3080 (C-H), 2918 (C-H), 1773 (C=O), 1653 (C=N), 1451 (C=C), 1352 (C=C); δ_H (300 MHz, $CDCl_3$) 3.09-3.30 (2H, m, C(3)*H*$_2$), 4.95 (1H, ddd, *J* 6.3, 2.8, 0.9, C(4)*H*), 7.06 (1H, dd, *J* 5.0, 1.4, thiopheneC(4)*H*), 7.16-7.34 (5H, m, Ar*H*), 7.34-7.42 (2H, mAr*H*), 7.42-7.53 (3H, m, Ar*H*), 7.65-7.71 (1H, m, benzoxazoleC(4)*H*); δ_C (126 MHz, $CDCl_3$) 35.8 (*C*(3)H_2), 35.8 (*C*(4)H), 108.1 (*C*(5)), 110.6 (benzoxazole*C*(7)H), 120.0 (benzoxazole*C*(4)H), 121.7 (thiophene*C*(2)H), 124.7 (benzoxazole*C*(6)H), 125.5 (benzoxazole*C*(5)H), 126.4 (thiophene*C*(5)H), 127.2 (thiophene*C*(4)H), 128.2 (2 × C(6)Ph*C*(2)H), 129.1 (2 × C(6)Ph*C*(3)H), 130.5 (C(6)Ph*C*(4)H), 132.6 (C(6)Ph*C*(1)), 139.9 (thiophene*C*(3)), 141.6 (benzoxazole*C*(4a)), 150.3 (benzoxazole*C*(7a)), 155.7 (*C*(6)), 161.1 (benzoxazole*C*(2)), 166.5 (*C*(2)O); *m/z* ($APCI^+$) 374 ($[M+H]^+$, 100%); HRMS ($APCI^+$) $C_{22}H_{16}O_3NS$ ($[M+H]^+$) requires 374.0845, found 374.0846 (+0.2 ppm).

Ethyl (*S*)-4-(dimethylcarbamoyl)-1-oxo-2,3-dihydro-1*H*-benzo[4,5]thiazolo[3,2-a]pyridine-3-carboxylate (29A)

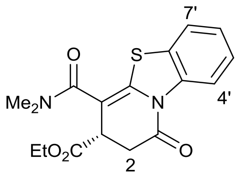

The title compound was prepared according to *General Procedure D* from *(E)*-4-ethoxy-4-oxobut-2-enoic anhydride (270 mg, 1.00 mmol), and 2-(1,3-benzothiazol-2-yl)-*N*,*N*-dimethylacetamide (158 mg, 0.72 mmol), EtN(*i*Pr)$_2$ (140 μL, 0.80 mmol) and HyperBTM (11.1 mg, 0.036 mmol) in THF (2 mL) and purified by chromatography on silica gel (10% EtOAc/CH_2Cl_2) to give **29A** as a yellow oil (191 mg, 77%); $[\alpha]_D^{20}$ –81.6 (*c* 1.0 in CH_2Cl_2); chiral HPLC analysis, ChiralPak AD-H (20% *i*-PrOH:hexane, flow rate 1.0 mL min^{-1}, 211 nm, 30 °C), t_R major: 17.5 min, t_R minor: 31.2 min, 92% ee; ν_{max} (film)/cm^{-1} 2928 (C-H), 1724 (C=O), 1709 (C=O), 1620 (C=O); δ_H (500 MHz, $CDCl_3$) 1.24 (3H, t, *J* 7.2, CH_2CH_3), 3.02 (1H, dd, *J* 16.5, 7.3, C(2)*H*H), 3.08 (6H, s, N(C*H*$_3$)$_2$), 3.10 (1H, dd, *J* 11.3, 5.2, C(2)*H*H), 3.91 (1H, dd, *J* 7.3, 5.1, C(3)*H*), 4.06-4.22 (2H, m, C*H*$_2$CH$_3$), 7.14 (1H, td, *J* 7.6, 1.2, C(5')*H*), 7.19-7.27 (2H, m, Ar*H*), 8.34 (1H, dd, *J* 8.3, 1.1, C(4')*H*); δ_C (126 MHz, $CDCl_3$) 14.1 (*C*(3)H), 34.6 (*C*(2)H$_2$), 37.0 (2 × N(*C*H$_3$)$_2$, 40.1 (CH$_2$*C*H$_3$), 61.7, *C*H$_2$CH$_3$), 100.5 (*C*(5)), 117.6 (*C*(4')H), 121.1 (*C*(7')H), 124.8 (*C*(7a')), 125.3 (*C*(6')H), 126.4 (*C*(5')H), 137.2 (*C*(4a')), 141.4 (*C*(5)), 166.7 (*C*(1)O), 169.2 (*C*ONMe$_2$), 171.4 (*C*O$_2$Et); *m/z* (NSI$^+$) 347 ([M+H]$^+$, 100%), plus unknown degradation peaks; HRMS (NSI$^+$) $C_{17}H_{19}O_4N_2S$ ([M+H]$^+$) requires 347.1060, found 347.1062 (+0.6 ppm).

Ethyl (*S*)-4-benzoyl-1-oxo-2,3-dihydro-1*H*-benzo[4,5]thiazolo[3,2-*a*]pyridine-3-carboxylate (30A) and ethyl (*S*)-5-(benzo[*d*]thiazol-2-yl)-2-oxo-6-phenyl-3,4-dihydro-2*H*-pyran-4-carboxylate (30B)

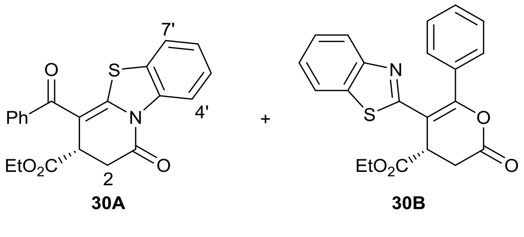

The title compounds were prepared according to *General Procedure D* from *(E)*-4-ethoxy-4-oxobut-2-enoic anhydride (297 mg, 1.00 mmol) and 2-phenacyl benzothiazole (182 mg, 0.72 mmol), EtN(*i*Pr)$_2$ (140 μL, 0.80 mmol) and HyperBTM (11.1 mg, 0.036 mmol) in THF (2

mL) and purified by chromatography on silica gel (10:2 CH_2Cl_2/hexane→CH_2Cl_2) to give **30A** as a yellow oil (136 mg, 50%) and **30B** as a yellow oil (13 mg, 5%).

30A (major): $[\alpha]_D^{20}$ –20.1 (*c* 1.0, CH_2Cl_2); chiral HPLC analysis, ChiralPak AD-H (20% *i*-PrOH:hexane, flow rate 1 mL min^{-1}, 211 nm, 30 °C), t_R major: 27.6 min, t_R minor: 23.9 min, 52% ee; ν_{max} (film)/cm^{-1} 2982 (C-H), 2249 (C-H), 1724 (C=O), 1609 (C=O); δ_H (300 MHz, $CDCl_3$) 1.13 (3H, t, *J* 7.1, CH_2CH_3), 3.05 (1H, dd, *J* 16.4, 6.5, C(2)*H*H), 3.19 (1H, dd, *J* 16.4, 2.6, C(2)*H*H), 4.00-4.10 (3H, m, CH_2CH_3, C(3)*H*), 7.29-7.35 (1H, m, C(6')*H*), 7.35-7.42 (1H, m, C(6')*H*), 7.42-7.60 (6H, m, AriH), 8.34-8.72 (1H, m, C(4')*H*); δ_C (75 MHz, $CDCl_3$) 14.0 (CH_2CH_3), 35.3 (*C*(2)H_2), 39.1 (*C*(3)H), 61.8 (CH_2CH_3), 102.8 (*C*(4)), 117.8 (*C*(4')H), 121.9 (*C*(7')H), 126.0 (*C*(6')H), 127.0 (2 × C(4)COPh*C*(2)H), 127.2 (*C*(5')H), 127.4 (*C*(7a')), 128.5 (2 × C(4)COPh*C*(3)H), 130.2 (C(4)COPh*C*(4)H), 136.1 (*C*(4a')), 139.8 (C(4)COPh*C*(1)), 157.3 (*C*(5)), 167.3 (*C*(1)O), 171.6 (*C*OOEt), 191.1 (C(4)*C*OPh); *m/z* (NSI^+) 380 ($[M+H]^+$, 70%), plus unknown degradation peaks; HRMS (NSI^+) $C_{21}H_{18}O_4NS$ ($[M+H]^+$) requires 380.0951, found 380.0951 (−0.0 ppm).

30B (minor): $[\alpha]_D^{20}$ –2.0 (*c* 0.25, CH_2Cl_2); chiral HPLC analysis, ChiralPak AD-H (20% *i*-PrOH:hexane, flow rate 1 mL min^{-1}, 211 nm, 30 °C), t_R major: 22.7 min, t_R minor: 11.1 min, 56% ee; ν_{max} (film)/cm^{-1} 2982 (C-H), 2361 (C-H), 1782 (C=O), 1728 (C=O), 1651 (C=O); δ_H (400 MHz, $CDCl_3$) 1.15 (3H, t, *J* 7.1, CH_2CH_3), 3.05 (1H, dd, *J* 16.3, 7.4, C(3)*H*H), 3.27 (1H, dd, *J* 16.3, 2.1, C(3)*H*H), 4.06 – 4.22 (2H, m, CH_2CH_3), 4.68 (1H, dd, *J* 7.4, 2.1, benzothiazoleC(6)*H*), 7.32 (1H, ddd, *J* 8.2, 7.2, 1.2, benzothiazoleC(5)*H*), 7.37-7.54 (6H, m, Ar*H*), 7.65 (1H, ddd, *J* 8.0, 1.3, 0.7, benzothiazoleC(7)*H*), 7.97 (1H, ddd, *J* 8.2, 1.2, 0.6, benzothiazoleC(4)*H*); δ_C (126 MHz, $CDCl_3$) 14.1 (CH_2CH_3), 31.3 (*C*(3)H_2), 41.5 (*C*(4)H), 62.0 (CH_2CH_3), 110.9 (*C*(5)), 121.4 (benzothiazole*C*(4)H), 123.2 (benzothiazole*C*(7)H), 125.6 (benzothiazole*C*(6)H), 126.2 (benzothiazole*C*(5)H), 129.0 (2 × C(6)Ph*C*(2)H), 130.1 (2 × C(6)Ph*C*(3)H), 131.1 (C(6)Ph*C*(4)H), 131.7 (C(6)Ph*C*(1)), 135.8 (benzothiazole*C*(7a), 152.5 (benzothiazole*C*(4a), 155.4 (*C*(6)), 163.9 (benzothiazole*C*(2)), 165.6 (*C*(2)O), 170.7 (*C*OOEt); *m/z* (NSI^+) 380 ($[M+H]^+$, 15%), plus unknown degradation peaks; HRMS (NSI^+) $C_{21}H_{18}O_4NS$ ($[M+H]^+$) requires 380.0951, found 380.0950 (−0.3 ppm).

Ethyl *(S)*-5-(benzo[d]oxazol-2-yl)-2-oxo-6-phenyl-3,4-dihydro-2H-pyran-4-carboxylate (31B) and ethyl *(S)*-4-benzoyl-1-oxo-2,3-dihydro-1H-benzo[4,5]oxazolo[3,2-a]pyridine-3-carboxylate (31A)

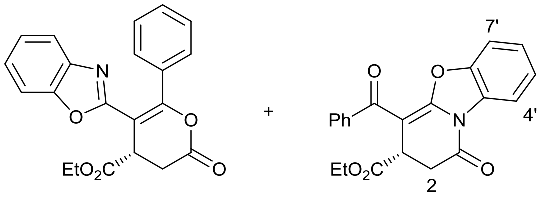

31B **31A**

The title compounds were prepared according to a modification of *General Procedure D* from *(E)*-4-ethoxy-4-oxobut-2-enoic anhydride (270 mg, 1.0 mmol), and 2-phenacylbenzoxazole (171 mg, 0.72 mmol), EtN(*i*Pr)$_2$ (140 µL, 0.80 mmol) and HyperBTM (11.1 mg, 0.036 mmol) in THF (2 mL). The reaction was conducted in anhydrous tetrahydrofuran under a nitrogen atmosphere, and the reaction temperature was maintained at 0 C for 5, followed by the stated work-up procedure. The crude product (**31B**:**31A**, 97:3 by ^{1}H NMR spectroscopic analysis) was purified by column chromography (hexane:EtOAc, 100:0→65:35) to give **31B/31A** as an off-white solid (96:4 mixture of constitutional isomers, 177 mg, 68%); mp 84-93 °C; $[\alpha]_D^{20}$ +43.8 (*c* 1.0, CHCl$_3$); ν_{max} (film)/cm^{-1} 2986 (C-H), 1790 (C=O), 1724 (C=O), 1647 (C=O); *m/z* (NSI$^+$) 386 ([M+Na]$^+$, 30%), plus unknown degradation peaks; HRMS (NSI$^+$) $C_{21}H_{17}O_5NNa$ ([M+Na]$^+$) requires 389.0999, found 389.0995 (–1.0 ppm).

31B (major): chiral HPLC analysis, ChiralPak IC (30% *i*-PrOH:hexane, flow rate 1.0 mL min^{-1}, 254 nm, 30 °C), t_R major: 10.2 min, t_R minor: 12.8 min, 94% ee; δ_H (500 MHz, CDCl$_3$) 1.12 (3H, t, *J* 7.1, CH$_2$*CH$_3$*), 3.00 (1H, dd, *J* 16.3, 7.3, C(3)*H*H), 3.28 (1H, dd, *J* 16.2, 2.0, C(3)*H*H), 4.09-4.21 (2H, m, *CH$_2$*CH$_3$), 4.48 (1H, dd, *J* 7.3, 2.0, C(4)*H*), 7.22-7.40 (5H, m, Ar*H*), 7.42-7.49 (3H, m, ArH), 7.67-7.72 (1H, m, benzoxazoleC(4)*H*); δ_C (101 MHz, CDCl$_3$) 13.9 (CH$_2$*C*H$_3$), 30.7 (*C*(3)H$_2$), 40.6 (*C*(4)H), 62.1 (*C*H$_2$CH$_3$), 103.0 (*C*(5)), 110.5 (benzoxazole*C*(7)H), 119.9 (benzoxazole*C*(4)H), 124.7 (benzoxazole*C*(6)H), 125.5 (benzoxazole*C*(4)H), 128.2 (2 × C(6)Ph*C*(2)H), 129.0 (2 × C(6)Ph*C*(3)H), 130.6 (C(6)Ph*C*(4)H), 132.3 (C(6)Ph*C*(1)), 141.4 (benzoxazole*C*(4a)), 150.2 (benzoxazole*C*(7a)), 156.8 (*C*(6)), 160.7 (benzoxazole*C*(2)), 165.1 (*C*(2)O), 170.3 (*C*OOEt).

31A (minor): chiral HPLC analysis, ChiralPak IC (30% *i*-PrOH:hexane, flow rate 1.0 mL min^{-1}, 254 nm, 30 °C), t_R major: 32.3 min, t_R minor: 23.7 min, 90% ee; δ_H (500 MHz, CDCl$_3$, characteristic peaks) 2.99 (1H, dd, *J* 17.0, 7.6, C(3)*H*H), 3.21 (H, dd, *J* 17.1, 2.8, C(3)*H*H),

7.96-8.00 (1H, m, C(4')*H*); δ_H (400 MHz, $CDCl_3$, characteristic peaks) (1H, dd, *J* 17.1, 7.6, C(2)*H*H), 3.19 (1H, dd, *J* 17.1, 2.8, C(2)*H*H), 7.95-7.99 (1H, m, C(4')*H*); δ_C (101 MHz, $CDCl_3$, characteristic peaks) 33.4 (*C*(2)H_2), 41.9 (*C*(3)H), 62.1 (*C*H_2CH_3), 133.8 (C(4)COPh*C*(4)H), 135.7 (*C*(4a')), 140.7 (C(4)COPh*C*(1)), 150.9 (*C*(5)), 161.2 (*C*(1)O), 193.2 (C(4)*C*OPh).

4-Benzoyl-3,3-dimethyl-2,3-dihydro-1H-benzo[4,5]thiazolo[3,2-a]pyridin-1-one (34)

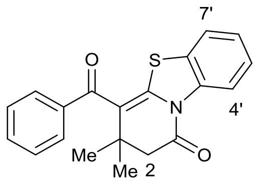

The title compound was prepared according to *General Procedure D* from 3-methylbut-2-enoic anhydride (91 mg, 0.50 mmol), and 2-phenacylbenzothiazole (91 mg, 0.36 mmol), EtN(*i*Pr)$_2$ (70 μL, 0.40 mmol) and HyperBTM (3.4 mg, 0.018 mmol) in THF (1 mL) and purified by chromatography on silica gel (10% EtOAc/petroleum ether) to give **22** as a yellow solid (75 mg, 62%); mp 110-113 °C; ν_{max} (film)/cm^{-1} 2928 (C-H), 2870 (C-H), 1719 (C=O), 1593 (C=O); δ_H (300 MHz, $CDCl_3$) 1.26 (6H, s, C(3)(C*H*$_3$)$_2$), 2.70 (2H, s C(2)*H*$_2$), 7.14-7.32 (3H, m, Ar*H*), 7.41-7.57 (3H, m, Ar*H*), 7.59-7.66 (2H, m, 2 × C(4)COPhC(2)*H*), 8.43-8.50 (1H, m, C(4')*H*); δ_C (126 MHz, $CDCl_3$) 26.7 (2 × C(3)(*C*H$_3$)$_2$), 35.1 (*C*(3)(CH$_3$)$_2$), 49.4 (*C*(2)H_2), 117.3 (*C*(4)), 117.6 (*C*(4')H), 121.3 (*C*(7')H), 125.7 (*C*(6')H), 126.7 (*C*(5')H), 126.9 (*C*(7a')), 128.1 (2 × *C*(4)COPh*C*(2)H), 128.9 (2 × *C*(4)COPh*C*(3)H), 131.6 (*C*(4)COPh*C*(4)H), 136.4 (*C*(4a')), 140.7 (*C*(4)COPh*C*(1)), 150.4 (*C*(5)), 168.9 (*C*1)O), 194.4 (*C*(4)*C*OPh); *m/z* (NSI$^+$) 336 ([M+H]$^+$, 30%), 374 ([M+K]$^+$, 100%); HRMS (NSI$^+$) $C_{20}H_{18}O_2NS$ ([M+H]$^+$) requires 336.1053, found 336.1048 (–1.4 ppm).

(*R*)-4-Benzoyl-3-methyl-3-(trifluoromethyl)-2,3-dihydro-1H-benzo[4,5]thiazolo[3,2-a]pyridin-1-one (35) and *(E)*-2-(benzo[d]thiazol-2-yl)-6,6,6-trifluoro-5-methyl-1-phenylhex-4-ene-1,3-dione (36)

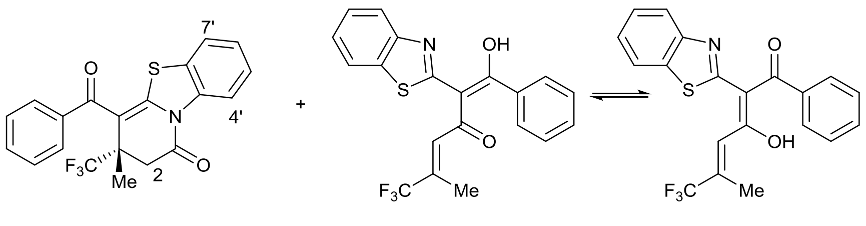

1,4-addition product **35** 1,2-addition product **36**

The title compounds were prepared according to *General Procedure D* from (2*E*)-4,4,4-trifluoro-3-methylbut-2-enoic anhydride (406 mg, 1.40 mmol) and 2-phenacyl benzothiazole (253 mg, 1.00 mmol), EtN(*i*Pr)$_2$ (190 µL, 1.10 mmol) and HyperBTM (15.4 mg, 0.05 mmol) in THF (2.5 mL) and purified by chromatography on silica gel (10:2 CH_2Cl_2/hexane) to give **35**/**36** as a yellow solid (75:25 mixture of isomers, 163 mg, 42%). The mixture was separated by Et_2O trituration to give **36** as a yellow solid (50 mg, 13%) and **35** as a yellow oil which was solidified by hexane trituration (110 mg, 28%).

35 (1,4-addition): mp 145-150 °C; $[\alpha]_D^{20}$ –18.0 (*c* 0.5 in CH_2Cl_2); chiral HPLC analysis, ChiralPak AD-H (10% *i*-PrOH:hexane, flow rate 1 mL min^{-1}, 211 nm, 30 °C), t_R major: 18.1 min, t_R minor: 16.4 min, 96% ee; ν_{max} (film)/cm^{-1} 3019 (C-H), 1721 (C=O), 1638 (C=O); δ_H (500 MHz, $CDCl_3$) 1.35 (3H, s, CH_3), 2.96 (1H, d, *J* 16.8, C(2)*H*H), 3.12 (1H, d, *J* 16.8, C(2)*H*H), 7.20-7.25 (1H, m, C(5')*H*), 7.29-7.35 (2H, m, Ar*H*), 7.43-7.49 (2H, m, 2 × C(4)COPhC(3)*H*), 7.49-7.55 (1H, m, C(7')*H*), 7.57-7.63 (2H, m, 2 × C(4)COPhC(2)*H*), 8.42 – 8.49 (1H, m, C(4')*H*); δ_C (126 MHz, $CDCl_3$) 21.1 (*C*H$_3$), 41.8 (*C*(2)H$_2$), 43.8 (q, $^2J_{CF}$ 26.9, *C*(3)CF$_3$), 105.3 (*C*(4)), 117.8 (*C*(4')H), 121.4 (*C*(7')H), 126.1 (*C*(6')H), 126.4 (*C*(7a'')), 127.2 (*C*(5')H), 127.6 (q, $^1J_{CF}$ 286.1, *C*F$_3$), 128.1 (2 × C(4)COPh*C*(2)H), 128.8 (2 × C(4)COPh*C*(3)H), 131.4 (C(4)COPh*C*(4)H), 136.0 (*C*(4a')), 140.0 (C(4)COPh*C*(1)), 156.0 (*C*(5)), 166.1 (*C*(1)O), 193.0 (C(4)*C*OPh); δ_F (470 MHz, $CDCl_3$) –75.64; *m/z* (NSI$^+$) 390 ([M+H]$^+$, 100%); HRMS (NSI$^+$) $C_{20}H_{15}F_3O_2NS$ ([M+H]$^+$) requires 390.0770, found 390.0766 (–1.1 ppm).

36 (1,2-addition): mp 196-198 °C; ν_{max} (film)/cm^{-1} 3023 (C-H), 1537 (C=O?), 1499 (C=O?), 1450 (C=N?) *highly conjugated system, carbonyl peaks very low?*; δ_H (500 MHz, $CDCl_3$) 1.98 (6H, s, br, CH_3), 6.08 (1H, dt, *J* 3.0, 1.6, *1-keto*-C(4)*H*), 6.18 (1H, dt, *J* 3.1, 1.7, *1-enol*-C(4)*H*), 7.37-7.44 (6H, m, Ar*H*), 7.47-7.56 (8H, m, Ar*H*), 7.63 (2H, t, *J* 8.0,

benzothiazoleC(7)*H*), 7.85 (2H, d, *J* 7.9, benzothiazoleC(4)*H*), 14.72 (1H, s, *1-keto*-O*H*), 14.98 (1H, s, *1-enol*-O*H*); δ_C (126 MHz, $CDCl_3$) 12.4 (CH_3), 12.4 (CH_3), 108.9 (*C*(2)), 109.1 (*C*(2)), 114.2 (benzothiazole*C*(7)H), 114.4 (benzothiazole*C*(7)H), 122.5 (benzothiazole*C*(4)H), 123.4 (q, $^1J_{CF}$ 274.0, CF_3), 123.5 (q, $^1J_{CF}$ 274.0, CF_3), 125.2 (benzothiazole*C*(5)H), 125.2 (benzothiazole*C*(5)H), 127.7 (benzothiazole*C*(6)H), 127.8 (benzothiazole*C*(6)H), 128.2 (2 × C(1)Ph*C*(2)H), 128.2 (2 × C(1)Ph*C*(2)H), 128.5 (2 × C(1)Ph*C*(3)H), 128.5 (2 × C(1)Ph*C*(3)H), 128.9 (q, $^2J_{CF}$ 21.5, *C*(5)CF_3), 130.4 (q, $^3J_{CF}$ 5.7, *1-keto-C*(4)H), 130.9 (q, $^3J_{CF}$ 5.7, *1-enol-C*(4)H), 131.6 (C(1)Ph*C*(4)H), 131.7 (C(1)Ph*C*(4)H), 132.0 (C(1)Ph*C*(1)), 132.1 (C(1)Ph*C*(1)), 137.9 (benzothiazole*C*(4a)), 138.1 (benzothiazole*C*(4a)), 141.7 (benzothiazole*C*(7a)), 142.0 (benzothiazole*C*(7a)), 168.8 (benzothiazole*C*(2)), 169.2 (benzothiazole*C*(2)), 187.3 (*1-keto-C*(3)OH), 187.6 (*1-enol-C*(3)O), 193.2 (*1-enol-C*(1)OH), 194.1 (*1-keto-C*(1)O); δ_F (470 MHz, $CDCl_3$) –70.96, –70.95; *m/z* (NSI^+) 390 ($[M+H]^+$, 100%); HRMS (NSI^+) $C_{20}H_{15}F_3O_2NS$ ($[M+H]^+$) requires 390.0770, found 390.0763 (–1.8 ppm).

(*R*)-3-(4'-Methoxy-[1,1'-biphenyl]-3-yl)-*N*,*N*-dimethyl-1-oxo-2,3-dihydro-1*H*-benzo[4,5]thiazolo[3,2-*a*]pyridine-4-carboxamide (37)

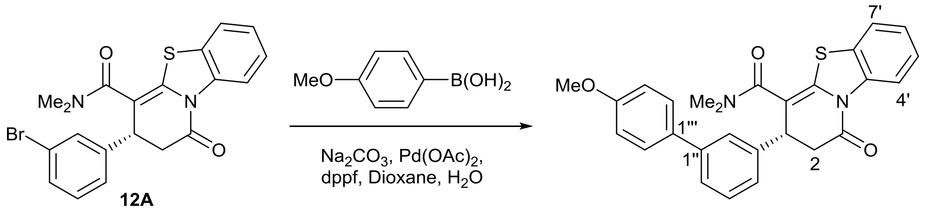

(*R*)-3-(3-Bromophenyl)-*N*,*N*-dimethyl-1-oxo-2,3-dihydro-1*H*-benzo[4,5]thiazolo[3,2-*a*]pyridine-4-carboxamide **12A** (128 mg, 0.30 mmol), 4-methoxyboronic acid (68 mg, 0.45 mmol), sodium carbonate (64 mg, 0.60 mmol), $Pd(OAc)_2$ (3.4 mg, 0.015 mmol) and diphenylphosphinoferrocene (dppf, 16.6 mg, 0.03 mmol) were added to degassed dioxane/water (9:1, 3 mL) under N_2 and heated in a sealed tube at 100 °C for 15 h. The reaction was then cooled to room temperature and flushed through a plug of silica with EtOAc, the collected solution was concentrated *in vacuo* and then purified by chromatography on silica gel (CH_2Cl_2→10% EtOAC/CH_2Cl_2) to give **37** as a colourless solid (103 mg, 70%); mp 75-76 °C; $[\alpha]_D^{20}$ –78.7 (*c* 1.0, CH_2Cl_2); chiral HPLC analysis, ChiralPak AS-H (20% *i*-PrOH:hexane, flow rate 1 mL min^{-1}, 220 nm, 30 °C), t_R major: 13.0 min, t_R minor: 26.8 min, >99% ee; ν_{max} (film)/cm^{-1} 2930 (C-H), 2836 (C-H), 1701 (C=O), 1608 (C=O), 1585 (C=N), 1481 (C=C); δ_H (400 MHz, $CDCl_3$) 2.86 (6H, s, N(CH_3)$_2$), 3.02 (1H, dd, *J* 16.2, 6.4, C(2)*H*H), 3.20 (1H, dd, *J* 16.2, 7.1, C(2)*H*H), 3.83 (3H, s, OCH_3), 4.26 (1H, at, *J*

6.7, C(3)*H*), 6.90-7.00 (2H, m, 2 × biphenylC(3''')*H*), 7.12-7.36 (5H, m, Ar*H*), 7.39-7.45 (2H, m, Ar*H*), 7.45-7.50 (2H, m, 2 × biphenylC(2''')*H*), 8.34-8.39 (1H, m, C(4')*H*); δ_C (101 MHz, $CDCl_3$) 37.0 (2 × N(CH_3)$_2$), 40.4 (*C*(3)H), 40.9 (*C*(4)H_2), 55.4 (OCH_3), 106.4 (*C*(4)), 114.3 (2 × biphenyl*C*(3''')H), 117.5 (*C*(4')H), 121.3 (*C*(7')H), 125.0 (Ar*C*H), 125.3 (Ar*C*H), 125.4 (Ar*C*H), 125.9 (Ar*C*H), 126.0 (*C*(7a')), 126.3 (Ar*C*H), 128.2 (2 × biphenylC(2''')H), 129.5 (Ar*C*H), 133.2 (biphenyl*C*(1''')), 137.2, (*C*(4a')), 141.4 (Ar*C*), 141.6 (Ar*C*), 142.3 (Ar*C*), 159.4 (*C*(5)), 167.7 (*C*(1)O), 169.3 (*C*ONMe$_2$); *m/z* (NSI^+) 457 ($[M+H]^+$, 100%), 479 ($[M+Na]^+$, 55%), 495 ($[M+K]^+$, 50%), 935 ($2M+Na]^+$, 55%), 951 ($2M+K]^+$, 50%); HRMS (NSI+) C27H25O3N2S ([M+H]+) requires 457.1580, found 457.1577 (–0.7 ppm).

Methyl (*R*,*E*)-3-(3-(4-(dimethylcarbamoyl)-1-oxo-2,3-dihydro-1H-benzo[4,5]thiazolo[3,2-*a*]pyridin-3-yl)phenyl)acrylate (38)

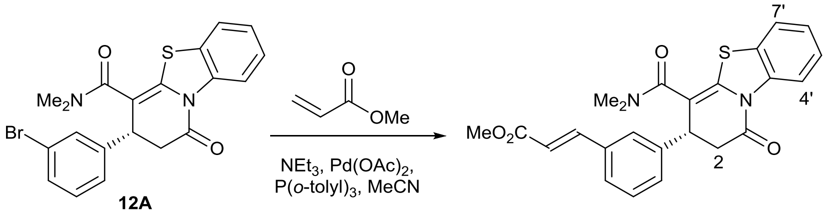

(*R*)-3-(3-Bromophenyl)-*N*,*N*-dimethyl-1-oxo-2,3-dihydro-1*H*-benzo[4,5]thiazolo[3,2-*a*]pyridine-4-carboxamide **12A** (128 mg, 0.30 mmol), methyl acrylate (34 μL, 0.38 mmol), triethylamine (52 μL, 0.38 mmol), $Pd(OAc)_2$ (3.4 mg, 0.015 mmol) and P(*o*-tolyl)$_3$ (9.1 mg, 0.03 mmol) were added to degassed DMF (2 mL) under N_2 and heated in a sealed tube at 125 °C for 15 h. The reaction was then cooled to room temperature and flushed through a plug of silica with EtOAc, the collected solution was concentrated *in vacuo* and redissolved in Et_2O (20 mL). This solution was washed with brine (4 × 20 mL), dried over $MgSO_4$, filtered and concentrated *in vacuo.* The crude oil obtained was then purified by chromatography on silica gel (10% EtOAc/CH_2Cl_2→20% EtOAc/CH_2Cl_2) to give **38** as a colourless solid (115 mg, 89%) plus recovered starting material (14 mg, 11%); mp 42-44 °C $[\alpha]_D^{20}$ –81.6 (*c* 1.0, CH_2Cl_2); chiral HPLC analysis, ChiralPak IC (40% *i*-PrOH:hexane, flow rate 1 mL min^{-1}, 211 nm, 30 °C), t_R major: 60.2 min, t_R minor: 47.4 min, 97% ee; ν_{max} (film)/cm^{-1} 3007 (C-H), 2947 (C-H), 1701 (C=O), 1616 (br, 2 × C=O), 1581 (C=N), 1487, (C=C); δ_H (400 MHz, $CDCl_3$) 2.86 (6H, s, N(CH_3)$_2$), 2.96 (1H, dd, *J* 16.3, 6.1, C(2)*H*H), 3.18 (1H, dd, *J* 16.3, 7.1, C(2)*H*H), 3.78 (3H, s, OCH_3), 4.22 (1H, at, *J* 6.6, C(3)*H*), 6.39 (1H, d, *J* 16.0, ArCH=C*H*CO_2Me), 7.09-7.36 (5H, m, Ar*H*), 7.40-7.44 (1H, m, C(7')*H*), 7.62 (1H, d, *J* 16.0, ArC*H*=CHCO_2Me), 8.33-8.36 (1H, m, C(4')*H*); δ_C (126 MHz, $CDCl_3$) 36.9, 40.1, 40.7, 51.8, 105.6, 117.6, 118.4, 121.3, 125.5, 125.8, 126.4, 126.8, 126.9, 128.7, 129.8, 135.2, 137.1,

141.8, 142.5, 144.4, 167.3, 167.4, 169.1; *m/z* (NSI^+) 435 ($[M+H]^+$, 100%), 457 ($[M+Na]^+$, 60%), 891 ($2M+Na]^+$, 30%); HRMS (NSI^+) $C_{24}H_{23}O_4N_2S$ ($[M+H]^+$) requires 435.1373, found 4355.1369 (–0.9 ppm).

Isomerisation and Epimerisation Experiments

Isomerisation experiments using (4*R*)-5-(1,3-benzothiazol-2-yl)-4,6-diphenyl-3,4-dihydro-2H-pyran-2-one 4B

The potential isomerisation of (4*R*)-5-(1,3-benzothiazol-2-yl)-4,6-diphenyl-3,4-dihydro-2H-pyran-2-one **4B** (9 mg, 0.025 mmol, 89% ee) was investigated under a range of conditions. After each experiment, the solvent was removed under reduced pressure and the residue dissolved in $CDCl_3$ for analysis using ^{1}H NMR spectroscopy. The $CDCl_3$ was then removed under reduced pressure and the residue used for the next isomerisation experiment. The following experiments were performed in the order given:

A: stirred in anhydrous THF (0.15 mL) at room temperature for 3 hours under a nitrogen atmosphere.

B: stirred with Hünig's base (5 μL, 0.029 mmol, 1.15 equiv.) in anhydrous THF (0.15 mL) at room temperature for 3 hours under a nitrogen atmosphere.

C: stirred with HyperBTM **1** (0.15 mL, 0.0033 M in THF, 0.0005 mmol, 2 mol%) and Hünig's base (5 μL, 0.029 mmol, 1.15 equiv.) at room temperature for 3 hours under a nitrogen atmosphere.

D: stirred with 2-phenacylbenzothiazole **S18** (19 mg, 0.075 mmol, 2.5 equiv.), HyperBTM **1** (0.0005 mmol, 2 mol%) and Hünig's base (5 μL, 0.029 mmol, 1.15 equiv.) in anhydrous THF (0.15 mL) at room temperature for 3 hours under a nitrogen atmosphere.

E: stirred with (*E*)-cinnamic anhydride **S7** (21 mg, 0.075 mmol, 2.5 equiv.), 2-phenacylbenzothiazole **S18** (19 mg, 0.075 mmol, 2.5 equiv.), HyperBTM **1** (0.0005 mmol, 2 mol%) and Hünig's base (5 μL, 0.029 mmol, 1.15 equiv.) in anhydrous THF (0.15 mL) at room temperature for 16 hours under a nitrogen atmosphere.

Selected regions of the NMR spectra for each of the experiments along with the spectra of the purified lactam **4A** and lactone **4B** are given below. The formation of lactam **4A** was only observed in experiment **E** (full experimental conditions). In this case the ratio of lactam:lactone after 16 hours was 2.3:1. This reflects the ratio of (*E*)-cinnamic anhydride **S7**,

2-phenacylbenzothiazole **S18** and lactone **4B** used in the reaction, and not the ratio observed under usual reaction conditions (88:12).

These results suggest that isomerisation of lactone **4B** to give lactam **4A** does not occur under the reaction conditions.

Figure S1. NMR spectra of lactam **4A**, lactone **4B** and isomerisation experiments **A-E** (CH_2 region)

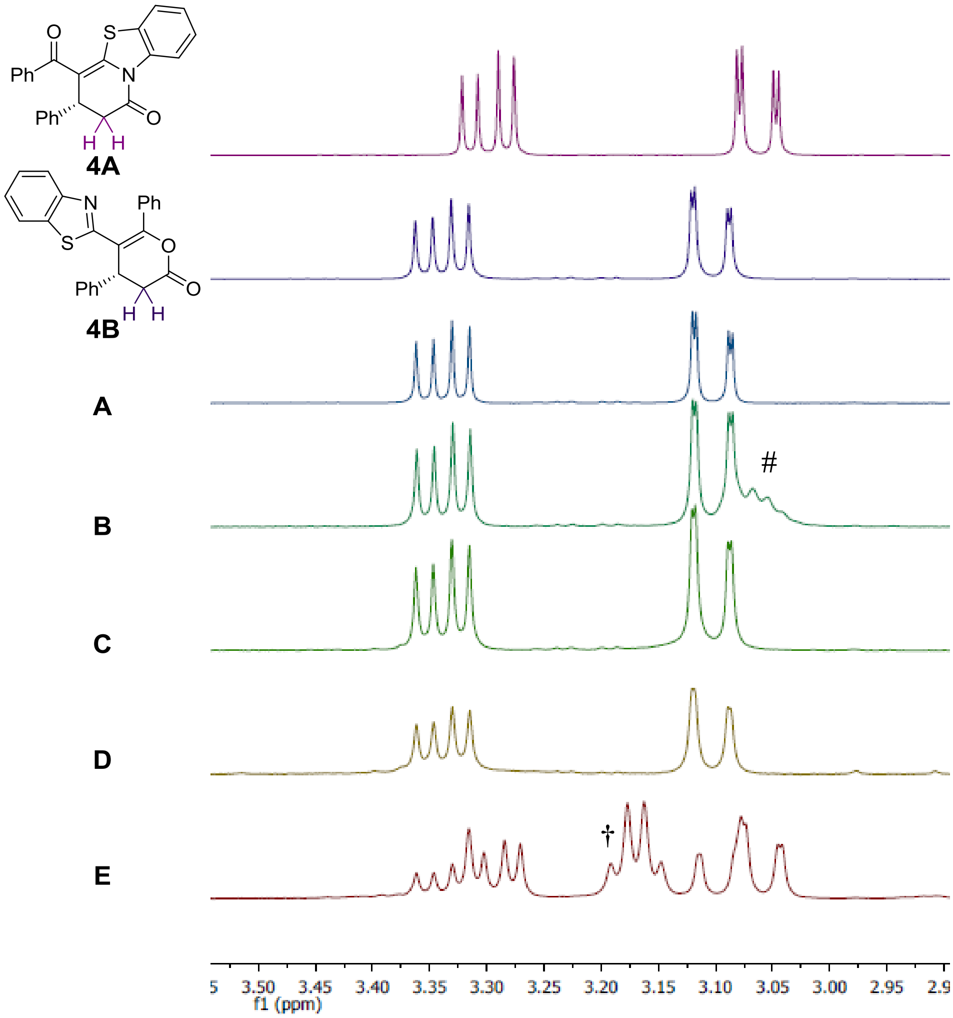

# = ^{i}Pr$_2$NEt; † = [^{i}Pr$_2$NHEt]$^+$

Figure S2. NMR spectra of lactam **4A**, lactone **4B** and isomerisation experiments **A-E** (CH region)

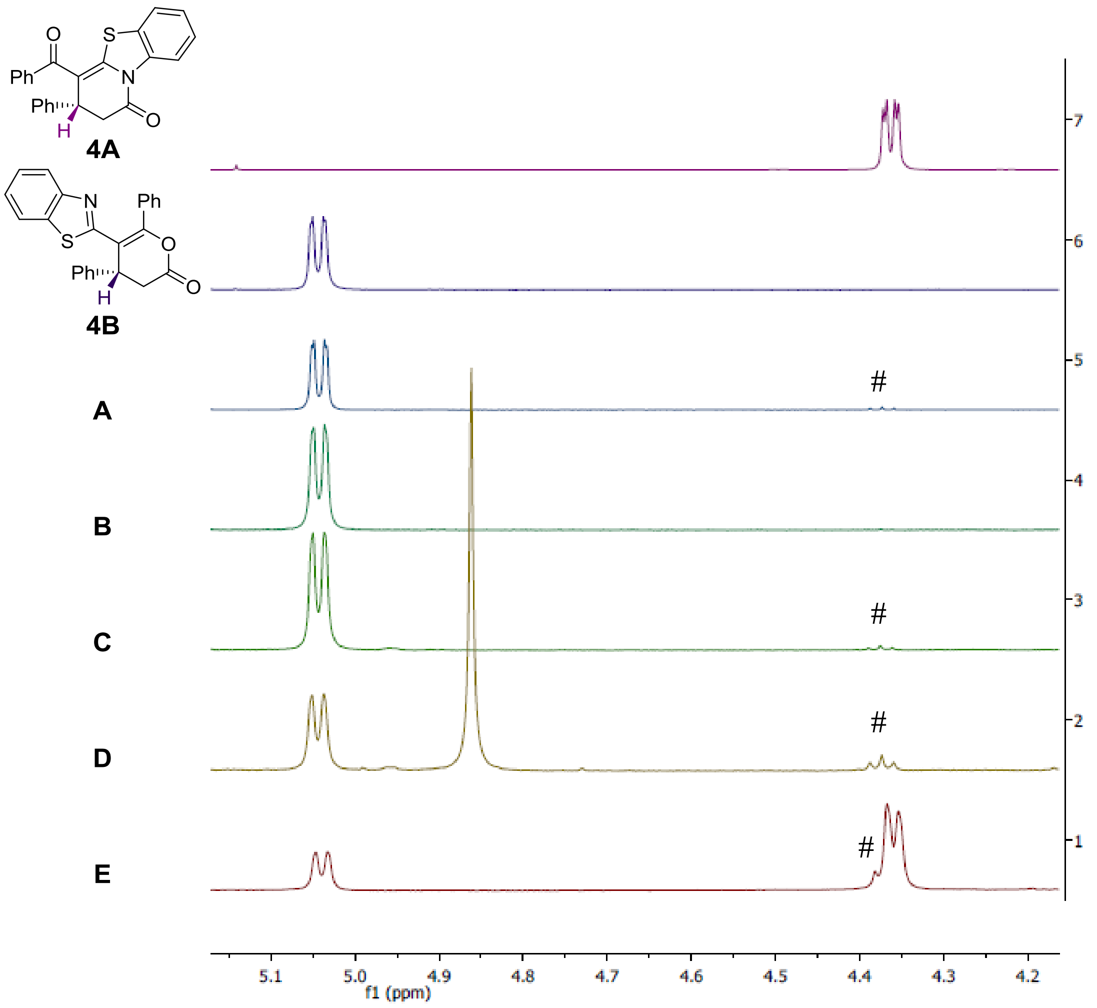

# = γ-Butyrolactone impurity from the anhydrous THF

Figure S3. NMR spectra of lactam **4A**, lactone **4B** and isomerisation experiments **A-E** (aromatic region)

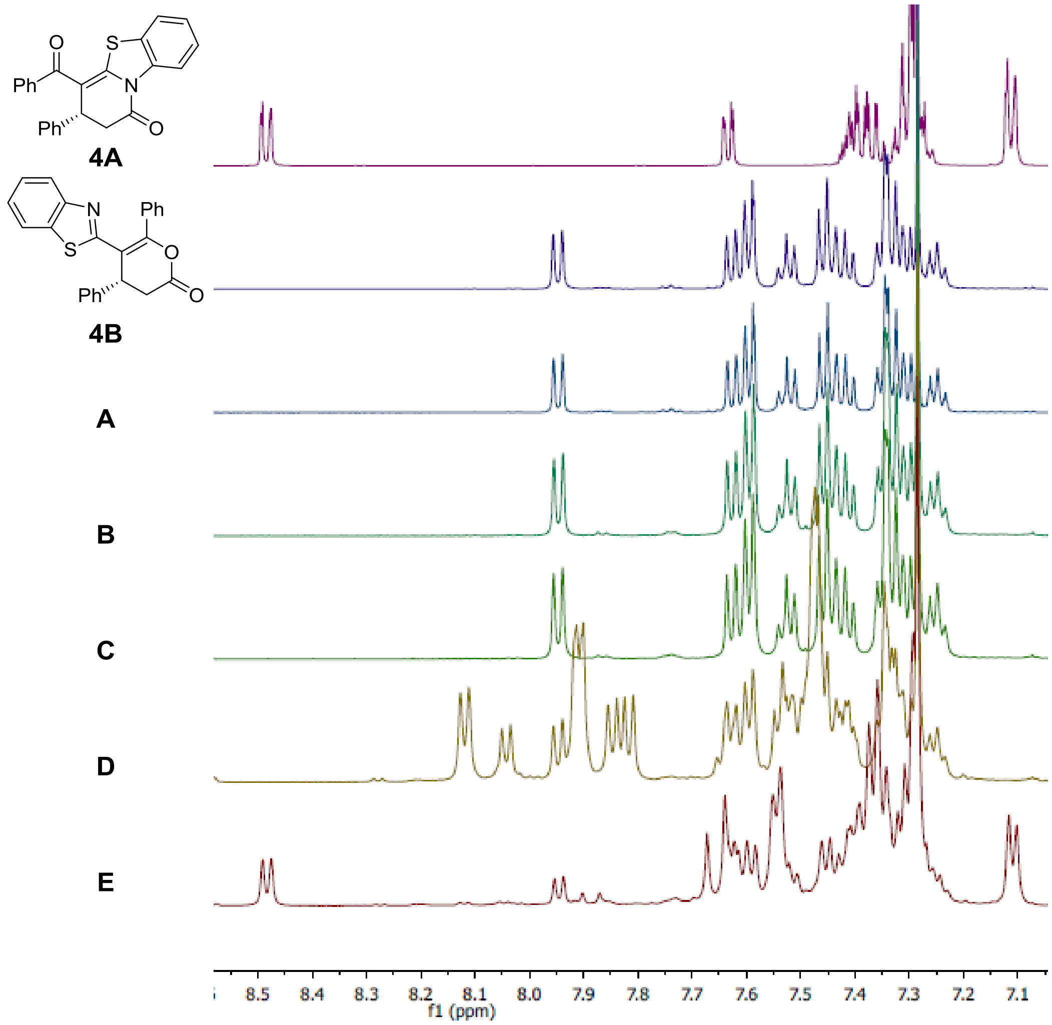

Studies on the reaction using (*E*)-2-bromocinnamic anhydride, and possible epimerisation of lactone product 16B

Based on the low %ee obtained for lactone **16B** (31% ee, vs. 81% ee for lactam **16A**), further studies were undertaken to investigate the origin of this anomalous result.

The standard reaction protocol was performed in the **absence of HyperBTM**. ~3% Lactam **16A** and no lactone **16B** was obtained after 16 h, based on quantitative ^{1}H NMR spectroscopic analysis of the crude reaction products, using 1,3,5-trimethoxybenzene (0.2 equiv.) as an internal standard. This indicates that the low %ee for lactone **16B** **cannot be rationalised by a background (racemic) reaction** that favours the formation of lactone **16B**.

A 'purified' sample containing a 98:2 mixture of lactam **16A** (84% ee) and lactone **16B** (33% ee) was resubjected to the reaction conditions ($i$$Pr_2$NEt, HyperBTM, THF). Samples were removed periodically and analysed by chiral HPLC. Over a 20 h period the %ee of lactone **16B** was unchanged (4 samples, 32-34% ee), indicating that the product is **not epimerised under the reaction conditions**.

Samples were removed periodically from a standard reaction to assess the **%ee of the lactone** **16B** as a **function of reaction conversion**. The samples were purified by column chromatography as 2-phenacylbenzothiazole co-elutes with lactone **16B** on the HPLC. Separation of the products **16A** and **16B** from 2-phenacylbenzothiazole by column chromatography was also challenging and therefore adequately pure samples at very low conversion were difficult to obtain. The conversion of the reaction was determined by NMR spectroscopic analysis of the crude reaction product.

% Conversion	Lactone **16B** %ee
35	45
76	40.5
100	40.5
100 (+24 h)	42.5

No significant change in %ee of lactone **16B** was observed over the course of the reaction. This indicates that once lactone **16B** is formed no change in %ee takes place. The low stereoselectivity in this case therefore most likely arises in the formation, or epimerisation, of a reaction intermediate prior to lactonization.

HPLC Traces

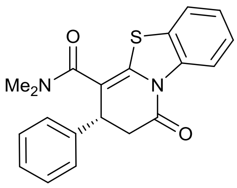

2A

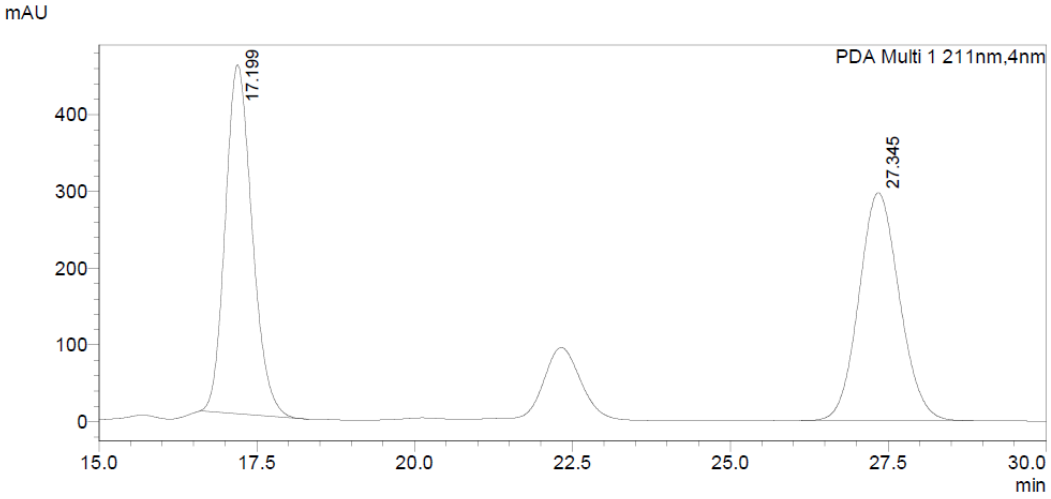

PDA Ch1 211nm

Peak#	Ret. Time	Area	Area%
1	17.199	13423555	50.349
2	27.345	13237701	49.651
Total		26661256	100.000

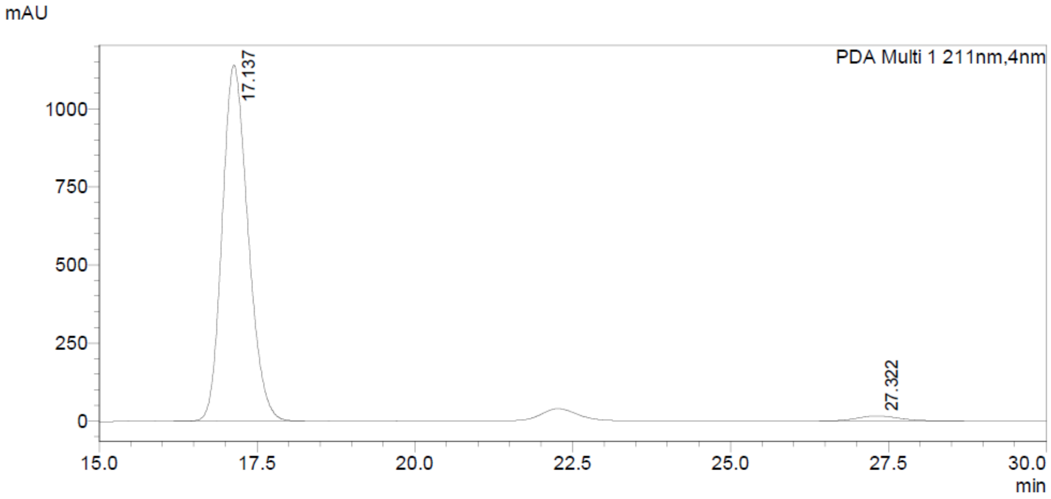

PDA Ch1 211nm

Peak#	Ret. Time	Area	Area%
1	17.137	32465565	97.877
2	27.322	704098	2.123
Total		33169663	100.000

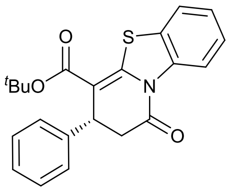

3A

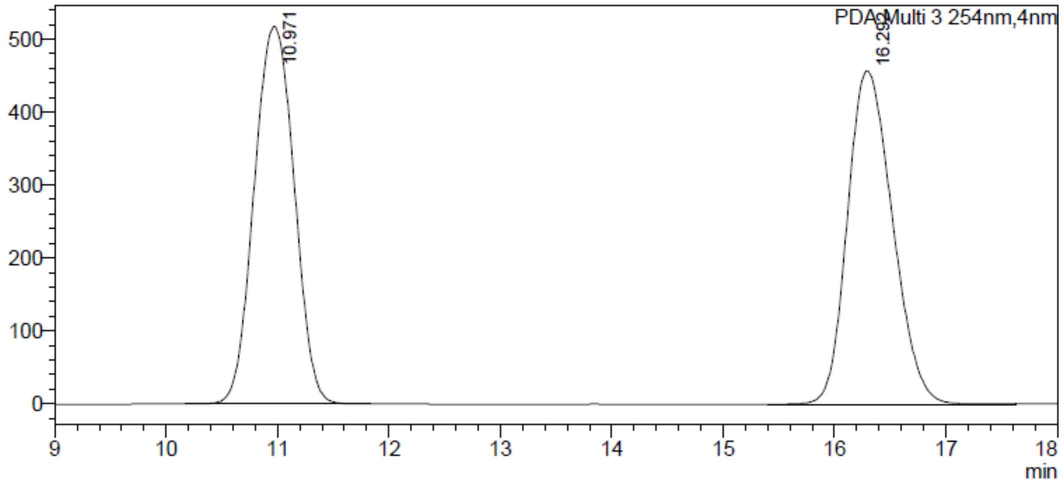

PDA Ch3 254nm

Peak#	Ret. Time	Area	Area%
1	10.971	12943282	50.085
2	16.292	12899457	49.915
Total		25842739	100.000

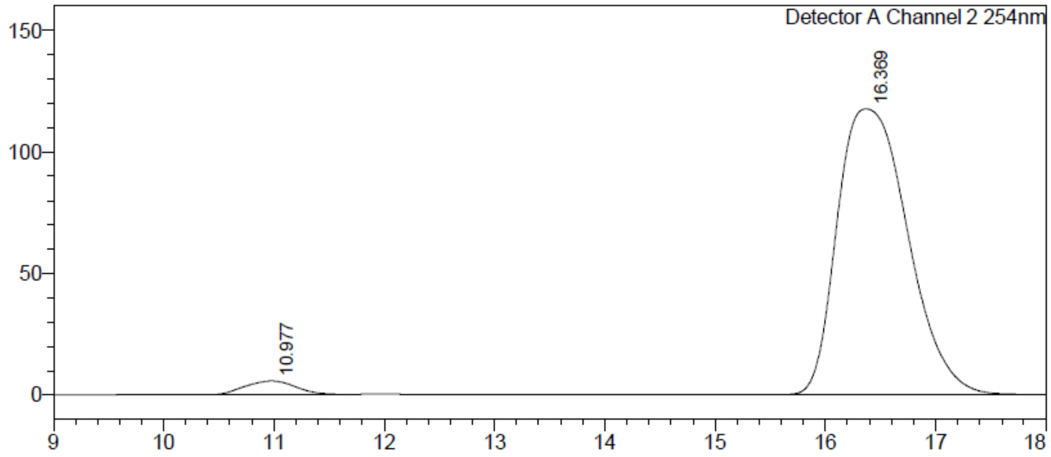

Detector A Channel 2 254nm

Peak#	Ret. Time	Area%
1	10.977	3.265
2	16.369	96.735
Total		100.000

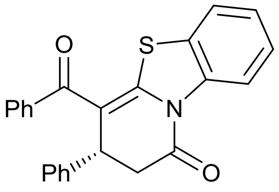

4A

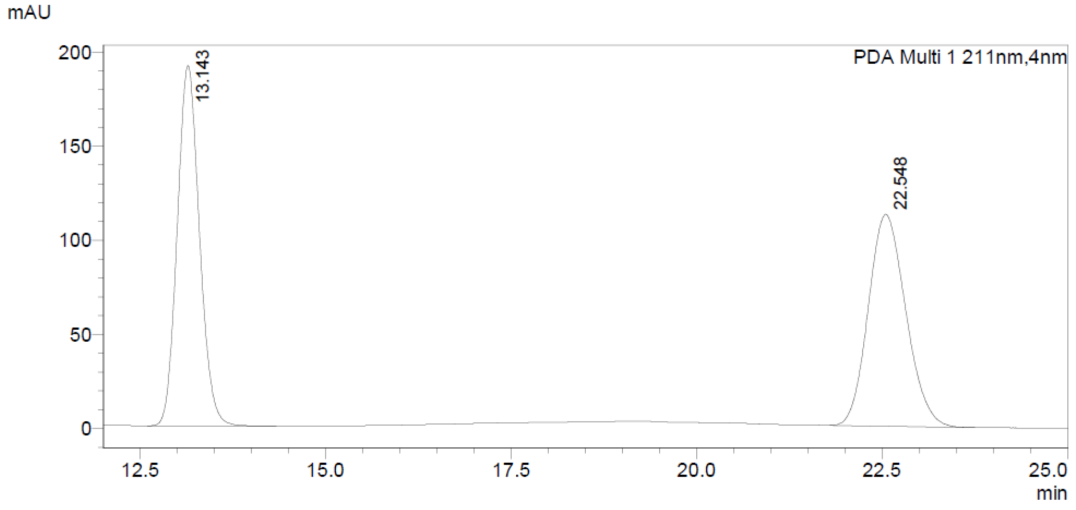

<Peak Table>

PDA Ch1 211nm

Peak#	Ret. Time	Area	Area%
1	13.143	3989307	50.198
2	22.548	3957761	49.802
Total		7947068	100.000

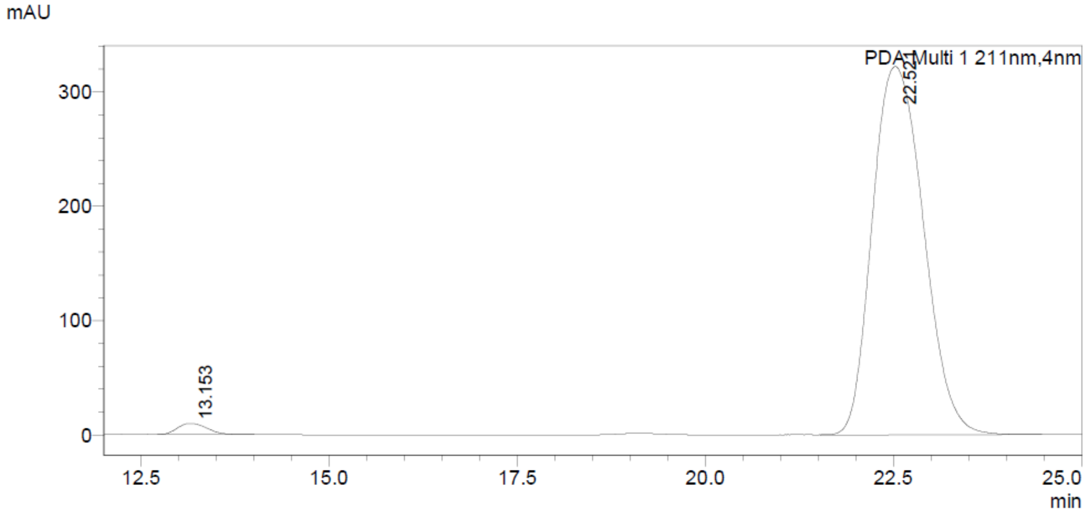

<Peak Table>

PDA Ch1 211nm

Peak#	Ret. Time	Area	Area%
1	13.153	267397	1.721
2	22.521	15266872	98.279
Total		15534269	100.000

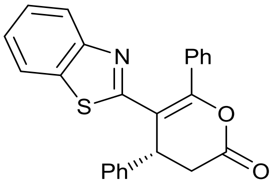

4B

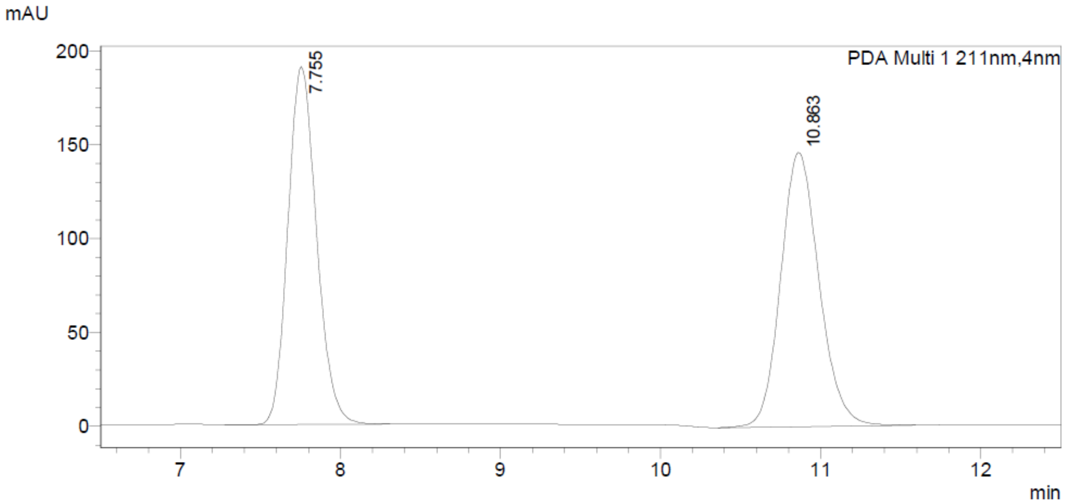

<Peak Table>

PDA Ch1 211nm

Peak#	Ret. Time	Area	Area%
1	7.755	2374617	49.400
2	10.863	2432318	50.600
Total		4806935	100.000

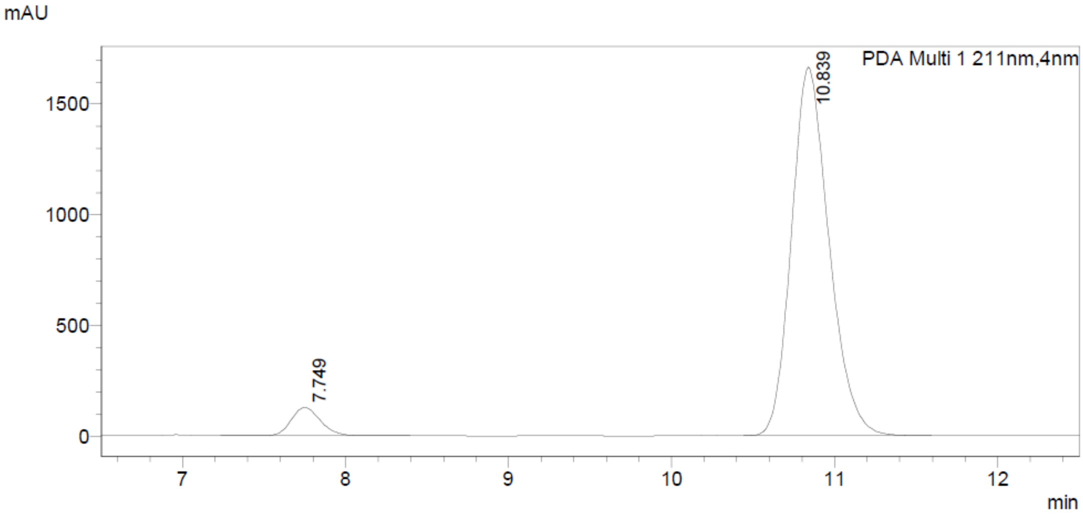

<Peak Table>

PDA Ch1 211nm

Peak#	Ret. Time	Area	Area%
1	7.749	1613932	5.704
2	10.839	26682109	94.296
Total		28296041	100.000

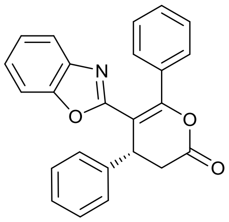

5B

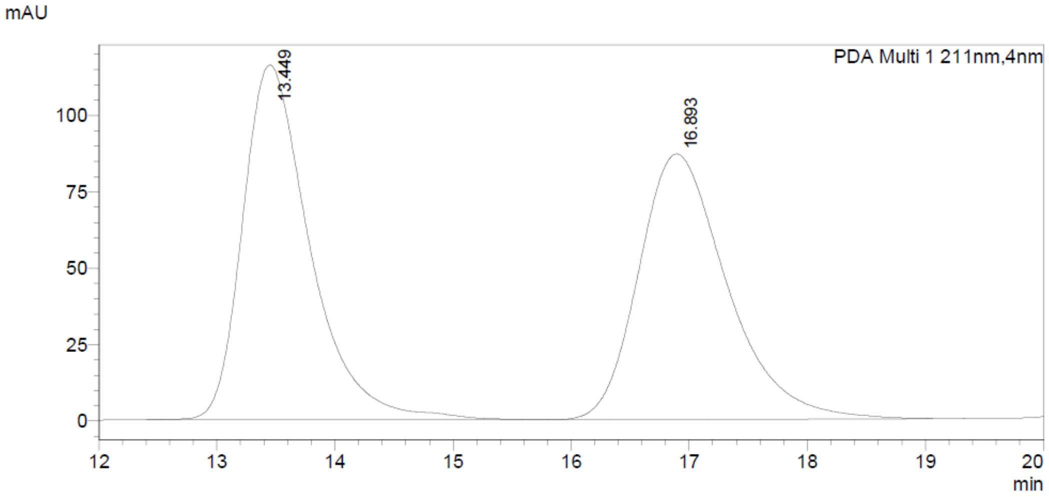

PDA Ch1 211nm

Peak#	Ret. Time	Area	Area%
1	13.449	4629365	50.551
2	16.893	4528461	49.449
Total		9157826	100.000

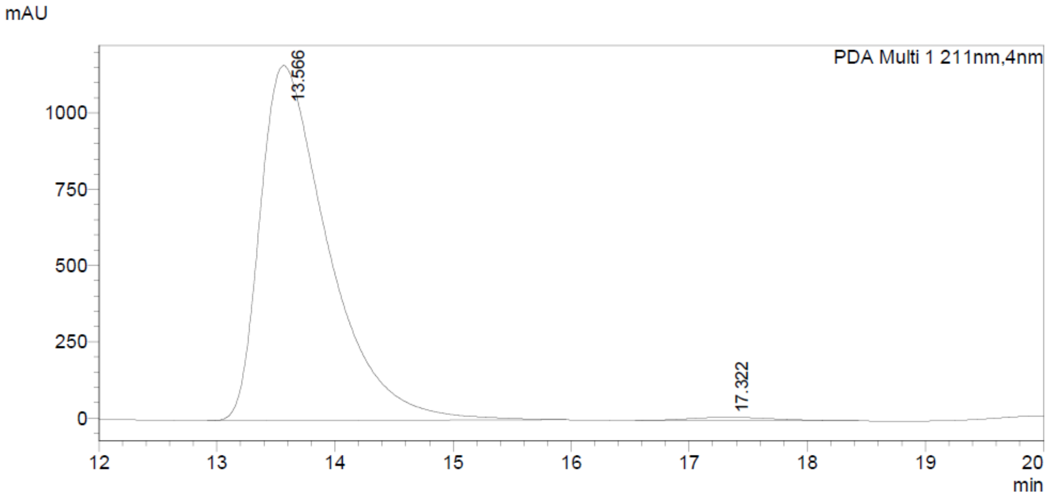

PDA Ch1 211nm

Peak#	Ret. Time	Area	Area%
1	13.566	46763276	98.844
2	17.322	546710	1.156
Total		47309986	100.000

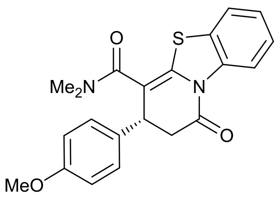

6A

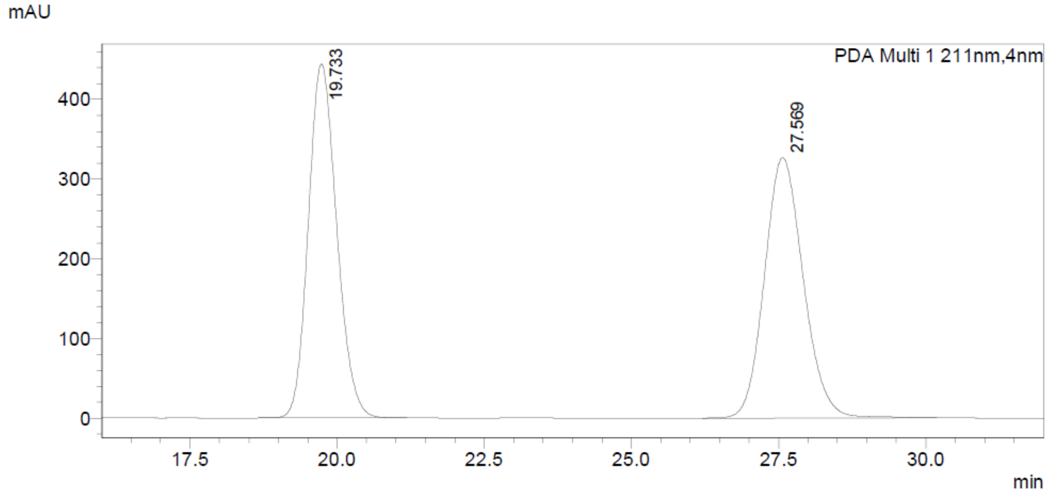

PDA Ch1 211nm

Peak#	Ret. Time	Area	Area%
1	19.733	14917719	49.850
2	27.569	15007219	50.150
Total		29924937	100.000

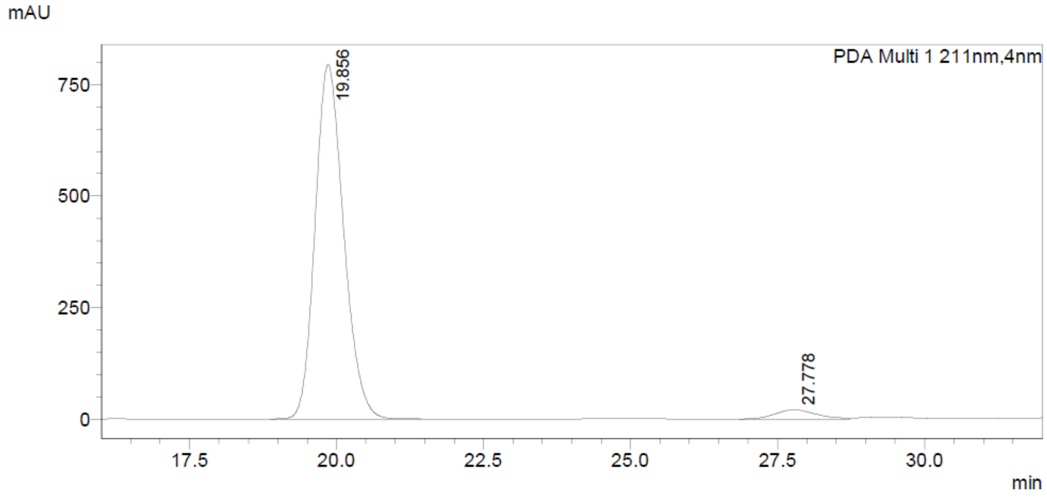

PDA Ch1 211nm

Peak#	Ret. Time	Area	Area%
1	19.856	26812855	96.385
2	27.778	1005557	3.615
Total		27818412	100.000

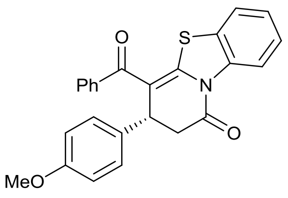

7A

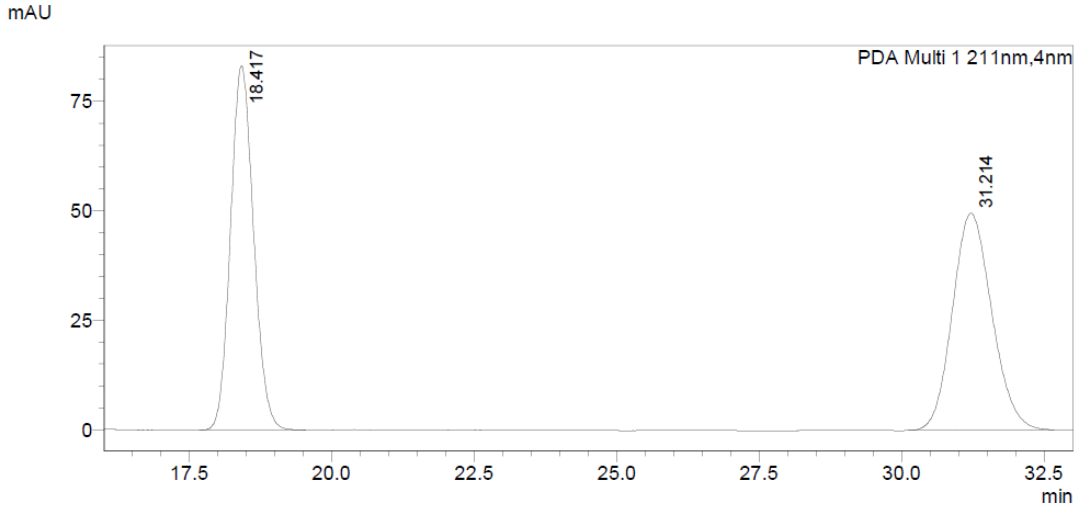

<Peak Table>

PDA Ch1 211nm

Peak#	Ret. Time	Area	Area%
1	18.417	2348456	49.550
2	31.214	2385992	50.342
3	33.808	5114	0.108
Total		4739562	100.000

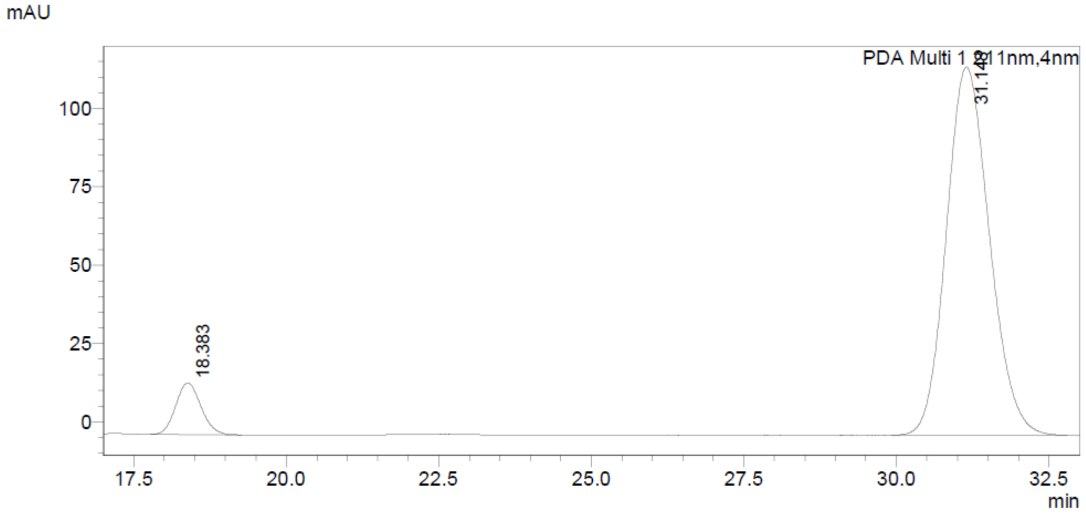

<Peak Table>

PDA Ch1 211nm

Peak#	Ret. Time	Area	Area%
1	18.383	473352	7.589
2	31.148	5763671	92.411
Total		6237023	100.000

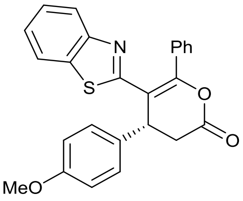

7B

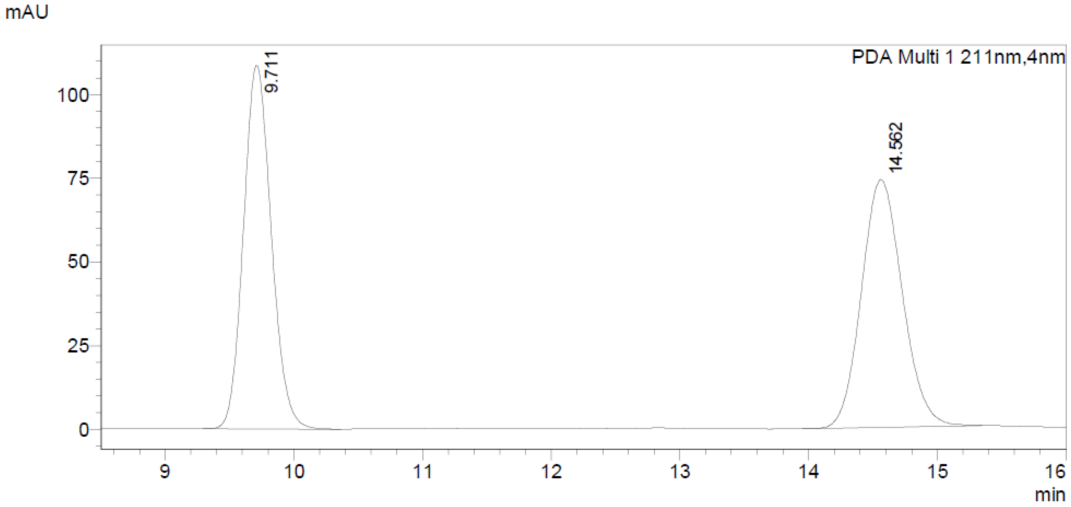

<Peak Table>

PDA Ch1 211nm

Peak#	Ret. Time	Area	Area%
1	9.711	1636034	49.971
2	14.562	1637944	50.029
Total		3273978	100.000

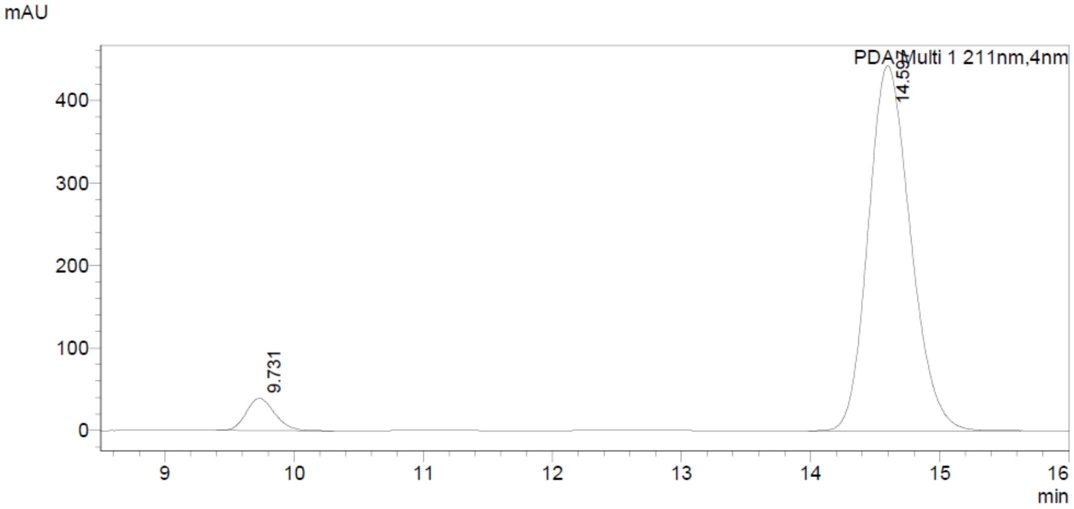

<Peak Table>

PDA Ch1 211nm

Peak#	Ret. Time	Area	Area%
1	9.731	604793	5.677
2	14.597	10047698	94.323
Total		10652491	100.000

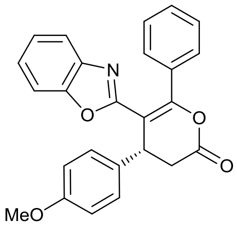

8B

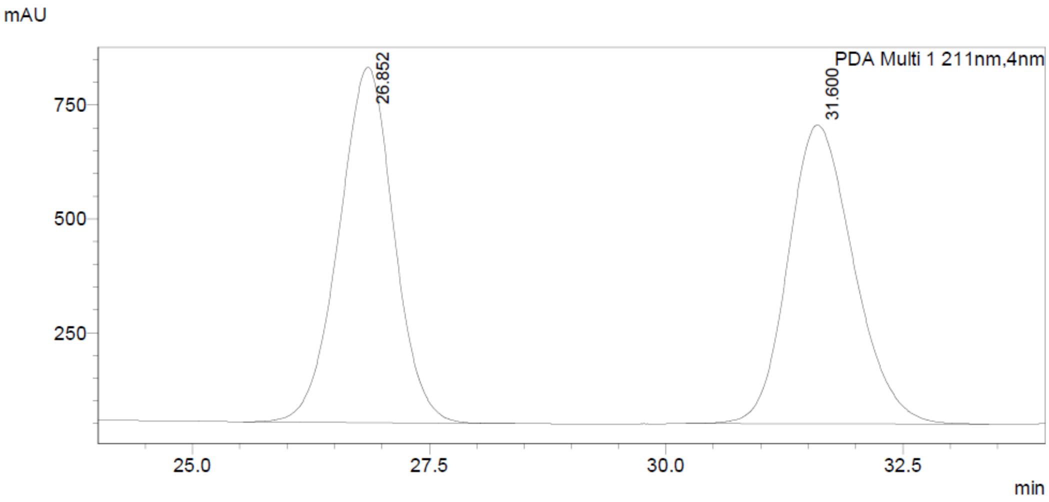

PDA Ch1 211nm

Peak#	Ret. Time	Area	Area%
1	26.852	31566235	49.936
2	31.600	31646999	50.064
Total		63213234	100.000

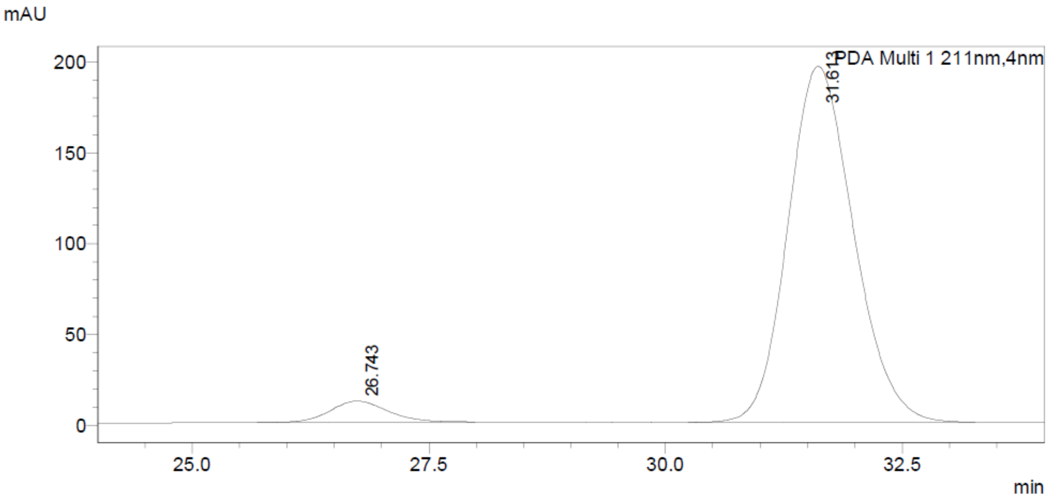

PDA Ch1 211nm

Peak#	Ret. Time	Area	Area%
1	26.743	499216	5.017
2	31.613	9450380	94.983
Total		9949596	100.000

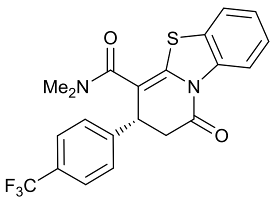

9A

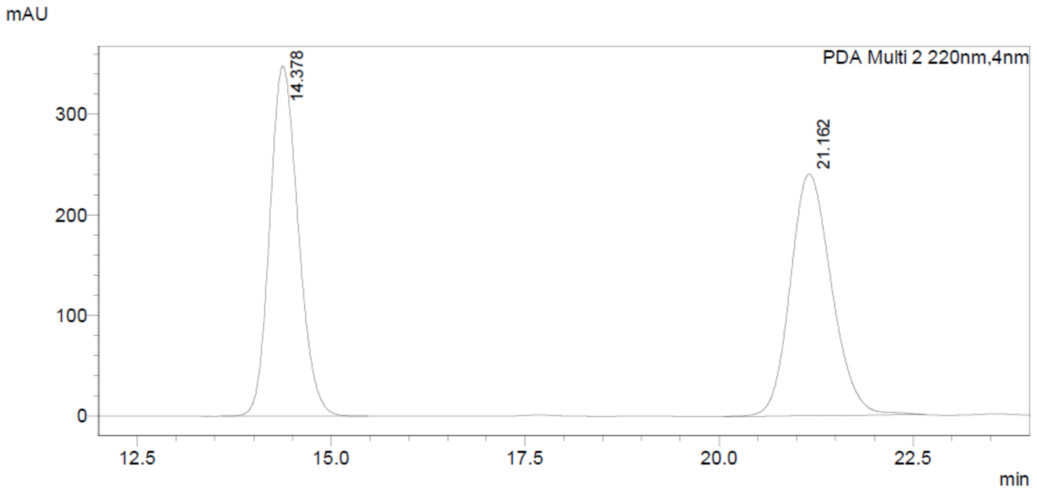

PDA Ch2 220nm

Peak#	Ret. Time	Area	Area%
1	14.378	8903303	50.002
2	21.162	8902484	49.998
Total		17805786	100.000

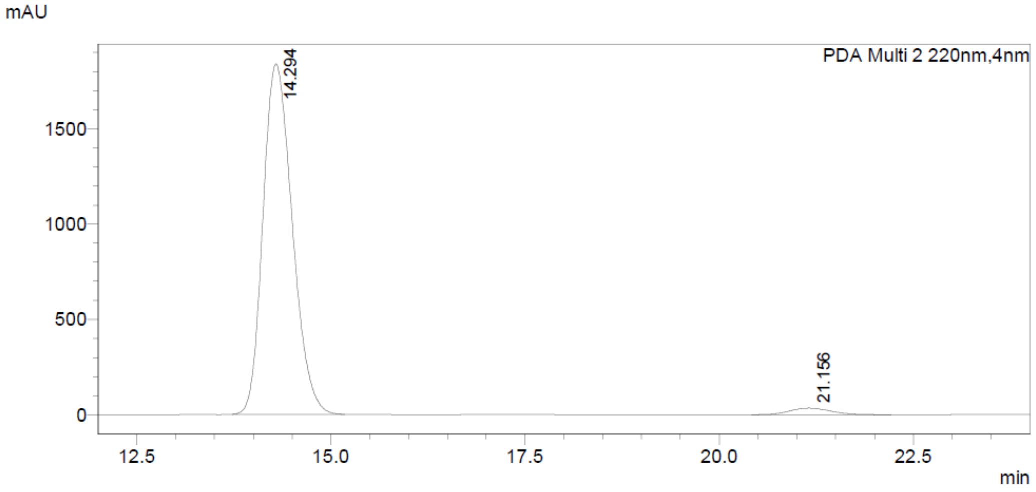

PDA Ch2 220nm

Peak#	Ret. Time	Area	Area%
1	14.294	48218576	97.365
2	21.156	1305191	2.635
Total		49523767	100.000

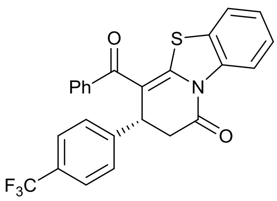

10A

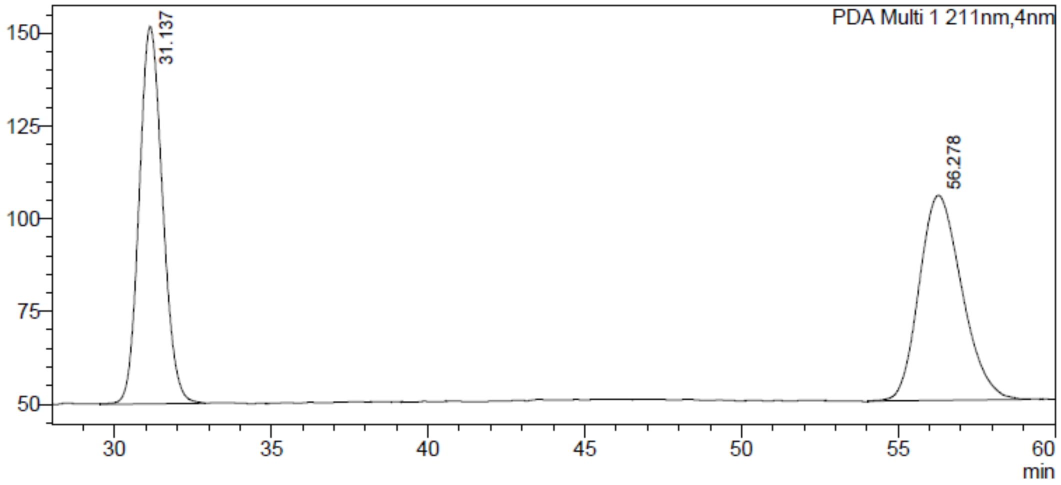

PDA Ch1 211nm

Peak#	Ret. Time	Area	Area%
1	31.137	5276545	50.140
2	56.278	5247080	49.860
Total		10523624	100.000

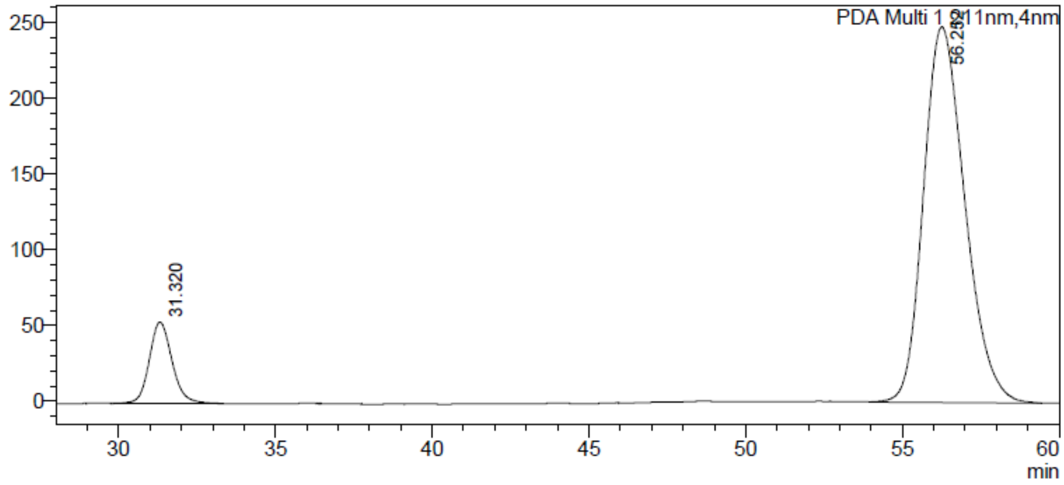

PDA Ch1 211nm

Peak#	Ret. Time	Area	Area%
1	31.320	2721184	10.685
2	56.252	22745062	89.315
Total		25466247	100.000

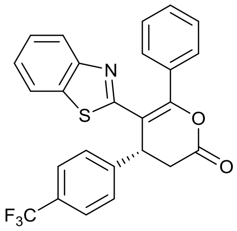

10B

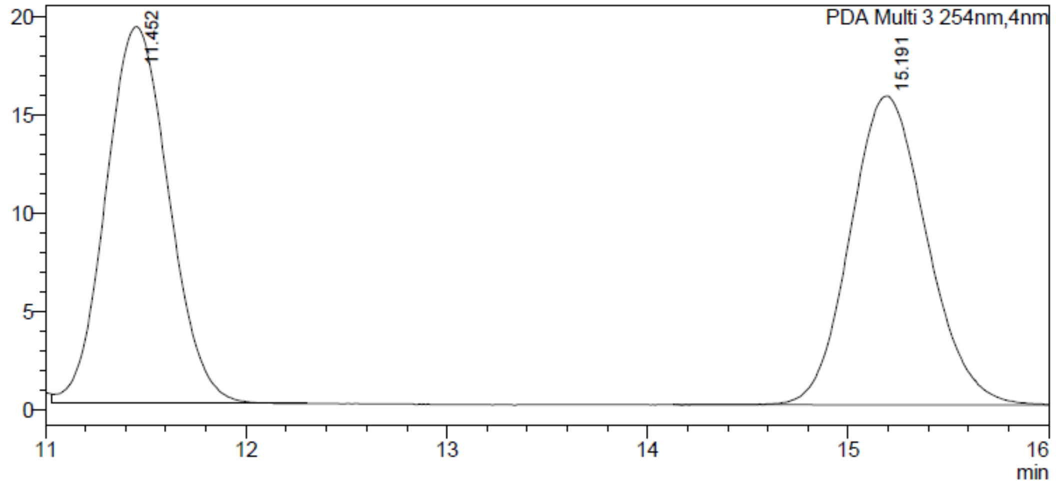

PDA Ch3 254nm

Peak#	Ret. Time	Area	Area%
1	11.452	419836	50.155
2	15.191	417235	49.845
Total		837071	100.000

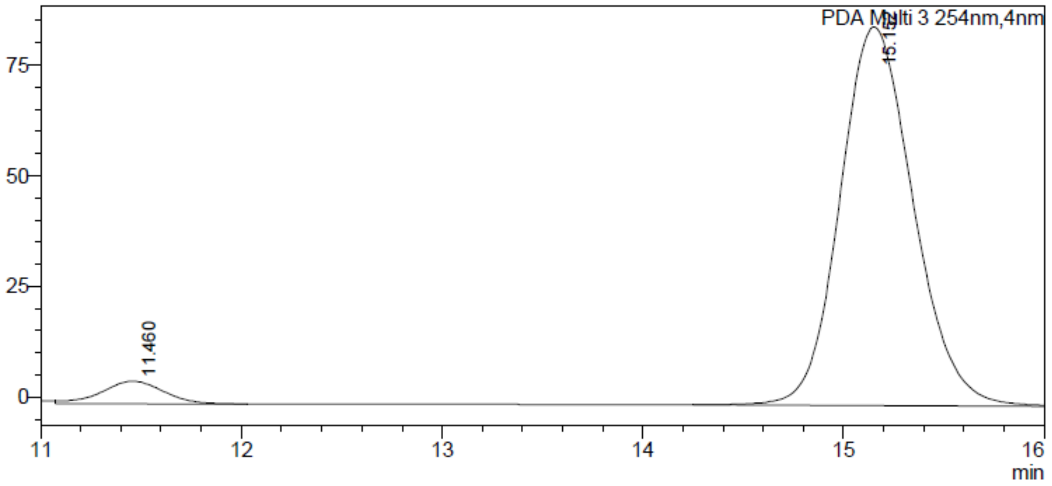

PDA Ch3 254nm

Peak#	Ret. Time	Area	Area%
1	11.460	109959	4.829
2	15.152	2167031	95.171
Total		2276990	100.000

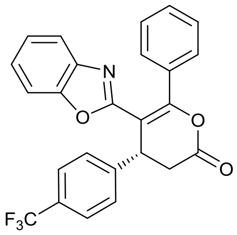

11B

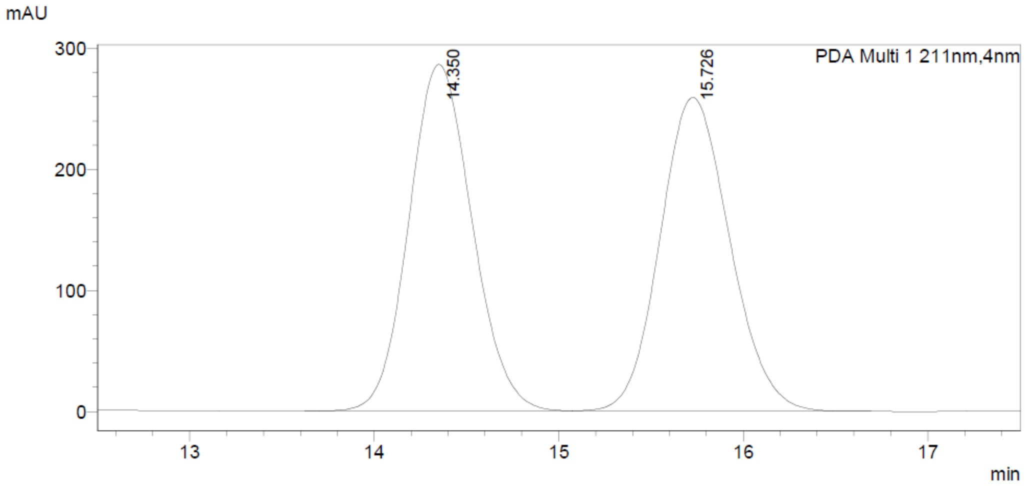

PDA Ch1 211nm

Peak#	Ret. Time	Area	Area%
1	14.350	6818411	50.036
2	15.726	6808473	49.964
Total		13626884	100.000

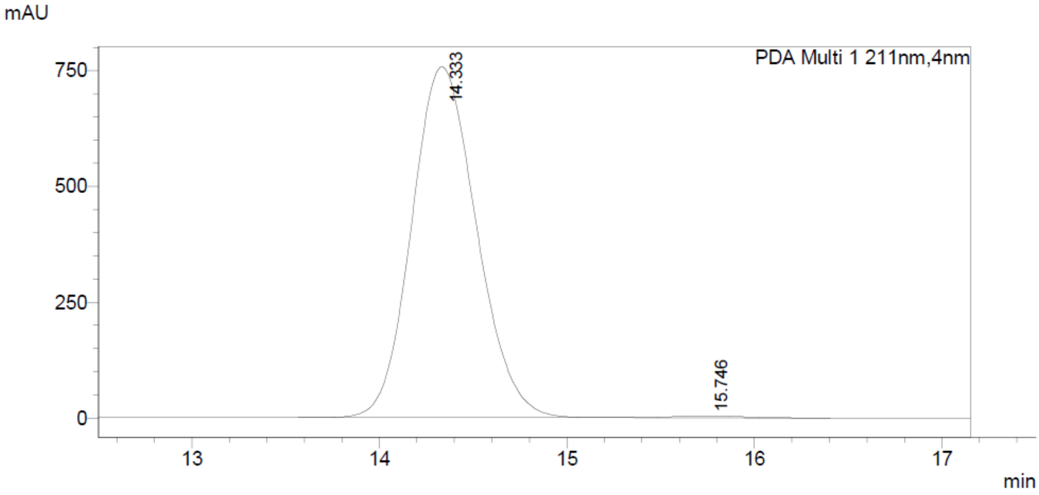

PDA Ch1 211nm

Peak#	Ret. Time	Area	Area%
1	14.333	18230845	99.414
2	15.746	107501	0.586
Total		18338346	100.000

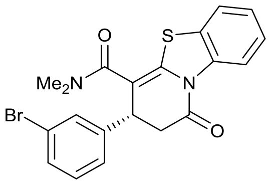

12A

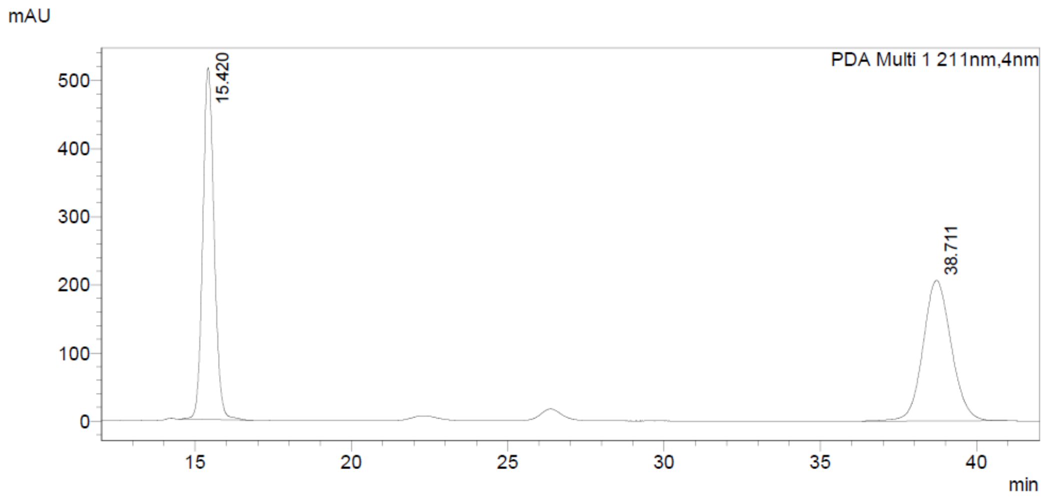

PDA Ch1 211nm

Peak#	Ret. Time	Area	Area%
1	15.420	13041943	50.175
2	38.711	12950756	49.825
Total		25992699	100.000

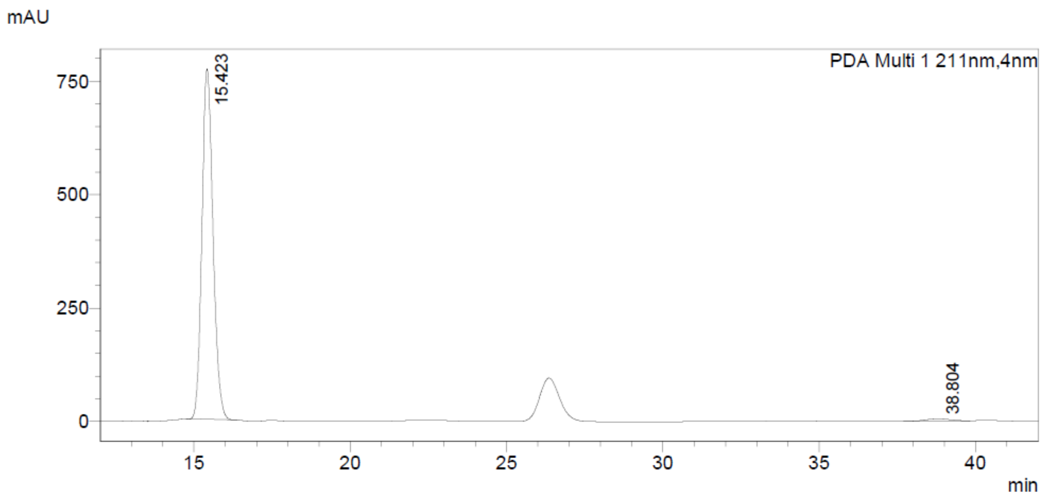

PDA Ch1 211nm

Peak#	Ret. Time	Area	Area%
1	15.423	19017246	98.113
2	38.804	365706	1.887
Total		19382953	100.000

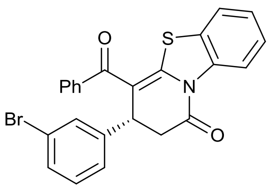

13A

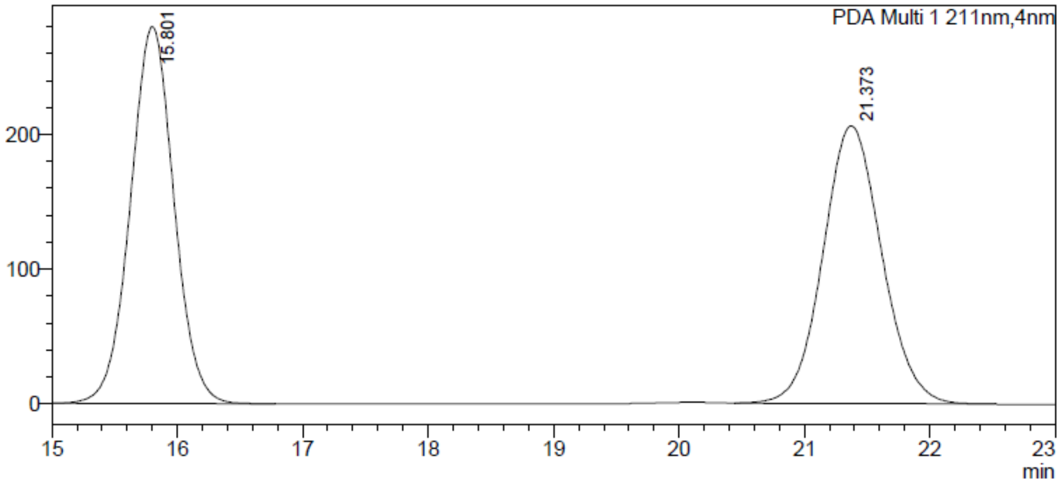

PDA Ch1 211nm

Peak#	Ret. Time	Area	Area%
1	15.801	6785642	50.240
2	21.373	6720773	49.760
Total		13506416	100.000

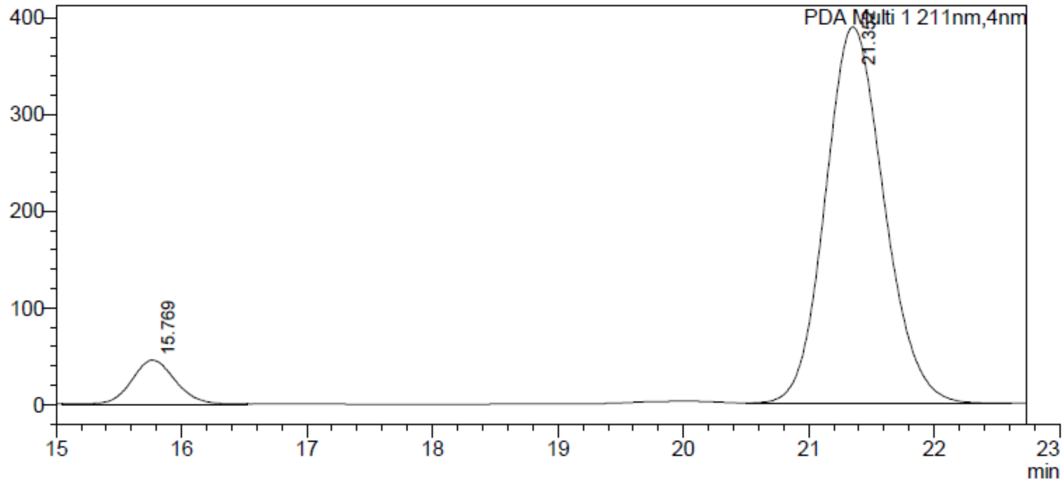

PDA Ch1 211nm

Peak#	Ret. Time	Area	Area%
1	15.769	1090842	7.912
2	21.352	12695526	92.088
Total		13786369	100.000

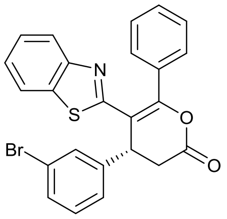

13B

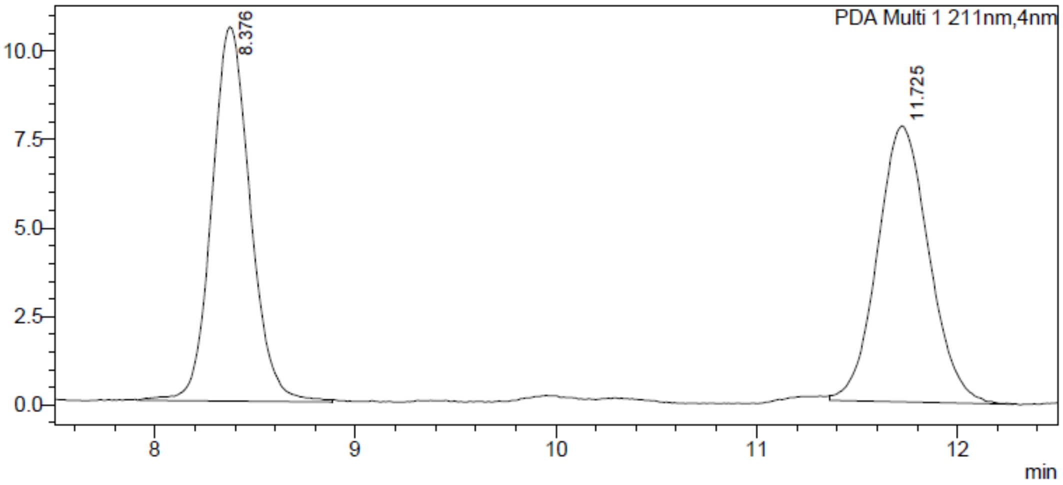

PDA Ch1 211nm

Peak#	Ret. Time	Area	Area%
1	8.376	140634	50.233
2	11.725	139330	49.767
Total		279965	100.000

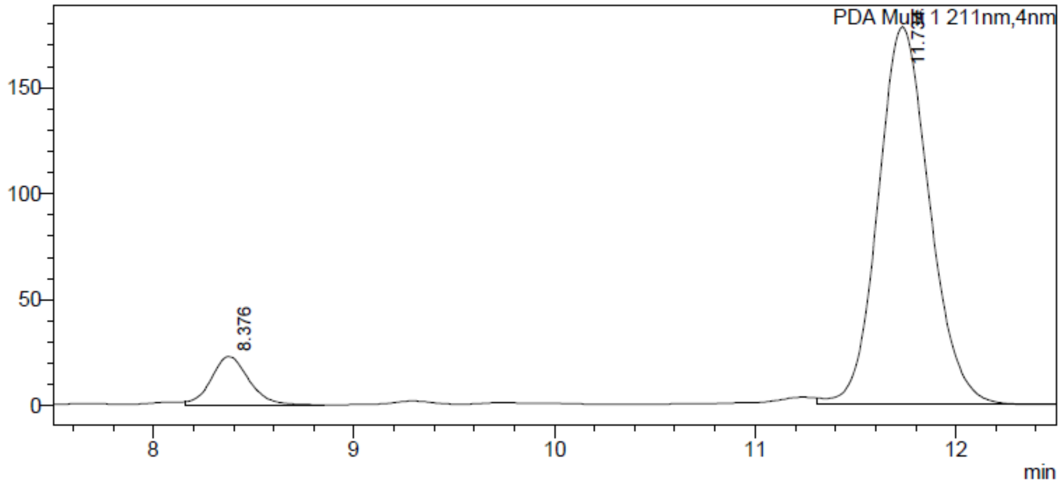

PDA Ch1 211nm

Peak#	Ret. Time	Area	Area%
1	8.376	292430	8.443
2	11.734	3171286	91.557
Total		3463717	100.000

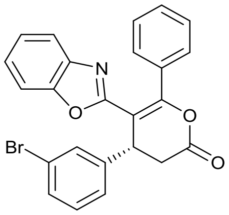

14B

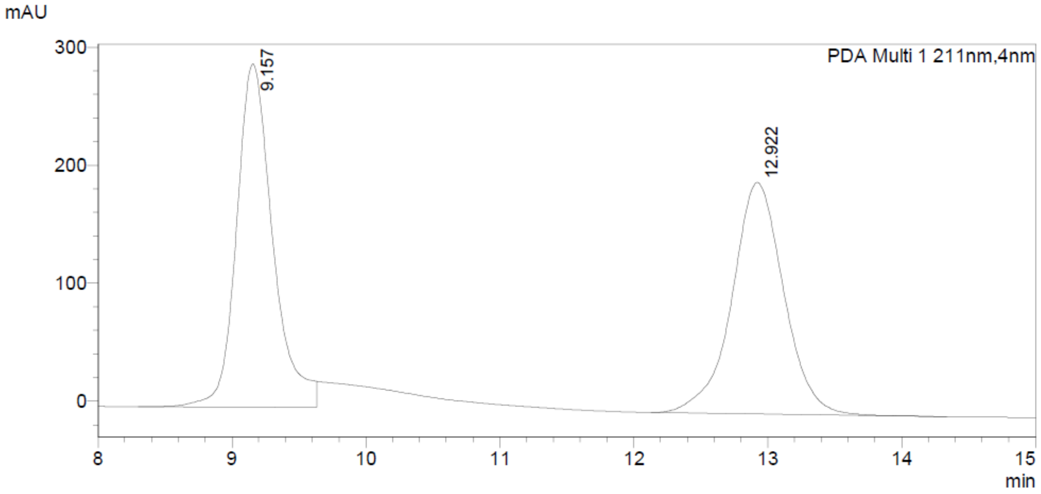

PDA Ch1 211nm

Peak#	Ret. Time	Area	Area%
1	9.157	5453169	50.733
2	12.922	5295488	49.267
Total		10748657	100.000

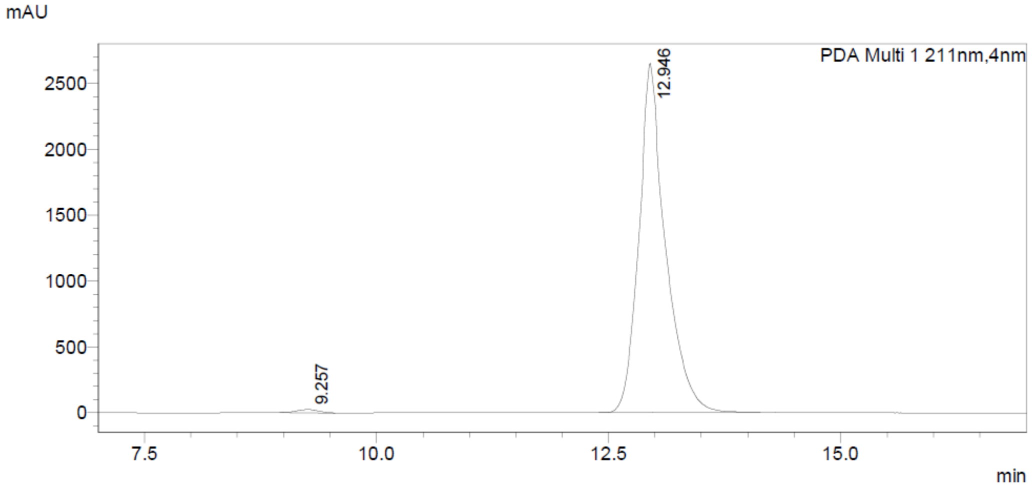

PDA Ch1 211nm

Peak#	Ret. Time	Area	Area%
1	9.257	360331	0.679
2	12.946	52671002	99.321
Total		53031332	100.000

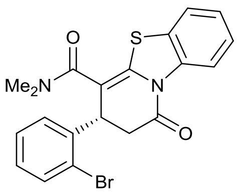

15A

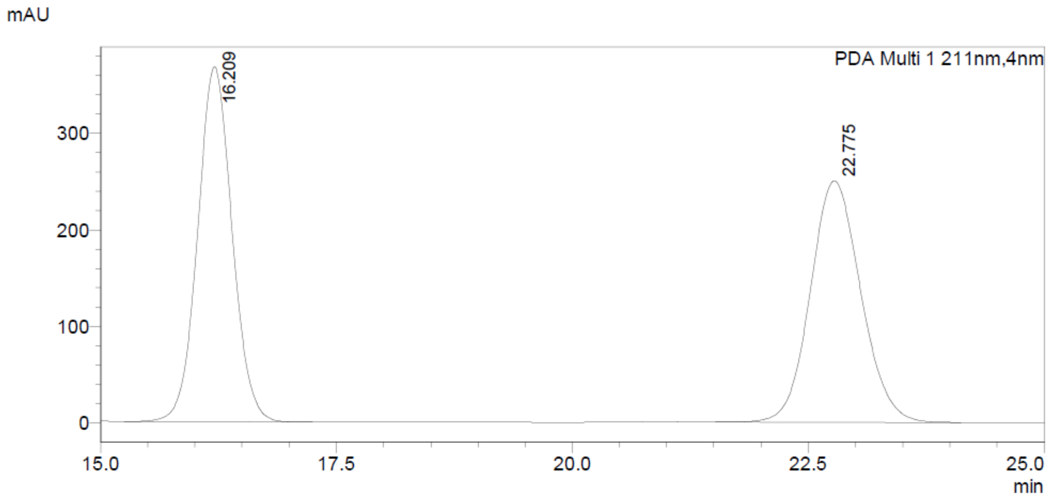

PDA Ch1 211nm

Peak#	Ret. Time	Area	Area%
1	16.209	9339800	50.147
2	22.775	9284974	49.853
Total		18624774	100.000

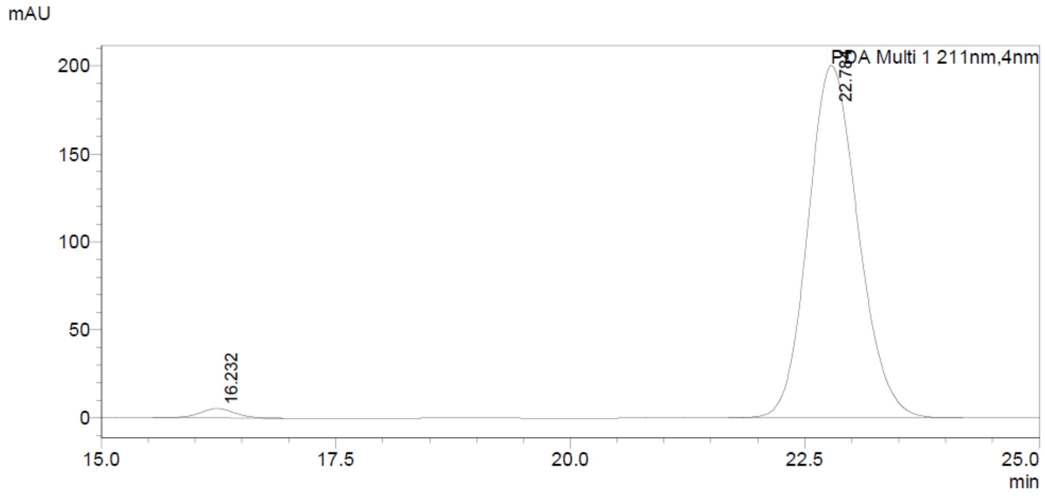

PDA Ch1 211nm

Peak#	Ret. Time	Area	Area%
1	16.232	136023	1.790
2	22.784	7461998	98.210
Total		7598021	100.000

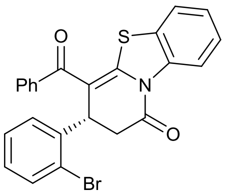

16A

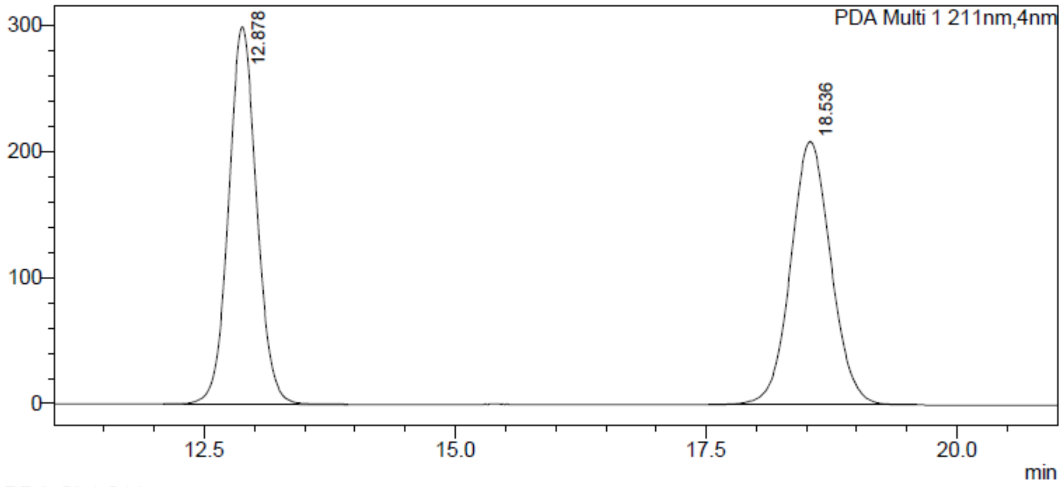

PDA Ch1 211nm

Peak#	Ret. Time	Area	Area%
1	12.878	5832173	50.077
2	18.536	5814337	49.923
Total		11646510	100.000

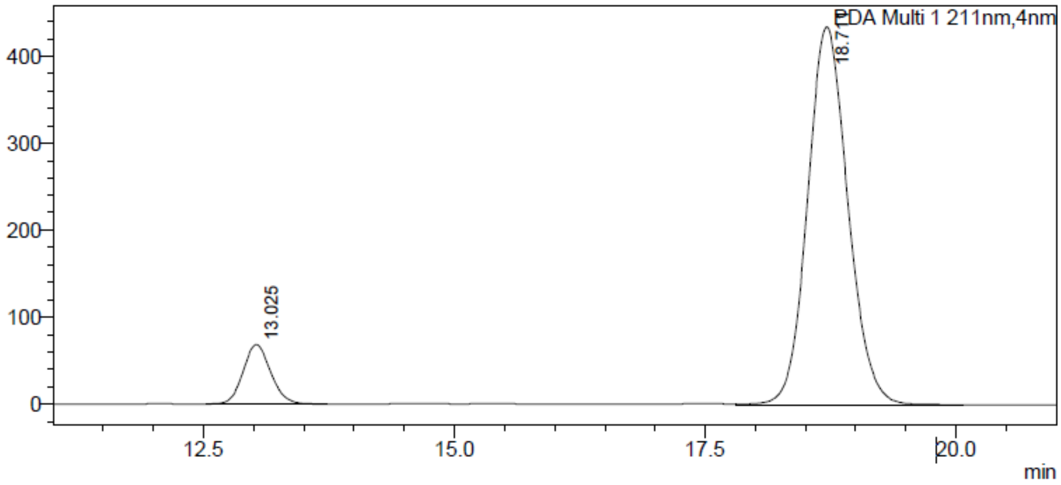

PDA Ch1 211nm

Peak#	Ret. Time	Area	Area%
1	13.025	1310340	9.747
2	18.711	12133172	90.253
Total		13443511	100.000

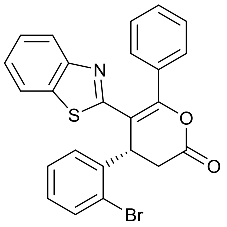

16B

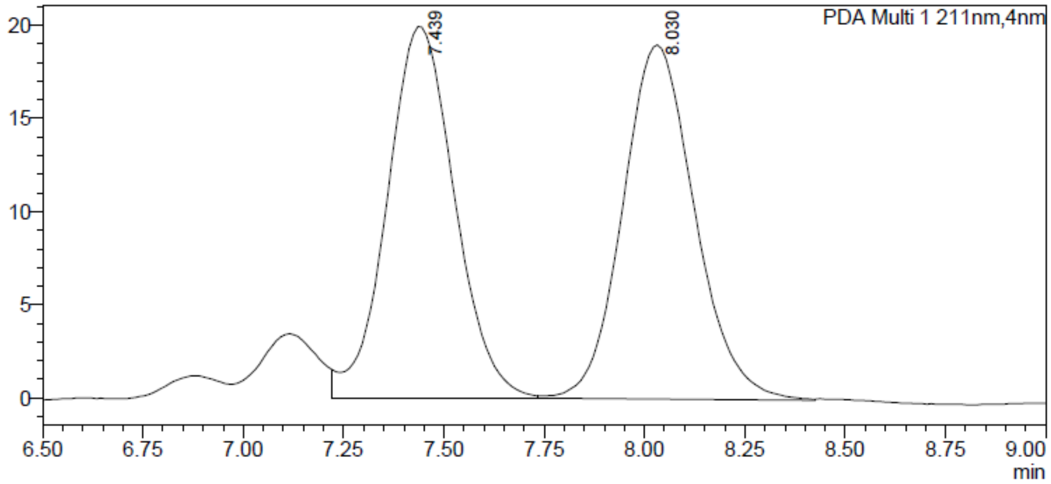

PDA Ch1 211nm

Peak#	Ret. Time	Area	Area%
1	7.439	232636	49.545
2	8.030	236914	50.455
Total		469550	100.000

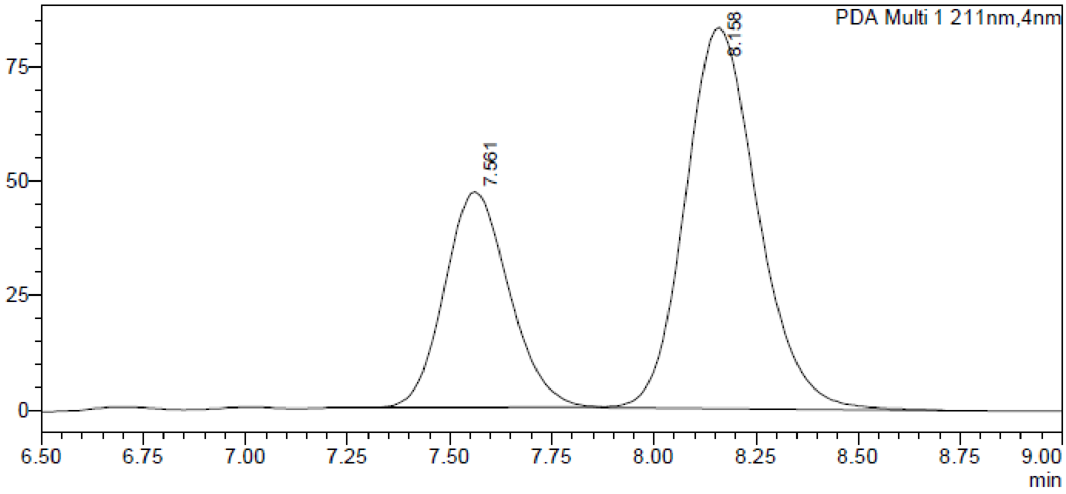

PDA Ch1 211nm

Peak#	Ret. Time	Area	Area%
1	7.561	519184	33.584
2	8.158	1026720	66.416
Total		1545904	100.000

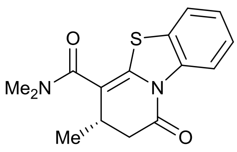

17A

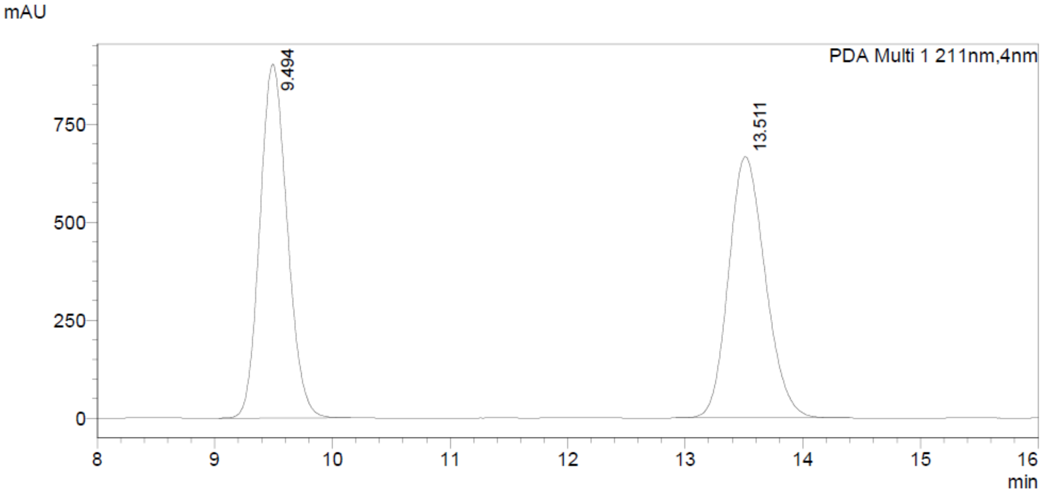

PDA Ch1 211nm

Peak#	Ret. Time	Area	Area%
1	9.494	14623818	50.179
2	13.511	14519294	49.821
Total		29143111	100.000

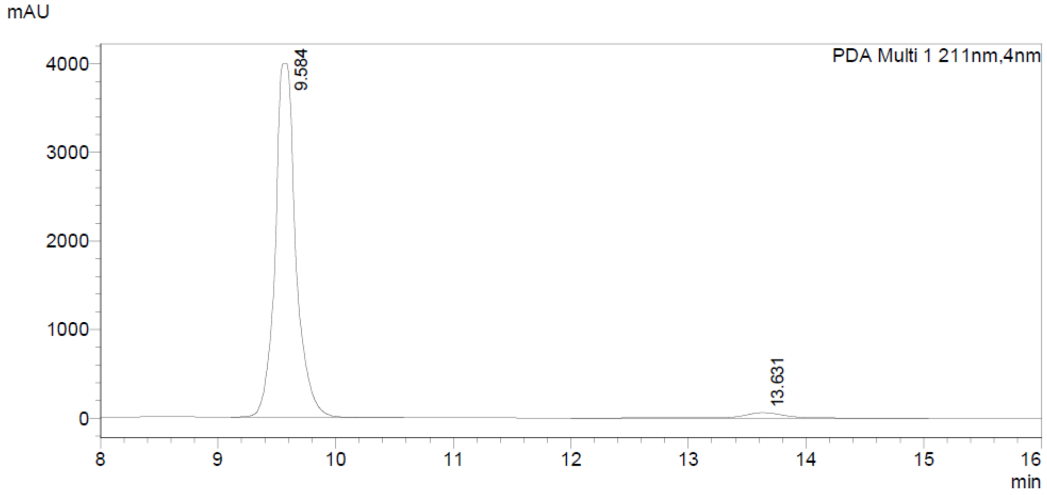

PDA Ch1 211nm

Peak#	Ret. Time	Area	Area%
1	9.584	48660768	97.041
2	13.631	1483743	2.959
Total		50144511	100.000

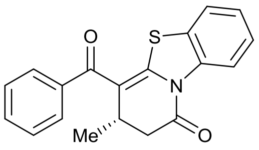

18A

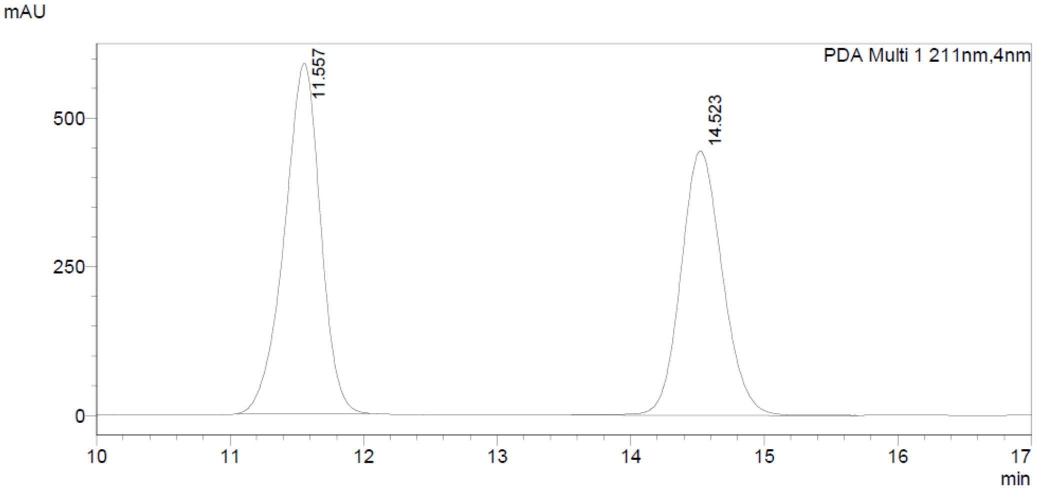

PDA Ch1 211nm

Peak#	Ret. Time	Area	Area%
1	11.557	11073949	53.664
2	14.523	9561574	46.336
Total		20635523	100.000

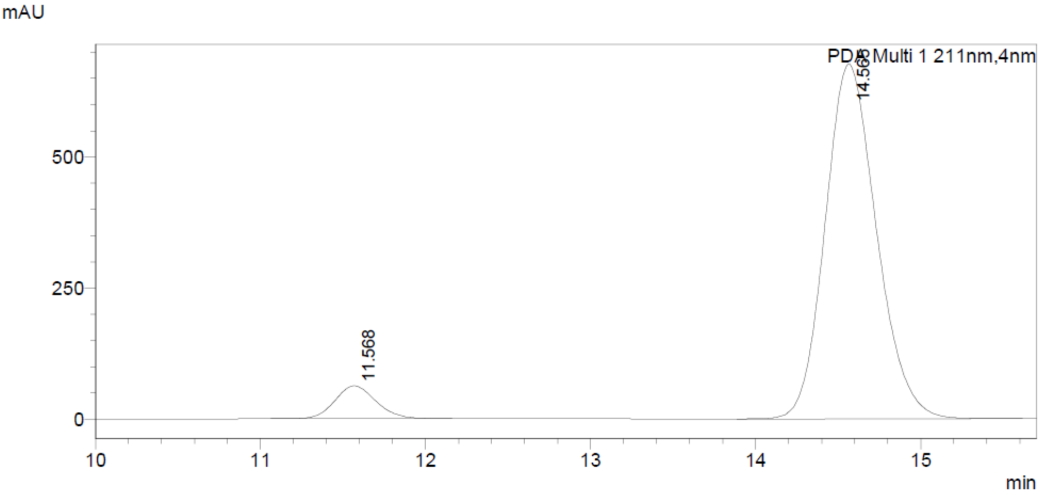

PDA Ch1 211nm

Peak#	Ret. Time	Area	Area%
1	11.568	1093412	6.957
2	14.565	14623833	93.043
Total		15717245	100.000

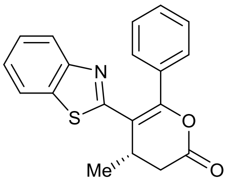

18B

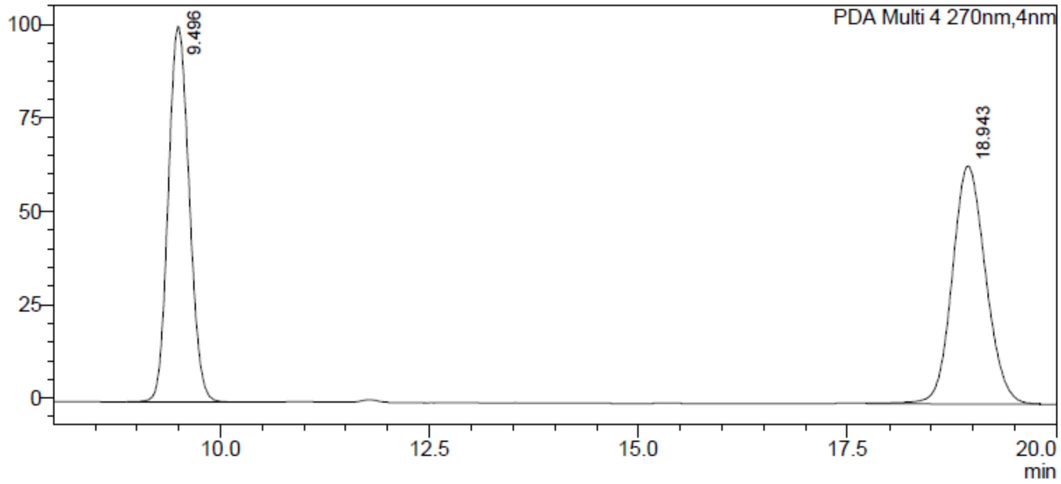

PDA Ch4 270nm

Peak#	Ret. Time	Area	Area%
1	9.496	1752699	49.709
2	18.943	1773202	50.291
Total		3525901	100.000

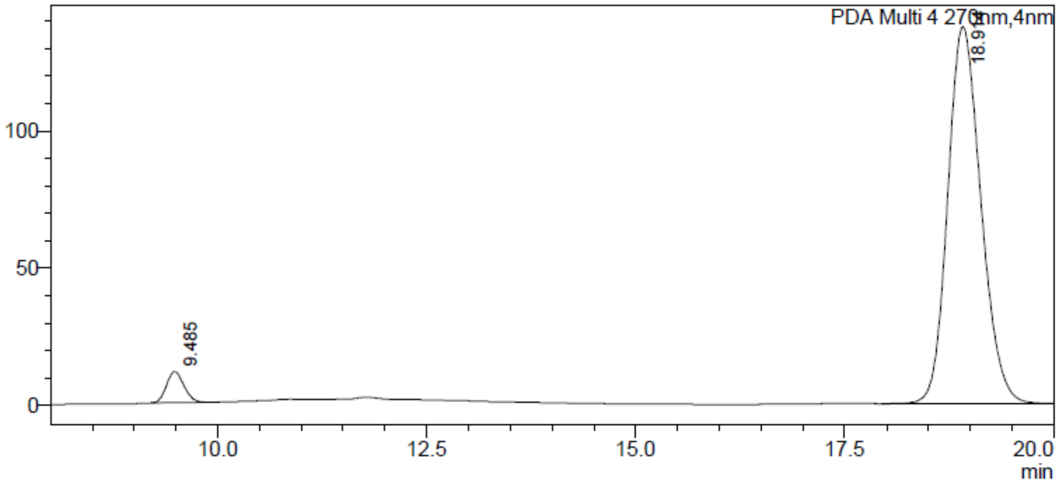

PDA Ch4 270nm

Peak#	Ret. Time	Area	Area%
1	9.485	164743	4.153
2	18.914	3802476	95.847
Total		3967219	100.000

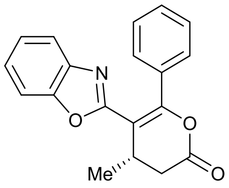

19B

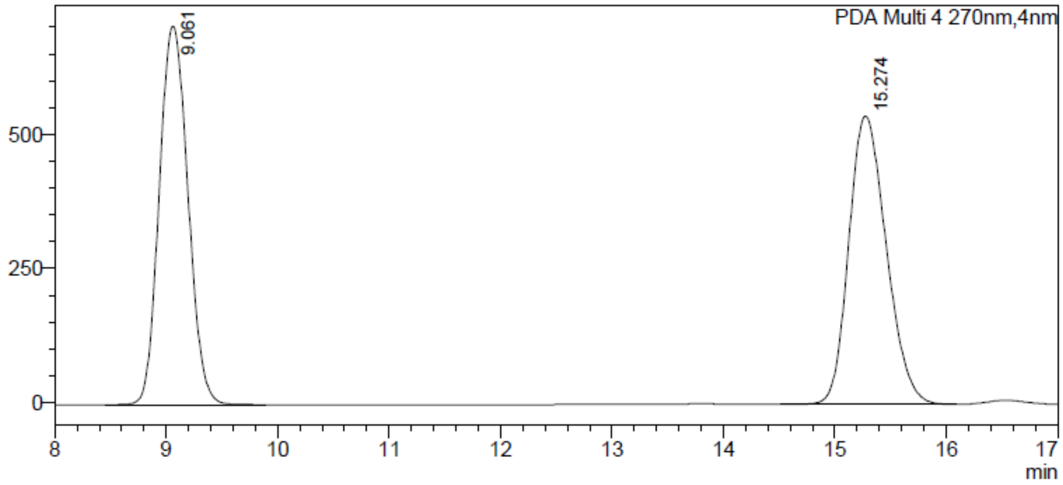

PDA Ch4 270nm

Peak#	Ret. Time	Area	Area%
1	9.061	12612050	50.059
2	15.274	12582260	49.941
Total		25194310	100.000

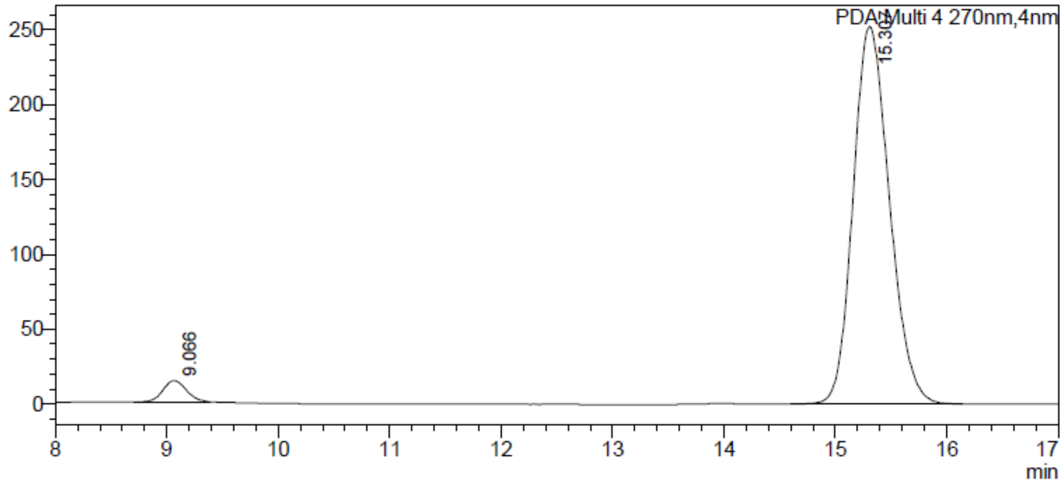

PDA Ch4 270nm

Peak#	Ret. Time	Area	Area%
1	9.066	206825	3.497
2	15.307	5707076	96.503
Total		5913902	100.000

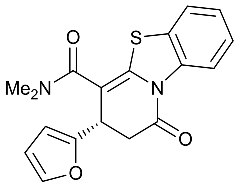

20A

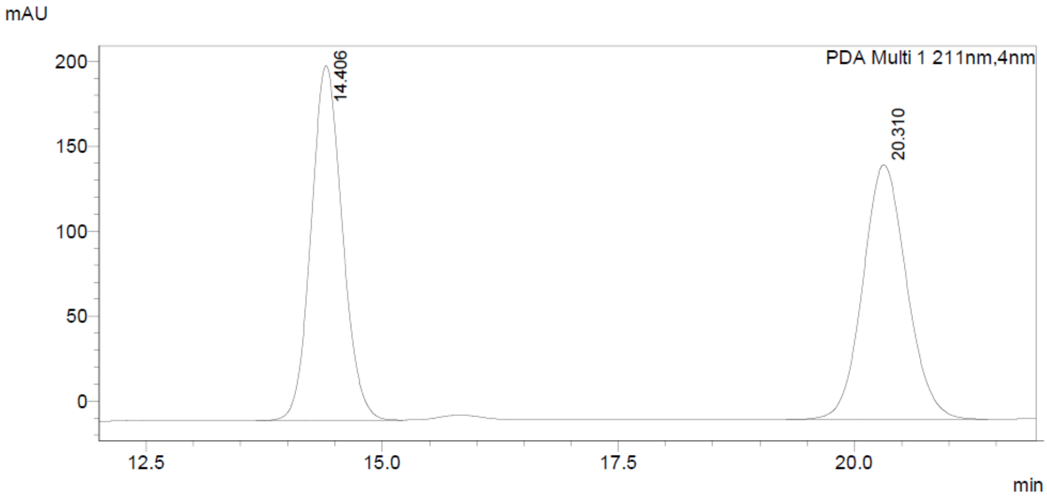

PDA Ch1 211nm

Peak#	Ret. Time	Area	Area%
1	14.406	4811488	49.883
2	20.310	4833967	50.117
Total		9645455	100.000

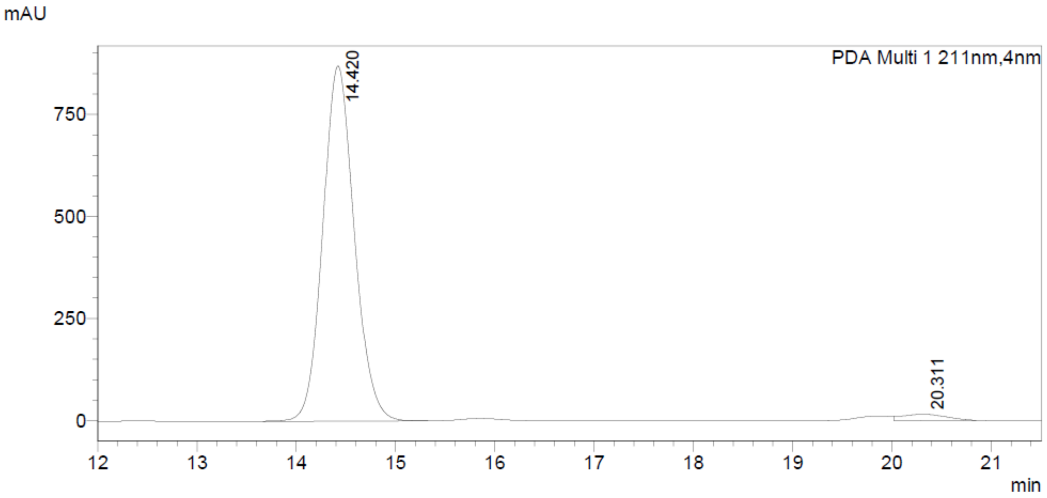

PDA Ch1 211nm

Peak#	Ret. Time	Area	Area%
1	14.420	19157990	97.547
2	20.311	481705	2.453
Total		19639695	100.000

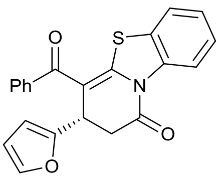

21A

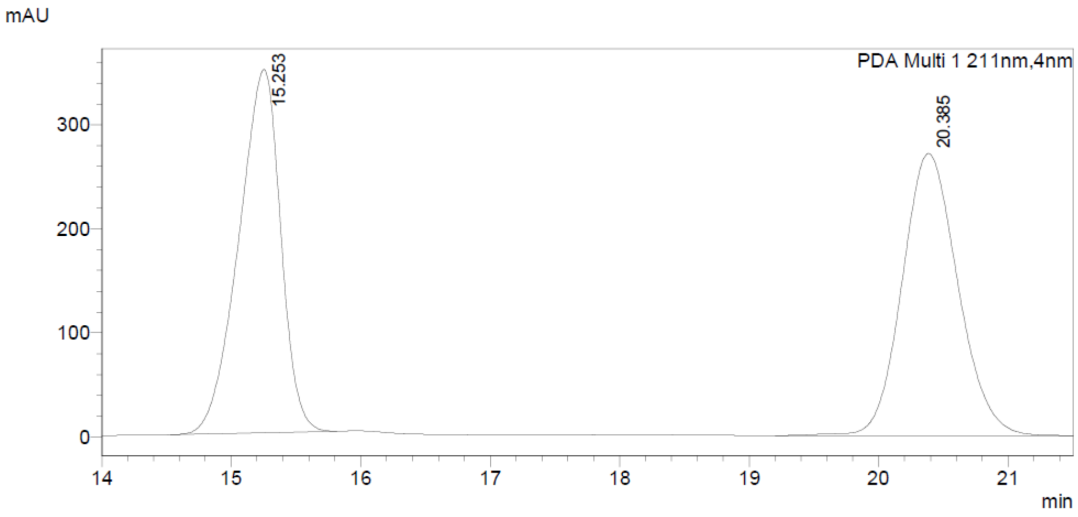

<Peak Table>

PDA Ch1 211nm

Peak#	Ret. Time	Area	Area%
1	15.253	7874774	49.261
2	20.385	8110945	50.739
Total		15985719	100.000

<Chromatogram>

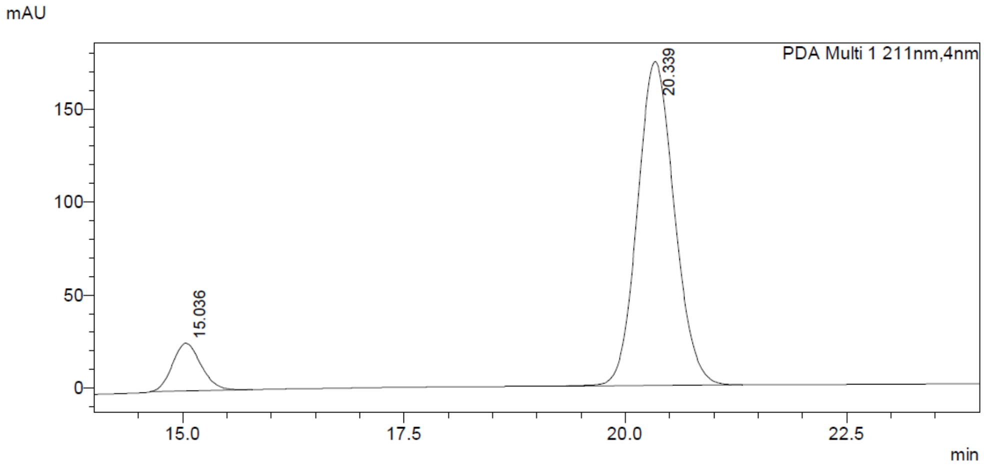

<Peak Table>

PDA Ch1 211nm

Peak#	Ret. Time	Area	Area%
1	15.036	580528	10.282
2	20.339	5065469	89.718
Total		5645997	100.000

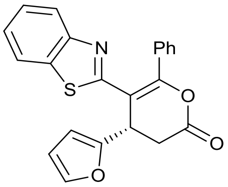

21B

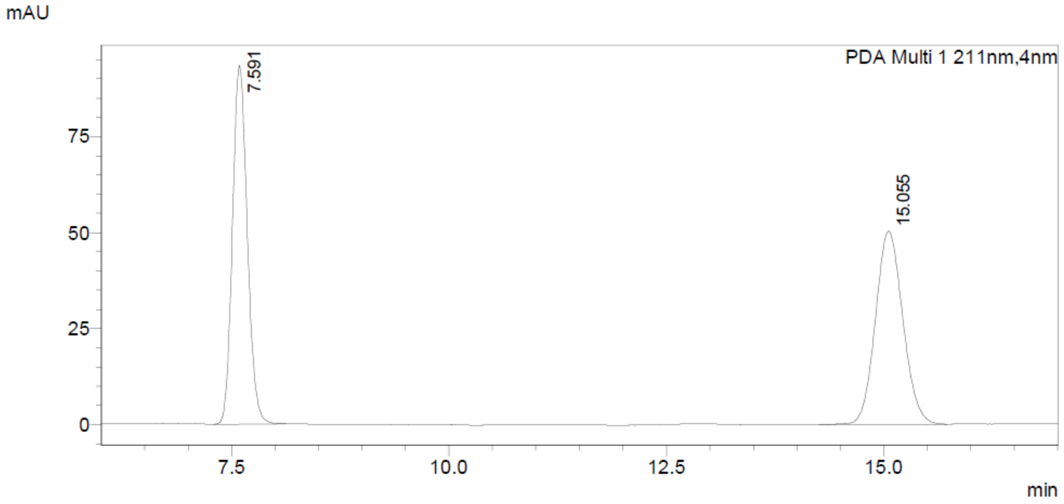

<Peak Table>

PDA Ch1 211nm

Peak#	Ret. Time	Area	Area%
1	7.591	1106875	49.955
2	15.055	1108864	50.045
Total		2215738	100.000

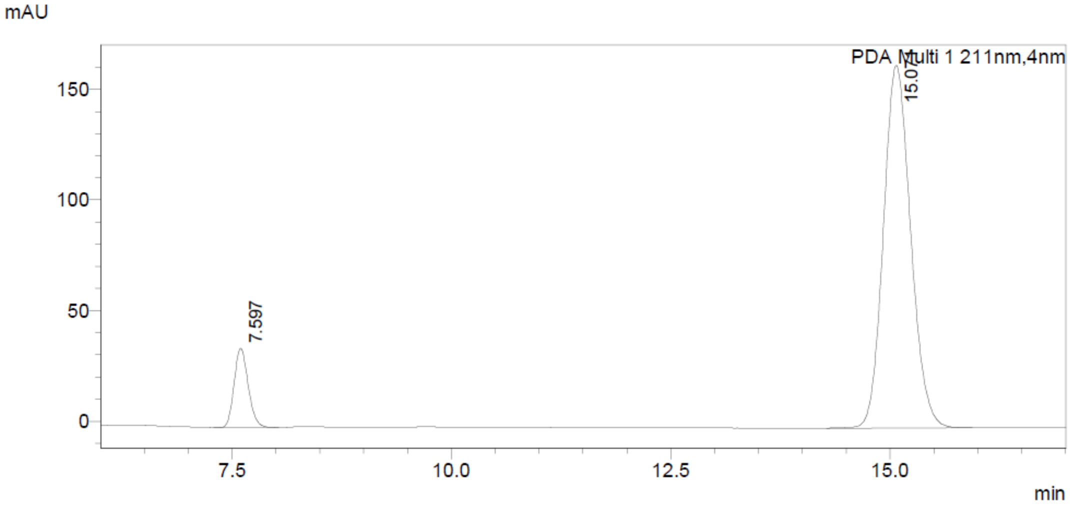

<Peak Table>

PDA Ch1 211nm

Peak#	Ret. Time	Area	Area%
1	7.597	401752	10.190
2	15.071	3540907	89.810
Total		3942659	100.000

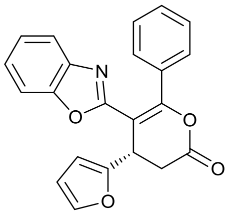

22B

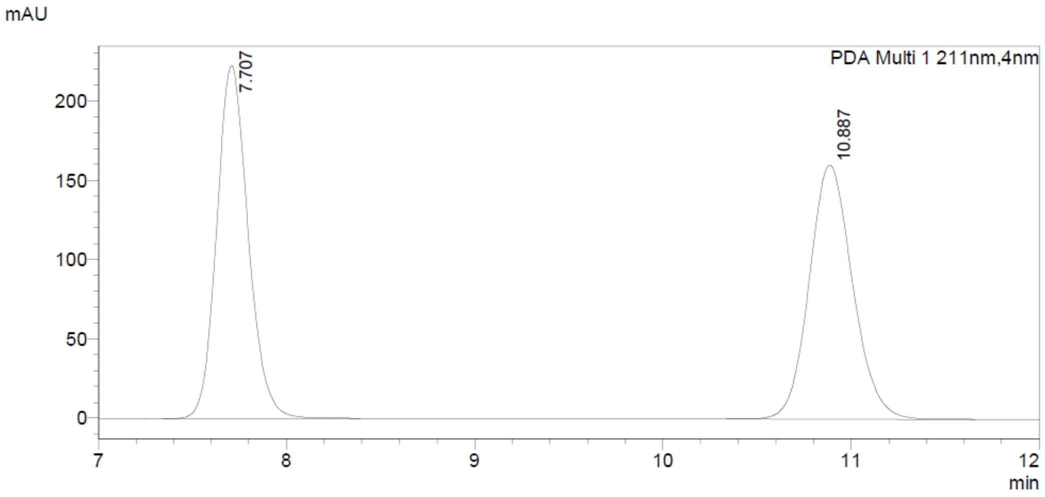

PDA Ch1 211nm

Peak#	Ret. Time	Area	Area%
1	7.707	2590247	50.173
2	10.887	2572408	49.827
Total		5162654	100.000

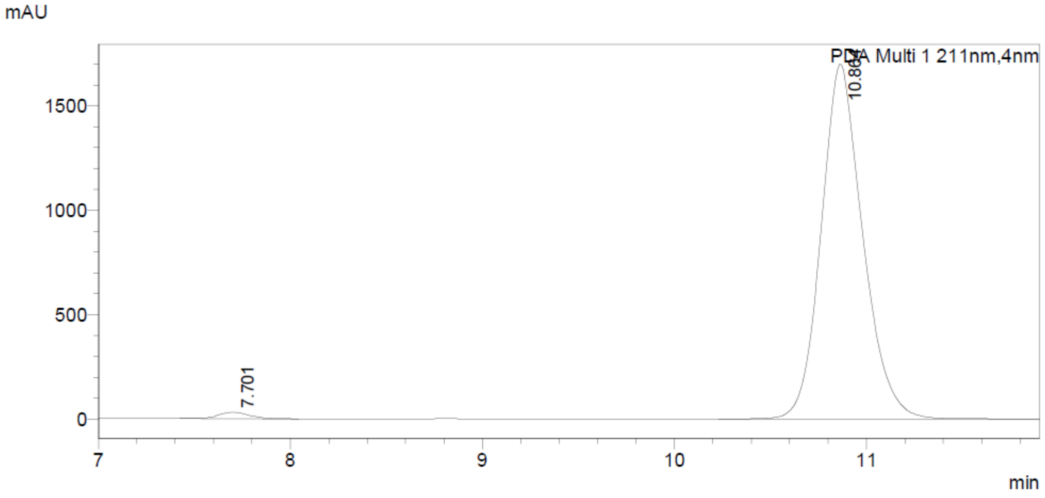

PDA Ch1 211nm

Peak#	Ret. Time	Area	Area%
1	7.701	351652	1.357
2	10.864	25561270	98.643
Total		25912923	100.000

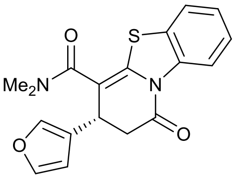

23A

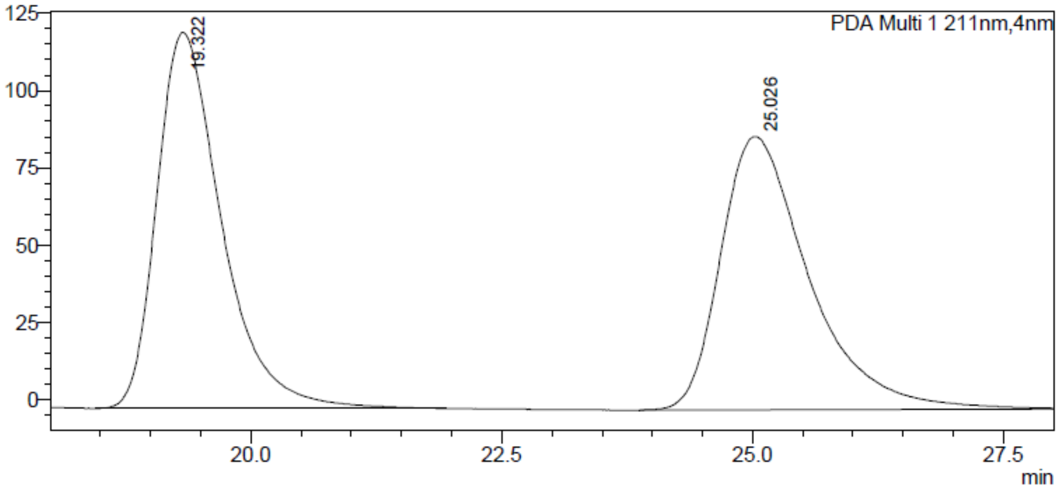

PDA Ch1 211nm

Peak#	Ret. Time	Area	Area%
1	19.415	1924853	2.297
2	24.456	81890166	97.703
Total		83815019	100.000

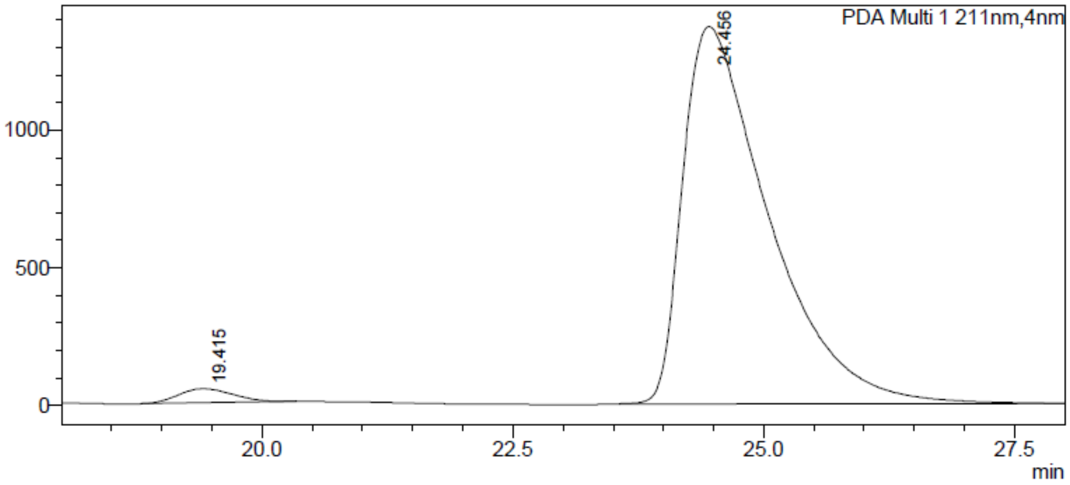

PDA Ch1 211nm

Peak#	Ret. Time	Area	Area%
1	19.322	5462439	50.223
2	25.026	5413826	49.777
Total		10876265	100.000

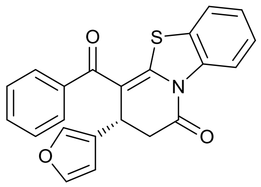

24A

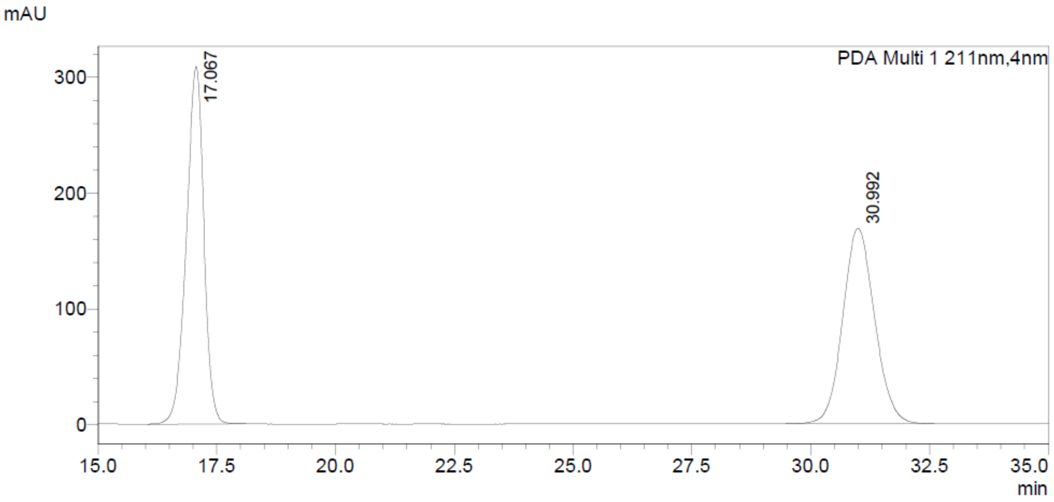

PDA Ch1 211nm

Peak#	Ret. Time	Area	Area%
1	17.067	7936308	50.170
2	30.992	7882634	49.830
Total		15818942	100.000

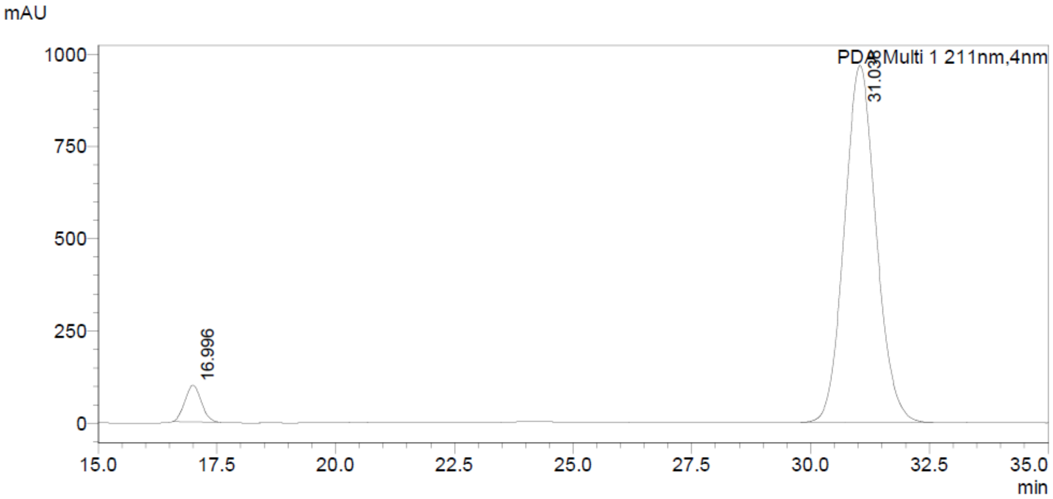

PDA Ch1 211nm

Peak#	Ret. Time	Area	Area%
1	16.996	2364041	4.989
2	31.038	45024040	95.011
Total		47388080	100.000

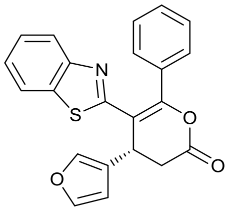

24B

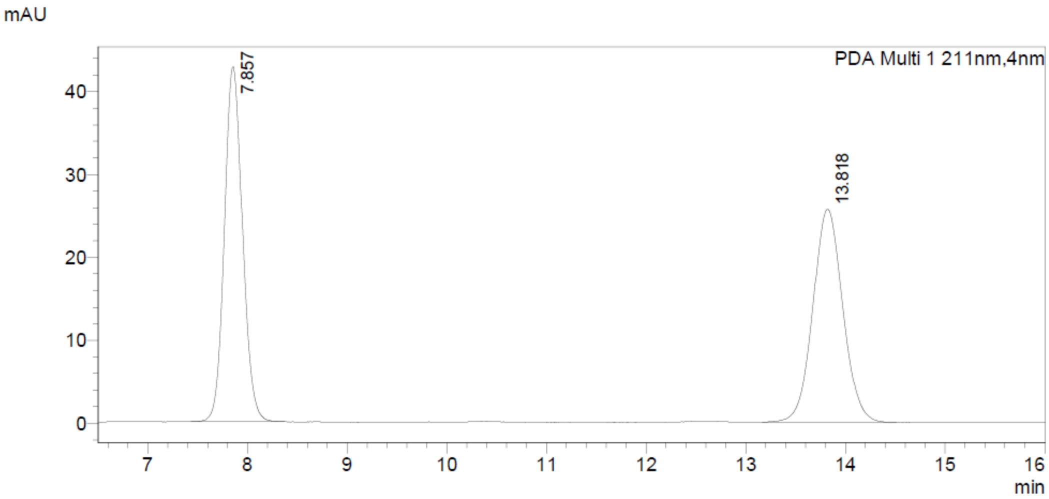

PDA Ch1 211nm

Peak#	Ret. Time	Area	Area%
1	7.857	541087	50.206
2	13.818	536640	49.794
Total		1077727	100.000

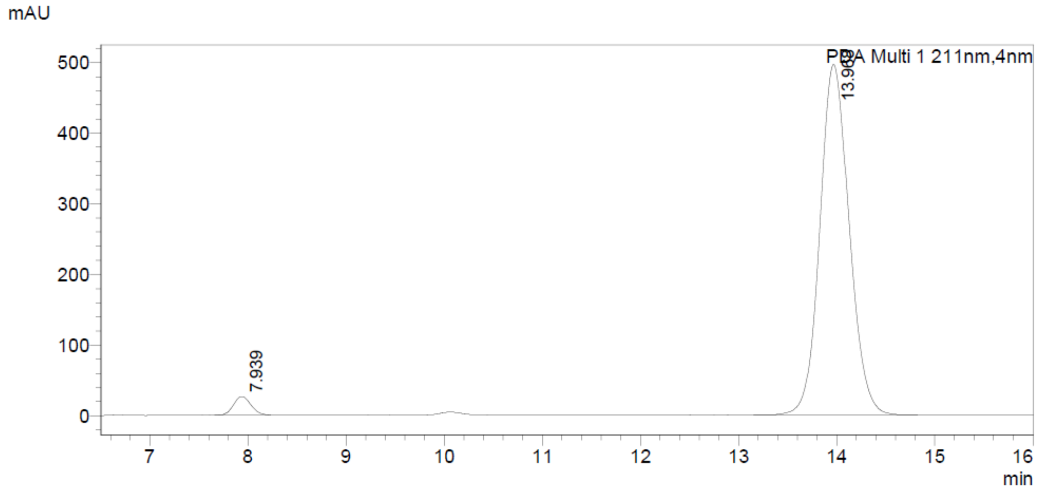

PDA Ch1 211nm

Peak#	Ret. Time	Area	Area%
1	7.939	322241	3.027
2	13.968	10323051	96.973
Total		10645292	100.000

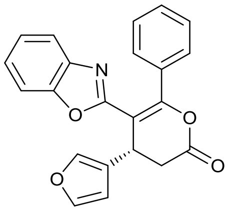

25B

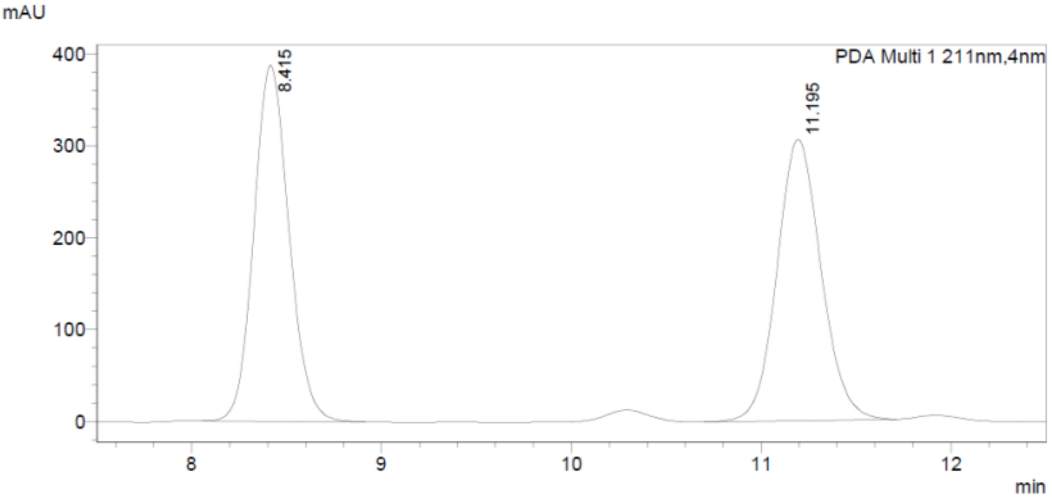

PDA Ch1 211nm

Peak#	Ret. Time	Area	Area%
1	8.415	4993734	50.169
2	11.195	4960121	49.831
Total		9953855	100.000

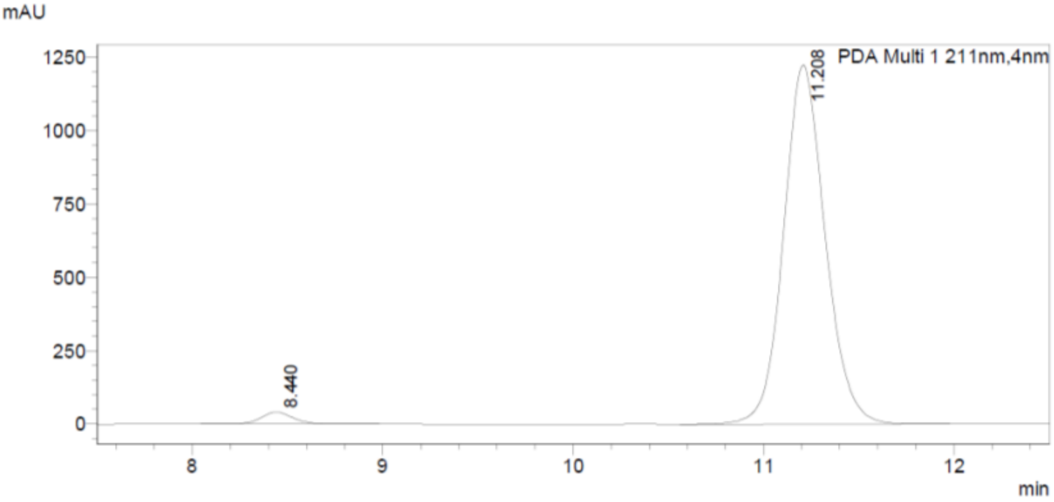

PDA Ch1 211nm

Peak#	Ret. Time	Area	Area%
1	8.440	478262	2.505
2	11.208	18612940	97.495
Total		19091202	100.000

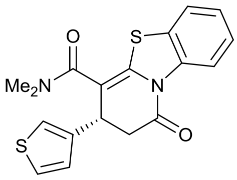

26A

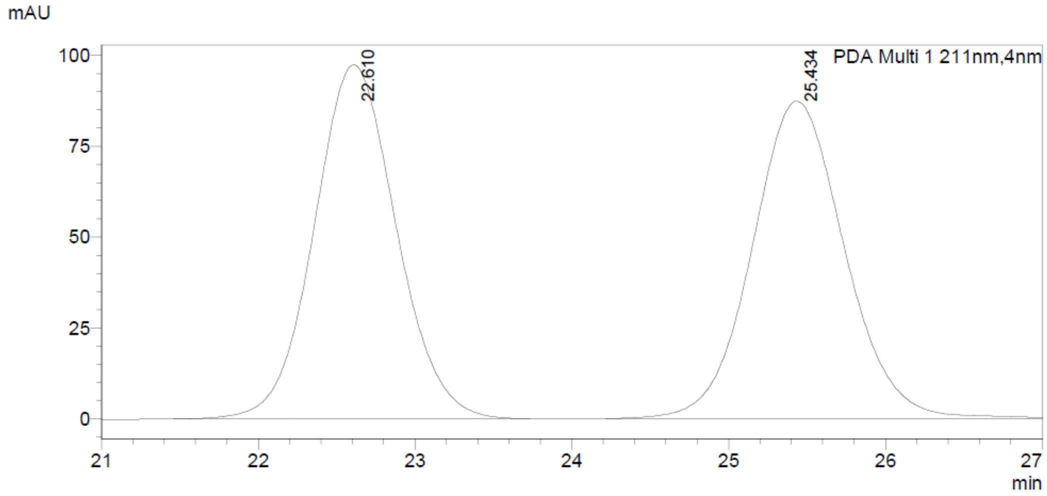

PDA Ch1 211nm

Peak#	Ret. Time	Area	Area%
1	22.610	3547207	49.732
2	25.434	3585500	50.268
Total		7132707	100.000

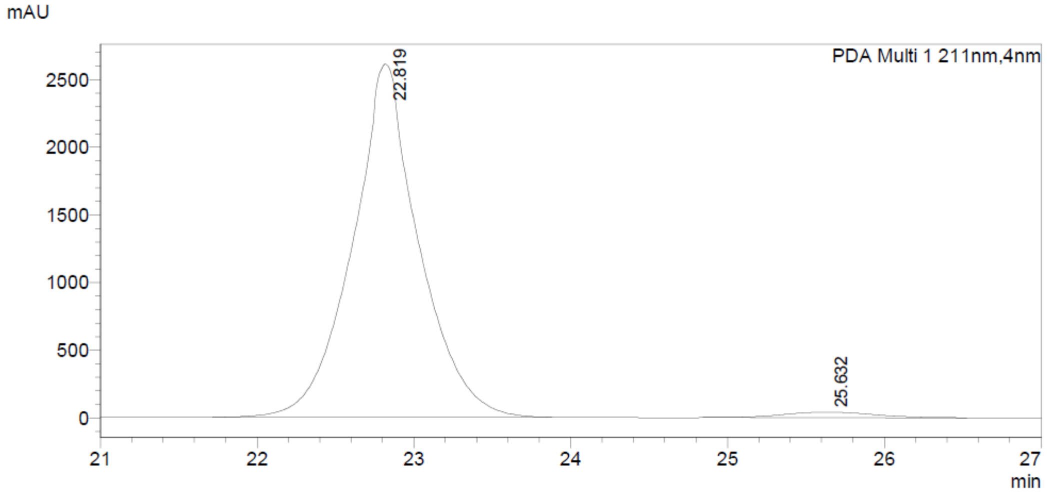

PDA Ch1 211nm

Peak#	Ret. Time	Area	Area%
1	22.819	76784394	97.923
2	25.632	1628596	2.077
Total		78412990	100.000

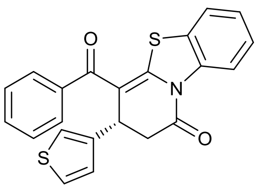

27A

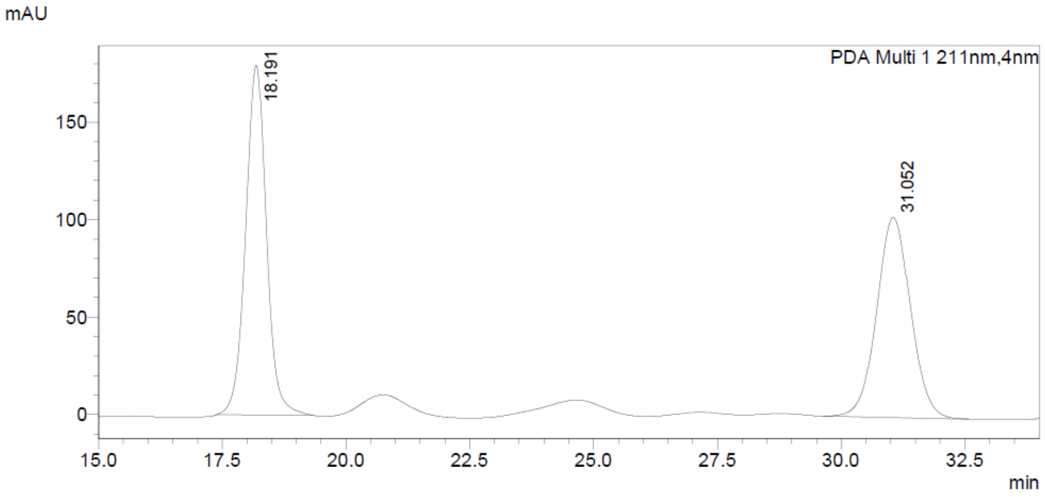

PDA Ch1 211nm

Peak#	Ret. Time	Area	Area%
1	18.191	5227117	51.628
2	31.052	4897366	48.372
Total		10124483	100.000

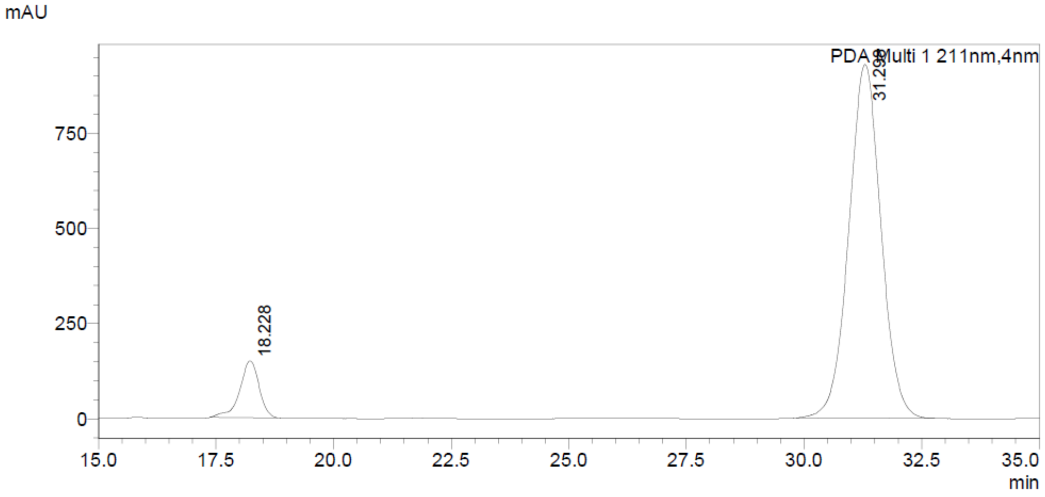

PDA Ch1 211nm

Peak#	Ret. Time	Area	Area%
1	18.228	4266730	8.800
2	31.298	44221091	91.200
Total		48487821	100.000

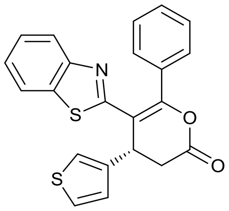

27B

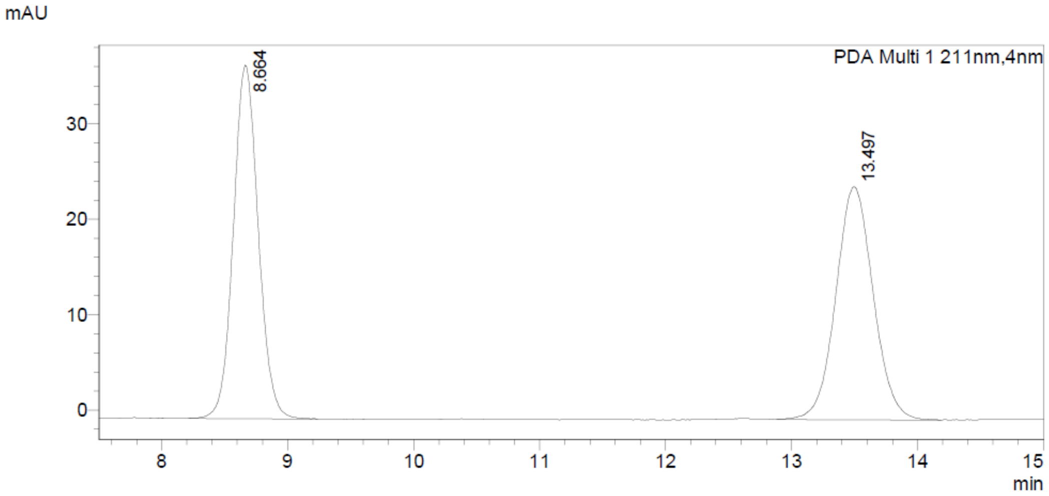

PDA Ch1 211nm

Peak#	Ret. Time	Area	Area%
1	8.664	508900	50.283
2	13.497	503177	49.717
Total		1012077	100.000

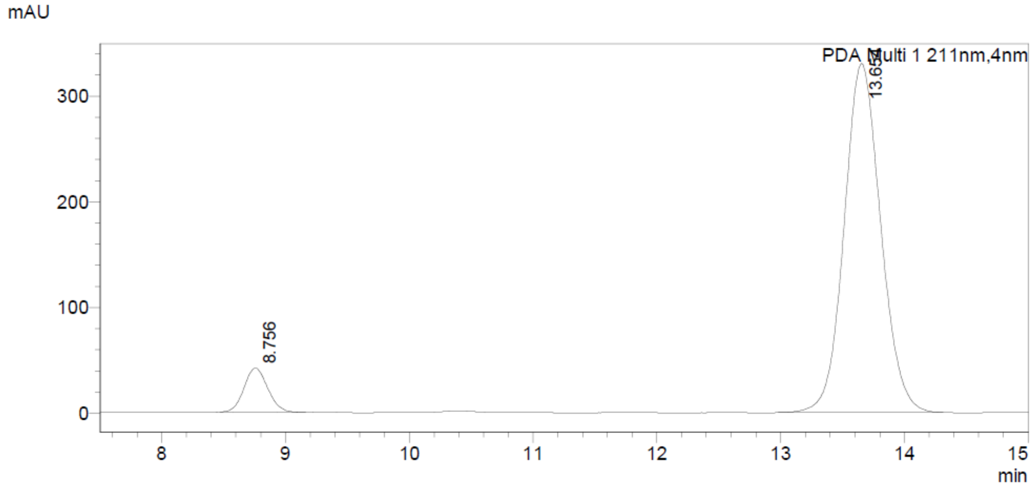

PDA Ch1 211nm

Peak#	Ret. Time	Area	Area%
1	8.756	557827	7.580
2	13.654	6801269	92.420
Total		7359096	100.000

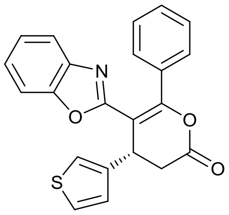

28B

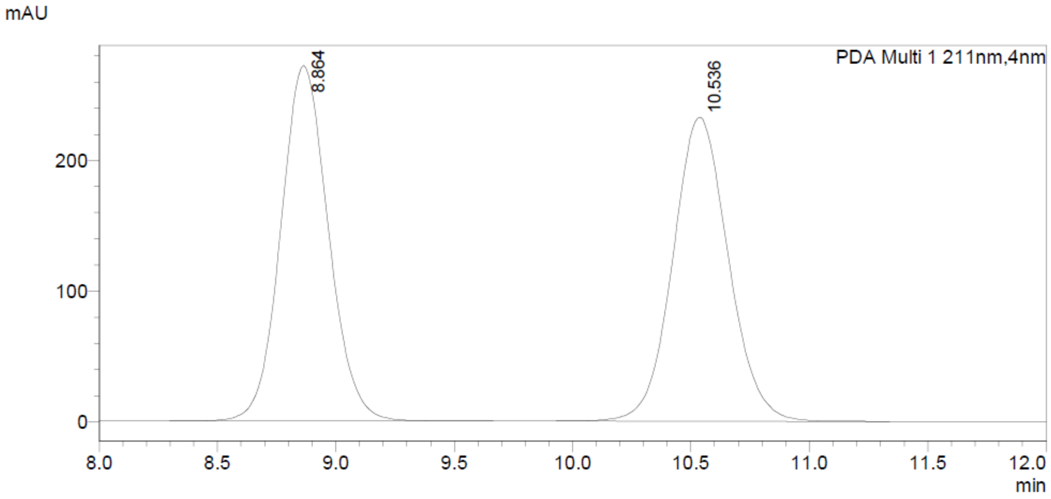

PDA Ch1 211nm

Peak#	Ret. Time	Area	Area%
1	8.864	3810217	50.089
2	10.536	3796652	49.911
Total		7606869	100.000

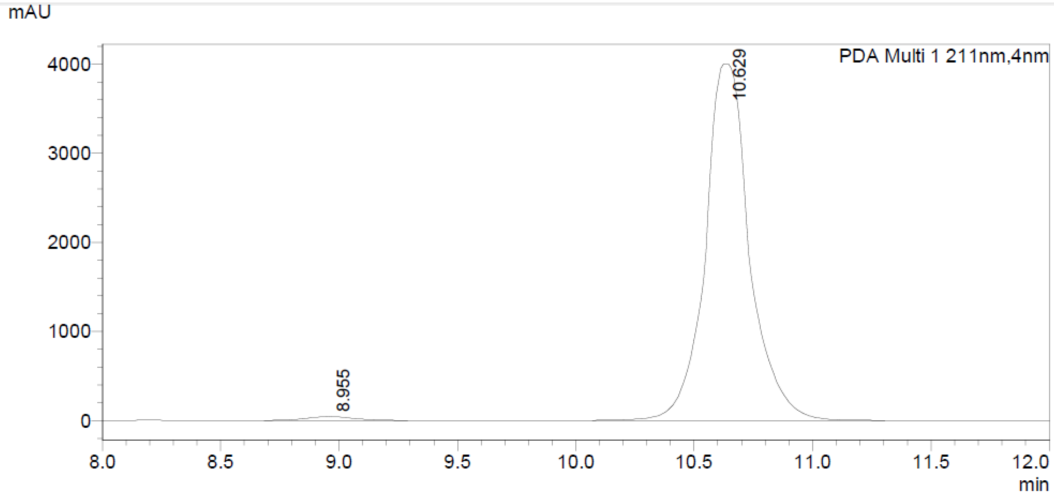

PDA Ch1 211nm

Peak#	Ret. Time	Area	Area%
1	8.955	571794	1.085
2	10.629	52138319	98.915
Total		52710113	100.000

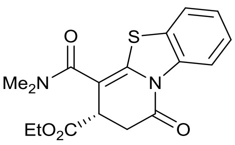

29A

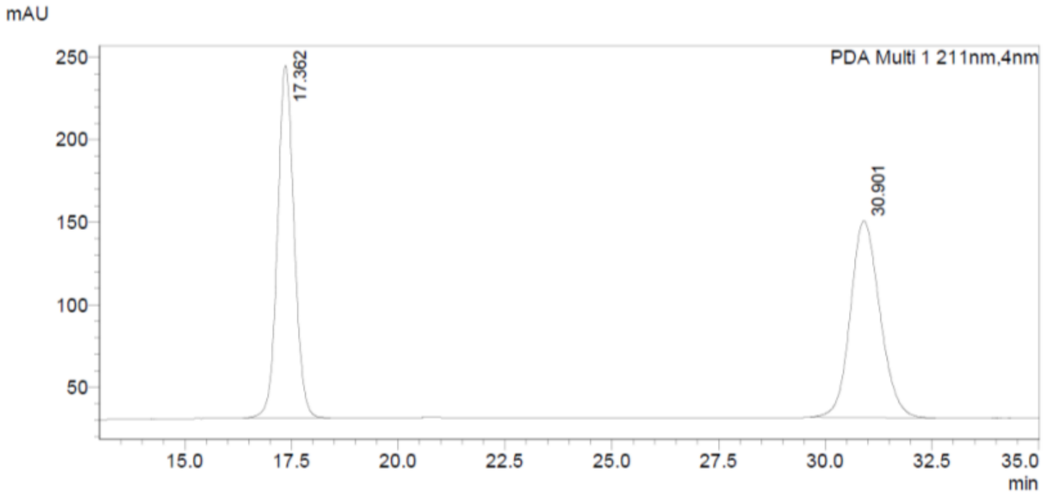

PDA Ch1 211nm

Peak#	Ret. Time	Area	Area%
1	17.362	5770862	50.182
2	30.901	5729018	49.818
Total		11499881	100.000

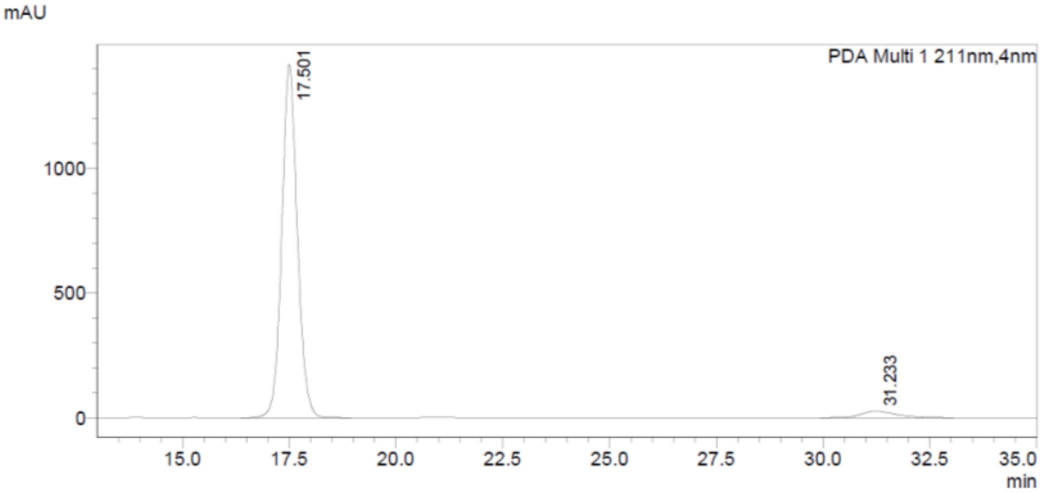

PDA Ch1 211nm

Peak#	Ret. Time	Area	Area%
1	17.501	35744796	95.988
2	31.233	1493993	4.012
Total		37238789	100.000

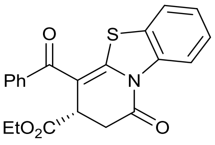

30A

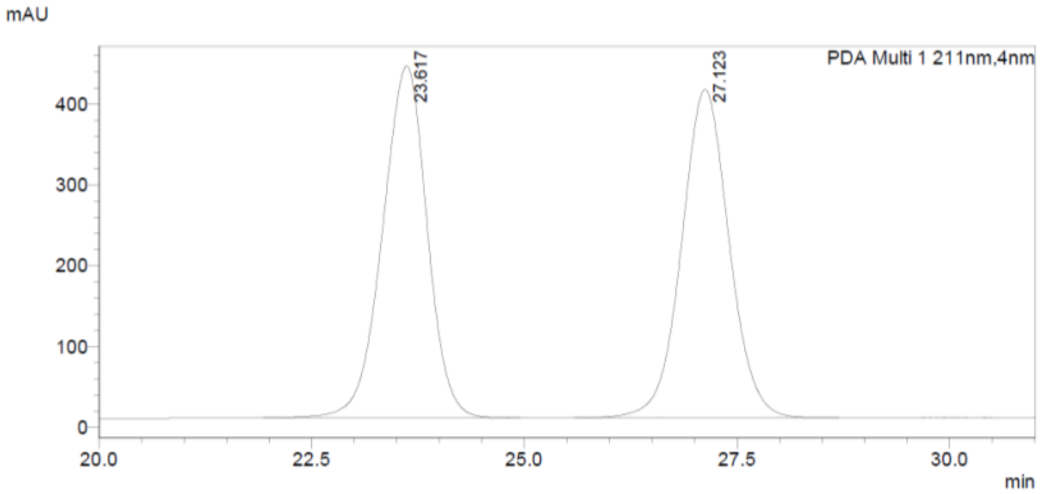

PDA Ch1 211nm

Peak#	Ret. Time	Area	Area%
1	23.617	15971249	50.181
2	27.123	15856139	49.819
Total		31827388	100.000

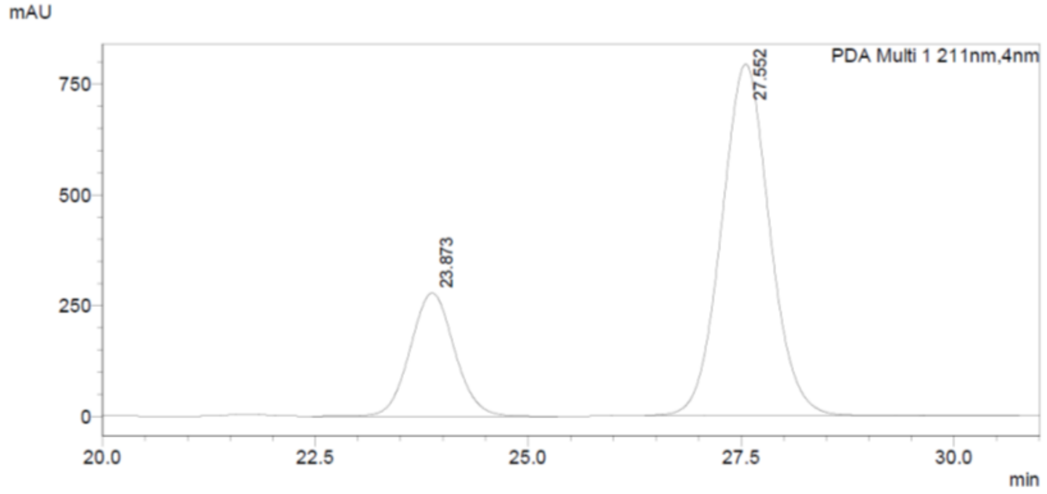

PDA Ch1 211nm

Peak#	Ret. Time	Area	Area%
1	23.873	10064871	24.361
2	27.552	31250872	75.639
Total		41315743	100.000

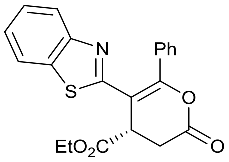

30B

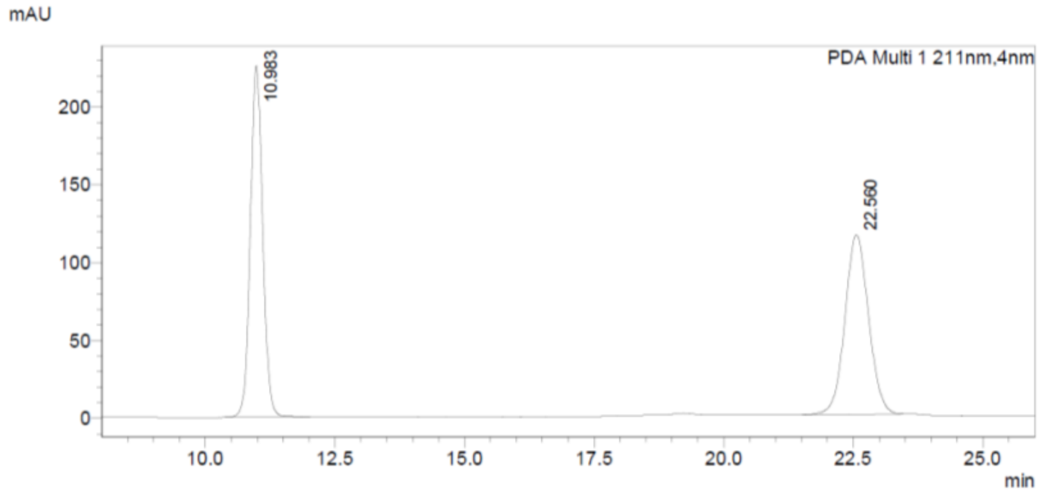

PDA Ch1 211nm

Peak#	Ret. Time	Area	Area%
1	10.983	3748953	50.595
2	22.560	3660714	49.405
Total		7409667	100.000

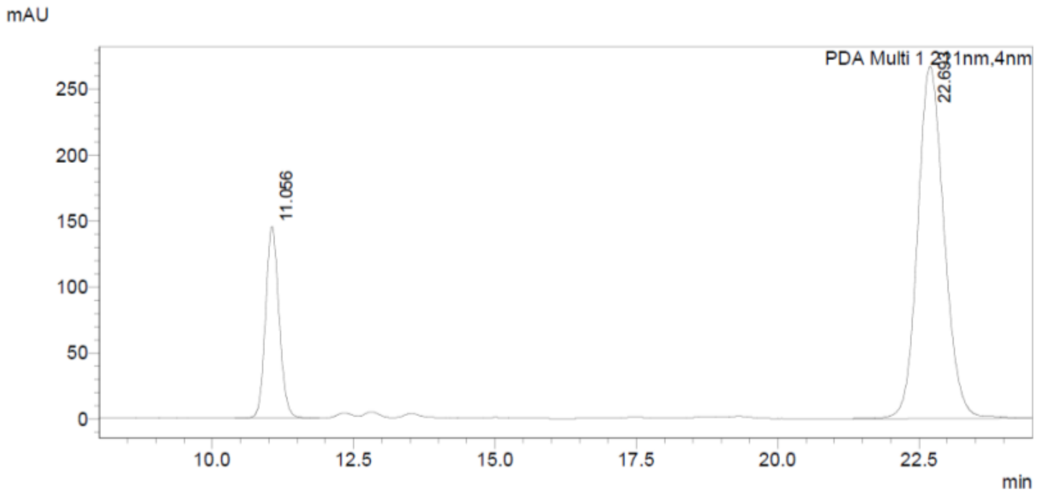

PDA Ch1 211nm

Peak#	Ret. Time	Area	Area%
1	11.056	2431394	21.863
2	22.693	8689699	78.137
Total		11121092	100.000

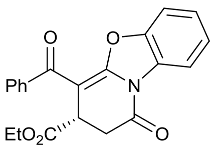

31A

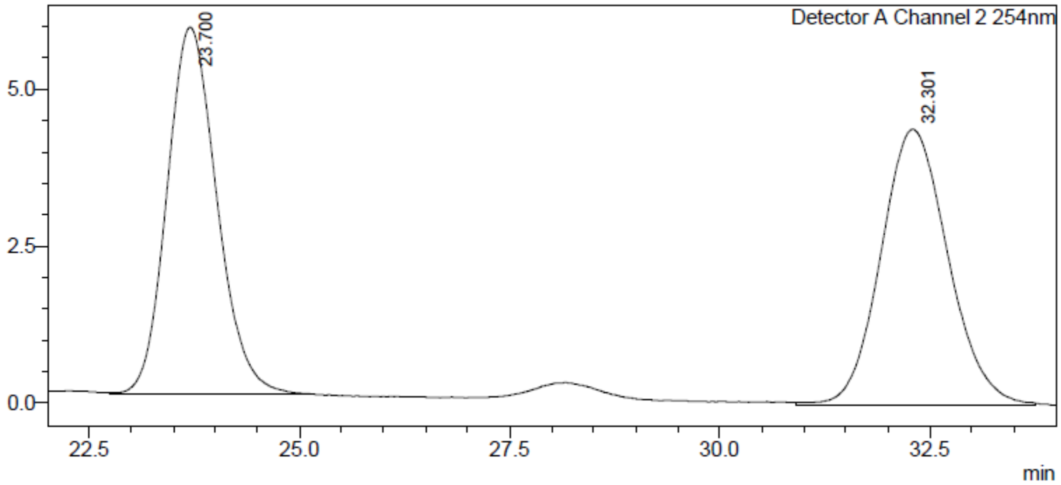

Detector A Channel 1 211nm

Peak#	Ret. Time	Area%
1	23.695	50.010
2	32.338	49.990
Total		100.000

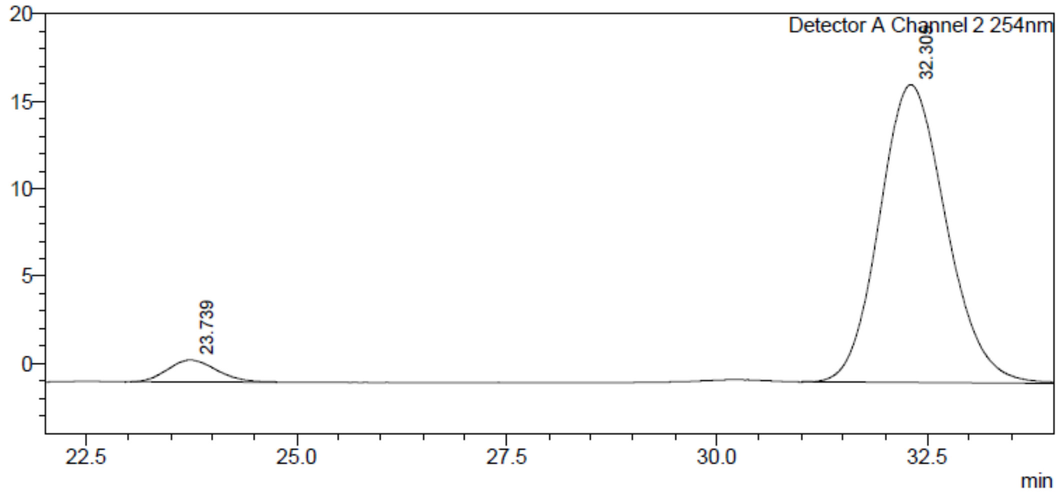

Detector A Channel 2 254nm

Peak#	Ret. Time	Area%
1	23.739	5.116
2	32.305	94.884
Total		100.000

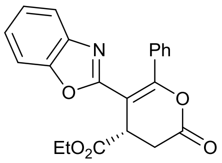

31B

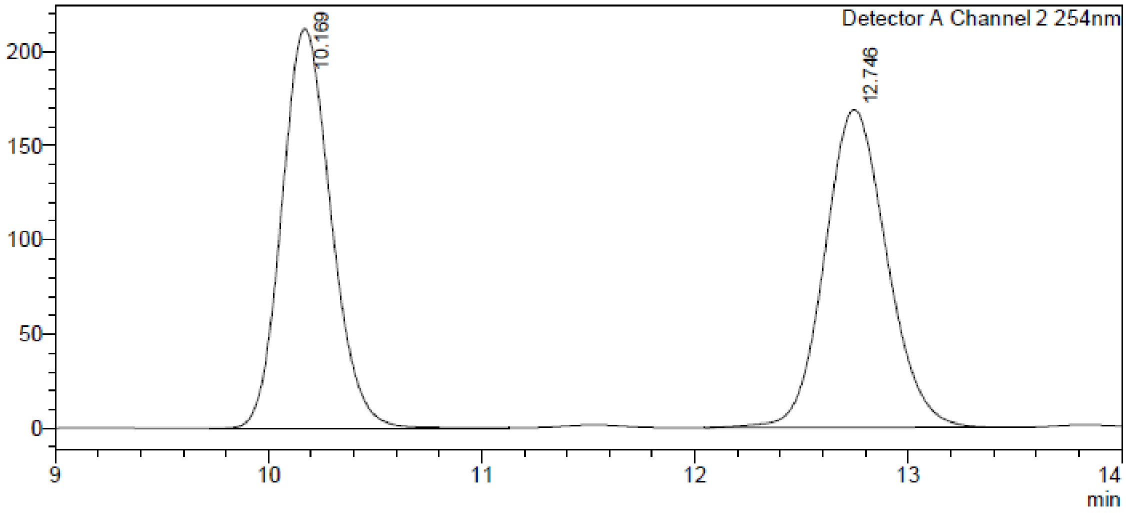

Detector A Channel 2 254nm

Peak#	Ret. Time	Area%
1	10.169	49.891
2	12.746	50.109
Total		100.000

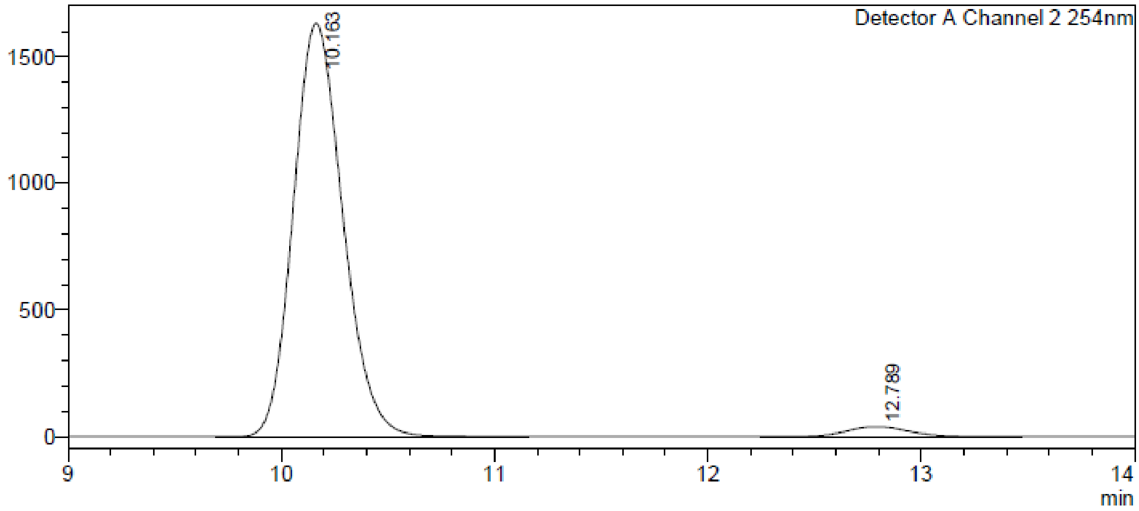

Detector A Channel 2 254nm

Peak#	Ret. Time	Area%
1	10.163	96.964
2	12.789	3.036
Total		100.000

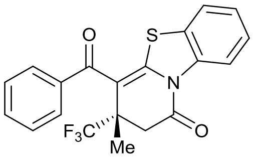

35

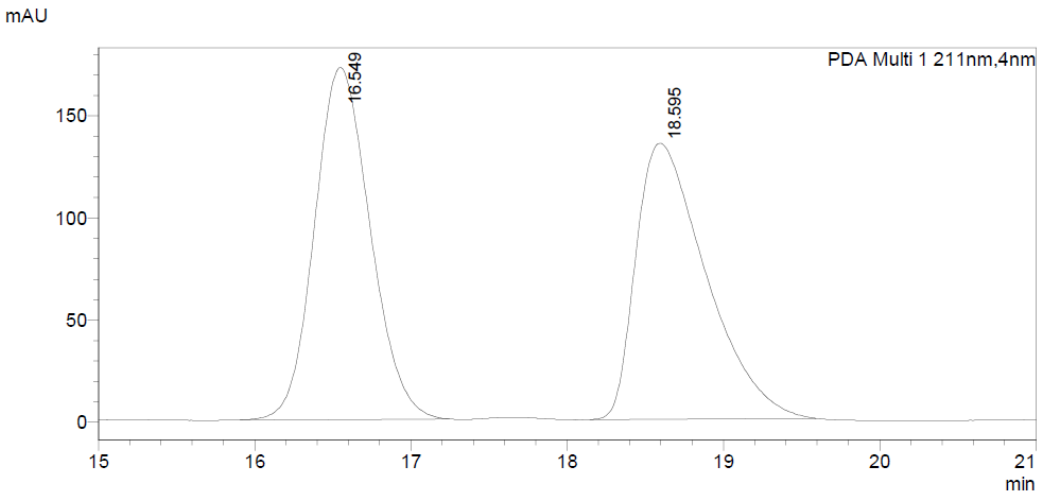

PDA Ch1 211nm

Peak#	Ret. Time	Area	Area%
1	16.549	4269242	50.342
2	18.595	4211296	49.658
Total		8480539	100.000

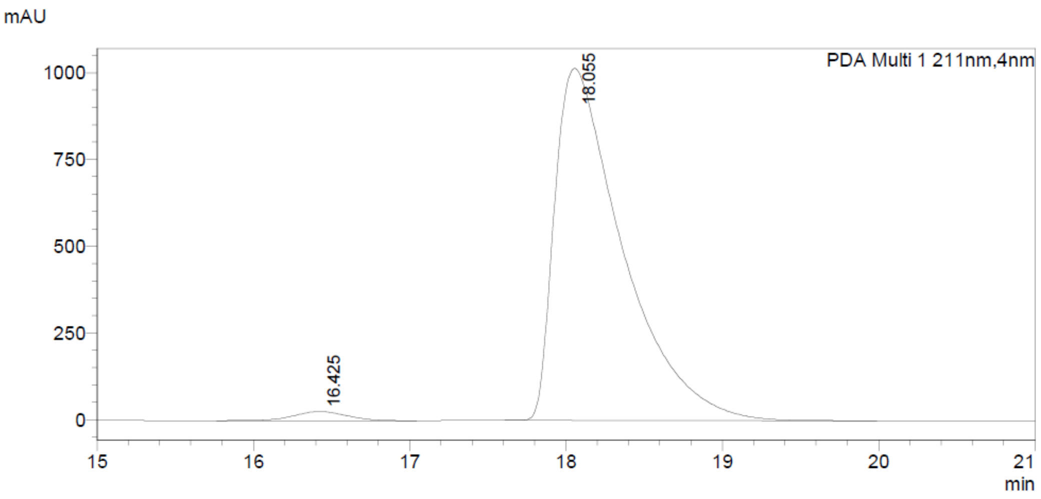

PDA Ch1 211nm

Peak#	Ret. Time	Area	Area%
1	16.425	611034	1.948
2	18.055	30756317	98.052
Total		31367351	100.000

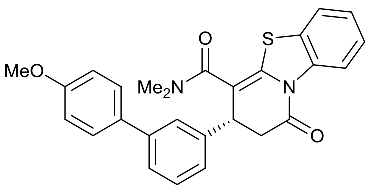

37

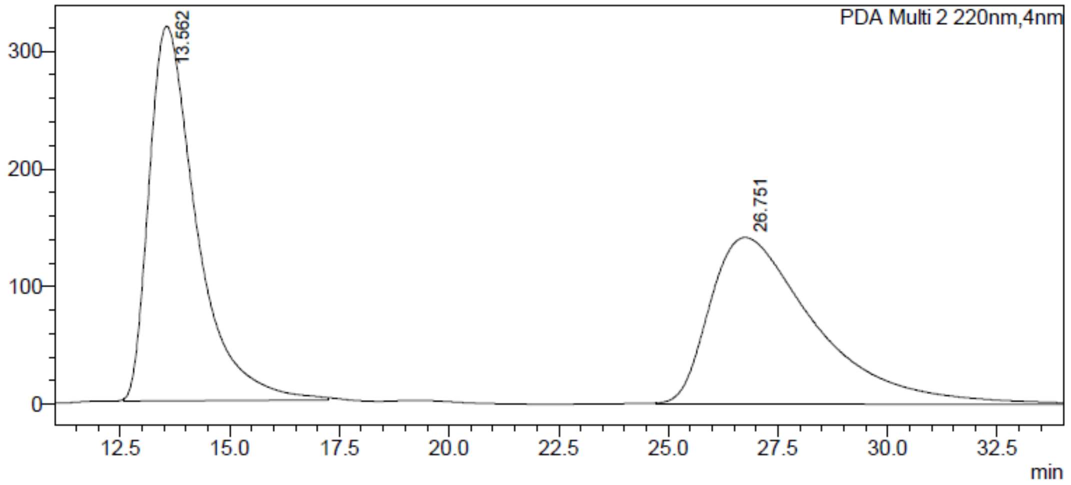

PDA Ch2 220nm

Peak#	Ret. Time	Area%
1	13.562	50.229
2	26.751	49.771
Total		100.000

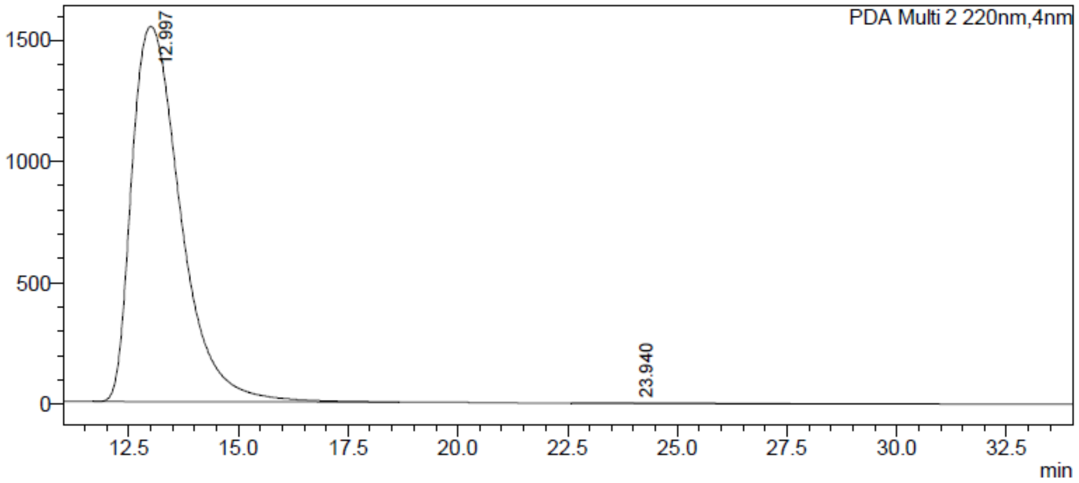

PDA Ch2 220nm

Peak#	Ret. Time	Area%
1	12.997	99.841
2	23.940	0.159
Total		100.000

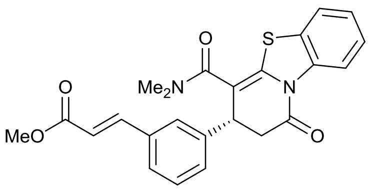

38

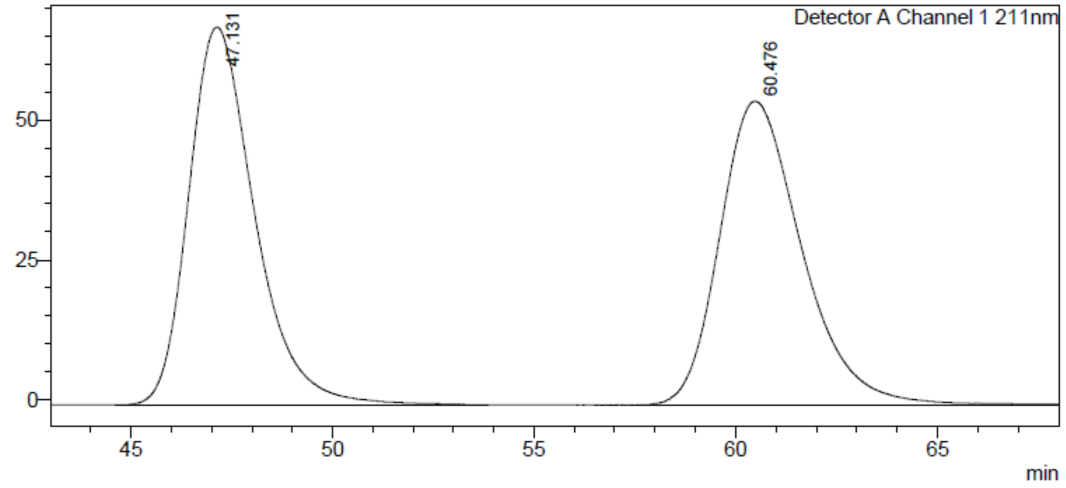

Detector A Channel 1 211nm

Peak#	Ret. Time	Area%
1	47.131	49.924
2	60.476	50.076
Total		100.000

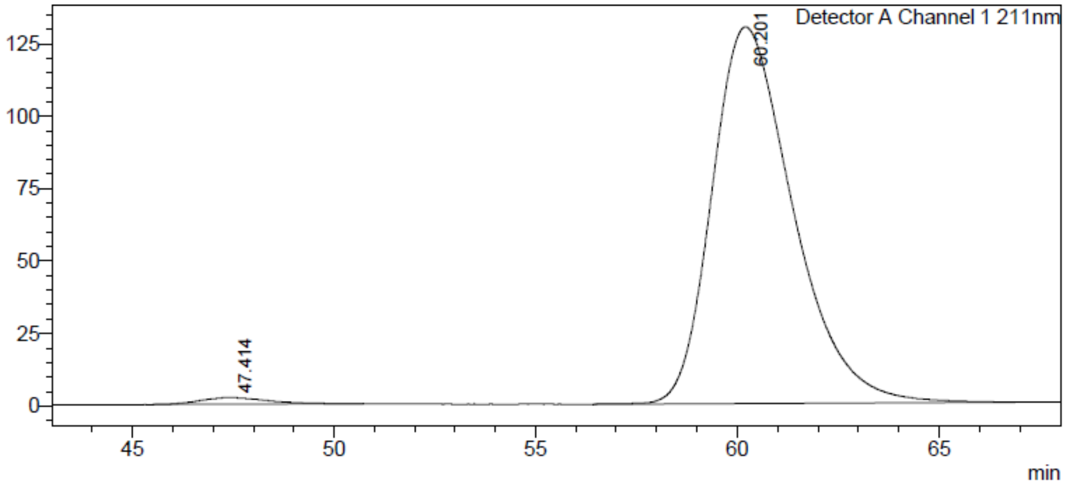

Detector A Channel 1 211nm

Peak#	Ret. Time	Area%
1	47.414	1.478
2	60.201	98.522
Total		100.000

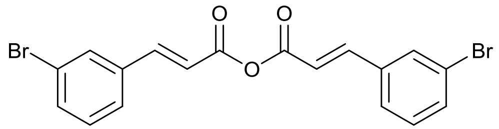

S10
^{1}H, 500 MHz, $CDCl_3$

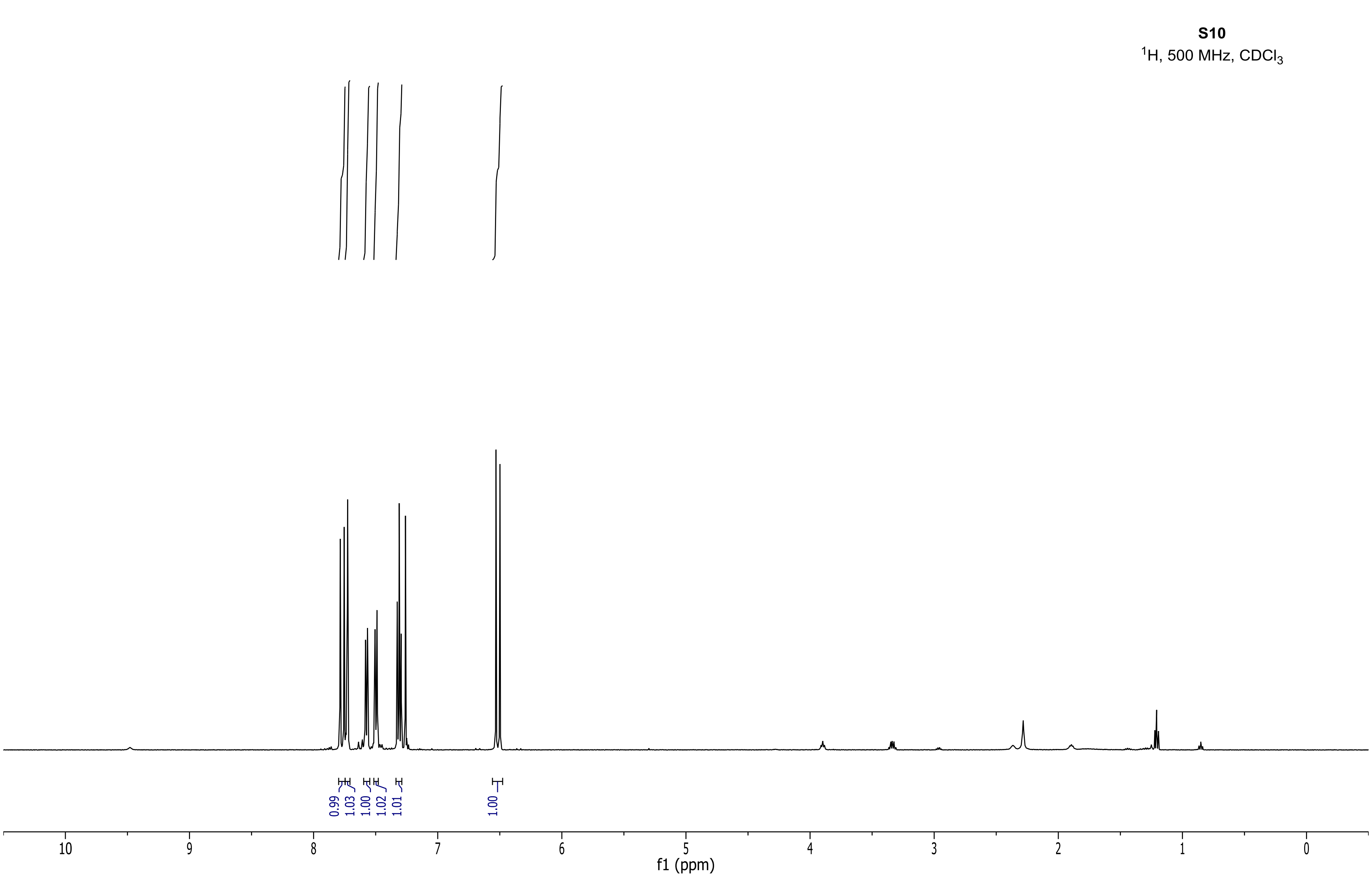

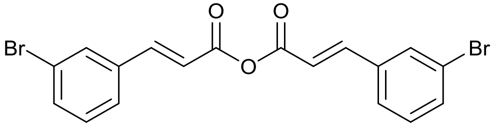

S10

^{13}C, 75 MHz, $CDCl_3$

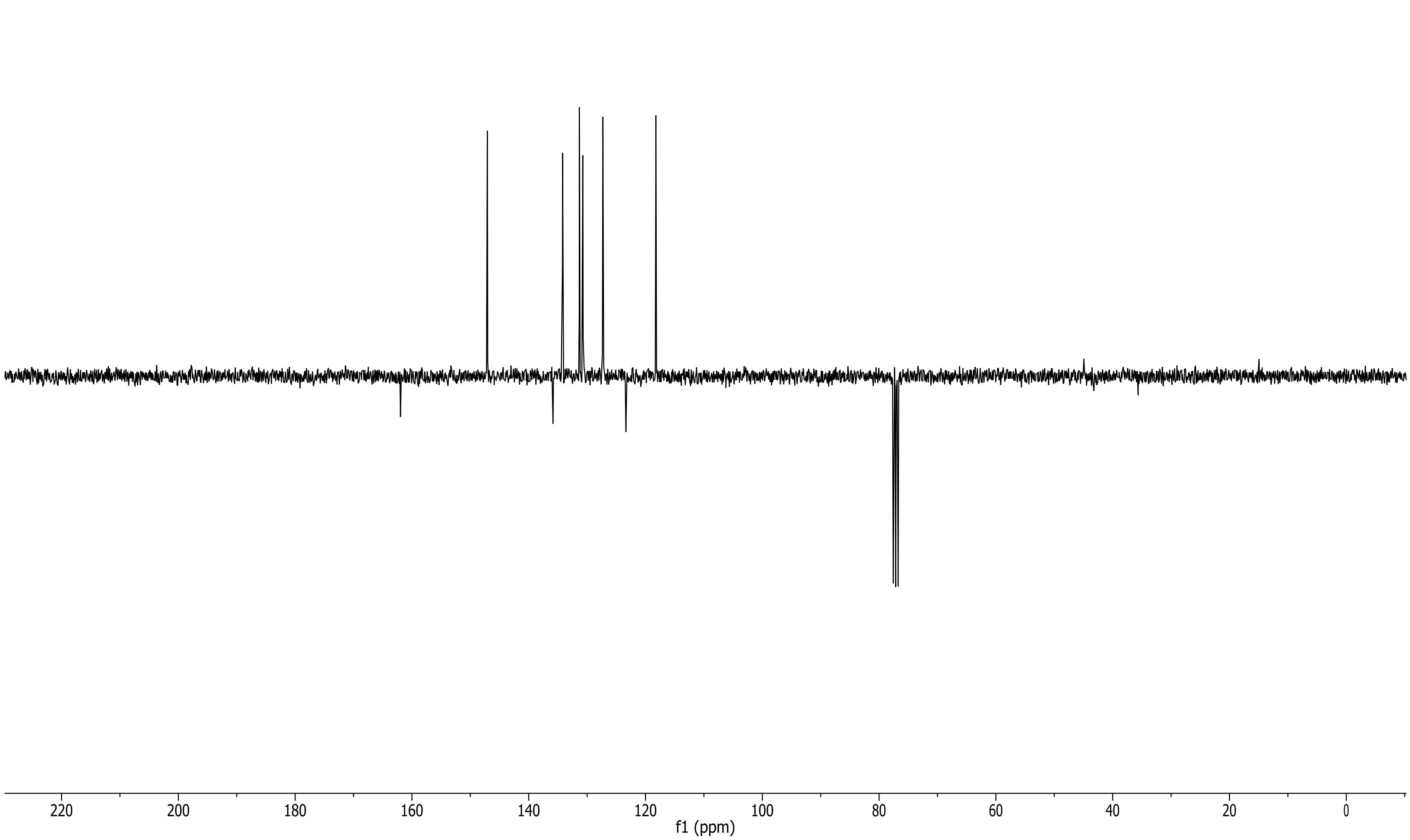

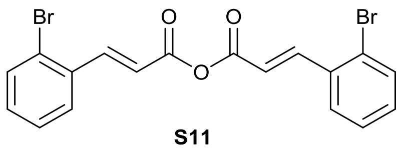

S11

^{1}H, 500 MHz, $CDCl_3$

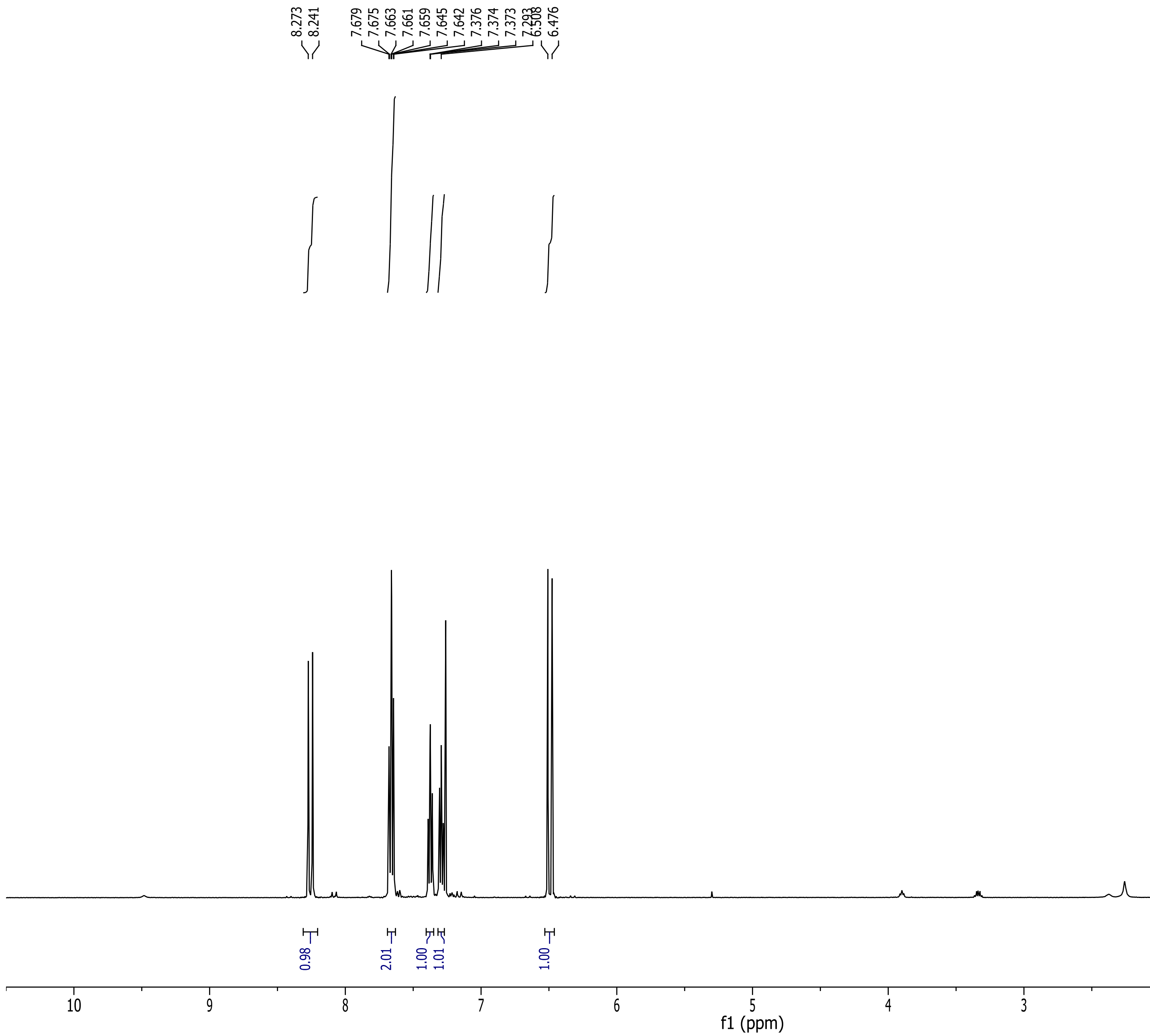

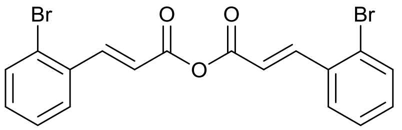

S11

^{13}C, 125 MHz, $CDCl_3$

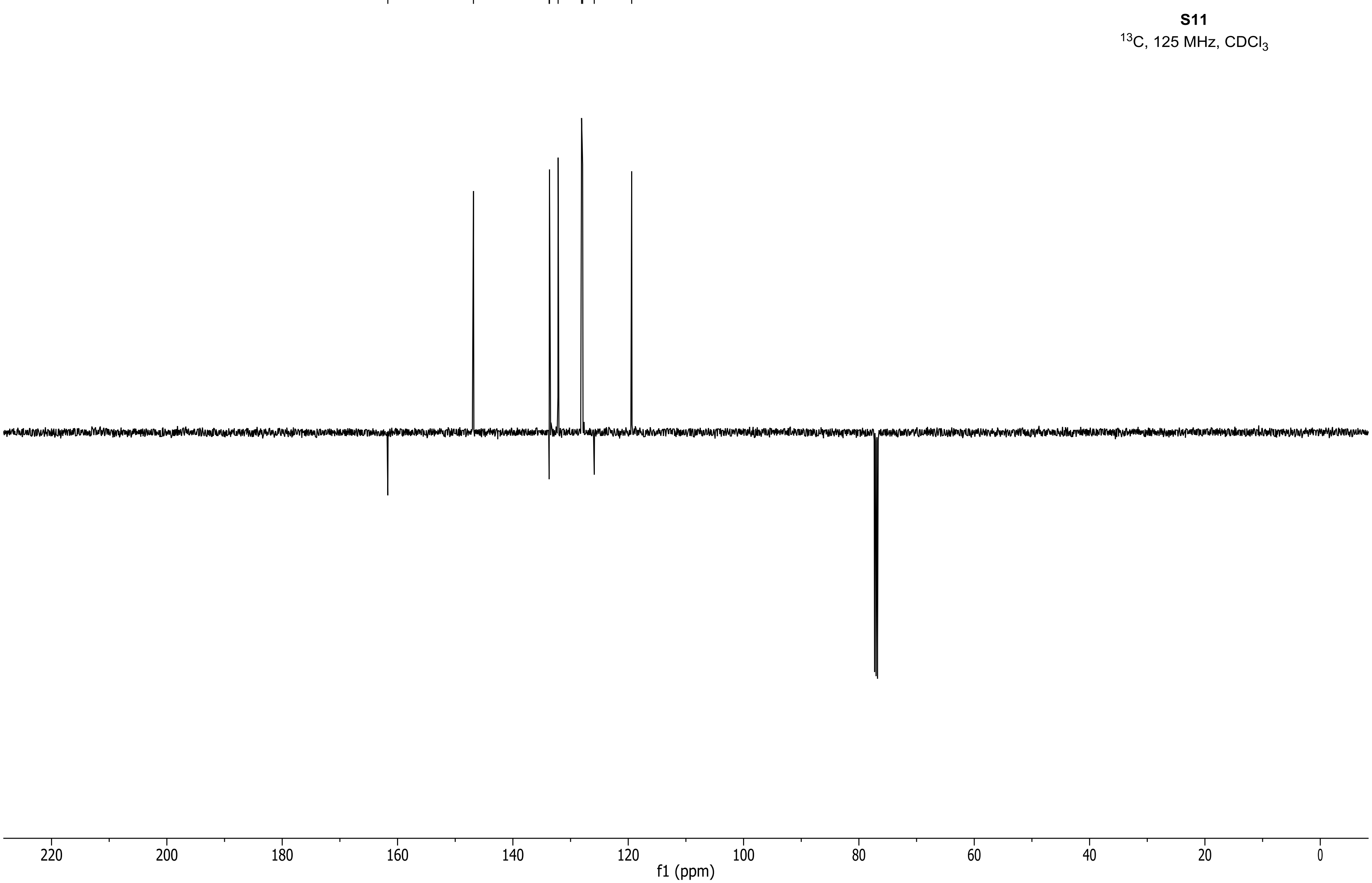

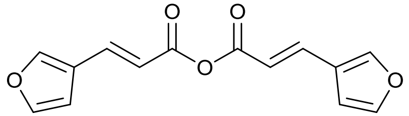

S14

^{1}H, 500 MHz, $CDCl_3$

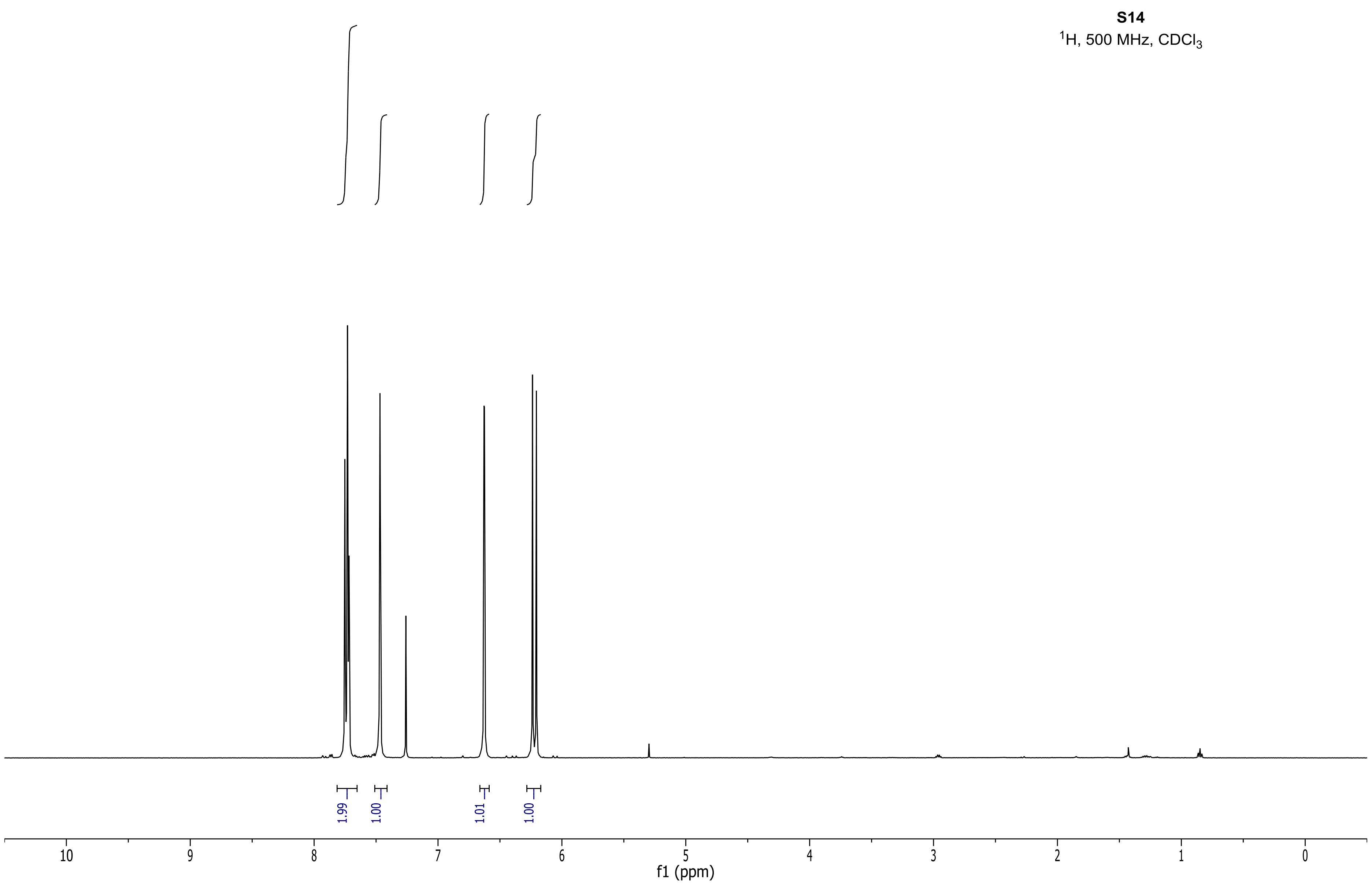

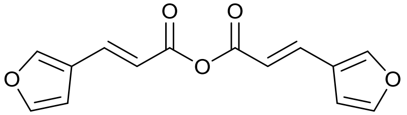

S14
^{13}C, 75 MHz, $CDCl_3$

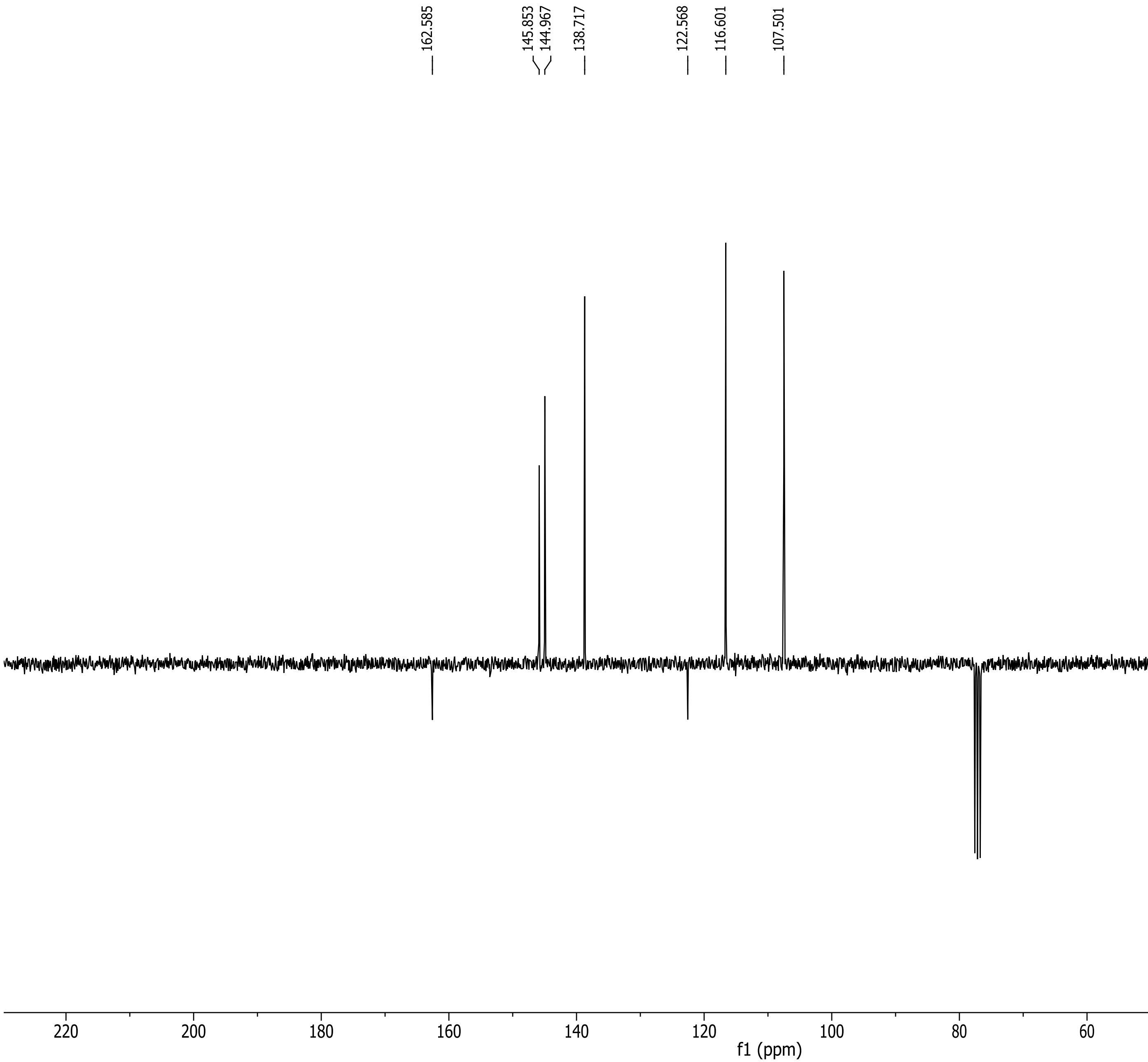

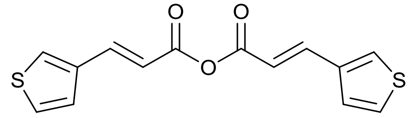

S15
^{1}H, 500 MHz, $CDCl_3$

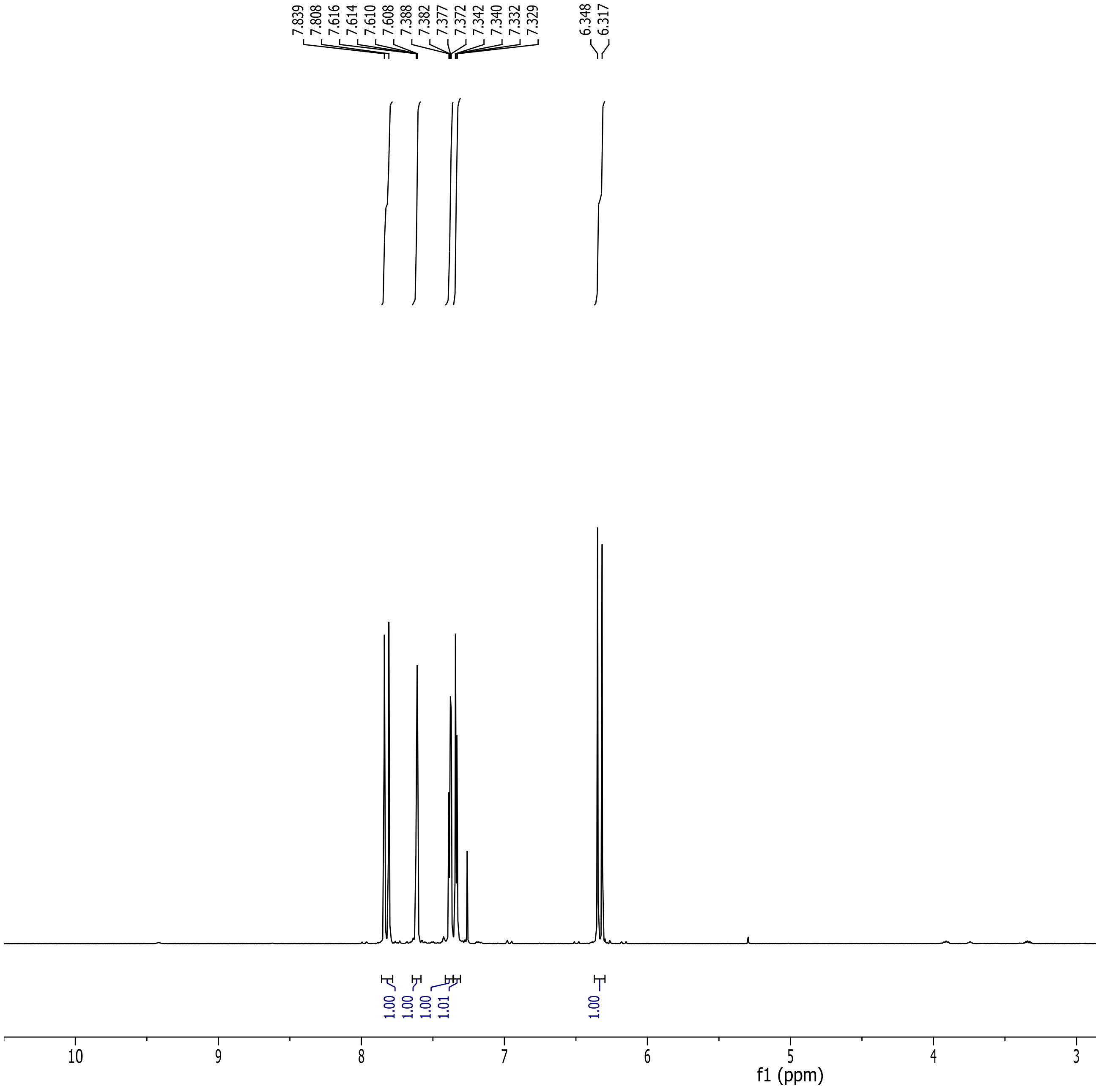

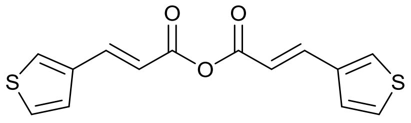

S15
^{13}C, 125 MHz, $CDCl_3$

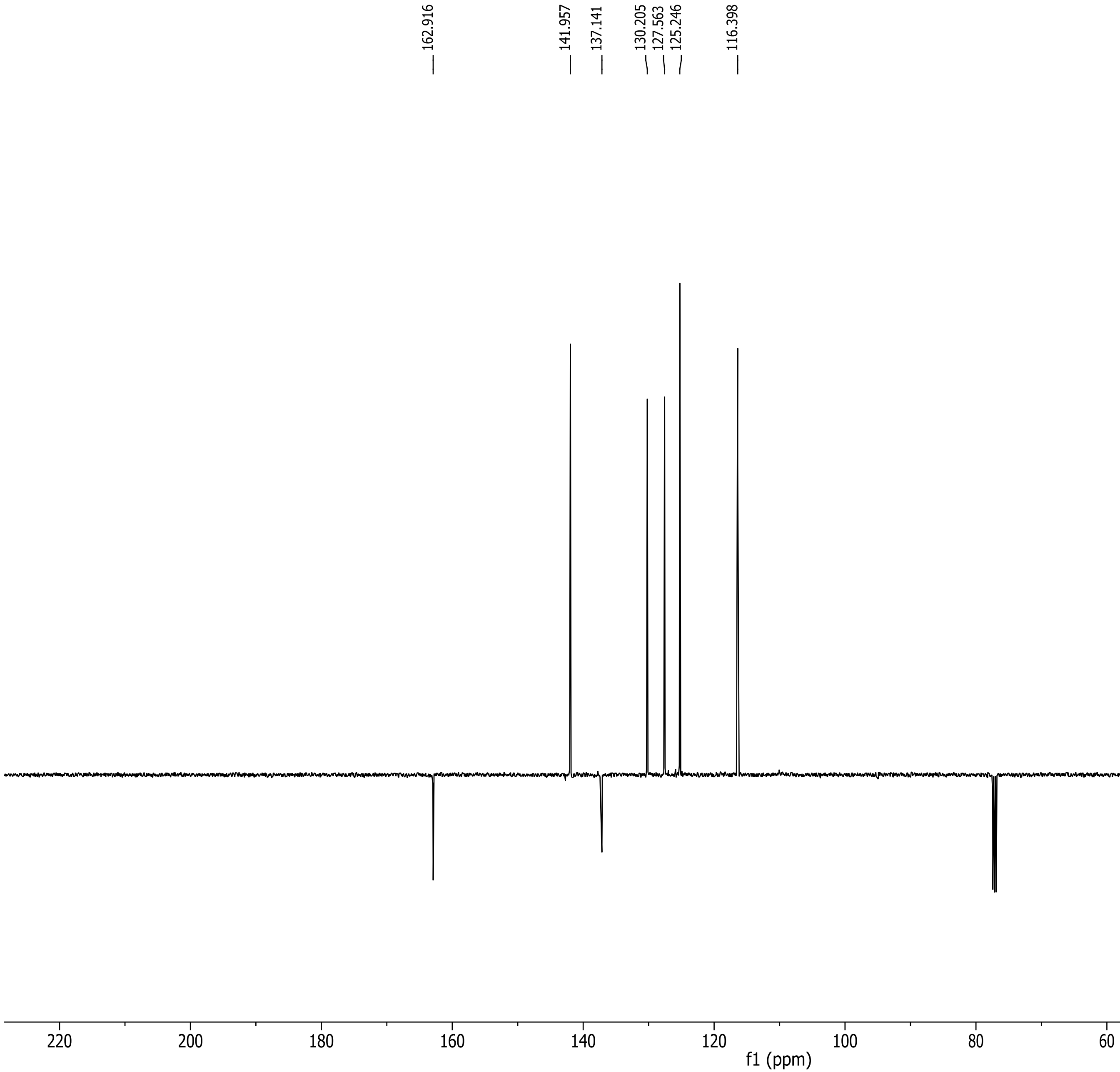

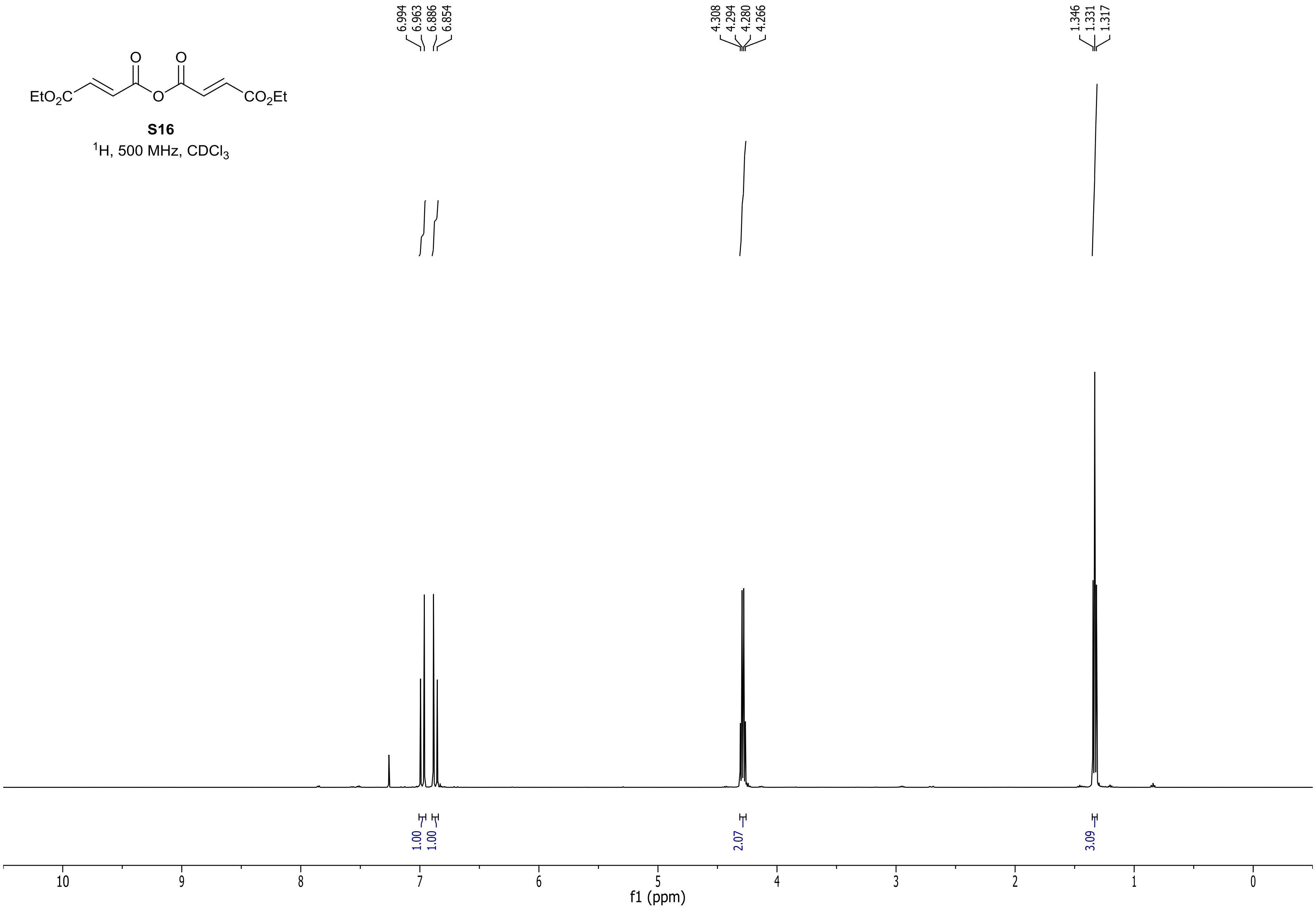
EtO2C
O
O
O
CO2Et
S16
1H, 500 MHz, CDCl3
6.994
6.963
6.886
6.854
4.308
4.294
4.280
4.266
1.346
1.331
1.317
1.00
1.00
2.07
3.09
10
9
8
7
6
5
4
3
2
1
0
f1 (ppm)

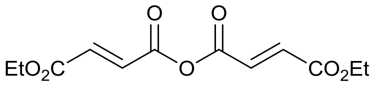

S16
^{13}C, 75 MHz, $CDCl_3$

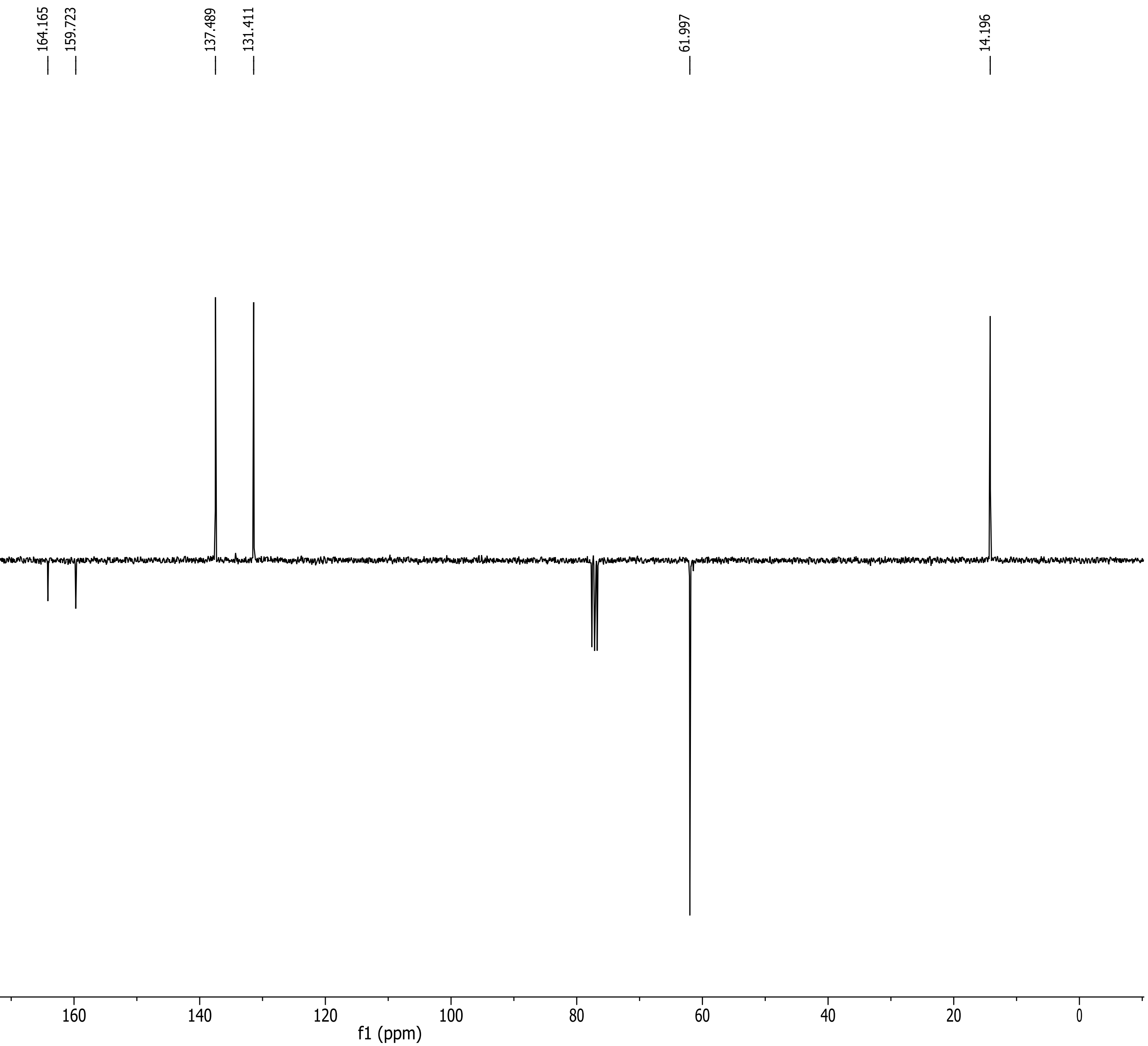

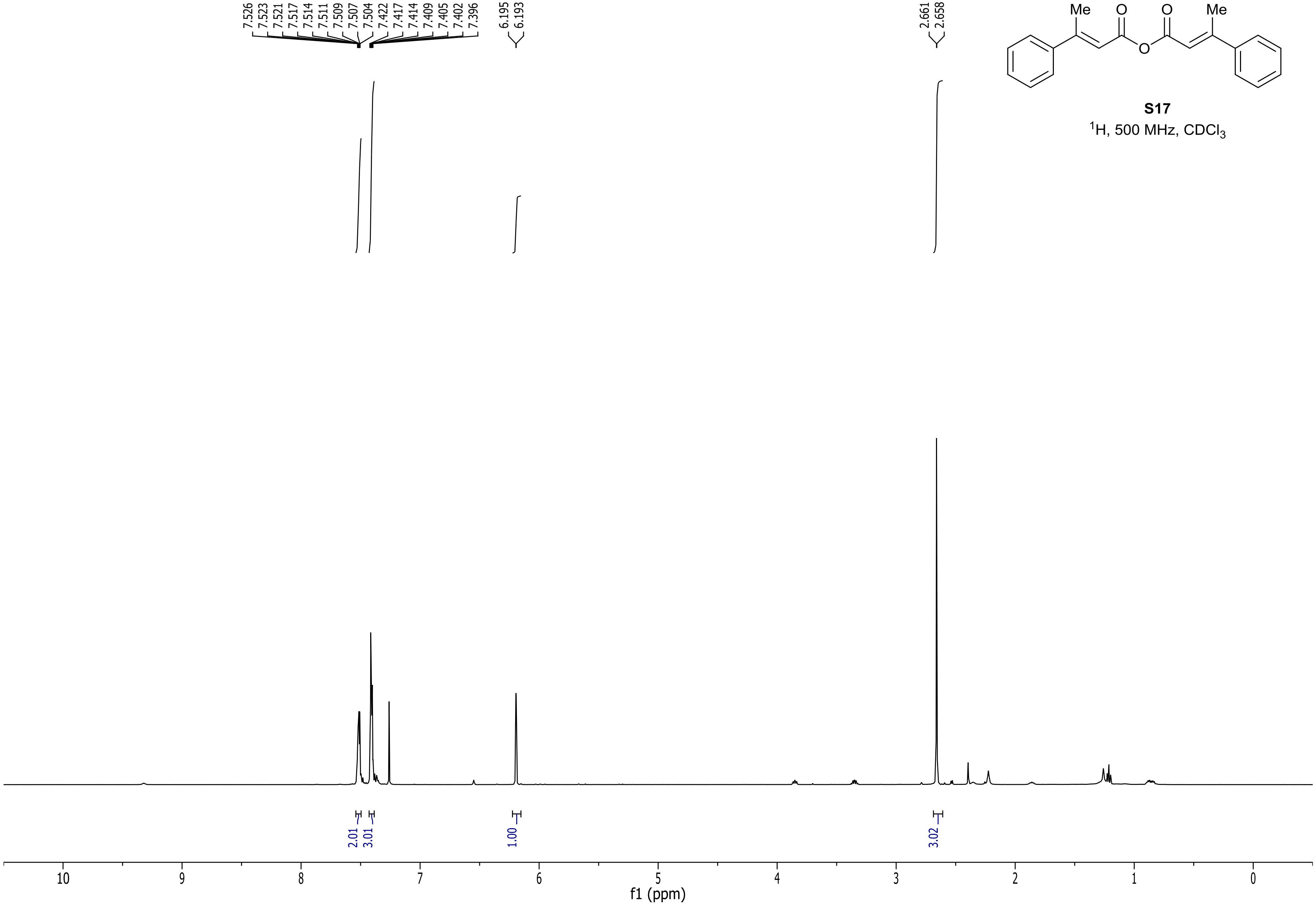

S17

^{1}H, 500 MHz, $CDCl_3$

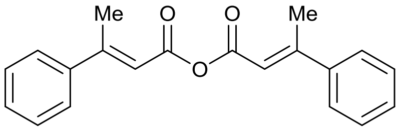

S17
^{13}C, 125 MHz, $CDCl_3$

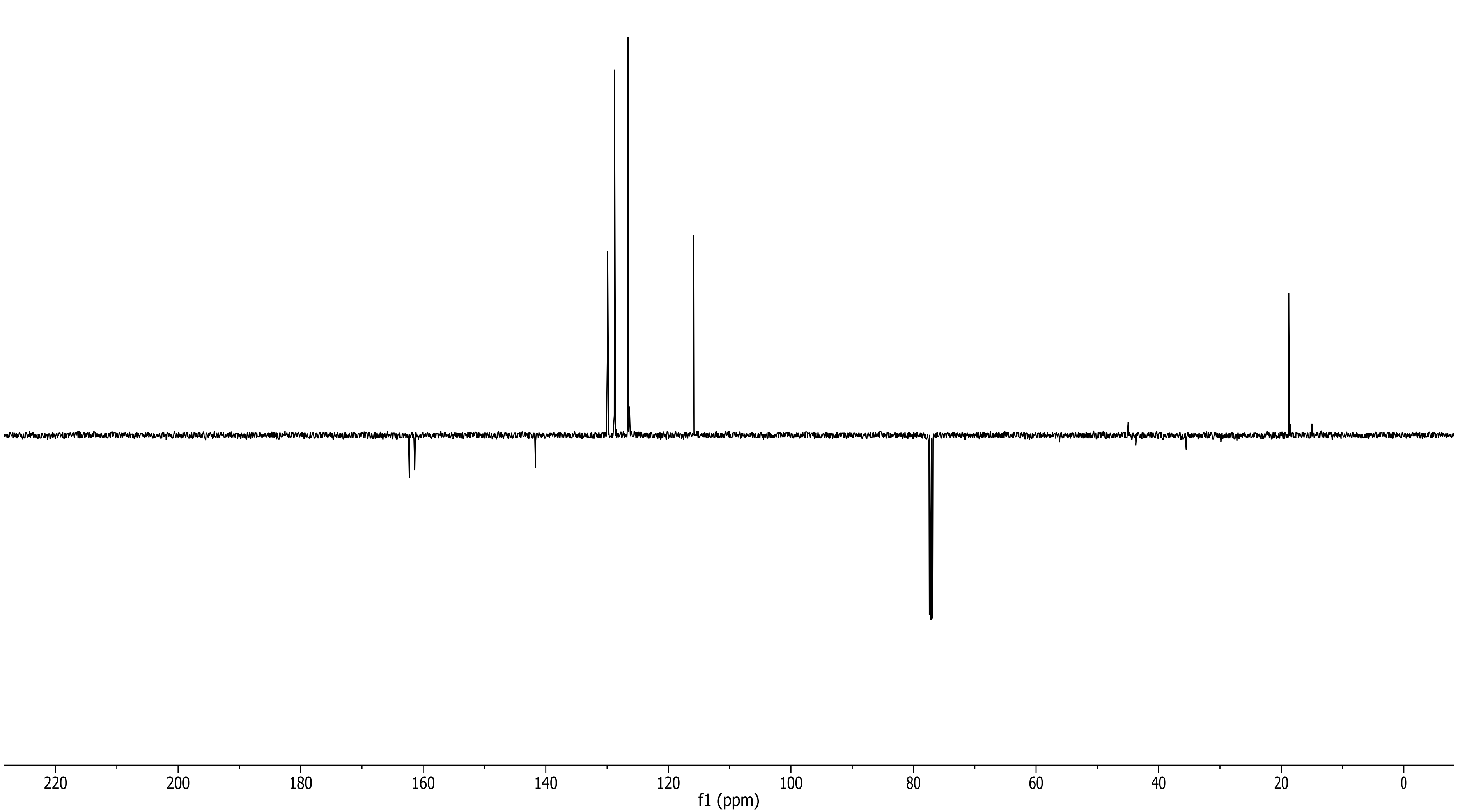

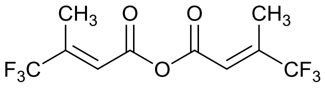

34
^{1}H, 500 MHz, $CDCl_3$
used crude

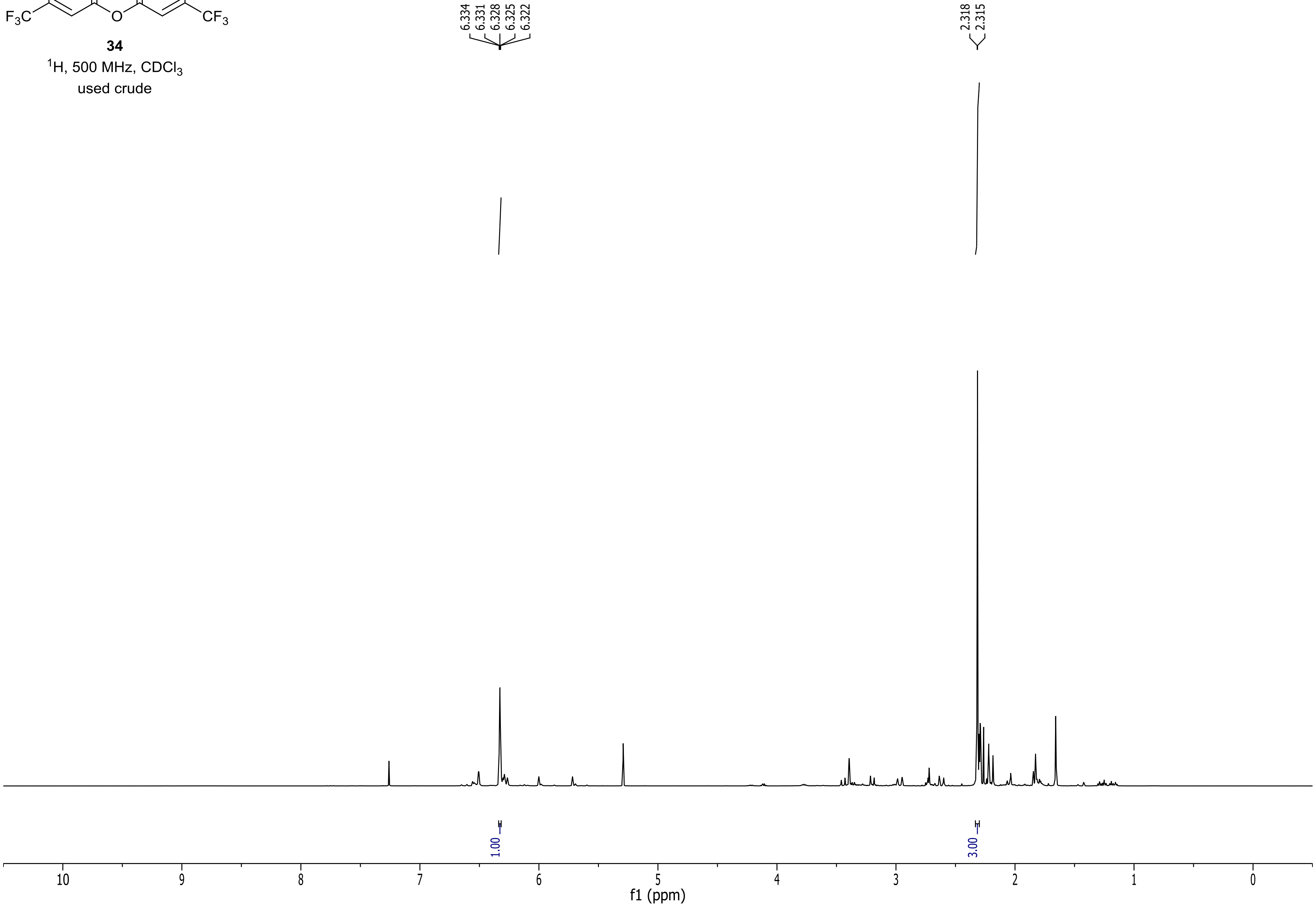

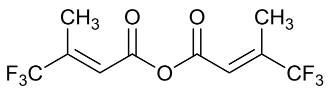

34

^{13}C, 125 MHz, $CDCl_3$

used crude

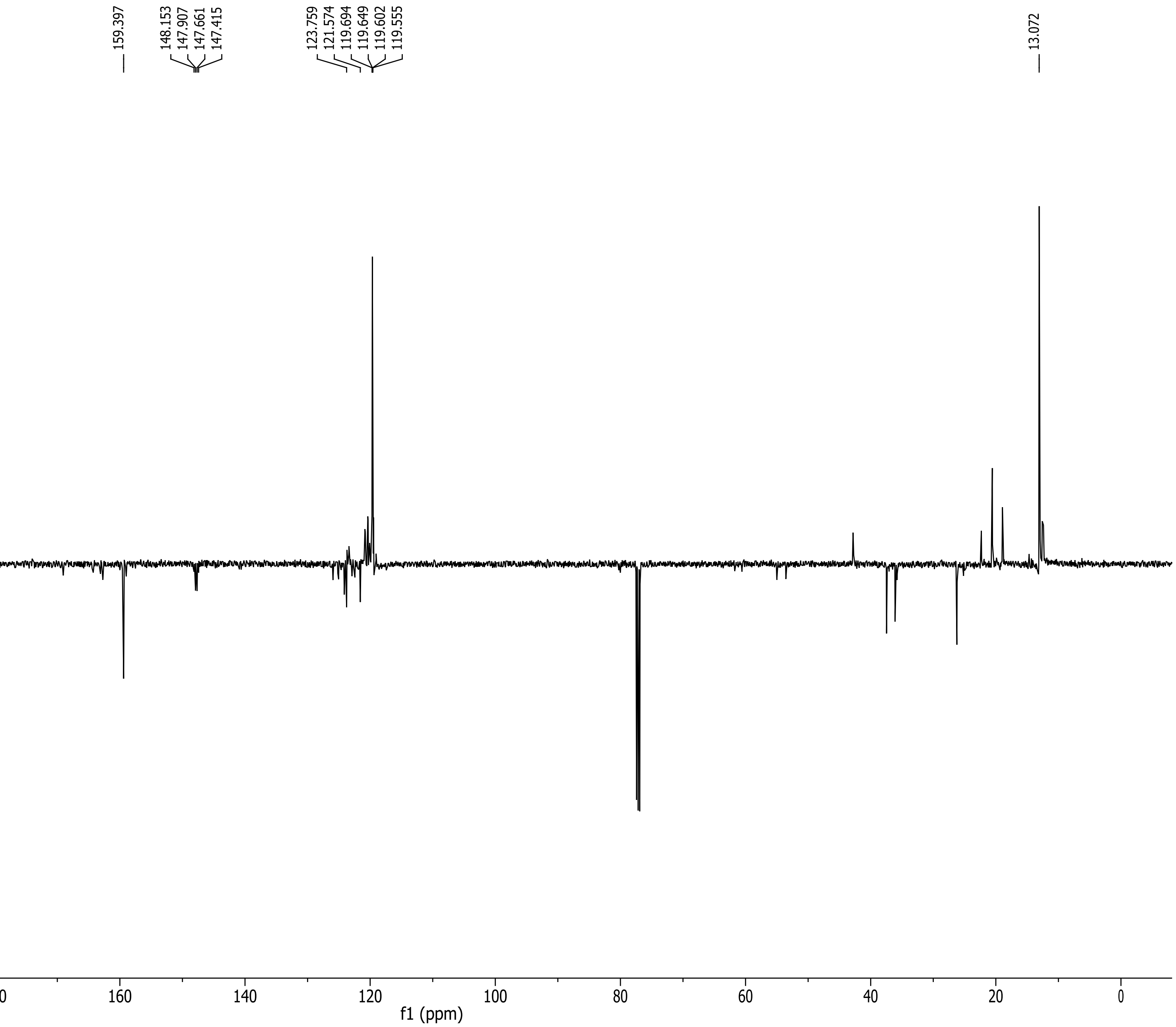

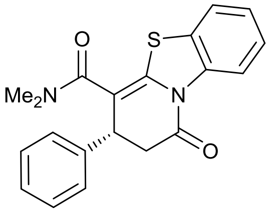

2A

^{1}H, 500 MHz, $CDCl_3$

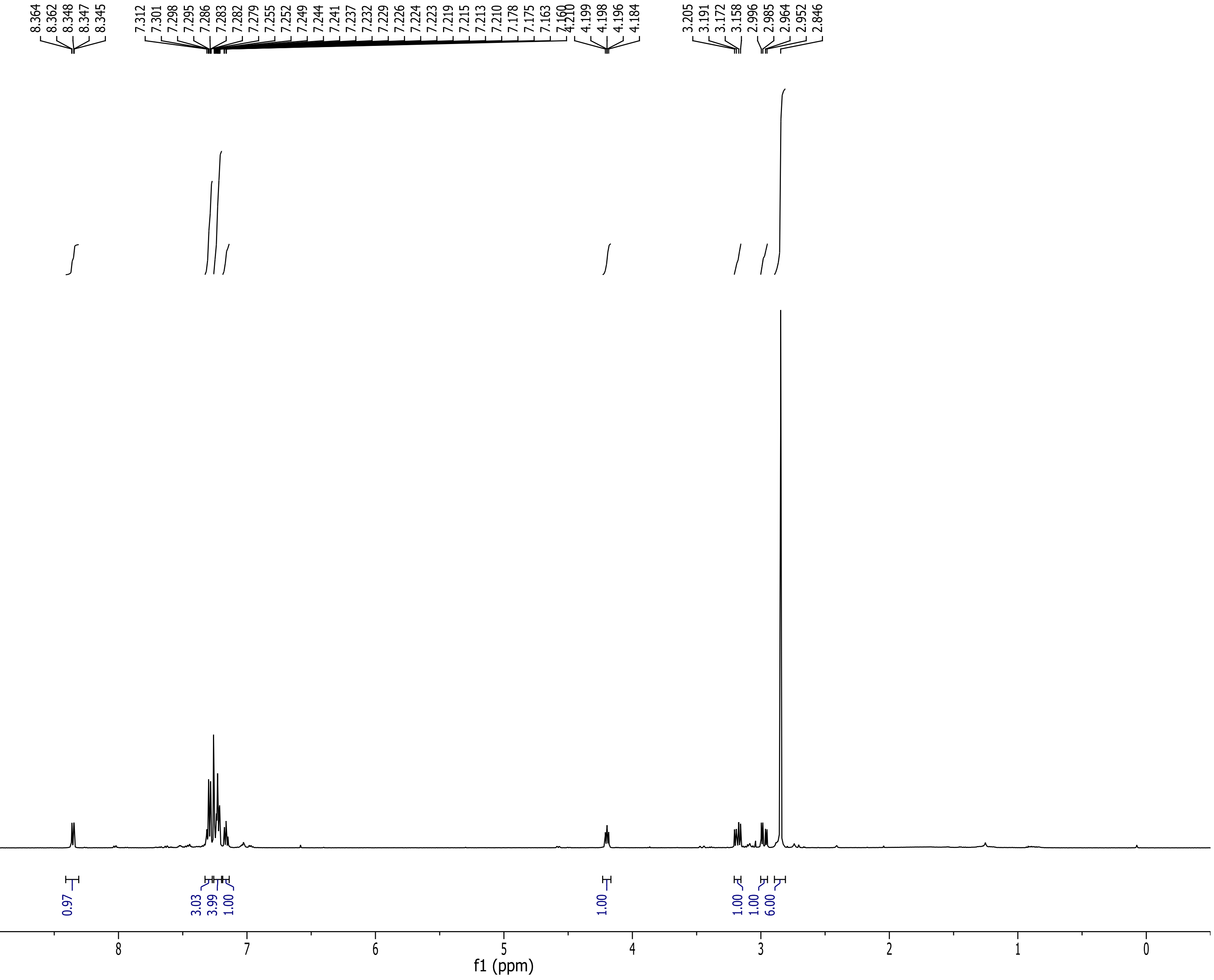

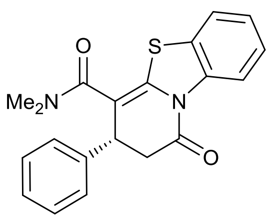

2A

^{13}C, 125 MHz, $CDCl_3$

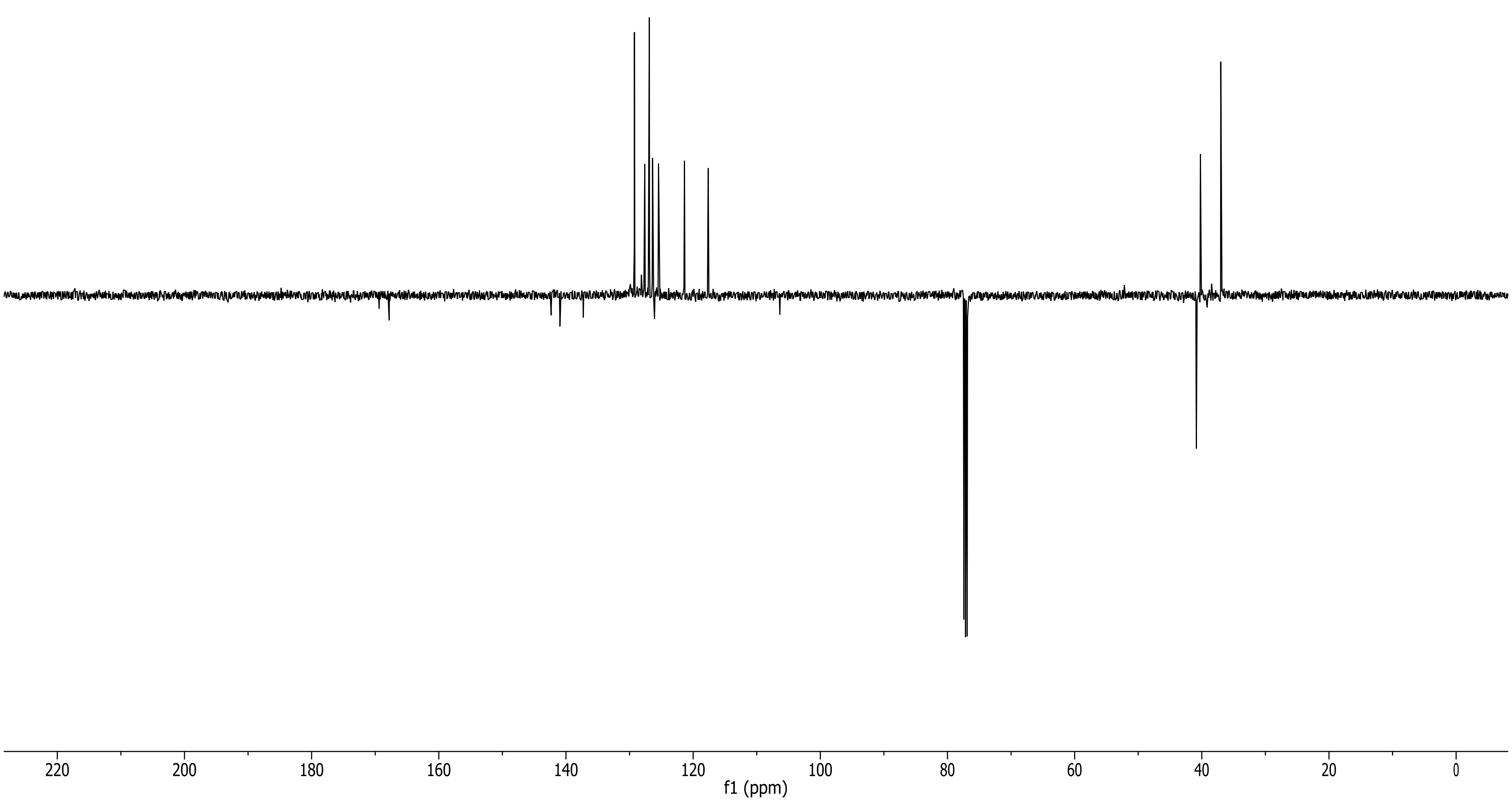

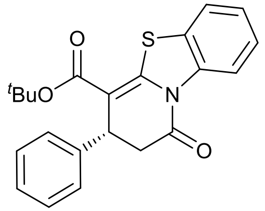

3A

^1H, 500 MHz, $CDCl_3$

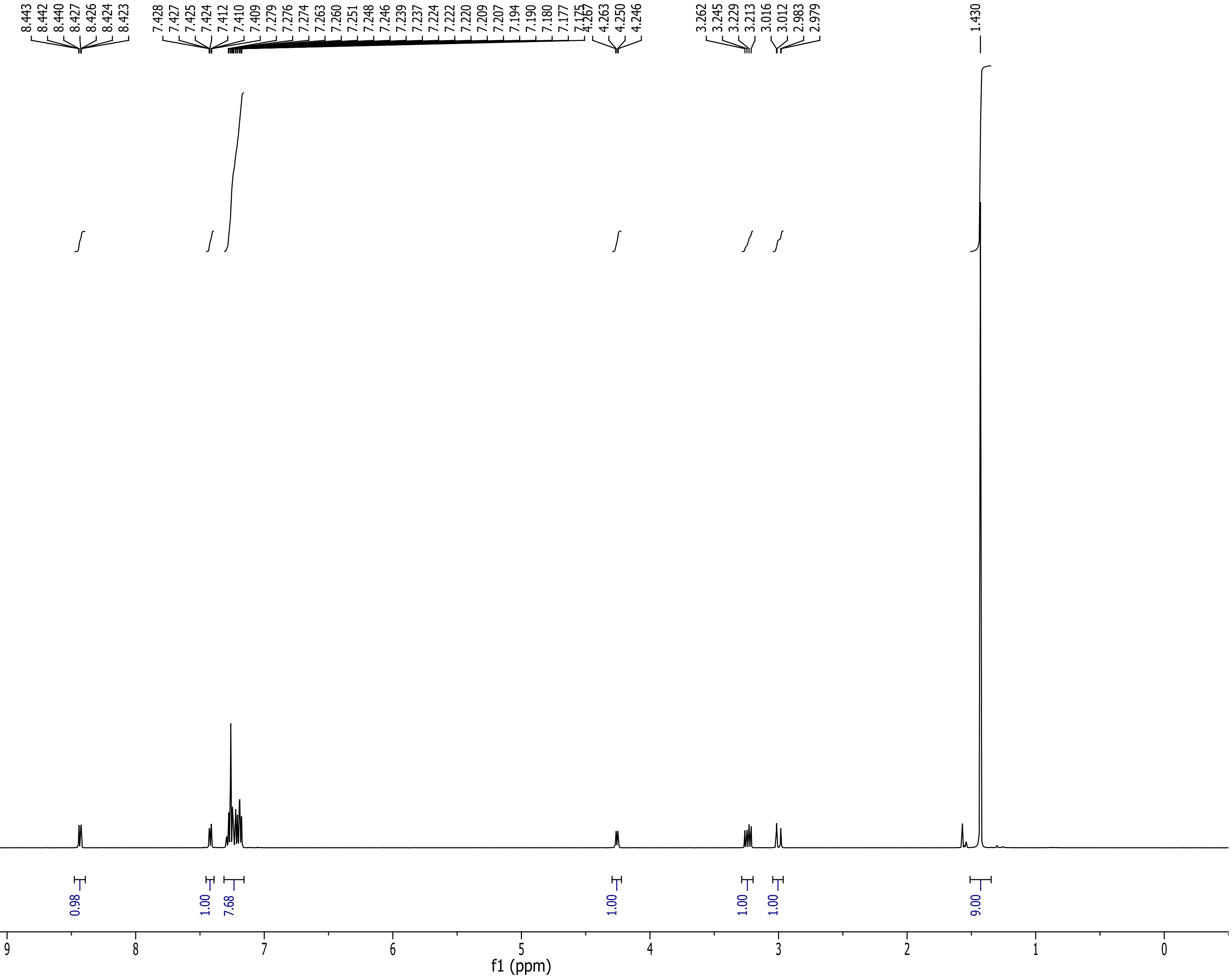

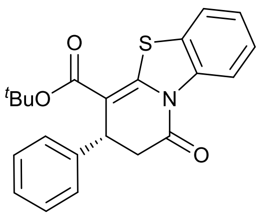

3A

^{13}C, 125 MHz, $CDCl_3$

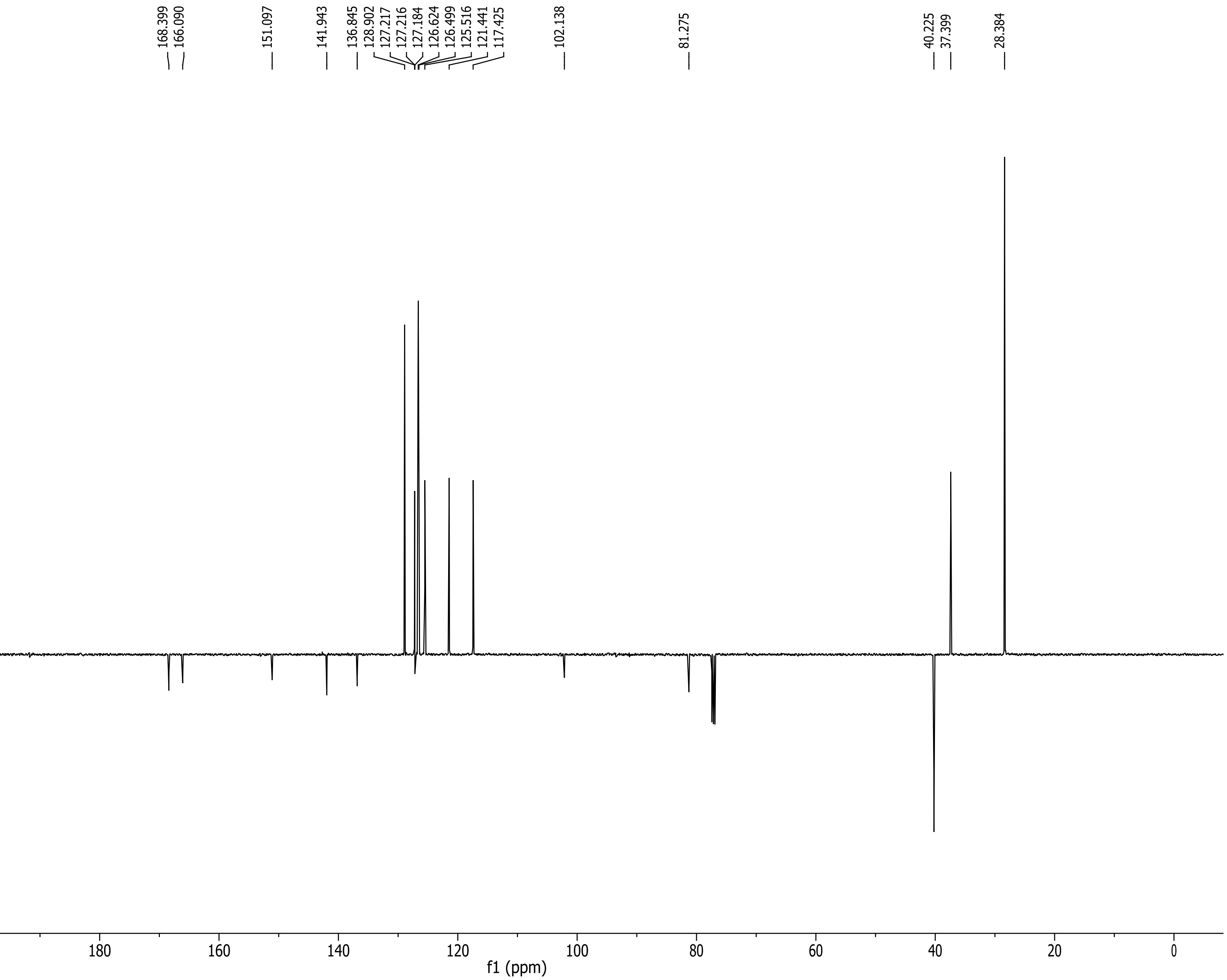

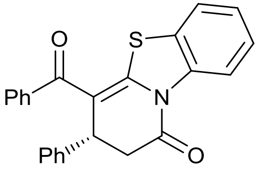

4A

^{1}H, 500 MHz, $CDCl_3$

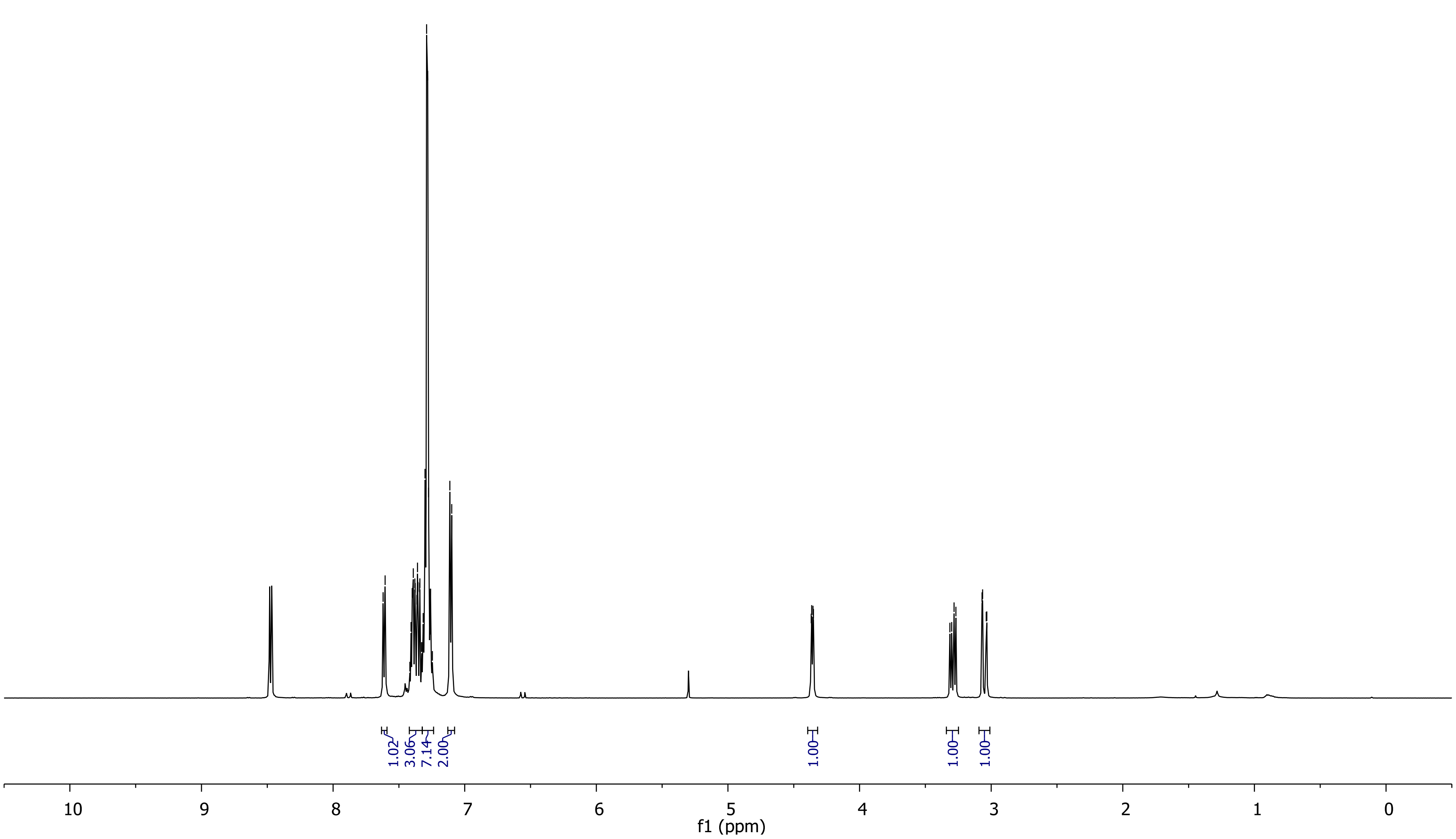

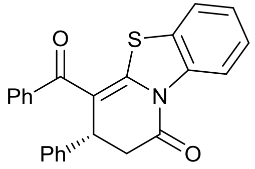

4A

^{13}C, 126 MHz, $CDCl_3$

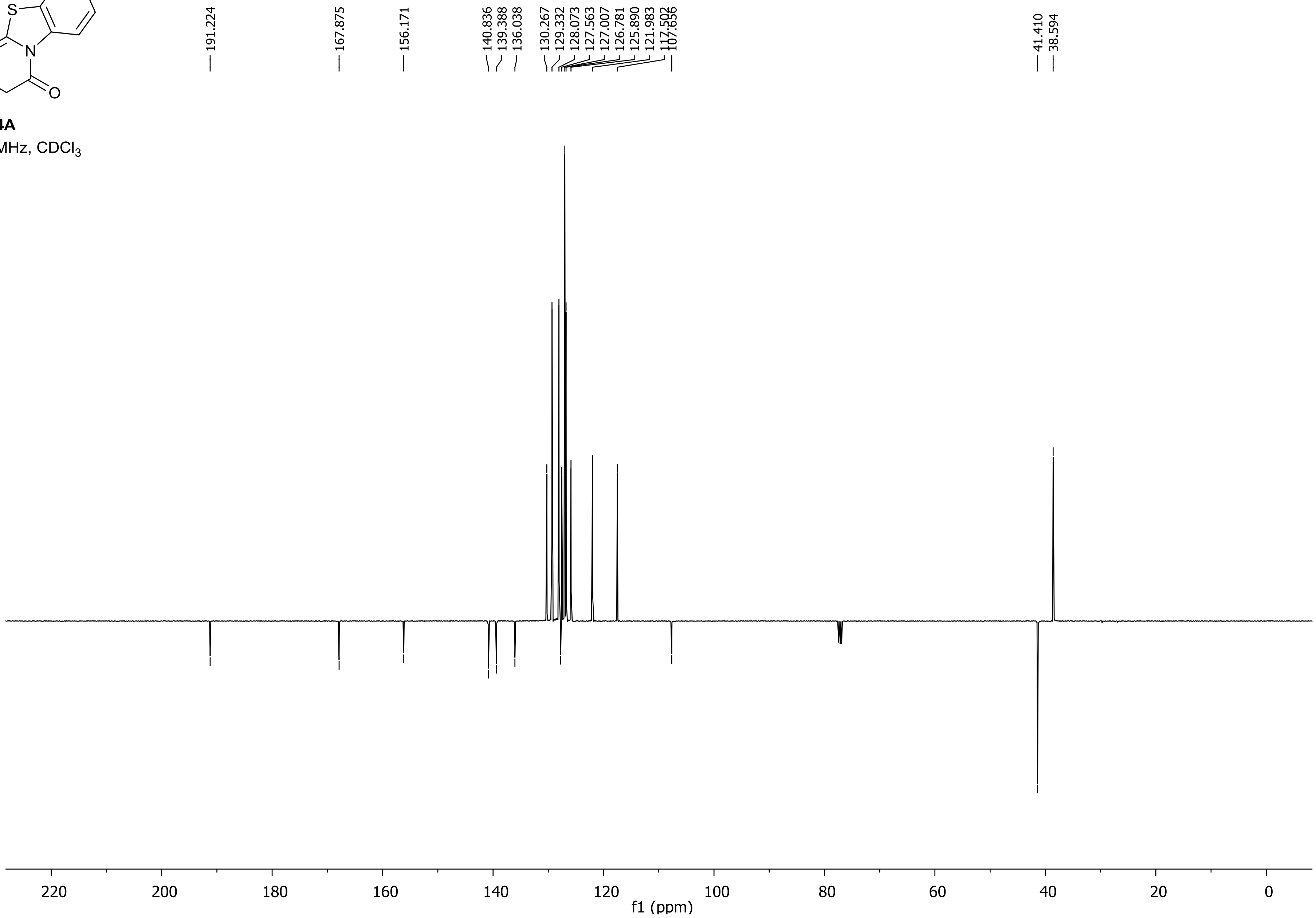

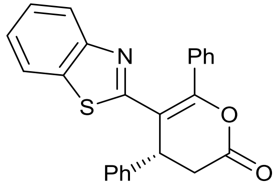

4B

^{1}H, 300 MHz, $CDCl_3$

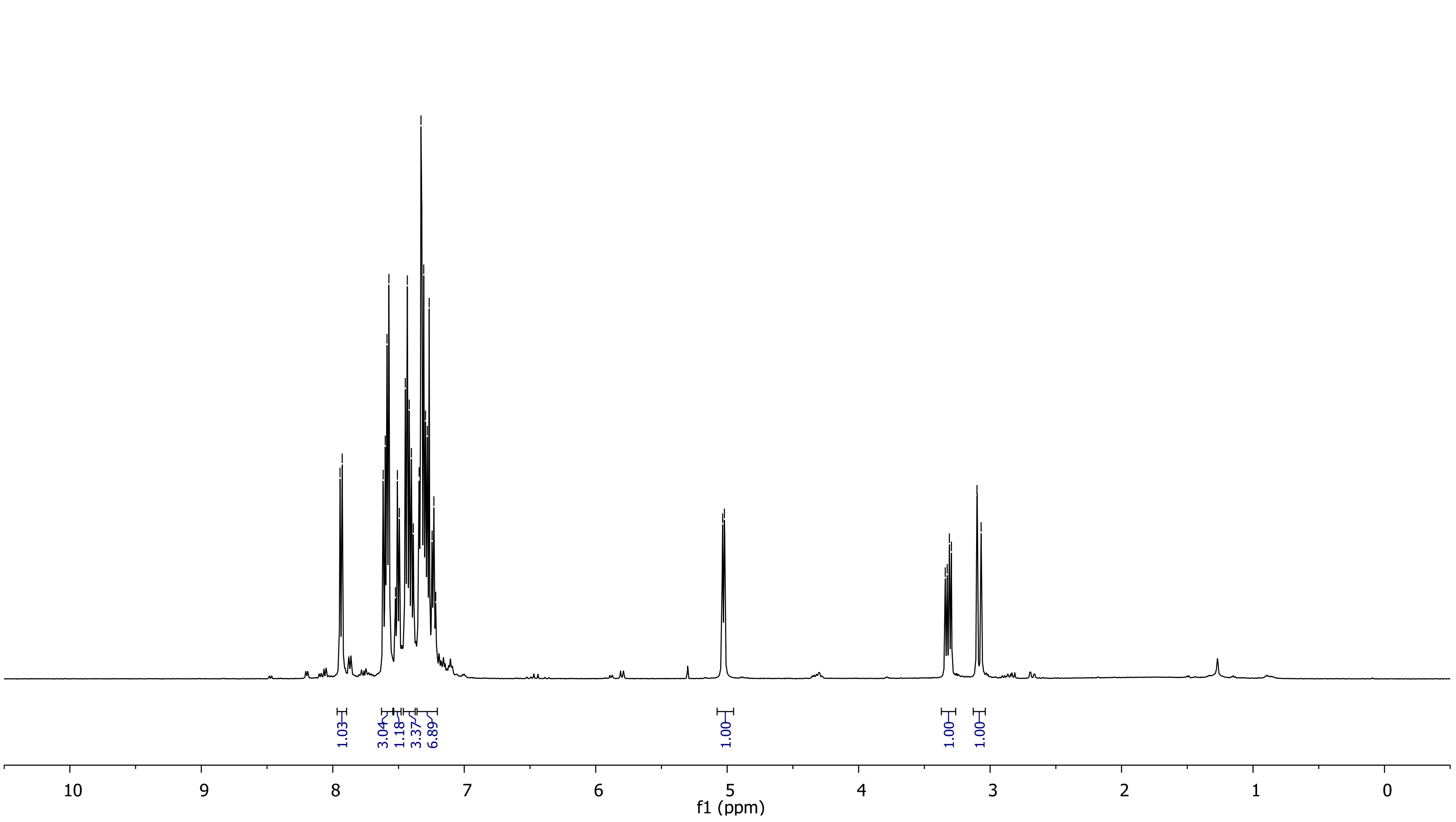

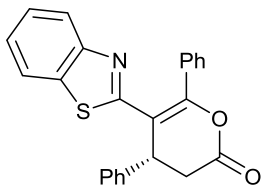

4B

^{13}C, 75 MHz, $CDCl_3$

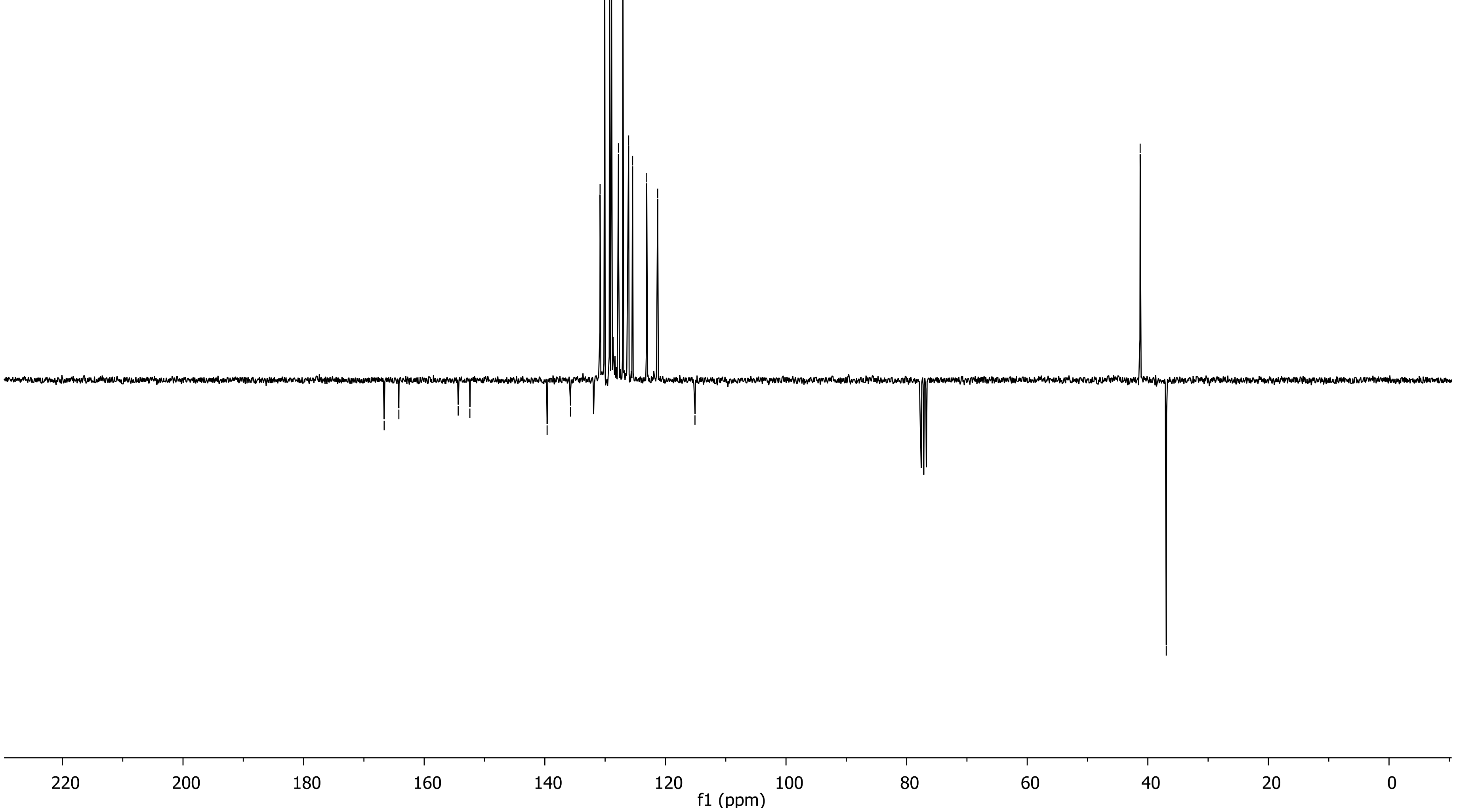

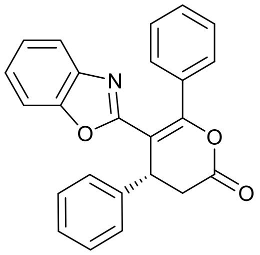

5B

^{1}H, 500 MHz, $CDCl_3$

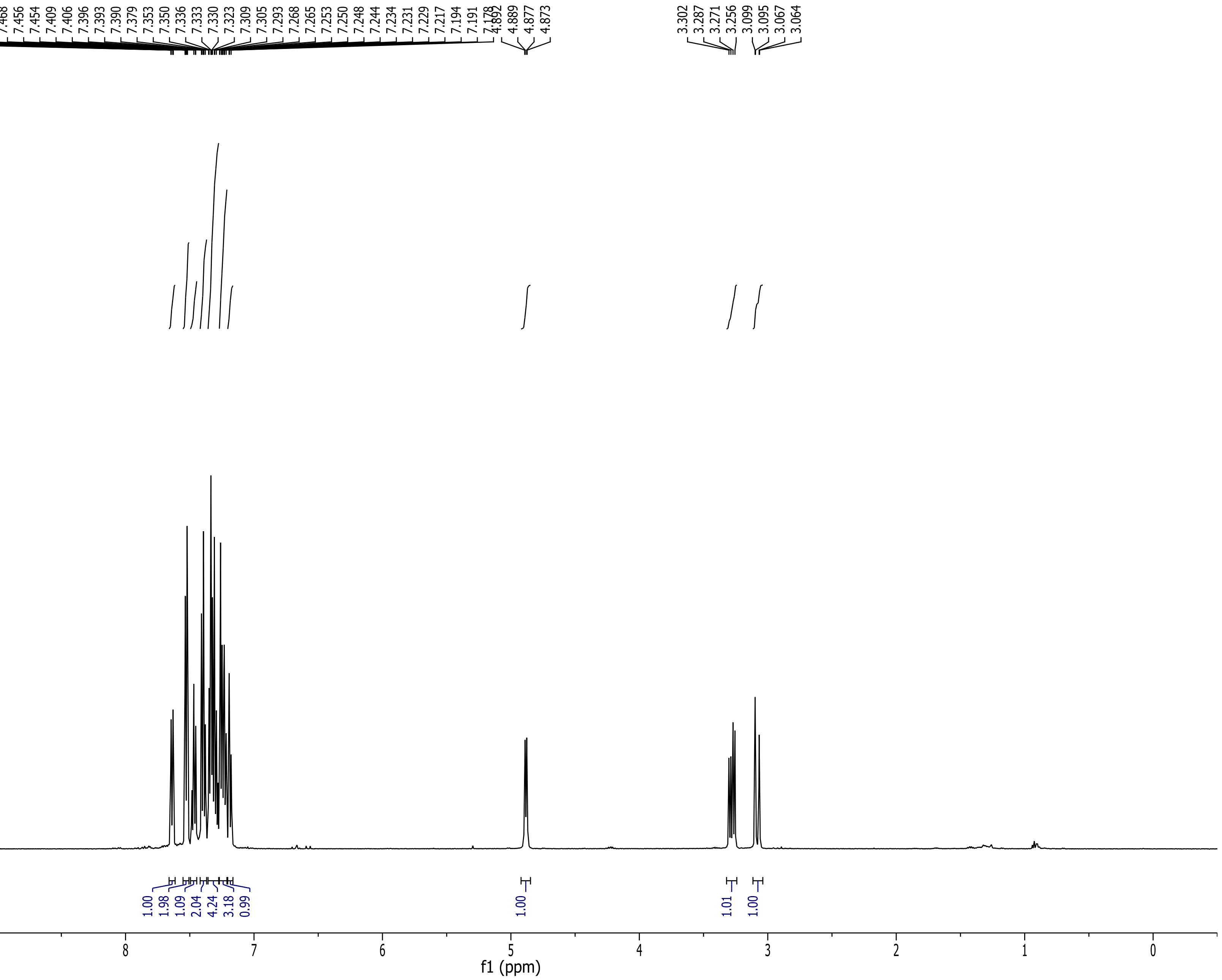

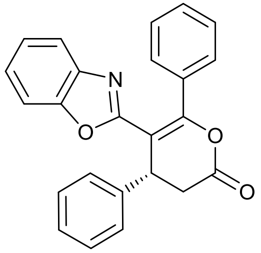

5B
^{13}C, 125 MHz, $CDCl_3$

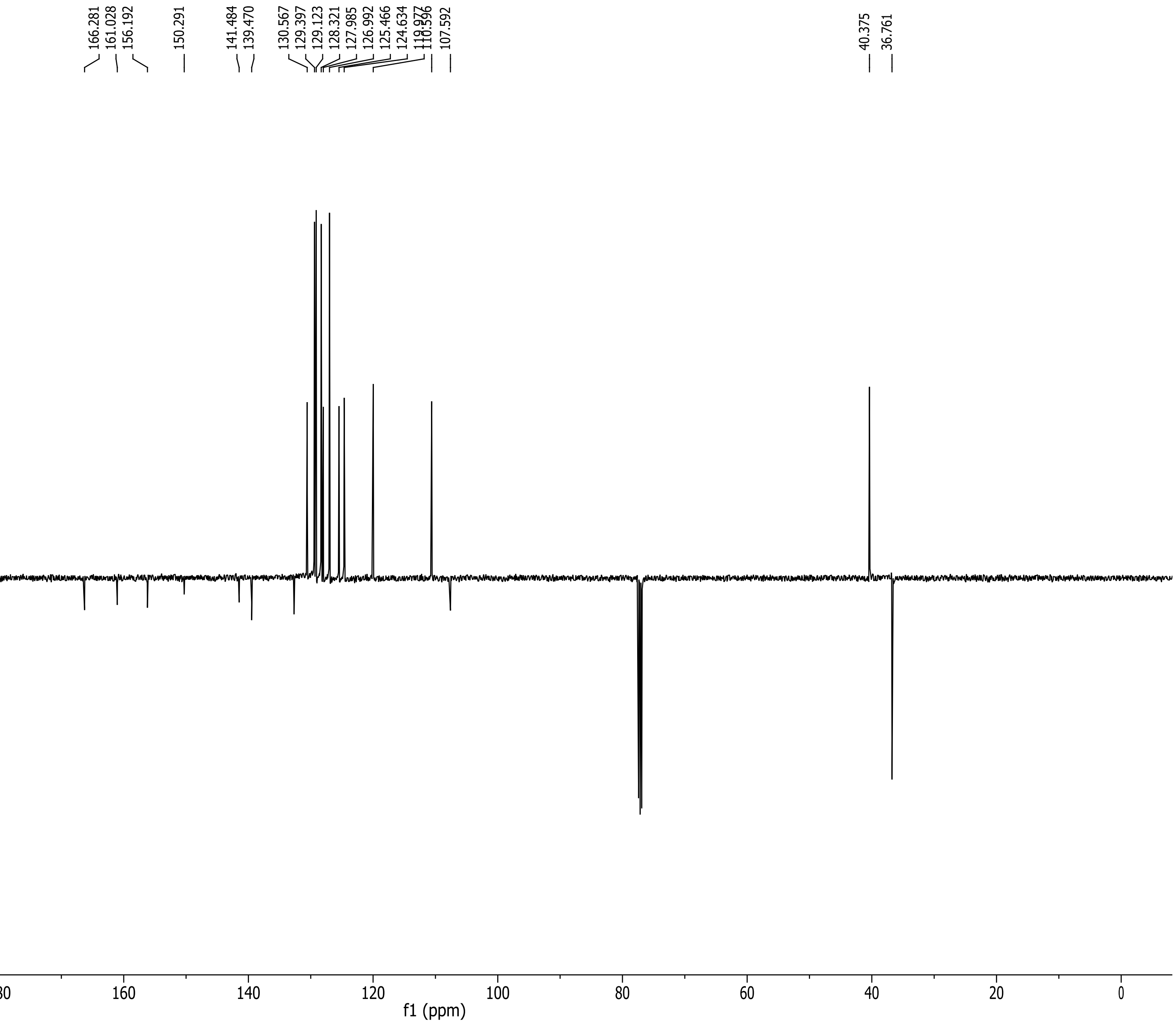

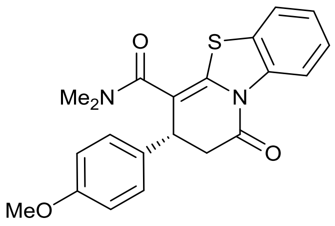

6A

^{1}H, 500 MHz, $CDCl_3$

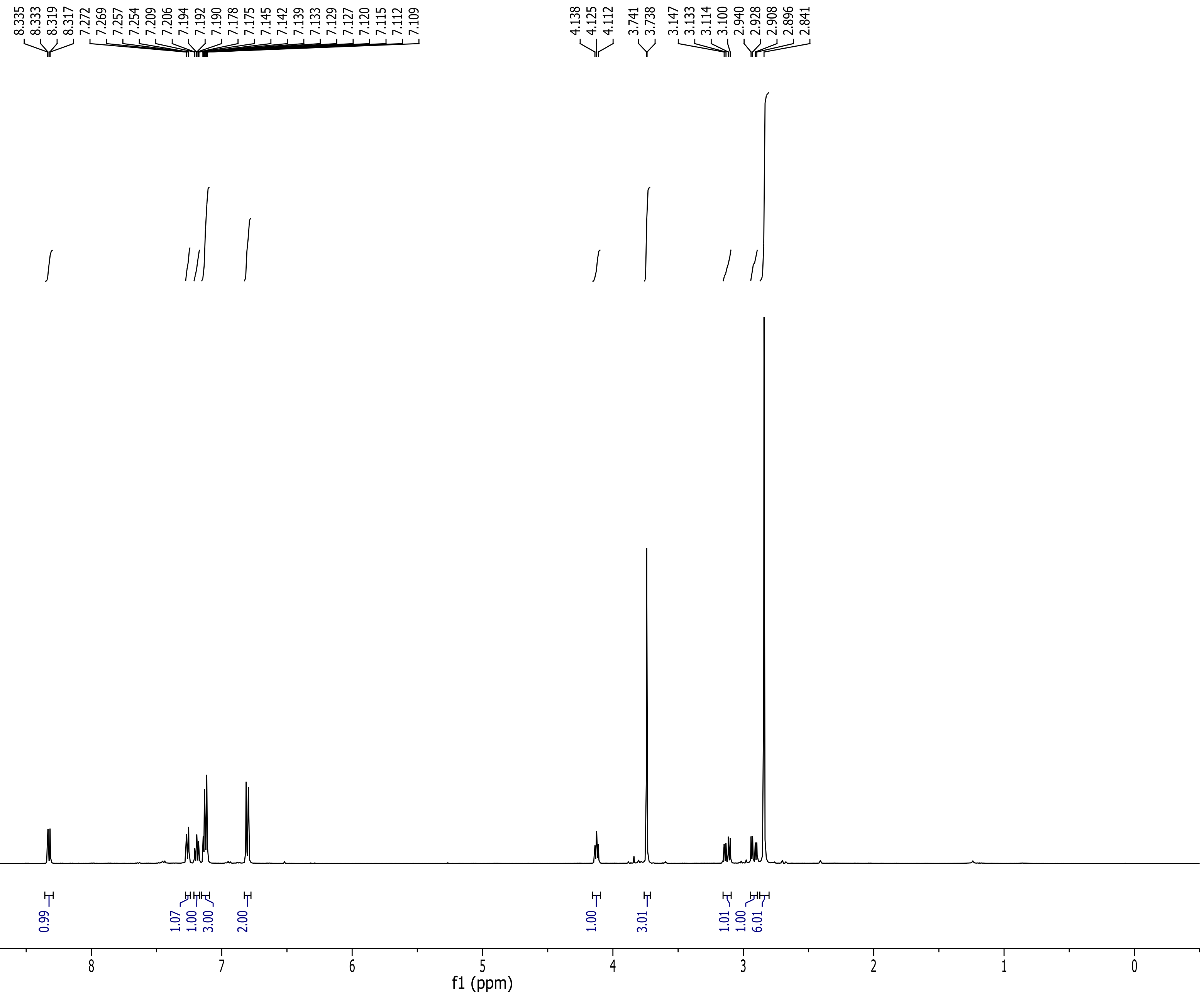

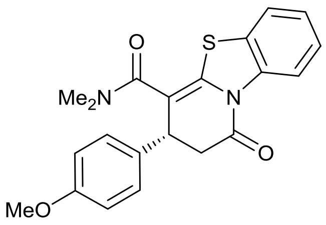

6A

^{13}C, 125 MHz, $CDCl_3$

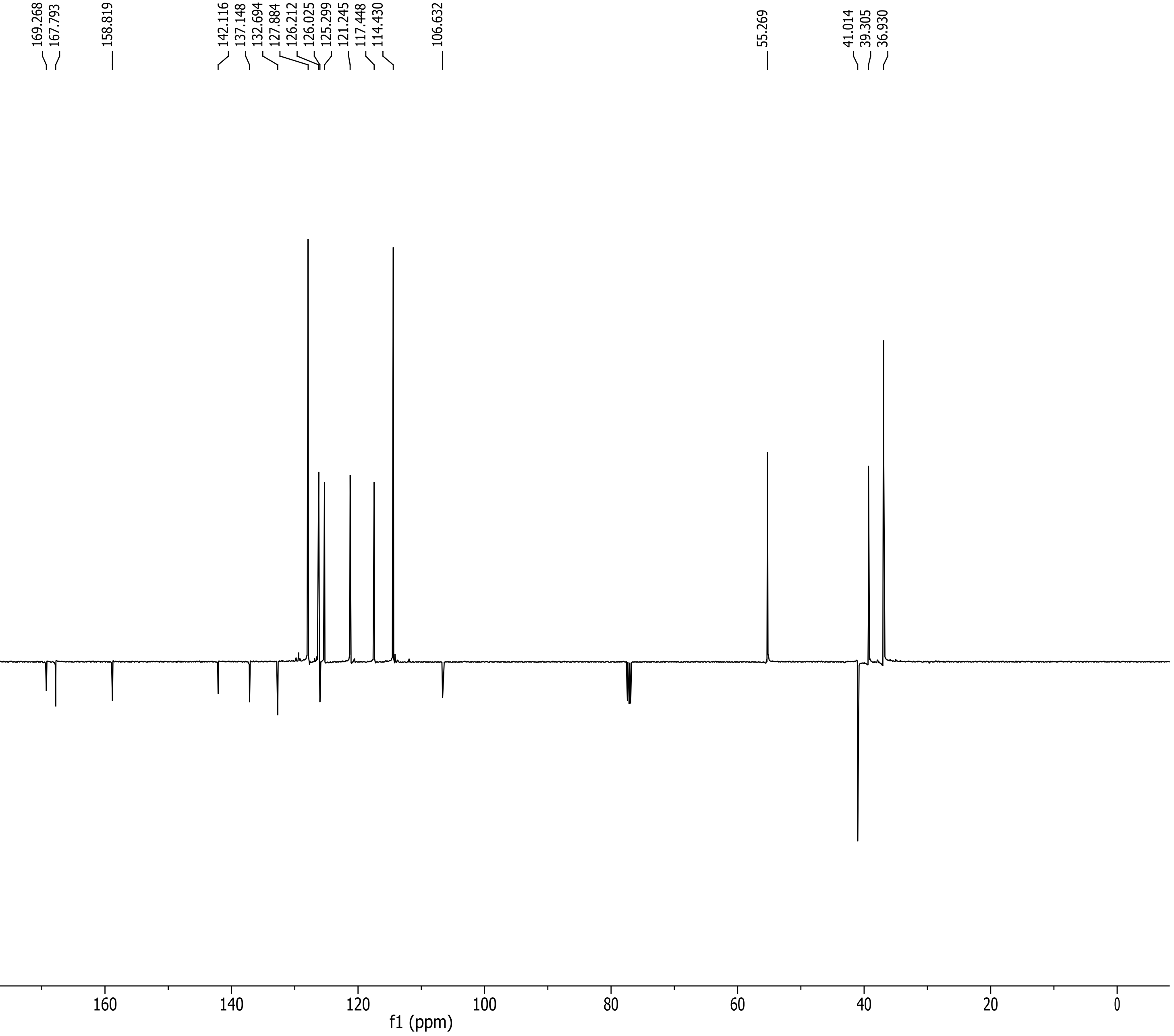

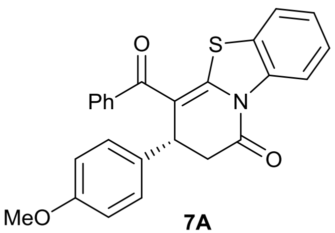

7A

^{1}H, 300 MHz, $CDCl_3$

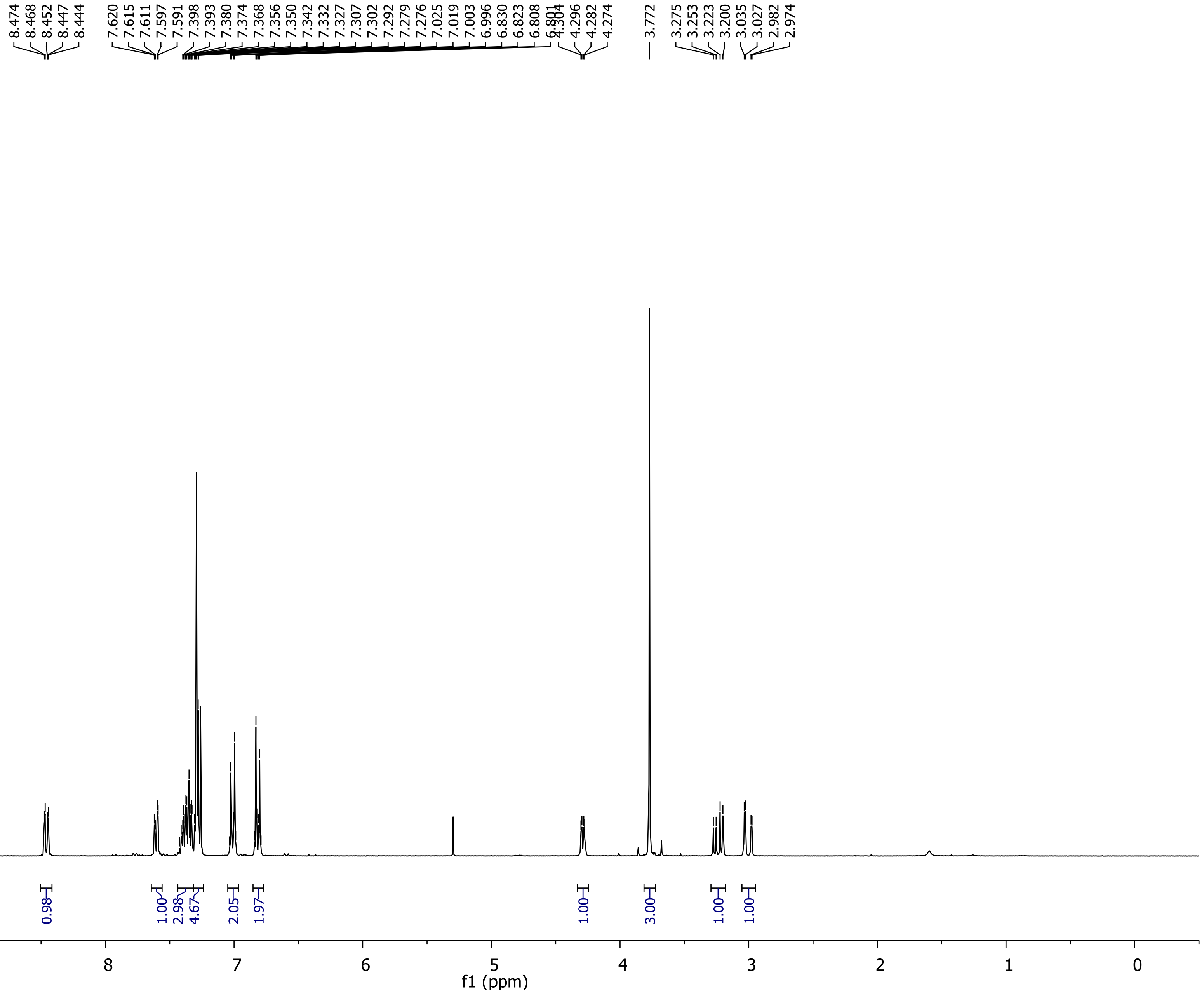

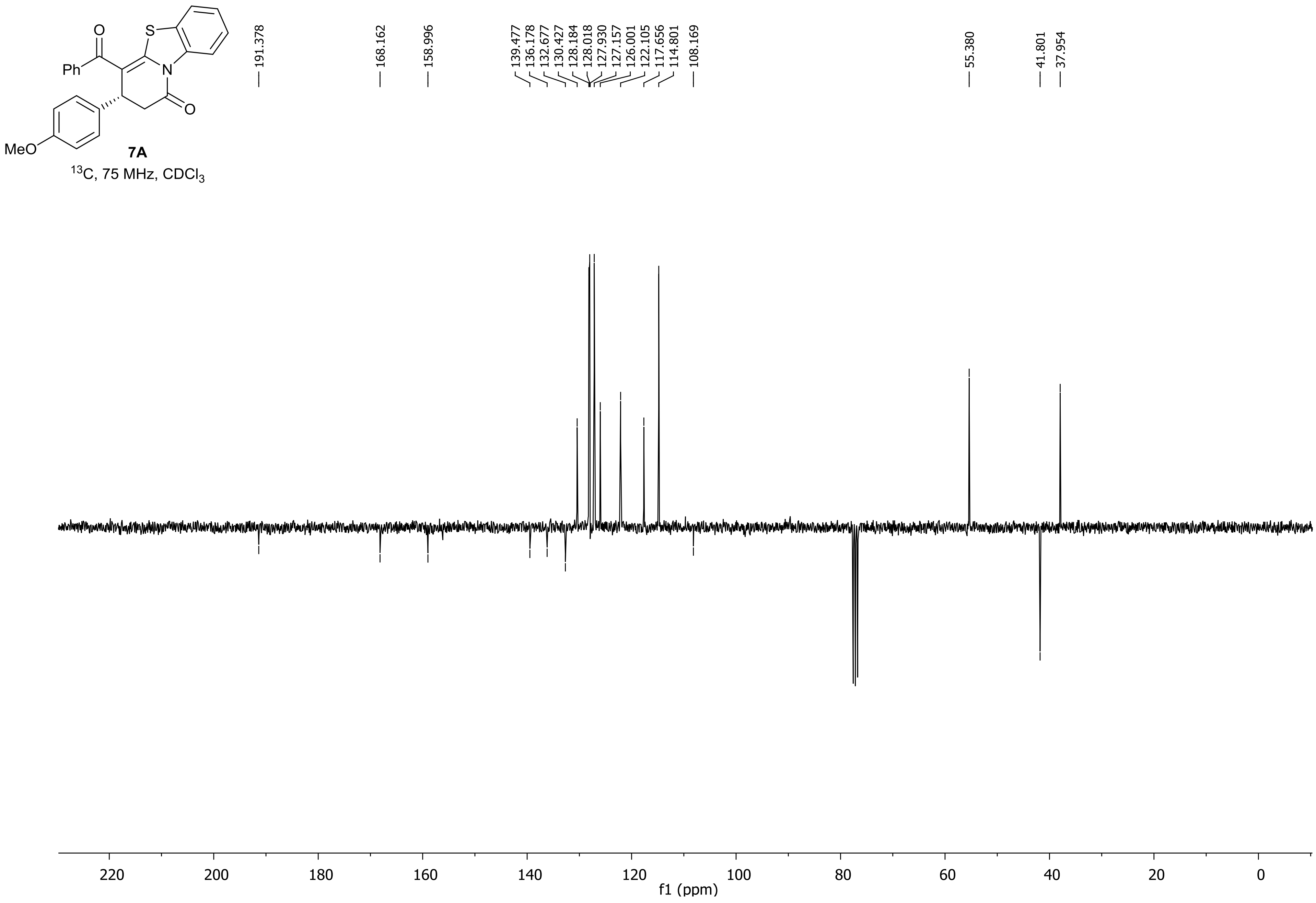

O
S
Ph
N
O
MeO
7A
^{13}C, 75 MHz, $CDCl_3$
191.378
168.162
158.996
139.477
136.178
132.677
130.427
128.184
128.018
127.930
127.157
126.001
122.105
117.656
114.801
108.169
55.380
41.801
37.954
220 200 180 160 140 120 100 80 60 40 20 0
f1 (ppm)

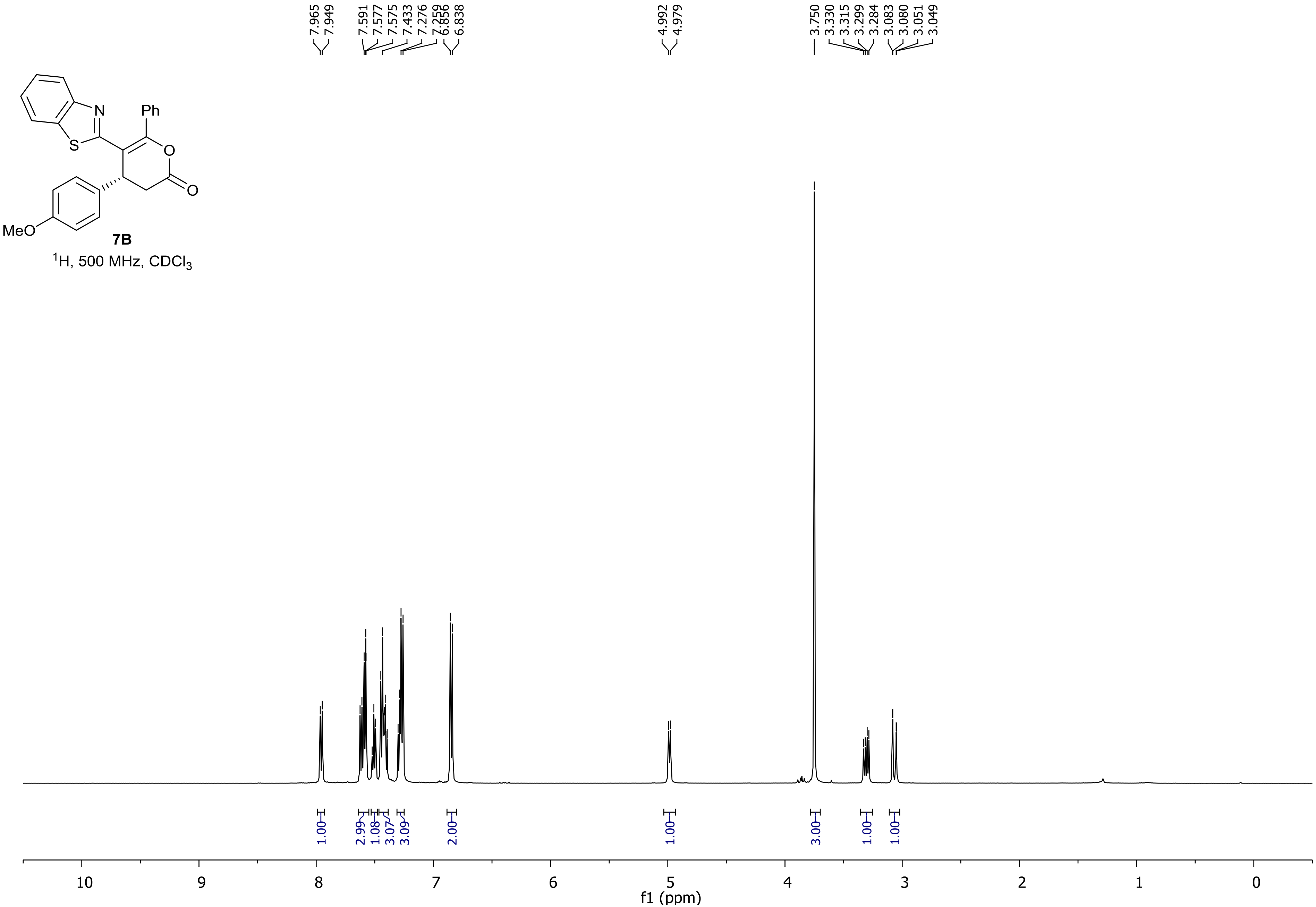
Ph
N
S
O
O
MeO
7B
1H, 500 MHz, CDCl3
7.965
7.949
7.591
7.577
7.575
7.433
7.276
7.259
6.856
6.838
4.992
4.979
3.750
3.330
3.315
3.299
3.284
3.083
3.080
3.051
3.049
1.00
2.99
1.08
3.07
3.09
2.00
1.00
3.00
1.00
1.00
10
9
8
7
6
5
4
3
2
1
0
f1 (ppm)

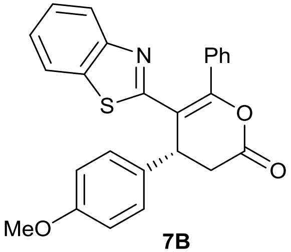

7B

^{13}C, 126 MHz, $CDCl_3$

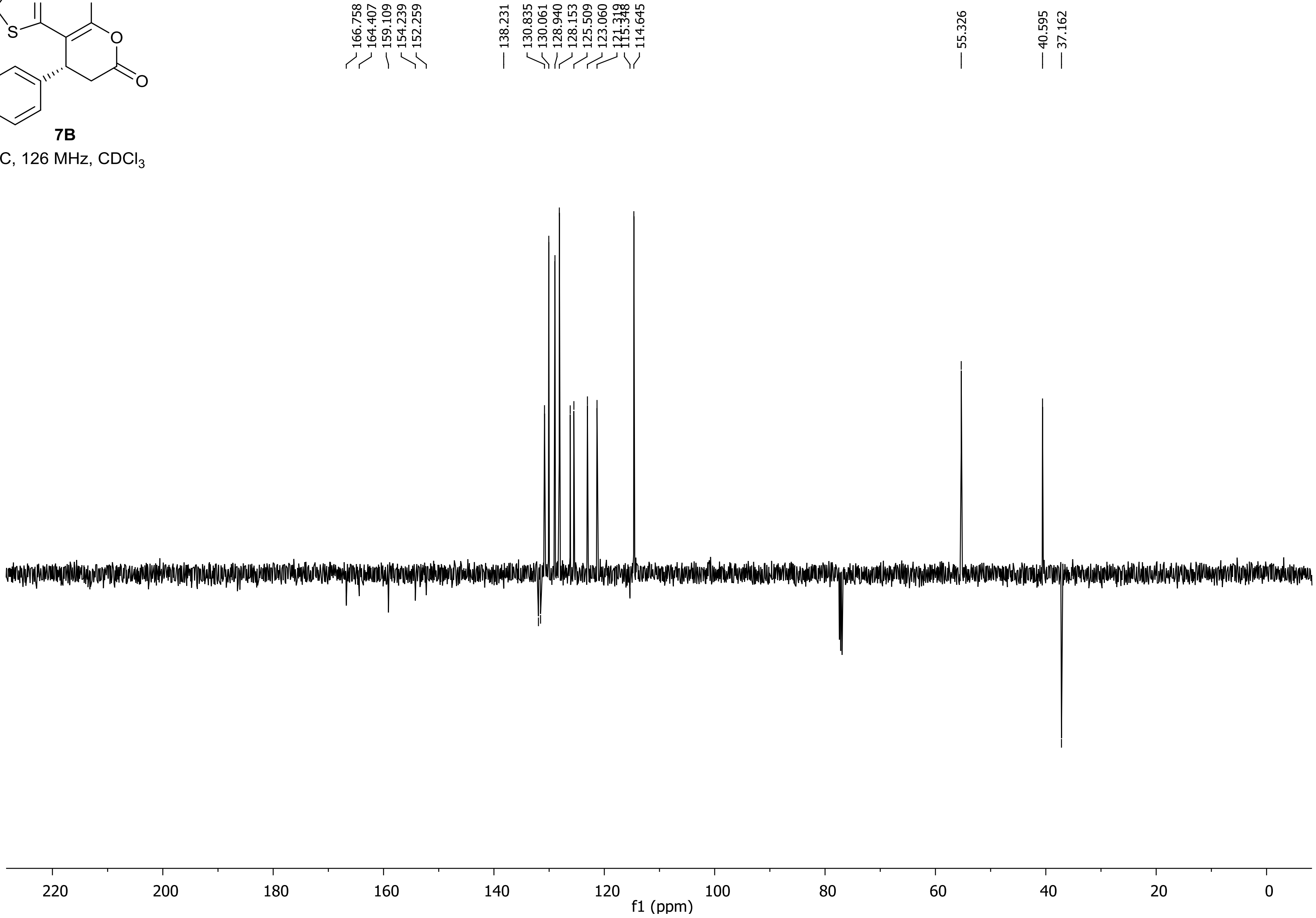

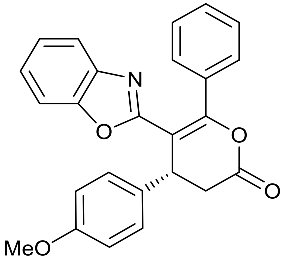

8B

^{1}H, 500 MHz, $CDCl_3$

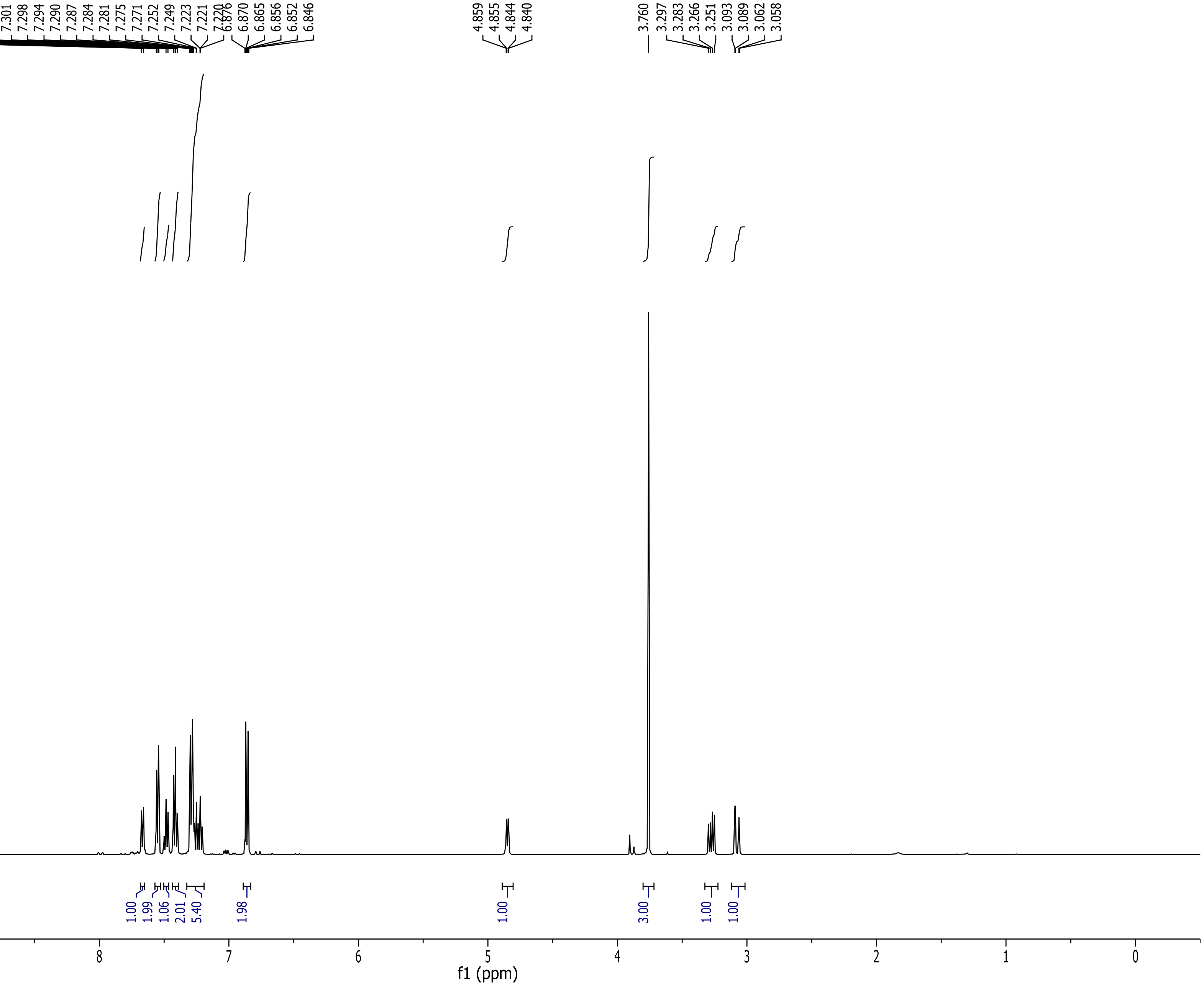

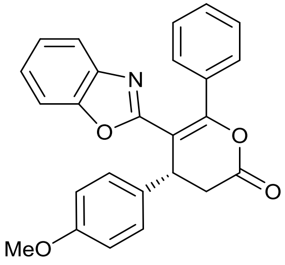

8B

^{13}C, 125 MHz, $CDCl_3$

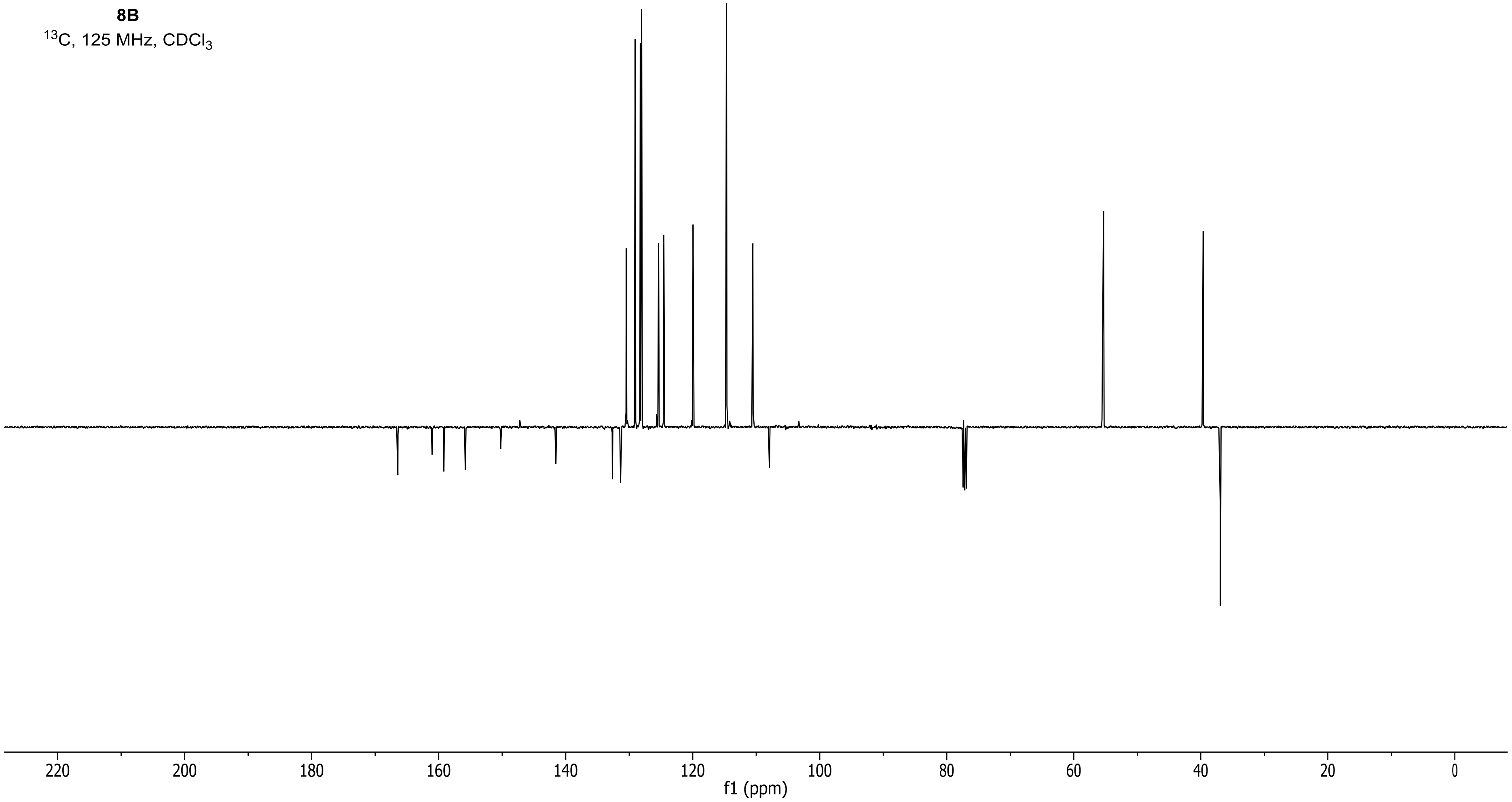

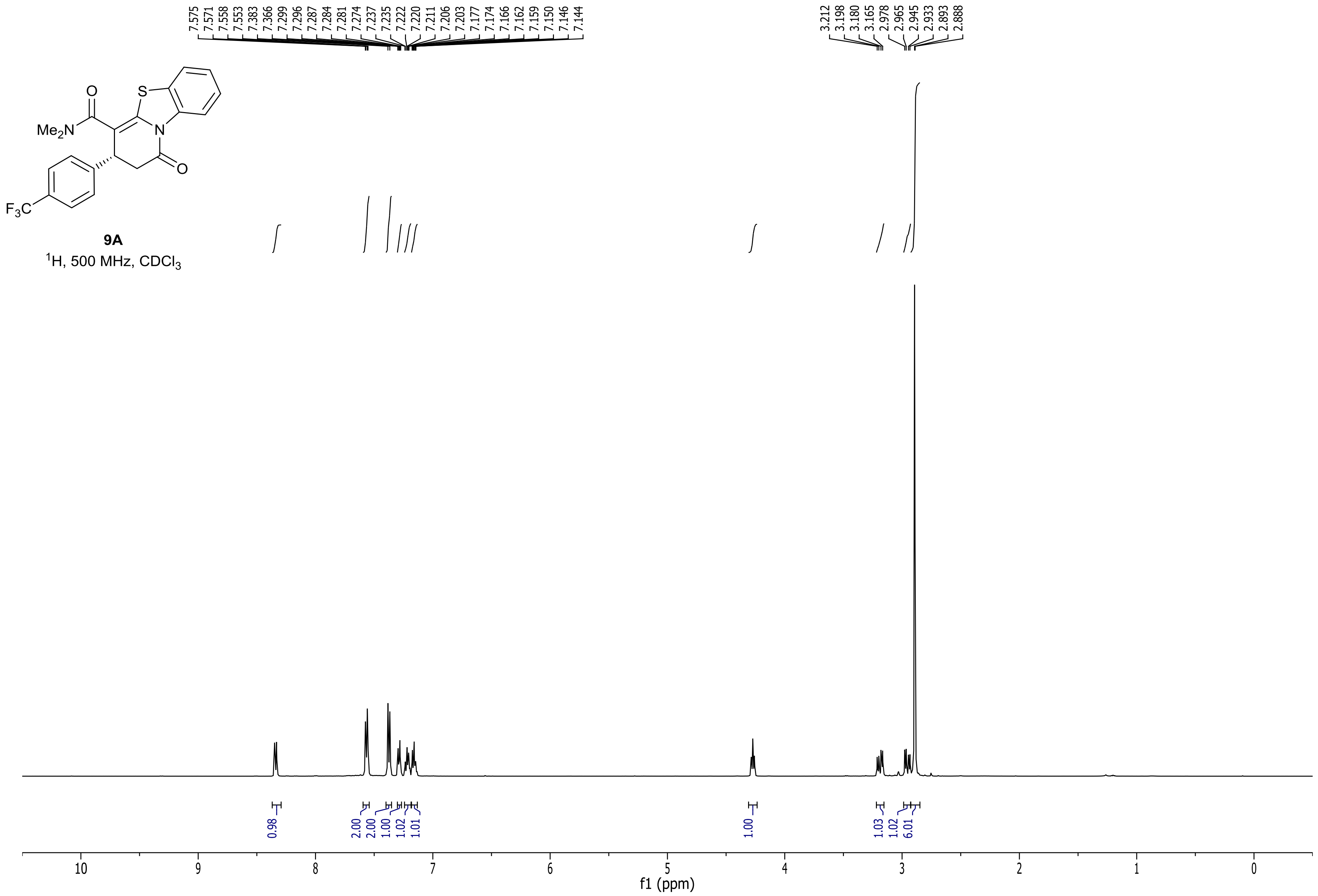

9A

^{1}H, 500 MHz, $CDCl_3$

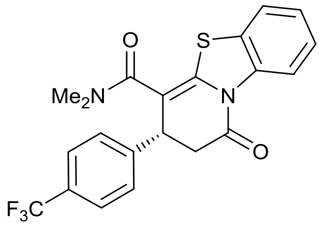

9A
^{13}C, 125 MHz, $CDCl_3$

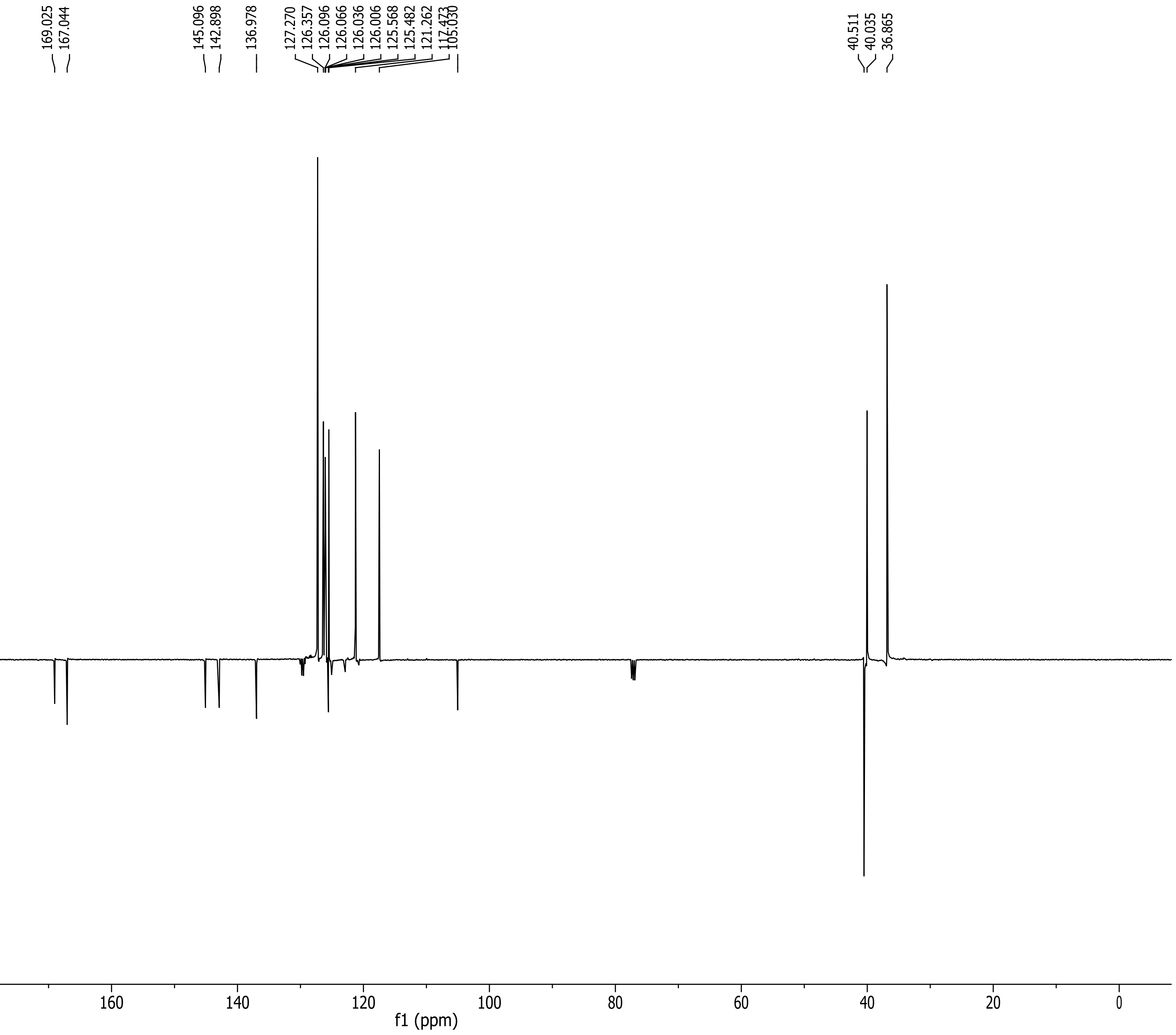

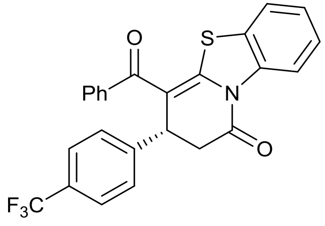

10A
^{1}H, 500 MHz, $CDCl_3$

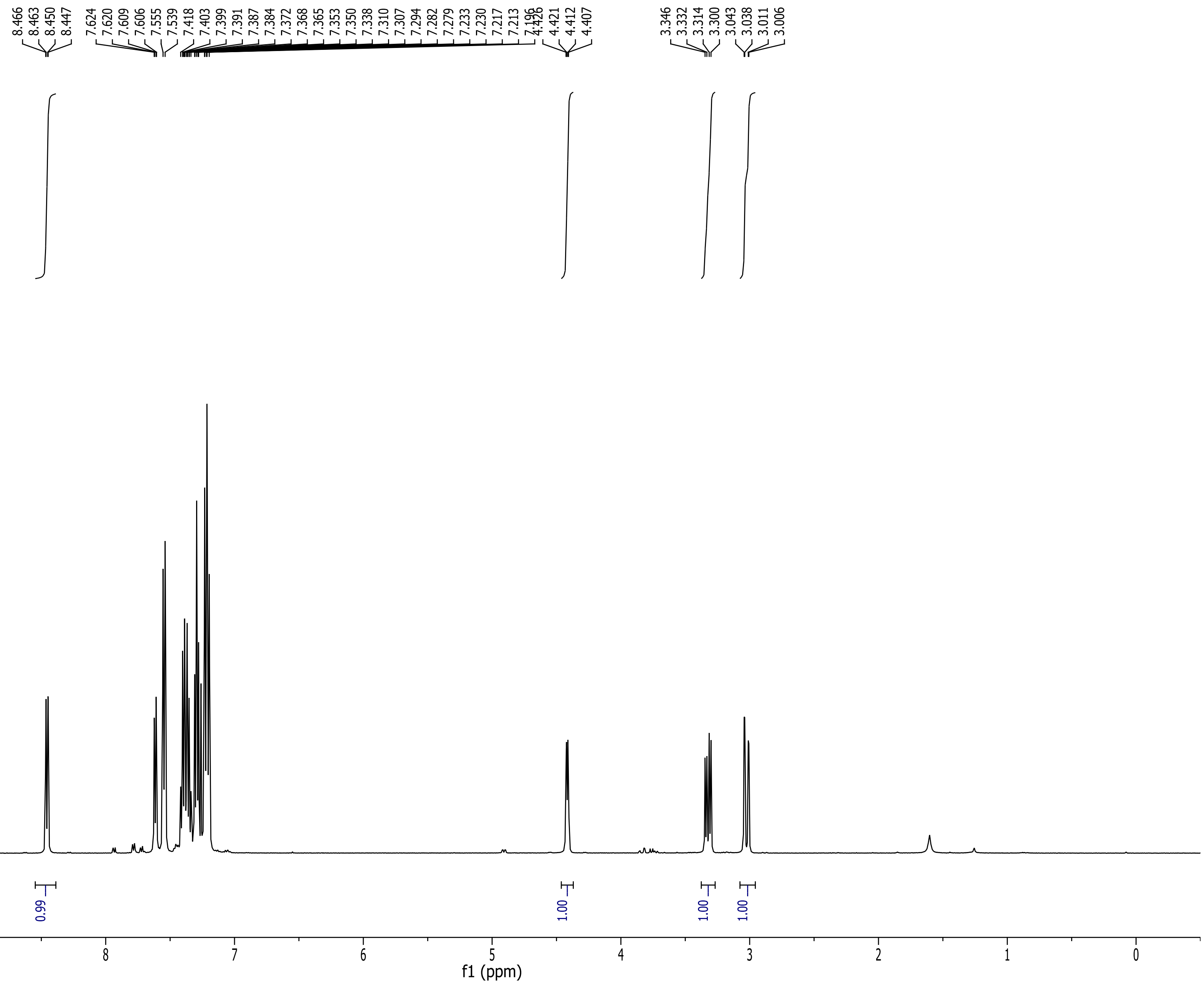

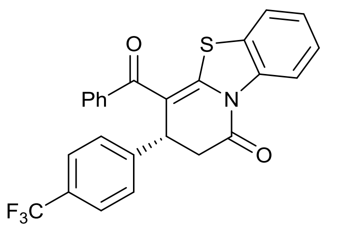

10A
^{13}C, 125 MHz, $CDCl_3$

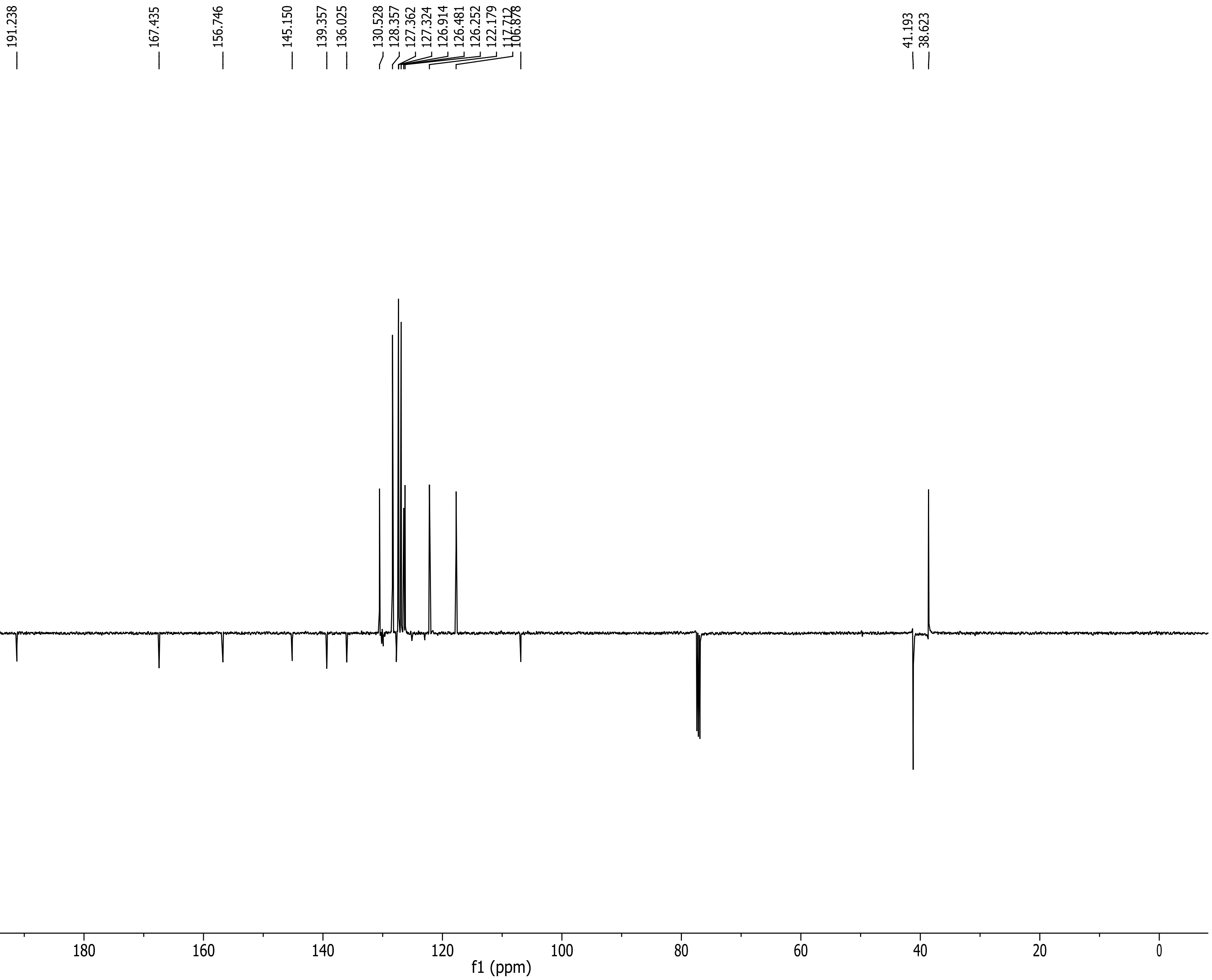

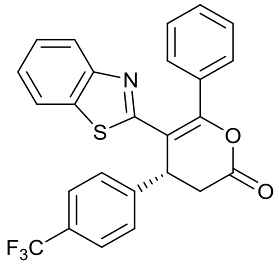

10B

^{1}H, 500 MHz, $CDCl_3$

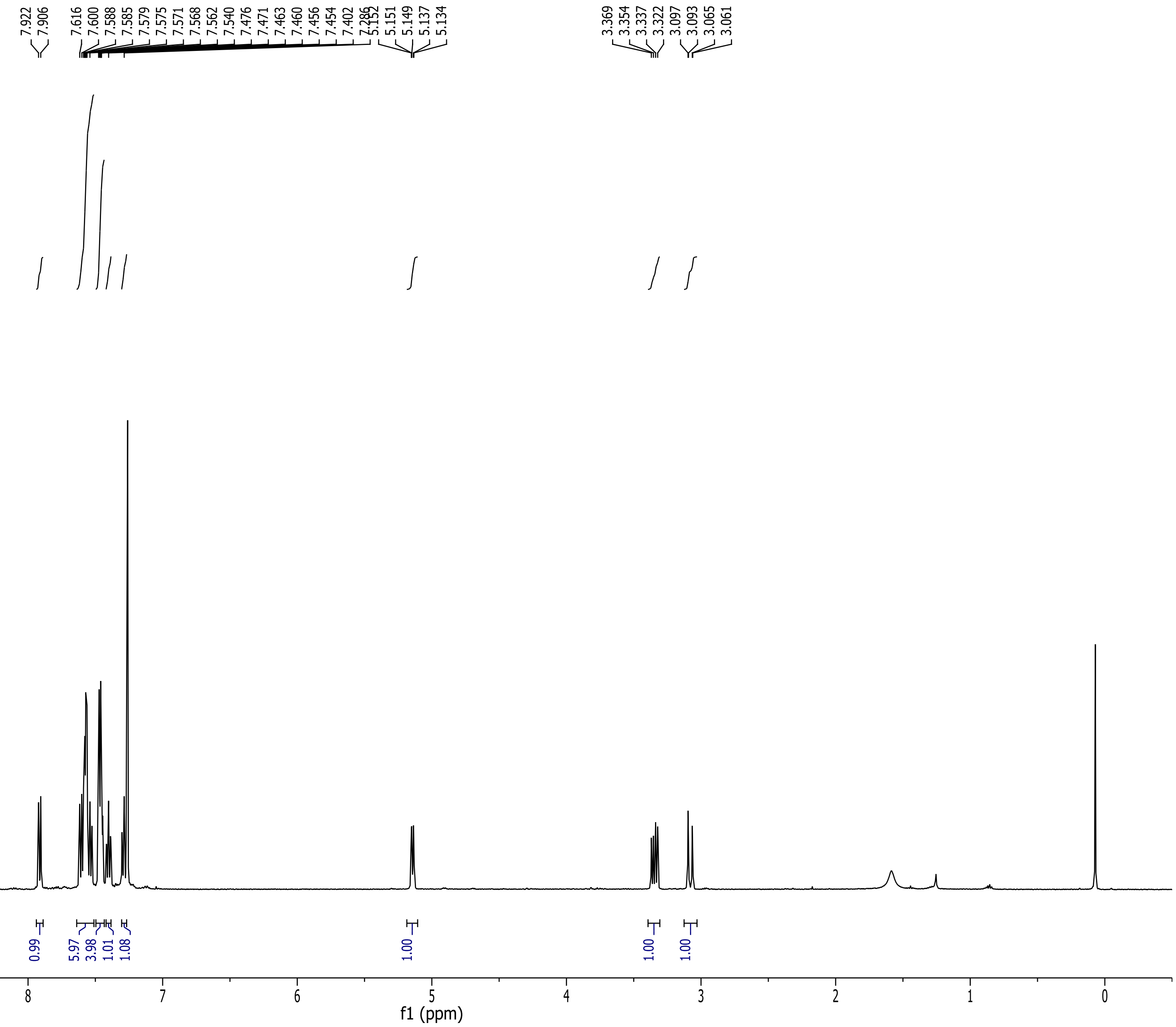

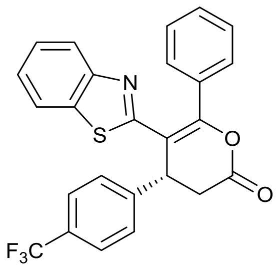

10B

^{13}C, 125 MHz, $CDCl_3$

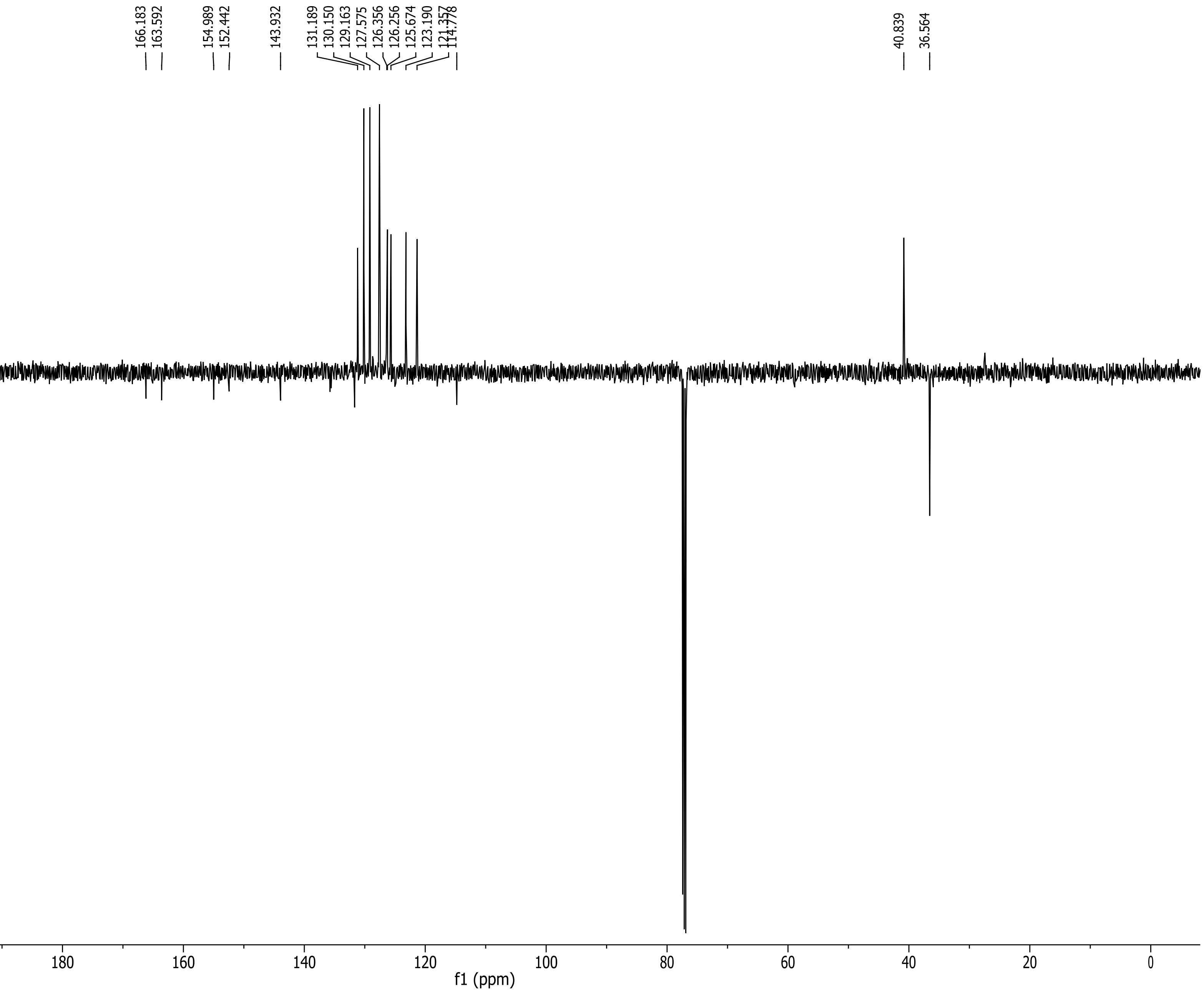

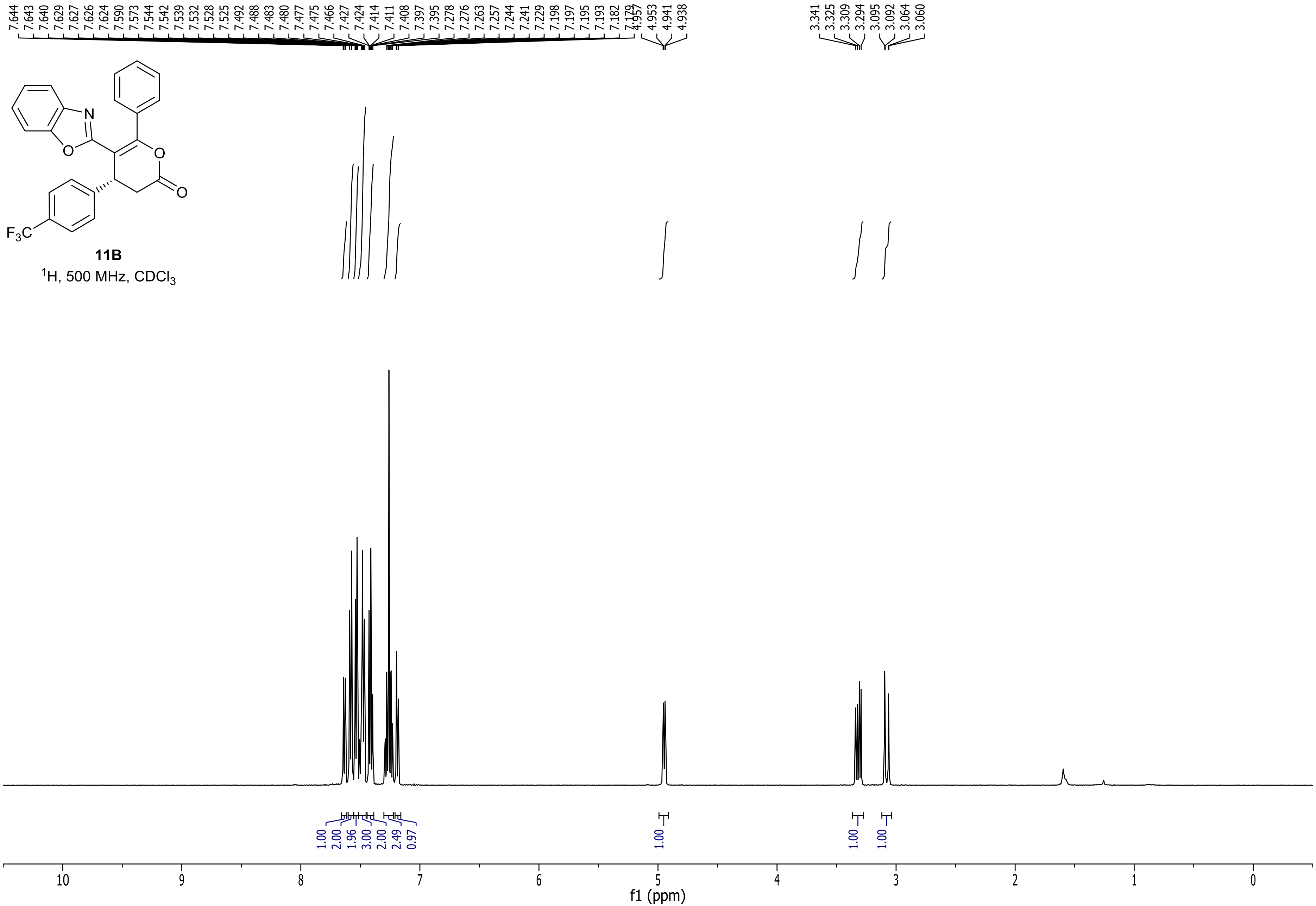

11B

^{1}H, 500 MHz, $CDCl_3$

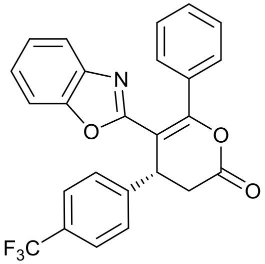

11B

^{13}C, 125 MHz, $CDCl_3$

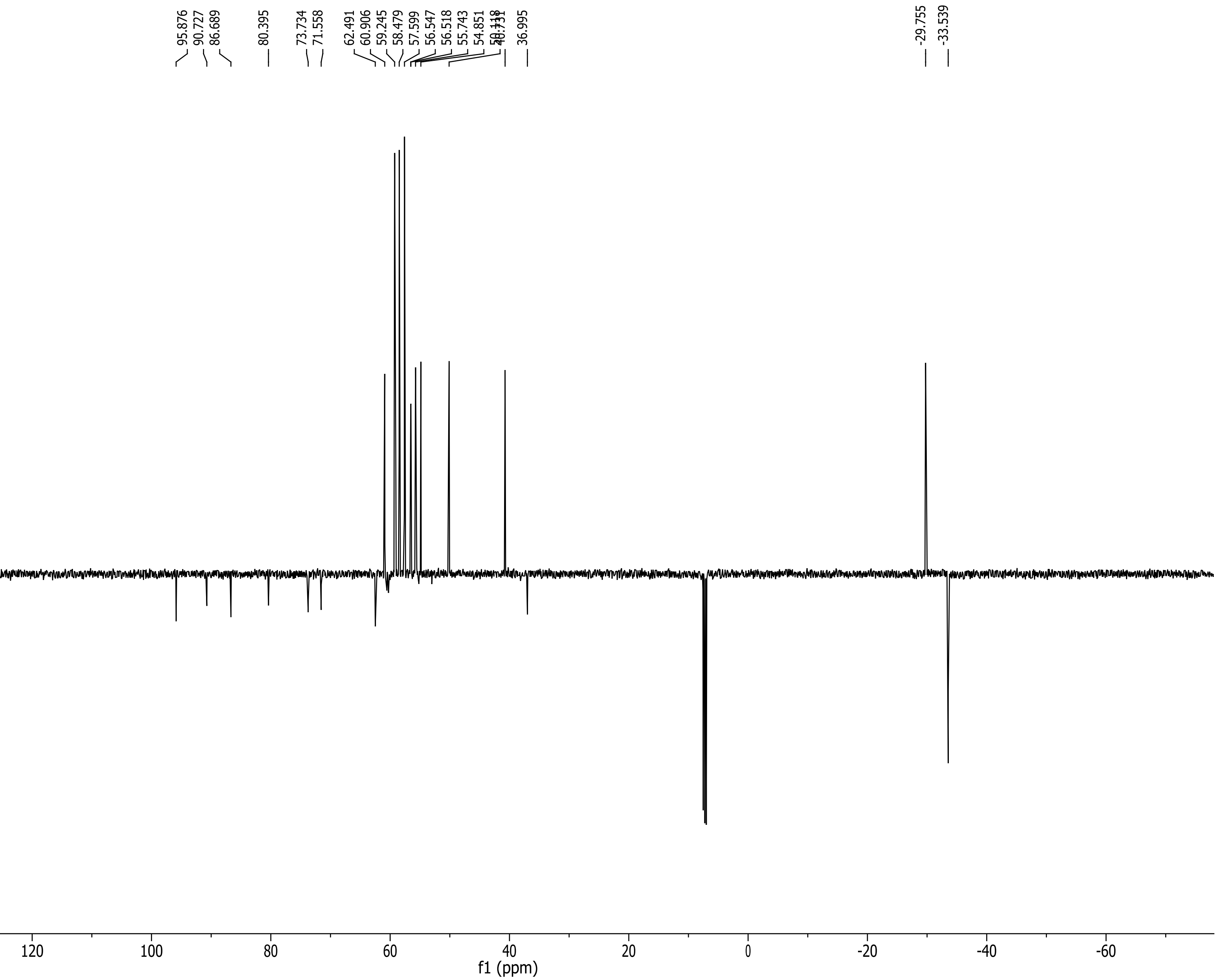

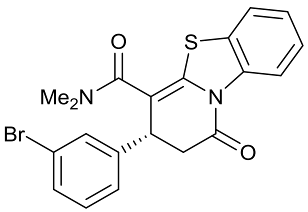

12A
^{1}H, 500 MHz, $CDCl_3$

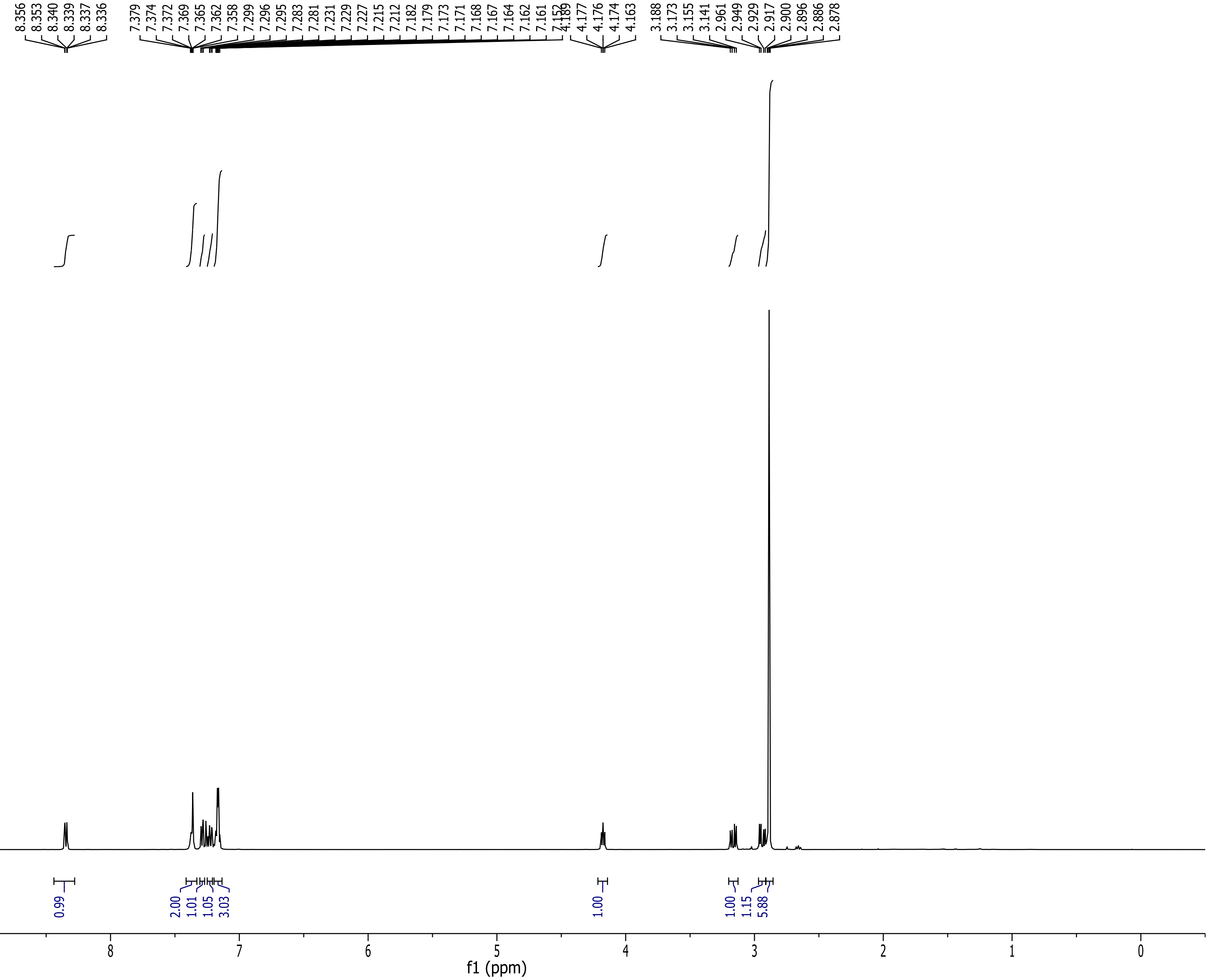

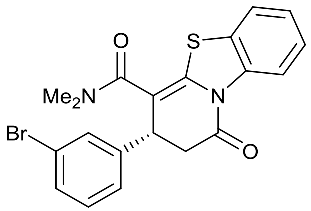

12A

^{13}C, 75 MHz, $CDCl_3$

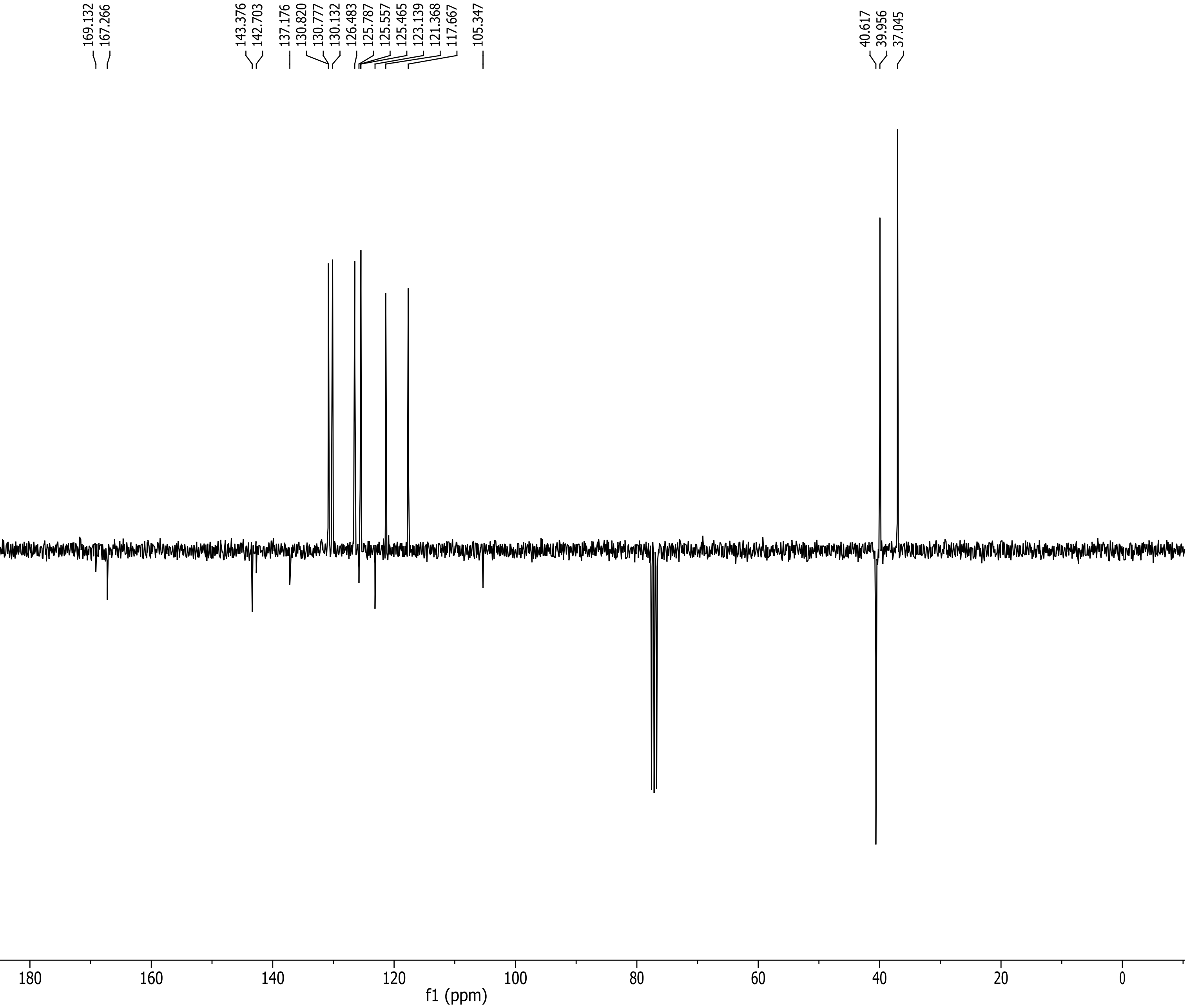

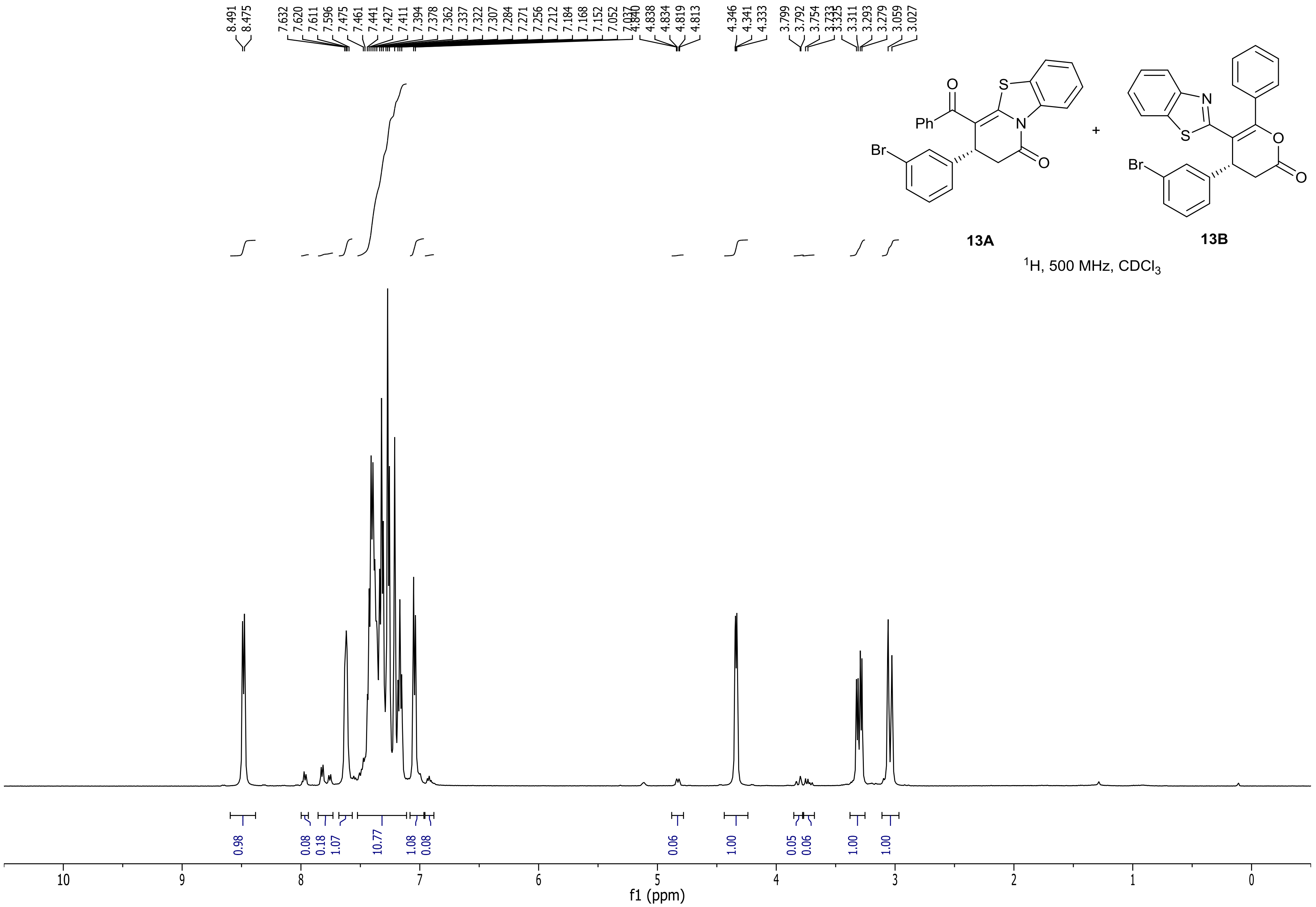
13A
13B
[1]H, 500 MHz, CDCl3
f1 (ppm)

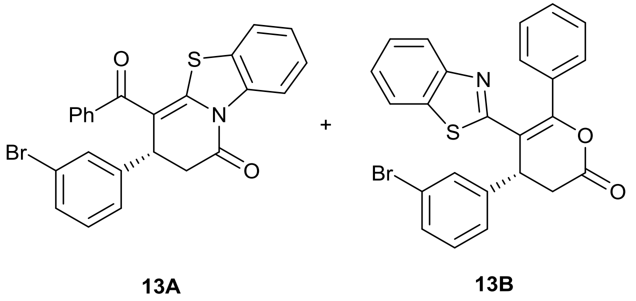

^{13}C, 125 MHz, $CDCl_3$

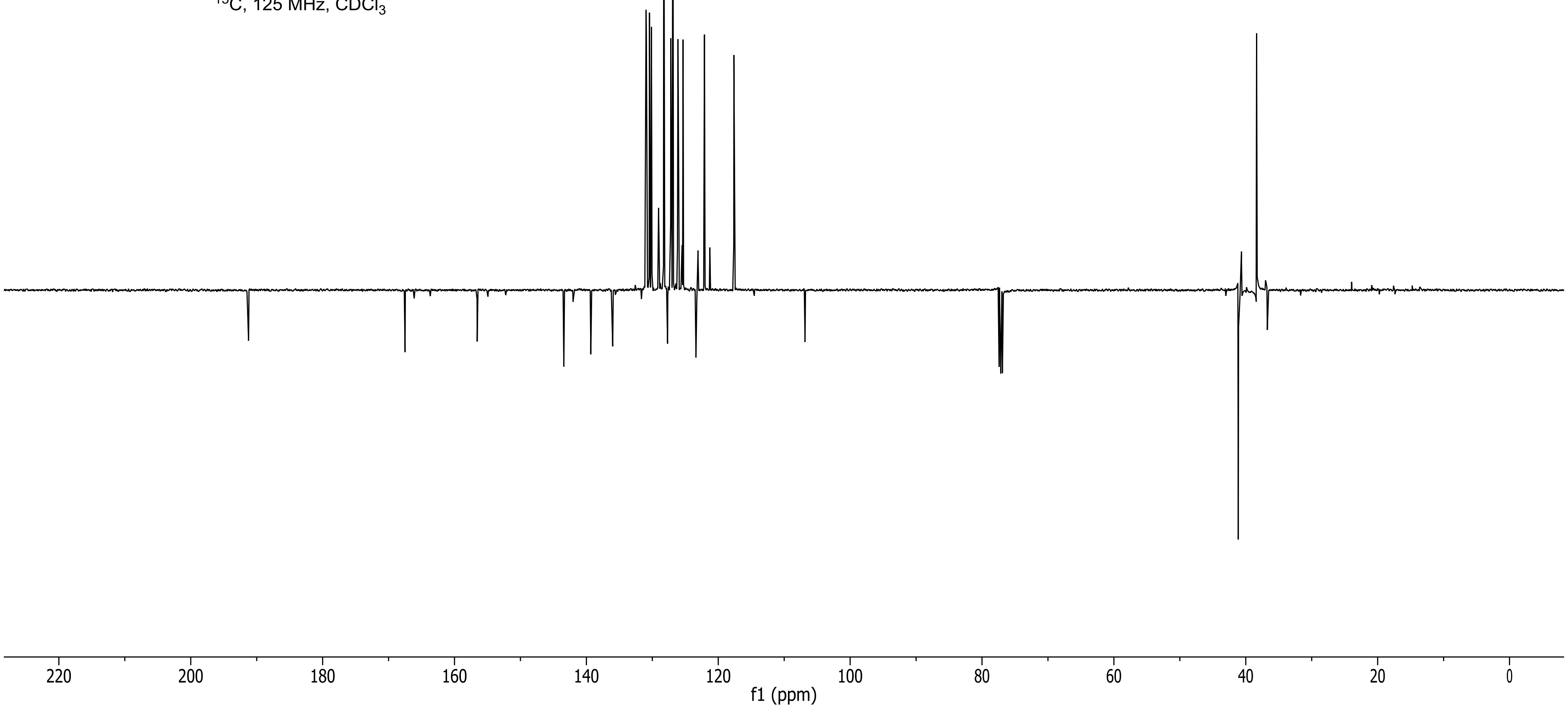

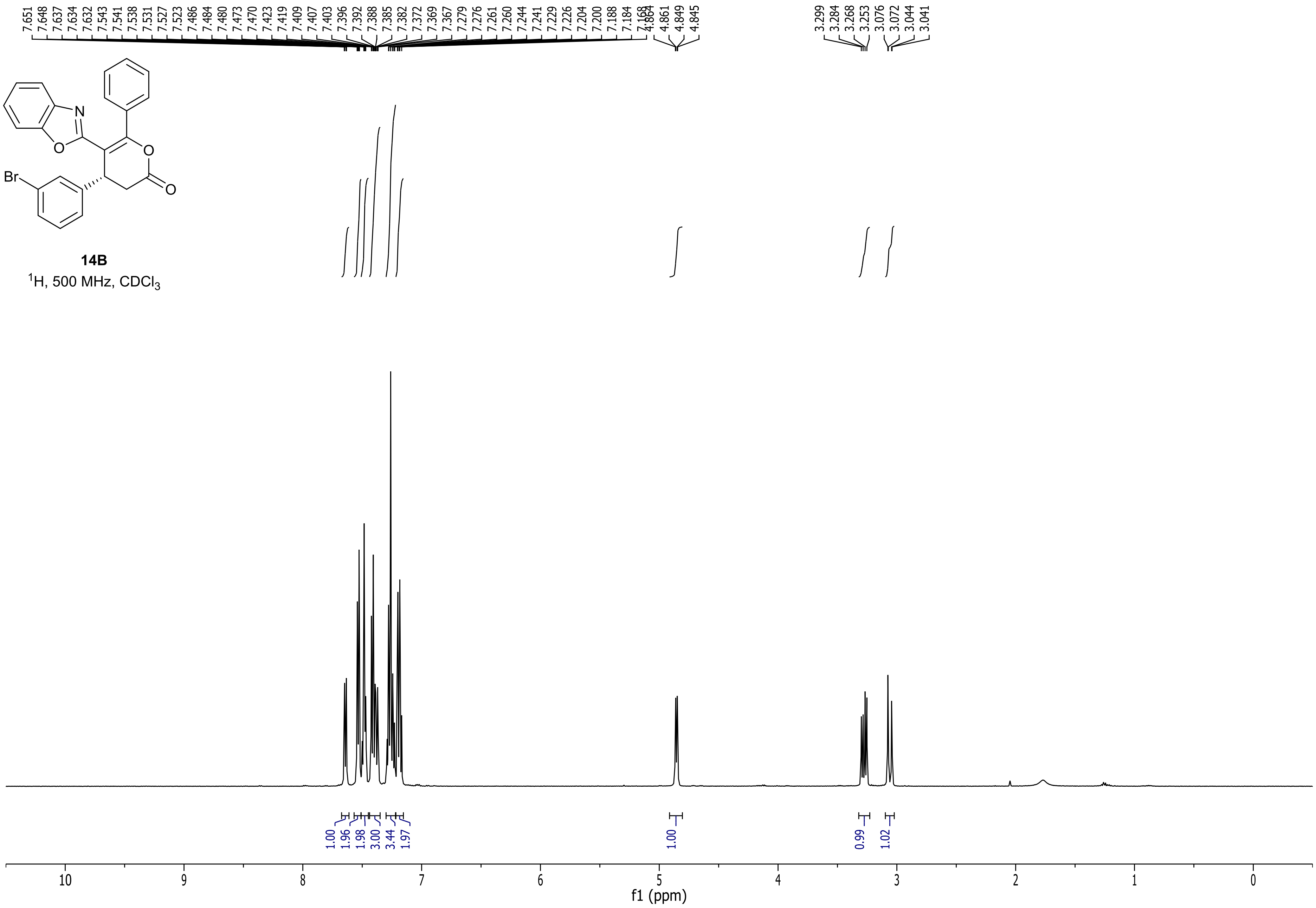

14B

^1H, 500 MHz, $CDCl_3$

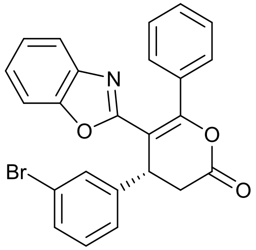

14B

^{13}C, 100 MHz, $CDCl_3$

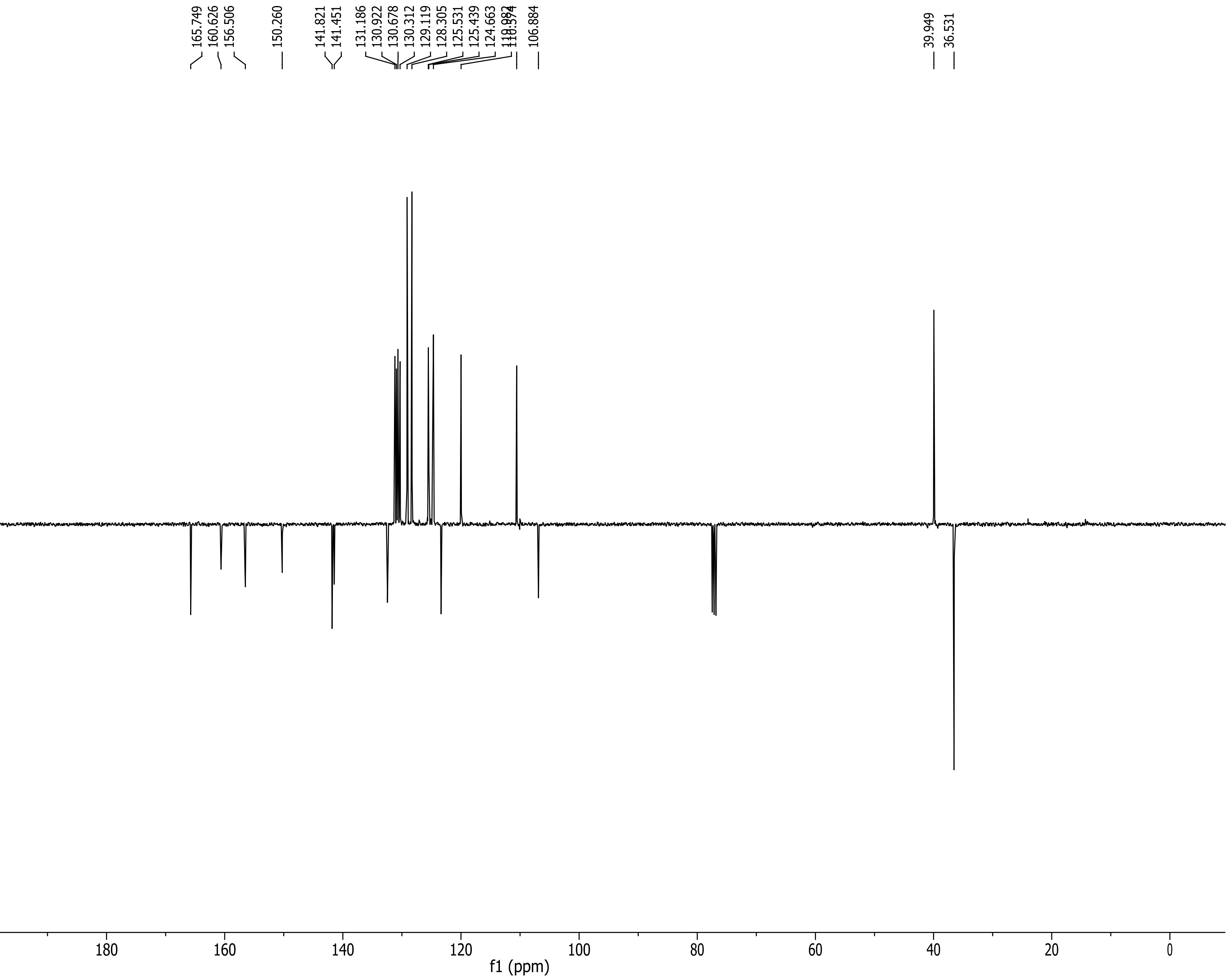

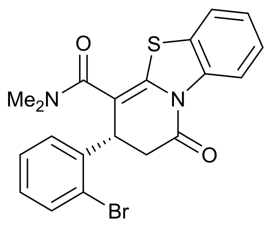

15A

^{1}H, 500 MHz, $CDCl_3$

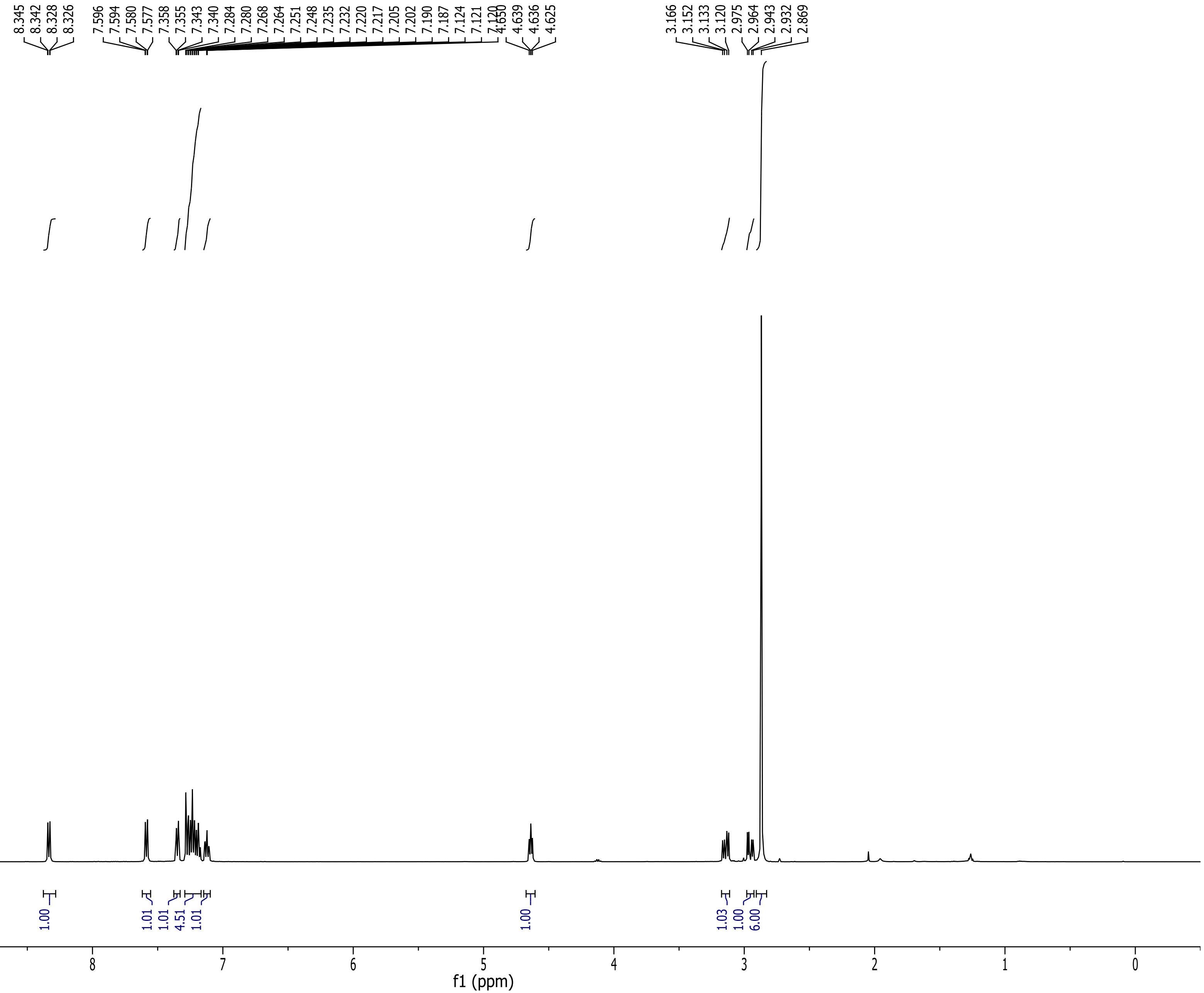

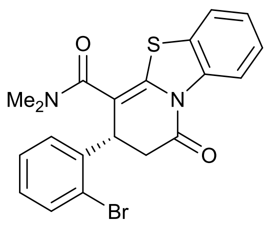

15A

^{13}C, 125 MHz, $CDCl_3$

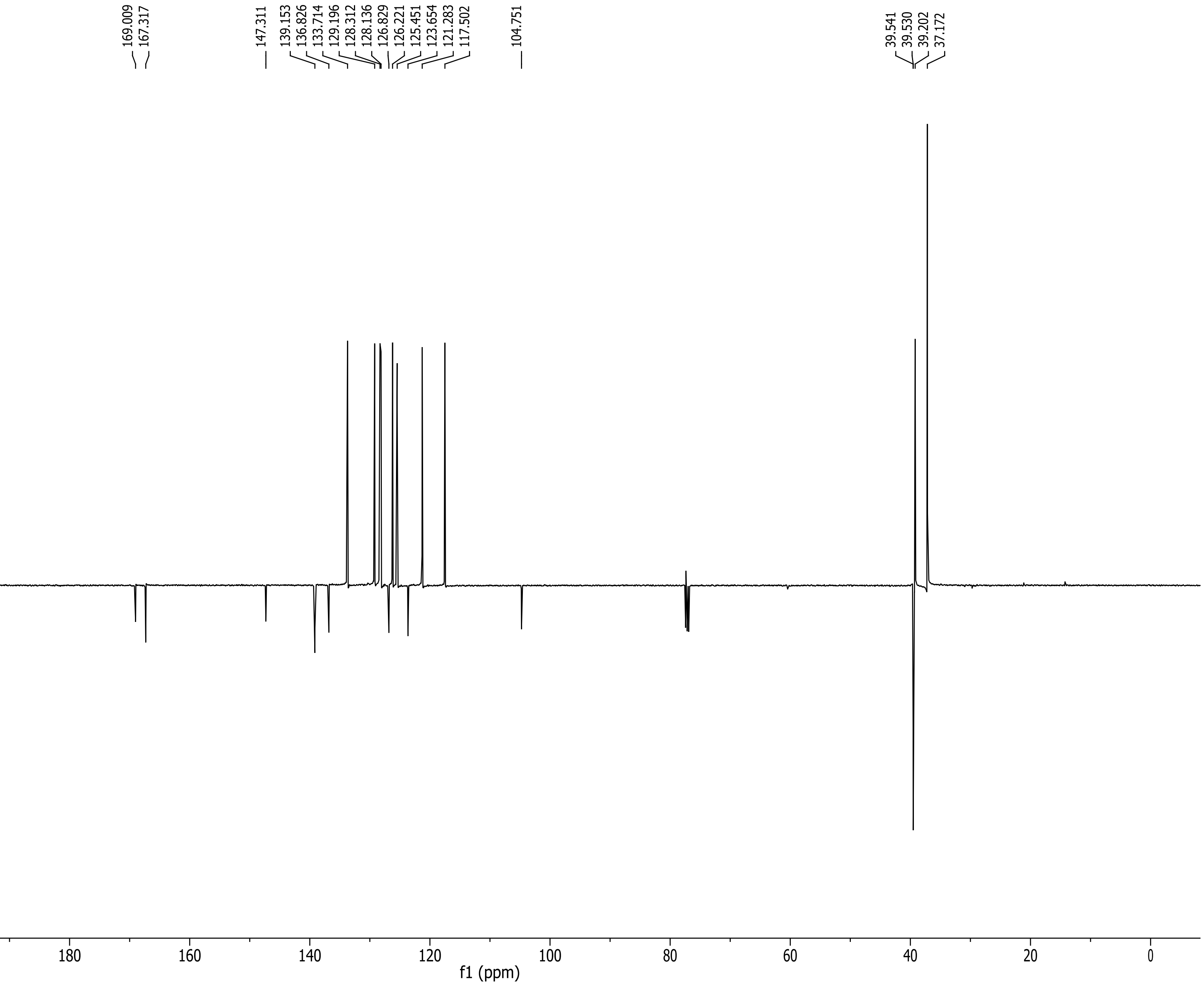

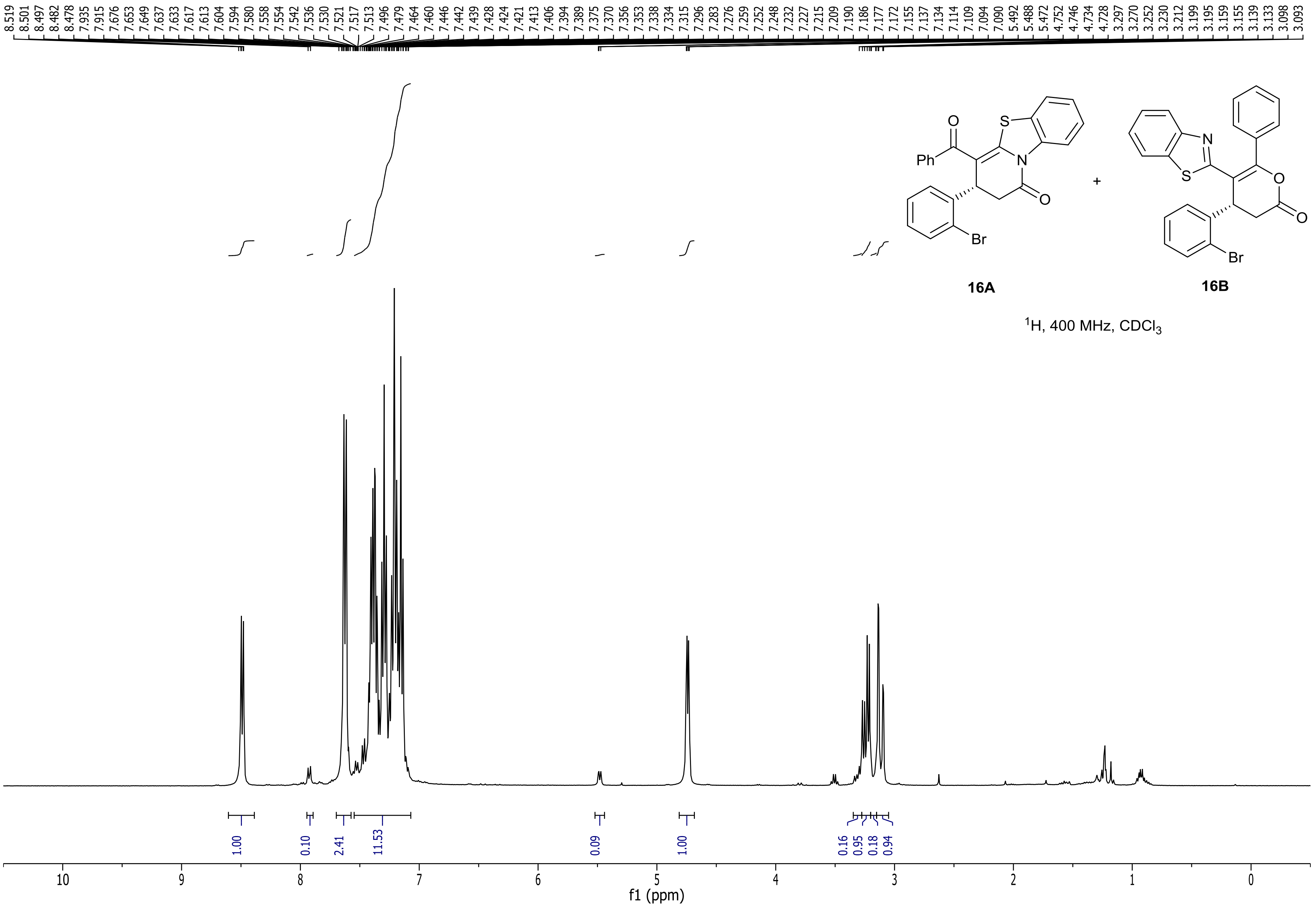
8.519 8.501 8.497 8.482 8.478 7.935 7.915 7.676 7.653 7.649 7.637 7.633 7.617 7.613 7.604 7.594 7.580 7.558 7.554 7.542 7.536 7.530 7.521 7.517 7.513 7.496 7.479 7.464 7.460 7.446 7.442 7.439 7.428 7.424 7.421 7.413 7.406 7.394 7.389 7.375 7.370 7.356 7.353 7.338 7.334 7.315 7.296 7.283 7.276 7.259 7.252 7.248 7.232 7.227 7.215 7.209 7.190 7.186 7.177 7.172 7.155 7.137 7.134 7.114 7.109 7.094 7.090 5.492 5.488 5.472 4.752 4.746 4.734 4.728 3.297 3.270 3.252 3.230 3.212 3.199 3.195 3.159 3.155 3.139 3.133 3.098 3.093
Ph
Br
16A
16B
^{1}H, 400 MHz, CDCl$_3$
1.00 0.10 2.41 11.53 0.09 1.00 0.16 0.95 0.18 0.94
10 9 8 7 6 5 4 3 2 1 0
f1 (ppm)

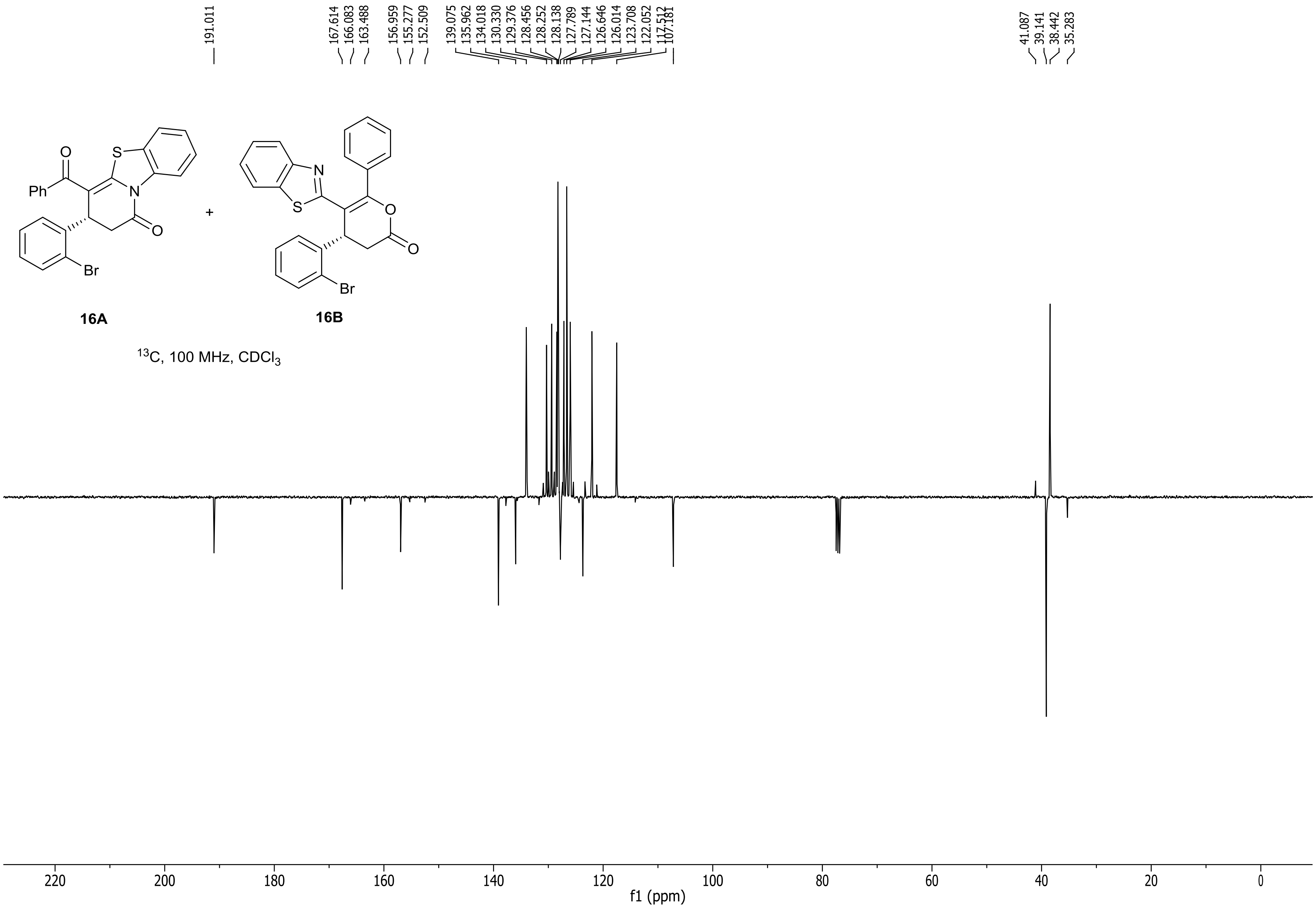
191.011
167.614
166.083
163.488
156.959
155.277
152.509
139.075
135.962
134.018
130.330
129.376
128.456
128.252
128.138
127.789
127.144
126.646
126.014
123.708
122.052
117.512
107.181
41.087
39.141
38.442
35.283
Ph
O
S
N
O
Br
+
N
S
O
O
Br
16A
16B
13C, 100 MHz, CDCl3
220
200
180
160
140
120
100
80
60
40
20
0
f1 (ppm)

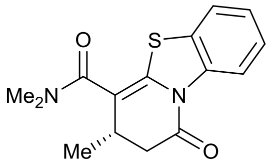

17A

^{1}H, 500 MHz, $CDCl_3$

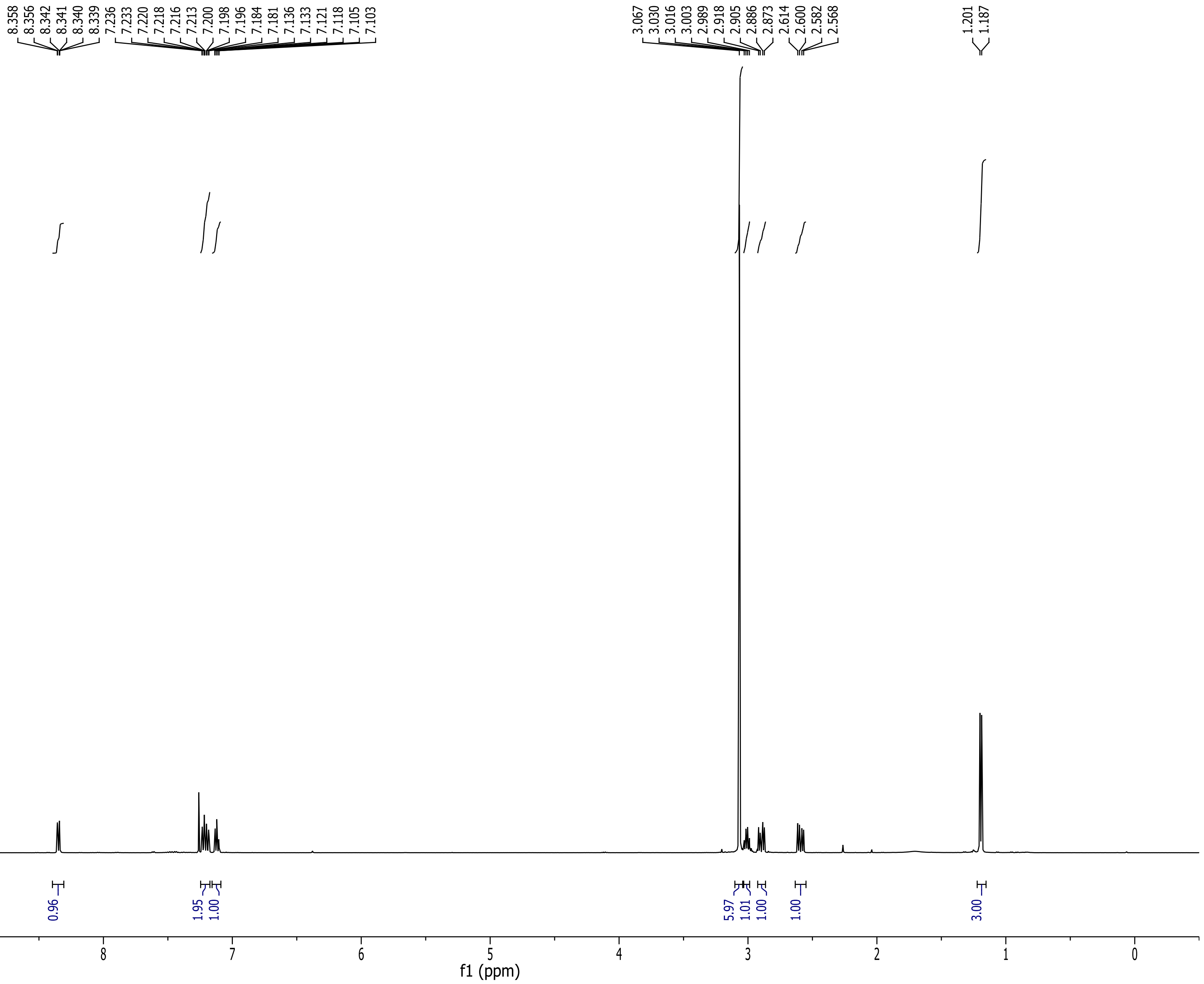

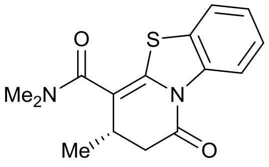

17A

^{13}C, 125 MHz, $CDCl_3$

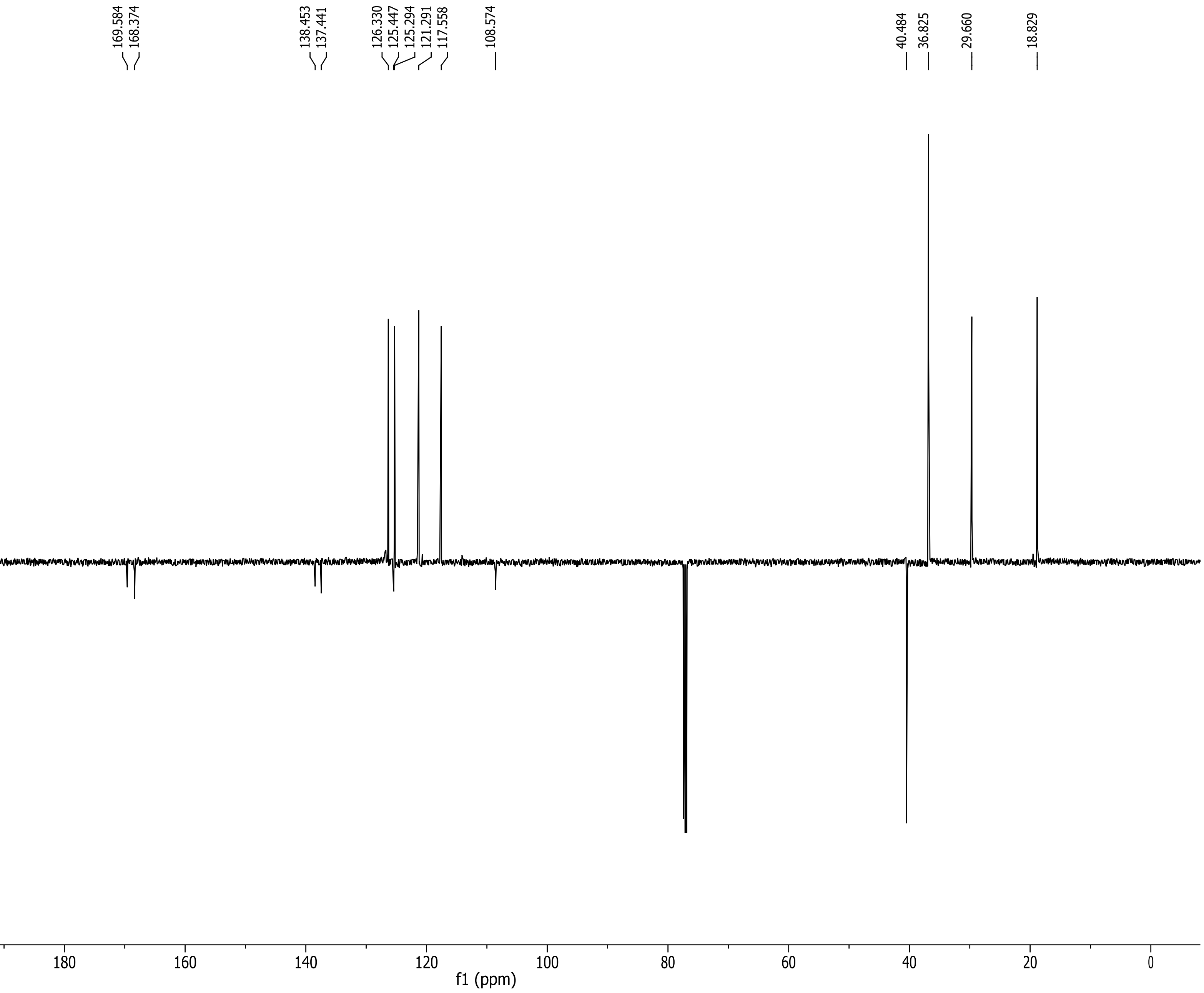

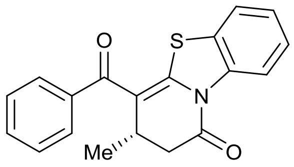

18A

^1H, 400 MHz, $CDCl_3$

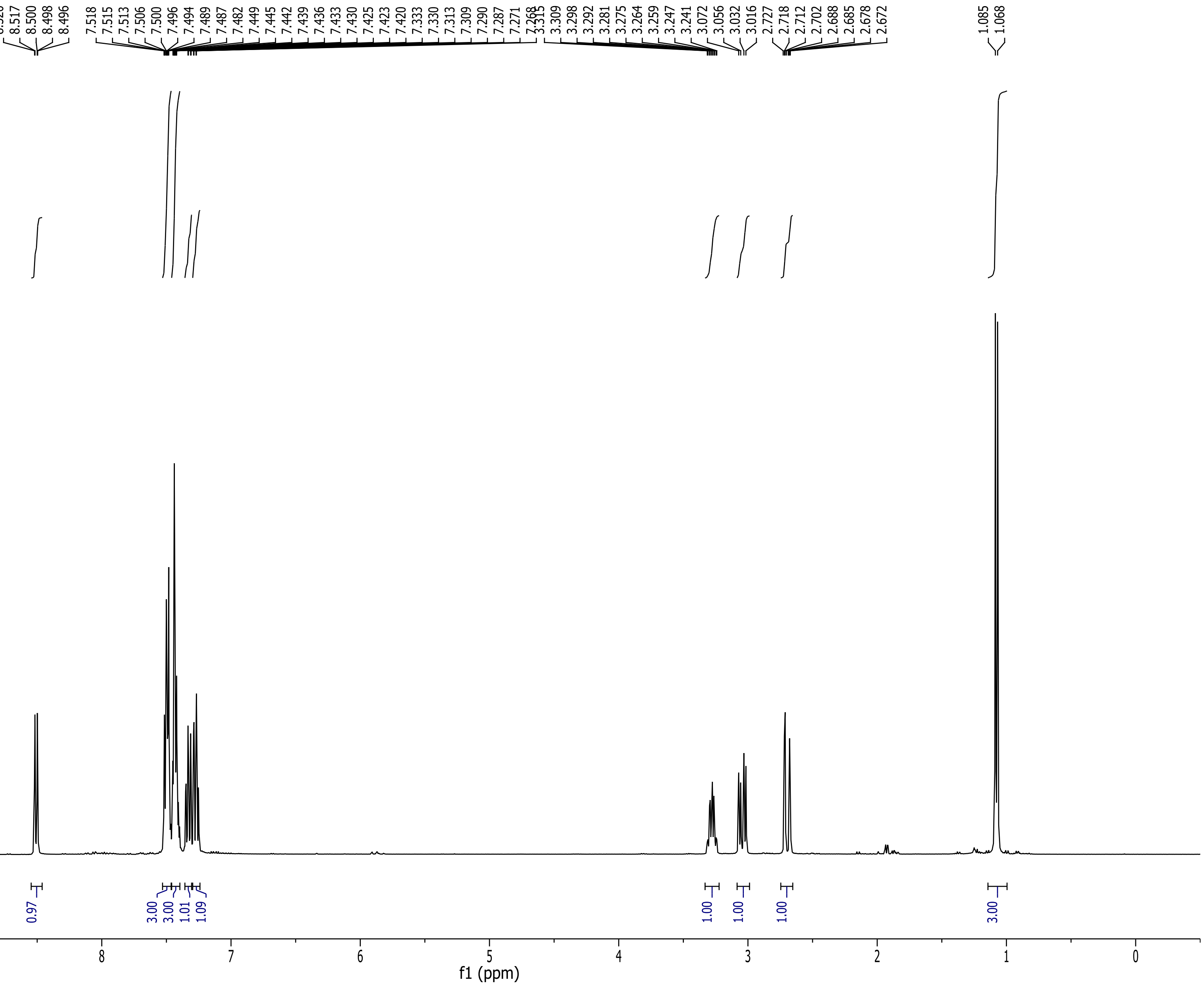

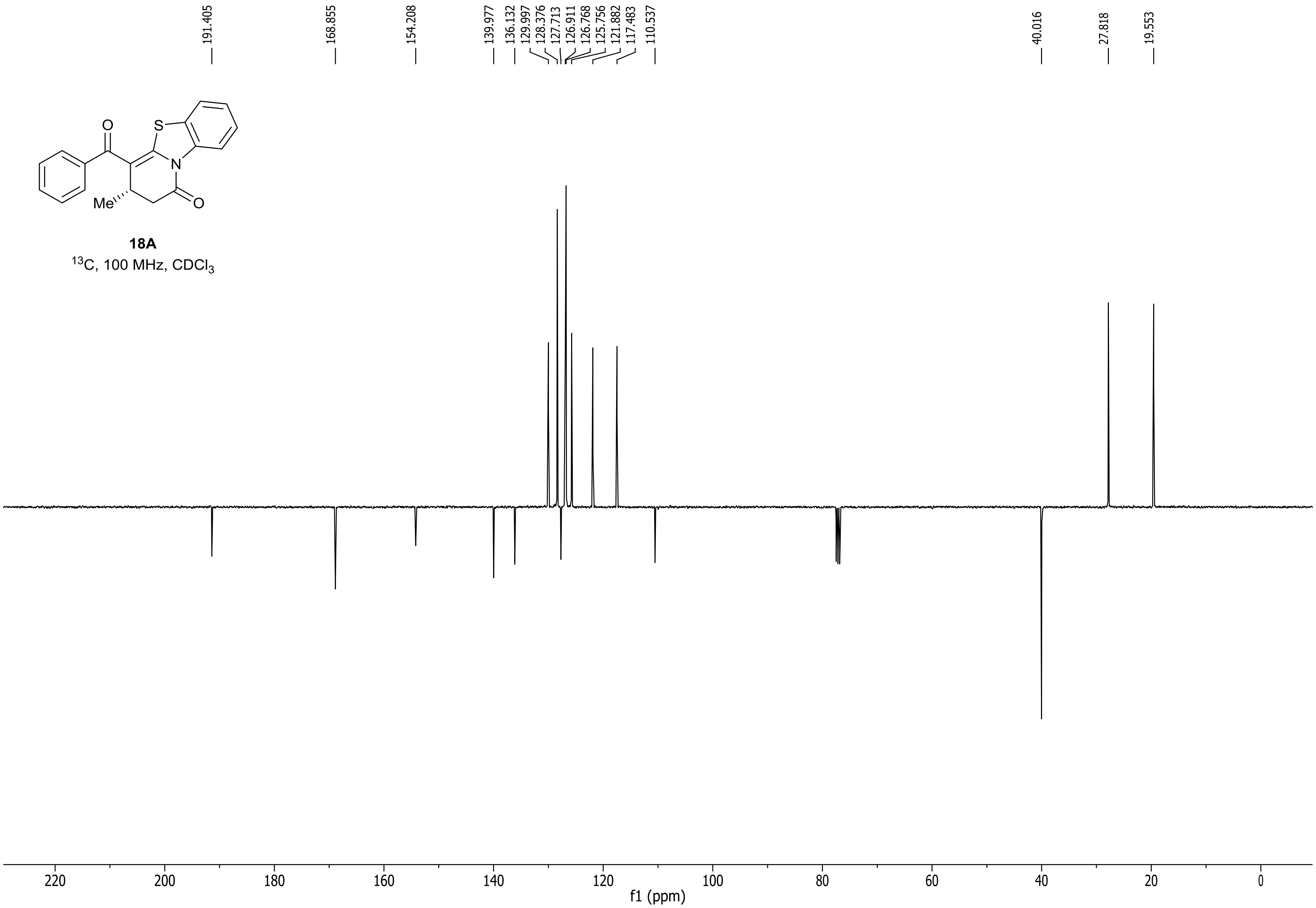

18A
^{13}C, 100 MHz, $CDCl_3$

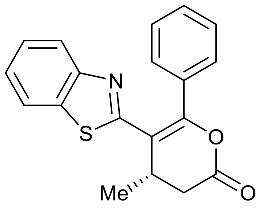

18B

^{1}H, 500 MHz, $CDCl_3$

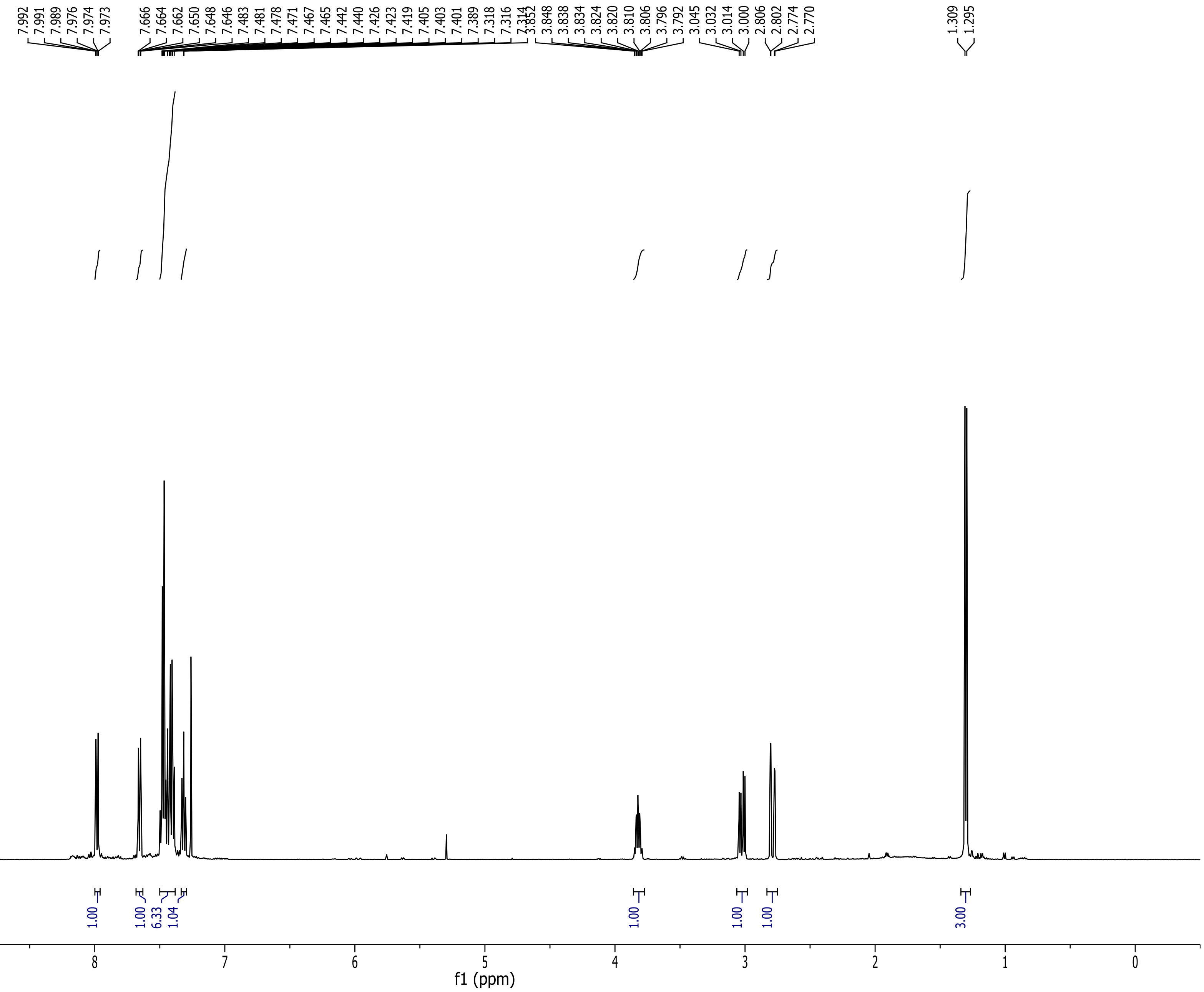

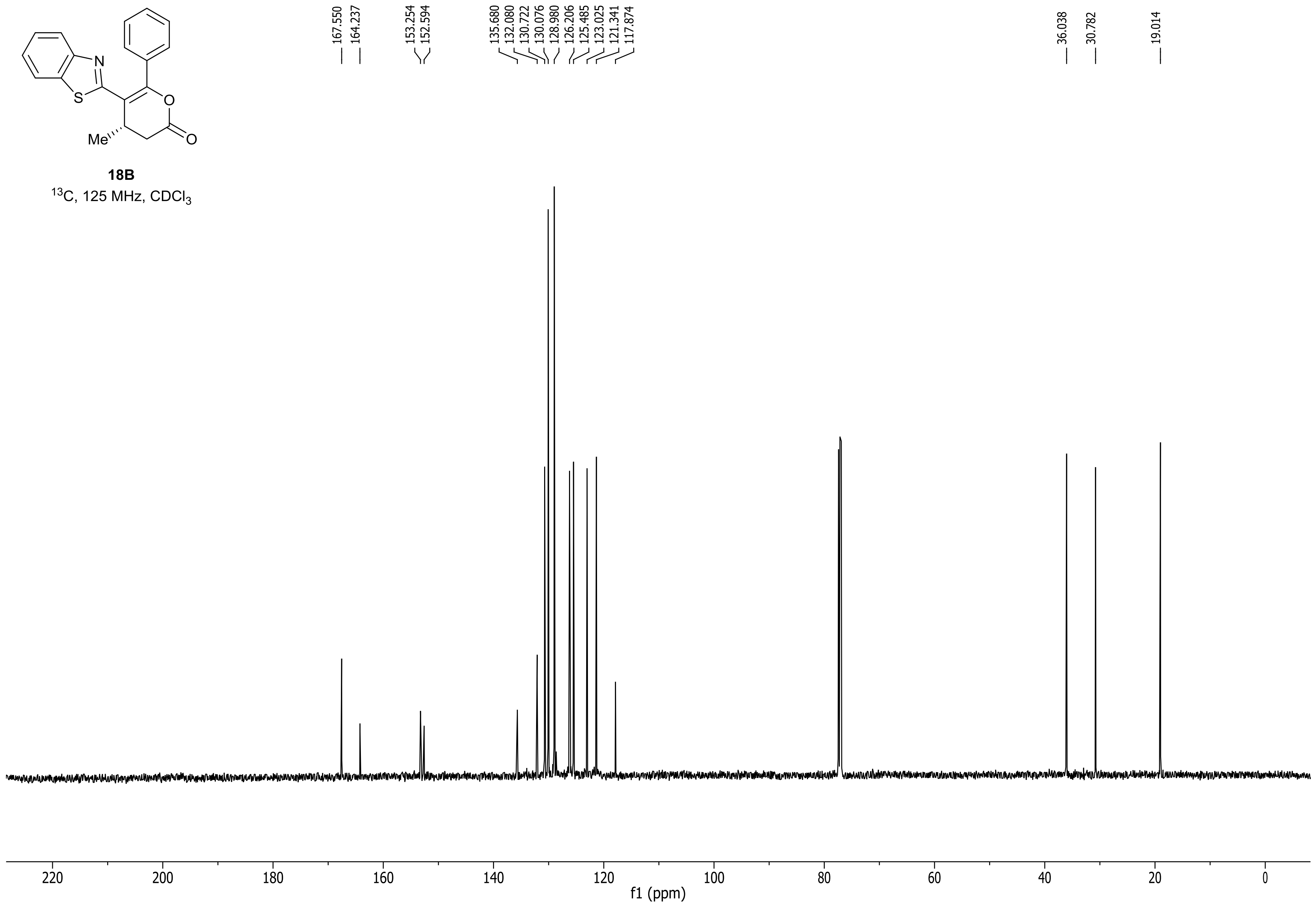
N
S
O
O
Me
18B
^{13}C, 125 MHz, CDCl$_3$
167.550
164.237
153.254
152.594
135.680
132.080
130.722
130.076
128.980
126.206
125.485
123.025
121.341
117.874
36.038
30.782
19.014
220
200
180
160
140
120
100
80
60
40
20
0
f1 (ppm)

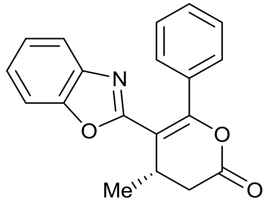

19B
^{1}H, 400 MHz, $CDCl_3$

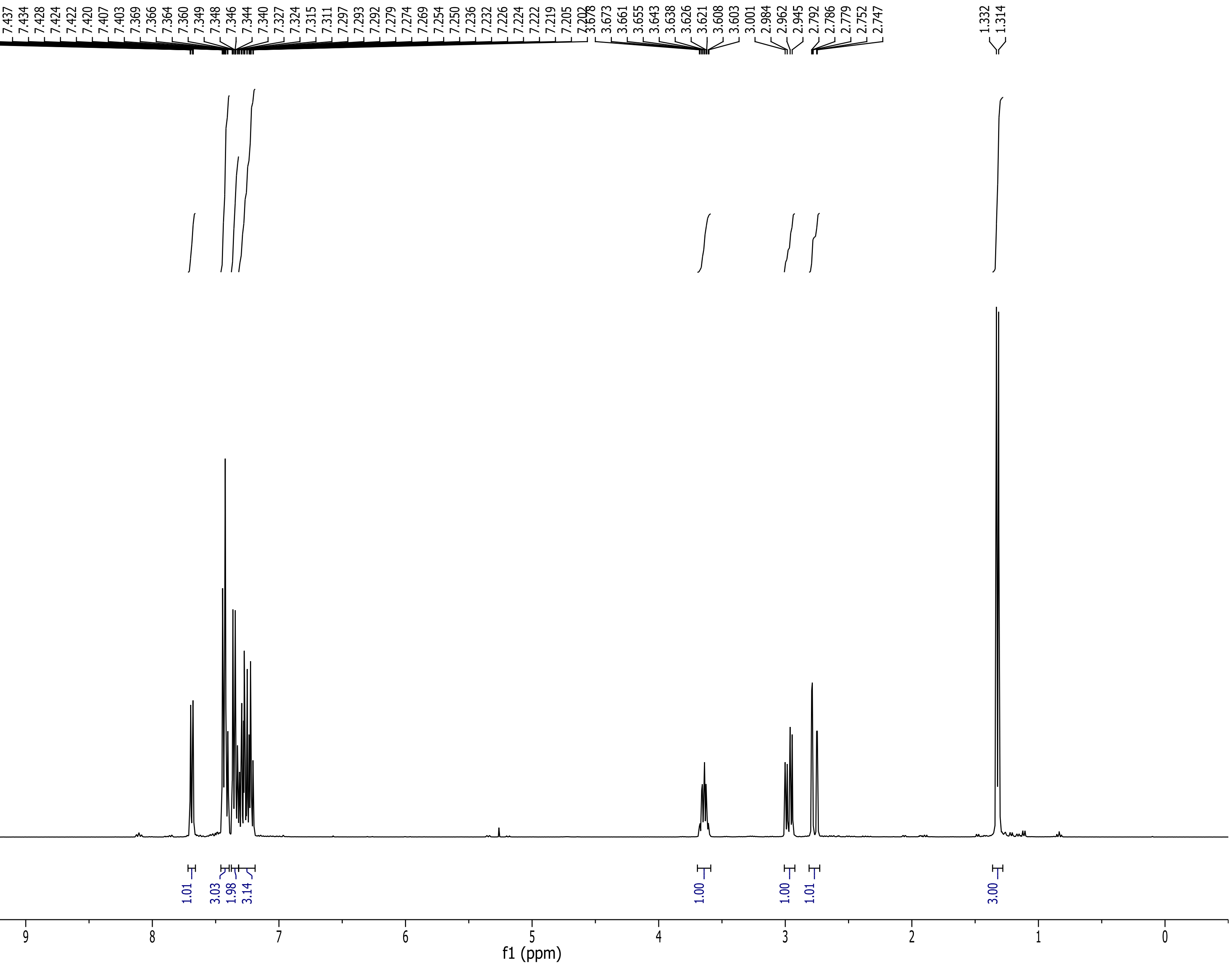

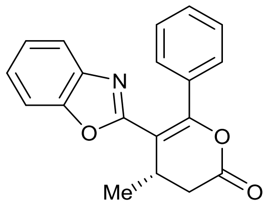

19B
^{13}C, 100 MHz, $CDCl_3$

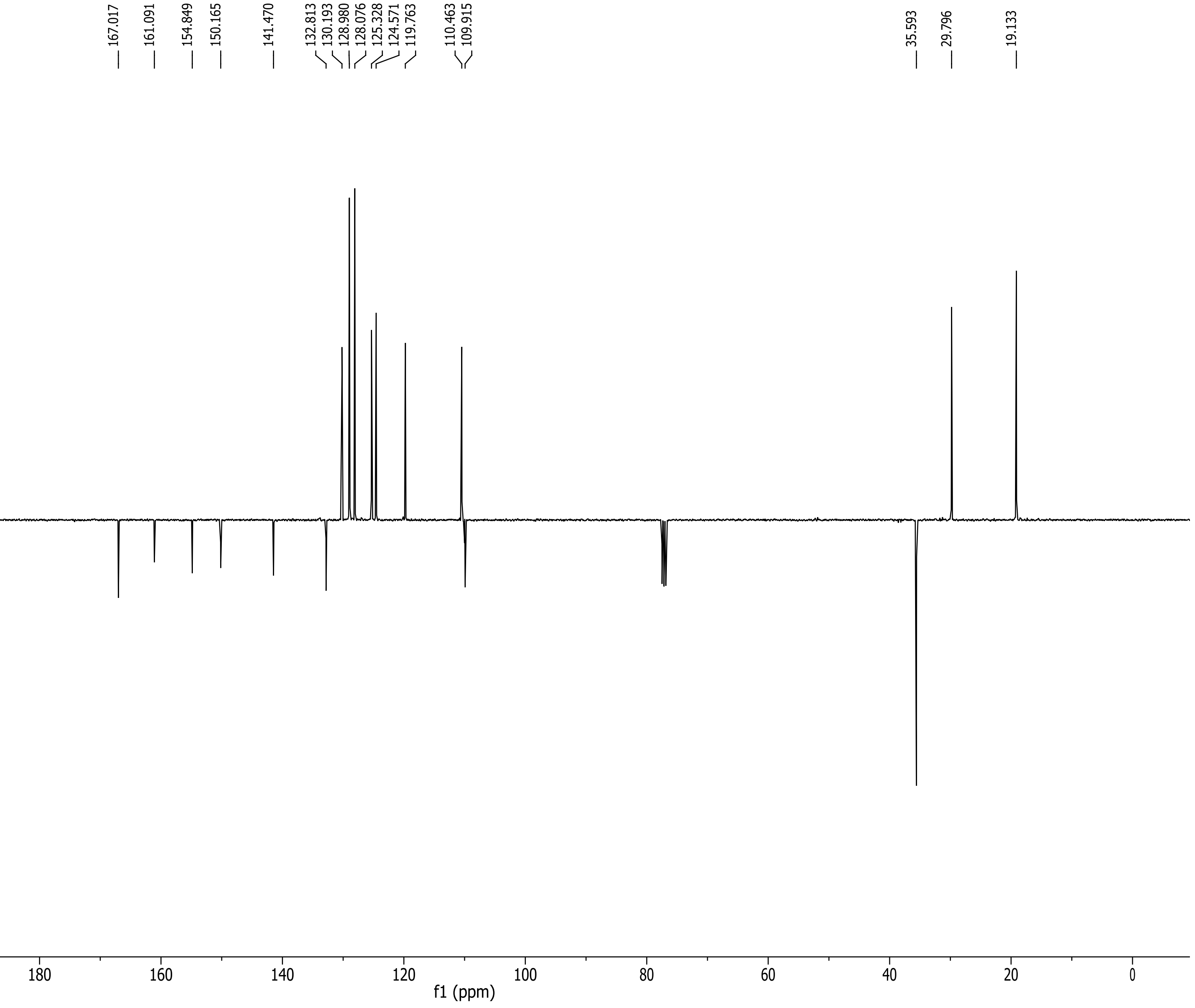

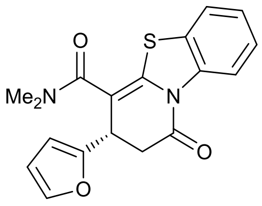

20A

^1H, 500 MHz, $CDCl_3$

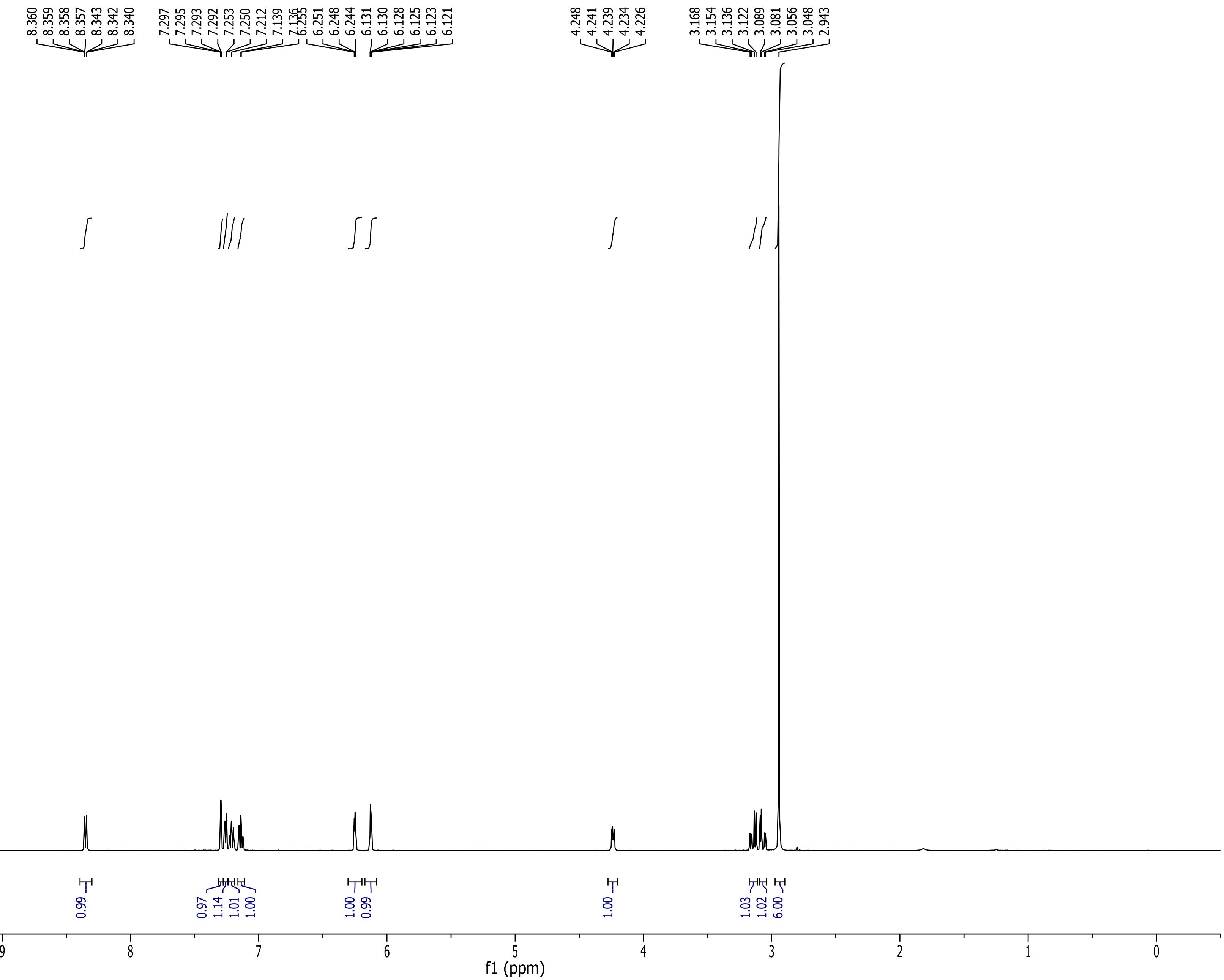

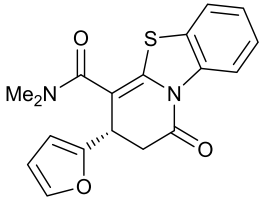

20A

^{13}C, 125 MHz, $CDCl_3$

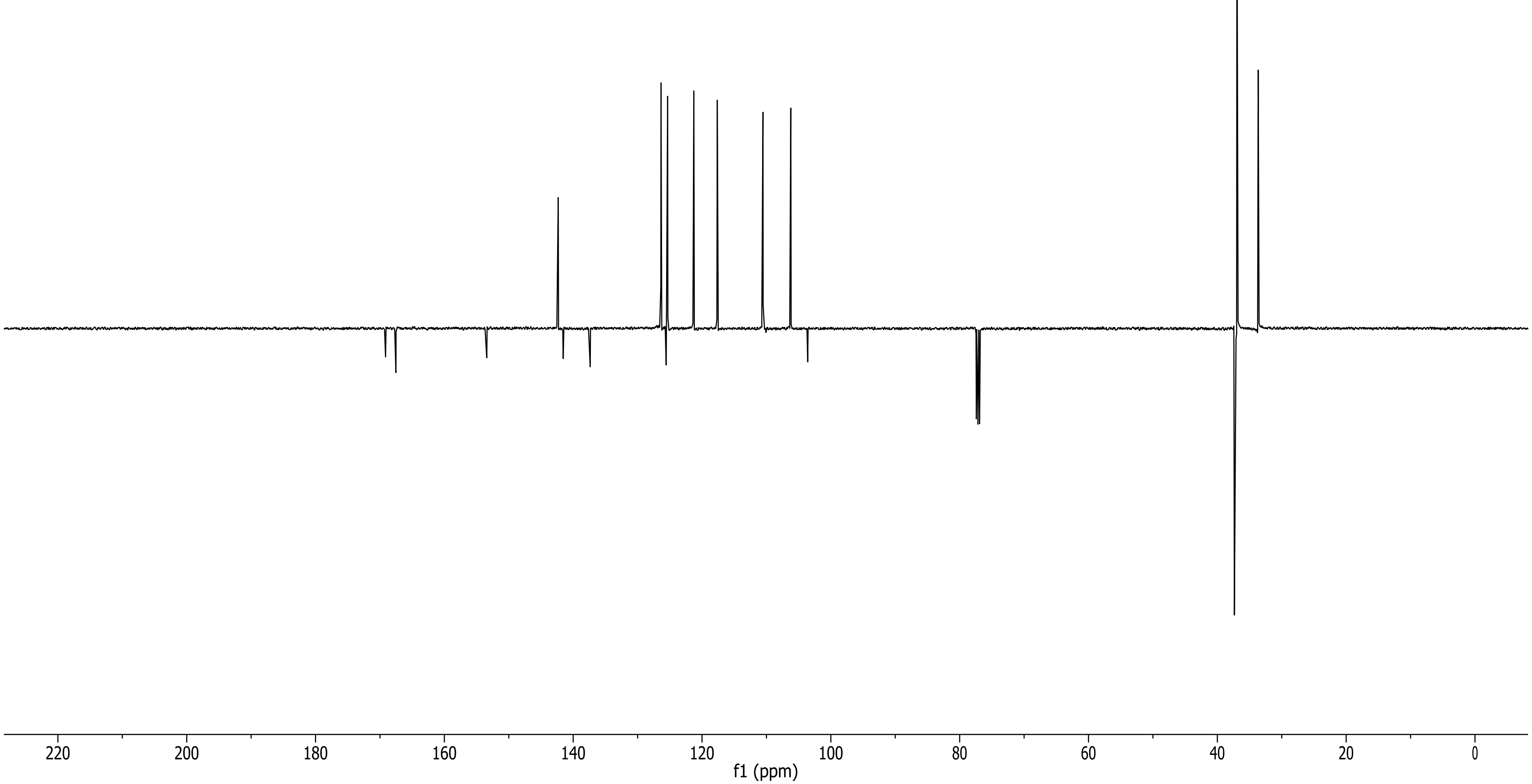

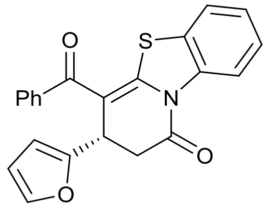

21A

^{1}H, 300 MHz, $CDCl_3$

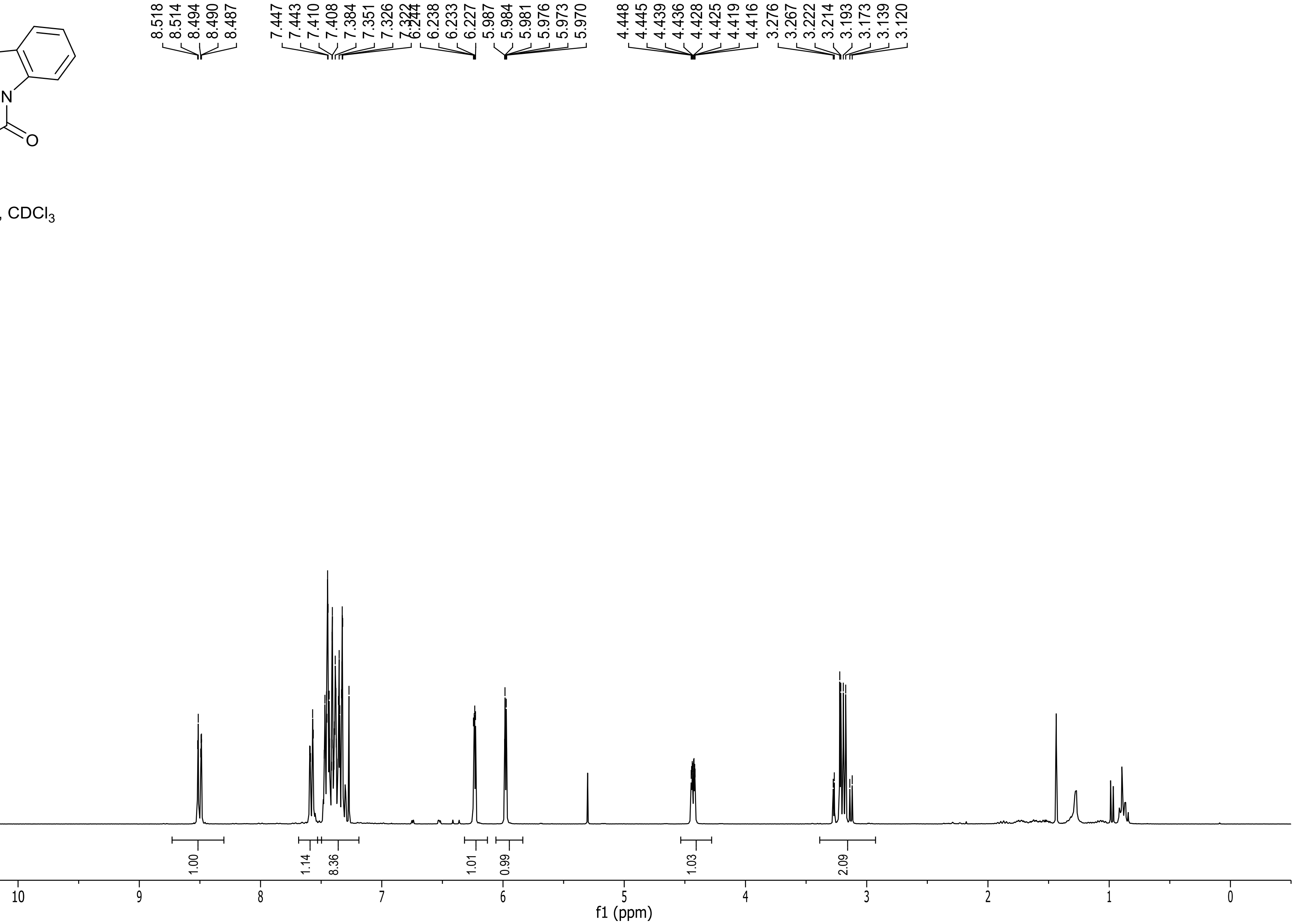

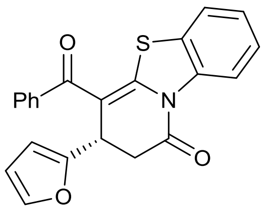
21A
^{13}C, 100 MHz, $CDCl_3$

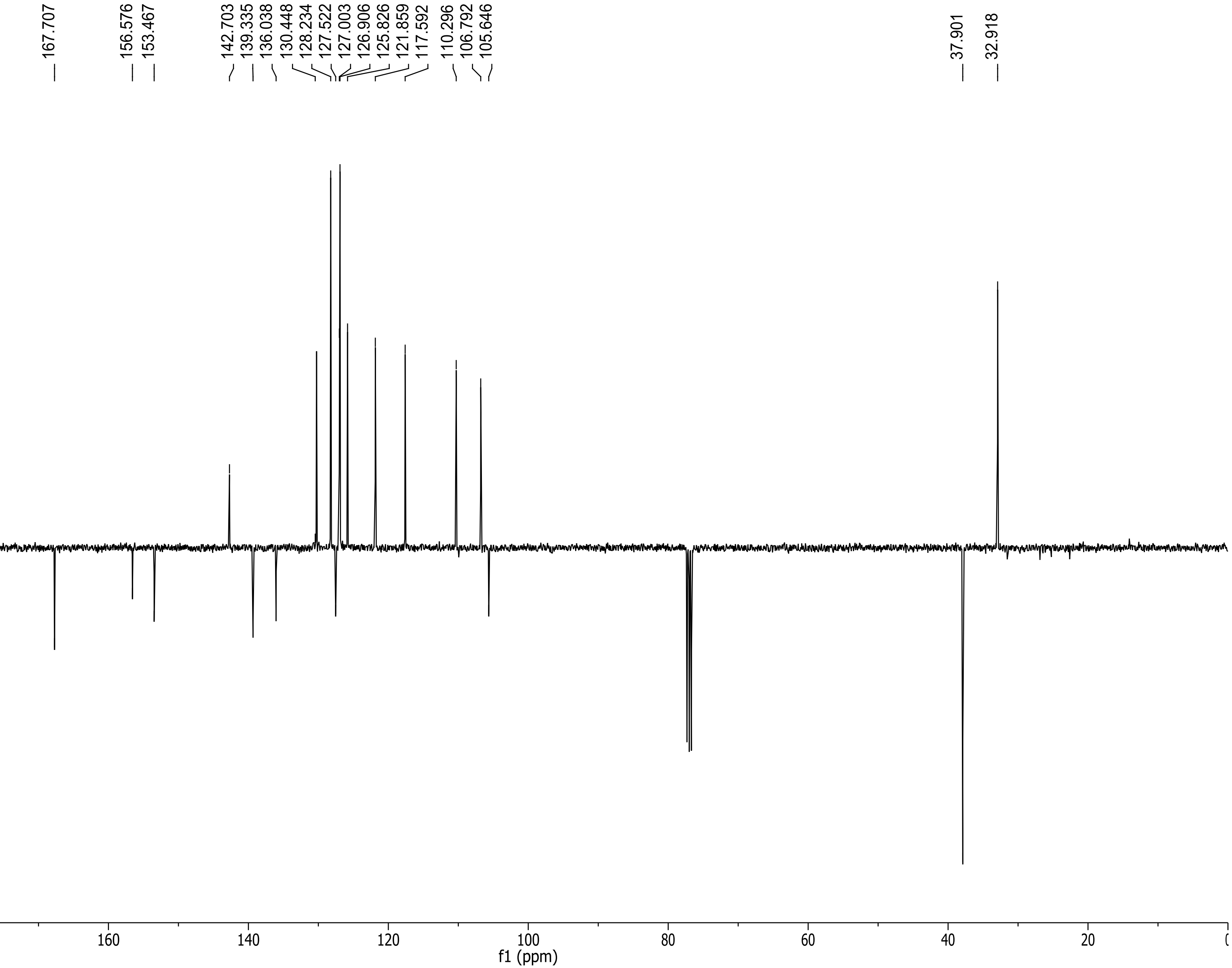

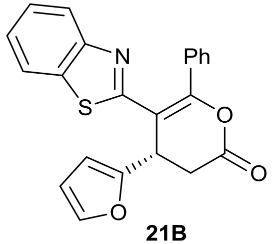

21B

^1H, 400 MHz, $CDCl_3$

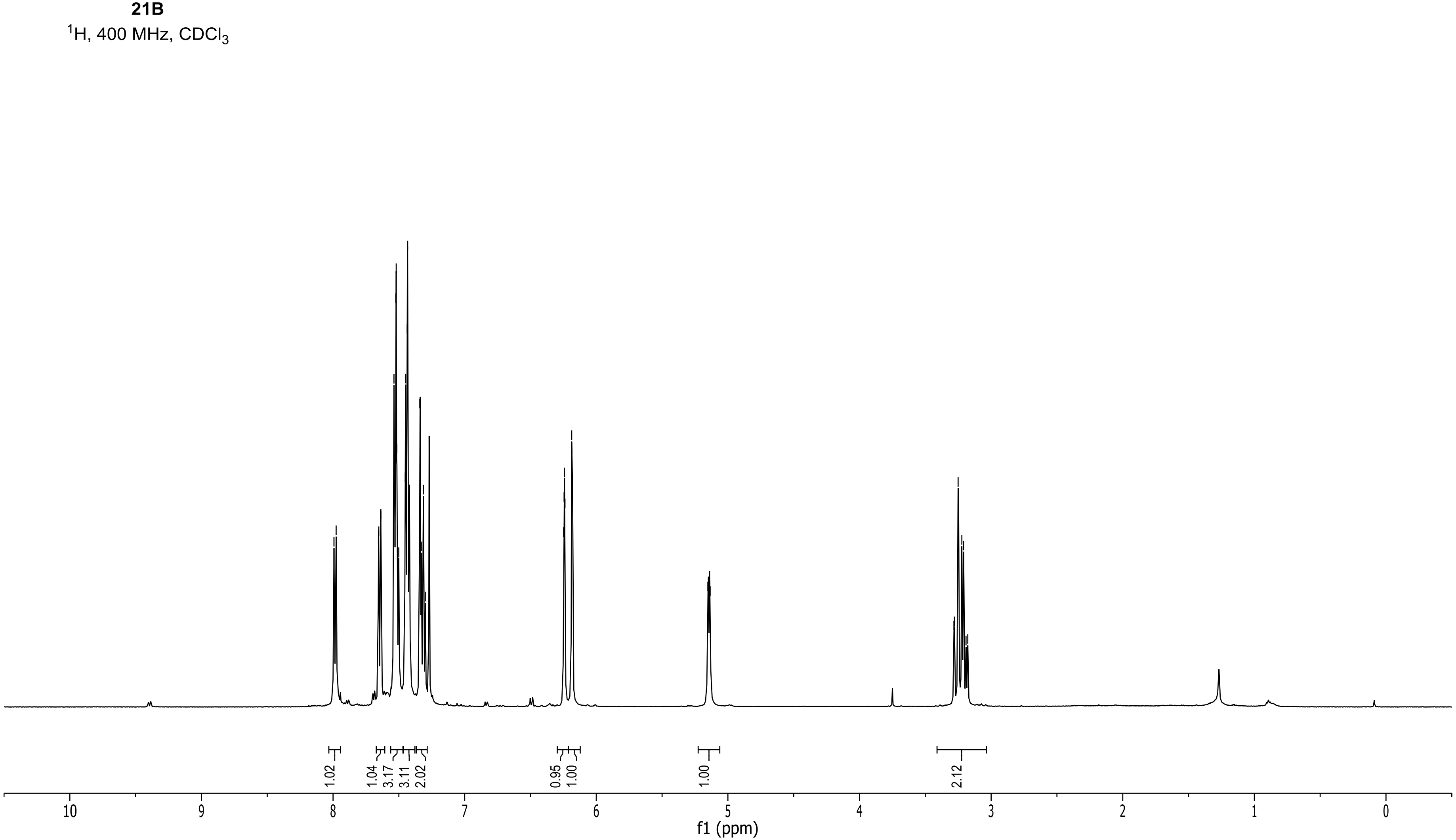

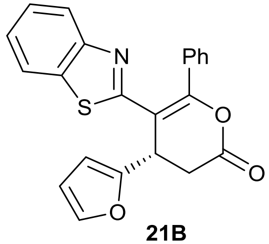

21B

^{13}C, 100 MHz, $CDCl_3$

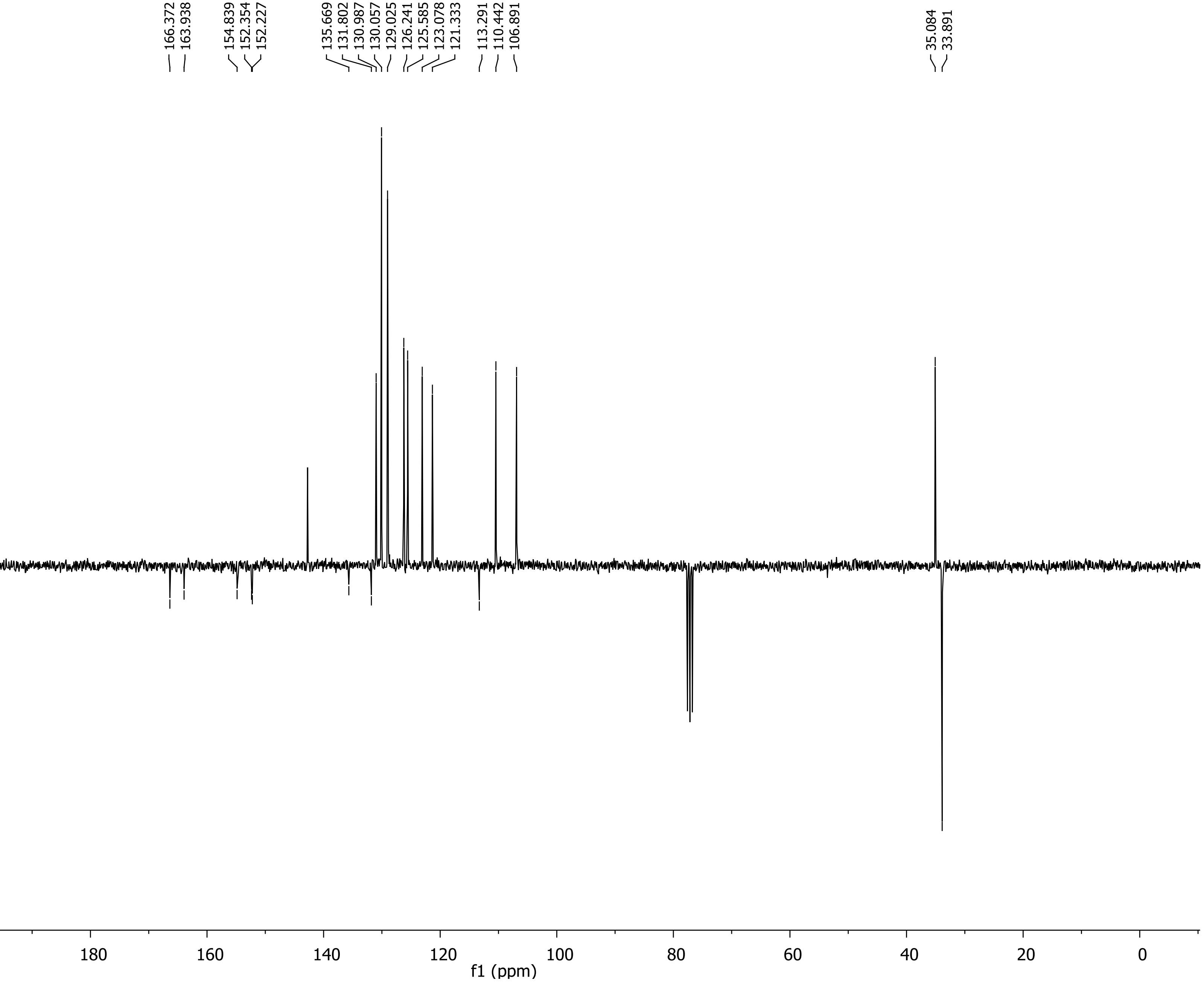

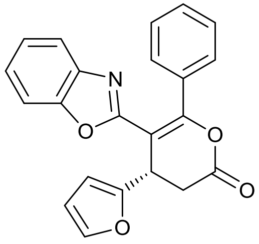

22B
^{1}H, 500 MHz, $CDCl_3$

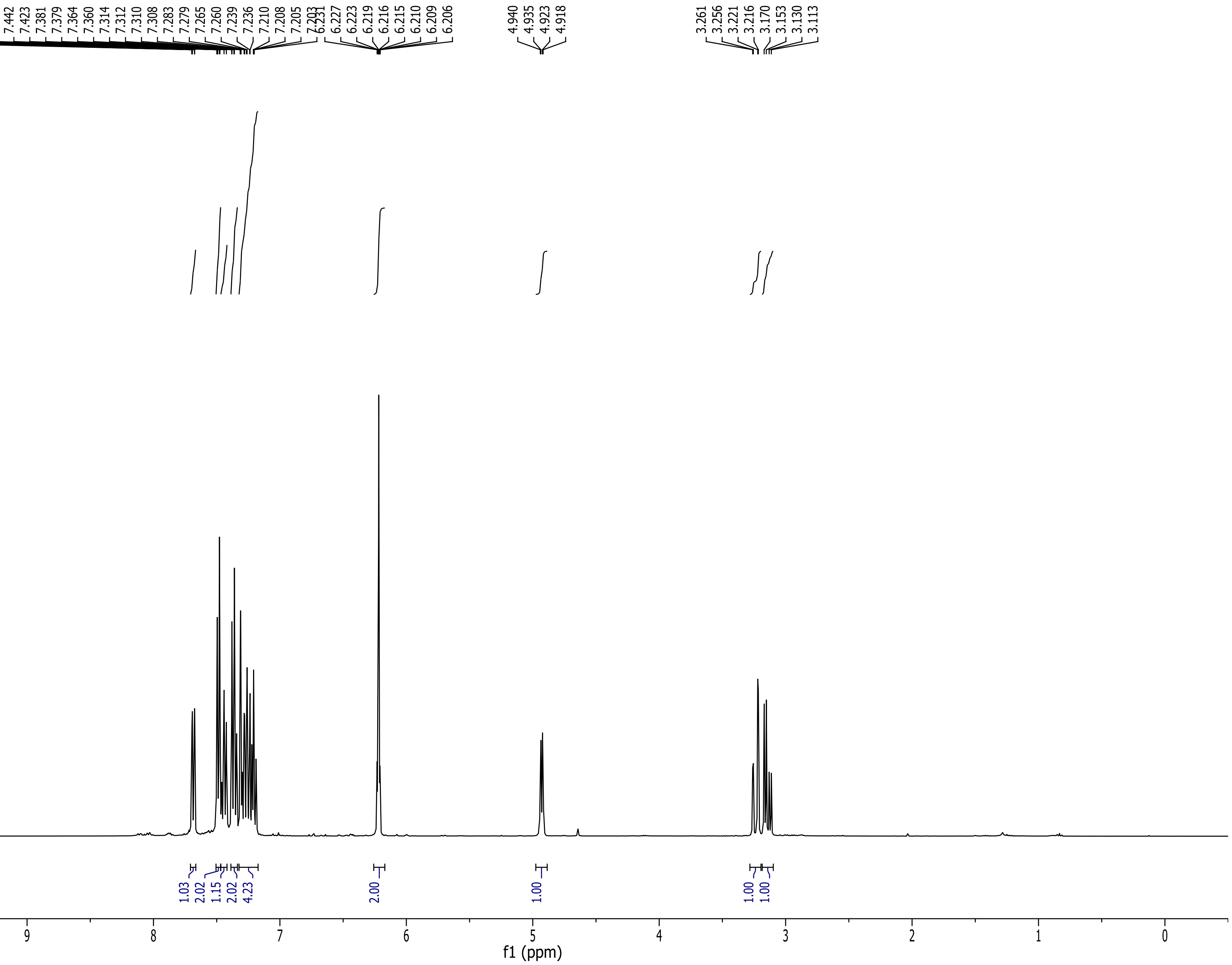

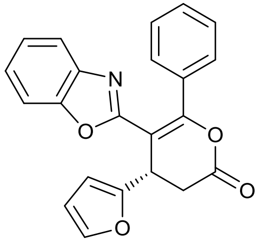

22B

^{13}C, 100 MHz, $CDCl_3$

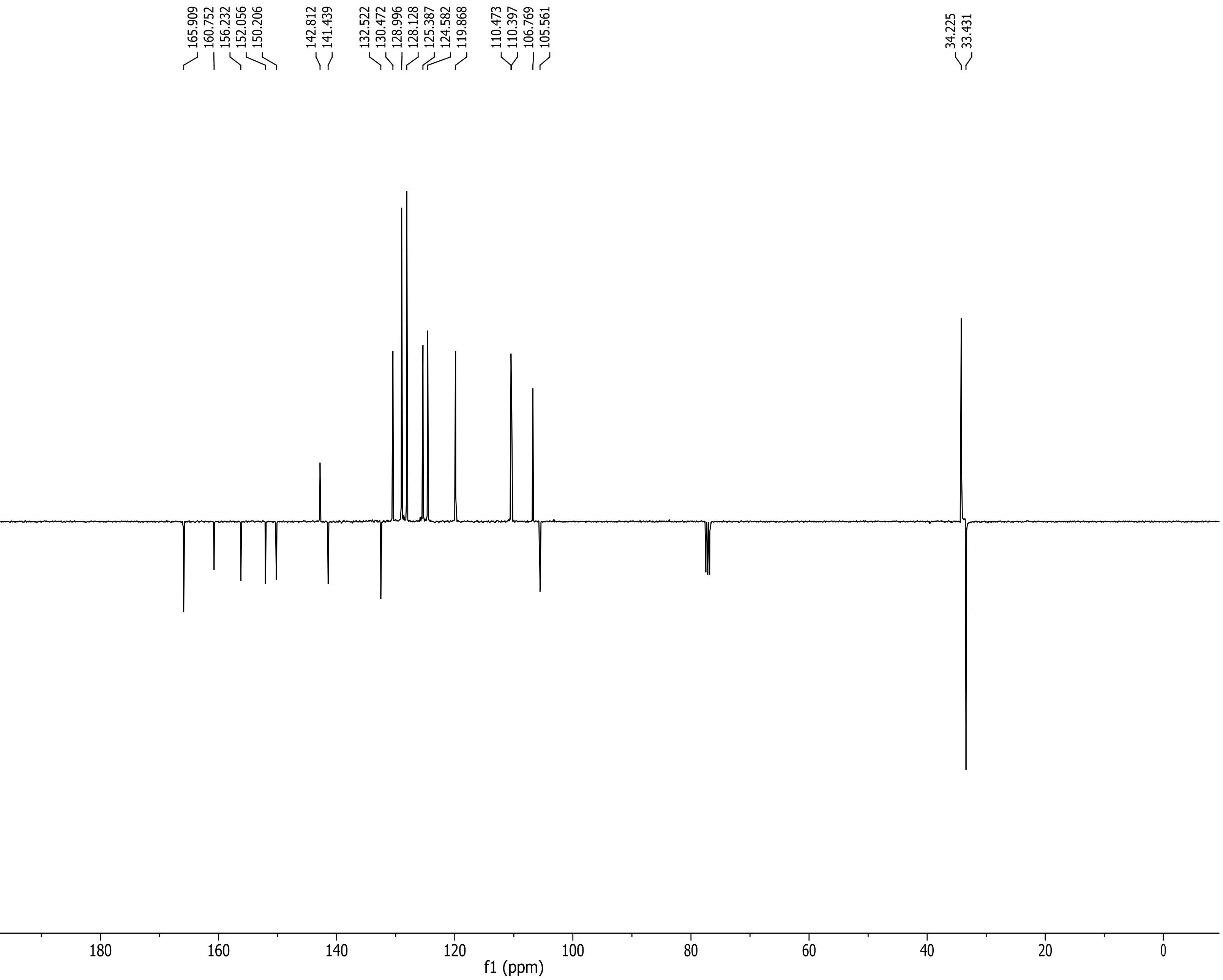

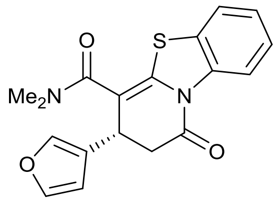

23A

^{1}H, 300 MHz, $CDCl_3$

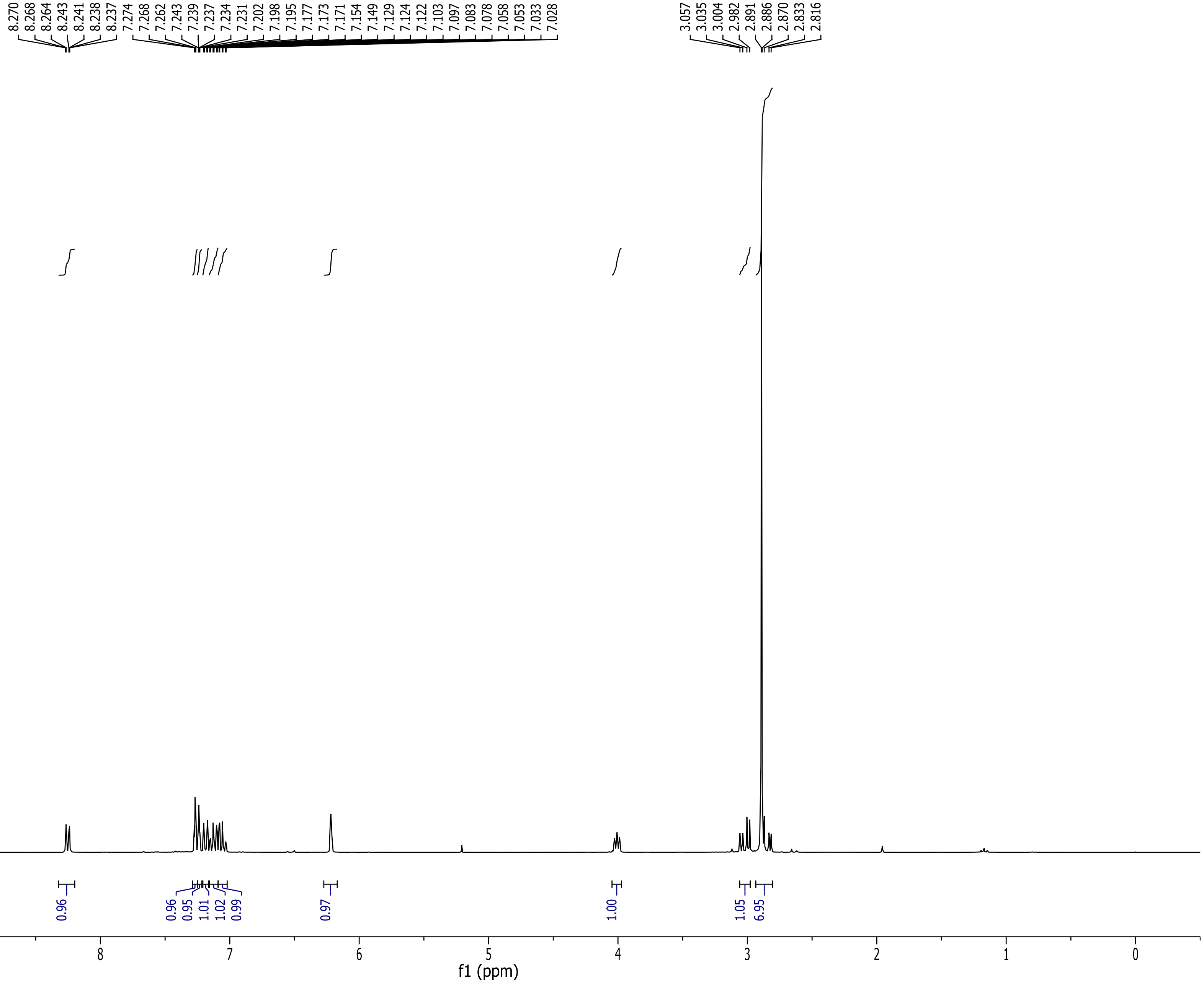

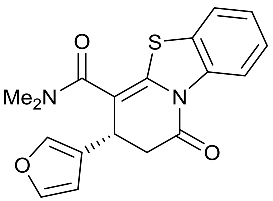

23A

^{13}C, 75 MHz, $CDCl_3$

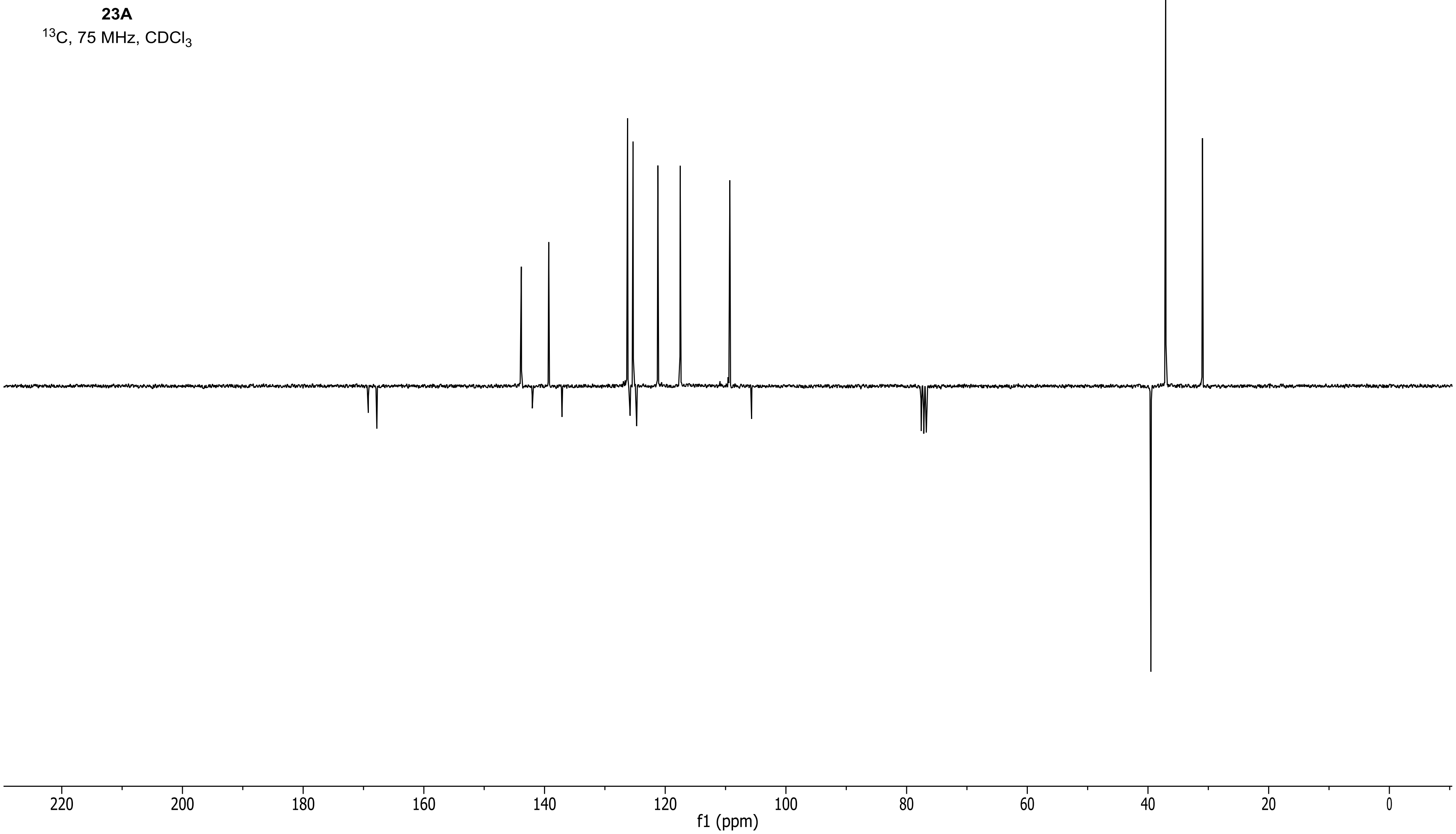

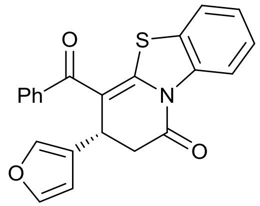

24A

^1H, 500 MHz, $CDCl_3$

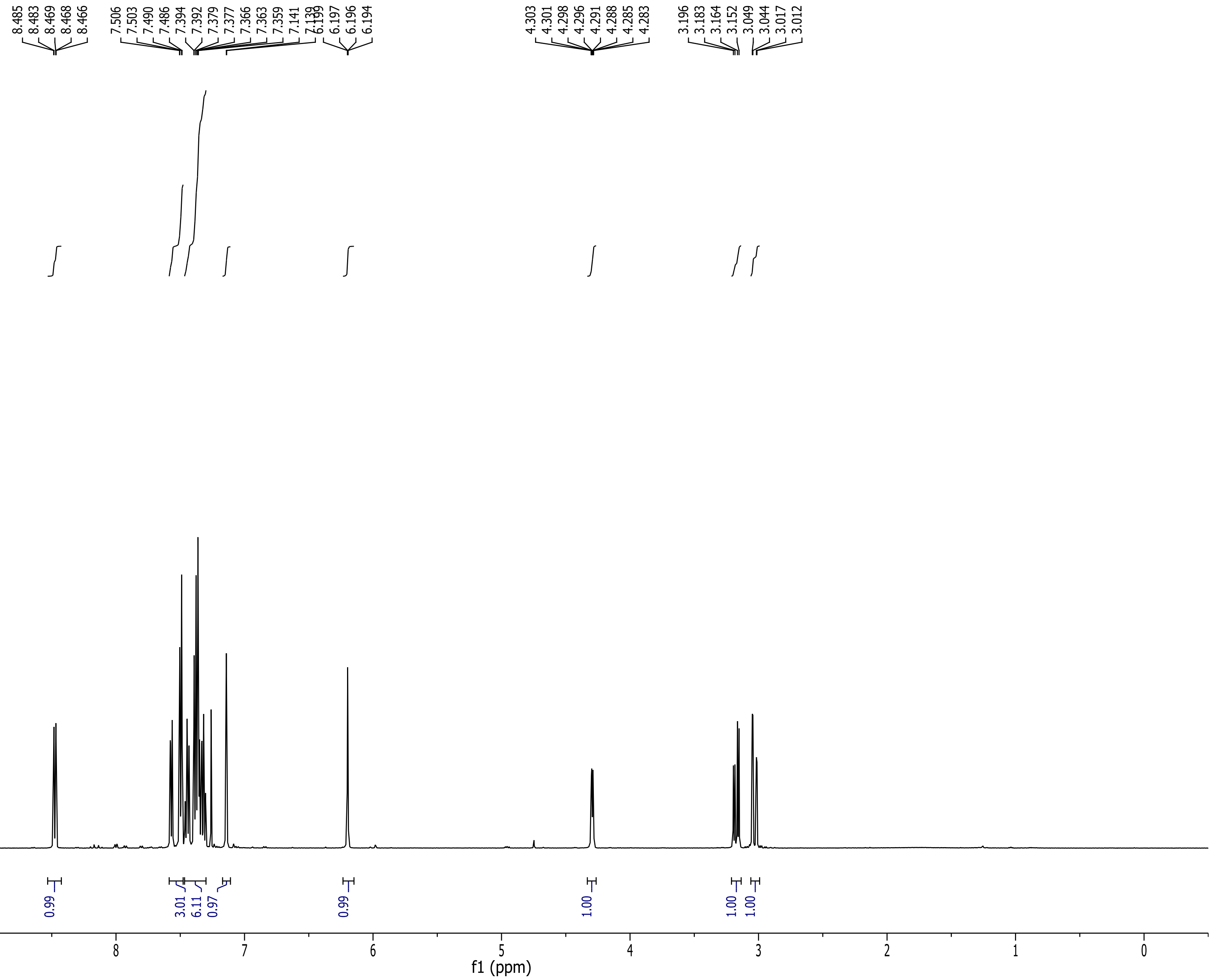

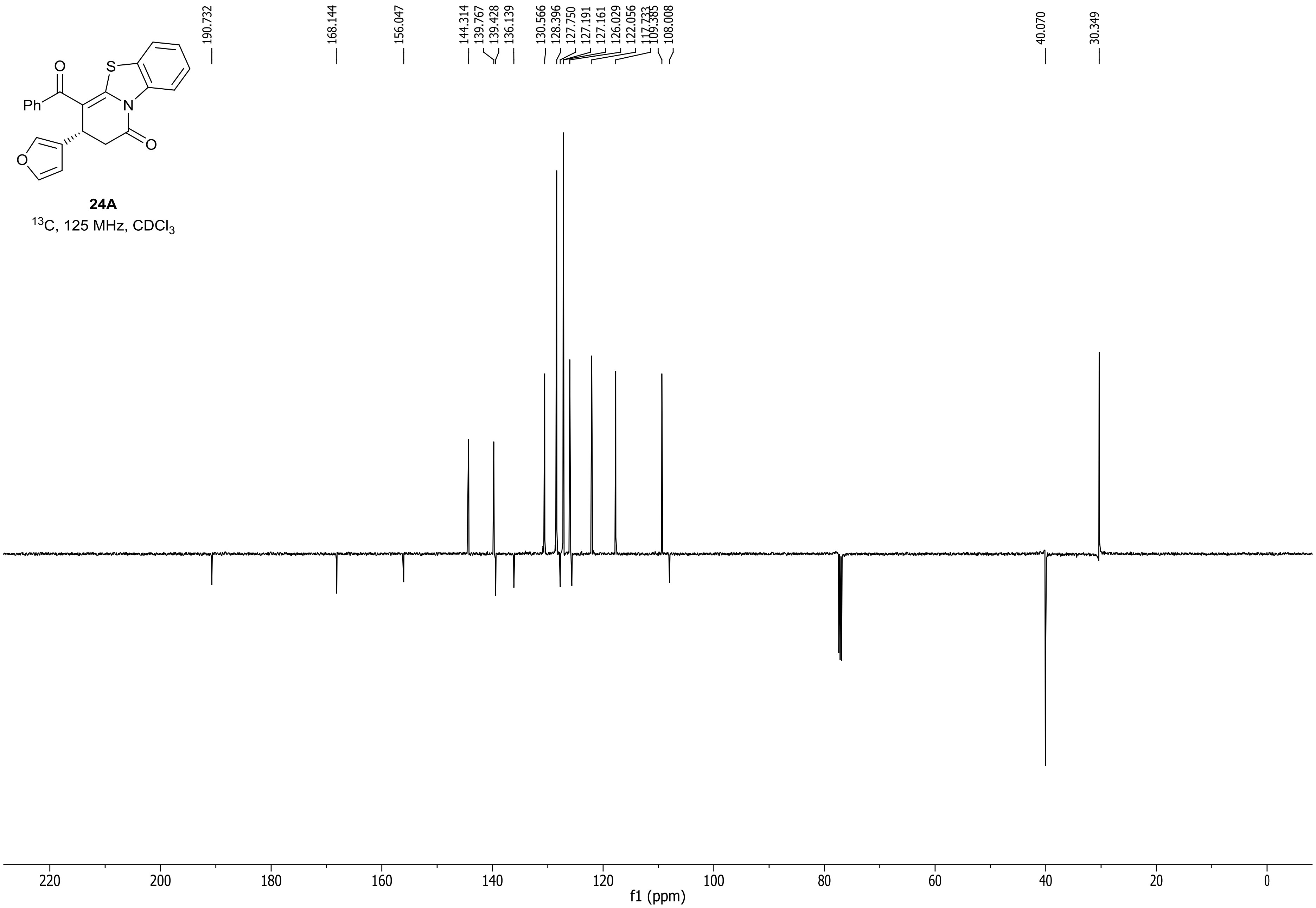
O
S
Ph
N
O
O
24A
^{13}C, 125 MHz, CDCl$_3$
190.732
168.144
156.047
144.314
139.767
139.428
136.139
130.566
128.396
127.750
127.191
127.161
126.029
122.056
117.733
109.385
108.008
40.070
30.349
220
200
180
160
140
120
100
80
60
40
20
0
f1 (ppm)

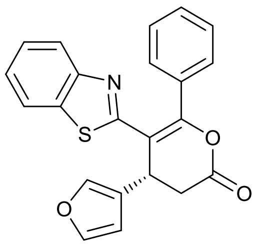

24B

[1]H, 500 MHz, $CDCl_3$

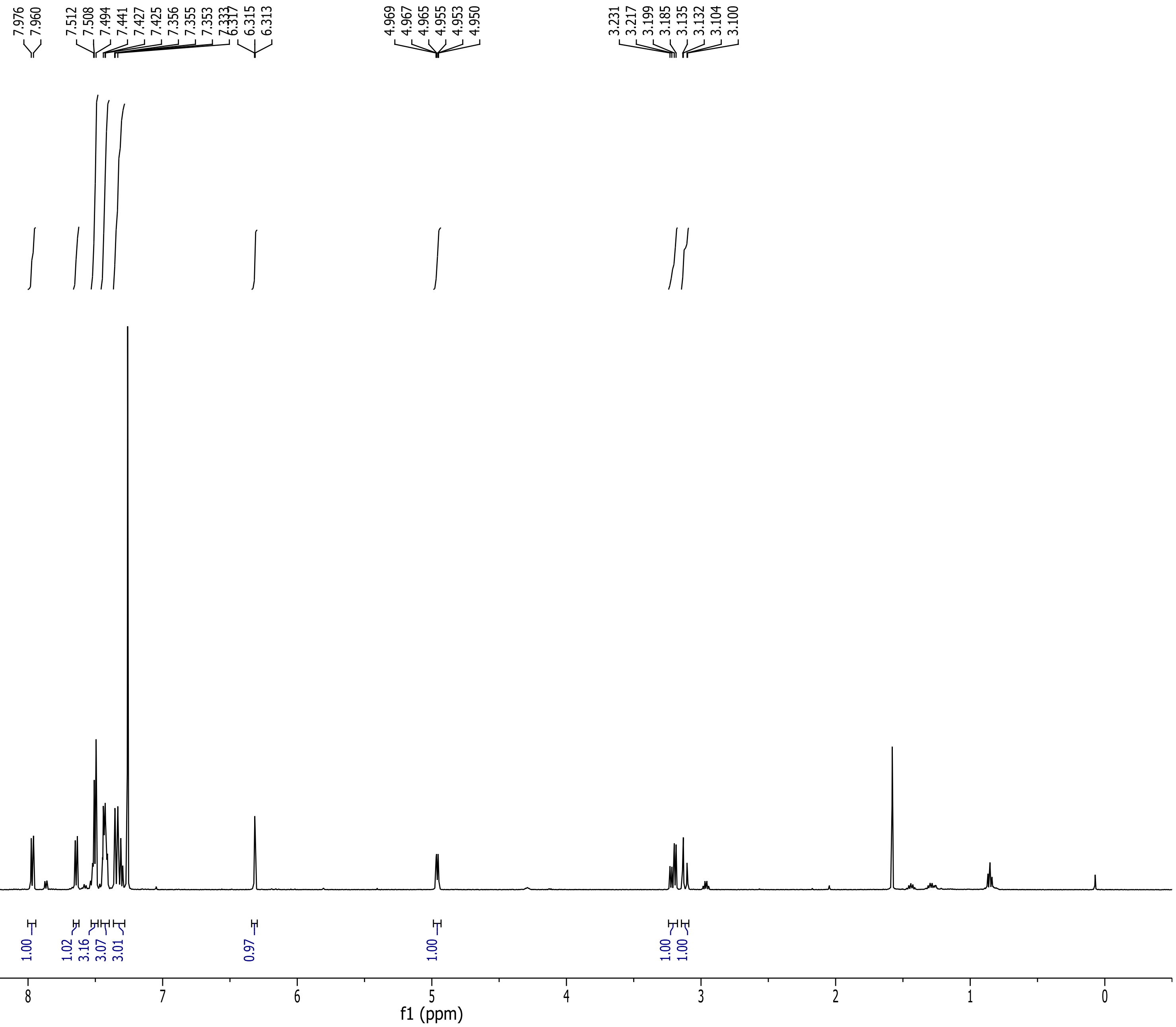

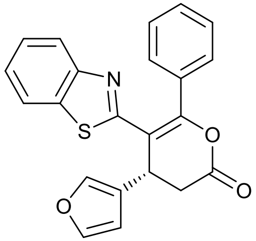

24B

^{13}C, 125 MHz, $CDCl_3$

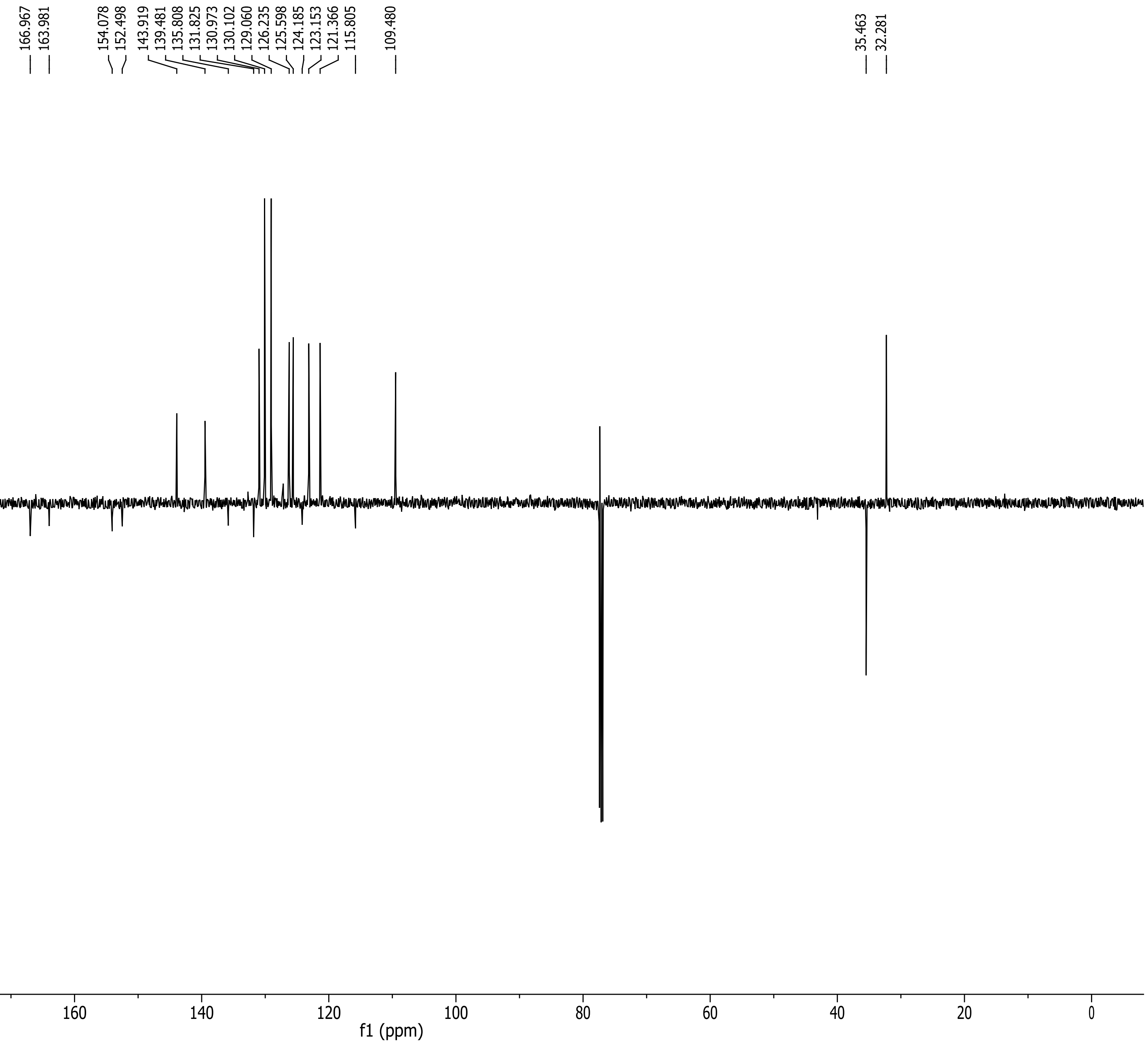

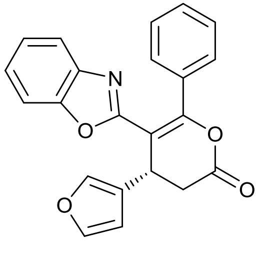

25B

^{1}H, 300 MHz, $CDCl_3$

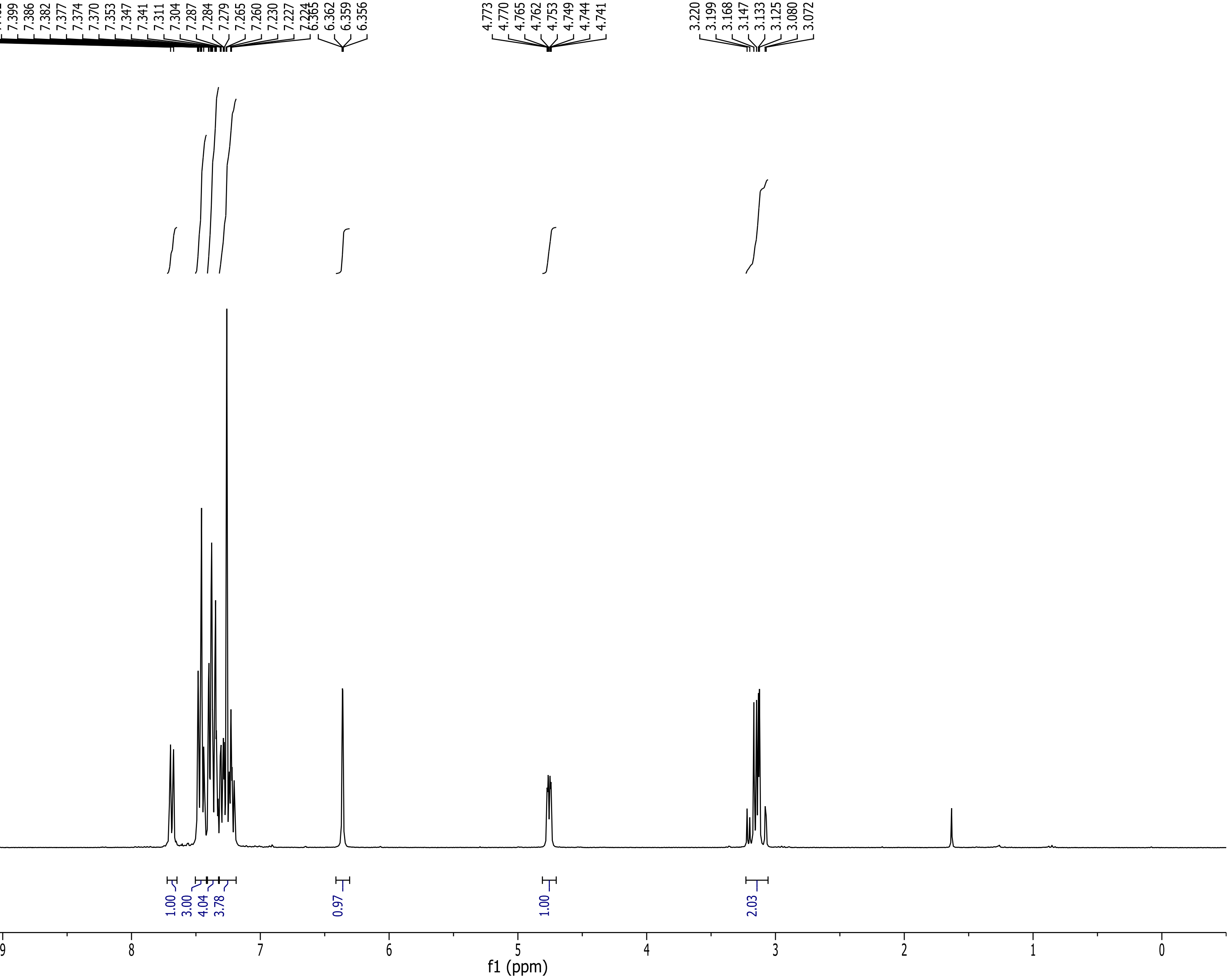

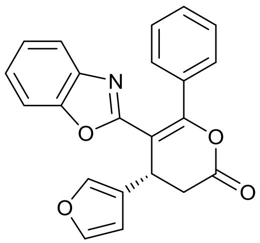
25B
^{13}C, 125 MHz, $CDCl_3$

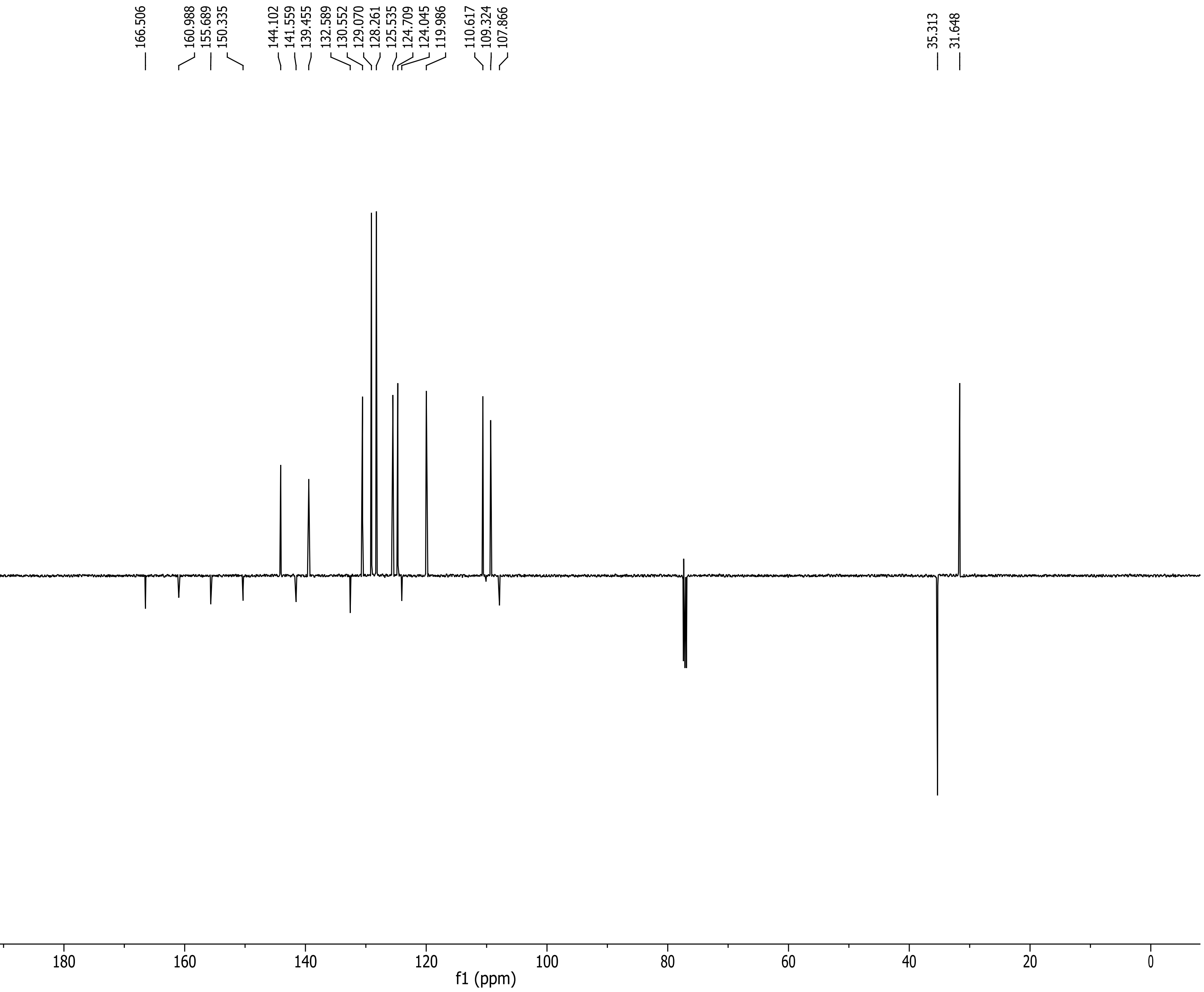

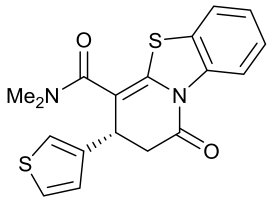

26A

^1H, 300 MHz, $CDCl_3$

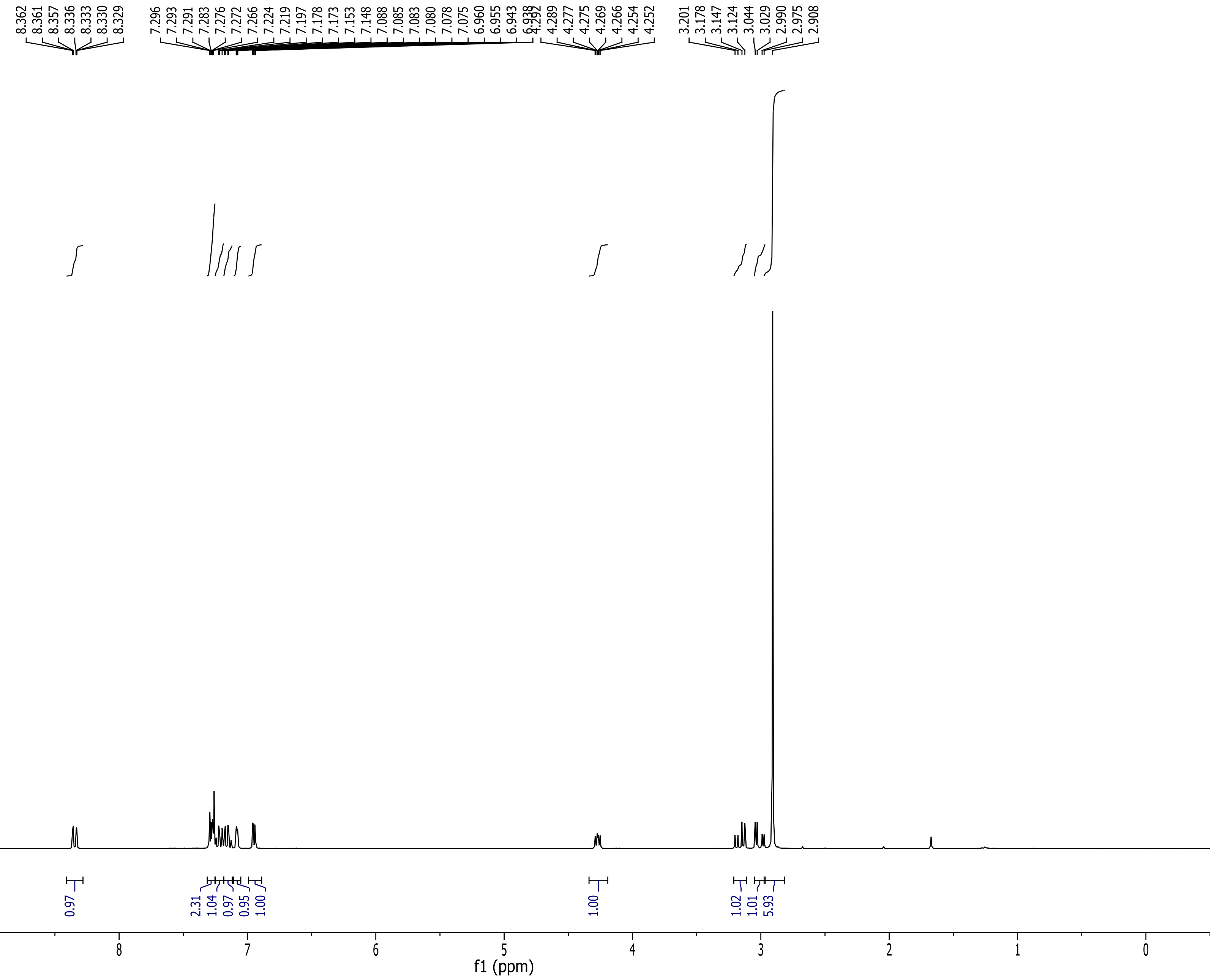

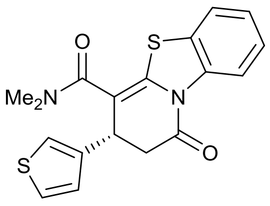

26A

^{13}C, 125 MHz, $CDCl_3$

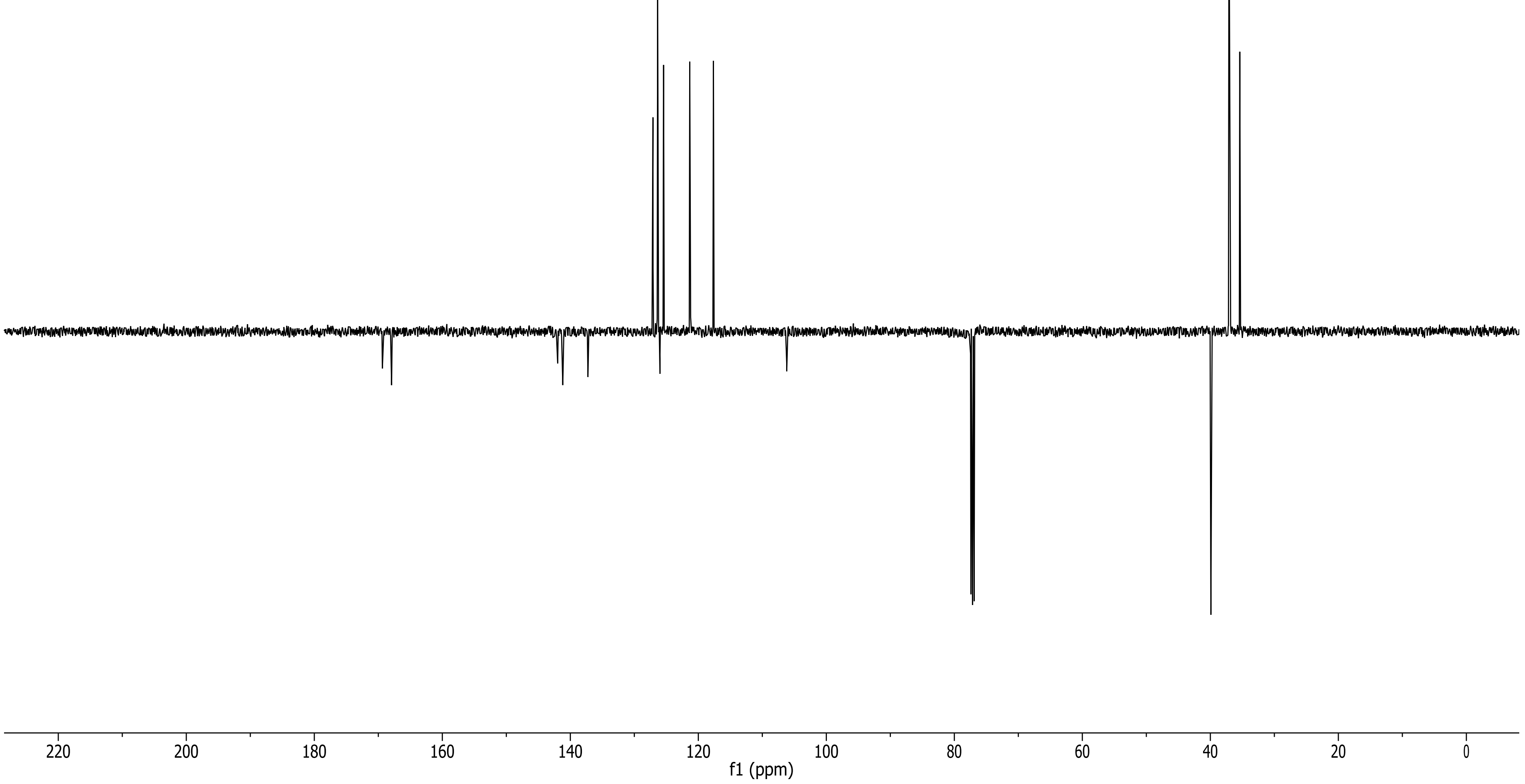

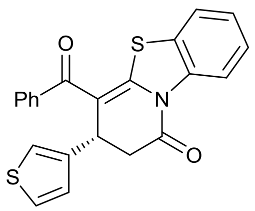

27A
^1H, 500 MHz, $CDCl_3$

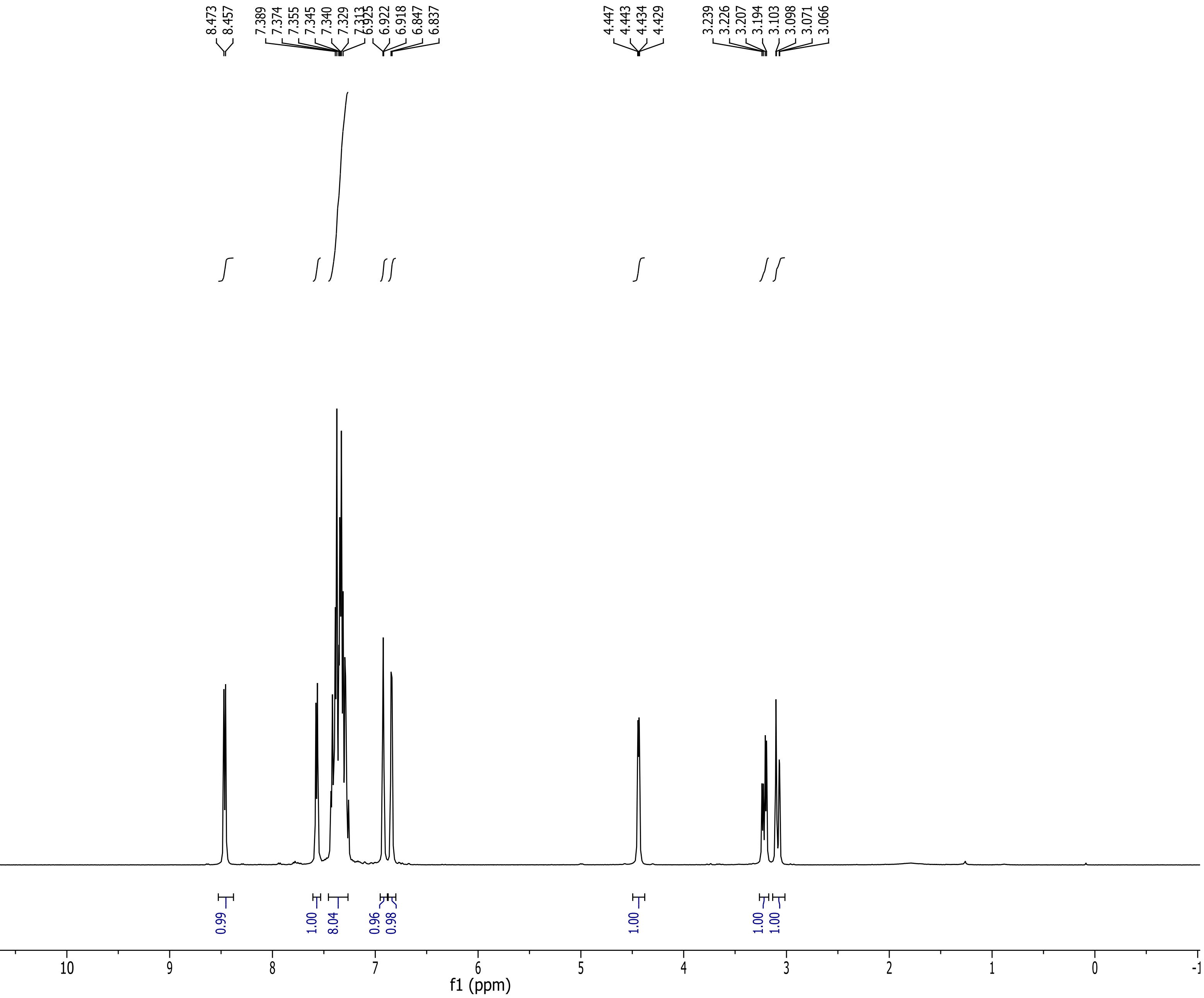

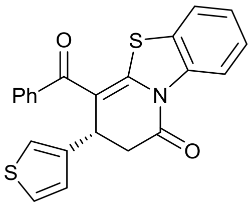

27A
^{13}C, 125 MHz, $CDCl_3$

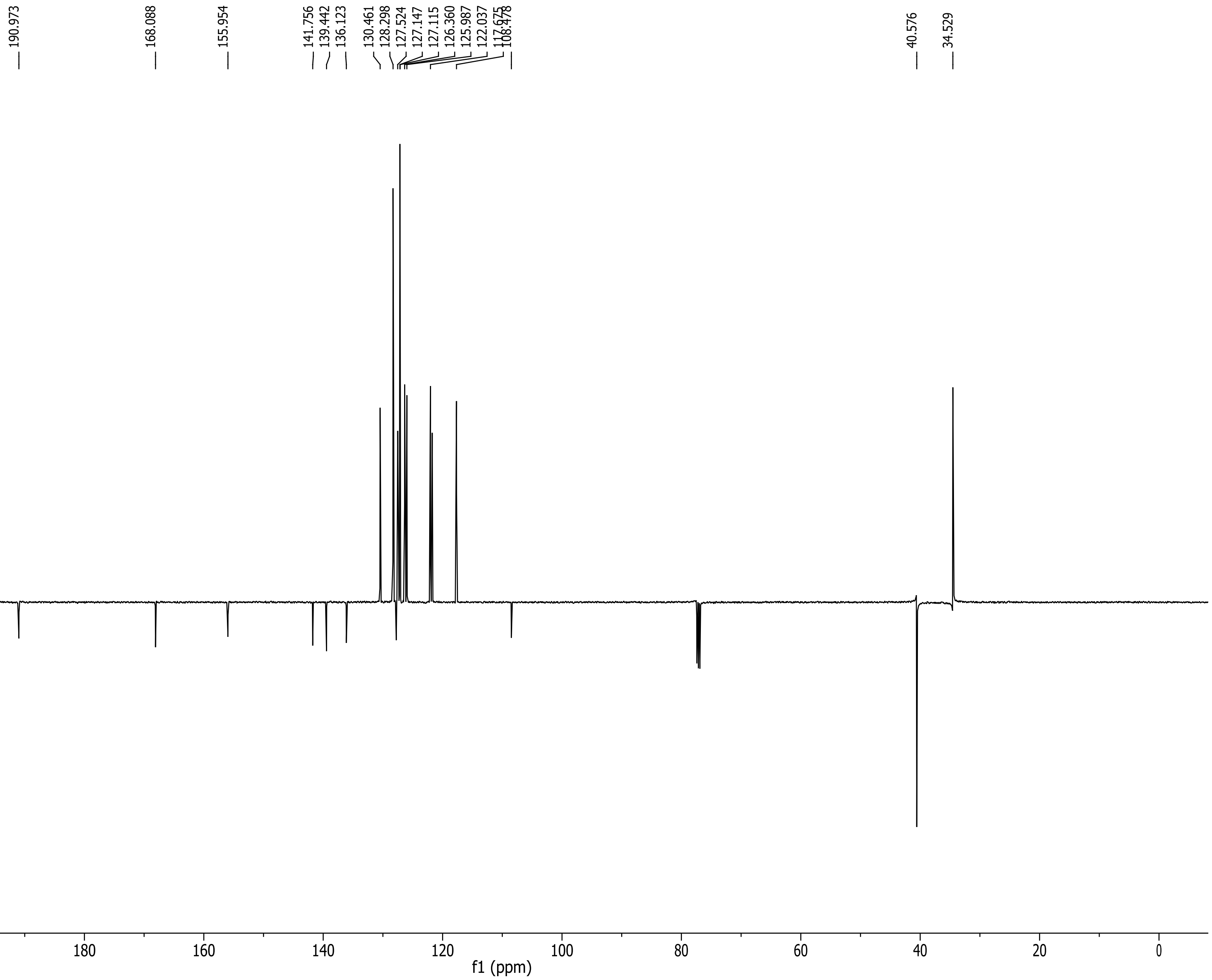

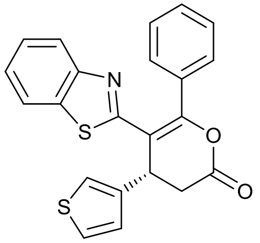

27B

^{1}H, 500 MHz, $CDCl_3$

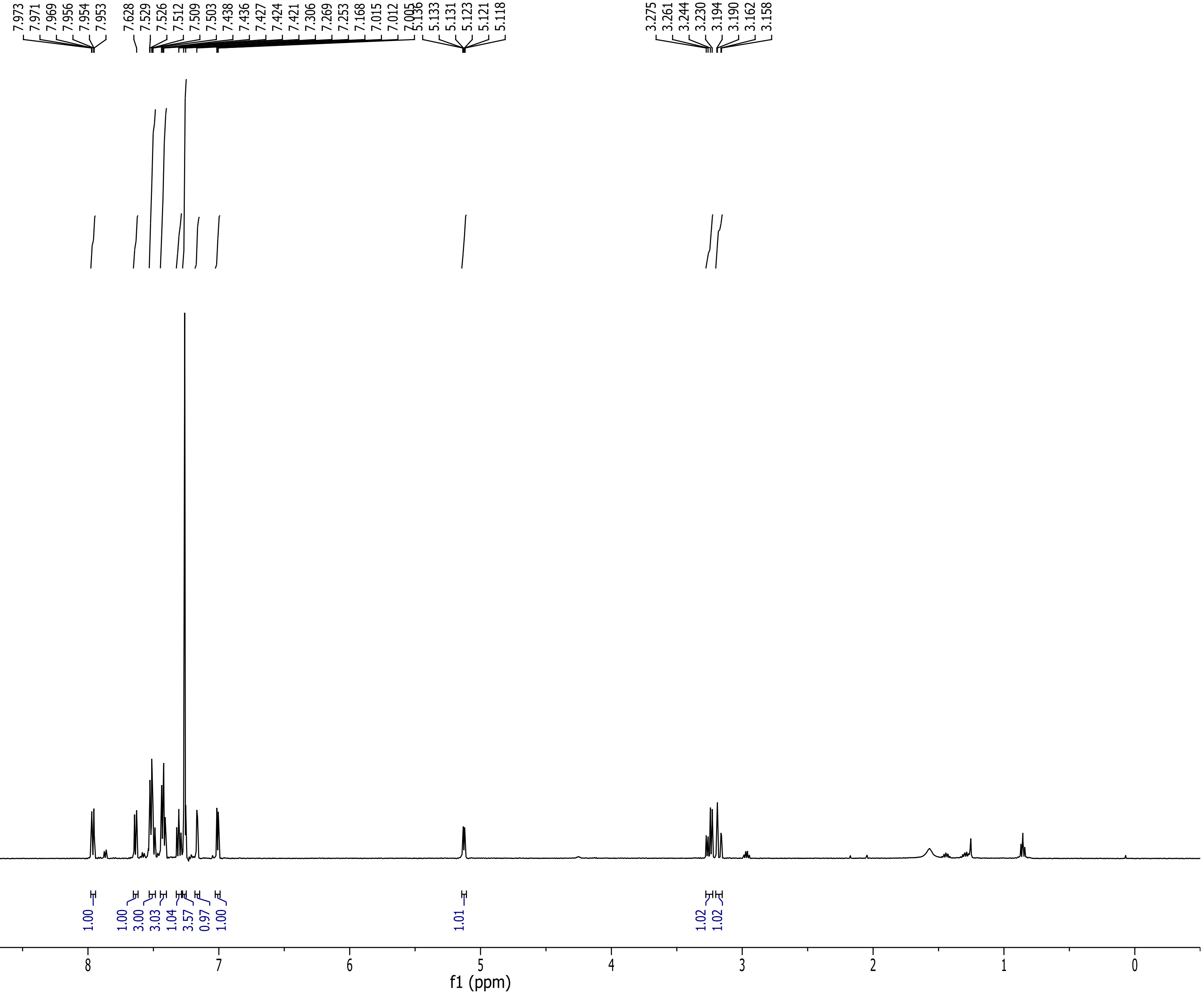

,

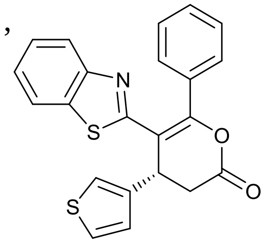

27B

^{13}C, 125 MHz, $CDCl_3$

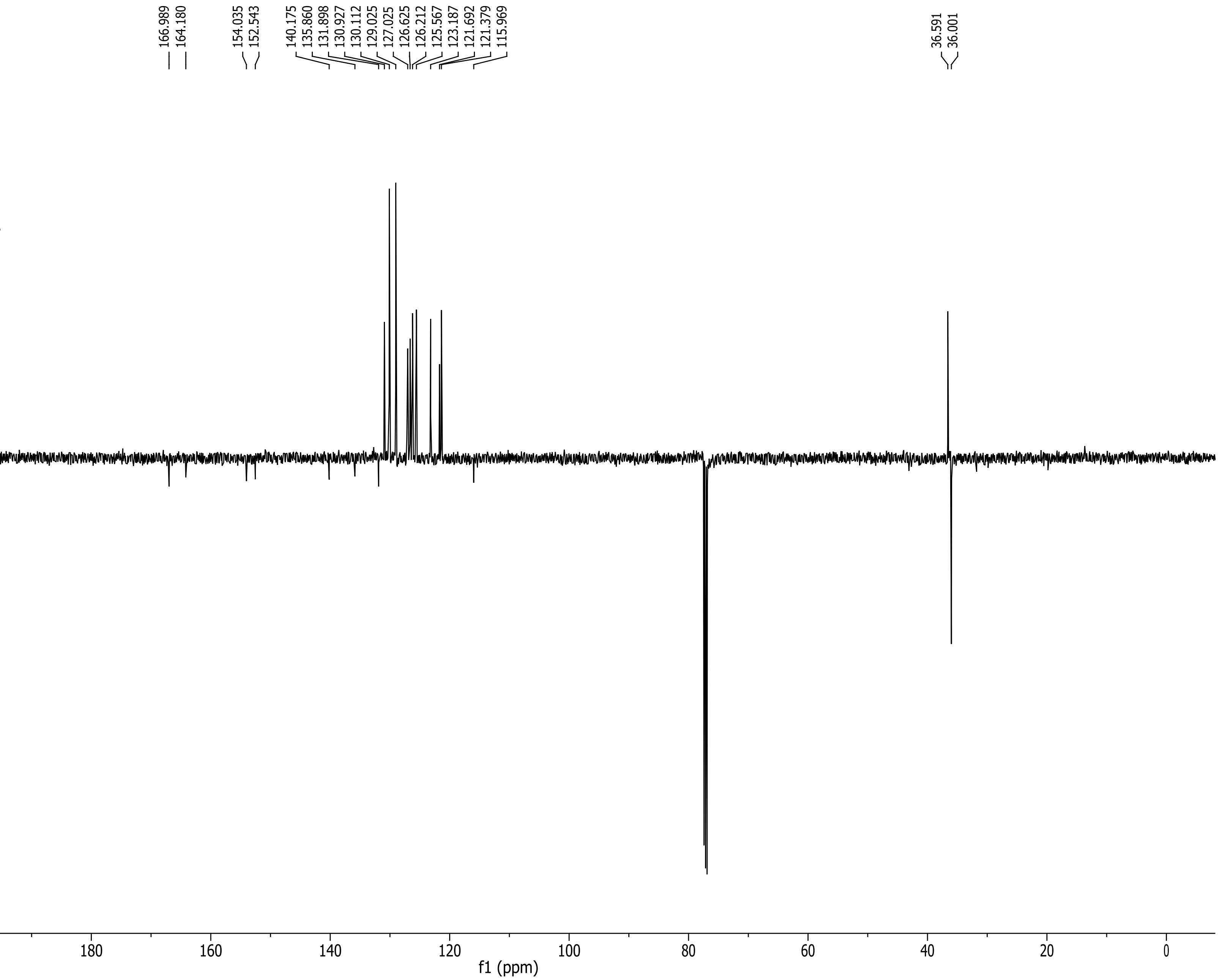

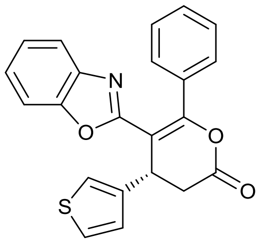

28B

^{1}H, 300 MHz, $CDCl_3$

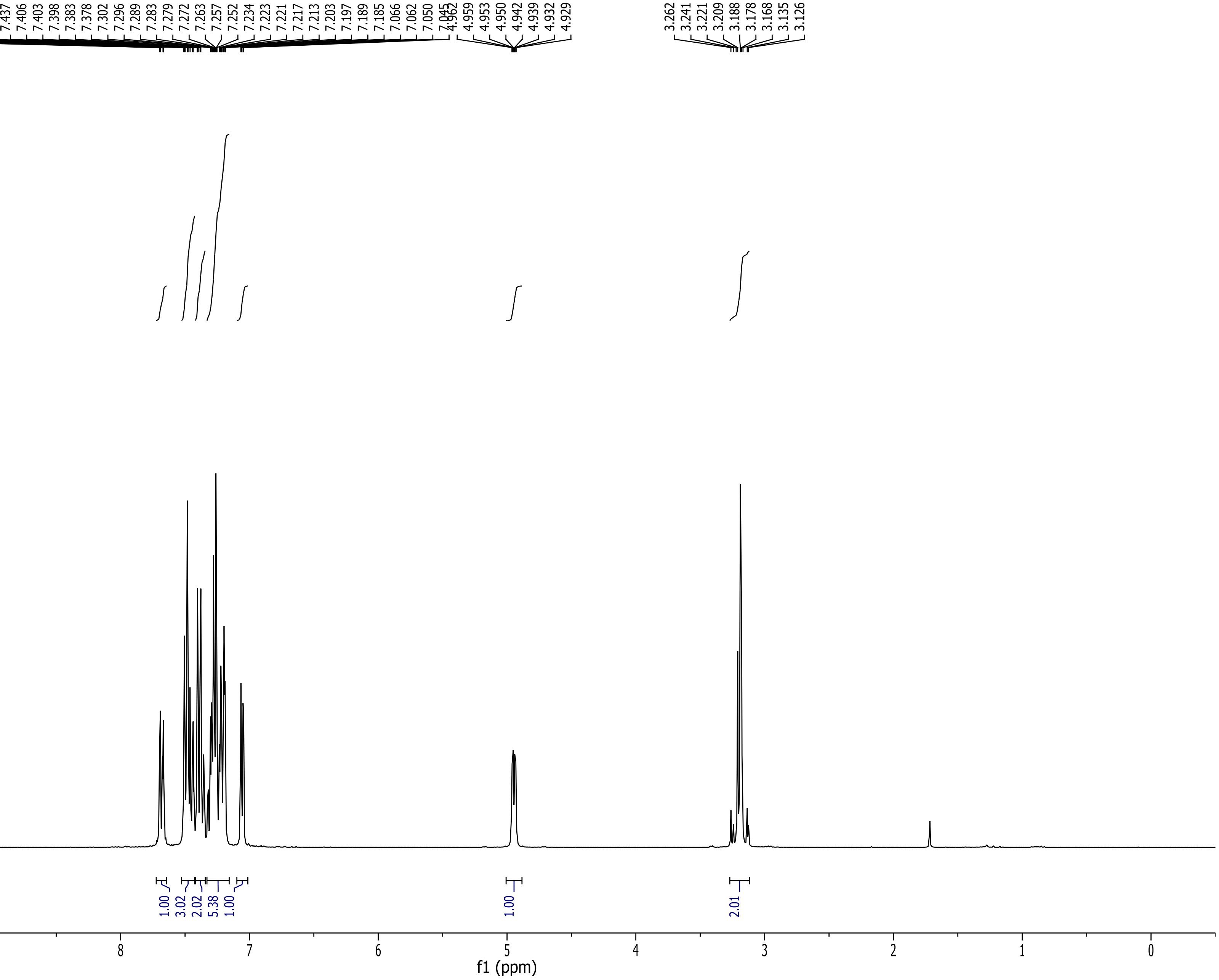

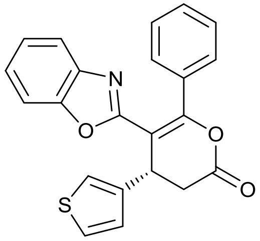

28B

^{13}C, 125 MHz, $CDCl_3$

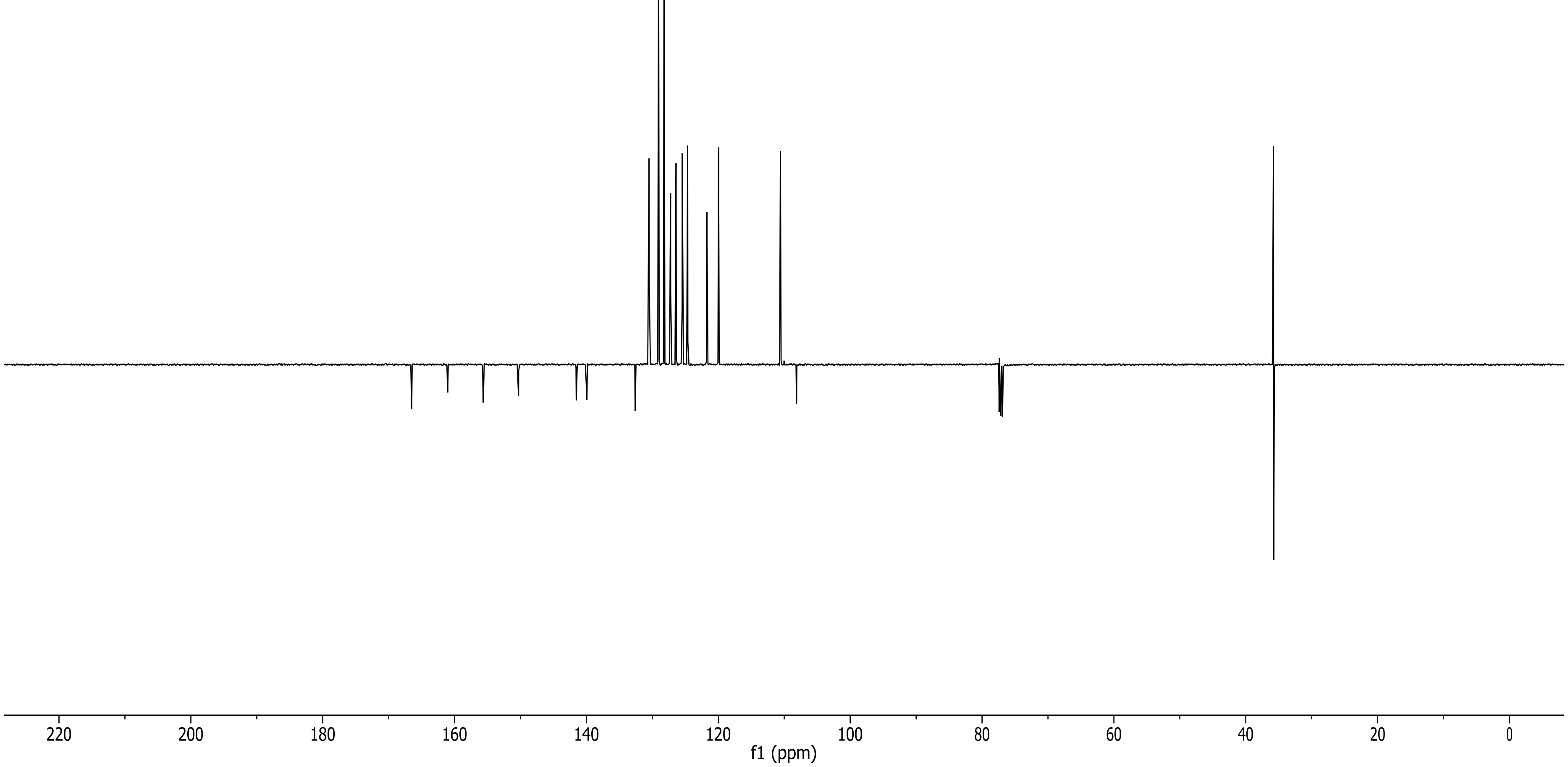

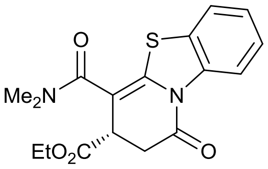

29A

^{1}H, 500 MHz, $CDCl_3$

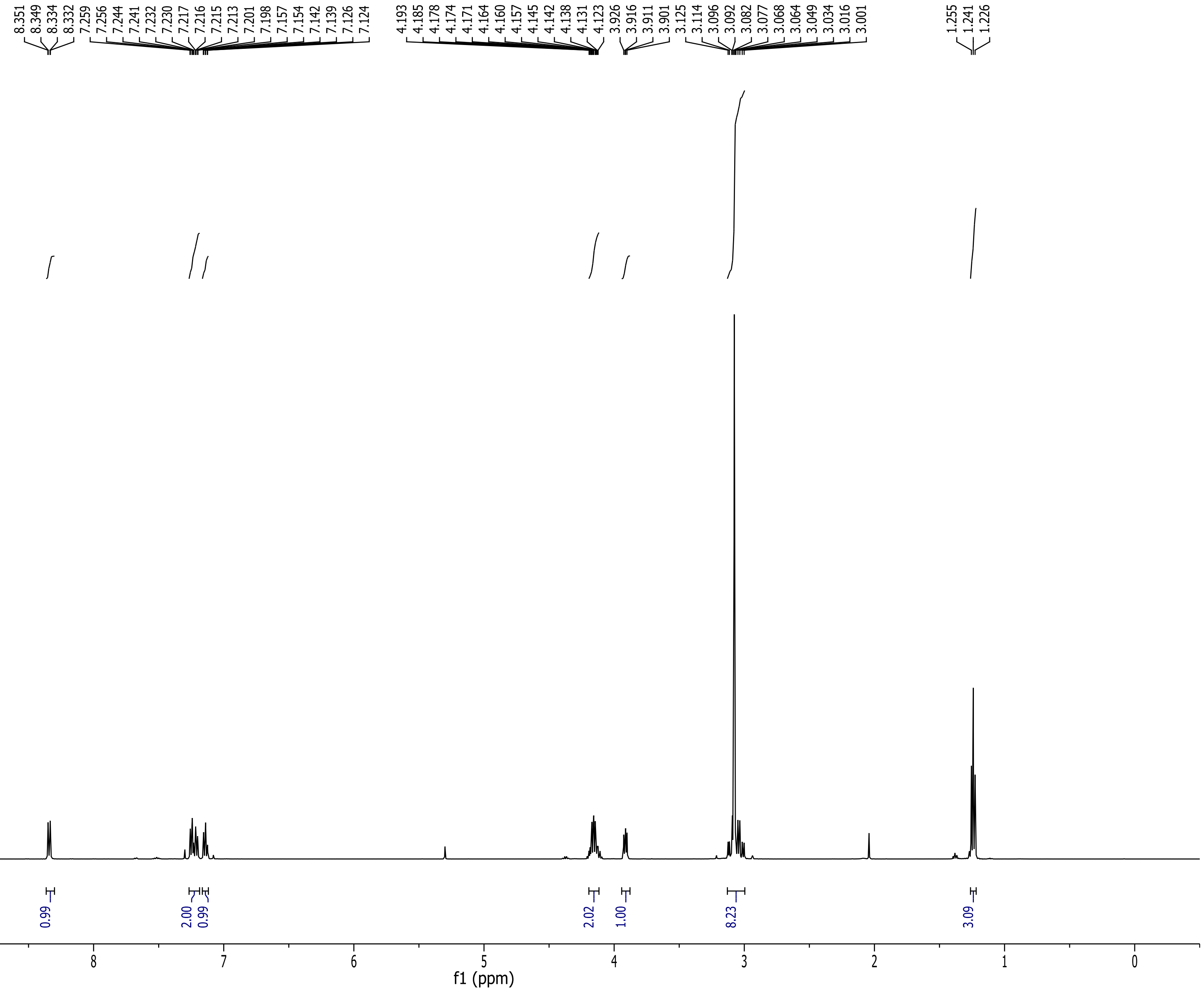

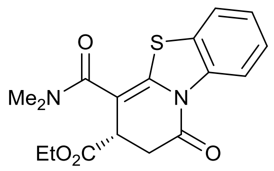

29A
^{13}C, 125 MHz, $CDCl_3$

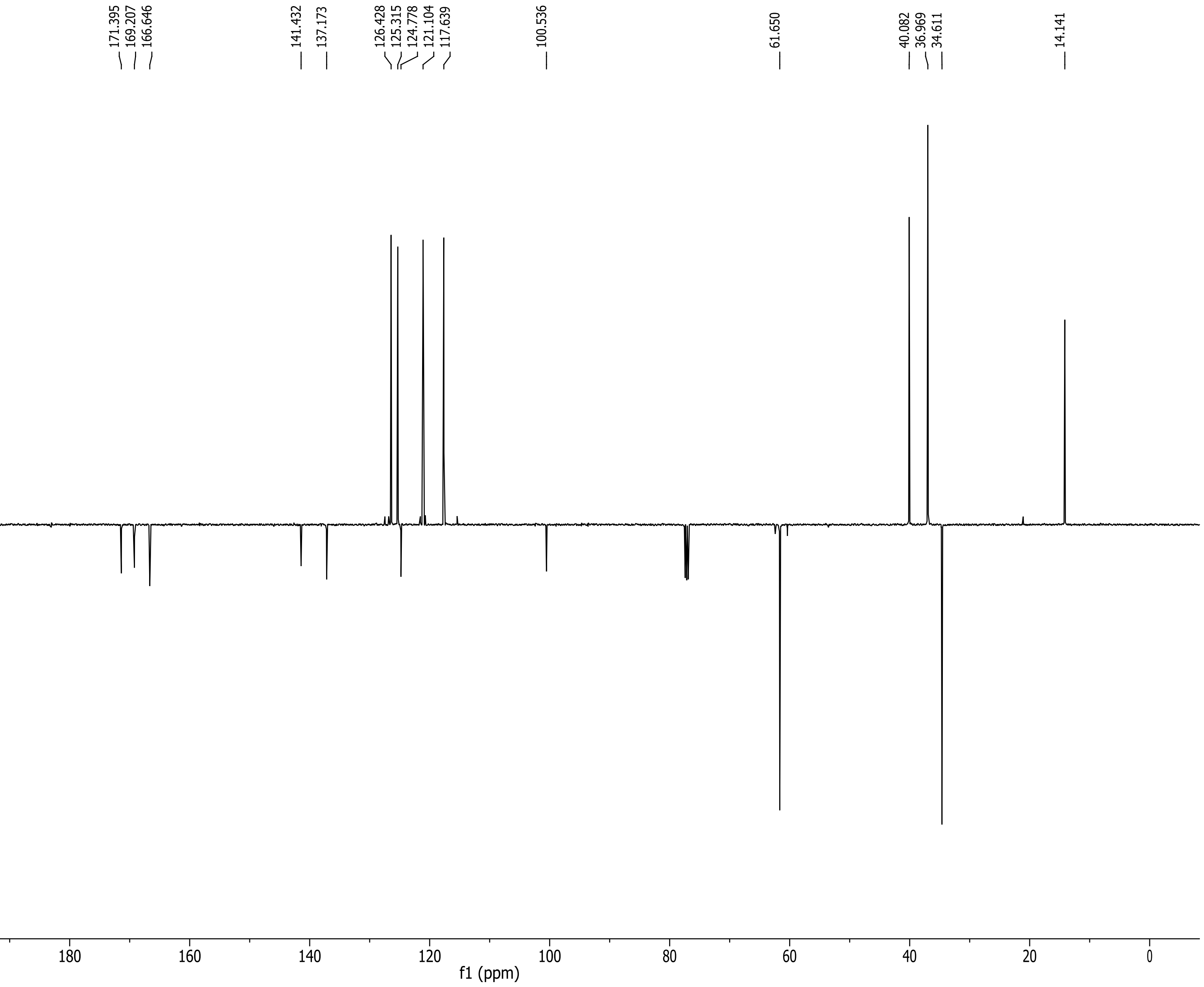

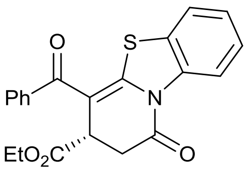

30A

^{1}H, 300 MHz, $CDCl_3$

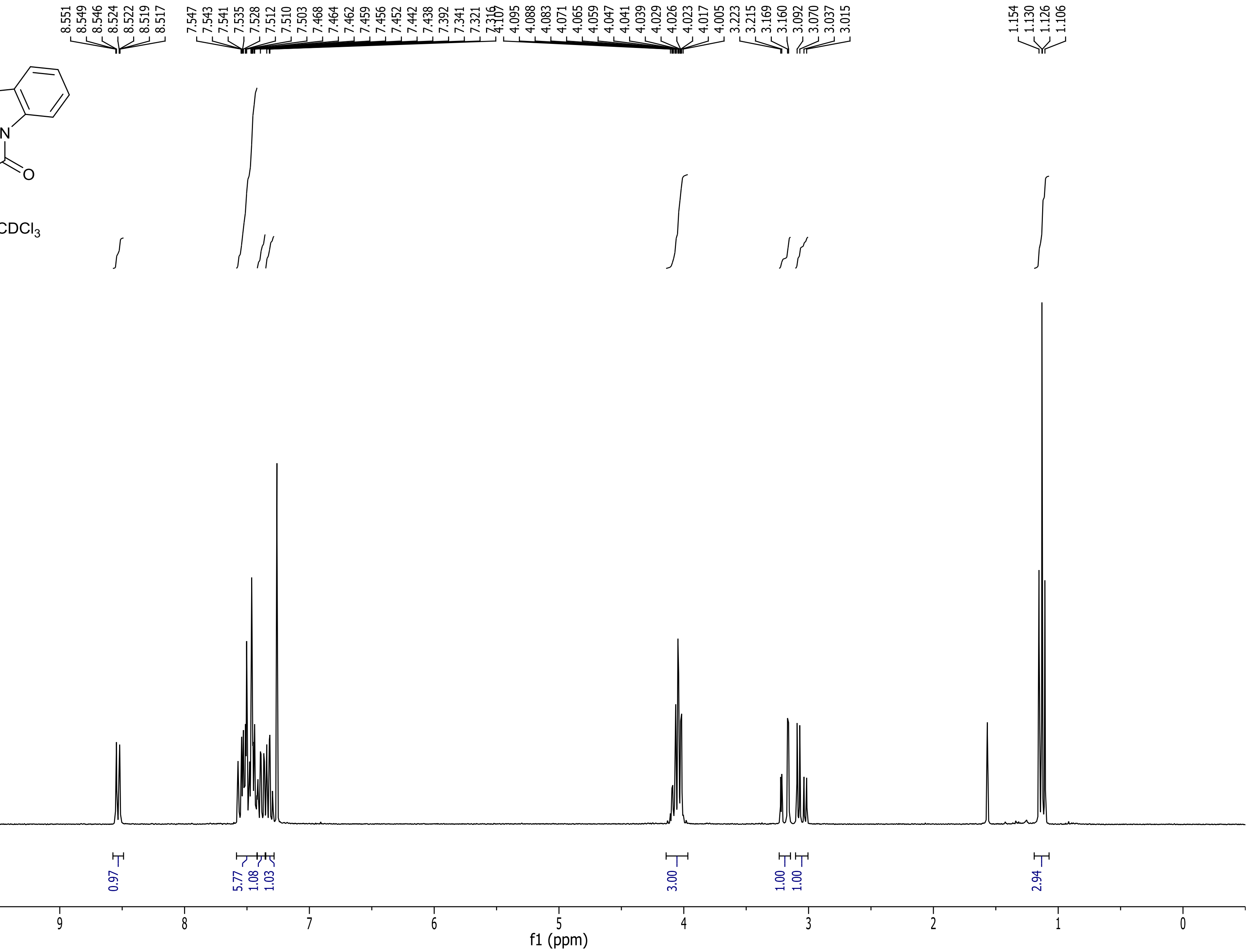

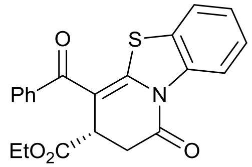

30A

^{13}C, 75 MHz, CDCl$_3$

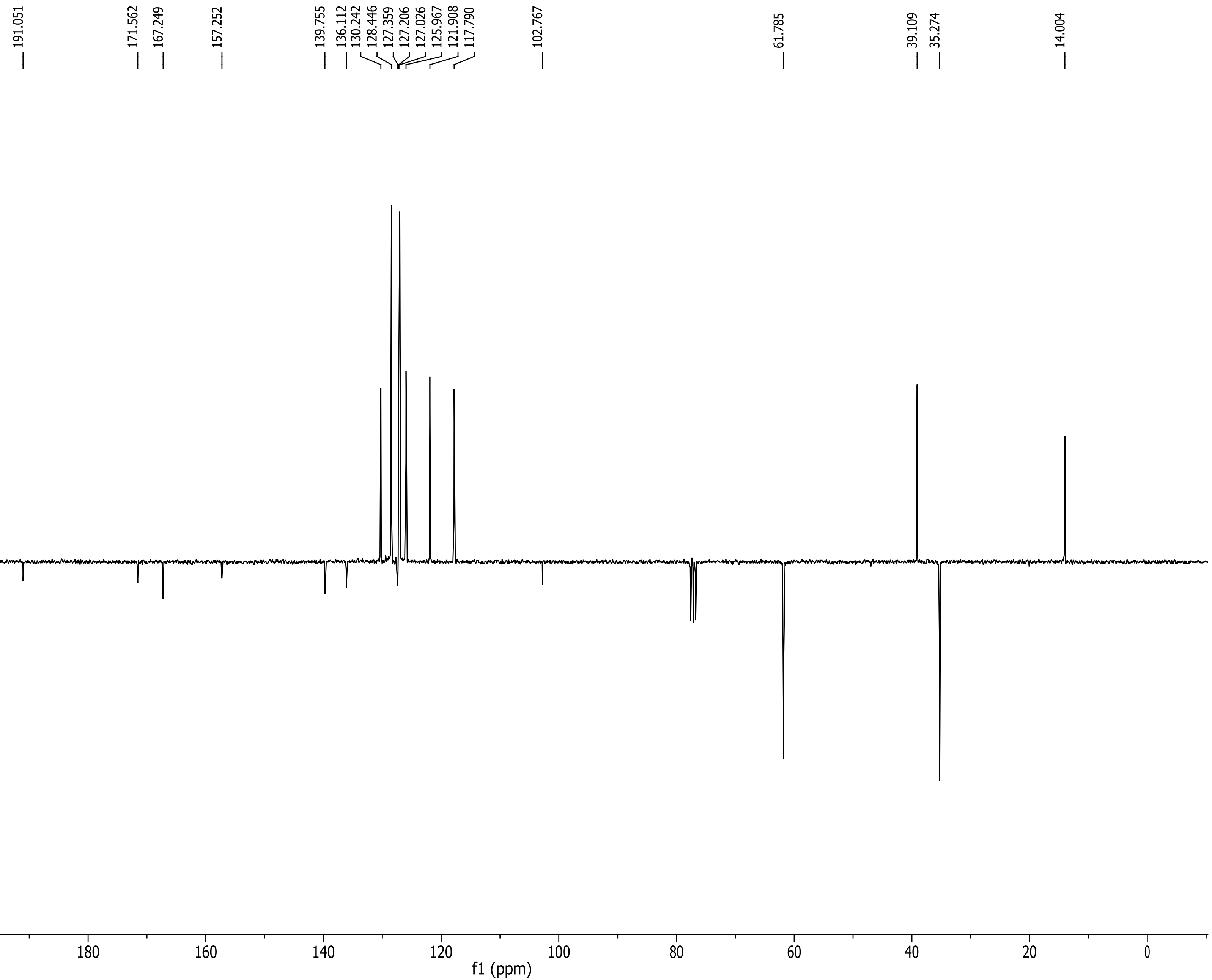

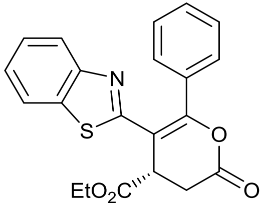

30B
^{1}H, 400 MHz, $CDCl_3$

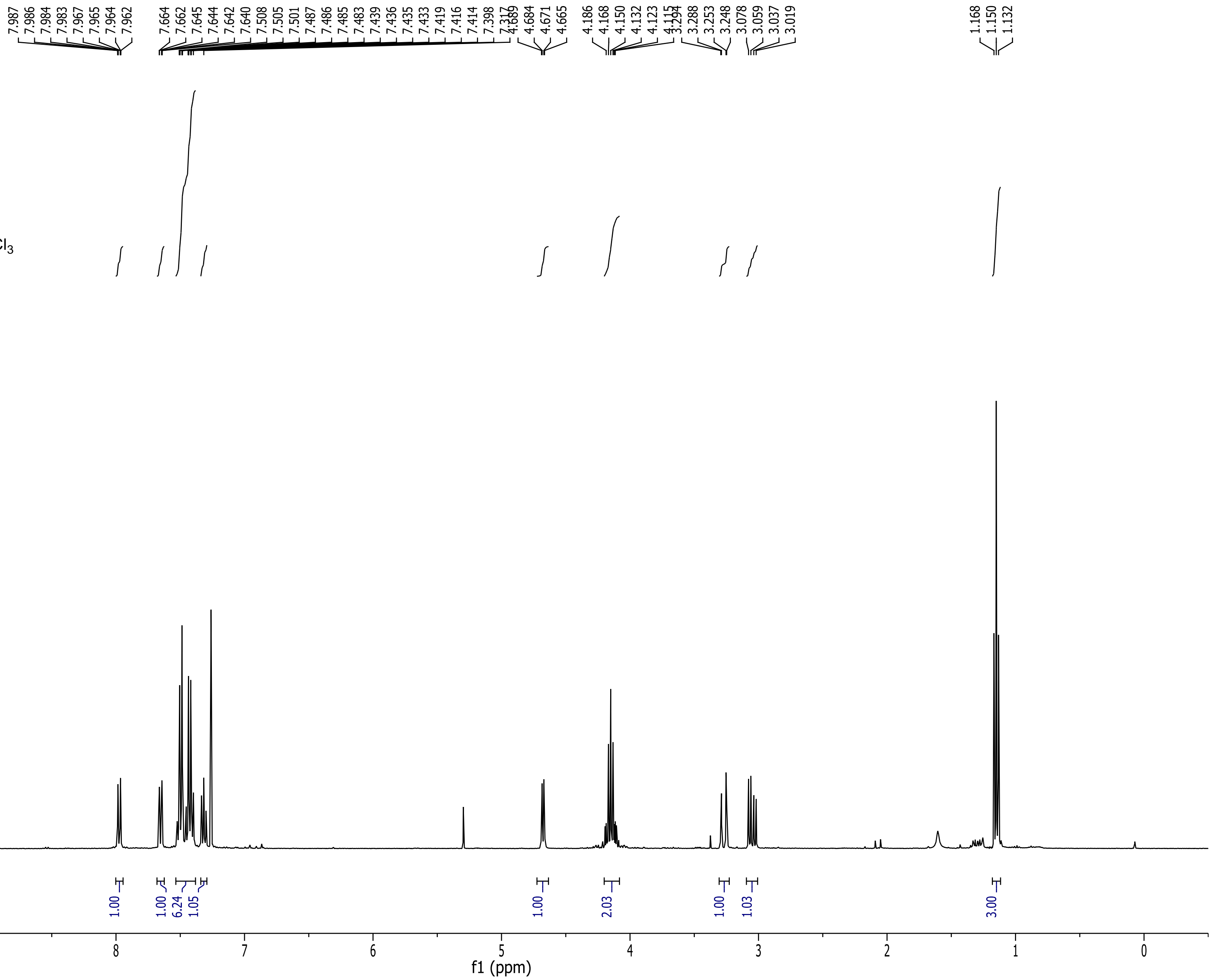

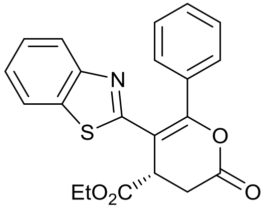

30B
^{13}C, 125 MHz, $CDCl_3$

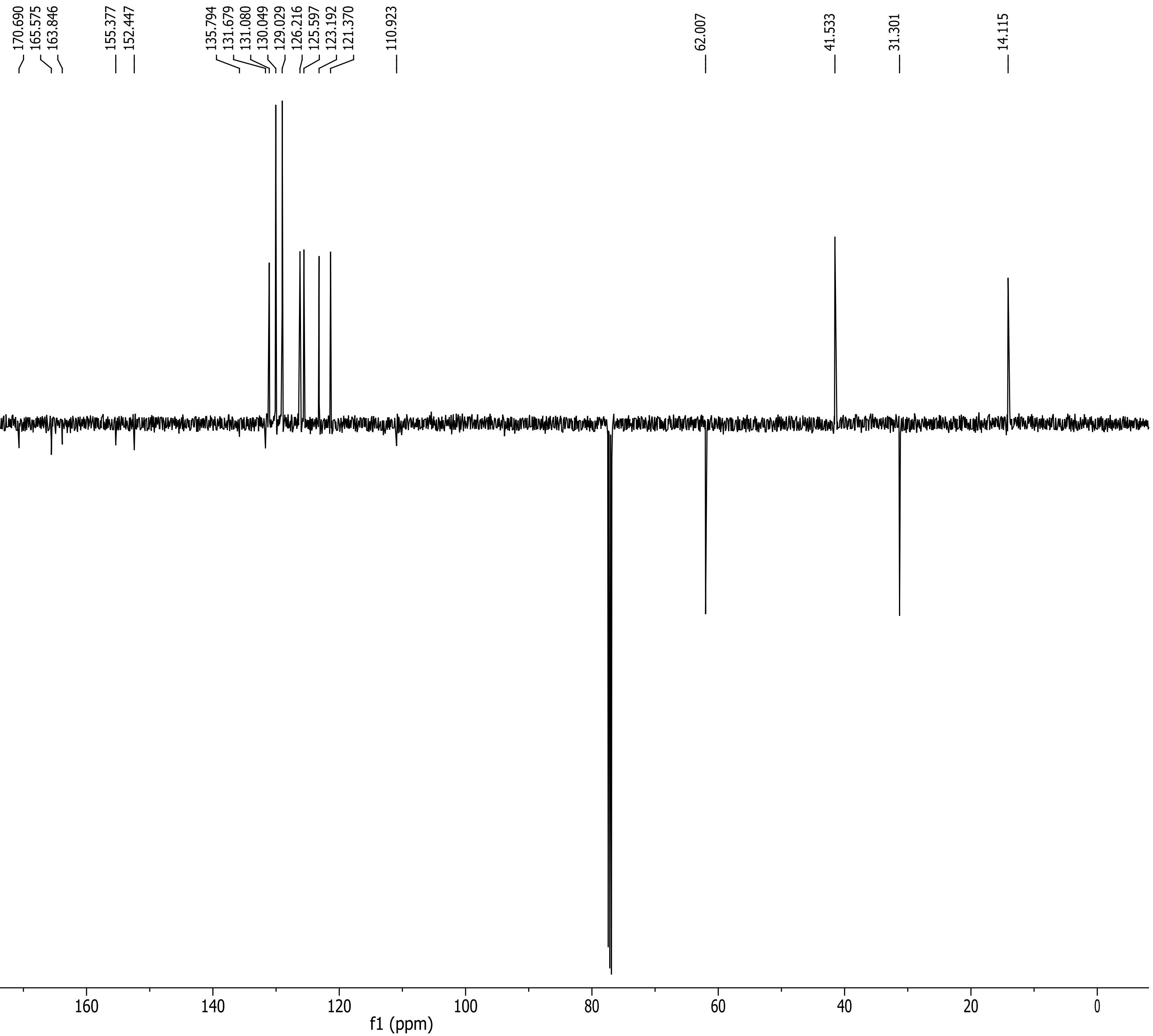

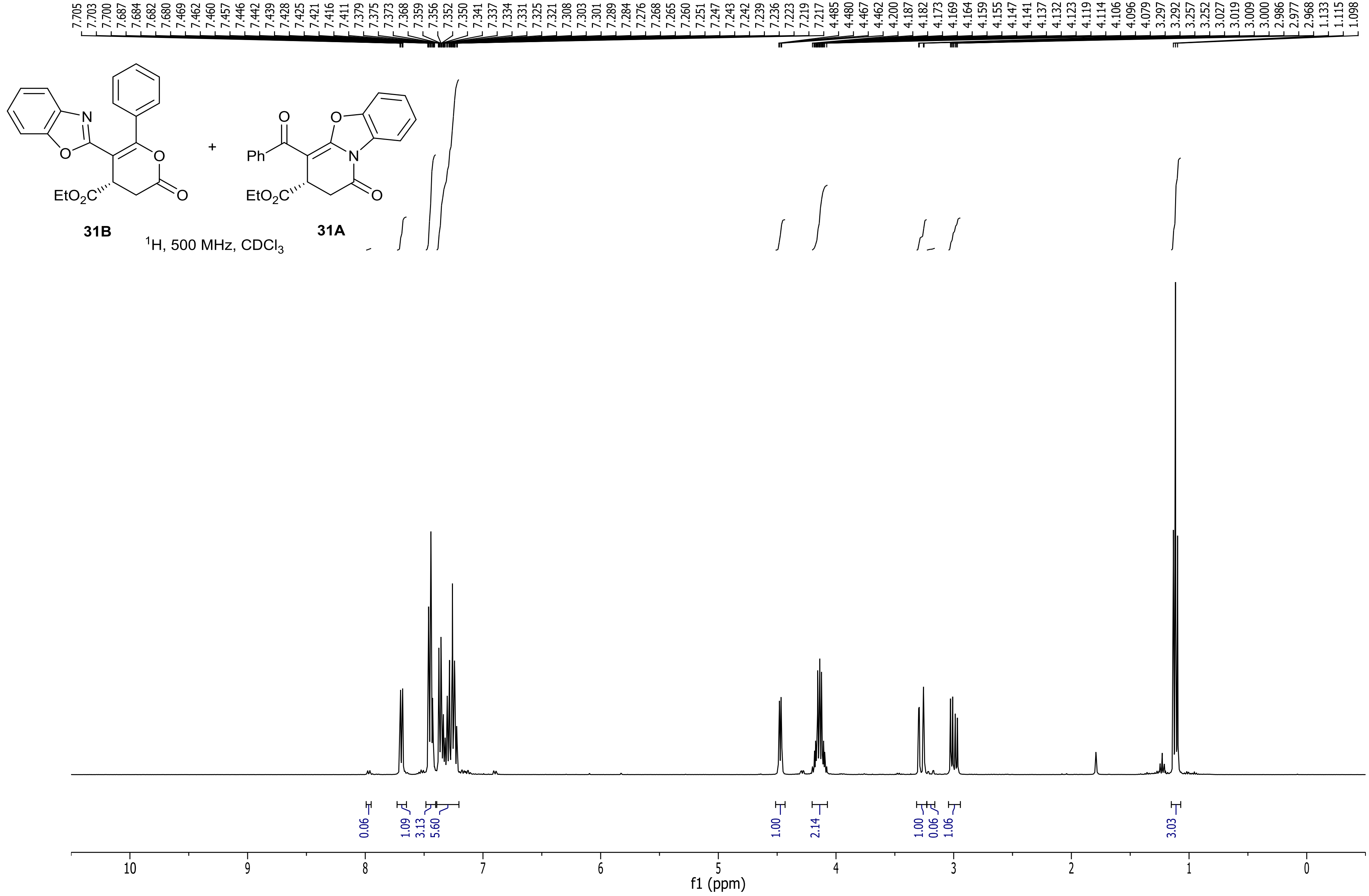
7.705 7.703 7.700 7.687 7.684 7.682 7.680 7.469 7.462 7.460 7.457 7.446 7.442 7.439 7.428 7.425 7.421 7.416 7.411 7.379 7.375 7.373 7.368 7.359 7.356 7.352 7.350 7.341 7.337 7.334 7.331 7.325 7.321 7.308 7.303 7.301 7.289 7.284 7.276 7.268 7.265 7.260 7.251 7.247 7.243 7.242 7.239 7.236 7.223 7.219 7.217 4.485 4.480 4.467 4.462 4.200 4.187 4.182 4.173 4.169 4.164 4.159 4.155 4.147 4.141 4.137 4.132 4.123 4.119 4.114 4.106 4.096 4.079 3.297 3.292 3.257 3.252 3.027 3.019 3.009 3.000 2.986 2.977 2.968 1.133 1.115 1.098
N
O
O
O
EtO2C
Ph
O
O
N
O
EtO2C
O
+
31B
31A
1H, 500 MHz, CDCl3
0.06
1.09
3.13
5.60
1.00
2.14
1.00
0.06
1.06
3.03
10 9 8 7 6 5 4 3 2 1 0
f1 (ppm)

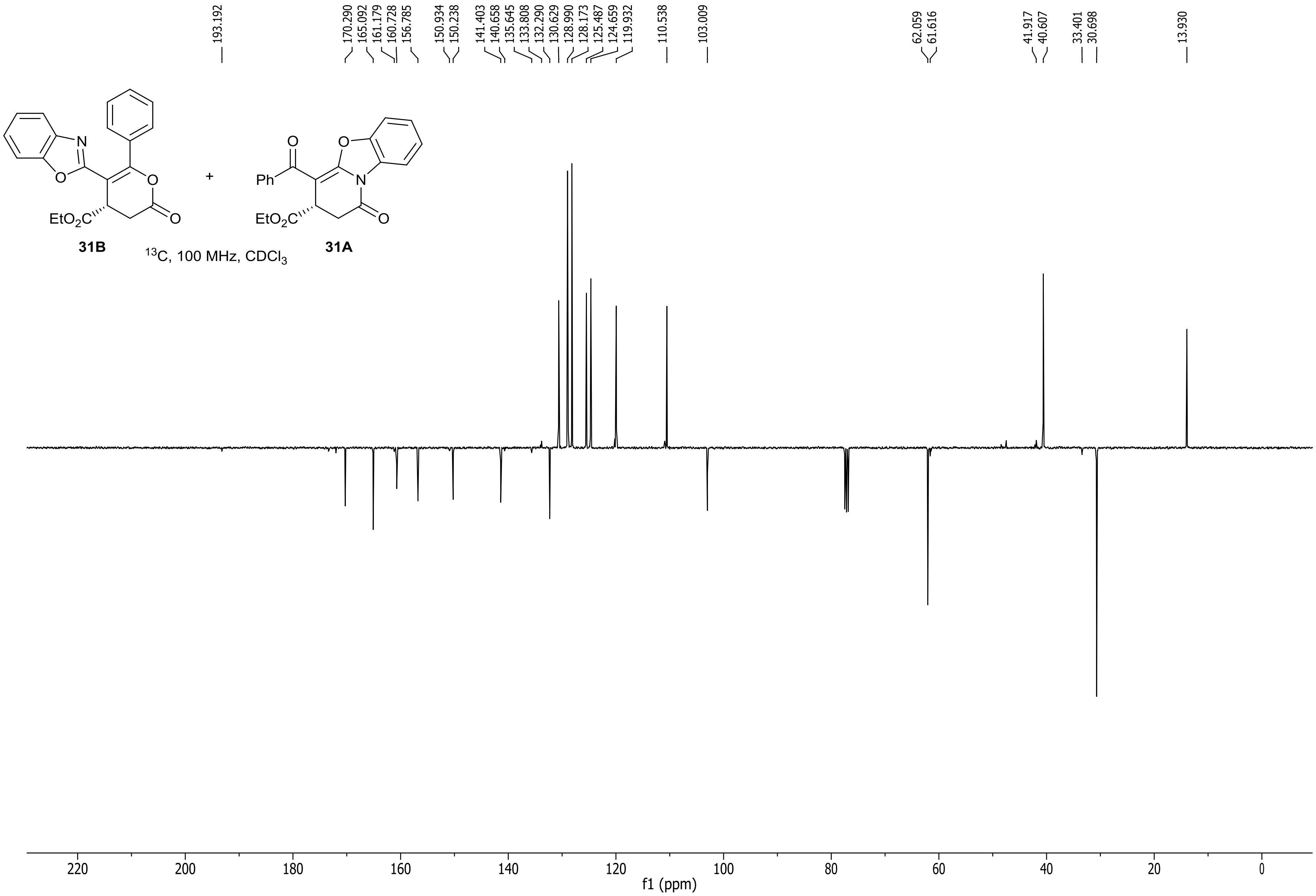

193.192
170.290
165.092
161.179
160.728
156.785
150.934
150.238
141.403
140.658
135.645
133.808
132.290
130.629
128.990
128.173
125.487
124.659
119.932
110.538
103.009
62.059
61.616
41.917
40.607
33.401
30.698
13.930
31B
+
31A
EtO2C
Ph
13C, 100 MHz, CDCl3
220
200
180
160
140
120
100
80
60
40
20
0
f1 (ppm)

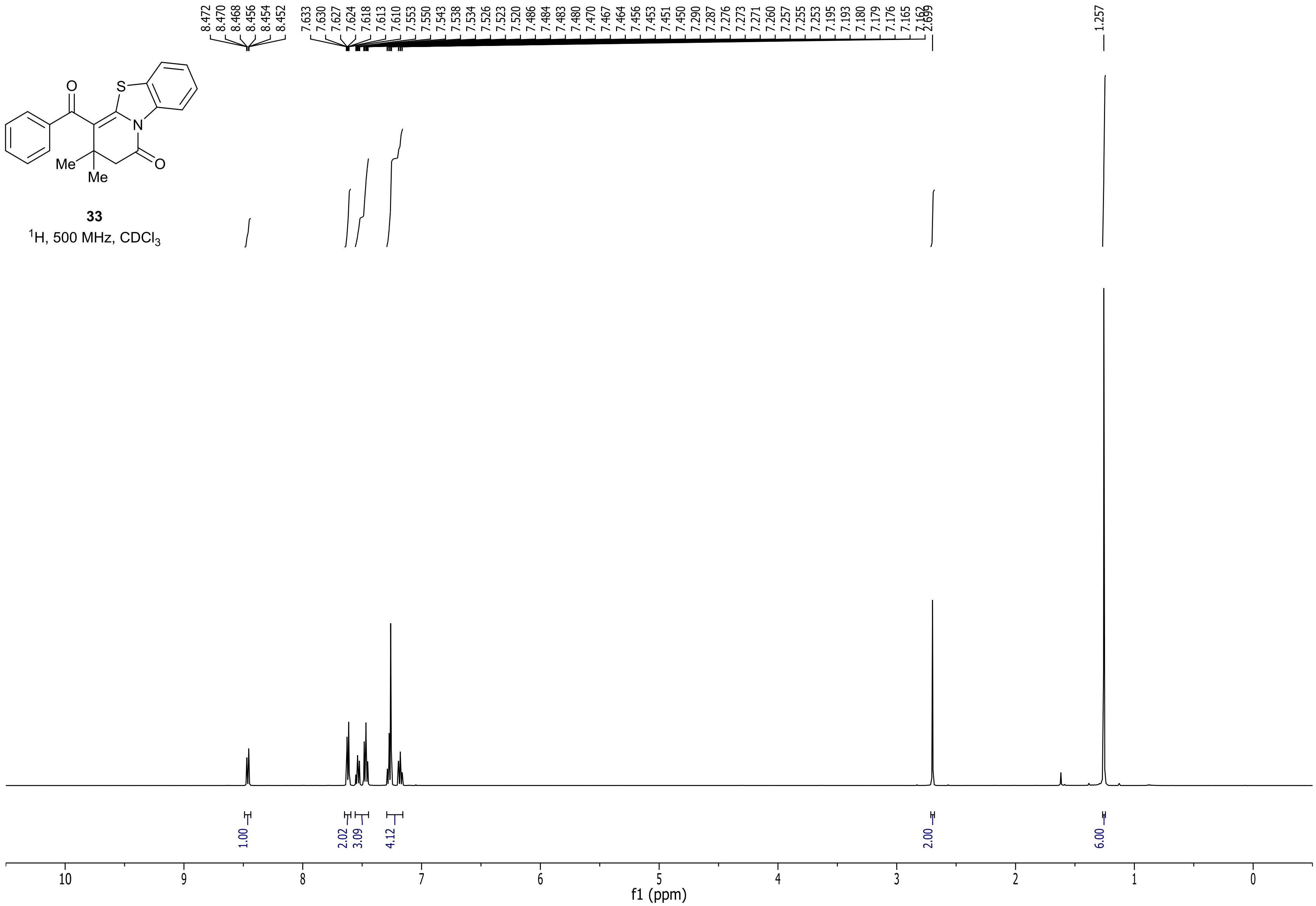

33
^1H, 500 MHz, $CDCl_3$

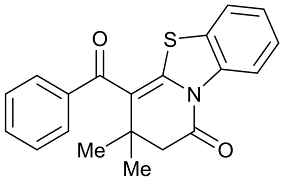

33
^{13}C, 125 MHz, $CDCl_3$

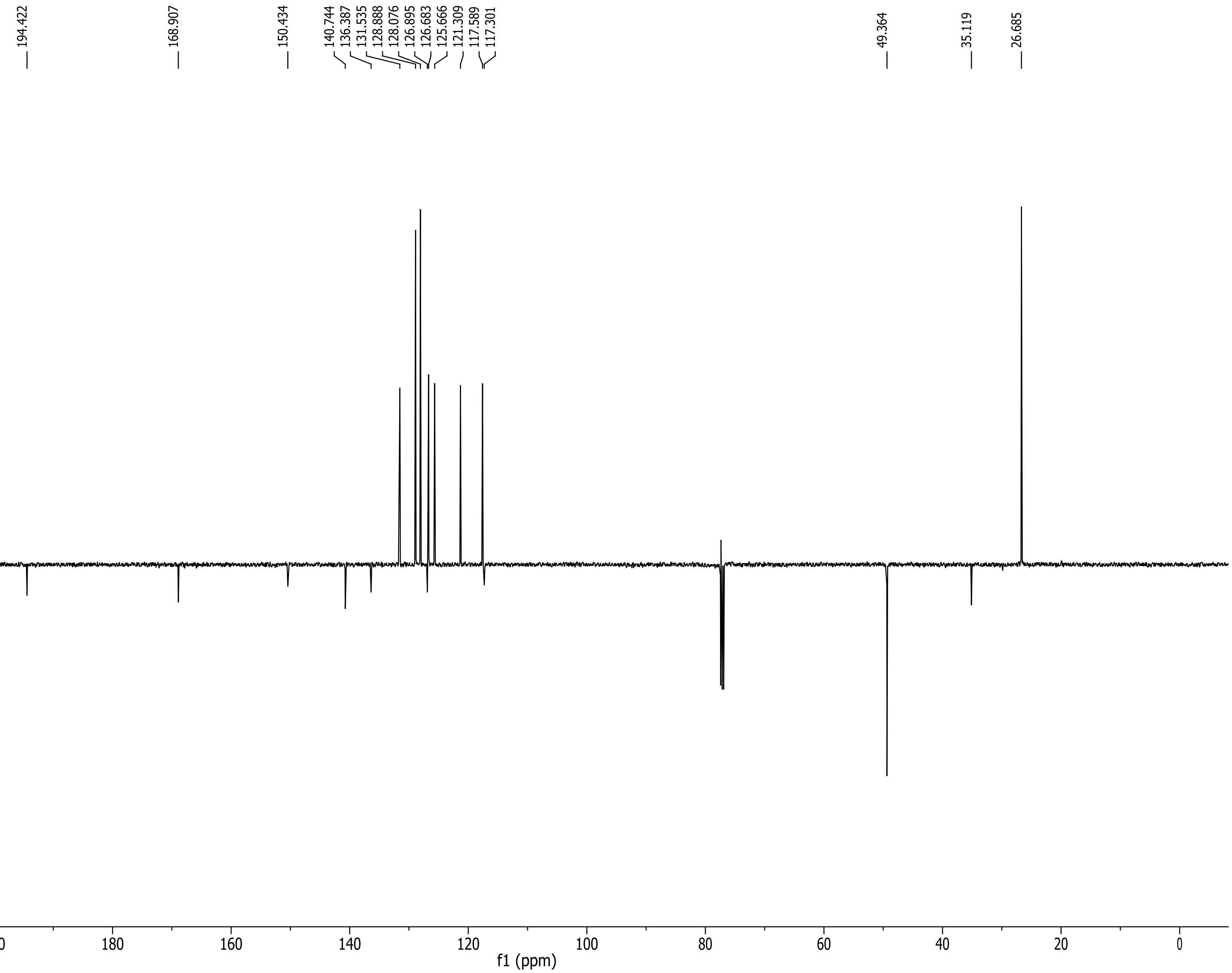

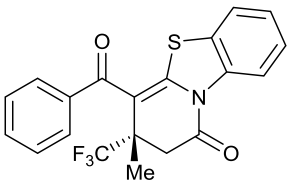

35 (1,4-addition)
^{1}H, 500 MHz, $CDCl_3$

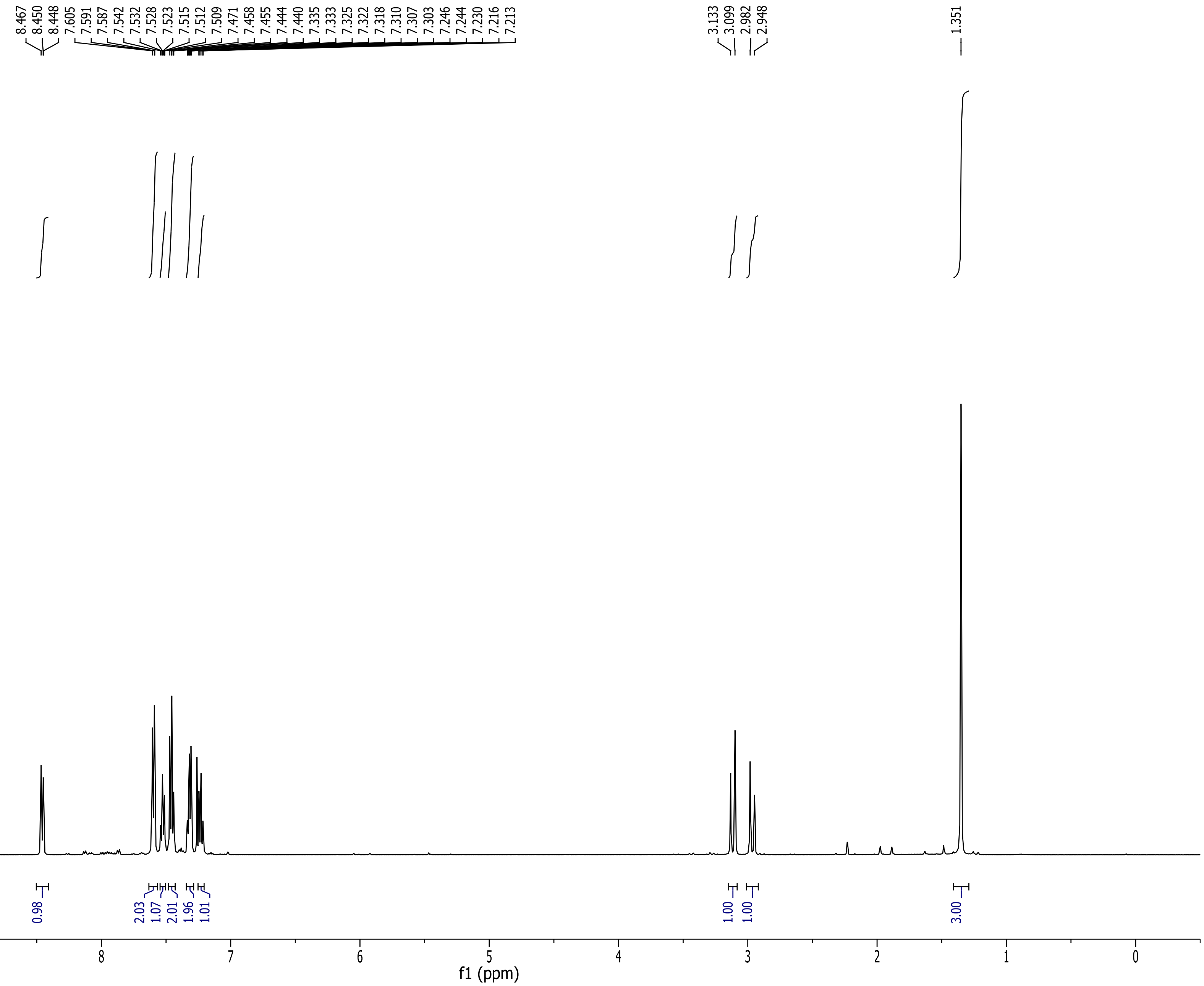

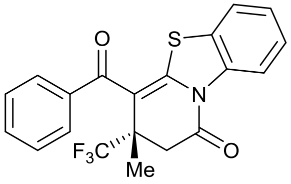

35 (1,4-addition)
^{13}C, 125 MHz, $CDCl_3$

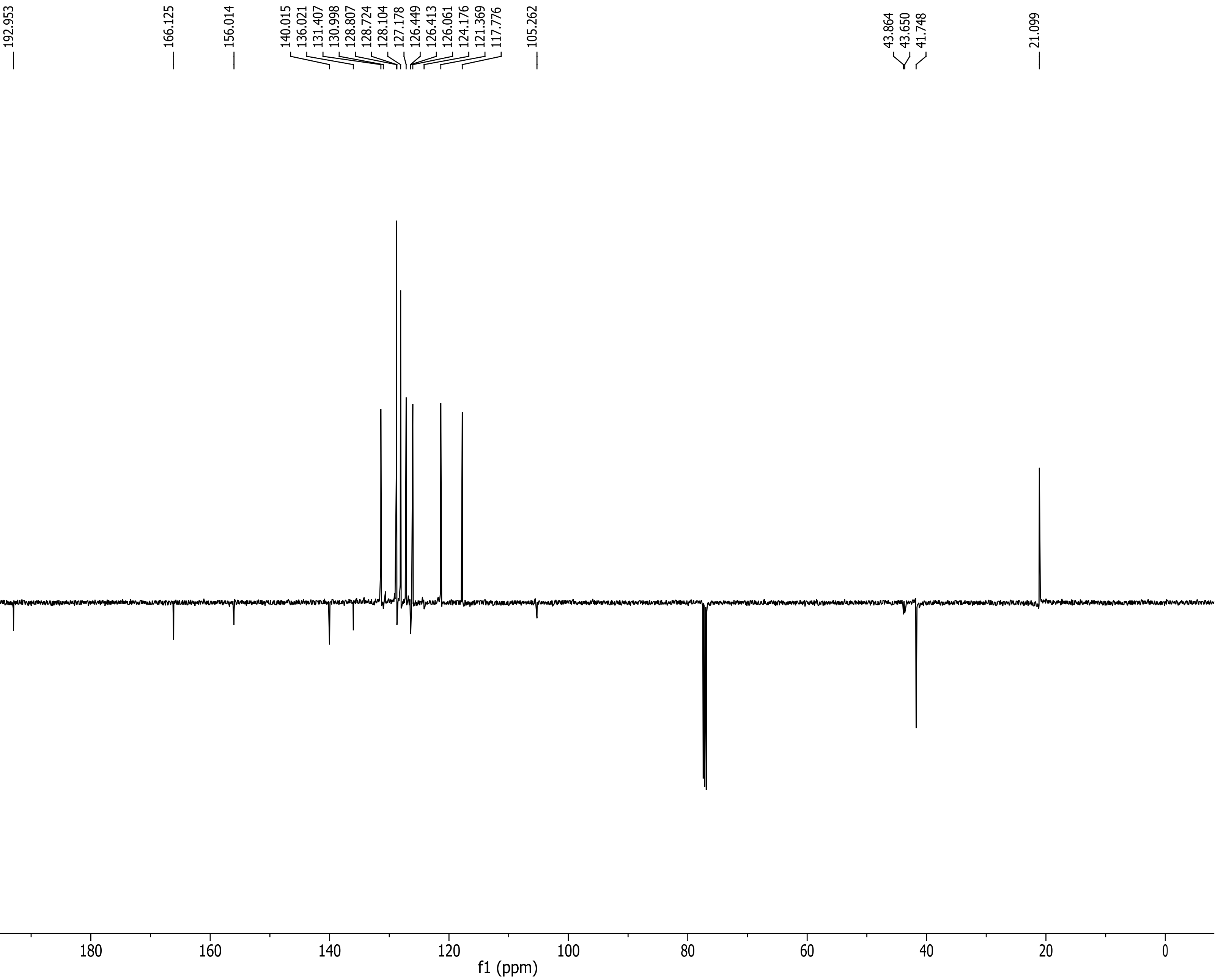

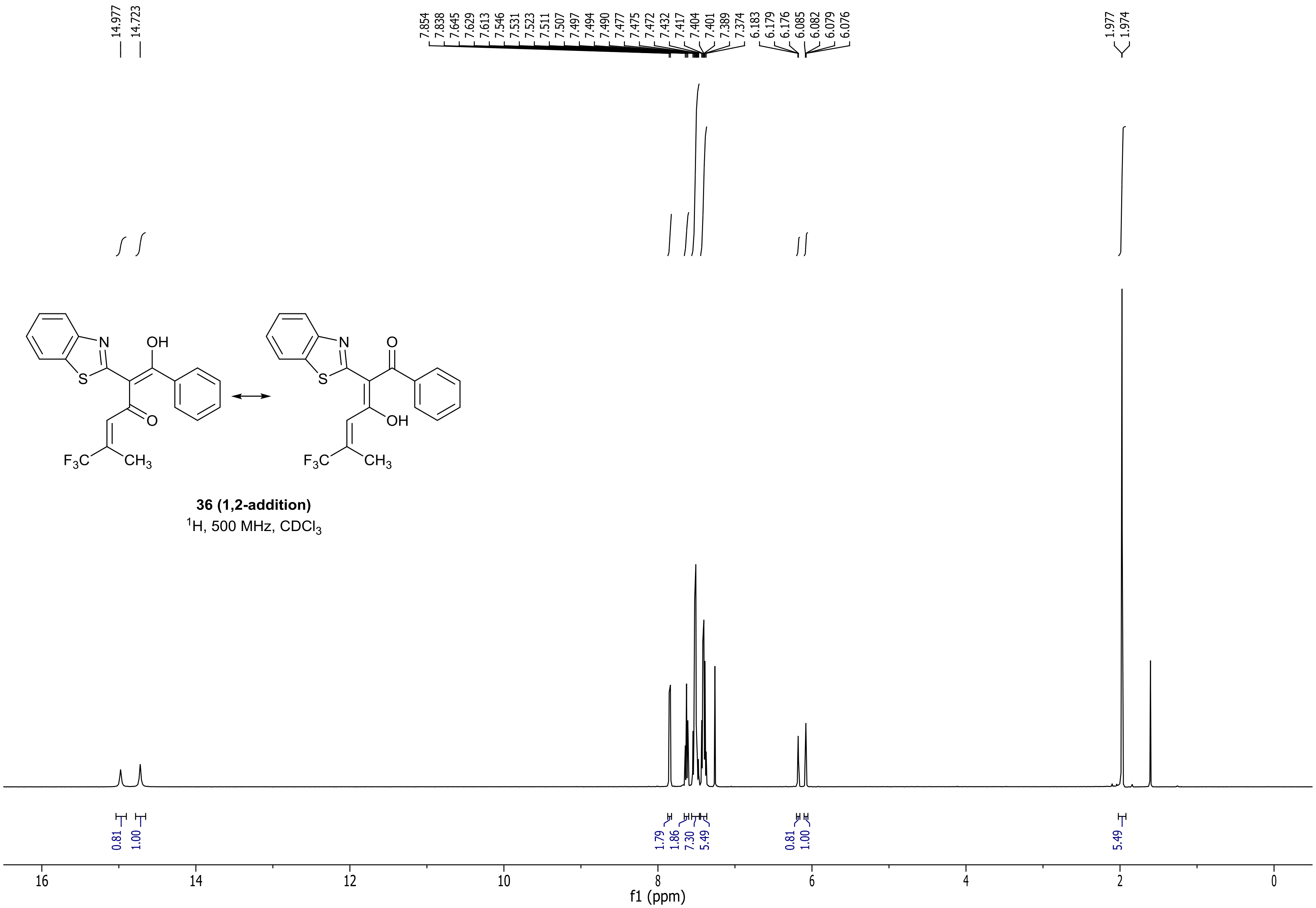
14.977
14.723
7.854
7.838
7.645
7.629
7.613
7.546
7.531
7.523
7.511
7.507
7.497
7.494
7.490
7.477
7.475
7.472
7.432
7.417
7.404
7.401
7.389
7.374
6.183
6.179
6.176
6.085
6.082
6.079
6.076
1.977
1.974
N
OH
S
O
F_3C
CH_3
N
O
S
OH
F_3C
CH_3
36 (1,2-addition)
^1H, 500 MHz, $CDCl_3$
0.81
1.00
1.79
1.86
7.30
5.49
0.81
1.00
5.49
16
14
12
10
8
6
4
2
0
f1 (ppm)

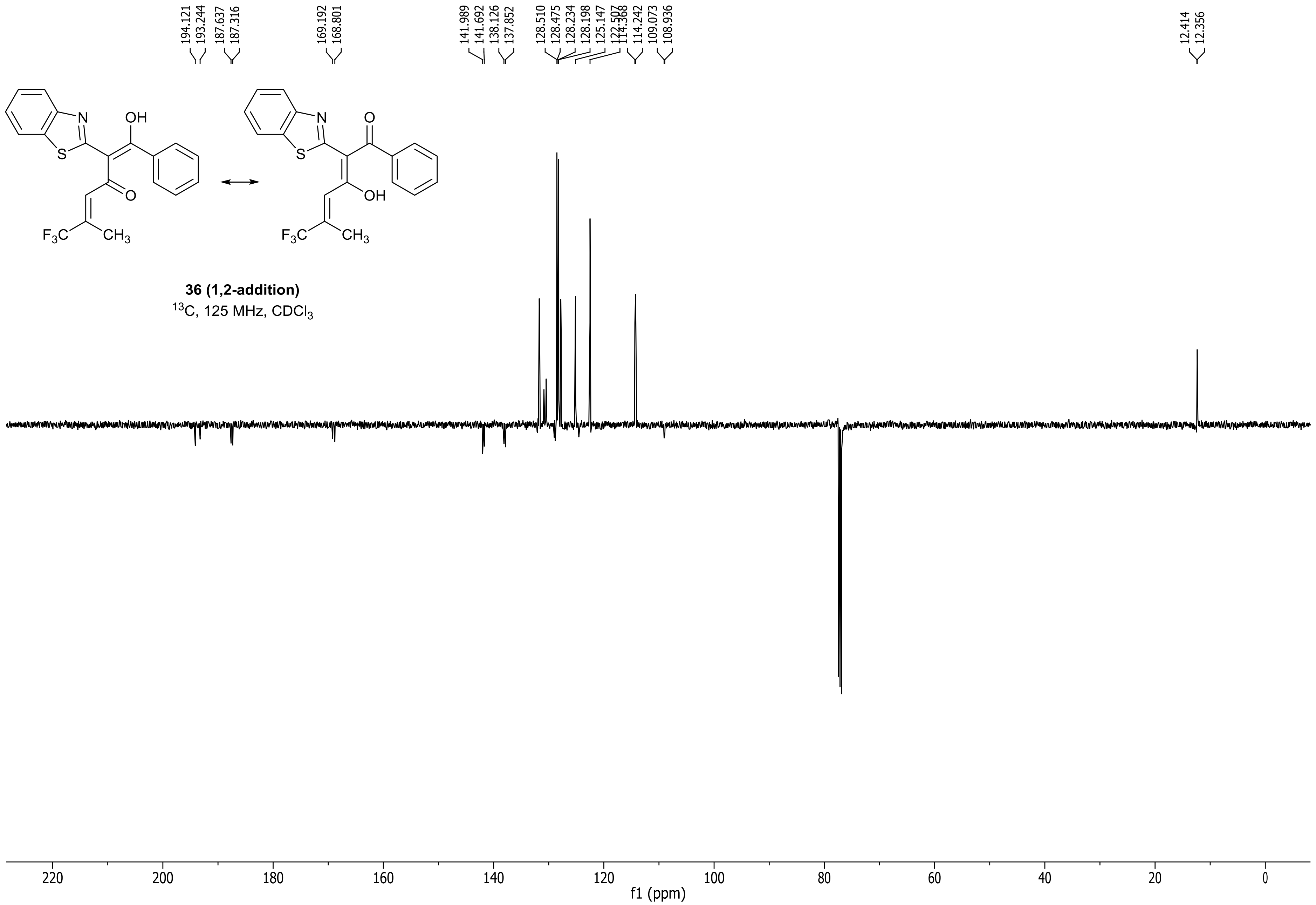
194.121
193.244
187.637
187.316
169.192
168.801
141.989
141.692
138.126
137.852
128.510
128.475
128.234
128.198
125.147
122.507
114.368
114.242
109.073
108.936
12.414
12.356
N
OH
S
O
F3C
CH3
36 (1,2-addition)
13C, 125 MHz, CDCl3
220
200
180
160
140
120
100
80
60
40
20
0
f1 (ppm)

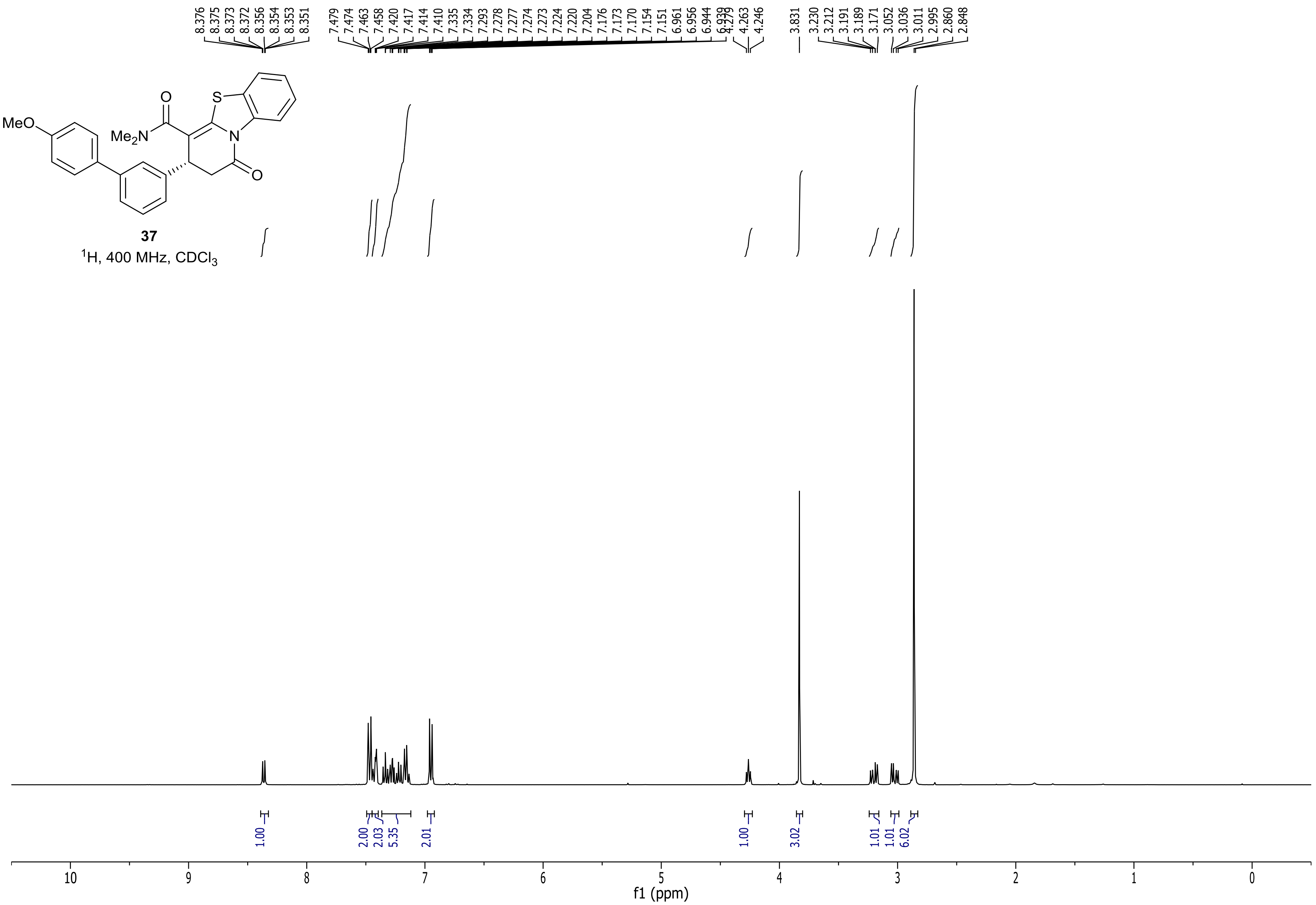
8.376
8.375
8.373
8.372
8.356
8.354
8.353
8.351
7.479
7.474
7.463
7.458
7.420
7.417
7.414
7.410
7.335
7.334
7.293
7.278
7.277
7.274
7.273
7.224
7.220
7.204
7.176
7.173
7.170
7.154
7.151
6.961
6.956
6.944
6.939
4.279
4.263
4.246
3.831
3.230
3.212
3.191
3.189
3.171
3.052
3.036
3.011
2.995
2.860
2.848
MeO
Me2N
O
S
N
O
37
1H, 400 MHz, CDCl3
1.00
2.00
2.03
5.35
2.01
1.00
3.02
1.01
1.01
6.02
10
9
8
7
6
5
4
3
2
1
0
f1 (ppm)

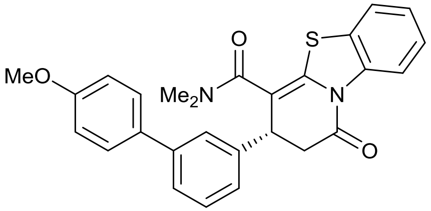

37

^{13}C, 100 MHz, $CDCl_3$

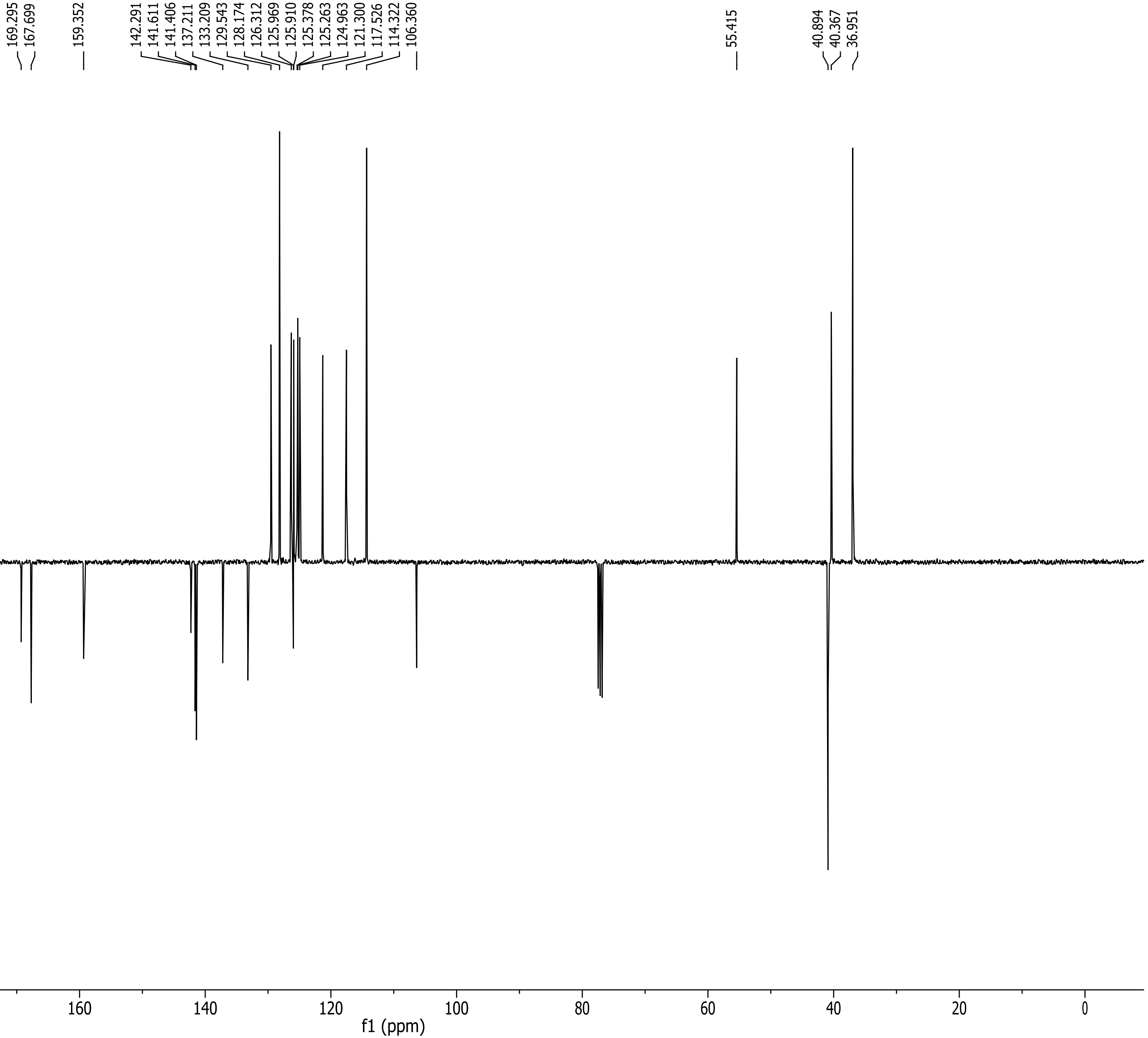

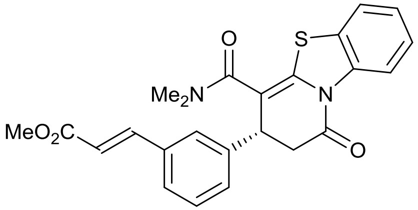

38
^{1}H, 400 MHz, $CDCl_3$

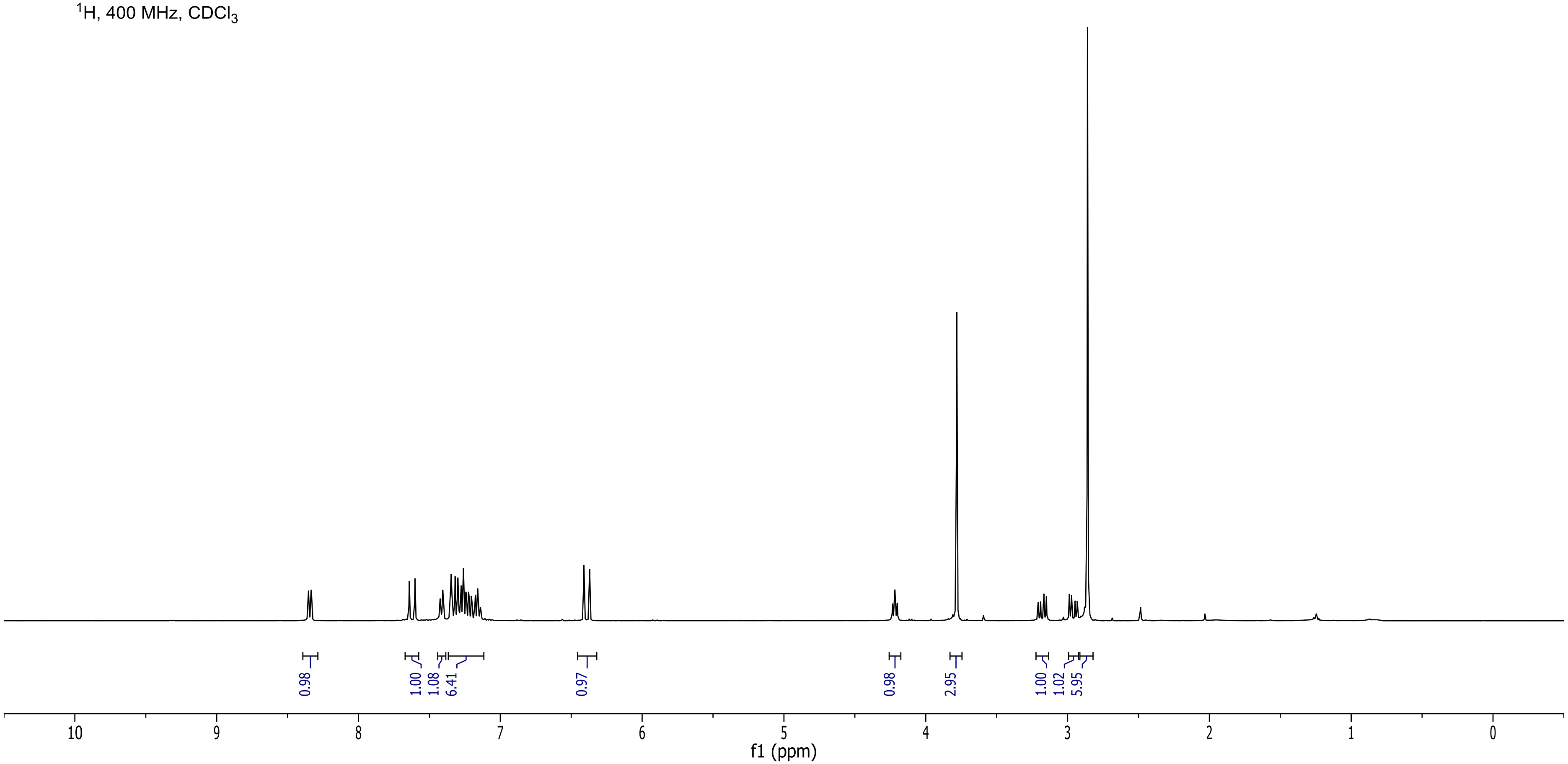

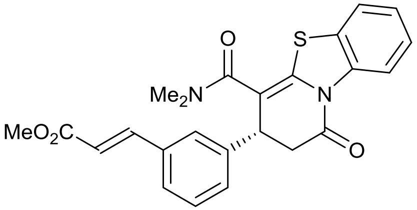

38

^{13}C, 125 MHz, $CDCl_3$

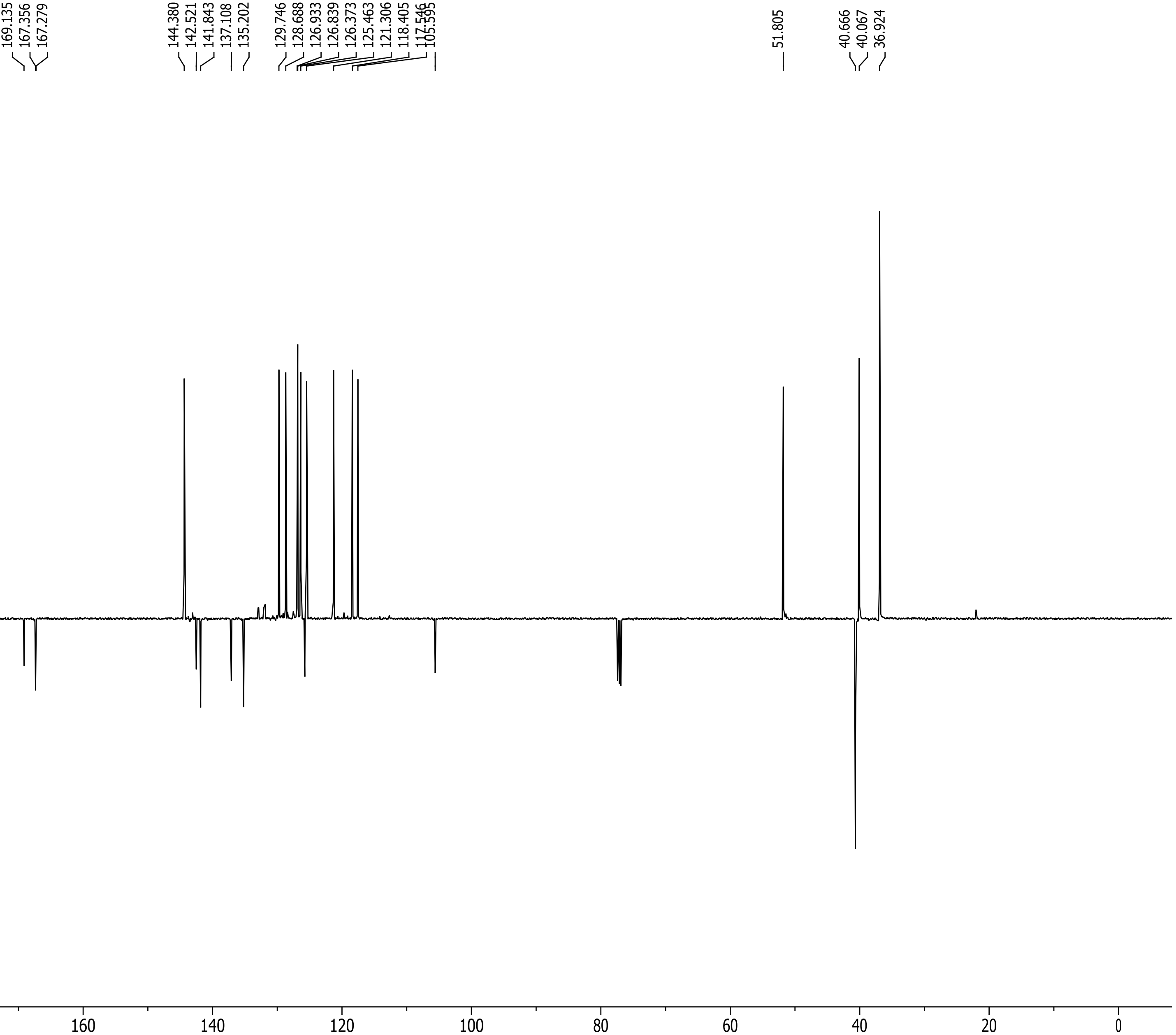

References

1. W. Szymanski, B. Wu, B. Weiner, S. De Wildeman, B. L. Feringa and D. B. Janssen, *J. Org. Chem*., 2009, **74**, 9152-9157.

2. D. R. Brittelli, *J. Org. Chem.*, 1981, **46**, 2514-2520.

3. Y. Shizuri, M. Ojika and K. Yamada, *Tetrahedron Lett.*, 1981 , **22**, 4291-4294.

4. R. S. Menon and M. G. Banwell, *Org. Biomol. Chem.*, 2010, **8**, 5483-5485.

5. W. J. Raich and C. S. Hamilton, *J. Am. Chem. Soc.*, 1957, **79**, 3800-3804.

6. F. Freeman, L. Y. Chang, J. C. Kappos and L. Sumarta, *J. Org. Chem.*, 1987, **52**, 1460-1464.

7. P. Tarrant and R. E. Tayor, *J. Org. Chem.*, 1959, **24**, 1888-1890.

8. H. Nemoto, A. Satoh, K. Fukumoto and C. Kabuto, *J. Org. Chem.*, 1995, **60**, 594-600.

9. P. S. Tiseni and R. Peters, *Angew. Chem. Int. Ed.*, 2007, **46**, 5325-5328.

10. M. Abarbri, J. Thibonnet, J. L. Parrain and A. Duchêne, *Synthesis*, 2002, **4**, 543-551.

11. J. Cabré-Castellví, A. Palomo-Coll and A. L. Palomo-Coll, *Synthesis*, 1981, **8**, 616-620.

12. N. Armesto, F. Miguel, S. Fernandez and V. Gotor, *J. Org. Chem.*, 2003, **68**, 5784-5787.

13. E. R. T. Robinson, C. Fallan, C. Simal, A. M. Z. Slawin and A. D. Smith, *Chem. Sci.*, 2013, **4**, 2193-2200.

14. K. S. Keshavamurthy, Y. D. Vankar, and D. N. Dhar, *Synthesis*, 1982, **6**, 506-508.

15. J. J. Kim, Y. D. Park, W. S. Lee, S. D. Cho and Y. J. Yoon, *Synthesis*, 2003, **10**, 1517-1520.

16. Y. Kubota, S. Tanaka, K. Funabiki and M. Matsui, *Org. Lett.*, 2012, **14**, 4682-4685.

17. C. Fallan and H. W. Lam, *Chem. Eur. J.*, 2012, **18**, 11214-11218.

18. H. C. Shen, F. X. Ding and S. L. Colletti, *Org. Lett.*, 2006, **8**, 1447-1450.

19. H. I. De Silva, S. Chatterjee, W. P. Henry and C. U. Pittman Jr., *Synthesis*, 2012, **44**, 3453-3464.
