# Supplementary material for: Non-bonding 1,5-S···O interactions govern chemo- and enantioselectivity in isothiourea-catalyzed annulations of benzazoles
Source: Chem Sci. 2016 Jul 4;7(12):6919–27. doi: 10.1039/c6sc00940a (PMC5450589; doi:10.1039/c6sc00940a)
Supplement: Supplementary file 2 [file SC-007-C6SC00940A-s002.pdf]

Electronic Supplementary Material (ESI) for Chemical Science.
This journal is © The Royal Society of Chemistry 2016

Non-Bonding S•••O Interactions Govern Chemo- and Enantioselectivity in Isothiourea-Catalyzed Annulations of Benzazoles

Computational Details

Emily R. T. Robinson, Daniel M. Walden, Charlene Fallan, Mark Greenhalgh, Paul Ha-Yeon Cheong, and Andrew D. Smith*,†*

† EaStCHEM, School of Chemistry, University of St Andrews
North Haugh, St Andrews, Fife, UK, KY16 9ST.
Corresponding E-mail: ads10@st-andrews.ac.uk
Homepage: http://ch-www.st-andrews.ac.uk/staff/ads/group/

Complete Gaussian09 authorship **S2**

Computational methods **S2**

Comparison of intermediate V crystal structure to computed structure **S3**

Computed reaction coordinates **S4**

Coordinates, energies, and thermal corrections S4

Annulation facial selectivity **S117**

Model systems **S152**

Anionic nucleophile dihedral rotation energies S152

S•••O vs. O•••O vs. C–H•••O complexes S205

Regioselective structures sans Ph S220

Aromatic interactions S252

Reaction coordinates for 16A and 16B **S258**

References **S337**

Complete Gaussian09 Authorship

Gaussian 09, Revision **D.01**, M. J. Frisch, G. W. Trucks, H. B. Schlegel, G. E. Scuseria, M. A. Robb, J. R. Cheeseman, G. Scalmani, V. Barone, B. Mennucci, G. A. Petersson, H. Nakatsuji, M. Caricato, X. Li, H. P. Hratchian, A. F. Izmaylov, J. Bloino, G. Zheng, J. L. Sonnenberg, M. Hada, M. Ehara, K. Toyota, R. Fukuda, J. Hasegawa, M. Ishida, T. Nakajima, Y. Honda, O. Kitao, H. Nakai, T. Vreven, J. A. Montgomery, Jr., J. E. Peralta, F. Ogliaro, M. Bearpark, J. J. Heyd, E. Brothers, K. N. Kudin, V. N. Staroverov, R. Kobayashi, J. Normand, K. Raghavachari, A. Rendell, J. C. Burant, S. S. Iyengar, J. Tomasi, M. Cossi, N. Rega, J. M. Millam, M. Klene, J. E. Knox, J. B. Cross, V. Bakken, C. Adamo, J. Jaramillo, R. Gomperts, R. E. Stratmann, O. Yazyev, A. J. Austin, R. Cammi, C. Pomelli, J. W. Ochterski, R. L. Martin, K. Morokuma, V. G. Zakrzewski, G. A. Voth, P. Salvador, J. J. Dannenberg, S. Dapprich, A. D. Daniels, Ö. Farkas, J. B. Foresman, J. V. Ortiz, J. Cioslowski, and D. J. Fox, Gaussian, Inc., Wallingford CT, 2009.

Computational Methods

All computations were completed using the Gaussian09 computational package. Exhaustive manual conformational searches were completed to ensure all pertinent intermediates and transition structures were investigated. All minima on the potential energy surface were located with optimizations using M06-2X/6-31G(d)[i] with implicit solvent modelled with the polarized continuum model[ii] (PCM) using the dielectric constant of tetrahydrofuran (THF). Minima were confirmed with vibrational frequency computations, with ground state minima having zero imaginary vibrational frequencies and transition state minima having one imaginary frequency corresponding to the vibrational mode of the forming/breaking bond. Energy refinements were done with M06-2X/6-31+G(d,p) using PCM in THF to account for the energy of solvation. These self-consistent field (SCF) energies were then converted to

Gibbs free energies using the Gibbs thermal correction factor of the respective optimized structure. All energies are reported as kcal/mol and all barriers shown associated with structures and on the reaction coordinates are calculated as relative energies from starting material. All distances shown are given in Ångstroms (Å). All 3D structure images were rendered in CylView visualization software.[iii] Both predicted experimental barriers seen in the reaction coordinates calculated using the Eyring equation.[iv]

Comparison of intermediate V crystal structure to computed structure

α,β-unsaturated acyl ammonium

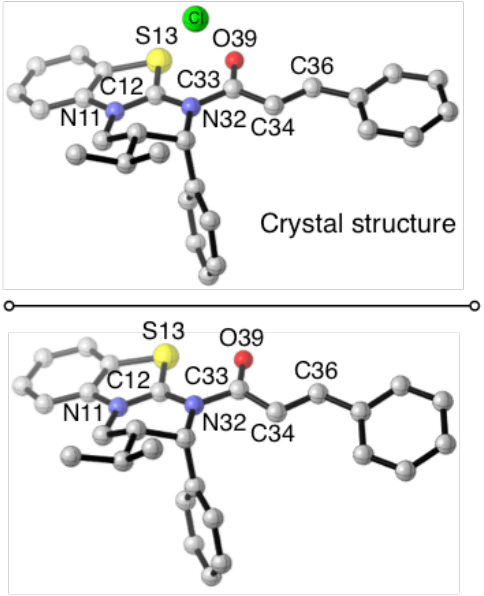

M062X/6-31G(d)/PCM(THF) optimized structure

Parameter	Computed	Crystal
S13–O39	2.57	2.48
S13–C12	1.74	1.74
N11–C12	1.33	1.33
N32–C12	1.35	1.36
N32–C33	1.43	1.41
C33–C34	1.47	1.47
C34–C36	1.35	1.33
C36–C37	1.46	1.46
S13–C12–N11	113	114
S13–C12–N32	125	125
C12–N32–C33	119	118
N32–C33–O39	119	118
O39–C33–C34	124	122
C12–N32–C33–O39	11	6

Computed Reaction Coordinates

All computed relative energies (intermediates and transition states) are relative to the reaction's starting material ($\Delta G = 0.0$ = HyperBTM catalyst, cinnamic anhydride, benzoxazole nucleophile, and Hunig's base). Experimental barriers (ΔG_{expt}) computed using the Eyring equation.[iv]

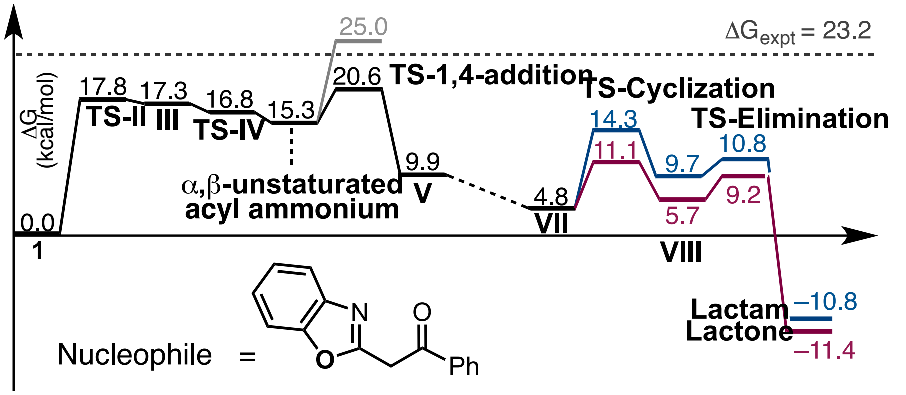

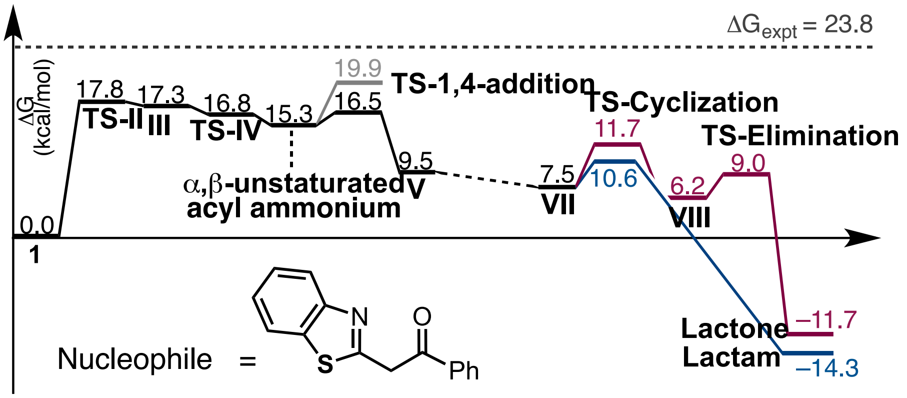

Coordinates, energies, and thermal corrections:

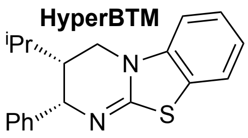

1

Supporting Information: 0000-HyperBTM-2.log

Using Gaussian 09: AM64L-G09RevC.01 23-Sep-2011

===

#M062X/6-31G(d) scf=(maxcycle=300,direct,tight) density=current
SCRF=(PCM,SOLVENT=THF) opt=(maxcycle=250,gdiis) iop(1/8=18) freq=noraman
#N Geom=AllCheck Guess=TCheck SCRF=Check Test GenChk RM062X/6-31G(d) Freq

---

Pointgroup= C1 Stoichiometry= C19H20N2S C1[X(C19H20N2S)] #Atoms= 42
Charge = 0 Multiplicity = 1

SCF Energy= -1243.38807245 Predicted Change= -3.780822D-09

===

Optimization completed. {Found 2 times}

Item	Max Val.	Criteria	Pass?	RMS Val.	Criteria	Pass?
Force	0.00000 ||	0.00045	[YES]	0.00000 ||	0.00030	[YES]
Displ	0.00101 ||	0.00180	[YES]	0.00101 ||	0.00180	[YES]

Atomic Type	Coordinates (Angstroms) X	Y	Z
C	-4.267420	0.777623	1.763328
H	-4.684114	1.285391	2.627153
C	-2.912754	0.937488	1.470282
H	-2.278106	1.553704	2.098307
C	-2.393224	0.279698	0.359486
C	-3.216158	-0.534538	-0.433990
C	-4.561425	-0.690580	-0.138925
H	-5.191996	-1.319702	-0.758905
C	-5.086194	-0.024034	0.970244
H	-6.137209	-0.136761	1.213712
N	-1.079739	0.321747	-0.090745

C	-0.804409	-0.457605	-1.199410
S	-2.300558	-1.260125	-1.754835
C	-0.045848	1.170346	0.485639
H	0.351326	0.714204	1.403846
H	-0.512145	2.122687	0.753275
C	1.066640	1.396643	-0.540502
C	2.246292	2.178181	0.064661
C	3.241229	2.603820	-1.018707
H	2.752334	3.264235	-1.745136
H	3.660221	1.756559	-1.567170
H	4.075753	3.154365	-0.573555
H	2.761835	1.517410	0.775054
C	1.774395	3.421066	0.827066
H	1.158582	4.060698	0.182599
H	2.635818	4.009927	1.156437
H	1.190070	3.168491	1.716731
H	0.638407	2.005826	-1.350865
C	1.465749	0.044231	-1.190441
C	2.205648	-0.880832	-0.232987
H	2.145325	0.262760	-2.020410
N	0.310836	-0.639398	-1.780956
C	1.523195	-1.676383	0.693223
H	0.436374	-1.677091	0.713878
C	2.219574	-2.488734	1.584790
H	1.670389	-3.099287	2.295272
C	3.611903	-2.526877	1.560180
H	4.154372	-3.163346	2.252487
C	4.302157	-1.750730	0.632764
H	5.386857	-1.782168	0.593867
C	3.601866	-0.939270	-0.256699
H	4.149870	-0.349419	-0.987322

Statistical Thermodynamic Analysis

Temperature= 298.150 Kelvin   Pressure= 1.00000 Atm

==

SCF Energy= -1243.38807245 Predicted Change= -3.780822D-09

Zero-point correction (ZPE)= -1243.0335 0.35456

Internal Energy (U)= -1243.0150 0.37305

Enthalpy (H)= -1243.0140 0.37399

Gibbs Free Energy (G)= -1243.0803 0.30768

Frequencies -- 31.5000 37.6909 62.6480

==

#M062X/6-31+G(d,p) scf=(maxcycle=300,direct,tight) density=current

SCRF=(PCM,SOLVENT=THF)

Pointgroup= C1 Stoichiometry= C19H20N2S C1[X(C19H20N2S)] #Atoms= 42

Charge = 0 Multiplicity = 1

SCF Energy= -1243.43328732

==

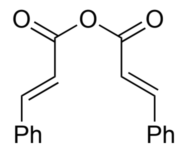

Supporting Information: 0000-Cinnamic-anhydride-6.log

Using Gaussian 09: AM64L-G09RevC.01 23-Sep-2011

==

#M062X/6-31G(d) scf=(maxcycle=300,direct,tight) density=current

SCRF=(PCM,SOLVENT=THF) opt=(maxcycle=250,gdiis) iop(1/8=18) freq=noraman

#N Geom=AllCheck Guess=TCheck SCRF=Check Test GenChk RM062X/6-31G(d) Freq

----

Pointgroup= C1 Stoichiometry= C18H14O3 C1[X(C18H14O3)] #Atoms= 35

Charge = 0 Multiplicity = 1

SCF Energy= -919.635362424 Predicted Change= -1.533633D-09

==

Optimization completed. {Found 2 times}

Item	Max Val.	Criteria	Pass?	RMS Val.	Criteria	Pass?
Force	0.00000 ||	0.00045	[YES]	0.00000 ||	0.00030	[YES]
Displ	0.00109 ||	0.00180	[YES]	0.00109 ||	0.00180	[YES]

Atomic Type	Coordinates (Angstroms) X	Y	Z
O	0.269734	1.060641	-0.253978
C	-0.951475	1.446848	0.262854
C	-1.978729	0.445247	-0.065337
H	-1.668669	-0.404667	-0.662696
C	-3.233985	0.635940	0.367431
H	-3.422680	1.537466	0.949744
C	-4.391736	-0.230579	0.135417
O	-1.099514	2.444158	0.916855
C	1.266422	1.997513	-0.460555
C	2.613193	1.415077	-0.392881
H	3.401058	2.122779	-0.628012
C	2.851797	0.136409	-0.063622
H	1.998959	-0.504506	0.150779
C	4.166623	-0.505228	0.034384
O	1.029833	3.147255	-0.718678
C	-5.642562	0.189604	0.606536
H	-5.720568	1.139071	1.129559
C	-6.776991	-0.590447	0.409011
H	-7.738781	-0.249131	0.778073
C	-6.673791	-1.806624	-0.261192
H	-7.556335	-2.419202	-0.417434
C	-5.432179	-2.238703	-0.731880

H	-5.348986	-3.187958	-1.251628
C	-4.300523	-1.459171	-0.536723
H	-3.340897	-1.809433	-0.904485
C	4.221080	-1.878432	0.307200
H	3.293769	-2.429144	0.442178
C	5.442204	-2.538002	0.403140
H	5.466917	-3.602386	0.613676
C	6.628263	-1.829340	0.230747
H	7.583568	-2.339163	0.307312
C	6.587626	-0.459639	-0.036499
H	7.511189	0.095407	-0.166925
C	5.369188	0.197906	-0.134144
H	5.354475	1.264136	-0.337054

Statistical Thermodynamic Analysis

Temperature= 298.150 Kelvin Pressure= 1.00000 Atm

==

SCF Energy= -919.635362424 Predicted Change= -1.533633D-09

Zero-point correction (ZPE)= -919.3592 0.27614

Internal Energy (U)= -919.3410 0.29429

Enthalpy (H)= -919.3401 0.29523

Gibbs Free Energy (G)= -919.4107 0.22457

Frequencies -- 8.7854 13.5528 33.4022

==

#M062X/6-31+G(d,p) scf=(maxcycle=300,direct,tight) density=current SCRF=(PCM,SOLVENT=THF)

Pointgroup= C1 Stoichiometry= C18H14O3 C1[X(C18H14O3)] #Atoms= 35

Charge = 0 Multiplicity = 1

SCF Energy= -919.681633796

==

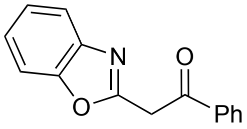

Supporting Information: 0000-Benzoxazole-1.log

Using Gaussian 09: AM64L-G09RevC.01 23-Sep-2011

==

#m062X/6-31G(d) scf=(maxcycle=300,direct,tight) density=current

SCRF=(PCM,SOLVENT=THF) opt=(maxcycle=250) freq=noraman

#N Geom=AllCheck Guess=TCheck SCRF=Check GenChk RM062X/6-31G(d) Freq

----

Pointgroup= C1 Stoichiometry= C15H11NO2 C1[X(C15H11NO2)] #Atoms= 29

Charge = 0 Multiplicity = 1

SCF Energy= -783.115706156 Predicted Change= -6.173327D-10

==

Optimization completed. {Found 2 times}

Item	Max Val.	Criteria	Pass?	RMS Val.	Criteria	Pass?
Force	0.00000 ||	0.00045	[YES]	0.00000 ||	0.00030	[YES]
Displ	0.00128 ||	0.00180	[YES]	0.00128 ||	0.00180	[YES]

Atomic Type	Coordinates (Angstroms) X	Y	Z
C	3.331916	-1.188450	-0.910631
H	3.402428	-1.746894	-1.837718
C	4.245108	-1.361462	0.123280
H	5.053595	-2.075358	0.004976
C	4.148386	-0.634646	1.322312
H	4.884174	-0.800331	2.102297
C	3.133554	0.294276	1.536914
H	3.048482	0.860714	2.456943

C	2.236272	0.446705	0.492261
C	2.310660	-0.260928	-0.708123
O	1.148776	1.272896	0.409622
C	0.628323	1.024167	-0.825724
C	-0.592151	1.798257	-1.187973
C	-1.717230	1.604054	-0.166430
C	-2.341314	0.255136	-0.026741
O	-2.080769	2.545912	0.507177
H	-0.908606	1.486994	-2.186413
H	-0.362179	2.866616	-1.202566
N	1.252186	0.141335	-1.527942
C	-3.314637	0.084912	0.965152
H	-3.569249	0.931428	1.594382
C	-3.936645	-1.145013	1.130590
H	-4.690190	-1.273729	1.900824
C	-3.591479	-2.214910	0.303326
H	-4.076729	-3.177614	0.431547
C	-2.624929	-2.051783	-0.686279
H	-2.356925	-2.884505	-1.328258
C	-1.998049	-0.819855	-0.853775
H	-1.240862	-0.709893	-1.623760

Statistical Thermodynamic Analysis

Temperature= 298.150 Kelvin Pressure= 1.00000 Atm

==

SCF Energy= -783.115706156 Predicted Change= -6.173327D-10

Zero-point correction (ZPE)= -782.8871 0.22858

Internal Energy (U)= -782.8736 0.24204

Enthalpy (H)= -782.8727 0.24299

Gibbs Free Energy (G)= -782.9298 0.18581

Frequencies -- 16.7098 31.9724 59.8107

==

#m062X/6-31+G(d,p) scf=(maxcycle=300,direct,tight) density=current
SCRF=(PCM,SOLVENT=THF)

Pointgroup= C1 Stoichiometry= C15H11NO2 C1[X(C15H11NO2)] #Atoms= 29
Charge = 0 Multiplicity = 1

SCF Energy= -783.152457418

===

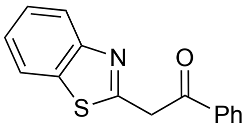

Supporting Information: 0000-Benzothioazole-1.log

Using Gaussian 09: AM64L-G09RevC.01 23-Sep-2011

===

#m062X/6-31G(d) scf=(maxcycle=300,direct,tight) density=current
SCRF=(PCM,SOLVENT=THF) opt=(maxcycle=250) freq=noraman
#N Geom=AllCheck Guess=TCheck SCRF=Check GenChk RM062X/6-31G(d) Freq

----

Pointgroup= C1 Stoichiometry= C15H11NOS C1[X(C15H11NOS)] #Atoms= 29
Charge = 0 Multiplicity = 1

SCF Energy= -1106.08031711 Predicted Change= -7.035219D-10

===

Optimization completed. {Found 2 times}

Item	Max Val.		Criteria	Pass?	RMS Val.		Criteria	Pass?
Force	0.00000	||	0.00045	[YES]	0.00000	||	0.00030	[YES]
Displ	0.00065	||	0.00180	[YES]	0.00065	||	0.00180	[YES]

Atomic Coordinates (Angstroms)
Type X Y Z

C	-3.172204	1.382292	0.967016
H	-3.024349	1.964193	1.870931
C	-4.243198	1.609461	0.118637
H	-4.960297	2.389038	0.354564
C	-4.414989	0.844380	-1.046456
H	-5.261705	1.042107	-1.695934
C	-3.520167	-0.161311	-1.383610
H	-3.655063	-0.750495	-2.284591
C	-2.441849	-0.389492	-0.527300
C	-2.259290	0.371935	0.643352
S	-1.156770	-1.560607	-0.655131
C	-0.496046	-0.936055	0.855029
C	0.761017	-1.529702	1.421621
C	1.910767	-1.472732	0.415024
C	2.540802	-0.154592	0.109491
O	2.277115	-2.493992	-0.132633
H	0.605019	-2.584770	1.661718
H	1.003826	-0.986489	2.338371
N	-1.143954	0.036196	1.399381
C	2.103580	1.037874	0.696015
H	1.270281	1.041151	1.392068
C	2.727380	2.239459	0.371442
H	2.383832	3.164187	0.823674
C	3.787195	2.254648	-0.532227
H	4.272481	3.193390	-0.781427
C	4.226679	1.066824	-1.118717
H	5.053254	1.080220	-1.821963
C	3.604274	-0.132349	-0.800253
H	3.928079	-1.068093	-1.244311

Statistical Thermodynamic Analysis

Temperature= 298.150 Kelvin Pressure= 1.00000 Atm

===

SCF Energy= -1106.08031711 Predicted Change= -7.035219D-10

Zero-point correction (ZPE)= -1105.8558 0.22448

Internal Energy (U)= -1105.8417 0.23860

Enthalpy (H)= -1105.8407 0.23954

Gibbs Free Energy (G)= -1105.8996 0.18066

Frequencies -- 18.2847 25.8674 47.4198

===

#m062X/6-31+G(d,p) scf=(maxcycle=300,direct,tight) density=current

SCRF=(PCM,SOLVENT=THF)

Pointgroup= C1 Stoichiometry= C15H11NOS C1[X(C15H11NOS)] #Atoms= 29

Charge = 0 Multiplicity = 1

SCF Energy= -1106.11556028

===

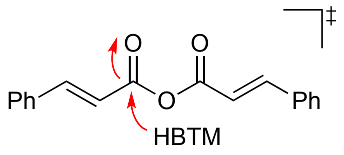

TS-II

Supporting Information: 0005-TS-HYPERBTM-Anhydride-Re-attack-6.log

----

Using Gaussian 09: AM64L-G09RevC.01 23-Sep-2011

===

#m062X/6-31G(d) scf=(maxcycle=300,direct,tight) density=current

SCRF=(PCM,SOLVENT=THF) opt=(maxcycle=250,ts,calcfc,noeigentest)

freq=noraman

#N Geom=AllCheck Guess=TCheck SCRF=Check GenChk RM062X/6-31G(d) Freq

----

Pointgroup= C1 Stoichiometry= C37H34N2O3S C1[X(C37H34N2O3S)] #Atoms= 77

Charge = 0 Multiplicity = 1

SCF Energy= -2163.02240715 Predicted Change= -3.756551D-09

==

Optimization completed on the basis of negligible forces. {Found 2 times}

Item	Max Val.	Criteria	Pass?	RMS Val.	Criteria	Pass?
Force	0.00000 ||	0.00045	[YES]	0.00000 ||	0.00030	[YES]
Displ	0.01256 ||	0.00180	[NO]	0.01256 ||	0.00180	[NO]

Atomic Type	Coordinates (Angstroms) X	Y	Z
C	1.931469	5.979914	-0.017960
H	2.120910	6.896501	0.530688
C	1.178514	4.972837	0.582452
H	0.775295	5.099706	1.581312
C	0.951699	3.807111	-0.142085
C	1.451468	3.654522	-1.437697
C	2.198678	4.661679	-2.033460
H	2.586100	4.541202	-3.039903
C	2.439263	5.828089	-1.309143
H	3.022782	6.624660	-1.758164
N	0.241013	2.683271	0.284524
C	0.155835	1.677411	-0.631662
S	0.963560	2.114628	-2.146214
C	-0.273053	2.531763	1.644645
H	-1.216390	3.085640	1.743935
H	0.462025	2.978961	2.317720
C	-0.461091	1.052775	1.975369
C	-1.145427	0.855903	3.339244
C	-1.136407	-0.620258	3.745297
H	-0.106635	-0.993132	3.799328
H	-1.684483	-1.255221	3.044748
H	-1.593683	-0.745885	4.731330

H	-2.187922	1.191444	3.248521
C	-0.467999	1.686386	4.434378
H	-0.577741	2.762737	4.271974
H	0.602874	1.454650	4.489142
H	-0.910056	1.456601	5.408442
H	0.535630	0.590635	2.021252
C	-1.188311	0.342069	0.812060
C	-2.647551	0.735537	0.639444
H	-1.141011	-0.730050	1.002963
N	-0.435203	0.547775	-0.426744
C	-0.383687	-0.661076	-1.753526
O	1.034876	-1.070492	-1.621203
C	1.587771	-1.500357	-0.484415
C	3.028871	-1.785100	-0.683864
H	3.425335	-1.610891	-1.678366
C	3.767561	-2.232624	0.339503
C	5.199092	-2.553492	0.321858
H	3.256699	-2.377767	1.290984
O	1.004545	-1.636602	0.576325
C	-1.308725	-1.740323	-1.256260
H	-0.917261	-2.471358	-0.556436
C	-2.583865	-1.733006	-1.650591
C	-3.626068	-2.677925	-1.220540
H	-2.901697	-0.926261	-2.310632
O	-0.586278	-0.072522	-2.817086
C	-3.028024	1.925295	0.010845
H	-2.277649	2.591304	-0.407186
C	-4.373550	2.261575	-0.117881
H	-4.649717	3.187379	-0.613288
C	-5.360592	1.410214	0.373573
H	-6.409075	1.671260	0.267862
C	-4.993259	0.213718	0.984615
H	-5.752385	-0.473247	1.346421

C	-3.647367	-0.119967	1.110228
H	-3.371072	-1.074777	1.552129
C	6.008183	-2.353855	-0.806833
H	5.581273	-1.943284	-1.716747
C	7.359267	-2.671564	-0.770542
H	7.973480	-2.509384	-1.650890
C	7.928416	-3.194617	0.391944
H	8.985282	-3.441393	0.416001
C	7.136866	-3.396553	1.519283
H	7.571801	-3.802112	2.427293
C	5.783184	-3.076437	1.482675
H	5.164349	-3.233260	2.362564
C	-3.326047	-3.916234	-0.636093
H	-2.290129	-4.226279	-0.532201
C	-4.342908	-4.759908	-0.204626
H	-4.094619	-5.717533	0.243021
C	-5.678379	-4.384086	-0.352428
H	-6.470781	-5.045822	-0.016400
C	-5.989596	-3.160298	-0.940363
H	-7.026360	-2.861485	-1.062527
C	-4.970398	-2.317653	-1.374359
H	-5.210940	-1.356754	-1.822721

Statistical Thermodynamic Analysis

Temperature= 298.150 Kelvin Pressure= 1.00000 Atm

==

SCF Energy= -2163.02240715 Predicted Change= -3.756551D-09

Zero-point correction (ZPE)= -2162.3904 0.63197

Internal Energy (U)= -2162.3533 0.66902

Enthalpy (H)= -2162.3524 0.66996

Gibbs Free Energy (G)= -2162.4645 0.55783

Frequencies -- -181.5675 5.7549 11.0832

===

#m062X/6-31+G(d,p) scf=(maxcycle=300,direct,tight) density=current
SCRF=(PCM,SOLVENT=THF)

Pointgroup= C1 Stoichiometry= C37H34N2O3S C1[X(C37H34N2O3S)] #Atoms= 77
Charge = 0 Multiplicity = 1

SCF Energy= -2163.11220778

===

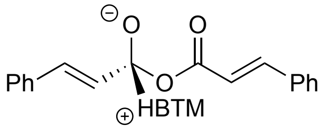

III

Supporting Information: 0010-HYPERBTM-Anhydride-Re-attack-Tet-Int.log

----

Using Gaussian 09: AM64L-G09RevC.01 23-Sep-2011

===

#M062X/6-31G(d) scf=(maxcycle=300,direct,tight) density=current
SCRF=(PCM,SOLVENT=THF) opt=(maxcycle=250) freq=noraman
#N Geom=AllCheck Guess=TCheck SCRF=Check GenChk RM062X/6-31G(d) Freq

----

Pointgroup= C1 Stoichiometry= C37H34N2O3S C1[X(C37H34N2O3S)] #Atoms= 77
Charge = 0 Multiplicity = 1

SCF Energy= -2163.02339797 Predicted Change= -1.762699D-08

===

Optimization completed. {Found 1 times}

Item	Max Val.	Criteria	Pass?	RMS Val.	Criteria	Pass?
Force	0.00001 ||	0.00045	[YES]	0.00000 ||	0.00030	[YES]
Displ	0.00697 ||	0.00180	[NO]	0.00697 ||	0.00180	[YES]

Atomic Coordinates (Angstroms)

Type	X	Y	Z
C	1.075572	6.307405	-0.122621
H	1.173931	7.241231	0.420639
C	0.535520	5.199558	0.525137
H	0.207673	5.262790	1.556992
C	0.423977	4.016382	-0.198707
C	0.826990	3.939276	-1.531836
C	1.361723	5.049022	-2.174828
H	1.673962	4.990104	-3.212539
C	1.487519	6.234881	-1.455613
H	1.906488	7.111099	-1.938647
N	-0.069564	2.792766	0.268490
C	-0.078734	1.803394	-0.654405
S	0.505550	2.346758	-2.222020
C	-0.466435	2.581483	1.661458
H	-1.458987	3.022430	1.822752
H	0.254578	3.117614	2.282664
C	-0.454736	1.094375	1.997583
C	-1.046847	0.824062	3.391545
C	-0.863351	-0.643489	3.787041
H	0.198981	-0.913805	3.768783
H	-1.393388	-1.329300	3.121346
H	-1.240481	-0.811990	4.800111
H	-2.122060	1.048642	3.354039
C	-0.405714	1.721849	4.454888
H	-0.635174	2.780766	4.303409
H	0.684750	1.603094	4.455324
H	-0.772251	1.447551	5.448420
H	0.590358	0.755077	1.996093
C	-1.145478	0.289276	0.878428
C	-2.649494	0.482708	0.769886
H	-0.938932	-0.761745	1.070790

N	-0.481008	0.586106	-0.399363
C	-0.358335	-0.485164	-1.571829
O	1.149741	-0.833122	-1.507000
C	1.758924	-1.236448	-0.405229
C	3.201874	-1.495822	-0.650005
H	3.562679	-1.317367	-1.657548
C	3.980572	-1.935142	0.346140
C	5.414224	-2.243406	0.279179
H	3.502982	-2.086554	1.313708
O	1.226853	-1.385040	0.686585
C	-1.120391	-1.725468	-1.146040
H	-0.656719	-2.396489	-0.428271
C	-2.347713	-1.929388	-1.626527
C	-3.236243	-3.048017	-1.272427
H	-2.753039	-1.179174	-2.304742
O	-0.603372	0.037447	-2.683140
C	-3.218664	1.625831	0.199986
H	-2.589321	2.411528	-0.210787
C	-4.602467	1.764787	0.119365
H	-5.026046	2.657807	-0.329643
C	-5.438277	0.759657	0.600010
H	-6.516327	0.866833	0.530065
C	-4.881562	-0.390131	1.154962
H	-5.521476	-1.192881	1.508499
C	-3.498429	-0.525566	1.234477
H	-3.072953	-1.443408	1.632955
C	6.179929	-2.044299	-0.879241
H	5.716469	-1.641589	-1.774727
C	7.533622	-2.353517	-0.892262
H	8.112950	-2.192209	-1.796096
C	8.149143	-2.867634	0.250289
H	9.207552	-3.108533	0.236418
C	7.401131	-3.068695	1.407033

H	7.871890	-3.468008	2.299929
C	6.044805	-2.757119	1.419518
H	5.460512	-2.914309	2.322635
C	-2.755288	-4.250206	-0.735468
H	-1.685513	-4.392576	-0.610588
C	-3.632395	-5.269573	-0.383277
H	-3.243073	-6.197261	0.025194
C	-5.006517	-5.108654	-0.563278
H	-5.689079	-5.907517	-0.290093
C	-5.496397	-3.921706	-1.103400
H	-6.564057	-3.789004	-1.250686
C	-4.616790	-2.903481	-1.459353
H	-4.999054	-1.973491	-1.872997

Statistical Thermodynamic Analysis

Temperature= 298.150 Kelvin Pressure= 1.00000 Atm

==

SCF Energy= -2163.02339797 Predicted Change= -1.762699D-08

Zero-point correction (ZPE)= -2162.3902 0.63310

Internal Energy (U)= -2162.3528 0.67057

Enthalpy (H)= -2162.3518 0.67151

Gibbs Free Energy (G)= -2162.4639 0.55947

Frequencies -- 10.1889 18.2994 20.9709

==

#m062X/6-31+G(d,p) scf=(maxcycle=300,direct,tight) density=current

SCRF=(PCM,SOLVENT=THF)

Pointgroup= C1 Stoichiometry= C37H34N2O3S C1[X(C37H34N2O3S)] #Atoms= 77

Charge = 0 Multiplicity = 1

SCF Energy= -2163.11451983

==

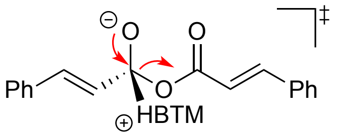

TS-IV

Supporting Information: 0015-Dissociation-from-Re-Te-Int.log

Using Gaussian 09: AM64L-G09RevC.01 23-Sep-2011

==

#m062X/6-31G(d) scf=(maxcycle=300,direct,tight) density=current

SCRF=(PCM,SOLVENT=THF) opt=(maxcycle=250,ts,calcfc,noeigentest)

freq=noraman

#N Geom=AllCheck Guess=TCheck SCRF=Check GenChk RM062X/6-31G(d) Freq

----

Pointgroup= C1 Stoichiometry= C37H34N2O3S C1[X(C37H34N2O3S)] #Atoms= 77

Charge = 0 Multiplicity = 1

SCF Energy= -2163.02090696 Predicted Change= -1.891779D-09

==

Optimization completed on the basis of negligible forces. {Found 2 times}

Item	Max Val.		Criteria	Pass?	RMS Val.		Criteria	Pass?
Force	0.00000	||	0.00045	[YES]	0.00000	||	0.00030	[YES]
Displ	0.00301	||	0.00180	[NO]	0.00301	||	0.00180	[YES]

Atomic Type	Coordinates (Angstroms) X	Y	Z
C	1.513782	6.226192	-0.040213
H	1.749577	7.109817	0.542864
C	0.943754	5.127684	0.594884
H	0.726446	5.148593	1.657042
C	0.656690	4.009417	-0.183010
C	0.919771	3.988676	-1.551754

C	1.486513	5.090388	-2.183320
H	1.689984	5.075009	-3.248916
C	1.784831	6.209349	-1.411852
H	2.230512	7.078686	-1.882991
N	0.095756	2.804113	0.258917
C	-0.094607	1.893052	-0.714313
S	0.397256	2.476672	-2.287946
C	-0.189673	2.541577	1.672091
H	-1.122009	3.050130	1.949156
H	0.628846	2.981867	2.244779
C	-0.266062	1.041665	1.923502
C	-0.735515	0.728416	3.354786
C	-0.643743	-0.773660	3.636177
H	0.370762	-1.136884	3.436517
H	-1.333057	-1.357366	3.020711
H	-0.884269	-0.978473	4.683560
H	-1.784714	1.043497	3.446279
C	0.091279	1.497249	4.390226
H	-0.195862	1.193047	5.400880
H	-0.053124	2.579727	4.321819
H	1.160101	1.284588	4.266199
H	0.740085	0.621056	1.791002
C	-1.126506	0.350229	0.852304
C	-2.620918	0.612391	0.913001
H	-0.936368	-0.715678	0.960305
N	-0.600657	0.696014	-0.484546
C	-0.736878	-0.256356	-1.625415
O	1.087829	-0.841985	-1.623951
C	1.660491	-1.296450	-0.568803
C	3.117081	-1.611898	-0.760949
H	3.522299	-1.414234	-1.749352
C	3.851195	-2.108212	0.240394
C	5.278490	-2.459829	0.211242

H	3.337224	-2.271351	1.187228
O	1.123086	-1.482611	0.536178
C	-1.395746	-1.541017	-1.232064
H	-0.869246	-2.191499	-0.541257
C	-2.626572	-1.792359	-1.685079
C	-3.446821	-2.956425	-1.316110
H	-3.098041	-1.052265	-2.330907
O	-0.877198	0.251416	-2.730945
C	-3.474617	-0.419654	1.311643
H	-3.058780	-1.397022	1.545590
C	-4.852110	-0.226133	1.367462
H	-5.498048	-1.045594	1.667292
C	-5.396055	1.007327	1.017403
H	-6.469953	1.161346	1.054099
C	-4.556298	2.037966	0.601060
H	-4.973500	2.996135	0.307116
C	-3.178766	1.840479	0.544464
H	-2.546883	2.647326	0.180711
C	6.085933	-2.277173	-0.921400
H	5.659476	-1.861129	-1.829202
C	7.431441	-2.621062	-0.895174
H	8.041423	-2.471714	-1.781054
C	8.000396	-3.155012	0.262177
H	9.052504	-3.422269	0.278917
C	7.211178	-3.342256	1.393581
H	7.643520	-3.756989	2.299019
C	5.863212	-2.996484	1.365536
H	5.247778	-3.142782	2.249857
C	-4.840408	-2.862255	-1.417736
H	-5.282604	-1.941549	-1.790184
C	-5.655885	-3.922529	-1.033015
H	-6.734817	-3.831189	-1.113707
C	-5.086912	-5.098591	-0.549643

H	-5.719423	-5.930035	-0.254489
C	-3.699234	-5.207869	-0.454899
H	-3.250915	-6.126732	-0.089450
C	-2.885269	-4.147097	-0.835046
H	-1.805110	-4.246682	-0.775960

Statistical Thermodynamic Analysis

Temperature= 298.150 Kelvin Pressure= 1.00000 Atm

==

SCF Energy= -2163.02090696 Predicted Change= -1.891779D-09

Zero-point correction (ZPE)= -2162.3885 0.63233

Internal Energy (U)= -2162.3511 0.66972

Enthalpy (H)= -2162.3502 0.67066

Gibbs Free Energy (G)= -2162.4634 0.55749

Frequencies -- -114.8179 7.5217 16.2836

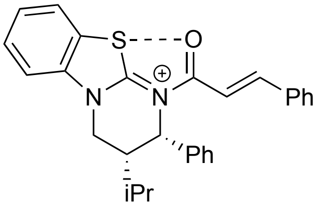

α,β−unsaturated acyl ammonium

Supporting Information: 0020-Alpha-beta-unsaturated-HYPERBTM-1.log

----

Using Gaussian 09: AM64L-G09RevC.01 23-Sep-2011

==

#M062X/6-31G(d) scf=(maxcycle=300,direct,tight) density=current

SCRF=(PCM,SOLVENT=THF) opt=(maxcycle=250,gdiis) iop(1/8=18) freq=noraman

#N Geom=AllCheck Guess=TCheck SCRF=Check Test GenChk RM062X/6-31G(d) Freq

----

Pointgroup= C1 Stoichiometry= C28H27N2OS(1+) C1[X(C28H27N2OS)] #Atoms= 59

Charge = 1 Multiplicity = 1

SCF Energy= -1665.46191819 Predicted Change= -5.735051D-09

==

Optimization completed. {Found 1 times}

Item	Max Val.	Criteria	Pass?	RMS Val.	Criteria	Pass?
Force	0.00001 ||	0.00045	[YES]	0.00000 ||	0.00030	[YES]
Displ	0.00214 ||	0.00180	[NO]	0.00214 ||	0.00180	[YES]

Atomic Type	Coordinates (Angstroms) X	Y	Z
C	-6.166109	-0.920994	0.419624
H	-7.144371	-0.465355	0.525261
C	-5.070035	-0.112771	0.151680
H	-5.179375	0.961613	0.057122
C	-3.829886	-0.737145	0.028525
C	-3.688202	-2.114071	0.177532
C	-4.791205	-2.922617	0.442857
H	-4.681852	-3.995769	0.556081
C	-6.030902	-2.308467	0.561386
H	-6.906261	-2.912776	0.772243
N	-2.601382	-0.116358	-0.238168
C	-1.561635	-0.952255	-0.262441
S	-2.018554	-2.610282	-0.021174
C	-2.500269	1.321300	-0.523463
H	-2.528658	1.875394	0.422294
H	-3.378081	1.580696	-1.115867
C	-1.226226	1.588092	-1.307664
C	-1.025966	3.087930	-1.601625
C	0.121228	3.301208	-2.593738
H	-0.114190	2.826914	-3.553453
H	1.076853	2.896761	-2.247672
H	0.265875	4.369867	-2.773953

H	-0.784306	3.594532	-0.658580
C	-2.304078	3.717086	-2.166861
H	-3.115033	3.749773	-1.433679
H	-2.653550	3.165526	-3.047774
H	-2.102961	4.746602	-2.475199
H	-1.312806	1.065480	-2.271322
C	-0.035247	0.949650	-0.563884
C	0.295301	1.587081	0.774498
H	0.828833	1.020042	-1.226876
N	-0.291660	-0.507653	-0.414108
C	0.785043	-1.443088	-0.360148
C	2.140427	-0.895382	-0.244394
H	2.280088	0.147134	0.008579
C	3.181997	-1.732271	-0.405246
C	4.597643	-1.392284	-0.284009
H	2.957882	-2.768688	-0.654102
O	0.525256	-2.630704	-0.391770
C	-0.314877	1.172612	1.960969
H	-1.021123	0.345696	1.964825
C	-0.008382	1.798964	3.166858
H	-0.488681	1.464737	4.080752
C	0.915280	2.839826	3.201363
H	1.155712	3.323525	4.142482
C	1.537819	3.250035	2.024725
H	2.267649	4.053012	2.043447
C	1.231437	2.624849	0.819677
H	1.728588	2.946307	-0.092554
C	5.042639	-0.123629	0.121940
H	4.327635	0.655999	0.368650
C	6.400385	0.142689	0.222486
H	6.735302	1.124561	0.540667
C	7.335136	-0.849816	-0.080303
H	8.396396	-0.636778	0.000365

C	6.906404	-2.112485	-0.481410
H	7.630034	-2.886919	-0.713882
C	5.545627	-2.381363	-0.580248
H	5.206427	-3.365765	-0.891097

Statistical Thermodynamic Analysis

Temperature= 298.150 Kelvin Pressure= 1.00000 Atm

==

SCF Energy= -1665.46191819 Predicted Change= -5.735051D-09

Zero-point correction (ZPE)= -1664.9675 0.49436

Internal Energy (U)= -1664.9404 0.52149

Enthalpy (H)= -1664.9394 0.52244

Gibbs Free Energy (G)= -1665.0263 0.43556

Frequencies -- 16.3996 27.3485 33.0210

==

#M062X/6-31+G(d,p) scf=(maxcycle=300,direct,tight) density=current SCRF=(PCM,SOLVENT=THF)

Pointgroup= C1 Stoichiometry= C28H27N2OS(1+) C1[X(C28H27N2OS)] #Atoms= 59

Charge = 1 Multiplicity = 1

SCF Energy= -1665.52071455

==

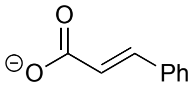

Supporting Information: 0000-Cinnamyl-acetate.log

Using Gaussian 09: AM64L-G09RevC.01 23-Sep-2011

==

#m062X/6-31G(d) scf=(maxcycle=300,direct,tight) density=current SCRF=(PCM,SOLVENT=THF) opt=(maxcycle=250) freq=noraman

#N Geom=AllCheck Guess=TCheck SCRF=Check GenChk RM062X/6-31G(d) Freq

----

Pointgroup= C1 Stoichiometry= C9H7O2(1-) C1[X(C9H7O2)] #Atoms= 18

Charge = -1 Multiplicity = 1

SCF Energy= -497.527506487 Predicted Change= -4.152790D-11

==

Optimization completed. {Found 2 times}

Item	Max Val.	Criteria	Pass?	RMS Val.	Criteria	Pass?
Force	0.00000 ||	0.00045	[YES]	0.00000 ||	0.00030	[YES]
Displ	0.00024 ||	0.00180	[YES]	0.00024 ||	0.00180	[YES]

Atomic Type	Coordinates (Angstroms) X	Y	Z
C	1.697650	1.258769	0.000101
H	1.296468	2.269386	0.000186
C	0.806587	0.177077	-0.000073
C	-0.641120	0.445848	-0.000127
H	-0.935582	1.495199	-0.000427
C	-1.638108	-0.444147	0.000201
H	-1.450670	-1.517507	0.000585
C	-3.109538	-0.029431	0.000039
O	-3.353799	1.200870	-0.000260
O	-3.925158	-0.982734	0.000214
C	1.345877	-1.118872	-0.000213
H	0.680504	-1.977095	-0.000415
C	2.720868	-1.319957	-0.000133
H	3.115310	-2.332018	-0.000248
C	3.594379	-0.231403	0.000067
H	4.668207	-0.391885	0.000120
C	3.075703	1.060733	0.000177
H	3.743625	1.917120	0.000317

Statistical Thermodynamic Analysis

Temperature= 298.150 Kelvin Pressure= 1.00000 Atm

===

SCF Energy= -497.527506487 Predicted Change= -4.152790D-11

Zero-point correction (ZPE)= -497.3898 0.13767

Internal Energy (U)= -497.3806 0.14687

Enthalpy (H)= -497.3796 0.14781

Gibbs Free Energy (G)= -497.4261 0.10135

Frequencies -- 24.6044 84.1683 108.3655

===

#m062X/6-31+G(d,p) scf=(maxcycle=300,direct,tight) density=current

SCRF=(PCM,SOLVENT=THF)

Pointgroup= C1 Stoichiometry= C9H7O2(1-) C1[X(C9H7O2)] #Atoms= 18

Charge = -1 Multiplicity = 1

SCF Energy= -497.574409223

===

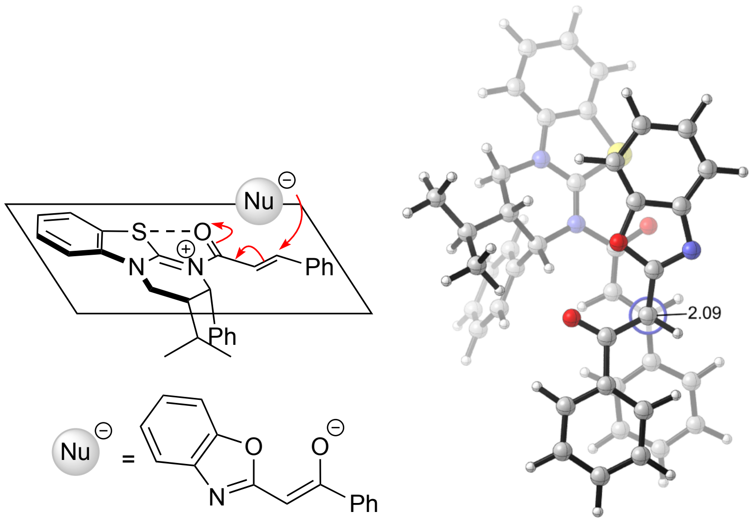

TS-1,4-addition-benzoxazole-Major

Supporting Information: 0025-S-S-Major-oxazole-nuc-attack.log

----

Using Gaussian 09: AM64L-G09RevC.01 23-Sep-2011

===

#M062X/6-31G(d) scf=(maxcycle=300,direct,tight) density=current

SCRF=(PCM,SOLVENT=THF) opt=(maxcycle=250,ts,calcfc,noeigentest,gdiis)

iop(1/8=18) freq=noraman

#N Geom=AllCheck Guess=TCheck SCRF=Check Test GenChk RM062X/6-31G(d) Freq

----

Pointgroup= C1 Stoichiometry= C43H37N3O3S C1[X(C43H37N3O3S)] #Atoms= 87

Charge = 0 Multiplicity = 1

SCF Energy= -2448.11606298 Predicted Change= -6.701876D-10

===

Optimization completed. {Found 2 times}

Item	Max Val.	Criteria	Pass?	RMS Val.	Criteria	Pass?
Force	0.00000 ||	0.00045	[YES]	0.00000 ||	0.00030	[YES]
Displ	0.00038 ||	0.00180	[YES]	0.00038 ||	0.00180	[YES]

Atomic Type	Coordinates (Angstroms) X	Y	Z
N	3.506622	0.828395	-0.059146
C	2.579804	0.071366	-0.673227
N	1.277740	0.311591	-0.561127
C	0.315892	-0.585670	-1.217376
C	-1.010604	-0.130033	-1.280732
H	-1.244644	0.890872	-1.022567
C	-2.037729	-1.080896	-1.410284
C	-3.415499	-0.707550	-1.819015
H	-1.723815	-2.066000	-1.753679
O	0.782151	-1.654596	-1.628358
C	0.781481	1.384440	0.333193

H	-0.132313	0.972613	0.770404
C	0.451325	2.638247	-0.453502
C	1.785615	1.608999	1.481399
H	1.828214	0.663447	2.040411
C	1.336906	2.706669	2.464182
C	0.079309	2.271984	3.222213
H	0.308477	1.408378	3.858492
H	-0.745729	1.979968	2.568122
H	-0.265874	3.083126	3.870558
H	1.111366	3.610396	1.883197
C	2.441013	3.050210	3.469184
H	2.783742	2.149863	3.993974
H	2.054630	3.745604	4.219901
H	3.306832	3.525863	2.998802
C	3.172506	1.872737	0.914163
H	3.926595	1.816986	1.701435
H	3.237968	2.856399	0.432732
C	4.828707	0.487251	-0.373708
C	4.888106	-0.597591	-1.246312
S	3.283603	-1.200233	-1.643075
C	5.983108	1.119337	0.081198
H	5.937302	1.977919	0.741875
C	7.205300	0.620139	-0.355873
H	8.120429	1.094793	-0.018499
C	7.272333	-0.475439	-1.222892
H	8.238360	-0.844698	-1.549670
C	6.113515	-1.096517	-1.676687
H	6.159798	-1.945826	-2.350324
C	-4.277225	-1.700094	-2.301036
H	-3.909834	-2.720264	-2.390524
C	-5.587990	-1.398728	-2.655745
H	-6.240490	-2.181835	-3.030302
C	-6.062014	-0.094342	-2.526999

H	-7.087053	0.143542	-2.794843
C	-5.213658	0.902390	-2.049093
H	-5.577766	1.919612	-1.939724
C	-3.900203	0.600346	-1.703522
H	-3.255016	1.387337	-1.322128
C	-0.725305	3.333524	-0.157465
H	-1.403787	2.933470	0.594228
C	-1.041793	4.507510	-0.835782
H	-1.959399	5.036722	-0.598430
C	-0.189537	4.993400	-1.825623
H	-0.437780	5.905335	-2.359261
C	0.973618	4.294965	-2.140144
H	1.633282	4.658006	-2.921990
C	1.290499	3.121617	-1.459475
H	2.187917	2.572186	-1.736258
C	1.466881	-4.816953	0.612501
H	1.151991	-5.771578	0.204081
C	2.782126	-4.588997	1.006061
H	3.514195	-5.383713	0.901791
C	3.187084	-3.356830	1.542066
H	4.220233	-3.219807	1.845295
C	2.288161	-2.301493	1.694105
H	2.594845	-1.352611	2.121376
C	0.989926	-2.544119	1.276606
C	0.557275	-3.767951	0.755375
O	-0.083627	-1.701866	1.287965
C	-1.113913	-2.447591	0.763547
C	-2.392894	-1.834570	0.508477
H	-3.144170	-2.584160	0.291724
C	-2.807554	-0.610070	1.156470
C	-4.278666	-0.314816	1.289922
O	-1.995820	0.258757	1.503625
N	-0.789978	-3.673559	0.451312

C	-5.289229	-1.233861	0.990801
H	-5.048936	-2.242145	0.672112
C	-6.628275	-0.865978	1.089027
H	-7.402150	-1.587473	0.844599
C	-6.975538	0.419268	1.496934
H	-8.021094	0.703451	1.570676
C	-5.974976	1.337124	1.815799
H	-6.239089	2.339445	2.139845
C	-4.639507	0.970286	1.711923
H	-3.847491	1.674854	1.944719

Statistical Thermodynamic Analysis

Temperature= 298.150 Kelvin Pressure= 1.00000 Atm

==

SCF Energy= -2448.11606298 Predicted Change= -6.701876D-10

Zero-point correction (ZPE)= -2447.4053 0.71071

Internal Energy (U)= -2447.3647 0.75133

Enthalpy (H)= -2447.3637 0.75227

Gibbs Free Energy (G)= -2447.4785 0.63753

Frequencies -- -374.7227 18.2373 26.3049

==

#M062X/6-31+G(d,p) scf=(maxcycle=300,direct,tight) density=current SCRF=(PCM,SOLVENT=THF)

Pointgroup= C1 Stoichiometry= C43H37N3O3S C1[X(C43H37N3O3S)] #Atoms= 87

Charge = 0 Multiplicity = 1

SCF Energy= -2448.21766814

==

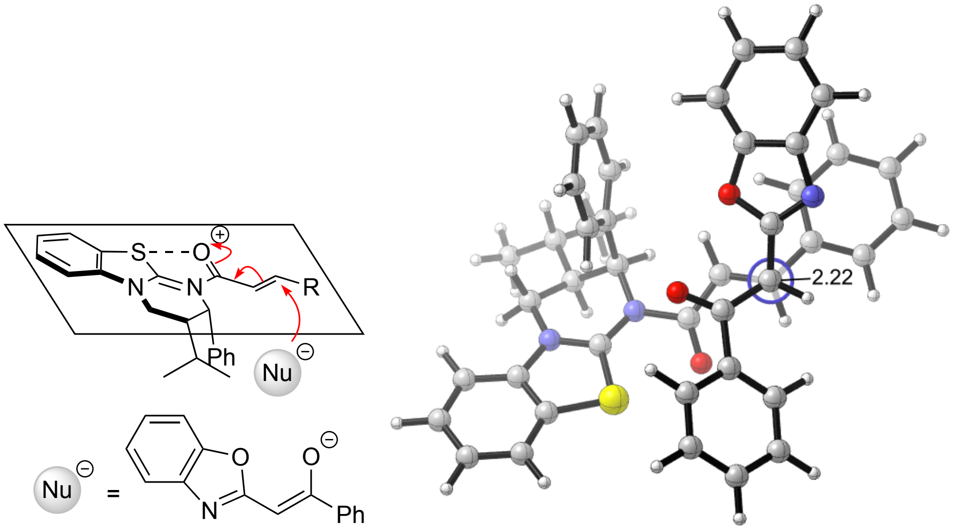

TS-1,4-addition-benzoxazole-*minor*

Supporting Information: 0025-S-R-Minor-oxazole-nuc-attack.log

----

Using Gaussian 09: AM64L-G09RevC.01 23-Sep-2011

==

#M062X/6-31G(d) scf=(maxcycle=300,direct,tight) density=current

SCRF=(PCM,SOLVENT=THF) opt=(maxcycle=250,ts,calcfc,noeigentest)

freq=noraman

#N Geom=AllCheck Guess=TCheck SCRF=Check GenChk RM062X/6-31G(d) Freq

----

Pointgroup= C1 Stoichiometry= C43H37N3O3S C1[X(C43H37N3O3S)] #Atoms= 87

Charge = 0 Multiplicity = 1

SCF Energy= -2448.10290370 Predicted Change= -4.794267D-09

==

Optimization completed. {Found 2 times}

Item	Max Val.	Criteria	Pass?	RMS Val.	Criteria	Pass?
Force	0.00000 ||	0.00045	[YES]	0.00000 ||	0.00030	[YES]
Displ	0.00167 ||	0.00180	[YES]	0.00167 ||	0.00180	[YES]

Atomic Coordinates (Angstroms)

Type X Y Z

N	2.627562	0.807984	-0.385039
C	1.538694	1.206893	0.290287
N	0.822539	0.384908	1.039393
C	-0.442071	0.882507	1.645572
C	-1.562857	0.036955	1.546625
H	-2.422850	0.411485	2.093755
C	-1.759282	-1.002169	0.612922
C	-2.915250	-1.920884	0.790669
H	-0.881923	-1.511384	0.216313
O	-0.374868	1.995350	2.160594
C	1.359437	-0.950704	1.355855
H	0.495197	-1.555814	1.624380
C	2.273762	-0.921821	2.570843
C	2.003609	-1.517823	0.072323
H	1.224517	-1.487766	-0.701323
C	2.478847	-2.973814	0.213298
C	1.268844	-3.907436	0.314592
H	1.592533	-4.945001	0.439149
H	0.672420	-3.846421	-0.606900
H	0.615209	-3.664236	1.159293
H	3.075806	-3.056363	1.131305
C	3.355130	-3.406868	-0.965735
H	2.825251	-3.258398	-1.914226
H	3.595677	-4.470569	-0.878947
H	4.301488	-2.858919	-1.005570
C	3.108855	-0.576891	-0.389714
H	3.395790	-0.810128	-1.417276
H	3.998687	-0.641923	0.249690
C	3.217484	1.811940	-1.162790
C	2.534435	3.023224	-1.052556
S	1.199807	2.902941	0.082324
C	4.345602	1.688097	-1.968200

H	4.886231	0.751110	-2.042758
C	4.763623	2.812608	-2.672451
H	5.638820	2.742502	-3.309308
C	4.077383	4.026422	-2.571286
H	4.421834	4.888492	-3.132147
C	2.956620	4.145714	-1.755696
H	2.424305	5.087083	-1.670549
C	-2.744410	-3.295063	0.585504
H	-1.769966	-3.671501	0.281698
C	-3.804288	-4.180501	0.766143
H	-3.650795	-5.243868	0.608683
C	-5.054442	-3.701524	1.148556
H	-5.882864	-4.389129	1.288022
C	-5.236365	-2.332931	1.348768
H	-6.210387	-1.952032	1.640575
C	-4.177351	-1.448851	1.174506
H	-4.333746	-0.382618	1.305653
C	2.223886	-2.000256	3.458846
H	1.511183	-2.803561	3.286403
C	3.066100	-2.053092	4.565599
H	3.010110	-2.897437	5.245342
C	3.966178	-1.017663	4.804815
H	4.619801	-1.051154	5.670600
C	4.013364	0.068290	3.934585
H	4.702202	0.886187	4.120757
C	3.171490	0.118309	2.825821
H	3.214422	0.988742	2.176714
C	-3.369143	5.139802	0.846858
H	-3.136524	6.119057	1.252797
C	-4.710287	4.733418	0.758148
H	-5.488681	5.411177	1.094791
C	-5.063792	3.484959	0.255588
H	-6.099755	3.168748	0.191038

C	-4.031046	2.643256	-0.161996
C	-2.704844	3.076386	-0.069416
C	-2.328408	4.311214	0.430164
H	-1.288172	4.605996	0.503341
N	-4.045076	1.358330	-0.690943
C	-2.785657	1.076217	-0.894064
C	-2.259412	-0.202818	-1.287659
H	-3.047986	-0.869512	-1.613317
C	-0.965993	-0.344306	-1.924943
C	-0.661062	-1.661720	-2.600026
O	-0.066014	0.499795	-1.865123
O	-1.915708	2.072999	-0.548246
C	0.555011	-1.764127	-3.286903
H	1.198577	-0.889883	-3.321125
C	0.925375	-2.953488	-3.903517
H	1.867229	-3.015258	-4.441061
C	0.090306	-4.068518	-3.826329
H	0.380970	-5.000940	-4.300612
C	-1.120484	-3.978908	-3.143714
H	-1.776452	-4.841833	-3.081320
C	-1.499373	-2.780366	-2.541291
H	-2.446815	-2.737711	-2.014332

Statistical Thermodynamic Analysis

Temperature= 298.150 Kelvin Pressure= 1.00000 Atm

==

SCF Energy= -2448.10290370 Predicted Change= -4.794267D-09

Zero-point correction (ZPE)= -2447.3929 0.70995

Internal Energy (U)= -2447.3519 0.75093

Enthalpy (H)= -2447.3510 0.75188

Gibbs Free Energy (G)= -2447.4673 0.63556

Frequencies -- -372.2761 20.3856 26.1715

==

#M062X/6-31+G(d,p) scf=(maxcycle=300,direct,tight) density=current SCRF=(PCM,SOLVENT=THF)

Pointgroup= C1   Stoichiometry= C43H37N3O3S   C1[X(C43H37N3O3S)] #Atoms= 87

Charge = 0   Multiplicity = 1

SCF Energy= -2448.20539636

==

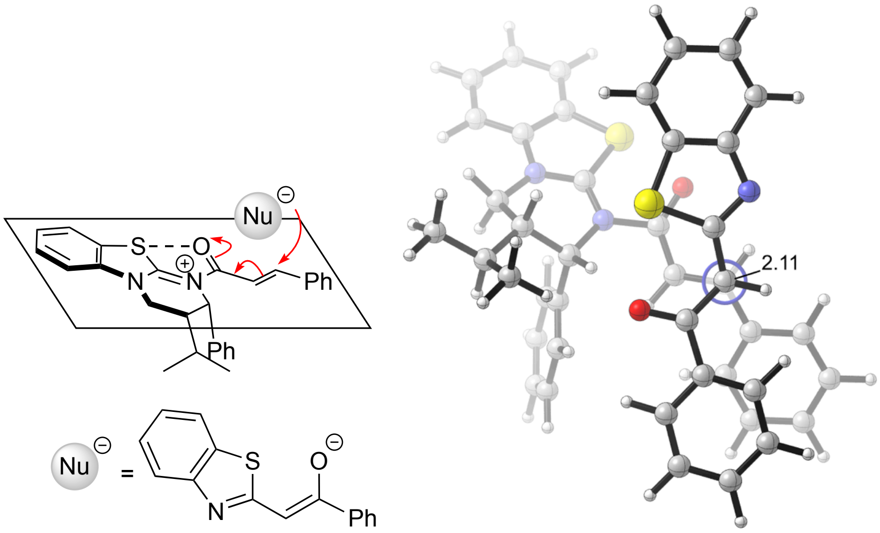

TS-1,4-addition-benzothiazole-*Major*

Supporting Information: 0025-S-S-Major-thiazole-nuc-attack.log

----

Using Gaussian 09: AM64L-G09RevC.01 23-Sep-2011

==

#m062X/6-31G(d) scf=(maxcycle=300,direct,tight) density=current SCRF=(PCM,SOLVENT=THF) opt=(maxcycle=250,ts,calcfc,noeigentest) freq=noraman

#N Geom=AllCheck Guess=TCheck SCRF=Check GenChk RM062X/6-31G(d) Freq

----

Pointgroup= C1   Stoichiometry= C43H37N3O2S2   C1[X(C43H37N3O2S2)] #Atoms= 87

Charge = 0   Multiplicity = 1

SCF Energy= -2771.08369558 Predicted Change= -9.067552D-09

==

Optimization completed. {Found 1 times}

Item	Max Val.	Criteria	Pass?	RMS Val.	Criteria	Pass?
Force	0.00000 ||	0.00045	[YES]	0.00000 ||	0.00030	[YES]
Displ	0.00605 ||	0.00180	[NO]	0.00605 ||	0.00180	[YES]

Atomic Type	Coordinates (Angstroms) X	Y	Z
N	-3.623812	-1.025350	-0.147010
C	-2.683225	-0.283839	-0.758586
N	-1.392598	-0.397517	-0.476126
C	-0.413827	0.479223	-1.150348
C	0.907282	0.005426	-1.142508
C	1.961590	0.885474	-1.433767
C	3.267934	0.413275	-1.958052
O	-0.878440	1.498775	-1.668218
C	-0.947175	-1.298327	0.611565
C	-0.494883	-2.644918	0.079697
C	-2.054176	-1.374293	1.681641
C	-1.651037	-2.225794	2.898444
C	-0.511763	-1.551714	3.670038
C	-2.834916	-2.460849	3.841617
C	-3.348533	-1.848358	1.035274
C	-4.911332	-0.871378	-0.676435
C	-4.938801	0.061302	-1.711516
S	-3.349629	0.770748	-1.983885
C	-6.060533	-1.544803	-0.272167
C	-7.247187	-1.247725	-0.933674
C	-7.283444	-0.306702	-1.967504
C	-6.129087	0.359986	-2.366102

C	4.156912	1.344001	-2.509910
C	5.399776	0.948410	-2.992472
C	5.777243	-0.391753	-2.926917
C	4.899485	-1.328631	-2.384830
C	3.653162	-0.931705	-1.910647
C	0.670439	-3.206144	0.611153
C	1.123082	-4.446563	0.170692
C	0.417685	-5.135854	-0.814221
C	-0.734417	-4.575182	-1.360191
C	-1.187619	-3.333648	-0.917954
H	1.114342	-0.995355	-0.793635
H	1.660653	1.864773	-1.806002
H	-0.082767	-0.793691	1.050460
H	-2.218276	-0.343344	2.031934
H	-0.861790	-0.602811	4.095120
H	0.363463	-1.329909	3.052482
H	-0.190274	-2.189732	4.498921
H	-1.307898	-3.202533	2.532767
H	-3.281766	-1.508112	4.151479
H	-2.493993	-2.977094	4.743773
H	-3.617444	-3.076838	3.388076
H	-4.192713	-1.710875	1.713651
H	-3.298192	-2.904517	0.742969
H	-6.032505	-2.283186	0.521674
H	-8.157106	-1.760748	-0.641552
H	-8.221769	-0.093963	-2.468102
H	-6.153756	1.090278	-3.168066
H	3.865216	2.391545	-2.552085
H	6.074084	1.685224	-3.418672
H	6.748794	-0.705037	-3.296983
H	5.186347	-2.374681	-2.330136
H	2.979779	-1.675745	-1.493884
H	1.232672	-2.644612	1.354902

H	2.032910	-4.867679	0.587423
H	0.772156	-6.099920	-1.164872
H	-1.278107	-5.099045	-2.140024
H	-2.072648	-2.896464	-1.375498
C	-0.486339	5.511222	0.145877
C	-1.748443	5.899851	0.564814
C	-2.530414	5.060015	1.375193
C	-2.056381	3.818057	1.776239
C	-0.785757	3.424572	1.352474
C	0.007686	4.259140	0.538808
S	0.076946	1.947099	1.679582
C	1.420031	2.557023	0.686503
C	2.583076	1.756474	0.384464
C	2.863577	0.548034	1.115433
C	4.246196	-0.033577	1.078876
O	1.956066	-0.091448	1.677524
N	1.238306	3.747353	0.184993
C	5.374330	0.690295	0.679873
C	6.621385	0.075445	0.623784
C	6.757087	-1.268028	0.966396
C	5.640382	-1.993312	1.380598
C	4.396760	-1.377114	1.439755
H	0.126379	6.150429	-0.481936
H	-2.140610	6.865824	0.261781
H	-3.516615	5.384545	1.691864
H	-2.660675	3.169049	2.403148
H	3.407605	2.346500	0.001070
H	5.291046	1.737433	0.409126
H	7.488447	0.647293	0.307393
H	7.730500	-1.746820	0.915046
H	5.741356	-3.039544	1.653711
H	3.516914	-1.928269	1.755641

Statistical Thermodynamic Analysis

Temperature= 298.150 Kelvin Pressure= 1.00000 Atm

==

SCF Energy= -2771.08369558 Predicted Change= -9.067552D-09

Zero-point correction (ZPE)= -2770.3771 0.70657

Internal Energy (U)= -2770.3354 0.74819

Enthalpy (H)= -2770.3345 0.74914

Gibbs Free Energy (G)= -2770.4539 0.62977

Frequencies -- -359.2090 7.8688 18.5685

==

#m062X/6-31+G(d,p) scf=(maxcycle=300,direct,tight) density=current

SCRF=(PCM,SOLVENT=THF)

Pointgroup= C1 Stoichiometry= C43H37N3O2S2 C1[X(C43H37N3O2S2)] #Atoms= 87

Charge = 0 Multiplicity = 1

SCF Energy= -2771.18475399

==

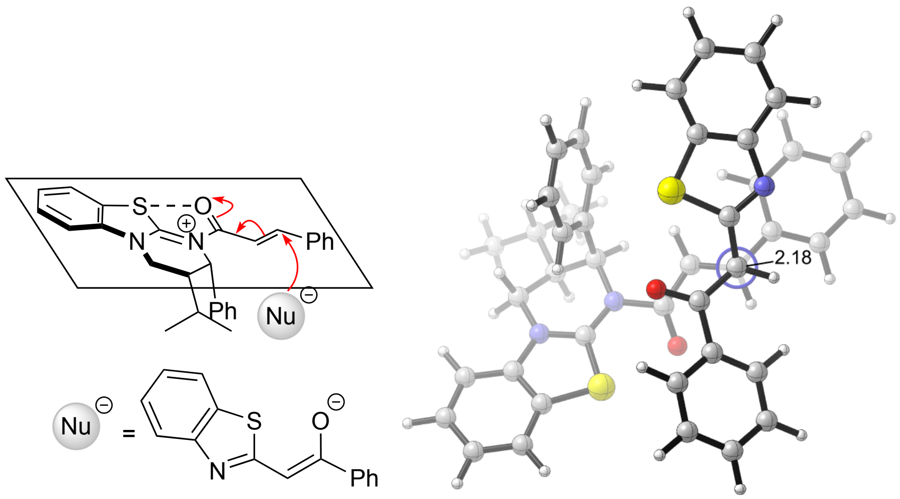

TS-1,4-addition-benzothiazole-*minor*

Supporting Information: 0025-S-R-Minor-thiazole-nuc-attack.log

----

Using Gaussian 09: AM64L-G09RevC.01 23-Sep-2011

==

#m062X/6-31G(d) scf=(maxcycle=300,direct,tight) density=current

SCRF=(PCM,SOLVENT=THF) opt=(maxcycle=250,ts,calcfc,noeigentest)

freq=noraman

#N Geom=AllCheck Guess=TCheck SCRF=Check GenChk RM062X/6-31G(d) Freq

----

Pointgroup= C1 Stoichiometry= C43H37N3O2S2 C1[X(C43H37N3O2S2)] #Atoms= 87

Charge = 0 Multiplicity = 1

SCF Energy= -2771.08208225 Predicted Change= -1.100400D-09

==

Optimization completed. {Found 2 times}

Item	Max Val.	Criteria	Pass?	RMS Val.	Criteria	Pass?
Force	0.00000 ||	0.00045	[YES]	0.00000 ||	0.00030	[YES]
Displ	0.00042 ||	0.00180	[YES]	0.00042 ||	0.00180	[YES]

Atomic Type	Coordinates (Angstroms) X	Y	Z
N	-3.782828	-0.872142	0.240065
C	-2.823854	-0.001742	0.598856
N	-1.636491	-0.399469	1.050049
C	-0.635370	0.610551	1.389383
C	0.703655	0.186380	1.476678
C	1.669276	1.185205	1.650855
C	3.063537	0.887644	2.040168
O	-1.067240	1.753682	1.565587
C	-1.323566	-1.840707	1.113297
C	-0.667619	-2.336397	-0.165000
C	-2.609978	-2.604309	1.498439
C	-2.389849	-4.114578	1.700782
C	-1.472695	-4.379715	2.898041

C	-3.719246	-4.847095	1.913989
C	-3.686830	-2.317522	0.461523
C	-4.913175	-0.276579	-0.332945
C	-4.800919	1.111591	-0.369299
S	-3.280306	1.660358	0.328014
C	-6.036558	-0.932009	-0.830081
C	-7.056120	-0.147796	-1.357802
C	-6.955478	1.247403	-1.388047
C	-5.826947	1.891947	-0.892790
C	3.834362	1.895792	2.630286
C	5.156029	1.665044	2.995902
C	5.734441	0.417605	2.766603
C	4.979361	-0.593292	2.174422
C	3.655513	-0.361975	1.816516
C	0.342383	-3.298055	-0.080061
C	0.921119	-3.824783	-1.231779
C	0.493059	-3.394111	-2.485984
C	-0.496044	-2.418018	-2.578096
C	-1.066476	-1.884802	-1.425552
H	0.987843	-0.826384	1.230575
H	1.289478	2.137040	2.017153
H	-0.619657	-1.939408	1.941541
H	-2.948189	-2.182527	2.456039
H	-1.323304	-5.455646	3.026107
H	-1.926290	-3.991257	3.817361
H	-0.484834	-3.922881	2.794853
H	-1.918947	-4.516146	0.794161
H	-4.282554	-4.398144	2.741145
H	-3.529787	-5.893805	2.167948
H	-4.351295	-4.837872	1.021293
H	-4.664414	-2.645066	0.820031
H	-3.474454	-2.813775	-0.493676
H	-6.112789	-2.013696	-0.816963

H	-7.942543	-0.630298	-1.755159
H	-7.765712	1.835303	-1.805664
H	-5.743516	2.973521	-0.916663
H	3.387226	2.873190	2.798780
H	5.735712	2.459355	3.456547
H	6.768139	0.235285	3.044478
H	5.426140	-1.564154	1.981215
H	3.087086	-1.154979	1.336123
H	0.686618	-3.635235	0.894989
H	1.706798	-4.569372	-1.145832
H	0.937258	-3.806385	-3.387086
H	-0.816062	-2.056063	-3.549967
H	-1.801892	-1.091041	-1.522615
C	5.556890	-2.544168	-2.429015
C	6.498948	-1.592281	-2.007207
C	6.095035	-0.370145	-1.491903
C	4.724587	-0.089323	-1.391174
C	3.786674	-1.057684	-1.808400
C	4.195250	-2.283896	-2.334754
N	4.189579	1.077619	-0.889381
C	2.882448	1.037790	-0.898166
C	2.064964	2.066827	-0.308696
C	0.700890	2.250972	-0.733631
C	0.053908	3.593470	-0.536140
O	0.040564	1.320012	-1.221205
S	2.171632	-0.451006	-1.551906
C	-1.082985	3.892953	-1.292015
C	-1.725973	5.118756	-1.151444
C	-1.243904	6.059939	-0.242488
C	-0.113270	5.767846	0.518453
C	0.535163	4.545827	0.367747
H	5.895328	-3.494445	-2.830374
H	7.558177	-1.818174	-2.085913

H	6.813432	0.372074	-1.157488
H	3.464304	-3.018689	-2.659530
H	2.642909	2.941388	-0.027159
H	-1.450020	3.146472	-1.989222
H	-2.603033	5.343230	-1.751809
H	-1.744668	7.016649	-0.128350
H	0.264845	6.493324	1.232402
H	1.409213	4.332006	0.975967

Statistical Thermodynamic Analysis

Temperature= 298.150 Kelvin   Pressure= 1.00000 Atm

===

SCF Energy=   -2771.08208225   Predicted Change= -1.100400D-09

Zero-point correction (ZPE)=   -2770.3746 0.70745

Internal Energy (U)=   -2770.3334 0.74858

Enthalpy (H)=   -2770.3325   0.74953

Gibbs Free Energy (G)=   -2770.4481   0.63393

Frequencies -- -305.9726   18.7695   25.6698

===

#m062X/6-31+G(d,p) scf=(maxcycle=300,direct,tight) density=current

SCRF=(PCM,SOLVENT=THF)

Pointgroup= C1   Stoichiometry= C43H37N3O2S2   C1[X(C43H37N3O2S2)] #Atoms= 87

Charge = 0   Multiplicity = 1

SCF Energy= -2771.18271562

===

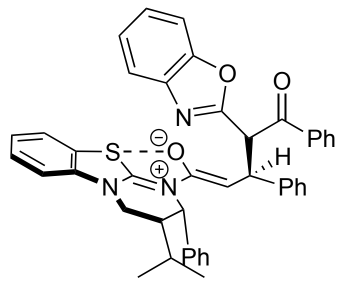

V

Supporting Information: 0030-Major-R-R-INT-after-oxazole-nuc-attack.log

----

Using Gaussian 09: AM64L-G09RevD.01 24-Apr-2013

==

#m062X/6-31G(d) scf=(maxcycle=300,direct,tight) density=current

SCRF=(PCM,SOLVENT=THF) opt=(maxcycle=250) freq=noraman

#N Geom=AllCheck Guess=TCheck SCRF=Check GenChk RM062X/6-31G(d) Freq

----

Pointgroup= C1 Stoichiometry= C43H37N3O3S C1[X(C43H37N3O3S)] #Atoms= 87

Charge = 0 Multiplicity = 1

SCF Energy= -2448.12857881 Predicted Change= -1.498141D-08

==

Optimization completed. {Found 1 times}

Item	Max Val.	Criteria	Pass?	RMS Val.	Criteria	Pass?
Force	0.00001 ||	0.00045	[YES]	0.00000 ||	0.00030	[YES]
Displ	0.00282 ||	0.00180	[NO]	0.00282 ||	0.00180	[YES]

Atomic Type	Coordinates (Angstroms) X	Y	Z
C	-7.377018	-1.999748	-1.286691
C	-6.927178	-2.804614	-2.337119
C	-5.575692	-2.854300	-2.667847
C	-4.685383	-2.085554	-1.926532
C	-5.143282	-1.278241	-0.887390

C	-6.491202	-1.224099	-0.544860
S	-2.938593	-1.943186	-2.145282
C	-2.874668	-0.847964	-0.776272
N	-4.096793	-0.580149	-0.271917
N	-1.764974	-0.363568	-0.251930
C	-0.447817	-0.717070	-0.871732
C	0.651549	-0.498358	-0.095618
C	2.015675	-0.674408	-0.691499
C	2.731378	-1.969394	-0.336397
C	2.916990	0.533826	-0.255386
C	4.232580	0.539196	-1.040739
C	5.501777	0.085207	-0.390843
O	4.225201	0.892907	-2.204855
C	2.163628	1.805671	-0.457906
O	-0.548073	-1.175672	-2.036646
C	-1.830776	0.529555	0.919304
C	-1.634927	-0.239440	2.214914
C	-3.135561	1.352842	0.861606
C	-3.271136	2.363066	2.014923
C	-2.182500	3.435574	1.924034
C	-4.647031	3.037239	2.007634
C	-4.325893	0.406737	0.783698
C	-0.840128	0.311059	3.224097
C	-0.660870	-0.357002	4.432067
C	-1.271575	-1.591196	4.643498
C	-2.054155	-2.153929	3.638285
C	-2.233075	-1.483848	2.430415
C	3.624160	-2.550835	-1.240361
C	4.359597	-3.681512	-0.891981
C	4.209462	-4.248185	0.371439
C	3.309725	-3.685407	1.276037
C	2.573119	-2.557703	0.921469
O	1.643585	2.043112	-1.691887

C	0.949403	3.208927	-1.551944
C	1.103938	3.630137	-0.229980
N	1.892152	2.693047	0.442807
C	0.212631	3.897566	-2.502334
C	-0.387188	5.073879	-2.060117
C	-0.241056	5.525083	-0.737123
C	0.505402	4.815367	0.197688
C	6.691526	0.275752	-1.103636
C	7.903606	-0.148832	-0.575893
C	7.936148	-0.784437	0.665601
C	6.755572	-0.986551	1.376456
C	5.541964	-0.546572	0.856047
H	-8.433019	-1.978217	-1.039519
H	-7.636774	-3.400289	-2.901249
H	-5.223241	-3.479065	-3.482186
H	-6.841755	-0.608975	0.276741
H	0.577978	-0.170638	0.932527
H	1.923754	-0.620277	-1.782981
H	3.097573	0.471229	0.820446
H	-0.992087	1.217726	0.786057
H	-3.101303	1.920685	-0.079763
H	-1.169636	3.025307	1.971192
H	-2.285913	4.152939	2.743813
H	-2.267257	3.986286	0.979538
H	-3.160427	1.819728	2.962762
H	-4.680477	3.825950	2.764826
H	-5.457504	2.336834	2.229421
H	-4.847485	3.499402	1.033257
H	-5.230609	0.953474	0.511483
H	-4.498906	-0.108492	1.737235
H	-0.339609	1.261702	3.054539
H	-0.035429	0.082789	5.202622
H	-1.128990	-2.116270	5.582652

H	-2.520829	-3.122391	3.788365
H	-2.825099	-1.952345	1.647607
H	3.752752	-2.101699	-2.223376
H	5.050707	-4.117589	-1.607218
H	4.783017	-5.127920	0.647197
H	3.178009	-4.129331	2.258646
H	1.860898	-2.128259	1.622386
H	0.112168	3.538646	-3.520031
H	-0.980215	5.658479	-2.755929
H	-0.724573	6.450454	-0.440306
H	0.617305	5.160538	1.220429
H	6.642711	0.759978	-2.073422
H	8.822833	0.009328	-1.131068
H	8.881657	-1.125418	1.076329
H	6.777038	-1.490442	2.337567
H	4.631487	-0.735853	1.414252

Statistical Thermodynamic Analysis

Temperature= 298.150 Kelvin Pressure= 1.00000 Atm

==

SCF Energy= -2448.12857881 Predicted Change= -1.498141D-08

Zero-point correction (ZPE)= -2447.4161 0.71240

Internal Energy (U)= -2447.3747 0.75379

Enthalpy (H)= -2447.3738 0.75474

Gibbs Free Energy (G)= -2447.4943 0.63423

Frequencies -- 9.2967 15.4895 17.9875

==

#m062X/6-31+G(d,p) scf=(maxcycle=300,direct,tight) density=current SCRF=(PCM,SOLVENT=THF)

Pointgroup= C1 Stoichiometry= C43H37N3O3S C1[X(C43H37N3O3S)] #Atoms= 87

Charge = 0 Multiplicity = 1

SCF Energy= -2448.23149702

==

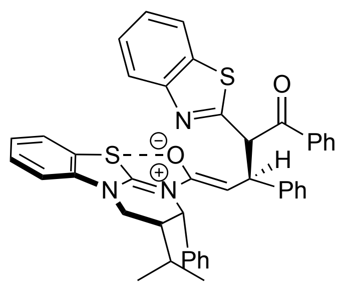

V

Supporting Information: 0030-Major-R-R-int-after-thiazole-nuc-attack.log

----

Using Gaussian 09: AM64L-G09RevC.01 23-Sep-2011

==

#m062X/6-31G(d) scf=(maxcycle=300,direct,tight) density=current

SCRF=(PCM,SOLVENT=THF) opt=(maxcycle=250) freq=noraman

#N Geom=AllCheck Guess=TCheck SCRF=Check GenChk RM062X/6-31G(d) Freq

----

Pointgroup= C1 Stoichiometry= C43H37N3O2S2 C1[X(C43H37N3O2S2)] #Atoms= 87

Charge = 0 Multiplicity = 1

SCF Energy= -2771.09811887 Predicted Change= -2.395198D-08

==

Optimization completed. {Found 1 times}

Item	Max Val.	Criteria	Pass?	RMS Val.	Criteria	Pass?
Force	0.00002 ||	0.00045	[YES]	0.00000 ||	0.00030	[YES]
Displ	0.00275 ||	0.00180	[NO]	0.00275 ||	0.00180	[YES]

Atomic Type	Coordinates (Angstroms) X	Y	Z
C	5.831415	2.180043	-3.183055

H	6.832328	2.594693	-3.237457
C	4.907337	2.467144	-4.191673
H	5.197705	3.098022	-5.024855
C	3.614514	1.954544	-4.137453
H	2.891344	2.177509	-4.915484
C	3.269196	1.146242	-3.059765
C	4.198350	0.855533	-2.063947
C	5.491181	1.371272	-2.102534
H	6.209808	1.164002	-1.317230
S	1.707496	0.389326	-2.741750
C	2.357656	-0.298171	-1.264019
N	3.655704	0.023072	-1.076661
N	1.675450	-1.037836	-0.405750
C	0.207383	-1.219685	-0.597778
C	-0.496586	-1.697512	0.462764
H	-0.016761	-1.954991	1.393934
C	-2.007372	-1.747390	0.460436
H	-2.332965	-1.776074	1.505248
C	-2.586163	-2.978587	-0.225413
C	-2.633107	-0.454117	-0.125115
H	-2.471703	-0.445429	-1.205641
C	-4.132233	-0.371576	0.188672
C	-4.977782	0.608756	-0.569028
O	-4.625309	-1.064059	1.055722
C	-1.995282	0.776642	0.474792
O	-0.199955	-0.831393	-1.734462
C	2.333647	-1.537259	0.815979
H	1.864573	-2.504241	1.006830
C	2.068471	-0.616472	1.997871
C	3.827177	-1.795991	0.525744
H	3.865576	-2.523335	-0.298548
C	4.588630	-2.399036	1.720711
C	4.090942	-3.814193	2.029235

H	4.611526	-4.214636	2.903945
H	4.295339	-4.479622	1.182351
H	3.018073	-3.859874	2.235342
H	4.413313	-1.760100	2.595405
C	6.098290	-2.443890	1.460741
H	6.598621	-2.985072	2.268833
H	6.544746	-1.446478	1.411389
H	6.317387	-2.966850	0.521778
C	4.460315	-0.508012	0.024770
H	5.456105	-0.700000	-0.377728
H	4.537429	0.244987	0.819259
C	1.949916	0.766626	1.846289
H	2.023180	1.222420	0.860915
C	1.691532	1.578759	2.948642
H	1.577213	2.649887	2.810421
C	1.552005	1.018711	4.215676
H	1.335021	1.653459	5.069398
C	1.671058	-0.359538	4.376698
H	1.550919	-0.808790	5.357579
C	1.922342	-1.169797	3.273498
H	1.985824	-2.247615	3.404119
C	-3.417227	-3.857926	0.471413
H	-3.667542	-3.639110	1.506032
C	-3.934366	-4.996177	-0.144998
H	-4.575772	-5.669365	0.416838
C	-3.633971	-5.265087	-1.477796
H	-4.039386	-6.148386	-1.962566
C	-2.808453	-4.390752	-2.184414
H	-2.567779	-4.594844	-3.224186
C	-2.285024	-3.259428	-1.564006
H	-1.626972	-2.583634	-2.105567
S	-0.901908	1.806836	-0.455575
C	-0.803645	2.872808	0.915942

C	-1.565783	2.341411	1.974771
N	-2.241176	1.166229	1.680877
C	-0.089326	4.064109	1.062099
H	0.492190	4.471556	0.241075
C	-0.149699	4.717456	2.286039
H	0.389123	5.650289	2.419970
C	-0.896752	4.188856	3.351941
H	-0.922065	4.717424	4.299581
C	-1.605694	3.006826	3.205640
H	-2.189117	2.587433	4.018964
C	-4.484360	1.384005	-1.622682
H	-3.447892	1.302350	-1.933281
C	-5.322490	2.275218	-2.287735
H	-4.932429	2.873609	-3.104904
C	-6.656203	2.396871	-1.906143
H	-7.308034	3.091746	-2.427024
C	-7.154811	1.625707	-0.855555
H	-8.194131	1.719563	-0.556623
C	-6.319277	0.737368	-0.191915
H	-6.686449	0.128425	0.627713

Statistical Thermodynamic Analysis

Temperature= 298.150 Kelvin Pressure= 1.00000 Atm

==

SCF Energy= -2771.09811887 Predicted Change= -2.395198D-08

Zero-point correction (ZPE)= -2770.3889 0.70917

Internal Energy (U)= -2770.3471 0.75095

Enthalpy (H)= -2770.3462 0.75189

Gibbs Free Energy (G)= -2770.4664 0.63166

Frequencies -- 11.7033 17.9618 22.0346

==

#m062X/6-31+G(d,p) scf=(maxcycle=300,direct,tight) density=current

SCRF=(PCM,SOLVENT=THF)

Pointgroup= C1 Stoichiometry= C43H37N3O2S2 C1[X(C43H37N3O2S2)] #Atoms= 87

Charge = 0 Multiplicity = 1

SCF Energy= -2771.19780624

==

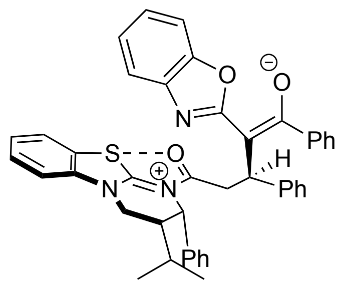

VII

Supporting Information: 0045-oxazole-precyclization-int.log

Using Gaussian 09: AM64L-G09RevD.01 24-Apr-2013

==

#m062X/6-31G(d) scf=(maxcycle=300,direct,tight) density=current

SCRF=(PCM,SOLVENT=THF) opt=(maxcycle=250) freq=noraman

#N Geom=AllCheck Guess=TCheck SCRF=Check GenChk RM062X/6-31G(d) Freq

----

Pointgroup= C1 Stoichiometry= C43H37N3O3S C1[X(C43H37N3O3S)] #Atoms= 87

Charge = 0 Multiplicity = 1

SCF Energy= -2448.14332650 Predicted Change= -6.806262D-09

==

Optimization completed. {Found 2 times}

Item	Max Val.	Criteria	Pass?	RMS Val.	Criteria	Pass?
Force	0.00001 ||	0.00045	[YES]	0.00000 ||	0.00030	[YES]
Displ	0.00122 ||	0.00180	[YES]	0.00122 ||	0.00180	[YES]

Atomic Type	Coordinates (Angstroms) X	Y	Z
N	-2.572354	1.318220	-0.265028
C	-1.662001	0.716796	-1.034196
N	-1.286892	-0.565770	-0.835346
C	-0.210649	-1.089657	-1.617511
C	0.539466	-2.317761	-1.167544
H	0.581861	-2.999390	-2.022205
H	0.098378	-2.833422	-0.320755
C	1.981537	-1.845603	-0.823909
C	1.994547	-0.664676	0.136759
C	3.028598	0.282089	-0.101406
C	1.082885	-0.588613	1.208786
C	0.678430	0.736818	1.821301
O	0.418801	-1.579554	1.626931
H	2.394430	-1.467008	-1.763914
C	2.841458	-3.025385	-0.406027
O	0.137890	-0.468400	-2.597964
C	-2.095329	-1.420601	0.080795
H	-1.398408	-2.150715	0.481824
C	-3.195872	-2.121701	-0.691954
C	-2.531904	-0.584916	1.292594
H	-1.598568	-0.293465	1.784177
C	-3.359598	-1.387945	2.310913
C	-2.553052	-2.575964	2.843167
H	-1.576844	-2.237446	3.206675
H	-2.374148	-3.334983	2.076665
H	-3.090444	-3.060553	3.663703
H	-4.264620	-1.760947	1.809899
C	-3.783642	-0.491642	3.479408
H	-4.478811	0.296463	3.173888
H	-2.905020	-0.017321	3.934527

H	-4.283033	-1.087079	4.248969
C	-3.253271	0.683367	0.869405
H	-3.232491	1.410399	1.684626
H	-4.296285	0.498716	0.582609
C	-2.784278	2.667544	-0.575261
C	-1.990644	3.083469	-1.640756
S	-1.019962	1.765430	-2.265488
C	-3.649770	3.546444	0.074190
H	-4.275022	3.223902	0.898679
C	-3.683298	4.859321	-0.376153
H	-4.345451	5.567279	0.110202
C	-2.880317	5.284946	-1.442793
H	-2.926640	6.317838	-1.770018
C	-2.026001	4.401775	-2.089453
H	-1.403205	4.724503	-2.916718
C	-3.287841	-3.513569	-0.620755
H	-2.555992	-4.071381	-0.042222
C	-4.295786	-4.197317	-1.297250
H	-4.350684	-5.279198	-1.229774
C	-5.219337	-3.494862	-2.065470
H	-6.003736	-4.024470	-2.596574
C	-5.124837	-2.107680	-2.160055
H	-5.831636	-1.552684	-2.768696
C	-4.120709	-1.425913	-1.479183
H	-4.057490	-0.346218	-1.589479
C	3.898596	-3.442780	-1.217264
H	4.102473	-2.906875	-2.141202
C	4.696804	-4.526028	-0.850042
H	5.518326	-4.833603	-1.490666
C	4.441549	-5.208963	0.335639
H	5.059417	-6.054419	0.623436
C	3.390093	-4.796141	1.154661
H	3.190503	-5.320392	2.085141

C	2.598005	-3.711602	0.789855
H	1.787899	-3.368581	1.428291
C	0.502133	1.881655	1.037606
H	0.845995	1.874693	0.006403
C	-0.127941	3.011679	1.554543
H	-0.277898	3.884394	0.923484
C	-0.572806	3.017805	2.876463
H	-1.066788	3.895988	3.281737
C	-0.374951	1.891847	3.676235
H	-0.709486	1.893959	4.709805
C	0.234321	0.756821	3.146640
H	0.341320	-0.144202	3.743959
O	3.314730	1.219840	0.874331
C	4.359409	1.943477	0.379719
C	4.689017	1.401452	-0.868588
N	3.829349	0.350829	-1.145296
C	5.007925	3.017006	0.957323
H	4.716184	3.405067	1.927131
C	6.059342	3.567008	0.217564
H	6.608910	4.410938	0.622217
C	6.413515	3.047235	-1.033510
H	7.234737	3.502079	-1.579378
C	5.739859	1.962154	-1.595891
H	6.017651	1.564872	-2.566918

Statistical Thermodynamic Analysis

Temperature= 298.150 Kelvin Pressure= 1.00000 Atm

===

SCF Energy= -2448.14332650 Predicted Change= -6.806262D-09

Zero-point correction (ZPE)= -2447.4292 0.71404

Internal Energy (U)= -2447.3886 0.75469

Enthalpy (H)= -2447.3876 0.75563

Gibbs Free Energy (G)= -2447.5050 0.63823

Frequencies -- 8.6466 18.4369 20.4383

==

#m062X/6-31+G(d,p) scf=(maxcycle=300,direct,tight) density=current SCRF=(PCM,SOLVENT=THF)

Pointgroup= C1 Stoichiometry= C43H37N3O3S C1[X(C43H37N3O3S)] #Atoms= 87

Charge = 0 Multiplicity = 1

SCF Energy= -2448.24360280

==

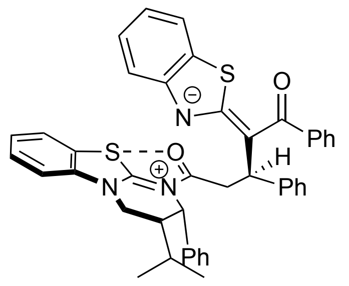

VII

Supporting Information: 0040-thiazole-precyclization-int.log

Using Gaussian 09: AM64L-G09RevC.01 23-Sep-2011

==

#m062X/6-31G(d) scf=(maxcycle=300,direct,tight) density=current

SCRF=(PCM,SOLVENT=THF) opt=(maxcycle=250) freq=noraman

#N Geom=AllCheck Guess=TCheck SCRF=Check GenChk RM062X/6-31G(d) Freq

----

Pointgroup= C1 Stoichiometry= C43H37N3O2S2 C1[X(C43H37N3O2S2)] #Atoms= 87

Charge = 0 Multiplicity = 1

SCF Energy= -2771.10599663 Predicted Change= -1.705358D-08

==

Optimization completed. {Found 2 times}

Item	Max Val.	Criteria	Pass?	RMS Val.	Criteria	Pass?
Force	0.00003 ||	0.00045	[YES]	0.00000 ||	0.00030	[YES]
Displ	0.00159 ||	0.00180	[YES]	0.00159 ||	0.00180	[YES]

Atomic Type	Coordinates (Angstroms) X	Y	Z
N	-2.787630	1.294757	-0.304358
C	-1.834938	0.733401	-1.052642
N	-1.413182	-0.533851	-0.847376
C	-0.304431	-1.016535	-1.609952
C	0.457959	-2.240586	-1.169571
H	0.531590	-2.897419	-2.041667
H	0.002635	-2.788312	-0.351480
C	1.886368	-1.760328	-0.785679
C	1.882489	-0.594305	0.199254
C	2.972445	0.313603	0.037149
C	0.934769	-0.559138	1.243341
C	0.494920	0.738704	1.886341
O	0.268297	-1.565080	1.615015
H	2.314417	-1.362158	-1.709279
C	2.750997	-2.937829	-0.371731
O	0.053056	-0.367851	-2.569452
C	-2.211904	-1.421061	0.046123
H	-1.499790	-2.128934	0.459152
C	-3.270238	-2.153958	-0.755587
C	-2.703073	-0.606673	1.251977
H	-1.792178	-0.277672	1.764137
C	-3.519054	-1.445719	2.250650
C	-2.676586	-2.598739	2.802999
H	-1.730111	-2.218237	3.201250
H	-2.435535	-3.342586	2.038646
H	-3.218614	-3.113571	3.601739

H	-4.394313	-1.856295	1.726738
C	-4.010642	-0.569303	3.407561
H	-4.731175	0.187933	3.083423
H	-3.165128	-0.057668	3.884574
H	-4.503163	-1.186229	4.164521
C	-3.468874	0.629602	0.812615
H	-3.502245	1.356648	1.627206
H	-4.495127	0.398753	0.499171
C	-3.051291	2.633679	-0.620351
C	-2.251482	3.084350	-1.666973
S	-1.207549	1.810939	-2.266569
C	-3.970374	3.473082	0.007515
H	-4.600944	3.122529	0.816324
C	-4.051241	4.783243	-0.444772
H	-4.755494	5.461319	0.024710
C	-3.242558	5.243903	-1.492320
H	-3.326717	6.273686	-1.821737
C	-2.334471	4.399856	-2.117457
H	-1.706619	4.750624	-2.929369
C	-3.324753	-3.547845	-0.683703
H	-2.595941	-4.083712	-0.081175
C	-4.290549	-4.261448	-1.390019
H	-4.315826	-5.344372	-1.322174
C	-5.209262	-3.587164	-2.188586
H	-5.960497	-4.140057	-2.743444
C	-5.152292	-2.197965	-2.282907
H	-5.855473	-1.664633	-2.914696
C	-4.190185	-1.486431	-1.572657
H	-4.155801	-0.405491	-1.683694
C	3.859032	-3.297601	-1.142379
H	4.098567	-2.714053	-2.027725
C	4.662235	-4.377966	-0.777959
H	5.522660	-4.641312	-1.386471

C	4.363046	-5.115270	0.364410
H	4.985107	-5.958610	0.649439
C	3.261951	-4.759248	1.143818
H	3.027071	-5.325694	2.040687
C	2.464194	-3.677908	0.781714
H	1.616445	-3.379500	1.392629
C	0.233460	1.871939	1.109353
H	0.550491	1.879413	0.069214
C	-0.439687	2.966673	1.648490
H	-0.657263	3.831031	1.025912
C	-0.838373	2.946831	2.984571
H	-1.364302	3.796891	3.409081
C	-0.554443	1.833084	3.776890
H	-0.854699	1.818978	4.820668
C	0.094638	0.731535	3.226143
H	0.271089	-0.161864	3.818207
S	3.417893	1.560137	1.267401
C	4.815335	1.982644	0.306551
C	4.847011	1.163428	-0.841939
N	3.825832	0.249342	-0.960565
C	5.781506	2.957337	0.537599
H	5.740673	3.583122	1.423949
C	6.809098	3.106543	-0.391284
H	7.576349	3.856185	-0.225610
C	6.857423	2.297480	-1.533458
H	7.663797	2.428228	-2.248866
C	5.886850	1.331372	-1.765797
H	5.914438	0.701909	-2.649777

Statistical Thermodynamic Analysis

Temperature= 298.150 Kelvin Pressure= 1.00000 Atm

===

SCF Energy= -2771.10599663 Predicted Change= -1.705358D-08

Zero-point correction (ZPE)= -2770.3948 0.71117

Internal Energy (U)= -2770.3535 0.75242

Enthalpy (H)= -2770.3526 0.75336

Gibbs Free Energy (G)= -2770.4709 0.63508

Frequencies -- 12.9939 14.6221 27.0676

==

#m062X/6-31+G(d,p) scf=(maxcycle=300,direct,tight) density=current

SCRF=(PCM,SOLVENT=THF)

Pointgroup= C1 Stoichiometry= C43H37N3O2S2 C1[X(C43H37N3O2S2)] #Atoms= 87

Charge = 0 Multiplicity = 1

SCF Energy= -2771.20441792

==

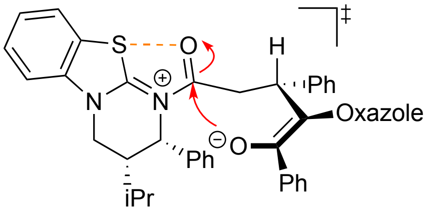

TS-Lactonization-(X=O0-Major

Supporting Information: 0045-oxazole-Si-lactonization.log

Using Gaussian 09: AM64L-G09RevC.01 23-Sep-2011

==

#M062X/6-31G(d) scf=(maxcycle=300,direct,tight) density=current

SCRF=(PCM,SOLVENT=THF) opt=(maxcycle=250,ts,calcfc,noeigentest,gdiis)

iop(1/8=18) freq=noraman

#N Geom=AllCheck Guess=TCheck SCRF=Check Test GenChk RM062X/6-31G(d) Freq

----

Pointgroup= C1 Stoichiometry= C43H37N3O3S C1[X(C43H37N3O3S)] #Atoms= 87

Charge = 0 Multiplicity = 1

SCF Energy= -2448.13243384 Predicted Change= -5.035723D-09

==

Optimization completed. {Found 2 times}

Item	Max Val.	Criteria	Pass?	RMS Val.	Criteria	Pass?
Force	0.00001 ||	0.00045	[YES]	0.00000 ||	0.00030	[YES]
Displ	0.00126 ||	0.00180	[YES]	0.00126 ||	0.00180	[YES]

Atomic Type	Coordinates (Angstroms) X	Y	Z
N	4.052756	-0.838226	-0.399409
C	2.798714	-0.797481	-0.877352
N	1.884654	0.043053	-0.396124
C	0.523573	0.008070	-0.922693
C	-0.299090	1.261840	-0.778163
H	-0.123983	1.767026	0.170791
H	0.003145	1.953843	-1.576131
O	0.280659	-0.829213	-1.774089
C	2.209732	0.925558	0.744575
H	1.283656	0.975699	1.316677
C	2.611495	2.308335	0.264978
C	3.250943	0.226012	1.641077
H	2.785571	-0.715571	1.967387
C	3.612743	1.041401	2.895338
C	2.404643	1.173475	3.827026
H	2.673714	1.758536	4.710990
H	2.073979	0.184128	4.163481
H	1.550259	1.665209	3.354814
H	3.925371	2.043383	2.573104
C	4.774896	0.402261	3.663362
H	5.714700	0.432866	3.104067
H	4.551295	-0.643398	3.907139

H	4.938191	0.936405	4.603676
C	4.475956	-0.125680	0.810568
H	5.130055	-0.806722	1.357550
H	5.049012	0.765203	0.526162
C	4.908718	-1.699686	-1.099276
C	4.245138	-2.365580	-2.126719
S	2.548294	-1.906212	-2.203284
C	6.263526	-1.906422	-0.852798
H	6.783893	-1.374702	-0.064040
C	6.932406	-2.814793	-1.664947
H	7.988583	-2.995024	-1.496807
C	6.268984	-3.494703	-2.692487
H	6.815516	-4.198490	-3.310753
C	4.917054	-3.277238	-2.934967
H	4.399360	-3.799801	-3.732392
C	2.168873	3.431549	0.968912
H	1.511809	3.305750	1.826234
C	2.549118	4.711577	0.576094
H	2.194246	5.572952	1.133043
C	3.371361	4.885111	-0.534609
H	3.665008	5.882619	-0.845466
C	3.803778	3.773196	-1.252750
H	4.433083	3.899622	-2.128139
C	3.423982	2.492734	-0.857039
H	3.753217	1.639069	-1.445103
C	-1.796528	0.940829	-0.939537
H	-1.951319	0.660804	-1.989288
C	-2.549034	2.246609	-0.702194
C	-2.279853	-0.198055	-0.051278
C	-3.601844	-0.678101	-0.343963
C	-1.445232	-0.824121	0.873718
C	-1.957741	-1.718474	1.966133
O	-0.172952	-0.658949	0.904838

C	-2.869319	3.082829	-1.773116
H	-2.627161	2.765613	-2.785470
C	-3.503007	4.305229	-1.560461
H	-3.752065	4.938722	-2.406929
C	-3.822874	4.710621	-0.266330
H	-4.320022	5.661155	-0.098039
C	-3.503519	3.884297	0.809866
H	-3.751675	4.189871	1.822356
C	-2.871021	2.663003	0.591376
H	-2.632041	2.011207	1.428983
O	-4.311794	0.097152	-1.248816
C	-5.494703	-0.553464	-1.427596
C	-5.460441	-1.701111	-0.628000
N	-4.244642	-1.755676	0.033577
C	-6.560676	-0.200562	-2.233809
H	-6.546603	0.699129	-2.839034
C	-7.652784	-1.071467	-2.217495
H	-8.520825	-0.844627	-2.828119
C	-7.647512	-2.229040	-1.426651
H	-8.515749	-2.880812	-1.441204
C	-6.558310	-2.563014	-0.622989
H	-6.556415	-3.460121	-0.012521
C	-3.064643	-1.368089	2.743069
H	-3.627524	-0.473695	2.490999
C	-3.443693	-2.155457	3.824572
H	-4.301726	-1.870691	4.426232
C	-2.723662	-3.308939	4.136924
H	-3.023103	-3.926341	4.978696
C	-1.613922	-3.660517	3.371338
H	-1.046666	-4.554444	3.613500
C	-1.224622	-2.858338	2.301625
H	-0.345779	-3.106160	1.714306

Statistical Thermodynamic Analysis

Temperature= 298.150 Kelvin   Pressure= 1.00000 Atm

==

SCF Energy=   -2448.13243384   Predicted Change= -5.035723D-09

Zero-point correction (ZPE)=   -2447.4195 0.71289

Internal Energy (U)=   -2447.3792 0.75322

Enthalpy (H)=   -2447.3782 0.75416

Gibbs Free Energy (G)=   -2447.4954 0.63699

Frequencies -- -107.7350   11.6842   19.6170

==

#M062X/6-31+G(d,p) scf=(maxcycle=300,direct,tight) density=current

SCRF=(PCM,SOLVENT=THF)

Pointgroup= C1   Stoichiometry= C43H37N3O3S   C1[X(C43H37N3O3S)] #Atoms= 87

Charge = 0   Multiplicity = 1

SCF Energy= -2448.23239713

==

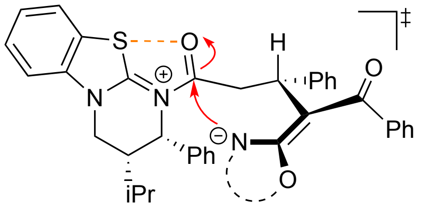

TS-Lactamization-(X=O)-Minor

Supporting Information: 0045-Oxazole-Si-lactamization.log

Using Gaussian 09: AM64L-G09RevD.01 24-Apr-2013

==

#m062X/6-31G(d) scf=(maxcycle=300,direct,tight) density=current

SCRF=(PCM,SOLVENT=THF) opt=(maxcycle=250,ts,calcfc,noeigentest)

freq=noraman

#N Geom=AllCheck Guess=TCheck SCRF=Check GenChk RM062X/6-31G(d) Freq

----

Pointgroup= C1 Stoichiometry= C43H37N3O3S C1[X(C43H37N3O3S)] #Atoms= 87

Charge = 0 Multiplicity = 1

SCF Energy= -2448.12448628 Predicted Change= -9.033427D-09

===

Optimization completed on the basis of negligible forces. {Found 2 times}

Item	Max Val.	Criteria	Pass?	RMS Val.	Criteria	Pass?
Force	0.00000 ||	0.00045	[YES]	0.00000 ||	0.00030	[YES]
Displ	0.01384 ||	0.00180	[NO]	0.01384 ||	0.00180	[NO]

Atomic Type	Coordinates (Angstroms) X	Y	Z
N	3.772491	-0.328835	-0.150724
C	2.589181	-0.346402	-0.787701
N	1.511136	0.255587	-0.295704
C	0.235380	0.192891	-1.048362
C	-0.715372	1.344411	-0.786366
O	0.279029	-0.369762	-2.134793
C	1.575896	0.986362	0.988353
C	1.784588	2.472575	0.748464
C	2.637333	0.344477	1.906234
C	2.769861	1.049536	3.269578
C	1.484312	0.900336	4.088074
C	3.946516	0.487836	4.074174
C	3.968689	0.268575	1.173519
C	4.810961	-0.970572	-0.841431
C	4.365022	-1.530304	-2.035760
S	2.651062	-1.228262	-2.295915
C	6.142006	-1.068039	-0.444034
C	7.012596	-1.759145	-1.279312

C	6.568527	-2.332656	-2.475490
C	5.239285	-2.221744	-2.868278
C	2.684931	2.935528	-0.215117
C	2.887589	4.301756	-0.395793
C	2.188358	5.221786	0.380605
C	1.277527	4.769367	1.332549
C	1.074962	3.403937	1.510662
C	-2.135330	1.070111	-1.305039
C	-2.921956	2.356957	-1.090001
C	-2.798176	-0.141270	-0.669072
C	-4.168472	-0.364122	-1.068733
C	-5.086463	-1.261410	-0.277544
O	-4.658524	0.231955	-2.036973
C	-2.018620	-1.106243	-0.046643
C	-3.499110	2.650737	0.147160
C	-4.146769	3.864883	0.361685
C	-4.222003	4.808883	-0.660907
C	-3.646134	4.526553	-1.897733
C	-3.000377	3.310071	-2.106481
O	-2.524143	-2.354627	0.245778
C	-1.460271	-3.113145	0.650775
C	-0.313915	-2.308423	0.614264
N	-0.704150	-1.031831	0.242176
C	-1.466698	-4.439073	1.021636
C	-0.224177	-4.996193	1.352531
C	0.939937	-4.229704	1.298070
C	0.917291	-2.879424	0.931455
C	-5.048500	-1.315645	1.117679
C	-5.976128	-2.080628	1.818075
C	-6.944973	-2.807949	1.127697
C	-6.992415	-2.753585	-0.264519
C	-6.076060	-1.970954	-0.960568
H	-0.769428	1.605505	0.270630

H	-0.303040	2.212921	-1.317675
H	0.602784	0.812158	1.452996
H	2.303815	-0.683681	2.101279
H	1.281581	-0.158391	4.287633
H	0.605995	1.318730	3.590073
H	1.589830	1.409673	5.050140
H	2.955913	2.115782	3.087420
H	3.951840	0.923946	5.077106
H	4.913899	0.712541	3.615821
H	3.859516	-0.600209	4.183025
H	4.661248	-0.382017	1.709552
H	4.429318	1.257501	1.059176
H	6.495910	-0.616048	0.475812
H	8.055452	-1.849394	-0.995254
H	7.268820	-2.867147	-3.108112
H	4.890917	-2.659224	-3.797932
H	3.223324	2.235771	-0.850216
H	3.587776	4.645368	-1.150540
H	2.343065	6.286226	0.235995
H	0.713357	5.478348	1.929983
H	0.345145	3.061195	2.239990
H	-2.067429	0.905667	-2.389380
H	-3.448730	1.908176	0.940872
H	-4.596034	4.073061	1.328670
H	-4.728658	5.755125	-0.496529
H	-3.704749	5.252460	-2.703751
H	-2.555377	3.091127	-3.074914
H	-2.383952	-5.016961	1.039301
H	-0.169588	-6.039802	1.643826
H	1.891698	-4.690661	1.543439
H	1.841099	-2.313456	0.883962
H	-4.288315	-0.750957	1.651010
H	-5.945287	-2.109509	2.903261

H	-7.665044	-3.409849	1.674261
H	-7.749154	-3.314035	-0.805493
H	-6.114708	-1.898352	-2.043276

Statistical Thermodynamic Analysis

Temperature= 298.150 Kelvin   Pressure= 1.00000 Atm

===

SCF Energy=   -2448.12448628   Predicted Change= -9.033427D-09

Zero-point correction (ZPE)=   -2447.4128 0.71158

Internal Energy (U)=   -2447.3724 0.75204

Enthalpy (H)=   -2447.3715 0.75298

Gibbs Free Energy (G)=   -2447.4892 0.63522

Frequencies -- -138.4921   4.8783   17.7797

===

#m062X/6-31+G(d,p) scf=(maxcycle=300,direct,tight) density=current SCRF=(PCM,SOLVENT=THF)

Pointgroup= C1 Stoichiometry= C43H37N3O3S C1[X(C43H37N3O3S)] #Atoms= 87

Charge = 0   Multiplicity = 1

SCF Energy= -2448.22560001

===

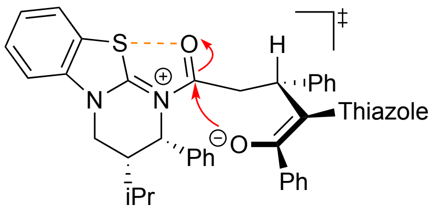

TS-Lactonization-(X=S)-Minor

Supporting Information: 0045-thiazole-Si-lactonization.log

Using Gaussian 09: AM64L-G09RevC.01 23-Sep-2011

===

#M062X/6-31G(d) scf=(maxcycle=300,direct,tight) density=current
SCRF=(PCM,SOLVENT=THF) opt=(maxcycle=250,ts,calcfc,noeigentest,gdiis)
iop(1/8=18) freq=noraman
#N Geom=AllCheck Guess=TCheck SCRF=Check Test GenChk RM062X/6-31G(d) Freq

----

Pointgroup= C1 Stoichiometry= C43H37N3O2S2 C1[X(C43H37N3O2S2)] #Atoms= 87
Charge = 0 Multiplicity = 1

SCF Energy= -2771.09554176 Predicted Change= -1.272284D-08

===

Optimization completed. {Found 1 times}

Item	Max Val.		Criteria	Pass?	RMS Val.		Criteria	Pass?
Force	0.00002	||	0.00045	[YES]	0.00000	||	0.00030	[YES]
Displ	0.00204	||	0.00180	[NO]	0.00204	||	0.00180	[YES]

Atomic Type	Coordinates (Angstroms) X	Y	Z
N	4.074300	-1.024251	-0.331106
C	2.839139	-0.899864	-0.843806
N	1.995443	0.045774	-0.434420
C	0.636476	0.079602	-0.965785
C	-0.108433	1.386253	-0.876725
H	0.115962	1.931159	0.038682
H	0.214677	2.015284	-1.717332
O	0.335433	-0.779481	-1.775372
C	2.376387	0.957337	0.665577
H	1.448015	1.122602	1.210977
C	2.915371	2.267062	0.120861
C	3.325564	0.215233	1.626477
H	2.765711	-0.661729	1.982817
C	3.727027	1.058035	2.850463

C	2.511586	1.342688	3.737191
H	2.808645	1.942381	4.602207
H	2.082774	0.404134	4.106639
H	1.718914	1.886183	3.216766
H	4.136477	2.011063	2.490244
C	4.807221	0.356929	3.681444
H	4.488063	-0.654603	3.960337
H	4.989106	0.915631	4.603877
H	5.761617	0.283410	3.151560
C	4.533114	-0.287844	0.851732
H	5.109918	-0.993732	1.451663
H	5.191064	0.531917	0.537367
C	4.862549	-1.999923	-0.956198
C	4.164882	-2.662327	-1.962989
S	2.520001	-2.057446	-2.112993
C	6.185518	-2.314688	-0.657149
H	6.732247	-1.785804	0.115352
C	6.787194	-3.327233	-1.394923
H	7.817471	-3.592808	-1.184360
C	6.088814	-4.003773	-2.401380
H	6.581790	-4.791594	-2.960400
C	4.769498	-3.678291	-2.696947
H	4.225814	-4.198751	-3.478228
C	3.757665	2.307961	-0.993961
H	4.009793	1.394472	-1.527404
C	4.267582	3.520419	-1.452066
H	4.917261	3.534777	-2.321363
C	3.937024	4.708026	-0.804498
H	4.331244	5.652955	-1.164520
C	3.086143	4.678563	0.297968
H	2.809988	5.600305	0.800077
C	2.575861	3.465992	0.753451
H	1.899734	3.454151	1.604913

C	-1.630103	1.153086	-0.993168
H	-1.828036	0.886363	-2.040466
C	-2.288410	2.507078	-0.737334
C	-2.167031	0.038285	-0.104814
C	-3.495644	-0.440265	-0.397687
C	-1.363479	-0.588500	0.846988
C	-1.914580	-1.455555	1.944102
O	-0.089053	-0.444122	0.902443
C	-2.590574	2.923017	0.561722
H	-2.406908	2.239224	1.387782
C	-3.139855	4.180858	0.795188
H	-3.376599	4.486560	1.810208
C	-3.394275	5.042732	-0.270567
H	-3.829954	6.020971	-0.090966
C	-3.092558	4.638594	-1.569366
H	-3.292083	5.301559	-2.406203
C	-2.541552	3.379594	-1.797879
H	-2.315508	3.061189	-2.813734
S	-4.672115	0.646040	-1.233966
C	-5.837642	-0.647611	-1.221763
C	-5.272320	-1.773033	-0.585437
N	-3.967709	-1.634922	-0.167585
C	-7.137877	-0.668033	-1.723187
H	-7.559520	0.204436	-2.212815
C	-7.885504	-1.830953	-1.574197
H	-8.902096	-1.866185	-1.953066
C	-7.338369	-2.955736	-0.940012
H	-7.938604	-3.854548	-0.835000
C	-6.041418	-2.937120	-0.447886
H	-5.607215	-3.802938	0.042080
C	-1.185276	-2.582990	2.325793
H	-0.282055	-2.828962	1.776184
C	-1.609508	-3.376035	3.388724

H	-1.042960	-4.260630	3.664825
C	-2.754326	-3.028826	4.102189
H	-3.082718	-3.639402	4.938129
C	-3.472035	-1.886255	3.745254
H	-4.356656	-1.603091	4.308044
C	-3.055816	-1.106291	2.671745
H	-3.614996	-0.218043	2.388809

Statistical Thermodynamic Analysis

Temperature= 298.150 Kelvin Pressure= 1.00000 Atm

==

SCF Energy= -2771.09554176 Predicted Change= -1.272284D-08

Zero-point correction (ZPE)= -2770.3865 0.70897

Internal Energy (U)= -2770.3455 0.74998

Enthalpy (H)= -2770.3446 0.75093

Gibbs Free Energy (G)= -2770.4636 0.63190

Frequencies -- -116.2652 12.0058 17.8171

==

#M062X/6-31+G(d,p) scf=(maxcycle=300,direct,tight) density=current

SCRF=(PCM,SOLVENT=THF)

Pointgroup= C1 Stoichiometry= C43H37N3O2S2 C1[X(C43H37N3O2S2)] #Atoms= 87

Charge = 0 Multiplicity = 1

SCF Energy= -2771.19453032

==

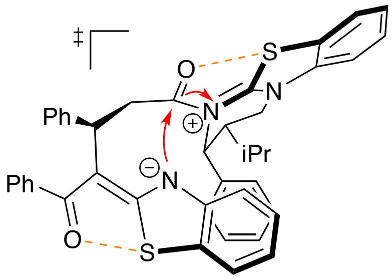

TS-Lactamization-(X=S)-Major

Supporting Information: 0045-thiazole-Re-lactmamization.log

Using Gaussian 09: AM64L-G09RevC.01 23-Sep-2011

==

#m062X/6-31G(d) scf=(maxcycle=300,direct,tight) density=current

SCRF=(PCM,SOLVENT=THF) opt=(maxcycle=250,ts,calcfc,noeigentest)

freq=noraman

#N Geom=AllCheck Guess=TCheck SCRF=Check GenChk RM062X/6-31G(d) Freq

----

Pointgroup= C1 Stoichiometry= C43H37N3O2S2 C1[X(C43H37N3O2S2)] #Atoms= 87

Charge = 0 Multiplicity = 1

SCF Energy= -2771.09894699 Predicted Change= -7.886391D-09

==

Optimization completed. {Found 2 times}

Item	Max Val.	Criteria	Pass?	RMS Val.	Criteria	Pass?
Force	0.00001 ||	0.00045	[YES]	0.00000 ||	0.00030	[YES]
Displ	0.00147 ||	0.00180	[YES]	0.00147 ||	0.00180	[YES]

Atomic Type	Coordinates (Angstroms) X	Y	Z
N	3.439401	-0.200867	-1.301812
C	2.494968	0.694232	-0.977469
N	1.239225	0.326889	-0.716260
C	0.167113	1.331735	-0.824088

C	-1.053806	0.866445	-1.604821
O	0.495080	2.495688	-0.729143
C	0.958492	-1.115858	-0.564327
C	1.333109	-1.675636	0.799009
C	1.660485	-1.841160	-1.735568
C	1.304714	-3.332224	-1.859813
C	-0.175026	-3.505277	-2.215387
C	2.167447	-4.025786	-2.918963
C	3.160865	-1.617240	-1.587952
C	4.716376	0.350412	-1.470172
C	4.711641	1.733692	-1.300476
S	3.103275	2.326886	-0.907782
C	5.883904	-0.340846	-1.782987
C	7.049699	0.401630	-1.928242
C	7.049387	1.791762	-1.767251
C	5.879251	2.474497	-1.453852
C	2.439556	-1.261014	1.548379
C	2.749953	-1.886581	2.751736
C	1.965838	-2.937961	3.222506
C	0.856183	-3.347589	2.491883
C	0.540802	-2.713390	1.293414
C	-2.450765	0.877494	-0.926988
C	-2.871412	2.309814	-0.576898
C	-2.604133	-0.135002	0.186453
C	-1.676104	-0.035467	1.242805
C	-3.635303	-1.110865	0.270031
C	-4.745172	-1.160358	-0.753096
O	-3.681788	-1.971624	1.176994
C	-3.192432	2.710912	0.721228
C	-3.607826	4.015662	0.984630
C	-3.718064	4.942905	-0.047320
C	-3.417248	4.551550	-1.350709
C	-3.000947	3.249232	-1.607407

S	-1.795055	-0.908651	2.791779
C	-0.371135	-0.050557	3.350924
C	0.089347	0.821381	2.343763
N	-0.651461	0.800491	1.186338
C	0.301212	-0.161634	4.563862
C	1.433831	0.621326	4.774487
C	1.886259	1.502168	3.783541
C	1.221887	1.613798	2.568427
C	-5.445244	-0.024973	-1.171936
C	-6.516643	-0.142655	-2.055681
C	-6.893926	-1.394911	-2.535035
C	-6.208240	-2.533662	-2.112177
C	-5.151607	-2.415762	-1.215765
H	-1.075630	1.565711	-2.447085
H	-0.891044	-0.123963	-2.035601
H	-0.122096	-1.219283	-0.659303
H	1.332116	-1.340900	-2.659099
H	-0.386076	-3.048052	-3.189602
H	-0.852469	-3.056710	-1.481708
H	-0.424998	-4.567938	-2.282903
H	1.498445	-3.812361	-0.892305
H	3.224710	-4.060325	-2.639566
H	2.082095	-3.515989	-3.886346
H	1.832170	-5.057901	-3.054975
H	3.685041	-1.854646	-2.515981
H	3.578946	-2.223467	-0.775344
H	5.888040	-1.418504	-1.902975
H	7.974910	-0.110227	-2.169408
H	7.973971	2.345941	-1.886433
H	5.875231	3.551782	-1.327750
H	3.051922	-0.423250	1.227795
H	3.600746	-1.539286	3.329493
H	2.209828	-3.421297	4.163601

H	0.217693	-4.144322	2.860375
H	-0.352262	-3.012887	0.749025
H	-3.111753	0.562390	-1.742047
H	-3.125486	1.994175	1.533191
H	-3.848216	4.304323	2.003742
H	-4.042908	5.958325	0.158861
H	-3.513875	5.259045	-2.169162
H	-2.784889	2.954682	-2.632731
H	-0.049658	-0.845306	5.331707
H	1.966917	0.549594	5.717588
H	2.766210	2.110970	3.971090
H	1.553567	2.300550	1.794056
H	-5.164445	0.953186	-0.787784
H	-7.060536	0.745200	-2.364310
H	-7.724743	-1.485165	-3.228456
H	-6.505708	-3.513243	-2.474794
H	-4.627490	-3.296152	-0.855653

Statistical Thermodynamic Analysis

Temperature= 298.150 Kelvin Pressure= 1.00000 Atm

===

SCF Energy= -2771.09894699 Predicted Change= -7.886391D-09

Zero-point correction (ZPE)= -2770.3903 0.70860

Internal Energy (U)= -2770.3496 0.74928

Enthalpy (H)= -2770.3487 0.75022

Gibbs Free Energy (G)= -2770.4648 0.63406

Frequencies -- -75.4058 11.9587 16.3033

===

#m062X/6-31+G(d,p) scf=(maxcycle=300,direct,tight) density=current SCRF=(PCM,SOLVENT=THF)

Pointgroup= C1 Stoichiometry= C43H37N3O2S2 C1[X(C43H37N3O2S2)] #Atoms= 87

Charge = 0 Multiplicity = 1

SCF Energy= -2771.19836373

==

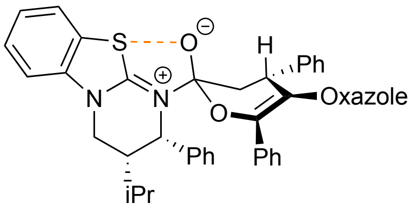

VIII

Supporting Information: 0050-tetrahedral-int-from-oxazole-Si-lactonization.log

----

Using Gaussian 09: AM64L-G09RevD.01 24-Apr-2013

==

#m062X/6-31G(d) scf=(maxcycle=300,direct,tight) density=current
SCRF=(PCM,SOLVENT=THF) opt=(maxcycle=250) freq=noraman
#N Geom=AllCheck Guess=TCheck SCRF=Check GenChk RM062X/6-31G(d) Freq

----

Pointgroup= C1 Stoichiometry= C43H37N3O3S C1[X(C43H37N3O3S)] #Atoms= 87
Charge = 0 Multiplicity = 1

SCF Energy= -2448.13904796 Predicted Change= -5.266725D-09

==

Optimization completed. {Found 2 times}

Item	Max Val.	Criteria	Pass?	RMS Val.	Criteria	Pass?
Force	0.00001 ||	0.00045	[YES]	0.00000 ||	0.00030	[YES]
Displ	0.00155 ||	0.00180	[YES]	0.00155 ||	0.00180	[YES]

Atomic Type	Coordinates (Angstroms) X	Y	Z
N	4.174167	-0.503131	-0.414099

C	2.906244	-0.527409	-0.878476
N	1.905291	0.009387	-0.220100
C	0.459879	-0.161416	-0.751993
C	-0.295338	1.162256	-0.619377
H	-0.239488	1.573866	0.394026
H	0.131464	1.894444	-1.311761
O	0.431499	-0.730068	-1.882987
C	2.136941	0.665498	1.075495
H	1.214385	0.505293	1.637672
C	2.370344	2.159200	0.911356
C	3.275038	-0.070685	1.816325
H	2.945254	-1.114756	1.920505
C	3.556753	0.485352	3.223909
C	2.360796	0.258739	4.152441
H	2.566563	0.677774	5.141720
H	2.172286	-0.814352	4.273475
H	1.439205	0.718713	3.785911
H	3.737742	1.564733	3.133846
C	4.803043	-0.157048	3.842428
H	5.720416	0.110731	3.310238
H	4.714185	-1.250222	3.848849
H	4.918444	0.174982	4.878269
C	4.520217	-0.062968	0.938959
H	5.261195	-0.768818	1.319670
H	4.975413	0.934768	0.893573
C	5.124534	-1.039105	-1.292841
C	4.534094	-1.525362	-2.456861
S	2.780627	-1.304559	-2.450291
C	6.499034	-1.107064	-1.087298
H	6.955769	-0.711660	-0.186532
C	7.272996	-1.691677	-2.085766
H	8.347521	-1.756766	-1.951774
C	6.688106	-2.190647	-3.252705

H	7.311718	-2.642676	-4.016632
C	5.311725	-2.111708	-3.449017
H	4.854983	-2.497477	-4.354807
C	1.826662	3.042712	1.848324
H	1.212429	2.656328	2.658320
C	2.049251	4.413029	1.748599
H	1.615825	5.084135	2.483408
C	2.814618	4.920862	0.701600
H	2.985256	5.989476	0.617873
C	3.347705	4.050724	-0.245913
H	3.933621	4.437861	-1.073730
C	3.124733	2.679300	-0.143677
H	3.531368	2.020687	-0.907122
C	-1.758754	0.880208	-0.974773
H	-1.794665	0.632050	-2.042996
C	-2.595484	2.125178	-0.740942
C	-2.258768	-0.318621	-0.180934
C	-3.696229	-0.553970	-0.264617
C	-1.399652	-1.166475	0.443000
C	-1.740483	-2.392197	1.214302
O	-0.062254	-1.029244	0.374887
C	-2.925846	2.966054	-1.804824
H	-2.613891	2.696893	-2.811429
C	-3.651876	4.135460	-1.589998
H	-3.903146	4.776296	-2.430048
C	-4.061709	4.477098	-0.303143
H	-4.631462	5.385728	-0.133688
C	-3.740036	3.641908	0.765475
H	-4.059320	3.897943	1.771478
C	-3.011955	2.475789	0.546547
H	-2.768186	1.820375	1.380401
O	-4.246732	-0.195061	-1.475556
C	-5.577903	-0.448210	-1.344251

C	-5.779440	-0.957000	-0.060185
N	-4.554178	-0.994393	0.601922
C	-6.592546	-0.260624	-2.268429
H	-6.401620	0.138726	-3.257936
C	-7.870083	-0.617664	-1.841775
H	-8.706925	-0.495036	-2.521737
C	-8.100216	-1.131660	-0.555006
H	-9.112866	-1.395843	-0.267383
C	-7.063355	-1.309512	0.355490
H	-7.236925	-1.707507	1.349676
C	-2.596701	-2.385265	2.316524
H	-3.086659	-1.463817	2.606271
C	-2.819454	-3.559680	3.032421
H	-3.481037	-3.546608	3.893159
C	-2.194988	-4.744411	2.651133
H	-2.376212	-5.658584	3.208563
C	-1.330531	-4.753470	1.556285
H	-0.838112	-5.673690	1.256994
C	-1.093365	-3.579530	0.851470
H	-0.409627	-3.565036	0.007831

Statistical Thermodynamic Analysis

Temperature= 298.150 Kelvin Pressure= 1.00000 Atm

==

SCF Energy= -2448.13904796 Predicted Change= -5.266725D-09

Zero-point correction (ZPE)= -2447.4261 0.71287

Internal Energy (U)= -2447.3855 0.75353

Enthalpy (H)= -2447.3845 0.75448

Gibbs Free Energy (G)= -2447.5038 0.63521

Frequencies -- 8.9015 13.0897 21.0731

==

#m062X/6-31+G(d,p) scf=(maxcycle=300,direct,tight) density=current

SCRF=(PCM,SOLVENT=THF)

Pointgroup= C1 Stoichiometry= C43H37N3O3S C1[X(C43H37N3O3S)] #Atoms= 87

Charge = 0 Multiplicity = 1

SCF Energy= -2448.23916785

==

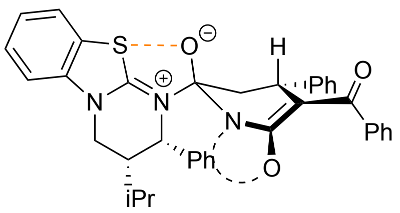

VIII

Supporting Information: 0050-tetrahedral-int-from-oxazole-Si-lactamization.log

----

Using Gaussian 09: AM64L-G09RevD.01 24-Apr-2013

==

#m062X/6-31G(d) scf=(maxcycle=300,direct,tight) density=current

SCRF=(PCM,SOLVENT=THF) opt=(maxcycle=250) freq=noraman

#N Geom=AllCheck Guess=TCheck SCRF=Check GenChk RM062X/6-31G(d) Freq

----

Pointgroup= C1 Stoichiometry= C43H37N3O3S C1[X(C43H37N3O3S)] #Atoms= 87

Charge = 0 Multiplicity = 1

SCF Energy= -2448.13199510 Predicted Change= -3.045435D-09

==

Optimization completed. {Found 2 times}

Item	Max Val.	Criteria	Pass?	RMS Val.	Criteria	Pass?
Force	0.00000 ||	0.00045	[YES]	0.00000 ||	0.00030	[YES]
Displ	0.00129 ||	0.00180	[YES]	0.00129 ||	0.00180	[YES]

Atomic Coordinates (Angstroms)

Type	X	Y	Z
N	3.771633	-0.078233	-0.005113
C	2.671589	-0.016610	-0.790772
N	1.452120	0.037949	-0.308620
C	0.199515	0.005173	-1.333214
C	-0.613531	1.270652	-1.026668
O	0.600768	-0.187176	-2.520236
C	1.243366	0.092400	1.149270
C	1.144573	1.511999	1.678397
C	2.333015	-0.753543	1.840076
C	2.154205	-0.895522	3.369942
C	2.627400	-2.281365	3.819712
C	2.856768	0.184548	4.201119
C	3.703061	-0.219155	1.449903
C	4.989100	-0.031028	-0.696077
C	4.798728	0.013606	-2.074849
S	3.085704	-0.003413	-2.505146
C	6.262112	-0.024455	-0.134297
C	7.350098	0.014283	-1.001782
C	7.168239	0.047030	-2.386830
C	5.888975	0.046860	-2.937003
C	0.183742	1.802765	2.650668
C	0.099377	3.075590	3.207443
C	0.972221	4.077864	2.789630
C	1.917849	3.802472	1.804702
C	2.000999	2.528055	1.248043
C	-2.046987	1.195196	-1.577757
C	-2.739350	2.501630	-1.230188
C	-2.756742	-0.023551	-1.008920
C	-4.206690	-0.103178	-1.145706
C	-5.025989	-0.977175	-0.237057
O	-4.791116	0.586457	-1.979668

C	-1.975325	-1.099204	-0.692070
C	-3.173707	2.755027	0.073675
C	-3.738845	3.981020	0.410894
C	-3.874201	4.978501	-0.554002
C	-3.441032	4.736684	-1.855156
C	-2.877200	3.506433	-2.187609
O	-2.438863	-2.340679	-0.364698
C	-1.354819	-3.188914	-0.322628
C	-0.198873	-2.464934	-0.614307
N	-0.606064	-1.142448	-0.753716
C	-1.359487	-4.538846	-0.054146
C	-0.116282	-5.182874	-0.105846
C	1.046012	-4.481763	-0.425203
C	1.029855	-3.106833	-0.689855
C	-4.665869	-1.209251	1.092403
C	-5.499518	-1.948994	1.925897
C	-6.694506	-2.470128	1.432017
C	-7.062085	-2.236844	0.107266
C	-6.236402	-1.481734	-0.718757
H	-0.678974	1.449747	0.050462
H	-0.074173	2.109464	-1.477189
H	0.284207	-0.405605	1.325150
H	2.235503	-1.755654	1.401505
H	3.693727	-2.418792	3.603526
H	2.073514	-3.076342	3.310675
H	2.490286	-2.404443	4.898318
H	1.076606	-0.830285	3.572082
H	3.946214	0.077150	4.141022
H	2.578318	0.071799	5.253132
H	2.594230	1.199821	3.890907
H	4.488821	-0.918363	1.750355
H	3.908028	0.754099	1.908828
H	6.407690	-0.039987	0.940307

H	8.353898	0.021438	-0.590579
H	8.031874	0.075549	-3.042446
H	5.743418	0.075074	-4.012046
H	-0.508659	1.026085	2.968756
H	-0.653958	3.285165	3.960382
H	0.905543	5.072591	3.218557
H	2.589353	4.582649	1.460250
H	2.730283	2.339097	0.463845
H	-1.995614	1.114878	-2.672359
H	-3.073204	1.976045	0.827509
H	-4.075633	4.158773	1.428227
H	-4.317422	5.934948	-0.293497
H	-3.546634	5.504703	-2.615849
H	-2.543858	3.319499	-3.205846
H	-2.278493	-5.066785	0.172522
H	-0.063867	-6.247882	0.092356
H	1.990034	-5.013833	-0.480881
H	1.934201	-2.579517	-0.970632
H	-3.734996	-0.797855	1.473634
H	-5.219820	-2.117573	2.961406
H	-7.341361	-3.053312	2.080645
H	-7.994868	-2.637674	-0.277422
H	-6.515734	-1.271073	-1.746481

Statistical Thermodynamic Analysis

Temperature= 298.150 Kelvin Pressure= 1.00000 Atm

===

SCF Energy= -2448.13199510 Predicted Change= -3.045435D-09

Zero-point correction (ZPE)= -2447.4191 0.71280

Internal Energy (U)= -2447.3784 0.75354

Enthalpy (H)= -2447.3775 0.75449

Gibbs Free Energy (G)= -2447.4950 0.63696

Frequencies -- 10.2160 14.2392 25.5468

==

#m062X/6-31+G(d,p) scf=(maxcycle=300,direct,tight) density=current
SCRF=(PCM,SOLVENT=THF)

Pointgroup= C1 Stoichiometry= C43H37N3O3S C1[X(C43H37N3O3S)] #Atoms= 87
Charge = 0 Multiplicity = 1

SCF Energy= -2448.23442163

==

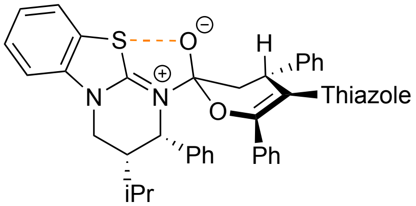

VIII

Supporting Information: 0050-tetrahedral-int-from-thiazole-Si-lactonization.log

----

Using Gaussian 09: AM64L-G09RevC.01 23-Sep-2011

==

#M062X/6-31G(d) scf=(maxcycle=300,direct,tight) density=current
SCRF=(PCM,SOLVENT=THF) opt=(maxcycle=250,gdiis) iop(1/8=18) freq=noraman
#N Geom=AllCheck Guess=TCheck SCRF=Check Test GenChk RM062X/6-31G(d) Freq

----

Pointgroup= C1 Stoichiometry= C43H37N3O2S2 C1[X(C43H37N3O2S2)] #Atoms= 87
Charge = 0 Multiplicity = 1

SCF Energy= -2771.10239863 Predicted Change= -4.638835D-09

==

Optimization completed. {Found 1 times}
Item Max Val. Criteria Pass? RMS Val. Criteria Pass?
Force 0.00000 || 0.00045 [YES] 0.00000 || 0.00030 [YES]

Displ 0.00705 || 0.00180 [NO] 0.00705 || 0.00180 [YES]

Atomic Type	Coordinates (Angstroms) X	Y	Z
N	-4.316757	0.554031	-0.348926
C	-3.057680	0.545472	-0.838252
N	-2.046487	0.044543	-0.168391
C	-0.609052	0.165257	-0.742440
C	0.099871	-1.185256	-0.615133
H	0.063652	-1.582219	0.405324
H	-0.376608	-1.907849	-1.284990
O	-0.598932	0.710262	-1.886952
C	-2.259846	-0.543576	1.162548
H	-1.322751	-0.370870	1.696064
C	-2.519891	-2.040089	1.081374
C	-3.373895	0.247073	1.884212
H	-3.033296	1.292123	1.918797
C	-3.629029	-0.216870	3.329557
C	-2.410390	0.059437	4.214409
H	-1.502784	-0.438684	3.863040
H	-2.599777	-0.283447	5.235770
H	-2.205845	1.135787	4.251954
H	-3.820318	-1.297960	3.313999
C	-4.856252	0.475057	3.932420
H	-4.758947	1.565645	3.867049
H	-4.951404	0.211273	4.989751
H	-5.787075	0.182197	3.438097
C	-4.637516	0.197718	1.034756
H	-5.367008	0.929556	1.387678
H	-5.098641	-0.798075	1.057919
C	-5.284144	1.029128	-1.243880
C	-4.715997	1.435722	-2.448900

S	-2.962117	1.220403	-2.458773
C	-6.655201	1.107625	-1.019971
H	-7.095572	0.773096	-0.086926
C	-7.448258	1.620372	-2.042753
H	-8.520630	1.691201	-1.895067
C	-6.885551	2.039678	-3.251154
H	-7.524156	2.436147	-4.033245
C	-5.512361	1.951372	-3.465023
H	-5.071921	2.275564	-4.402582
C	-1.999705	-2.877438	2.072579
H	-1.385725	-2.455430	2.864783
C	-2.245513	-4.247204	2.049799
H	-1.829755	-4.881882	2.826061
C	-3.011301	-4.801538	1.026780
H	-3.200380	-5.870047	1.003723
C	-3.520896	-3.978646	0.026152
H	-4.106520	-4.403238	-0.783301
C	-3.274804	-2.607485	0.051468
H	-3.663488	-1.987951	-0.752758
C	1.559194	-0.958642	-1.022242
H	1.551821	-0.686816	-2.087951
C	2.347805	-2.244435	-0.862938
C	2.129384	0.208490	-0.229787
C	3.584732	0.392861	-0.331498
C	1.319513	1.106385	0.382850
C	1.741909	2.344723	1.091248
O	-0.029070	1.030516	0.345581
C	2.867134	-2.626732	0.377093
H	2.759108	-1.956006	1.226700
C	3.531539	-3.841744	0.523044
H	3.933035	-4.124023	1.491956
C	3.684957	-4.692536	-0.570177
H	4.204101	-5.639395	-0.456492

C	3.173403	-4.318533	-1.810681
H	3.292920	-4.972555	-2.669474
C	2.511350	-3.101164	-1.953128
H	2.115638	-2.807378	-2.923007
S	4.281146	0.525984	-1.963943
C	5.863763	0.625609	-1.249109
C	5.741190	0.554632	0.152164
N	4.447241	0.409272	0.630706
C	7.110540	0.757257	-1.863118
H	7.198284	0.812025	-2.943177
C	8.235500	0.817743	-1.051902
H	9.215079	0.921646	-1.507575
C	8.125036	0.746682	0.345954
H	9.021538	0.793904	0.955760
C	6.886975	0.615173	0.954177
H	6.784297	0.557852	2.033034
C	2.686858	2.356222	2.119640
H	3.164514	1.430085	2.414900
C	3.011836	3.553493	2.754971
H	3.743846	3.552985	3.557128
C	2.398285	4.742996	2.372125
H	2.656561	5.673570	2.868623
C	1.443741	4.734194	1.354916
H	0.958156	5.657694	1.054234
C	1.108612	3.540302	0.727874
H	0.356565	3.515397	-0.055230

Statistical Thermodynamic Analysis

Temperature= 298.150 Kelvin   Pressure= 1.00000 Atm

===

SCF Energy=   -2771.10239863   Predicted Change= -4.638835D-09

Zero-point correction (ZPE)=   -2770.3931 0.70927

Internal Energy (U)=   -2770.3518 0.75056

Enthalpy (H)= -2770.3508 0.75150

Gibbs Free Energy (G)= -2770.4723 0.63006

Frequencies -- 6.0288 9.8316 18.6432

===

#m062X/6-31+G(d,p) scf=(maxcycle=300,direct,tight) density=current

SCRF=(PCM,SOLVENT=THF)

Pointgroup= C1 Stoichiometry= C43H37N3O2S2 C1[X(C43H37N3O2S2)] #Atoms= 87

Charge = 0 Multiplicity = 1

SCF Energy= -2771.20145237

===

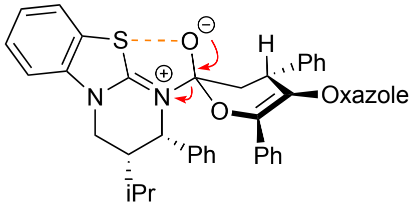

TS-Elimination

Supporting Information: 0055-HYPERBTM-release-from-oxazole-SI-lactonization.log

----

Using Gaussian 09: AM64L-G09RevC.01 23-Sep-2011

===

#M062X/6-31G(d) scf=(maxcycle=300,direct,tight) density=current

SCRF=(PCM,SOLVENT=THF) opt=(maxcycle=250,ts,calcfc,noeigentest,gdiis)

iop(1/8=18) freq=noraman

#N Geom=AllCheck Guess=TCheck SCRF=Check Test GenChk RM062X/6-31G(d) Freq

----

Pointgroup= C1 Stoichiometry= C43H37N3O3S C1[X(C43H37N3O3S)] #Atoms= 87

Charge = 0 Multiplicity = 1

SCF Energy= -2448.13609113 Predicted Change= -1.774365D-09

==

Optimization completed. {Found 2 times}

Item	Max Val.	Criteria	Pass?	RMS Val.	Criteria	Pass?
Force	0.00001 ||	0.00045	[YES]	0.00000 ||	0.00030	[YES]
Displ	0.00135 ||	0.00180	[YES]	0.00135 ||	0.00180	[YES]

Atomic Type	Coordinates (Angstroms) X	Y	Z
N	-4.385021	-0.276331	-0.263759
C	-3.178907	-0.495470	-0.858134
N	-2.130180	0.240319	-0.704515
C	-0.569224	-0.373484	-1.401991
C	-0.069577	-1.290355	-0.300603
H	-0.205787	-0.830406	0.679964
H	-0.624476	-2.231913	-0.333833
O	-0.767067	-0.789211	-2.545064
C	-2.187387	1.446350	0.131624
H	-1.576206	2.183372	-0.393813
C	-1.519696	1.178829	1.471014
C	-3.626945	2.005475	0.199005
H	-3.865823	2.347451	-0.818664
C	-3.755273	3.211097	1.147768
C	-2.856425	4.365478	0.698209
H	-2.994091	5.229270	1.355358
H	-3.111583	4.674409	-0.322582
H	-1.795546	4.103391	0.717913
H	-3.433743	2.885752	2.146872
C	-5.204705	3.698122	1.244215
H	-5.865255	2.959829	1.708583
H	-5.601331	3.935324	0.249311
H	-5.254605	4.607831	1.849672
C	-4.635293	0.911737	0.546740

H	-5.647337	1.251652	0.313909
H	-4.600702	0.654056	1.613225
C	-5.349657	-1.255423	-0.513471
C	-4.876946	-2.256731	-1.365193
S	-3.217005	-1.948439	-1.874901
C	-6.641602	-1.301832	-0.000052
H	-7.005418	-0.535898	0.676236
C	-7.454972	-2.370866	-0.372150
H	-8.465652	-2.425706	0.018308
C	-6.988964	-3.367663	-1.230248
H	-7.638435	-4.191094	-1.506932
C	-5.690676	-3.318518	-1.735609
H	-5.321764	-4.090762	-2.402727
C	-0.396287	1.923177	1.836815
H	-0.025906	2.696504	1.167065
C	0.276949	1.661282	3.029161
H	1.152052	2.247508	3.293824
C	-0.163207	0.640126	3.867330
H	0.364621	0.425658	4.791501
C	-1.275129	-0.119304	3.504330
H	-1.611260	-0.930940	4.142020
C	-1.946826	0.147105	2.314526
H	-2.776517	-0.492306	2.021213
C	1.428112	-1.537809	-0.522074
H	1.564066	-2.132415	-1.434546
C	1.971052	-2.325435	0.657589
C	2.121000	-0.191743	-0.679128
C	3.578062	-0.223310	-0.582398
C	1.421041	0.927232	-0.988922
C	1.938050	2.314460	-1.108394
O	0.100209	0.915269	-1.285449
C	2.266681	-3.683070	0.539712
H	2.152275	-4.171448	-0.425167

C	2.708463	-4.411068	1.643195
H	2.936276	-5.467509	1.536118
C	2.860586	-3.785297	2.878312
H	3.205533	-4.351419	3.738234
C	2.569907	-2.427269	3.003608
H	2.686701	-1.930952	3.962773
C	2.129135	-1.703604	1.900009
H	1.901780	-0.642866	1.997076
O	4.131274	-1.380159	-1.082841
C	5.471740	-1.237925	-0.890618
C	5.675261	0.003589	-0.286040
N	4.437287	0.614757	-0.095189
C	6.491760	-2.118194	-1.211959
H	6.298051	-3.075831	-1.681357
C	7.778390	-1.690351	-0.892065
H	8.621270	-2.334505	-1.120630
C	8.011123	-0.446488	-0.282100
H	9.031306	-0.157110	-0.050972
C	6.968340	0.419367	0.031379
H	7.144476	1.380898	0.501653
C	2.613840	2.937029	-0.056216
H	2.819519	2.376806	0.848909
C	3.018462	4.264092	-0.177177
H	3.539394	4.744965	0.644886
C	2.754590	4.973052	-1.346759
H	3.076981	6.005702	-1.440170
C	2.067903	4.359079	-2.393927
H	1.854181	4.910542	-3.304179
C	1.647225	3.039730	-2.269192
H	1.095269	2.557586	-3.070286

Statistical Thermodynamic Analysis

Temperature= 298.150 Kelvin Pressure= 1.00000 Atm

==

SCF Energy= -2448.13609113 Predicted Change= -1.774365D-09

Zero-point correction (ZPE)= -2447.4236 0.71246

Internal Energy (U)= -2447.3834 0.75268

Enthalpy (H)= -2447.3824 0.75363

Gibbs Free Energy (G)= -2447.4992 0.63687

Frequencies -- -113.0459 11.7940 20.1080

==

#m062X/6-31+G(d,p) scf=(maxcycle=300,direct,tight) density=current

SCRF=(PCM,SOLVENT=THF)

Pointgroup= C1 Stoichiometry= C43H37N3O3S C1[X(C43H37N3O3S)] #Atoms= 87

Charge = 0 Multiplicity = 1

SCF Energy= -2448.23518934

==

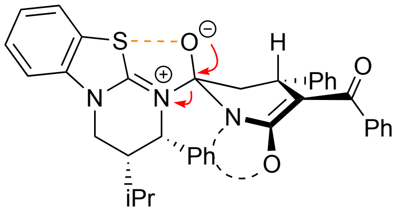

TS-Elimination

Supporting Information: 0055-HYPERBTM-release-from-oxazole-Si-lactamization.log

----

Using Gaussian 09: AM64L-G09RevD.01 24-Apr-2013

==

#m062X/6-31G(d) scf=(maxcycle=300,direct,tight) density=current

SCRF=(PCM,SOLVENT=THF) opt=(maxcycle=250,ts,calcfc,noeigentest)

freq=noraman

#N Geom=AllCheck Guess=TCheck SCRF=Check GenChk RM062X/6-31G(d) Freq

----

Pointgroup= C1 Stoichiometry= C43H37N3O3S C1[X(C43H37N3O3S)] #Atoms= 87

Charge = 0 Multiplicity = 1

SCF Energy= -2448.13233794 Predicted Change= -4.223498D-09

==

Optimization completed. {Found 2 times}

Item	Max Val.	Criteria	Pass?	RMS Val.	Criteria	Pass?
Force	0.00000 ||	0.00045	[YES]	0.00000 ||	0.00030	[YES]
Displ	0.00145 ||	0.00180	[YES]	0.00145 ||	0.00180	[YES]

Atomic Type	Coordinates (Angstroms) X	Y	Z
N	3.756185	-0.014229	0.146123
C	2.720835	0.174985	-0.711567
N	1.467218	0.052210	-0.370555
C	0.267253	0.206706	-1.581877
C	-0.528815	1.436714	-1.132134
O	0.819859	0.178794	-2.710168
C	1.122932	-0.278812	1.019849
C	0.831904	0.948596	1.863401
C	2.212737	-1.201096	1.604983
C	1.898072	-1.728731	3.024006
C	2.451966	-3.147760	3.183885
C	2.406080	-0.840178	4.165276
C	3.565241	-0.509260	1.509406
C	5.018725	0.278361	-0.381917
C	4.945119	0.657752	-1.721058
S	3.286555	0.636353	-2.322613
C	6.235403	0.223300	0.290853
C	7.386533	0.548252	-0.422811
C	7.320850	0.918487	-1.768022
C	6.096959	0.975270	-2.430577

C	-0.270357	0.929028	2.722015
C	-0.545096	2.013412	3.551123
C	0.278990	3.136050	3.523595
C	1.367315	3.172924	2.654044
C	1.639821	2.088086	1.824049
C	-1.998382	1.426809	-1.597969
C	-2.640021	2.693758	-1.058347
C	-2.689713	0.159878	-1.114973
C	-4.147707	0.113480	-1.119117
C	-4.893974	-0.871117	-0.263101
O	-4.793361	0.909443	-1.798273
C	-1.906315	-0.955244	-1.023909
C	-2.917617	2.820237	0.305793
C	-3.424727	4.008467	0.822069
C	-3.661490	5.093149	-0.021833
C	-3.386975	4.976313	-1.381936
C	-2.877735	3.783993	-1.893936
O	-2.342774	-2.229337	-0.800525
C	-1.260587	-3.071831	-0.946425
C	-0.135806	-2.315428	-1.269489
N	-0.555480	-0.992500	-1.250582
C	-1.233076	-4.440822	-0.803946
C	0.007733	-5.057051	-1.016394
C	1.137607	-4.314109	-1.361484
C	1.090316	-2.921675	-1.500096
C	-4.416170	-1.288658	0.981199
C	-5.181501	-2.135110	1.778036
C	-6.425822	-2.577360	1.331130
C	-6.909976	-2.159793	0.091934
C	-6.151867	-1.299148	-0.694448
H	-0.536275	1.515492	-0.042264
H	-0.006979	2.311101	-1.531946
H	0.203476	-0.870508	0.967664

H	2.241177	-2.066432	0.928549
H	3.541669	-3.157614	3.060981
H	2.021424	-3.828295	2.442361
H	2.228087	-3.540099	4.180525
H	0.803946	-1.787921	3.104426
H	3.501276	-0.854859	4.215726
H	2.032825	-1.224306	5.119290
H	2.080054	0.199483	4.070009
H	4.372971	-1.215932	1.722412
H	3.646685	0.331302	2.207477
H	6.289458	-0.055524	1.337660
H	8.347151	0.512981	0.080033
H	8.230242	1.167505	-2.304209
H	6.041444	1.264130	-3.475219
H	-0.921773	0.057797	2.736730
H	-1.407223	1.984317	4.210206
H	0.065568	3.984949	4.165292
H	2.002124	4.052489	2.614688
H	2.476576	2.143896	1.131314
H	-2.027039	1.467343	-2.696065
H	-2.734138	1.974221	0.966624
H	-3.634100	4.088836	1.885016
H	-4.058434	6.021188	0.378261
H	-3.571408	5.813563	-2.048856
H	-2.666160	3.695757	-2.956959
H	-2.123629	-5.002351	-0.546944
H	0.084262	-6.134747	-0.920585
H	2.077468	-4.826967	-1.537332
H	1.956646	-2.342801	-1.800604
H	-3.447116	-0.938069	1.326899
H	-4.809838	-2.448484	2.748832
H	-7.020481	-3.241772	1.950852
H	-7.880237	-2.500473	-0.256141

H -6.521806 -0.945342 -1.651747

Statistical Thermodynamic Analysis

Temperature= 298.150 Kelvin Pressure= 1.00000 Atm

==

SCF Energy= -2448.13233794 Predicted Change= -4.223498D-09

Zero-point correction (ZPE)= -2447.4200 0.71228

Internal Energy (U)= -2447.3799 0.75237

Enthalpy (H)= -2447.3790 0.75331

Gibbs Free Energy (G)= -2447.4939 0.63838

Frequencies -- -32.9649 11.9014 16.3197

==

#m062X/6-31+G(d,p) scf=(maxcycle=300,direct,tight) density=current

SCRF=(PCM,SOLVENT=THF)

Pointgroup= C1 Stoichiometry= C43H37N3O3S C1[X(C43H37N3O3S)] #Atoms= 87

Charge = 0 Multiplicity = 1

SCF Energy= -2448.23415504

==

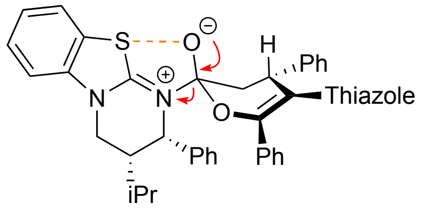

TS-Elimination

Supporting Information: 0055-HYPERBTM-release-from-thiazole-Si-lactonization.log

----

Using Gaussian 09: AM64L-G09RevC.01 23-Sep-2011

==

#M062X/6-31G(d) scf=(maxcycle=300,direct,tight) density=current

SCRF=(PCM,SOLVENT=THF) opt=(maxcycle=250,ts,calcfc,noeigentest)
freq=noraman
#N Geom=AllCheck Guess=TCheck SCRF=Check GenChk RM062X/6-31G(d) Freq

----

Pointgroup= C1 Stoichiometry= C43H37N3O2S2 C1[X(C43H37N3O2S2)] #Atoms= 87
Charge = 0 Multiplicity = 1

SCF Energy= -2771.09926087 Predicted Change= -2.671274D-09

==

Optimization completed. {Found 2 times}

Item	Max Val.	Criteria	Pass?	RMS Val.	Criteria	Pass?
Force	0.00000 ||	0.00045	[YES]	0.00000 ||	0.00030	[YES]
Displ	0.00156 ||	0.00180	[YES]	0.00156 ||	0.00180	[YES]

Atomic Type	Coordinates (Angstroms) X	Y	Z
N	-4.530550	-0.177794	-0.334962
C	-3.315531	-0.411088	-0.902724
N	-2.249736	0.285958	-0.689816
C	-0.705450	-0.343275	-1.349511
C	-0.279105	-1.337396	-0.282070
H	-0.438166	-0.919501	0.713600
H	-0.869530	-2.252545	-0.380206
O	-0.862243	-0.693282	-2.523531
C	-2.295036	1.479957	0.165276
H	-1.674245	2.217078	-0.348127
C	-1.628908	1.191560	1.501043
C	-3.725589	2.062050	0.234304
H	-3.942741	2.446866	-0.773001
C	-3.842355	3.236072	1.223828
C	-2.911020	4.384489	0.828463
H	-1.856713	4.097484	0.856893

H	-3.040678	5.227912	1.513082
H	-3.142309	4.734234	-0.184894
H	-3.543281	2.868965	2.215285
C	-5.281843	3.752117	1.316508
H	-5.657151	4.035956	0.325562
H	-5.321104	4.639454	1.955114
H	-5.966036	3.012424	1.742725
C	-4.764486	0.979605	0.522975
H	-5.763965	1.354742	0.290195
H	-4.754009	0.675963	1.577423
C	-5.515888	-1.116923	-0.650546
C	-5.047837	-2.097402	-1.528712
S	-3.365905	-1.818187	-1.980487
C	-6.822273	-1.145875	-0.173549
H	-7.183050	-0.397502	0.523726
C	-7.654758	-2.174777	-0.610240
H	-8.677102	-2.215103	-0.249540
C	-7.193411	-3.150155	-1.495197
H	-7.858532	-3.942028	-1.822346
C	-5.880623	-3.119693	-1.962883
H	-5.515248	-3.876124	-2.649752
C	-0.472635	1.893372	1.848207
H	-0.071502	2.633086	1.158455
C	0.192433	1.627915	3.043796
H	1.093800	2.180189	3.292320
C	-0.289979	0.645014	3.904688
H	0.229178	0.429419	4.833626
C	-1.430741	-0.077857	3.557003
H	-1.798126	-0.862205	4.211453
C	-2.093504	0.191602	2.362292
H	-2.949186	-0.418506	2.082197
C	1.215535	-1.649470	-0.452461
H	1.355406	-2.239191	-1.368822

C	1.668966	-2.484676	0.731604
C	1.977337	-0.339731	-0.596119
C	3.451611	-0.398879	-0.595326
C	1.333711	0.822072	-0.856693
C	1.953647	2.170588	-0.860724
O	0.010065	0.908497	-1.127833
C	1.659076	-1.933903	2.017050
H	1.391395	-0.886132	2.144567
C	2.001916	-2.704507	3.120421
H	1.989518	-2.261908	4.112674
C	2.363897	-4.042879	2.954630
H	2.633280	-4.646335	3.816208
C	2.379820	-4.597356	1.679062
H	2.661958	-5.636797	1.539303
C	2.032400	-3.819491	0.573169
H	2.043568	-4.256643	-0.422594
S	4.317285	0.040710	-2.082186
C	5.814215	-0.356281	-1.295225
C	5.539649	-0.809416	0.011334
N	4.200987	-0.822658	0.368987
C	7.123074	-0.281900	-1.777296
H	7.328070	0.068736	-2.783465
C	8.153422	-0.671258	-0.933419
H	9.178123	-0.624148	-1.288189
C	7.891189	-1.125996	0.369509
H	8.717593	-1.423956	1.006735
C	6.593586	-1.197142	0.848634
H	6.372737	-1.545980	1.852237
C	2.924053	2.521699	0.085218
H	3.238611	1.791602	0.825829
C	3.470128	3.801152	0.085414
H	4.221318	4.065006	0.823507
C	3.045369	4.744370	-0.849160

H	3.471417	5.743004	-0.846188
C	2.064296	4.406115	-1.779355
H	1.725178	5.139415	-2.504581
C	1.514276	3.128306	-1.781238
H	0.746148	2.858063	-2.498698

Statistical Thermodynamic Analysis

Temperature= 298.150 Kelvin Pressure= 1.00000 Atm

===

SCF Energy= -2771.09926087 Predicted Change= -2.671274D-09

Zero-point correction (ZPE)= -2770.3909 0.70831

Internal Energy (U)= -2770.3499 0.74927

Enthalpy (H)= -2770.3490 0.75022

Gibbs Free Energy (G)= -2770.4684 0.63079

Frequencies -- -97.9503 9.1840 12.4138

===

#m062X/6-31+G(d,p) scf=(maxcycle=300,direct,tight) density=current

SCRF=(PCM,SOLVENT=THF)

Pointgroup= C1 Stoichiometry= C43H37N3O2S2 C1[X(C43H37N3O2S2)] #Atoms= 87

Charge = 0 Multiplicity = 1

SCF Energy= -2771.19777085

===

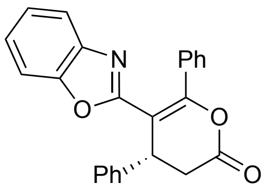

Benzoxazole-derived Lactone

Supporting Information: 0060-Oxazole-lactone.log

Using Gaussian 09: AM64L-G09RevC.01 23-Sep-2011

==

#M062X/6-31G(d) scf=(maxcycle=300,direct,tight) density=current

SCRF=(PCM,SOLVENT=THF) opt=(maxcycle=250) freq=noraman

#N Geom=AllCheck Guess=TCheck SCRF=Check GenChk RM062X/6-31G(d) Freq

----

Pointgroup= C1 Stoichiometry= C24H17NO3 C1[X(C24H17NO3)] #Atoms= 45

Charge = 0 Multiplicity = 1

SCF Energy= -1204.75433108 Predicted Change= -1.306763D-09

==

Optimization completed. {Found 2 times}

Item	Max Val.		Criteria	Pass?	RMS Val.		Criteria	Pass?
Force	0.00001	||	0.00045	[YES]	0.00000	||	0.00030	[YES]
Displ	0.00054	||	0.00180	[YES]	0.00054	||	0.00180	[YES]

Atomic Type	Coordinates (Angstroms) X	Y	Z
C	-2.589575	0.773368	-1.829101
H	-2.102299	1.138813	-2.741921
H	-3.590082	0.420501	-2.081052
C	-2.722687	1.943822	-0.897109
O	-3.718578	2.567497	-0.661771
O	-1.554008	2.345353	-0.301517
C	-0.397212	1.588829	-0.362925
C	0.774063	2.409898	0.004130
C	-0.442133	0.285279	-0.700415
C	0.673452	-0.641678	-0.553653
C	-1.729673	-0.330554	-1.198557
C	-2.481848	-1.078049	-0.107959
H	-1.471205	-1.047186	-1.983867
C	0.662704	3.352854	1.031382

H	-0.276951	3.454213	1.564794
C	1.752130	4.149855	1.367427
H	1.661848	4.873025	2.171598
C	2.952084	4.022581	0.670982
H	3.800244	4.648172	0.930903
C	3.060143	3.098804	-0.367837
H	3.987698	3.010264	-0.924293
C	1.975526	2.297156	-0.703133
H	2.052420	1.591847	-1.525767
C	-2.595355	-0.556351	1.183457
H	-2.091454	0.373062	1.440017
C	-3.338031	-1.225175	2.153093
H	-3.418065	-0.808050	3.152204
C	-3.971368	-2.426807	1.844300
H	-4.545648	-2.950413	2.602208
C	-3.859122	-2.955926	0.560309
H	-4.344414	-3.895299	0.313506
C	-3.118777	-2.283518	-0.408646
H	-3.029233	-2.699039	-1.409486
N	0.801674	-1.767950	-1.183286
C	1.972382	-2.319574	-0.669157
C	2.490296	-1.446757	0.289900
O	1.644517	-0.378115	0.367723
C	2.638971	-3.510765	-0.959523
H	2.251428	-4.200488	-1.701354
C	3.812177	-3.768240	-0.259980
H	4.359462	-4.683902	-0.458646
C	4.310765	-2.871669	0.701389
H	5.230002	-3.113756	1.224518
C	3.655963	-1.679868	1.000950
H	4.029831	-0.979078	1.738221

Statistical Thermodynamic Analysis

Temperature= 298.150 Kelvin   Pressure= 1.00000 Atm

==

SCF Energy= -1204.75433108 Predicted Change= -1.306763D-09

Zero-point correction (ZPE)= -1204.3978 0.35646

Internal Energy (U)= -1204.3767 0.37756

Enthalpy (H)= -1204.3758 0.37851

Gibbs Free Energy (G)= -1204.4504 0.30389

Frequencies -- 24.2780 29.4332 31.5152

==

#M062X/6-31+G(d,p) scf=(maxcycle=300,direct,tight) density=current

SCRF=(PCM,SOLVENT=THF)

Pointgroup= C1 Stoichiometry= C24H17NO3 C1[X(C24H17NO3)] #Atoms= 45

Charge = 0 Multiplicity = 1

SCF Energy= -1204.80942288

==

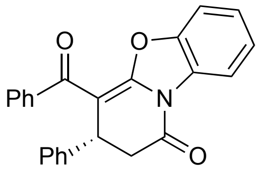

Benzoxazole-derived Lactam

Supporting Information: 0060-oxazole-lactam.log

Using Gaussian 09: AM64L-G09RevC.01 23-Sep-2011

==

#M062X/6-31G(d) scf=(maxcycle=300,direct,tight) density=current

SCRF=(PCM,SOLVENT=THF) opt=(maxcycle=250) freq=noraman

#N Geom=AllCheck Guess=TCheck SCRF=Check GenChk RM062X/6-31G(d) Freq

----

Pointgroup= C1 Stoichiometry= C24H17NO3 C1[X(C24H17NO3)] #Atoms= 45

Charge = 0 Multiplicity = 1

SCF Energy= -1204.75411750 Predicted Change= -2.869599D-09

===

Optimization completed. {Found 2 times}

Item	Max Val.	Criteria	Pass?	RMS Val.	Criteria	Pass?
Force	0.00000 ||	0.00045	[YES]	0.00000 ||	0.00030	[YES]
Displ	0.00153 ||	0.00180	[YES]	0.00153 ||	0.00180	[YES]

Atomic Type	Coordinates (Angstroms) X	Y	Z
C	-1.082643	-2.029825	-1.920840
H	-1.139171	-1.560841	-2.911989
H	-1.324483	-3.087739	-2.041793
C	-2.181494	-1.392733	-1.102130
O	-3.311724	-1.813595	-0.990093
N	-1.805267	-0.188731	-0.506147
C	-0.517812	0.353133	-0.605770
C	0.557056	-0.347297	-1.025964
C	1.901643	0.210436	-1.236679
C	2.375910	1.383537	-0.436922
C	0.321492	-1.817211	-1.351268
C	0.626545	-2.664874	-0.119842
H	1.042262	-2.078419	-2.131948
C	1.954269	-2.745446	0.321153
H	2.732911	-2.239853	-0.245046
C	2.285123	-3.467038	1.461683
H	3.320870	-3.520341	1.783393
C	1.291334	-4.125256	2.187447
H	1.547886	-4.690593	3.077887
C	-0.028130	-4.056962	1.756026
H	-0.810548	-4.569410	2.307528

C	-0.358952	-3.333288	0.608532
H	-1.398192	-3.311218	0.292402
O	2.664802	-0.315130	-2.040624
C	-2.660890	0.798548	0.018244
C	-1.864932	1.926962	0.176445
O	-0.570967	1.653491	-0.222967
C	-4.007023	0.816316	0.341847
H	-4.625381	-0.062342	0.216151
C	-4.512442	2.024489	0.835717
H	-5.561776	2.081649	1.104358
C	-3.703380	3.149762	0.992890
H	-4.131716	4.067390	1.381274
C	-2.343852	3.123456	0.660313
H	-1.698763	3.987000	0.771355
C	2.062427	1.510143	0.918316
H	1.413350	0.777226	1.390321
C	2.598626	2.556624	1.661618
H	2.364817	2.645651	2.717821
C	3.436815	3.487284	1.049552
H	3.847366	4.309438	1.627988
C	3.757037	3.359765	-0.301920
H	4.415117	4.081616	-0.775437
C	3.241304	2.299955	-1.039318
H	3.497250	2.170898	-2.086539

Statistical Thermodynamic Analysis

Temperature= 298.150 Kelvin Pressure= 1.00000 Atm

===

SCF Energy= -1204.75411750 Predicted Change= -2.869599D-09

Zero-point correction (ZPE)= -1204.3979 0.35619

Internal Energy (U)= -1204.3768 0.37730

Enthalpy (H)= -1204.3758 0.37824

Gibbs Free Energy (G)= -1204.4499 0.30417

Frequencies -- 19.2803 23.3982 40.1930

===

#M062X/6-31+G(d,p) scf=(maxcycle=300,direct,tight) density=current
SCRF=(PCM,SOLVENT=THF)

Pointgroup= C1 Stoichiometry= C24H17NO3 C1[X(C24H17NO3)] #Atoms= 45
Charge = 0 Multiplicity = 1

SCF Energy= -1204.80886451

===

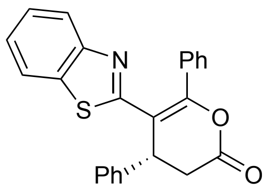

Benzothiazole-derived Lactone

Supporting Information: 0060-Thiazole-lactone.log

Using Gaussian 09: AM64L-G09RevC.01 23-Sep-2011

===

#M062X/6-31G(d) scf=(maxcycle=300,direct,tight) density=current
SCRF=(PCM,SOLVENT=THF) opt=(maxcycle=250) freq=noraman
#N Geom=AllCheck Guess=TCheck SCRF=Check GenChk RM062X/6-31G(d) Freq

----

Pointgroup= C1 Stoichiometry= C24H17NO2S C1[X(C24H17NO2S)] #Atoms= 45
Charge = 0 Multiplicity = 1

SCF Energy= -1527.71868312 Predicted Change= -6.264164D-09

===

Optimization completed. {Found 1 times}

Item	Max Val.		Criteria	Pass?	RMS Val.		Criteria	Pass?
Force	0.00001	||	0.00045	[YES]	0.00000	||	0.00030	[YES]

Displ 0.00327 || 0.00180 [NO] 0.00327 || 0.00180 [YES]

Atomic Type	Coordinates (Angstroms) X	Y	Z
C	-2.777661	-0.181369	-1.839703
H	-2.470788	0.323607	-2.764317
H	-3.561583	-0.901944	-2.075950
C	-3.359370	0.867942	-0.934722
O	-4.521396	1.058531	-0.709747
O	-2.442547	1.709628	-0.361177
C	-1.084098	1.448040	-0.406277
C	-0.306115	2.653015	-0.055540
C	-0.611325	0.224938	-0.708274
C	0.778576	-0.203401	-0.498402
C	-1.555140	-0.849783	-1.195689
C	-1.956795	-1.824736	-0.098789
H	-1.032111	-1.413664	-1.974035
C	-0.695599	3.449529	1.026261
H	-1.570441	3.174160	1.606992
C	0.044410	4.579733	1.358449
H	-0.252185	5.187725	2.207006
C	1.162999	4.929896	0.603666
H	1.737287	5.813528	0.863659
C	1.538627	4.152206	-0.490683
H	2.399353	4.431897	-1.089391
C	0.805960	3.018278	-0.821685
H	1.088538	2.411690	-1.677539
C	-2.124909	-1.416113	1.225827
H	-1.912167	-0.386995	1.507193
C	-2.549876	-2.321399	2.196514
H	-2.673968	-1.989621	3.222781
C	-2.809155	-3.645760	1.853258

H	-3.137422	-4.351267	2.610248
C	-2.641927	-4.061828	0.533729
H	-2.836877	-5.094053	0.259113
C	-2.218691	-3.155748	-0.433808
H	-2.086445	-3.482666	-1.462661
N	1.328331	-1.132145	-1.214755
C	2.586590	-1.456722	-0.737702
C	2.991641	-0.749883	0.411795
S	1.732885	0.353001	0.883855
C	3.444424	-2.406451	-1.307818
H	3.127340	-2.948554	-2.192672
C	4.679693	-2.627743	-0.721747
H	5.355218	-3.360305	-1.151278
C	5.073109	-1.914346	0.423024
H	6.046340	-2.104098	0.864218
C	4.238472	-0.968445	1.000399
H	4.543948	-0.416820	1.883137

Statistical Thermodynamic Analysis

Temperature= 298.150 Kelvin Pressure= 1.00000 Atm

==

SCF Energy= -1527.71868312 Predicted Change= -6.264164D-09

Zero-point correction (ZPE)= -1527.3655 0.35311

Internal Energy (U)= -1527.3438 0.37488

Enthalpy (H)= -1527.3428 0.37582

Gibbs Free Energy (G)= -1527.4200 0.29859

Frequencies -- 9.2935 20.4105 32.8593

==

#M062X/6-31+G(d,p) scf=(maxcycle=300,direct,tight) density=current SCRF=(PCM,SOLVENT=THF)

Pointgroup= C1 Stoichiometry= C24H17NO2S C1[X(C24H17NO2S)] #Atoms= 45

Charge = 0 Multiplicity = 1

SCF Energy= -1527.77296732

==

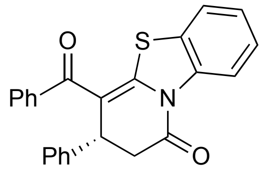

Benzothiazole-derived Lactam

Supporting Information: 0060-Thiazole-lactam.log

Using Gaussian 09: AM64L-G09RevC.01 23-Sep-2011

==

#M062X/6-31G(d) scf=(maxcycle=300,direct,tight) density=current SCRF=(PCM,SOLVENT=THF) opt=(maxcycle=250) freq=noraman

#N Geom=AllCheck Guess=TCheck SCRF=Check GenChk RM062X/6-31G(d) Freq

----

Pointgroup= C1 Stoichiometry= C24H17NO2S C1[X(C24H17NO2S)] #Atoms= 45

Charge = 0 Multiplicity = 1

SCF Energy= -1527.72434948 Predicted Change= -5.666021D-10

==

Optimization completed. {Found 2 times}

Item	Max Val.		Criteria	Pass?	RMS Val.		Criteria	Pass?
Force	0.00000	||	0.00045	[YES]	0.00000	||	0.00030	[YES]
Displ	0.00053	||	0.00180	[YES]	0.00053	||	0.00180	[YES]

Atomic Type	Coordinates (Angstroms) X	Y	Z
C	-0.205047	1.259479	2.030452
H	-0.348877	0.632773	2.921347

H	0.110973	2.250332	2.360489
C	-1.570174	1.404703	1.409830
O	-2.302954	2.346114	1.611881
N	-1.982775	0.309592	0.629726
C	-1.086049	-0.702338	0.256545
C	0.256175	-0.645128	0.493875
C	1.103986	-1.691575	-0.066479
C	2.594252	-1.583723	0.096071
C	0.842399	0.601220	1.125694
C	1.409201	1.550983	0.080839
H	1.670595	0.319328	1.778277
C	2.726707	1.998085	0.192629
H	3.342605	1.641085	1.015497
C	3.261802	2.878133	-0.746183
H	4.290393	3.211831	-0.648342
C	2.480228	3.322526	-1.809035
H	2.893801	4.008649	-2.541615
C	1.165635	2.875220	-1.933697
H	0.552663	3.209582	-2.765082
C	0.636411	1.991129	-0.998208
H	-0.383579	1.632790	-1.121475
O	0.638168	-2.631691	-0.710630
C	-3.317918	0.093499	0.206341
C	-3.445703	-1.126162	-0.461002
S	-1.913423	-1.983868	-0.612698
C	-4.430687	0.912542	0.396318
H	-4.339264	1.860781	0.903501
C	-5.659427	0.473258	-0.093194
H	-6.531775	1.102980	0.048001
C	-5.786386	-0.746612	-0.756978
H	-6.754023	-1.066280	-1.128881
C	-4.672590	-1.559075	-0.947229
H	-4.754773	-2.510459	-1.462893

C	3.384029	-1.525805	-1.053934
H	2.905361	-1.543549	-2.028764
C	4.767942	-1.429980	-0.945882
H	5.376843	-1.369339	-1.842467
C	5.370613	-1.419776	0.310857
H	6.450744	-1.349952	0.394725
C	4.587521	-1.510143	1.460212
H	5.055415	-1.523523	2.439682
C	3.201095	-1.584576	1.354182
H	2.591298	-1.662986	2.250925

Statistical Thermodynamic Analysis

Temperature= 298.150 Kelvin Pressure= 1.00000 Atm

===

SCF Energy= -1527.72434948 Predicted Change= -5.666021D-10

Zero-point correction (ZPE)= -1527.3714 0.35290

Internal Energy (U)= -1527.3496 0.37465

Enthalpy (H)= -1527.3487 0.37559

Gibbs Free Energy (G)= -1527.4245 0.29976

Frequencies -- 15.1501 28.5051 32.9279

===

#M062X/6-31+G(d,p) scf=(maxcycle=300,direct,tight) density=current SCRF=(PCM,SOLVENT=THF)

Pointgroup= C1 Stoichiometry= C24H17NO2S C1[X(C24H17NO2S)] #Atoms= 45

Charge = 0 Multiplicity = 1

SCF Energy= -1527.77826500

===

Annulation Facial Selectivity

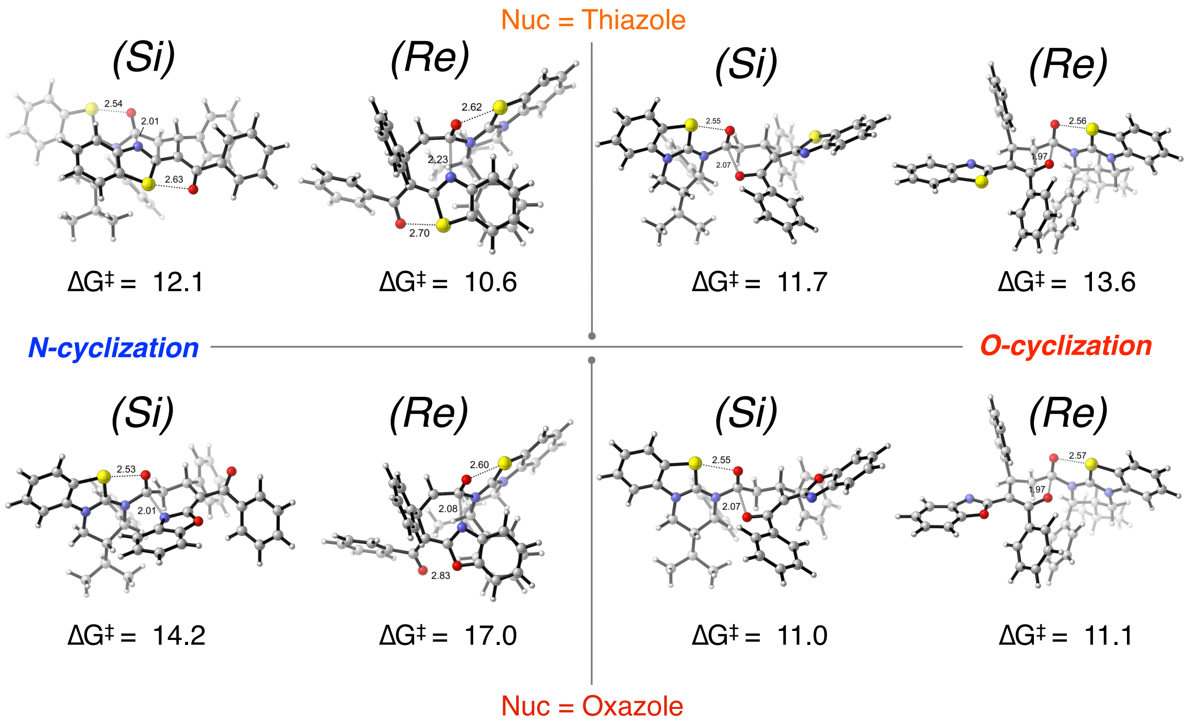

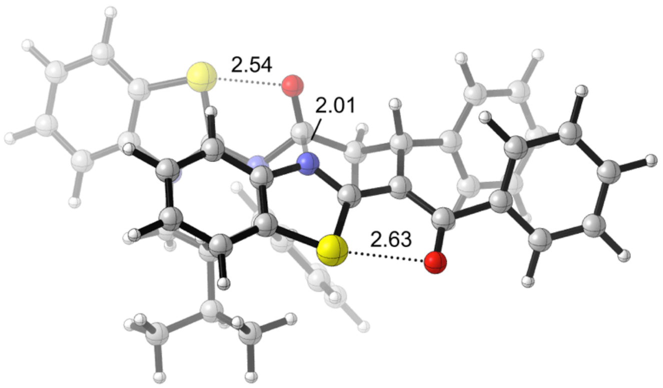

Supporting Information: 0045-Thiazole-Si-lactamization.log

Using Gaussian 09: AM64L-G09RevC.01 23-Sep-2011

==

#M062X/6-31G(d) scf=(maxcycle=300,direct,tight) density=current

SCRF=(PCM,SOLVENT=THF) opt=(maxcycle=250,ts,calcfc,noeigentest,gdiis)

iop(1/8=18) freq=noraman

#N Geom=AllCheck Guess=TCheck SCRF=Check Test GenChk RM062X/6-31G(d) Freq

----

Pointgroup= C1 Stoichiometry= C43H37N3O2S2 C1[X(C43H37N3O2S2)] #Atoms= 87

Charge = 0 Multiplicity = 1

SCF Energy= -2771.10098022 Predicted Change= -8.792690D-09

===

Optimization completed. {Found 2 times}

Item	Max Val.	Criteria	Pass?	RMS Val.	Criteria	Pass?
Force	0.00001 ||	0.00045	[YES]	0.00000 ||	0.00030	[YES]
Displ	0.00137 ||	0.00180	[YES]	0.00137 ||	0.00180	[YES]

Atomic Type	Coordinates (Angstroms) X	Y	Z
N	-3.748571	-0.549885	-0.023574
C	-2.644009	-0.778056	-0.753038
N	-1.424062	-0.497364	-0.304507
C	-0.249171	-0.699797	-1.204663
C	0.999476	-1.183726	-0.493699
H	0.973869	-1.008946	0.582542
H	0.986430	-2.268304	-0.647790
O	-0.516222	-1.060929	-2.345822
C	-1.259316	0.037666	1.067536
H	-0.363950	0.662634	1.025517
C	-1.038155	-1.077047	2.078149
C	-2.453091	0.959570	1.406469
H	-2.451160	1.767641	0.661821
C	-2.357760	1.603522	2.802620
C	-1.154972	2.544377	2.897758
H	-1.261193	3.362353	2.175619
H	-0.201100	2.050631	2.694993
H	-1.095519	2.978824	3.900084
H	-2.245637	0.801637	3.544487

C	-3.631522	2.391187	3.129342
H	-3.837338	3.129211	2.343800
H	-3.503450	2.931834	4.071410
H	-4.509869	1.748419	3.239323
C	-3.750103	0.180917	1.246350
H	-4.602288	0.862454	1.211318
H	-3.902056	-0.532271	2.066698
C	-4.933602	-1.030595	-0.596869
C	-4.707504	-1.611691	-1.841721
S	-3.005941	-1.544509	-2.286148
C	-6.208664	-0.973483	-0.040606
H	-6.381586	-0.535091	0.935840
C	-7.256867	-1.510938	-0.779598
H	-8.260704	-1.482026	-0.369880
C	-7.037532	-2.087216	-2.035188
H	-7.872967	-2.499621	-2.590546
C	-5.759466	-2.141978	-2.581309
H	-5.585360	-2.590133	-3.553857
C	-1.770362	-2.266850	2.047541
H	-2.496506	-2.451588	1.259884
C	-1.566767	-3.247601	3.016045
H	-2.143218	-4.166392	2.977241
C	-0.624222	-3.054900	4.022425
H	-0.465095	-3.821166	4.774236
C	0.123458	-1.879908	4.050080
H	0.873996	-1.727166	4.818951
C	-0.080260	-0.901204	3.081645
H	0.523680	0.003000	3.095837
C	2.326375	-0.602282	-1.055356
H	2.219835	-0.587923	-2.150737
C	3.403239	-1.607805	-0.692175
C	2.524109	0.812686	-0.518624
C	1.389420	1.620422	-0.610432

C	3.711018	1.416280	0.025206
C	5.088177	0.895526	-0.284865
O	3.659973	2.455417	0.711397
C	3.805886	-1.780257	0.634975
H	3.378869	-1.145400	1.409391
C	4.771140	-2.724904	0.967354
H	5.086877	-2.833037	2.000938
C	5.337759	-3.526265	-0.023381
H	6.096218	-4.259386	0.234458
C	4.921114	-3.382812	-1.343813
H	5.351666	-4.006022	-2.122224
C	3.957710	-2.431301	-1.671802
H	3.645215	-2.310887	-2.706774
S	1.235266	3.251919	0.095668
C	-0.378041	3.375278	-0.591740
C	-0.689607	2.205489	-1.308079
N	0.288017	1.234879	-1.273273
C	-1.298152	4.413267	-0.476599
H	-1.054153	5.309178	0.087059
C	-2.539894	4.279771	-1.097152
H	-3.270931	5.077099	-1.010489
C	-2.836978	3.138352	-1.852008
H	-3.795119	3.063197	-2.357485
C	-1.916850	2.102798	-1.975421
H	-2.124607	1.228760	-2.585614
C	5.440264	0.445019	-1.557251
H	4.686416	0.405186	-2.339801
C	6.747955	0.048952	-1.825820
H	7.014185	-0.297953	-2.819892
C	7.711872	0.096283	-0.821215
H	8.729664	-0.220698	-1.028810
C	7.369762	0.561505	0.448499
H	8.120774	0.609125	1.231624

C	6.067509	0.973244	0.708386
H	5.788949	1.355978	1.685660

Statistical Thermodynamic Analysis

Temperature= 298.150 Kelvin Pressure= 1.00000 Atm

==

SCF Energy= -2771.10098022 Predicted Change= -8.792690D-09

Zero-point correction (ZPE)= -2770.3914 0.70952

Internal Energy (U)= -2770.3512 0.74968

Enthalpy (H)= -2770.3503 0.75062

Gibbs Free Energy (G)= -2770.4630 0.63796

Frequencies -- -128.6508 22.9958 26.9458

==

#M062X/6-31+G(d,p) scf=(maxcycle=300,direct,tight) density=current SCRF=(PCM,SOLVENT=THF)

Pointgroup= C1 Stoichiometry= C43H37N3O2S2 C1[X(C43H37N3O2S2)] #Atoms= 87

Charge = 0 Multiplicity = 1

SCF Energy= -2771.19995224

==

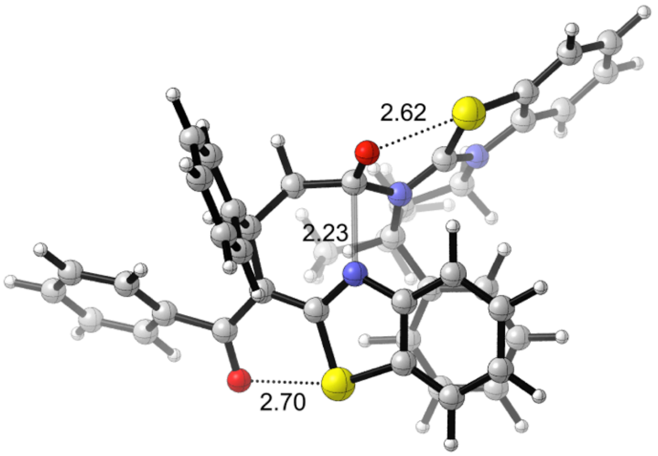

Supporting Information: Favored-Lactamization-(X=S)-TS.log

Using Gaussian 09: AM64L-G09RevC.01 23-Sep-2011

==

#m062X/6-31G(d) scf=(maxcycle=300,direct,tight) density=current

SCRF=(PCM,SOLVENT=THF) opt=(maxcycle=250,ts,calcfc,noeigentest)

freq=noraman

#N Geom=AllCheck Guess=TCheck SCRF=Check GenChk RM062X/6-31G(d) Freq

----

Pointgroup= C1 Stoichiometry= C43H37N3O2S2 C1[X(C43H37N3O2S2)] #Atoms= 87

Charge = 0 Multiplicity = 1

SCF Energy= -2771.09894699 Predicted Change= -7.886391D-09

==
============

Optimization completed. {Found 2 times}

Item	Max Val.		Criteria	Pass?	RMS Val.		Criteria	Pass?
Force	0.00001	||	0.00045	[YES]	0.00000	||	0.00030	[YES]
Displ	0.00147	||	0.00180	[YES]	0.00147	||	0.00180	[YES]

Atomic Type	Coordinates (Angstroms) X	Y	Z
N	3.439401	-0.200867	-1.301812

C	2.494968	0.694232	-0.977469
N	1.239225	0.326889	-0.716260
C	0.167113	1.331735	-0.824088
C	-1.053806	0.866445	-1.604821
O	0.495080	2.495688	-0.729143
C	0.958492	-1.115858	-0.564327
C	1.333109	-1.675636	0.799009
C	1.660485	-1.841160	-1.735568
C	1.304714	-3.332224	-1.859813
C	-0.175026	-3.505277	-2.215387
C	2.167447	-4.025786	-2.918963
C	3.160865	-1.617240	-1.587952
C	4.716376	0.350412	-1.470172
C	4.711641	1.733692	-1.300476
S	3.103275	2.326886	-0.907782
C	5.883904	-0.340846	-1.782987
C	7.049699	0.401630	-1.928242
C	7.049387	1.791762	-1.767251
C	5.879251	2.474497	-1.453852
C	2.439556	-1.261014	1.548379
C	2.749953	-1.886581	2.751736
C	1.965838	-2.937961	3.222506
C	0.856183	-3.347589	2.491883
C	0.540802	-2.713390	1.293414
C	-2.450765	0.877494	-0.926988
C	-2.871412	2.309814	-0.576898
C	-2.604133	-0.135002	0.186453
C	-1.676104	-0.035467	1.242805
C	-3.635303	-1.110865	0.270031
C	-4.745172	-1.160358	-0.753096
O	-3.681788	-1.971624	1.176994
C	-3.192432	2.710912	0.721228
C	-3.607826	4.015662	0.984630

C	-3.718064	4.942905	-0.047320
C	-3.417248	4.551550	-1.350709
C	-3.000947	3.249232	-1.607407
S	-1.795055	-0.908651	2.791779
C	-0.371135	-0.050557	3.350924
C	0.089347	0.821381	2.343763
N	-0.651461	0.800491	1.186338
C	0.301212	-0.161634	4.563862
C	1.433831	0.621326	4.774487
C	1.886259	1.502168	3.783541
C	1.221887	1.613798	2.568427
C	-5.445244	-0.024973	-1.171936
C	-6.516643	-0.142655	-2.055681
C	-6.893926	-1.394911	-2.535035
C	-6.208240	-2.533662	-2.112177
C	-5.151607	-2.415762	-1.215765
H	-1.075630	1.565711	-2.447085
H	-0.891044	-0.123963	-2.035601
H	-0.122096	-1.219283	-0.659303
H	1.332116	-1.340900	-2.659099
H	-0.386076	-3.048052	-3.189602
H	-0.852469	-3.056710	-1.481708
H	-0.424998	-4.567938	-2.282903
H	1.498445	-3.812361	-0.892305
H	3.224710	-4.060325	-2.639566
H	2.082095	-3.515989	-3.886346
H	1.832170	-5.057901	-3.054975
H	3.685041	-1.854646	-2.515981
H	3.578946	-2.223467	-0.775344
H	5.888040	-1.418504	-1.902975
H	7.974910	-0.110227	-2.169408
H	7.973971	2.345941	-1.886433
H	5.875231	3.551782	-1.327750

H	3.051922	-0.423250	1.227795
H	3.600746	-1.539286	3.329493
H	2.209828	-3.421297	4.163601
H	0.217693	-4.144322	2.860375
H	-0.352262	-3.012887	0.749025
H	-3.111753	0.562390	-1.742047
H	-3.125486	1.994175	1.533191
H	-3.848216	4.304323	2.003742
H	-4.042908	5.958325	0.158861
H	-3.513875	5.259045	-2.169162
H	-2.784889	2.954682	-2.632731
H	-0.049658	-0.845306	5.331707
H	1.966917	0.549594	5.717588
H	2.766210	2.110970	3.971090
H	1.553567	2.300550	1.794056
H	-5.164445	0.953186	-0.787784
H	-7.060536	0.745200	-2.364310
H	-7.724743	-1.485165	-3.228456
H	-6.505708	-3.513243	-2.474794
H	-4.627490	-3.296152	-0.855653

Statistical Thermodynamic Analysis

Temperature= 298.150 Kelvin Pressure= 1.00000 Atm

===

SCF Energy= -2771.09894699 Predicted Change= -7.886391D-09

Zero-point correction (ZPE)= -2770.3903 0.70860

Internal Energy (U)= -2770.3496 0.74928

Enthalpy (H)= -2770.3487 0.75022

Gibbs Free Energy (G)= -2770.4648 0.63406

Frequencies -- -75.4058 11.9587 16.3033

===

#m062X/6-31+G(d,p) scf=(maxcycle=300,direct,tight) density=current

SCRF=(PCM,SOLVENT=THF)

Pointgroup= C1 Stoichiometry= C43H37N3O2S2 C1[X(C43H37N3O2S2)] #Atoms= 87

Charge = 0 Multiplicity = 1

SCF Energy= -2771.19836373

==

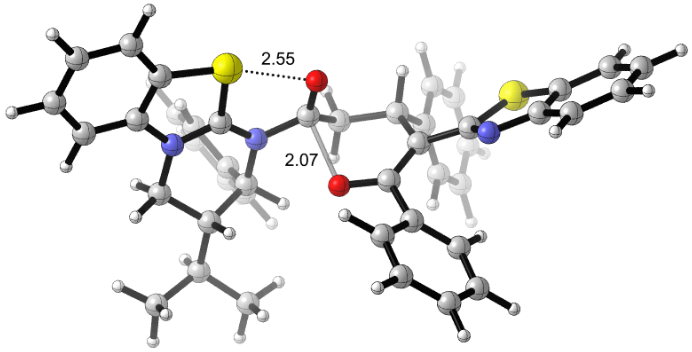

Supporting Information: Disfavored-Lactonization-(X=S)-TS.log

----

Using Gaussian 09: AM64L-G09RevC.01 23-Sep-2011

==

#M062X/6-31G(d) scf=(maxcycle=300,direct,tight) density=current

SCRF=(PCM,SOLVENT=THF) opt=(maxcycle=250,ts,calcfc,noeigentest,gdiis)

iop(1/8=18) freq=noraman

#N Geom=AllCheck Guess=TCheck SCRF=Check Test GenChk RM062X/6-31G(d) Freq

----

Pointgroup= C1 Stoichiometry= C43H37N3O2S2 C1[X(C43H37N3O2S2)] #Atoms= 87

Charge = 0 Multiplicity = 1

SCF Energy= -2771.09554176 Predicted Change= -1.272284D-08

==

Optimization completed. {Found 1 times}

Item	Max Val.	Criteria	Pass?	RMS Val.	Criteria	Pass?
Force	0.00002 ||	0.00045	[YES]	0.00000 ||	0.00030	[YES]

Displ 0.00204 || 0.00180 [NO] 0.00204 || 0.00180 [YES]

Atomic Type	Coordinates (Angstroms) X	Y	Z
N	4.074300	-1.024251	-0.331106
C	2.839139	-0.899864	-0.843806
N	1.995443	0.045774	-0.434420
C	0.636476	0.079602	-0.965785
C	-0.108433	1.386253	-0.876725
H	0.115962	1.931159	0.038682
H	0.214677	2.015284	-1.717332
O	0.335433	-0.779481	-1.775372
C	2.376387	0.957337	0.665577
H	1.448015	1.122602	1.210977
C	2.915371	2.267062	0.120861
C	3.325564	0.215233	1.626477
H	2.765711	-0.661729	1.982817
C	3.727027	1.058035	2.850463
C	2.511586	1.342688	3.737191
H	2.808645	1.942381	4.602207
H	2.082774	0.404134	4.106639
H	1.718914	1.886183	3.216766
H	4.136477	2.011063	2.490244
C	4.807221	0.356929	3.681444
H	4.488063	-0.654603	3.960337
H	4.989106	0.915631	4.603877
H	5.761617	0.283410	3.151560
C	4.533114	-0.287844	0.851732
H	5.109918	-0.993732	1.451663
H	5.191064	0.531917	0.537367
C	4.862549	-1.999923	-0.956198
C	4.164882	-2.662327	-1.962989

S	2.520001	-2.057446	-2.112993
C	6.185518	-2.314688	-0.657149
H	6.732247	-1.785804	0.115352
C	6.787194	-3.327233	-1.394923
H	7.817471	-3.592808	-1.184360
C	6.088814	-4.003773	-2.401380
H	6.581790	-4.791594	-2.960400
C	4.769498	-3.678291	-2.696947
H	4.225814	-4.198751	-3.478228
C	3.757665	2.307961	-0.993961
H	4.009793	1.394472	-1.527404
C	4.267582	3.520419	-1.452066
H	4.917261	3.534777	-2.321363
C	3.937024	4.708026	-0.804498
H	4.331244	5.652955	-1.164520
C	3.086143	4.678563	0.297968
H	2.809988	5.600305	0.800077
C	2.575861	3.465992	0.753451
H	1.899734	3.454151	1.604913
C	-1.630103	1.153086	-0.993168
H	-1.828036	0.886363	-2.040466
C	-2.288410	2.507078	-0.737334
C	-2.167031	0.038285	-0.104814
C	-3.495644	-0.440265	-0.397687
C	-1.363479	-0.588500	0.846988
C	-1.914580	-1.455555	1.944102
O	-0.089053	-0.444122	0.902443
C	-2.590574	2.923017	0.561722
H	-2.406908	2.239224	1.387782
C	-3.139855	4.180858	0.795188
H	-3.376599	4.486560	1.810208
C	-3.394275	5.042732	-0.270567
H	-3.829954	6.020971	-0.090966

C	-3.092558	4.638594	-1.569366
H	-3.292083	5.301559	-2.406203
C	-2.541552	3.379594	-1.797879
H	-2.315508	3.061189	-2.813734
S	-4.672115	0.646040	-1.233966
C	-5.837642	-0.647611	-1.221763
C	-5.272320	-1.773033	-0.585437
N	-3.967709	-1.634922	-0.167585
C	-7.137877	-0.668033	-1.723187
H	-7.559520	0.204436	-2.212815
C	-7.885504	-1.830953	-1.574197
H	-8.902096	-1.866185	-1.953066
C	-7.338369	-2.955736	-0.940012
H	-7.938604	-3.854548	-0.835000
C	-6.041418	-2.937120	-0.447886
H	-5.607215	-3.802938	0.042080
C	-1.185276	-2.582990	2.325793
H	-0.282055	-2.828962	1.776184
C	-1.609508	-3.376035	3.388724
H	-1.042960	-4.260630	3.664825
C	-2.754326	-3.028826	4.102189
H	-3.082718	-3.639402	4.938129
C	-3.472035	-1.886255	3.745254
H	-4.356656	-1.603091	4.308044
C	-3.055816	-1.106291	2.671745
H	-3.614996	-0.218043	2.388809

Statistical Thermodynamic Analysis

Temperature= 298.150 Kelvin   Pressure= 1.00000 Atm

==

SCF Energy=   -2771.09554176   Predicted Change= -1.272284D-08

Zero-point correction (ZPE)=   -2770.3865 0.70897

Internal Energy (U)=   -2770.3455 0.74998

Enthalpy (H)= -2770.3446 0.75093

Gibbs Free Energy (G)= -2770.4636 0.63190

Frequencies -- -116.2652 12.0058 17.8171

==

#M062X/6-31+G(d,p) scf=(maxcycle=300,direct,tight) density=current
SCRF=(PCM,SOLVENT=THF)

Pointgroup= C1 Stoichiometry= C43H37N3O2S2 C1[X(C43H37N3O2S2)] #Atoms= 87
Charge = 0 Multiplicity = 1

SCF Energy= -2771.19453032

==

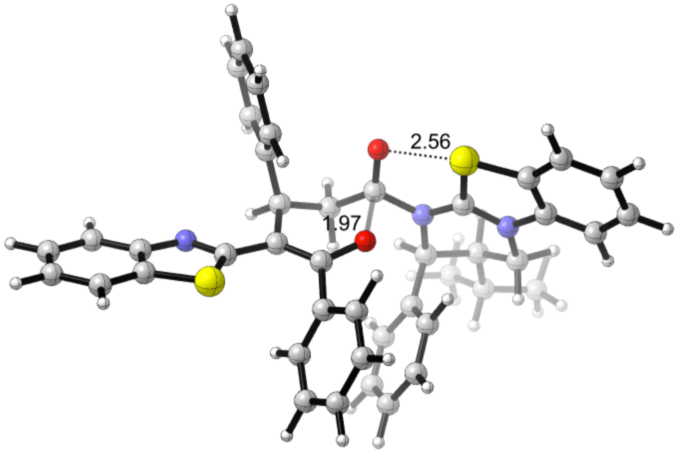

Supporting Information: 0045-Thiazole-Re-lactonization.log

Using Gaussian 09: AM64L-G09RevC.01 23-Sep-2011

==

#m062X/6-31G(d) scf=(maxcycle=300,direct,tight) density=current
SCRF=(PCM,SOLVENT=THF) opt=(maxcycle=250,ts,calcfc,noeigentest)
freq=noraman
#N Geom=AllCheck Guess=TCheck SCRF=Check GenChk RM062X/6-31G(d) Freq

----

Pointgroup= C1 Stoichiometry= C43H37N3O2S2 C1[X(C43H37N3O2S2)] #Atoms= 87

Charge = 0 Multiplicity = 1

SCF Energy= -2771.09517293 Predicted Change= -6.643546D-11

===

Optimization completed. {Found 2 times}

Item	Max Val.	Criteria	Pass?	RMS Val.	Criteria	Pass?
Force	0.00000 ||	0.00045	[YES]	0.00000 ||	0.00030	[YES]
Displ	0.00035 ||	0.00180	[YES]	0.00035 ||	0.00180	[YES]

Atomic Type	Coordinates (Angstroms) X	Y	Z
N	-4.094409	0.507724	-0.236259
C	-2.892005	1.100848	-0.138715
N	-2.034820	0.829848	0.838657
C	-0.688120	1.458725	0.755201
C	0.304928	1.034589	1.811310
H	0.163770	1.698885	2.673542
H	0.130483	0.004951	2.130478
O	-0.657012	2.544253	0.200238
C	-2.364187	-0.221403	1.823392
H	-1.860649	0.075047	2.745311
C	-1.869226	-1.598354	1.409922
C	-3.884716	-0.166150	2.099984
H	-4.102152	0.854364	2.447160
C	-4.343554	-1.146578	3.195707
C	-3.743930	-0.772656	4.553836
H	-4.064284	0.233785	4.848082
H	-2.650937	-0.791821	4.560207
H	-4.087236	-1.472249	5.321240
H	-3.999260	-2.151934	2.920484
C	-5.871439	-1.179106	3.309762

H	-6.167227	-1.796251	4.162786
H	-6.348227	-1.601519	2.420600
H	-6.272614	-0.171156	3.471127
C	-4.639926	-0.378193	0.794940
H	-5.692321	-0.112087	0.912048
H	-4.573337	-1.417482	0.449753
C	-4.809153	0.838163	-1.395024
C	-4.117691	1.756200	-2.182249
S	-2.584548	2.212929	-1.450904
C	-6.051001	0.339352	-1.779716
H	-6.580082	-0.390609	-1.177085
C	-6.585958	0.800615	-2.977859
H	-7.550084	0.426209	-3.304501
C	-5.901663	1.730863	-3.767050
H	-6.341337	2.074346	-4.697197
C	-4.659362	2.219576	-3.376543
H	-4.122755	2.938888	-3.986297
C	-1.328195	-2.439256	2.385075
H	-1.209776	-2.076328	3.403571
C	-0.933151	-3.737697	2.067775
H	-0.512994	-4.376192	2.838576
C	-1.065320	-4.203545	0.763569
H	-0.743230	-5.207899	0.506081
C	-1.582094	-3.363851	-0.221067
H	-1.640062	-3.703923	-1.250956
C	-1.980086	-2.070216	0.097962
H	-2.325926	-1.414042	-0.695066
C	1.768964	1.117295	1.331202
H	2.364417	0.855724	2.214025
C	2.204458	2.527670	0.946388
C	2.070207	0.063855	0.284067
C	3.471818	-0.238756	0.135580
C	1.044424	-0.416819	-0.514921

C	1.157703	-1.667072	-1.334604
O	-0.125791	0.117340	-0.566609
C	2.393069	2.905620	-0.383385
H	2.216543	2.173355	-1.166865
C	2.795826	4.198411	-0.706110
H	2.937979	4.474267	-1.747267
C	3.018020	5.137168	0.299986
H	3.334274	6.145185	0.048375
C	2.836977	4.769670	1.631436
H	3.015083	5.490221	2.424661
C	2.437493	3.473209	1.947618
H	2.310507	3.187664	2.990711
S	4.178185	-0.939073	-1.362570
C	5.771985	-0.710671	-0.692088
C	5.656062	-0.126396	0.585983
N	4.369487	0.129846	1.011793
C	7.011653	-1.036314	-1.237052
H	7.087210	-1.490723	-2.220064
C	8.153769	-0.756287	-0.493460
H	9.130417	-0.992999	-0.903859
C	8.054469	-0.170128	0.775919
H	8.957890	0.041502	1.339673
C	6.817312	0.144897	1.320833
H	6.727909	0.598337	2.303055
C	0.586362	-1.702030	-2.609476
H	0.142172	-0.795424	-3.009650
C	0.583781	-2.881080	-3.348621
H	0.151852	-2.896286	-4.345003
C	1.129198	-4.045155	-2.805977
H	1.120748	-4.967713	-3.378917
C	1.676411	-4.023322	-1.524525
H	2.084074	-4.931800	-1.090896
C	1.691551	-2.839202	-0.793219

H 2.097619 -2.819578 0.214562

Statistical Thermodynamic Analysis

Temperature= 298.150 Kelvin Pressure= 1.00000 Atm

===

SCF Energy= -2771.09517293 Predicted Change= -6.643546D-11

Zero-point correction (ZPE)= -2770.3866 0.70847

Internal Energy (U)= -2770.3460 0.74913

Enthalpy (H)= -2770.3450 0.75008

Gibbs Free Energy (G)= -2770.4617 0.63346

Frequencies -- -134.0813 14.8830 20.3018

===

#m062X/6-31+G(d,p) scf=(maxcycle=300,direct,tight) density=current SCRF=(PCM,SOLVENT=THF)

Pointgroup= C1 Stoichiometry= C43H37N3O2S2 C1[X(C43H37N3O2S2)] #Atoms= 87

Charge = 0 Multiplicity = 1

SCF Energy= -2771.19297595

===

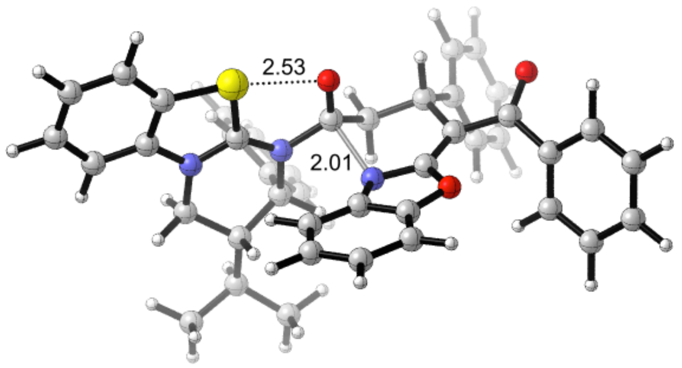

Supporting Information: Disfavored-Lactamization-(X=O)-TS.log

----

Using Gaussian 09: AM64L-G09RevD.01 24-Apr-2013

==

#m062X/6-31G(d) scf=(maxcycle=300,direct,tight) density=current
SCRF=(PCM,SOLVENT=THF) opt=(maxcycle=250,ts,calcfc,noeigentest)
freq=noraman
#N Geom=AllCheck Guess=TCheck SCRF=Check GenChk RM062X/6-31G(d) Freq

----

Pointgroup= C1 Stoichiometry= C43H37N3O3S C1[X(C43H37N3O3S)] #Atoms= 87
Charge = 0 Multiplicity = 1

SCF Energy= -2448.12448628 Predicted Change= -9.033427D-09

==

Optimization completed on the basis of negligible forces. {Found 2 times}

Item	Max Val.	Criteria	Pass?	RMS Val.	Criteria	Pass?
Force	0.00000 ||	0.00045	[YES]	0.00000 ||	0.00030	[YES]
Displ	0.01384 ||	0.00180	[NO]	0.01384 ||	0.00180	[NO]

Atomic Type	Coordinates (Angstroms) X	Y	Z
N	3.772491	-0.328835	-0.150724
C	2.589181	-0.346402	-0.787701
N	1.511136	0.255587	-0.295704
C	0.235380	0.192891	-1.048362
C	-0.715372	1.344411	-0.786366
O	0.279029	-0.369762	-2.134793
C	1.575896	0.986362	0.988353
C	1.784588	2.472575	0.748464
C	2.637333	0.344477	1.906234
C	2.769861	1.049536	3.269578
C	1.484312	0.900336	4.088074
C	3.946516	0.487836	4.074174
C	3.968689	0.268575	1.173519
C	4.810961	-0.970572	-0.841431

C	4.365022	-1.530304	-2.035760
S	2.651062	-1.228262	-2.295915
C	6.142006	-1.068039	-0.444034
C	7.012596	-1.759145	-1.279312
C	6.568527	-2.332656	-2.475490
C	5.239285	-2.221744	-2.868278
C	2.684931	2.935528	-0.215117
C	2.887589	4.301756	-0.395793
C	2.188358	5.221786	0.380605
C	1.277527	4.769367	1.332549
C	1.074962	3.403937	1.510662
C	-2.135330	1.070111	-1.305039
C	-2.921956	2.356957	-1.090001
C	-2.798176	-0.141270	-0.669072
C	-4.168472	-0.364122	-1.068733
C	-5.086463	-1.261410	-0.277544
O	-4.658524	0.231955	-2.036973
C	-2.018620	-1.106243	-0.046643
C	-3.499110	2.650737	0.147160
C	-4.146769	3.864883	0.361685
C	-4.222003	4.808883	-0.660907
C	-3.646134	4.526553	-1.897733
C	-3.000377	3.310071	-2.106481
O	-2.524143	-2.354627	0.245778
C	-1.460271	-3.113145	0.650775
C	-0.313915	-2.308423	0.614264
N	-0.704150	-1.031831	0.242176
C	-1.466698	-4.439073	1.021636
C	-0.224177	-4.996193	1.352531
C	0.939937	-4.229704	1.298070
C	0.917291	-2.879424	0.931455
C	-5.048500	-1.315645	1.117679
C	-5.976128	-2.080628	1.818075

C	-6.944973	-2.807949	1.127697
C	-6.992415	-2.753585	-0.264519
C	-6.076060	-1.970954	-0.960568
H	-0.769428	1.605505	0.270630
H	-0.303040	2.212921	-1.317675
H	0.602784	0.812158	1.452996
H	2.303815	-0.683681	2.101279
H	1.281581	-0.158391	4.287633
H	0.605995	1.318730	3.590073
H	1.589830	1.409673	5.050140
H	2.955913	2.115782	3.087420
H	3.951840	0.923946	5.077106
H	4.913899	0.712541	3.615821
H	3.859516	-0.600209	4.183025
H	4.661248	-0.382017	1.709552
H	4.429318	1.257501	1.059176
H	6.495910	-0.616048	0.475812
H	8.055452	-1.849394	-0.995254
H	7.268820	-2.867147	-3.108112
H	4.890917	-2.659224	-3.797932
H	3.223324	2.235771	-0.850216
H	3.587776	4.645368	-1.150540
H	2.343065	6.286226	0.235995
H	0.713357	5.478348	1.929983
H	0.345145	3.061195	2.239990
H	-2.067429	0.905667	-2.389380
H	-3.448730	1.908176	0.940872
H	-4.596034	4.073061	1.328670
H	-4.728658	5.755125	-0.496529
H	-3.704749	5.252460	-2.703751
H	-2.555377	3.091127	-3.074914
H	-2.383952	-5.016961	1.039301
H	-0.169588	-6.039802	1.643826

H	1.891698	-4.690661	1.543439
H	1.841099	-2.313456	0.883962
H	-4.288315	-0.750957	1.651010
H	-5.945287	-2.109509	2.903261
H	-7.665044	-3.409849	1.674261
H	-7.749154	-3.314035	-0.805493
H	-6.114708	-1.898352	-2.043276

Statistical Thermodynamic Analysis

Temperature= 298.150 Kelvin Pressure= 1.00000 Atm

==

SCF Energy= -2448.12448628 Predicted Change= -9.033427D-09

Zero-point correction (ZPE)= -2447.4128 0.71158

Internal Energy (U)= -2447.3724 0.75204

Enthalpy (H)= -2447.3715 0.75298

Gibbs Free Energy (G)= -2447.4892 0.63522

Frequencies -- -138.4921 4.8783 17.7797

==

#m062X/6-31+G(d,p) scf=(maxcycle=300,direct,tight) density=current SCRF=(PCM,SOLVENT=THF)

Pointgroup= C1 Stoichiometry= C43H37N3O3S C1[X(C43H37N3O3S)] #Atoms= 87

Charge = 0 Multiplicity = 1

SCF Energy= -2448.22560001

==

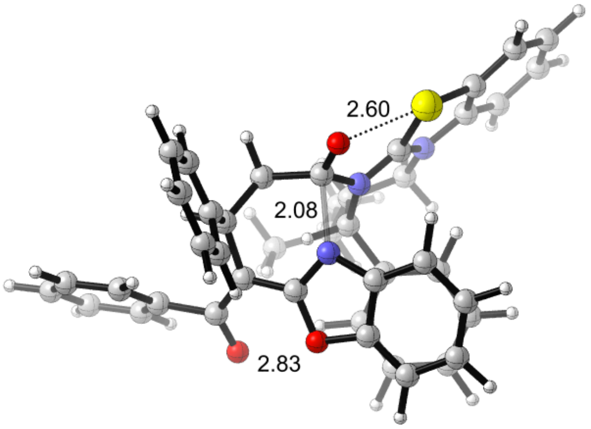

Supporting Information: 0045-Oxazole-Re-lactamization.log

Using Gaussian 09: AM64L-G09RevC.01 23-Sep-2011

==

#m062X/6-31G(d) scf=(maxcycle=300,direct,tight) density=current

SCRF=(PCM,SOLVENT=THF) opt=(maxcycle=250,ts,calcfc,noeigentest)

freq=noraman

#N Geom=AllCheck Guess=TCheck SCRF=Check GenChk RM062X/6-31G(d) Freq

---

Pointgroup= C1 Stoichiometry= C43H37N3O3S C1[X(C43H37N3O3S)] #Atoms= 87

Charge = 0 Multiplicity = 1

SCF Energy= -2448.12651398 Predicted Change= -6.734049D-09

==

Optimization completed. {Found 2 times}

Item	Max Val.	Criteria	Pass?	RMS Val.	Criteria	Pass?
Force	0.00000 ||	0.00045	[YES]	0.00000 ||	0.00030	[YES]
Displ	0.00174 ||	0.00180	[YES]	0.00174 ||	0.00180	[YES]

Atomic Type	Coordinates (Angstroms) X	Y	Z
N	-3.347510	0.666871	-1.100170
C	-2.457810	-0.339839	-1.112200

N	-1.192300	-0.162750	-0.748040
C	-0.167980	-1.158510	-1.167890
C	1.064480	-0.506921	-1.800630
O	-0.591531	-2.246910	-1.514180
C	-0.821579	1.120150	-0.118110
C	-1.152249	1.182770	1.365000
C	-1.474739	2.248060	-0.944060
C	-1.021838	3.659190	-0.533570
C	0.483612	3.833080	-0.764340
C	-1.789778	4.732141	-1.312080
C	-2.986819	2.074141	-0.867860
C	-4.653420	0.281512	-1.430630
C	-4.732780	-1.074808	-1.741080
S	-3.164221	-1.861749	-1.598040
C	-5.776119	1.104042	-1.472600
C	-6.984550	0.523953	-1.841660
C	-7.068840	-0.835167	-2.161350
C	-5.942031	-1.649748	-2.117550
C	-2.297350	0.610011	1.929010
C	-2.575730	0.771511	3.283190
C	-1.715539	1.508821	4.092910
C	-0.559489	2.056830	3.545930
C	-0.274879	1.888900	2.192790
C	2.452960	-0.615691	-1.108550
C	3.004319	-2.044992	-1.192700
C	2.477540	0.012119	0.270220
C	1.554310	-0.568341	1.155670
C	3.220461	1.176368	0.631220
C	4.361051	1.623738	-0.254180
O	2.978371	1.881618	1.630830
C	3.433209	-2.757812	-0.071260
C	3.966428	-4.040342	-0.195370
C	4.090948	-4.633542	-1.447900

C	3.685818	-3.926892	-2.578650
C	3.151479	-2.648852	-2.447630
O	1.575010	-0.411911	2.515080
C	0.542590	-1.183890	2.985080
C	-0.066621	-1.808900	1.890980
N	0.603149	-1.401390	0.753260
C	0.116999	-1.344600	4.287370
C	-0.983121	-2.187940	4.474010
C	-1.608181	-2.821080	3.393310
C	-1.161671	-2.646910	2.082520
C	5.276940	0.725737	-0.810800
C	6.351421	1.191707	-1.565060
C	6.514771	2.559317	-1.777390
C	5.610322	3.461647	-1.217350
C	4.548771	2.994728	-0.449050
H	1.116120	-0.988211	-2.781050
H	0.866250	0.552179	-1.989170
H	0.261521	1.197070	-0.208620
H	-1.168679	2.089930	-1.989430
H	0.731322	3.656859	-1.818610
H	1.095741	3.159469	-0.155550
H	0.784602	4.855649	-0.517510
H	-1.233498	3.790010	0.535690
H	-2.857158	4.738711	-1.071530
H	-1.681098	4.579941	-2.392840
H	-1.395077	5.723660	-1.072740
H	-3.486959	2.656381	-1.644420
H	-3.375199	2.375891	0.112700
H	-5.716189	2.157192	-1.221030
H	-7.876109	1.140353	-1.878840
H	-8.024311	-1.262267	-2.446100
H	-6.002531	-2.704268	-2.364720
H	-2.972900	0.004521	1.330990

H	-3.461710	0.307861	3.705660
H	-1.932559	1.631481	5.149800
H	0.139551	2.600300	4.174330
H	0.671111	2.256879	1.799520
H	3.094280	-0.009442	-1.756670
H	3.359259	-2.302542	0.911260
H	4.285918	-4.574852	0.694560
H	4.507218	-5.631642	-1.544880
H	3.791698	-4.368312	-3.565350
H	2.857609	-2.106591	-3.343700
H	0.603560	-0.836000	5.112300
H	-1.359431	-2.351470	5.478800
H	-2.459802	-3.467879	3.582020
H	-1.639961	-3.138779	1.241500
H	5.153030	-0.341563	-0.638330
H	7.063330	0.486687	-1.983750
H	7.348821	2.921267	-2.371280
H	5.739232	4.528497	-1.374870
H	3.850482	3.685328	0.015180

Statistical Thermodynamic Analysis

Temperature= 298.150 Kelvin Pressure= 1.00000 Atm

==

SCF Energy= -2448.12651398 Predicted Change= -6.734049D-09

Zero-point correction (ZPE)= -2447.4135 0.71291

Internal Energy (U)= -2447.3737 0.75273

Enthalpy (H)= -2447.3728 0.75367

Gibbs Free Energy (G)= -2447.4856 0.64088

Frequencies -- -111.3969 17.1657 23.9791

==

#m062X/6-31+G(d,p) scf=(maxcycle=300,direct,tight) density=current

SCRF=(PCM,SOLVENT=THF)

Pointgroup= C1 Stoichiometry= C43H37N3O3S C1[X(C43H37N3O3S)] #Atoms= 87

Charge = 0 Multiplicity = 1

SCF Energy= -2448.22673263

==

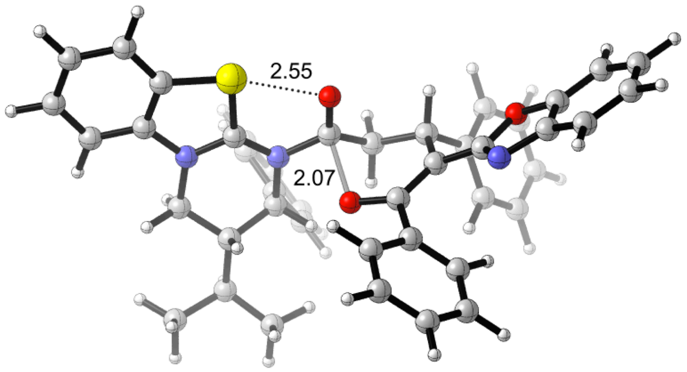

Supporting Information: Favored-Lactonization-(X=O)-TS.log

Using Gaussian 09: AM64L-G09RevC.01 23-Sep-2011

==

#M062X/6-31G(d) scf=(maxcycle=300,direct,tight) density=current

SCRF=(PCM,SOLVENT=THF) opt=(maxcycle=250,ts,calcfc,noeigentest,gdiis)

iop(1/8=18) freq=noraman

#N Geom=AllCheck Guess=TCheck SCRF=Check Test GenChk RM062X/6-31G(d) Freq

----

Pointgroup= C1 Stoichiometry= C43H37N3O3S C1[X(C43H37N3O3S)] #Atoms= 87

Charge = 0 Multiplicity = 1

SCF Energy= -2448.13243384 Predicted Change= -5.035723D-09

==

Optimization completed. {Found 2 times}

Item	Max Val.	Criteria	Pass?	RMS Val.	Criteria	Pass?
Force	0.00001 ||	0.00045	[YES]	0.00000 ||	0.00030	[YES]
Displ	0.00126 ||	0.00180	[YES]	0.00126 ||	0.00180	[YES]

Atomic Type	Coordinates (Angstroms) X	Y	Z
N	4.052756	-0.838226	-0.399409
C	2.798714	-0.797481	-0.877352
N	1.884654	0.043053	-0.396124
C	0.523573	0.008070	-0.922693
C	-0.299090	1.261840	-0.778163
H	-0.123983	1.767026	0.170791
H	0.003145	1.953843	-1.576131
O	0.280659	-0.829213	-1.774089
C	2.209732	0.925558	0.744575
H	1.283656	0.975699	1.316677
C	2.611495	2.308335	0.264978
C	3.250943	0.226012	1.641077
H	2.785571	-0.715571	1.967387
C	3.612743	1.041401	2.895338
C	2.404643	1.173475	3.827026
H	2.673714	1.758536	4.710990
H	2.073979	0.184128	4.163481
H	1.550259	1.665209	3.354814
H	3.925371	2.043383	2.573104
C	4.774896	0.402261	3.663362
H	5.714700	0.432866	3.104067
H	4.551295	-0.643398	3.907139
H	4.938191	0.936405	4.603676
C	4.475956	-0.125680	0.810568
H	5.130055	-0.806722	1.357550
H	5.049012	0.765203	0.526162
C	4.908718	-1.699686	-1.099276
C	4.245138	-2.365580	-2.126719
S	2.548294	-1.906212	-2.203284

C	6.263526	-1.906422	-0.852798
H	6.783893	-1.374702	-0.064040
C	6.932406	-2.814793	-1.664947
H	7.988583	-2.995024	-1.496807
C	6.268984	-3.494703	-2.692487
H	6.815516	-4.198490	-3.310753
C	4.917054	-3.277238	-2.934967
H	4.399360	-3.799801	-3.732392
C	2.168873	3.431549	0.968912
H	1.511809	3.305750	1.826234
C	2.549118	4.711577	0.576094
H	2.194246	5.572952	1.133043
C	3.371361	4.885111	-0.534609
H	3.665008	5.882619	-0.845466
C	3.803778	3.773196	-1.252750
H	4.433083	3.899622	-2.128139
C	3.423982	2.492734	-0.857039
H	3.753217	1.639069	-1.445103
C	-1.796528	0.940829	-0.939537
H	-1.951319	0.660804	-1.989288
C	-2.549034	2.246609	-0.702194
C	-2.279853	-0.198055	-0.051278
C	-3.601844	-0.678101	-0.343963
C	-1.445232	-0.824121	0.873718
C	-1.957741	-1.718474	1.966133
O	-0.172952	-0.658949	0.904838
C	-2.869319	3.082829	-1.773116
H	-2.627161	2.765613	-2.785470
C	-3.503007	4.305229	-1.560461
H	-3.752065	4.938722	-2.406929
C	-3.822874	4.710621	-0.266330
H	-4.320022	5.661155	-0.098039
C	-3.503519	3.884297	0.809866

H	-3.751675	4.189871	1.822356
C	-2.871021	2.663003	0.591376
H	-2.632041	2.011207	1.428983
O	-4.311794	0.097152	-1.248816
C	-5.494703	-0.553464	-1.427596
C	-5.460441	-1.701111	-0.628000
N	-4.244642	-1.755676	0.033577
C	-6.560676	-0.200562	-2.233809
H	-6.546603	0.699129	-2.839034
C	-7.652784	-1.071467	-2.217495
H	-8.520825	-0.844627	-2.828119
C	-7.647512	-2.229040	-1.426651
H	-8.515749	-2.880812	-1.441204
C	-6.558310	-2.563014	-0.622989
H	-6.556415	-3.460121	-0.012521
C	-3.064643	-1.368089	2.743069
H	-3.627524	-0.473695	2.490999
C	-3.443693	-2.155457	3.824572
H	-4.301726	-1.870691	4.426232
C	-2.723662	-3.308939	4.136924
H	-3.023103	-3.926341	4.978696
C	-1.613922	-3.660517	3.371338
H	-1.046666	-4.554444	3.613500
C	-1.224622	-2.858338	2.301625
H	-0.345779	-3.106160	1.714306

Statistical Thermodynamic Analysis

Temperature= 298.150 Kelvin   Pressure= 1.00000 Atm

==

SCF Energy=   -2448.13243384   Predicted Change= -5.035723D-09

Zero-point correction (ZPE=)   -2447.4195 0.71289

Internal Energy (U)=   -2447.3792 0.75322

Enthalpy (H)=   -2447.3782 0.75416

Gibbs Free Energy (G)= -2447.4954 0.63699

Frequencies -- -107.7350 11.6842 19.6170

==

#M062X/6-31+G(d,p) scf=(maxcycle=300,direct,tight) density=current SCRF=(PCM,SOLVENT=THF)

Pointgroup= C1 Stoichiometry= C43H37N3O3S C1[X(C43H37N3O3S)] #Atoms= 87

Charge = 0 Multiplicity = 1

SCF Energy= -2448.23239713

==

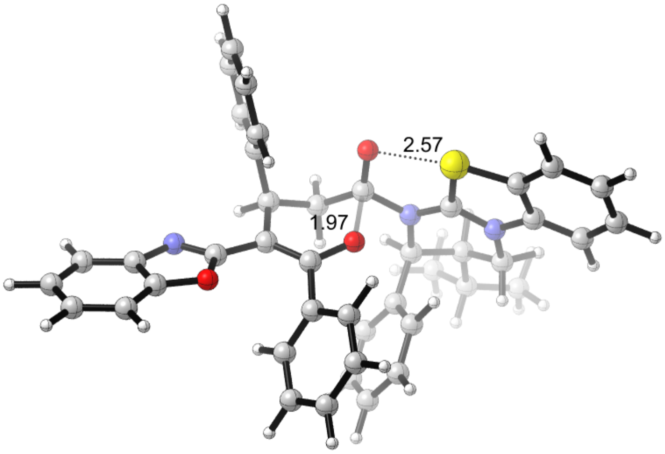

Supporting Information: 0045-Oxazole-Re-lactonization.log

Using Gaussian 09: AM64L-G09RevD.01 24-Apr-2013

==

#m062X/6-31G(d) scf=(maxcycle=300,direct,tight) density=current SCRF=(PCM,SOLVENT=THF) opt=(maxcycle=250,ts,calcfc,noeigentest,gdiis) iop(1/8=18) freq=noraman

#N Geom=AllCheck Guess=TCheck SCRF=Check Test GenChk RM062X/6-31G(d) Freq

----

Pointgroup= C1 Stoichiometry= C43H37N3O3S C1[X(C43H37N3O3S)] #Atoms= 87

Charge = 0 Multiplicity = 1

SCF Energy= -2448.13205596 Predicted Change= -6.026860D-10

==

Optimization completed. {Found 2 times}

Item	Max Val.	Criteria	Pass?	RMS Val.	Criteria	Pass?
Force	0.00000 ||	0.00045	[YES]	0.00000 ||	0.00030	[YES]
Displ	0.00119 ||	0.00180	[YES]	0.00119 ||	0.00180	[YES]

Atomic Type	Coordinates (Angstroms) X	Y	Z
N	-3.971463	0.363666	-0.290541
C	-2.802136	1.019910	-0.196139
N	-1.941961	0.816634	0.794783
C	-0.625654	1.506326	0.707051
C	0.372011	1.148724	1.783433
O	-0.636363	2.579997	0.129267
C	-2.228860	-0.219133	1.810083
C	-1.639909	-1.574915	1.456203
C	-3.753920	-0.247741	2.061507
C	-4.170927	-1.230614	3.171827
C	-3.613161	-0.793989	4.529234
C	-5.696036	-1.348714	3.266619
C	-4.476751	-0.531775	0.752197
C	-4.696440	0.639305	-1.457147
C	-4.048219	1.577876	-2.256835
S	-2.543624	2.123060	-1.526136
C	-5.910361	0.072837	-1.837151
C	-6.463314	0.487518	-3.043912

C	-5.822303	1.436736	-3.846732
C	-4.606981	1.992431	-3.461189
C	-1.031812	-2.328781	2.462811
C	-0.535755	-3.603717	2.197948
C	-0.634786	-4.133961	0.915318
C	-1.221144	-3.381174	-0.099956
C	-1.717418	-2.109564	0.166283
C	1.836035	1.286192	1.319382
C	2.219116	2.713676	0.939055
C	2.184069	0.234516	0.289221
C	3.582243	-0.068699	0.201020
C	1.198336	-0.310752	-0.519855
C	1.376113	-1.574072	-1.310524
O	0.006644	0.169744	-0.592510
C	2.326652	3.682268	1.940507
C	2.679833	4.993937	1.635606
C	2.939507	5.356335	0.315267
C	2.843930	4.395968	-0.689469
C	2.487021	3.086192	-0.377968
O	4.077280	-0.678610	-0.931849
C	5.422392	-0.770453	-0.726987
C	5.695488	-0.185165	0.513934
N	4.504128	0.250831	1.075733
C	6.384654	-1.323718	-1.551084
C	7.697533	-1.267375	-1.077452
C	8.001399	-0.684963	0.160875
C	7.011323	-0.136865	0.974646
C	0.726413	-1.696232	-2.541155
C	0.766887	-2.895155	-3.247364
C	1.436940	-3.995743	-2.714442
C	2.068459	-3.887679	-1.475953
C	2.038255	-2.683648	-0.779648
H	0.188717	1.822859	2.629729

H	0.239989	0.119631	2.122180
H	-1.763504	0.140192	2.729897
H	-4.036768	0.765207	2.382733
H	-2.521125	-0.751936	4.549652
H	-3.927563	-1.494747	5.307902
H	-3.993314	0.199168	4.796065
H	-3.764734	-2.219682	2.922855
H	-5.968609	-1.963404	4.129081
H	-6.135711	-1.816590	2.381059
H	-6.155939	-0.361984	3.400096
H	-5.544318	-0.326197	0.850985
H	-4.343893	-1.572350	0.431006
H	-6.404422	-0.672663	-1.223850
H	-7.407361	0.061822	-3.366585
H	-6.275365	1.742831	-4.783518
H	-4.104357	2.726427	-4.082157
H	-0.935254	-1.914459	3.463995
H	-0.061790	-4.171939	2.992178
H	-0.233197	-5.119087	0.698208
H	-1.256830	-3.769088	-1.113724
H	-2.116709	-1.515937	-0.651267
H	2.429962	1.052028	2.212347
H	2.138541	3.402627	2.975916
H	2.761921	5.731188	2.429257
H	3.219690	6.377211	0.073035
H	3.048635	4.666933	-1.721413
H	2.408479	2.337953	-1.161894
H	6.131936	-1.771547	-2.505806
H	8.496992	-1.683108	-1.682329
H	9.034810	-0.661730	0.492931
H	7.247451	0.312490	1.933751
H	0.181983	-0.841276	-2.930863
H	0.270342	-2.974578	-4.210195

H	1.461457	-4.935072	-3.259123
H	2.575108	-4.746945	-1.046114
H	2.504983	-2.607083	0.198685

Statistical Thermodynamic Analysis

Temperature= 298.150 Kelvin Pressure= 1.00000 Atm

==

SCF Energy= -2448.13205596 Predicted Change= -6.026860D-10

Zero-point correction (ZPE)= -2447.4207 0.71131

Internal Energy (U)= -2447.3804 0.75156

Enthalpy (H)= -2447.3795 0.75250

Gibbs Free Energy (G)= -2447.4961 0.63595

Frequencies -- -133.8467 11.0549 16.1845

==

#m062X/6-31+G(d,p) scf=(maxcycle=300,direct,tight) density=current SCRF=(PCM,SOLVENT=THF)

Pointgroup= C1 Stoichiometry= C43H37N3O3S C1[X(C43H37N3O3S)] #Atoms= 87

Charge = 0 Multiplicity = 1

SCF Energy= -2448.23128315

==

Computed Model Systems

Anionic Nucleophile Dihedral Rotation Energies

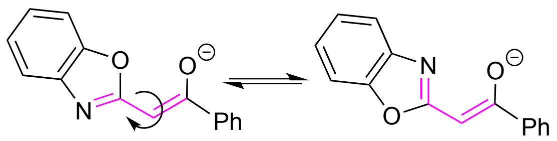

Anionic Benzoxazole

Supporting Information: oxazole-Ph.log

Using Gaussian 09: AM64L-G09RevD.01 24-Apr-2013

===

#m062X/6-31G(d) scf=(maxcycle=300,direct,tight) density=current

SCRF=(PCM,SOLVENT=THF) opt=(maxcycle=250,modredundant,gdiis) iop(1/8=18)

Modredundant Input: D 13 8 9 10 S 18 10.000

Pointgroup= C1 Stoichiometry= C15H10NO2(1-) C1[X(C15H10NO2)] #Atoms= 28

Charge = -1 Multiplicity = 1

Optimization completed. {Found 19 times}

Item	Max Val.	Criteria	Pass?	RMS Val.	Criteria	Pass?
Force	0.00001 ||	0.00045	[YES]	0.00000 ||	0.00030	[YES]
Displ	0.00125 ||	0.00180	[YES]	0.00125 ||	0.00180	[YES]

Dihedral = 0

SCF = -782.6216

XYZ =

28

000-oxazole-Ph.com

C 5.38346300 -0.48949700 -0.06830700

C	4.31539700	-1.37683100	-0.21348000
C	3.01985300	-0.86114200	-0.13640900
C	2.84760600	0.51448700	0.08161600
C	3.88736900	1.41048000	0.22884900
C	5.18013200	0.87787500	0.14941500
N	1.78264200	-1.46189900	-0.23824000
C	0.91005000	-0.47577700	-0.08622600
C	-0.49185000	-0.58288800	-0.10499100
C	-1.40001900	0.48647600	0.03602200
C	-2.87817200	0.12770400	0.02450500
O	-1.11515600	1.69815900	0.14333900
O	1.50637300	0.75859600	0.11222400
C	-3.37845200	-1.13339700	0.36457300
C	-4.74882000	-1.38425700	0.34683200
C	-5.64190200	-0.37757300	-0.01480600
C	-5.15477400	0.88614700	-0.34630100
C	-3.78611700	1.13438500	-0.31690700
H	6.39932700	-0.86995800	-0.12569100
H	4.47989900	-2.43656200	-0.38217400
H	3.70785400	2.46720300	0.39625000
H	6.03481500	1.53825300	0.25723000
H	-0.85535000	-1.58777500	-0.27184400
H	-2.69650200	-1.92106000	0.67054800
H	-5.12004800	-2.36703700	0.62354700

H	-6.70989400	-0.57469900	-0.03108100
H	-5.84410400	1.67855700	-0.62476400
H	-3.38681600	2.11517700	-0.55515000

Dihedral = 10

SCF = -782.62138

XYZ =

28

010-oxazole-Ph.com

C	5.38210300	-0.48161200	-0.12051800
C	4.31336100	-1.36138000	-0.30215800
C	3.01817400	-0.85498800	-0.17293800
C	2.84768400	0.50447300	0.13071000
C	3.88803600	1.39322800	0.31332400
C	5.18023600	0.86982000	0.18174300
N	1.77990300	-1.45065100	-0.29413700
C	0.90876700	-0.47749000	-0.06955200
C	-0.49307300	-0.58842400	-0.04113900
C	-1.39964600	0.49062500	0.02026100
C	-2.87782500	0.13227300	0.02063800
O	-1.11343400	1.70631400	0.05130200
O	1.50711200	0.74195100	0.20102200
C	-3.37810900	-1.10973700	0.42543400
C	-4.74768400	-1.36448400	0.41210700
C	-5.64012400	-0.38034000	-0.00875700

C	-5.15316000	0.86515600	-0.40360600
C	-3.78510500	1.11763400	-0.37946000
H	6.39744200	-0.85545600	-0.21693200
H	4.47723300	-2.40844600	-0.53746000
H	3.70906000	2.43761500	0.54601300
H	6.03539200	1.52461200	0.31661300
H	-0.85872900	-1.59359200	-0.20224800
H	-2.69577700	-1.87741500	0.77765900
H	-5.11894200	-2.33181200	0.73861700
H	-6.70761400	-0.58043700	-0.02175800
H	-5.84236400	1.63977000	-0.72857000
H	-3.38625400	2.08506200	-0.66810600

Dihedral = 20

SCF = -782.6205

XYZ =

28

020-oxazole-Ph.com

C	5.37705900	-0.48392600	-0.19442000
C	4.30045800	-1.34943500	-0.39650600
C	3.01119100	-0.84686100	-0.20794700
C	2.85407600	0.49444700	0.17294000
C	3.90227400	1.36995200	0.37449300
C	5.18826400	0.85022900	0.18377700
N	1.76689400	-1.43118600	-0.33092500

C	0.90642200	-0.47030800	-0.03058300
C	-0.49435300	-0.58364100	0.05953100
C	-1.40103100	0.49745800	0.04011800
C	-2.87849000	0.13766600	0.02881100
O	-1.11572200	1.71348200	0.01145700
O	1.51732600	0.72958000	0.29496100
C	-3.38664300	-1.08028400	0.49291100
C	-4.75489500	-1.33973200	0.45979500
C	-5.63779300	-0.38430400	-0.04068900
C	-5.14312000	0.83789000	-0.49456300
C	-3.77660300	1.09597000	-0.45011400
H	6.38819600	-0.85539100	-0.33423600
H	4.45379300	-2.38299900	-0.69119500
H	3.73376200	2.40108000	0.66661600
H	6.04940900	1.49395700	0.33324500
H	-0.86140500	-1.58958000	-0.09568900
H	-2.71077600	-1.82372000	0.90455400
H	-5.13311300	-2.28787500	0.83145300
H	-6.70423200	-0.58831300	-0.06979400
H	-5.82570100	1.58965900	-0.88110800
H	-3.37192500	2.04626100	-0.78404500

Dihedral = 30

SCF = -782.619

XYZ =

28

030-oxazole-Ph.com

C	5.37129000	-0.47270500	-0.25744900
C	4.28912800	-1.32099100	-0.49789800
C	3.00580500	-0.83226300	-0.24564100
C	2.85818700	0.47833300	0.23263200
C	3.91239800	1.33702400	0.47369100
C	5.19237900	0.83120700	0.21935100
N	1.75686900	-1.40436600	-0.38937100
C	0.90471400	-0.46707100	-0.00901700
C	-0.49566500	-0.58421600	0.11844400
C	-1.39986700	0.49485100	0.01738800
C	-2.87789100	0.13888900	0.01876800
O	-1.11028300	1.70604600	-0.08963700
O	1.52516500	0.70365300	0.39783300
C	-3.39142300	-1.04496800	0.55856200
C	-4.76045300	-1.30177200	0.53712100
C	-5.63842500	-0.37763600	-0.02694500
C	-5.13805200	0.81067500	-0.55786600
C	-3.77076800	1.06654000	-0.52552300
H	6.37877900	-0.83262100	-0.44556100
H	4.43328600	-2.33001600	-0.87135600
H	3.75312000	2.34458200	0.84279200
H	6.05799400	1.46149700	0.39759800

H -0.86221700 -1.59246400 -0.02670200

H -2.71845800 -1.76187900 1.01935400

H -5.14356300 -2.22326900 0.96645700

H -6.70552800 -0.57940400 -0.04581500

H -5.81704200 1.53880600 -0.99301300

H -3.36165500 1.99236700 -0.91745500

Dihedral = 40

SCF = -782.616915

XYZ =

28

040-oxazole-Ph.com

C 5.35703800 -0.47077200 -0.33597800

C 4.26306400 -1.29581400 -0.60128700

C 2.99089200 -0.81710800 -0.28332900

C 2.86471500 0.46036500 0.28203900

C 3.93127300 1.29669500 0.54870200

C 5.19972000 0.80094900 0.22862400

N 1.73290600 -1.37116800 -0.43175400

C 0.89880600 -0.45730600 0.02996500

C -0.49893400 -0.57413500 0.21222300

C -1.39729600 0.49908200 0.02903900

C -2.87547400 0.14680300 0.02482600

O -1.09894200 1.70084200 -0.14630400

O 1.53922900 0.67931300 0.49980500

C	-3.40095300	-0.99722800	0.63402600
C	-4.76896600	-1.25748300	0.59812200
C	-5.63329700	-0.37730800	-0.05073400
C	-5.12083200	0.77162200	-0.65180800
C	-3.75478800	1.03215700	-0.60414500
H	6.35670900	-0.82259800	-0.57398400
H	4.39052600	-2.27973300	-1.04173700
H	3.78892700	2.27951700	0.98519000
H	6.07452800	1.41307100	0.42437400
H	-0.86836500	-1.58402300	0.08137400
H	-2.73790400	-1.67804400	1.15981000
H	-5.16218500	-2.14716300	1.08204500
H	-6.69952500	-0.58231200	-0.08136800
H	-5.78906000	1.46576400	-1.15415900
H	-3.33610500	1.92933300	-1.04898000

Dihedral = 50

SCF = -782.61415

XYZ =

28

050-oxazole-Ph.com

C	5.34781000	-0.47943600	-0.39924400
C	4.23627900	-1.27024500	-0.69259800
C	2.97915200	-0.79457400	-0.31646600
C	2.88336500	0.44374800	0.33356700

C	3.96842100	1.24722100	0.62885300
C	5.22131200	0.75524000	0.24986000
N	1.70840600	-1.31858000	-0.47657500
C	0.89783400	-0.42742800	0.05825900
C	-0.49986400	-0.54297700	0.27949400
C	-1.40268400	0.50781200	0.01054800
C	-2.87962500	0.14978000	0.01791700
O	-1.10874400	1.69722100	-0.24307400
O	1.56720100	0.66369700	0.59603700
C	-3.40398000	-0.96468700	0.68060800
C	-4.77105500	-1.23079400	0.65254200
C	-5.63597800	-0.38615000	-0.04131200
C	-5.12467400	0.73344900	-0.69624800
C	-3.75942800	0.99987700	-0.65723200
H	6.33670200	-0.82842400	-0.68182500
H	4.33908700	-2.22571600	-1.19745400
H	3.85017100	2.20274300	1.12841600
H	6.10939100	1.34129600	0.46452200
H	-0.86368700	-1.55842900	0.17121800
H	-2.74100000	-1.61599100	1.24260000
H	-5.16316700	-2.09679300	1.17864900
H	-6.70152100	-0.59560500	-0.06512200
H	-5.79267500	1.39977400	-1.23528200
H	-3.34111100	1.87484600	-1.14469000

Dihedral = 60

SCF = -782.6108

XYZ =

28

060-oxazole-Ph.com

C	5.33398600	-0.48715400	-0.46445100
C	4.20289300	-1.23808300	-0.78426100
C	2.96330500	-0.76630400	-0.35016000
C	2.90046100	0.42790900	0.37982000
C	4.00656600	1.19324400	0.70183600
C	5.24147200	0.70578800	0.26468900
N	1.67937700	-1.25934200	-0.51606000
C	0.89559400	-0.39686700	0.09247300
C	-0.50075600	-0.51405800	0.35823400
C	-1.40462400	0.50647100	0.00016400
C	-2.88190300	0.15019700	0.01457000
O	-1.10613600	1.67701100	-0.32969900
O	1.59487900	0.64661600	0.68809800
C	-3.41189800	-0.93509200	0.71991300
C	-4.77892900	-1.20150900	0.69327200
C	-5.63870000	-0.38594100	-0.04090600
C	-5.12194000	0.70491100	-0.73849400
C	-3.75654900	0.97130300	-0.70196600
H	6.31062200	-0.83307400	-0.78978600
H	4.27596600	-2.16001400	-1.35270300

H	3.91763400	2.11562100	1.26555800
H	6.14398400	1.26229200	0.49699600
H	-0.85555300	-1.53678500	0.28772900
H	-2.75372000	-1.56295600	1.31325800
H	-5.17530200	-2.04464300	1.25236000
H	-6.70426700	-0.59551900	-0.06305700
H	-5.78526900	1.34868500	-1.30970600
H	-3.33354600	1.82386700	-1.22380300

Dihedral = 70

SCF = -782.6071

XYZ =

28

070-oxazole-Ph.com

C	5.32750100	-0.44251300	-0.52024900
C	4.18636500	-1.13663900	-0.91954900
C	2.95685900	-0.71094200	-0.41543200
C	2.91154600	0.38302100	0.45708300
C	4.02953900	1.09094200	0.86279600
C	5.25363400	0.64986600	0.35538400
N	1.66393800	-1.16824700	-0.62649700
C	0.89602800	-0.38341300	0.08893400
C	-0.50688400	-0.50801800	0.35870500
C	-1.40409300	0.48987400	-0.06030600
C	-2.88382000	0.14751200	-0.00665400

O	-1.09822700	1.63515900	-0.47213900
O	1.61204800	0.57232000	0.80277100
C	-3.40943100	-0.88865200	0.77228500
C	-4.77811200	-1.14648300	0.78479400
C	-5.64539200	-0.36947800	0.01811400
C	-5.13322200	0.67240300	-0.75379100
C	-3.76522700	0.92937000	-0.75773600
H	6.29770700	-0.75372200	-0.89578600
H	4.24420100	-1.98071100	-1.59956600
H	3.95586900	1.93588000	1.53877200
H	6.16436200	1.16454800	0.64522000
H	-0.85714900	-1.53482400	0.33442400
H	-2.74438000	-1.48343600	1.39148400
H	-5.16998500	-1.95056000	1.40160100
H	-6.71274600	-0.57085600	0.02799100
H	-5.80215800	1.28640600	-1.35067200
H	-3.34558600	1.74408300	-1.33931100

Dihedral = 80

SCF = -782.60355

XYZ =

28

080-oxazole-Ph.com

C	5.30826200	-0.35600600	-0.62413100
C	4.15250800	-0.96710700	-1.10537100

C	2.94090700	-0.62787800	-0.50243700
C	2.92575000	0.29817500	0.54591300
C	4.06052200	0.92247700	1.03734200
C	5.26520800	0.57108100	0.42796800
N	1.63630400	-1.03436500	-0.75553100
C	0.89507800	-0.38268100	0.10010700
C	-0.51444700	-0.52357900	0.37560500
C	-1.39920700	0.47166100	-0.05786600
C	-2.88260000	0.14696700	-0.00521100
O	-1.07751700	1.61114300	-0.48305500
O	1.63740300	0.43135800	0.95020300
C	-3.42305800	-0.86926200	0.78973800
C	-4.79404800	-1.11450300	0.79831600
C	-5.64932300	-0.34458100	0.01105200
C	-5.12257700	0.67810400	-0.77657000
C	-3.75222400	0.92288900	-0.77594700
H	6.26623900	-0.59951800	-1.07366300
H	4.18586300	-1.68106700	-1.92226400
H	4.01074000	1.63930100	1.84969200
H	6.18795600	1.02458700	0.77631200
H	-0.86283000	-1.55147900	0.37813200
H	-2.76653300	-1.45729700	1.42444000
H	-5.19751000	-1.90239000	1.42846400
H	-6.71856100	-0.53597600	0.01770900

H	-5.78210900	1.28680000	-1.38923600
H	-3.32096900	1.72306200	-1.36909600

Dihedral = 90

SCF = -782.6008

XYZ =

28

090-oxazole-Ph.com

C	5.28997100	0.52605900	-0.53495400
C	4.11670000	0.80339900	-1.23087000
C	2.92461300	0.28972200	-0.71894700
C	2.94211500	-0.47162500	0.45341900
C	4.09745700	-0.75742900	1.16522400
C	5.28136100	-0.24059100	0.64150800
N	1.60969100	0.39069200	-1.16203200
C	0.89679400	-0.27290900	-0.29831000
C	-0.52303300	-0.57480700	-0.31151400
C	-1.39468600	0.27130900	0.36779200
C	-2.88214300	0.07024000	0.12840000
O	-1.06126200	1.19296700	1.16337400
O	1.66489000	-0.84616600	0.71242700
C	-3.43652800	-1.11895900	-0.35753200
C	-4.80981500	-1.23748900	-0.55795900
C	-5.65544000	-0.16608700	-0.27299500
C	-5.11558300	1.01962400	0.22312100

C	-3.74299800	1.12982600	0.42734500
H	6.23459100	0.91090800	-0.90682800
H	4.12029600	1.39889800	-2.13814500
H	4.07775100	-1.35044100	2.07285400
H	6.21757700	-0.43591900	1.15508400
H	-0.87133900	-1.15465600	-1.15911300
H	-2.78845400	-1.96691200	-0.56003800
H	-5.22208500	-2.17127700	-0.93049500
H	-6.72653500	-0.25818700	-0.42907000
H	-5.76726200	1.85797200	0.45470400
H	-3.30411700	2.03866500	0.82649400

Dihedral = 100

SCF = -782.602

XYZ =

28

100-oxazole-Ph.com

C	5.29724300	0.51106600	0.10803100
C	4.21435100	1.19461500	-0.44030200
C	2.97015000	0.56463300	-0.41527700
C	2.85193400	-0.71123400	0.14605100
C	3.91487700	-1.41313100	0.69076400
C	5.15168200	-0.76881900	0.66445900
N	1.72487800	0.96084700	-0.88950600
C	0.91368200	-0.01941400	-0.60634800

C	-0.51440600	-0.12850800	-0.77253700
C	-1.38631000	0.57030000	0.06795200
C	-2.85070100	0.14670500	0.03849600
O	-1.10101400	1.53927800	0.81739900
O	1.54938900	-1.07878900	0.04814400
C	-3.29465200	-1.13694900	-0.29617500
C	-4.65220700	-1.45144700	-0.28204200
C	-5.59350600	-0.48495200	0.06570900
C	-5.16319200	0.79543700	0.41147400
C	-3.80534100	1.09992600	0.40624400
H	6.27805500	0.97693900	0.10574600
H	4.32679300	2.18356000	-0.87290800
H	3.78754600	-2.40154700	1.11857700
H	6.01879300	-1.26781400	1.08587100
H	-0.85442600	-1.05367300	-1.22511700
H	-2.57505300	-1.91058100	-0.54679100
H	-4.97415300	-2.45747300	-0.53684800
H	-6.65183600	-0.72955200	0.07489400
H	-5.88844400	1.55555800	0.68977700
H	-3.45005200	2.08539300	0.69000400

Dihedral = 110

SCF = -782.60535

XYZ =

28

110-oxazole-Ph.com

C	5.27607300	0.52639500	0.21415900
C	4.18566000	1.23749400	-0.28457500
C	2.95358900	0.58564100	-0.33854200
C	2.85713600	-0.74074100	0.09893100
C	3.92615900	-1.46939600	0.59049700
C	5.15125900	-0.80162700	0.64537300
N	1.70961000	1.00075800	-0.79435200
C	0.91502700	-0.02206800	-0.61857800
C	-0.50064800	-0.14474700	-0.79784600
C	-1.39568600	0.62525000	-0.03809900
C	-2.84780100	0.15745800	0.00526300
O	-1.13304300	1.68063700	0.58618400
O	1.56323400	-1.12475600	-0.05210900
C	-3.27257800	-1.14874500	-0.26124000
C	-4.62001600	-1.49416900	-0.17900100
C	-5.57116800	-0.53795000	0.17018400
C	-5.16036300	0.76509400	0.44682000
C	-3.81241400	1.10158000	0.37116700
H	6.24645900	1.01049900	0.27114500
H	4.28368700	2.26474900	-0.62094900
H	3.81357600	-2.49652400	0.92035100
H	6.02248600	-1.32183500	1.03121000
H	-0.82113000	-1.12851600	-1.12185500

H	-2.54872100	-1.91742000	-0.51292800
H	-4.92603900	-2.51669400	-0.38248200
H	-6.62159300	-0.80753400	0.23244200
H	-5.89258100	1.51849500	0.72499100
H	-3.47032100	2.10561600	0.59885900

Dihedral = 120

SCF = -782.6088

XYZ =

28

120-oxazole-Ph.com

C	5.27891500	0.54257300	0.24785300
C	4.16669300	1.27590200	-0.16579900
C	2.94538400	0.60879000	-0.26367200
C	2.88176500	-0.75510100	0.05000700
C	3.97144700	-1.50488200	0.45410400
C	5.18651500	-0.82161900	0.55321900
N	1.68905900	1.04095500	-0.66112500
C	0.91460800	-0.01171400	-0.56825900
C	-0.49257100	-0.15684600	-0.74173300
C	-1.41631700	0.65276000	-0.05624000
C	-2.85849200	0.15789600	0.00228100
O	-1.18638300	1.75615300	0.48883200
O	1.59204900	-1.14976400	-0.11673800
C	-3.25104100	-1.17511300	-0.15925300

C -4.59267200 -1.54108800 -0.07106300

C -5.56901900 -0.57895300 0.17866100

C -5.19019800 0.75153100 0.35052800

C -3.84813100 1.10916800 0.27075700

H 6.24088700 1.03890200 0.33528900

H 4.24064400 2.33215700 -0.40451300

H 3.88320900 -2.56094600 0.68554000

H 6.07330200 -1.35964700 0.87349100

H -0.79906400 -1.15893400 -1.01809400

H -2.50671700 -1.94600900 -0.33128200

H -4.87482300 -2.58340800 -0.19124200

H -6.61481300 -0.86506500 0.24483000

H -5.94292200 1.50967000 0.54905600

H -3.52976500 2.13566300 0.41972100

Dihedral = 130

SCF = -782.6122

XYZ =

28

130-oxazole-Ph.com

C 5.27983300 0.58176900 0.24615300

C 4.14041100 1.30684900 -0.10599800

C 2.93339000 0.61553800 -0.21418100

C 2.91182100 -0.76454600 0.03004900

C 4.02743600 -1.50527900 0.37261500

C	5.22843600	-0.79742500	0.48253900
N	1.65945300	1.03655500	-0.55976800
C	0.91287500	-0.04178700	-0.50459100
C	-0.48564800	-0.22123600	-0.66223300
C	-1.42813000	0.64683400	-0.07765000
C	-2.87015600	0.15375500	-0.00135300
O	-1.20688600	1.79493400	0.36632000
O	1.62863500	-1.18468400	-0.13151000
C	-3.26235200	-1.18494600	-0.11024900
C	-4.60462500	-1.54658800	-0.01781300
C	-5.58253200	-0.57480900	0.18470600
C	-5.20449600	0.76146300	0.30398300
C	-3.86184200	1.11566500	0.21774800
H	6.23064000	1.09834000	0.33932000
H	4.18361000	2.37550200	-0.29067000
H	3.96992000	-2.57371600	0.55100100
H	6.13453900	-1.32855600	0.75664500
H	-0.77835000	-1.24184800	-0.87447700
H	-2.51909800	-1.96347300	-0.24749300
H	-4.88633000	-2.59282600	-0.09824200
H	-6.62889000	-0.85792500	0.25450800
H	-5.95832000	1.52710600	0.46561700
H	-3.54440700	2.14797400	0.32134800

Dihedral = 140

SCF = -782.61506

XYZ =

28

140-oxazole-Ph.com

C	5.29648500	0.60457700	0.19502400
C	4.12957600	1.33245400	-0.04417100
C	2.92988700	0.62826600	-0.15195300
C	2.94444100	-0.76800700	-0.02174900
C	4.08636200	-1.51054100	0.20938600
C	5.27997900	-0.78941600	0.32205800
N	1.63433000	1.05139600	-0.39593600
C	0.90833800	-0.04510900	-0.39224200
C	-0.48610500	-0.24824200	-0.51367800
C	-1.44473200	0.64062700	0.01191100
C	-2.88661600	0.15066400	0.03549600
O	-1.24025100	1.79957000	0.43297700
O	1.66212900	-1.20073900	-0.15938900
C	-3.26271500	-1.19609500	0.07348800
C	-4.60744900	-1.56045100	0.09684500
C	-5.60028400	-0.58312100	0.07865600
C	-5.23754100	0.76288800	0.05195800
C	-3.89326900	1.12111000	0.04055300
H	6.24174100	1.13177200	0.28598700
H	4.14662000	2.41312500	-0.14326200

H	4.05462400	-2.59073100	0.30449800
H	6.20682600	-1.32137800	0.51242500
H	-0.77839400	-1.26568300	-0.74053200
H	-2.50272200	-1.97077400	0.10975600
H	-4.87997900	-2.61149000	0.13509100
H	-6.64833300	-0.86825800	0.09223000
H	-6.00472600	1.53248500	0.04194400
H	-3.58913200	2.16286700	0.03521000

Dihedral = 150

SCF = -782.6175

XYZ =

28

150-oxazole-Ph.com

C	5.30168000	0.60853500	0.22014600
C	4.12240900	1.33881900	0.06072600
C	2.92766100	0.63124900	-0.07616200
C	2.95893600	-0.77092700	-0.04873500
C	4.11252000	-1.51501600	0.10214100
C	5.30220700	-0.79066600	0.24136400
N	1.62513500	1.05804100	-0.26302600
C	0.90887800	-0.04503600	-0.33138600
C	-0.47876700	-0.26472200	-0.45679600
C	-1.45903800	0.65821300	-0.03757300
C	-2.89574900	0.15406400	0.01455900

O	-1.27215800	1.85403900	0.27088300
O	1.67811300	-1.20670000	-0.19507000
C	-3.25642800	-1.19345200	0.12094800
C	-4.59651800	-1.57173200	0.16934200
C	-5.60087300	-0.60764700	0.11229300
C	-5.25369300	0.73938700	0.01908600
C	-3.91393600	1.11186300	-0.02125800
H	6.24344300	1.13848500	0.32988700
H	4.12650200	2.42403400	0.04250700
H	4.09391400	-2.59961000	0.11457000
H	6.23873300	-1.32463100	0.36805100
H	-0.75990100	-1.29145300	-0.64997600
H	-2.48836700	-1.95738100	0.19199300
H	-4.85601300	-2.62294900	0.25883700
H	-6.64539100	-0.90368900	0.14735200
H	-6.02972200	1.49898800	-0.02097000
H	-3.62251400	2.15560900	-0.07974600

Dihedral = 160

SCF = -782.61925

XYZ =

28

160-oxazole-Ph.com

C	5.31281300	0.62906500	0.15944300
C	4.11743900	1.34767300	0.08845900

C	2.92685800	0.62919700	-0.02759900
C	2.97896000	-0.77239300	-0.07034700
C	4.14773800	-1.50458500	-0.00467000
C	5.33328600	-0.76918600	0.11492800
N	1.61165200	1.04458200	-0.12744100
C	0.90766100	-0.06648800	-0.21774500
C	-0.47679000	-0.30683200	-0.30621400
C	-1.46635500	0.64490300	0.01575600
C	-2.90709900	0.15275700	0.02955800
O	-1.28220300	1.85436800	0.26508000
O	1.69895800	-1.22081900	-0.18194500
C	-3.28021300	-1.18315600	0.21086000
C	-4.62307600	-1.55439900	0.21604100
C	-5.61747400	-0.59441300	0.03807300
C	-5.25818200	0.74205200	-0.13086100
C	-3.91613000	1.10846200	-0.12609100
H	6.25129900	1.16827600	0.25116400
H	4.10648900	2.43242800	0.12237300
H	4.14395500	-2.58882100	-0.04161300
H	6.28131300	-1.29459600	0.17461800
H	-0.75461000	-1.33594800	-0.48956800
H	-2.51984000	-1.94083000	0.37304800
H	-4.89306800	-2.59593100	0.36654800
H	-6.66411800	-0.88492700	0.03908800

H	-6.02643200	1.49857600	-0.26468700
H	-3.61720800	2.14537100	-0.24149900

Dihedral = 170

SCF = -782.62036

XYZ =

28

170-oxazole-Ph.com

C	5.31605100	0.63607100	0.14824700
C	4.11252600	1.34464400	0.16880700
C	2.92469200	0.62361600	0.04023700
C	2.98806300	-0.77120100	-0.10315200
C	4.16449100	-1.49316300	-0.12762500
C	5.34738900	-0.75500900	0.00348500
N	1.60343600	1.03070900	0.01680800
C	0.90677400	-0.08003100	-0.13159000
C	-0.47488900	-0.33205100	-0.20295100
C	-1.47070400	0.64103300	0.02446000
C	-2.91223800	0.15403500	0.02654000
O	-1.28803200	1.86335100	0.19782700
O	1.70923100	-1.22477700	-0.20690700
C	-3.29306900	-1.16341700	0.30193800
C	-4.63646700	-1.53306500	0.29749000
C	-5.62254100	-0.58995500	0.01501300
C	-5.25533500	0.72900800	-0.24946300

C	-3.91326200	1.09485800	-0.23406000
H	6.25234400	1.17791800	0.24667600
H	4.09368200	2.42408500	0.28041900
H	4.16883600	-2.57206100	-0.24163100
H	6.30122800	-1.27310900	-0.00798200
H	-0.75063100	-1.36268200	-0.38042800
H	-2.53795700	-1.90450300	0.54615100
H	-4.91338300	-2.55917600	0.52265600
H	-6.66964300	-0.87874800	0.00935400
H	-6.01809400	1.47281500	-0.46302400
H	-3.60891100	2.11966600	-0.42145400

Dihedral = 180

SCF = -782.6208

XYZ =

28

180-oxazole-Ph.com

C	5.32140700	0.63677000	0.05740200
C	4.11875900	1.33761500	0.17328200
C	2.92784200	0.61551200	0.08435500
C	2.98797100	-0.77218000	-0.11732000
C	4.16331900	-1.48656900	-0.23482900
C	5.34941100	-0.74750900	-0.14221100
N	1.60608900	1.01501300	0.15696400
C	0.90589200	-0.09313900	0.00382000

C	-0.47632900	-0.35037900	-0.01599800
C	-1.47335400	0.64021600	0.10852600
C	-2.91522100	0.15937500	0.05199800
O	-1.29154900	1.86879400	0.22955100
O	1.70692100	-1.22834000	-0.16869100
C	-3.32081500	-1.13474800	0.39413400
C	-4.66271700	-1.50276400	0.32308300
C	-5.62145300	-0.58191200	-0.09491100
C	-5.22954200	0.71434400	-0.42785800
C	-3.88992700	1.07994000	-0.34413800
H	6.25983600	1.17986200	0.12366300
H	4.10281500	2.41181200	0.32753800
H	4.16445000	-2.56025500	-0.39049400
H	6.30306600	-1.25886400	-0.22819600
H	-0.75482500	-1.38112800	-0.18928500
H	-2.58725000	-1.85565800	0.74267000
H	-4.96081800	-2.50966400	0.60146000
H	-6.66707600	-0.87010000	-0.15346500
H	-5.97089200	1.44000100	-0.75119700
H	-3.56635600	2.08782900	-0.58432600

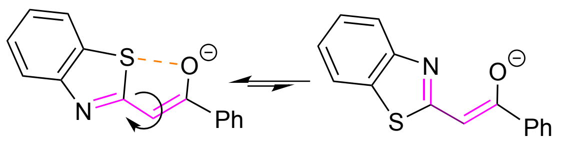

Anionic Benzothiazole

Supporting Information: thiazole-Ph.log

Using Gaussian 09: AM64L-G09RevD.01 24-Apr-2013

==

#m062X/6-31G(d) scf=(maxcycle=300,direct,tight) density=current

SCRF=(PCM,SOLVENT=THF) opt=(maxcycle=250,modredundant,gdiis) iop(1/8=18)

Modredundant Input: D 13 8 9 10 S 18 10.000

Pointgroup= C1 Stoichiometry= C15H10NOS(1-) C1[X(C15H10NOS)] #Atoms= 28

Charge = -1 Multiplicity = 1

==

Optimization completed. {Found 19 times}

Item	Max Val.	Criteria	Pass?	RMS Val.	Criteria	Pass?
Force	0.00001 ||	0.00045	[YES]	0.00000 ||	0.00030	[YES]
Displ	0.00054 ||	0.00180	[YES]	0.00054 ||	0.00180	[YES]

Dihedral = 0

SCF = -1105.59447

XYZ =

28

000-thiazole-Ph.com

C	-5.33313800	0.95875500	-0.12292900
C	-4.10394000	1.60043600	-0.19804100
C	-2.92234600	0.84815400	-0.10695800
C	-3.01770400	-0.55269400	0.05997300
C	-4.25089900	-1.19398300	0.13460500

C	-5.41257200	-0.43026900	0.04265900
N	-1.65049300	1.36416500	-0.16388100
C	-0.73418700	0.42020500	-0.04639300
C	0.65726500	0.63825600	-0.05504100
C	1.59496200	-0.40204500	0.04984800
C	3.06546100	-0.04623300	0.02231400
O	1.29903900	-1.61957500	0.14436300
S	-1.40930900	-1.23882400	0.14182000
C	3.56075000	1.22413900	0.33482200
C	4.92893200	1.48196200	0.29780800
C	5.82407500	0.47338400	-0.05419400
C	5.34140900	-0.79874500	-0.35826900
C	3.97464600	-1.05447300	-0.31167800
H	-6.24671800	1.54271500	-0.19160200
H	-4.03638000	2.67695700	-0.32480200
H	-4.30741300	-2.27143500	0.26343600
H	-6.38214100	-0.91532300	0.10148600
H	0.96909800	1.66556300	-0.19498800
H	2.87781000	2.01398900	0.63245300
H	5.29740400	2.47191300	0.55135300
H	6.89064700	0.67612600	-0.08465600
H	6.03254900	-1.59209300	-0.62908700
H	3.57894700	-2.04087500	-0.53160700

Dihedral = 10

SCF = -1105.59383

XYZ =

28

010-thiazole-Ph.com

C	-5.32640600	0.96457800	-0.20238800
C	-4.09297300	1.59857000	-0.27377900

C	-2.91705400	0.84632200	-0.12744800
C	-3.02299000	-0.54645100	0.09168800
C	-4.26000700	-1.18106500	0.15790900
C	-5.41586300	-0.41701200	0.01220700
N	-1.64190700	1.35480800	-0.17785700
C	-0.73199900	0.41155400	-0.01408700
C	0.65812000	0.63095400	0.04668600
C	1.59756900	-0.41391800	0.05845800
C	3.06660200	-0.05219700	0.02140900
O	1.30595300	-1.63519900	0.08777700
S	-1.41912400	-1.23542900	0.22506200
C	3.56506000	1.19915300	0.39926100
C	4.93123600	1.46451600	0.34856100
C	5.82126500	0.48278300	-0.08374800
C	5.33566400	-0.77084700	-0.45290900
C	3.97104700	-1.03509000	-0.39151700
H	-6.23574600	1.54860300	-0.31387600
H	-4.01811500	2.66914500	-0.44010500
H	-4.32387600	-2.25309200	0.32304200
H	-6.38861900	-0.89611900	0.06687700
H	0.97039700	1.66060200	-0.07569600
H	2.88619600	1.96638600	0.75872100
H	5.30222000	2.43856300	0.65447600
H	6.88630800	0.69149100	-0.12549500
H	6.02308900	-1.54310400	-0.78678700
H	3.57421600	-2.00875800	-0.66116700

Dihedral = 20

SCF = -1105.5922

XYZ =

28

020-thiazole-Ph.com

C	-5.31130100	0.97967800	-0.28982100
C	-4.07066200	1.60098500	-0.34659100
C	-2.90619300	0.84455900	-0.14286100
C	-3.03095400	-0.53940200	0.11834000
C	-4.27469400	-1.16250600	0.16536600
C	-5.41902000	-0.39381700	-0.03539100
N	-1.62524600	1.34052900	-0.17656300
C	-0.72816600	0.39503700	0.03329800
C	0.65870600	0.61063900	0.16711400
C	1.60077400	-0.43003500	0.07975600
C	3.06726300	-0.05918000	0.02495200
O	1.31409600	-1.65188900	0.04184600
S	-1.43692100	-1.23608800	0.30988200
C	3.57239000	1.16954200	0.46397600
C	4.93553300	1.44445700	0.38988900
C	5.81565700	0.49526600	-0.12766900
C	5.32358400	-0.73585700	-0.55881200
C	3.96223700	-1.01039200	-0.47400600
H	-6.21176300	1.56696700	-0.44678100
H	-3.98151600	2.66482400	-0.54586200
H	-4.35263800	-2.22856700	0.36007200
H	-6.39713400	-0.86315900	0.00659000
H	0.97008300	1.64364900	0.07004100
H	2.90098400	1.91005500	0.88772800
H	5.31241200	2.40000300	0.74317900
H	6.87834600	0.71145800	-0.18776900
H	6.00408600	-1.48255000	-0.95838900
H	3.56091000	-1.96770300	-0.79098900

Dihedral = 30

SCF = -1105.5895

XYZ =

28

030-thiazole-Ph.com

C	-5.29226700	0.99871400	-0.36195000
C	-4.04325700	1.60374300	-0.41264000
C	-2.89250800	0.84118300	-0.16098500
C	-3.03935900	-0.53222000	0.14157800
C	-4.29085400	-1.14042400	0.17867700
C	-5.42141600	-0.36497900	-0.06789700
N	-1.60456800	1.32157200	-0.18364100
C	-0.72311300	0.37369600	0.07059300
C	0.65825700	0.58133600	0.27676500
C	1.60298100	-0.44563600	0.08951200
C	3.06712700	-0.06525100	0.02716500
O	1.31941100	-1.66301400	-0.02532300
S	-1.45801200	-1.23896500	0.38494900
C	3.57899100	1.13816700	0.52468800
C	4.93945200	1.42205500	0.43529100
C	5.80985600	0.50799700	-0.15658400
C	5.31115300	-0.69813900	-0.64667100
C	3.95290100	-0.98229500	-0.54634200
H	-6.18205300	1.59103800	-0.55594600
H	-3.93719600	2.65966800	-0.64344200
H	-4.38535800	-2.19914400	0.40344700

H -6.40575900 -0.82152300 -0.03098000

H 0.96906300 1.61773800 0.21176700

H 2.91545800 1.85138700 1.00423400

H 5.32201100 2.35790200 0.83253600

H 6.87023400 0.73172300 -0.22899200

H 5.98421400 -1.41854400 -1.10321800

H 3.54660000 -1.92137800 -0.90772300

Dihedral = 40

SCF = -1105.585875

XYZ =

28

040-thiazole-Ph.com

C -5.26938200 1.02857100 -0.43462400

C -4.00800300 1.60777800 -0.48241500

C -2.87785400 0.83450200 -0.17731400

C -3.05787200 -0.52244900 0.17736000

C -4.32113100 -1.10640600 0.20963400

C -5.43061900 -0.31977500 -0.09037000

N -1.57902100 1.28799200 -0.19844000

C -0.72016100 0.33522100 0.10227600

C 0.66006100 0.52692700 0.35045600

C 1.61069500 -0.47154600 0.05798000

C 3.07160100 -0.07185800 0.01113600

O 1.34014300 -1.67779300 -0.15528800

S	-1.49661600	-1.24519400	0.48005800
C	3.57793300	1.09664400	0.59022700
C	4.93612200	1.39577500	0.51461900
C	5.80976300	0.53231300	-0.14433000
C	5.31647800	-0.63910000	-0.71692700
C	3.96065500	-0.93892400	-0.63060800
H	-6.14316000	1.62908600	-0.67087400
H	-3.87601200	2.65104600	-0.75359200
H	-4.44088000	-2.15349000	0.47287700
H	-6.42411500	-0.75626000	-0.05687200
H	0.96708600	1.56691500	0.32221700
H	2.91266100	1.76880100	1.12378600
H	5.31453100	2.30411000	0.97490600
H	6.86830400	0.76802600	-0.20486500
H	5.99164400	-1.32092400	-1.22667200
H	3.55808000	-1.85385300	-1.05305100

Dihedral = 50

SCF = -1105.5815

XYZ =

28

050-thiazole-Ph.com

C	-5.23429600	1.08520100	-0.49943800
C	-3.95461300	1.62280800	-0.54486400

C	-2.85525000	0.82483200	-0.19547400
C	-3.08365800	-0.51301700	0.20136500
C	-4.36517100	-1.05674100	0.23028400
C	-5.44308900	-0.24547900	-0.11437700
N	-1.54131100	1.23599000	-0.21106800
C	-0.71649700	0.27082500	0.13414900
C	0.66485200	0.43671600	0.41819000
C	1.62442800	-0.51896000	0.02338400
C	3.07867700	-0.08791300	0.00008100
O	1.37522900	-1.70559600	-0.29545900
S	-1.55339700	-1.26325200	0.57088800
C	3.56966400	1.03301000	0.67801600
C	4.92086800	1.36518200	0.61675900
C	5.80369000	0.58358200	-0.12692600
C	5.32633400	-0.54105000	-0.79811900
C	3.97760200	-0.87508800	-0.72522900
H	-6.08525900	1.70441000	-0.76802900
H	-3.78454000	2.65206700	-0.84648500
H	-4.52271200	-2.08991600	0.52600400
H	-6.45037500	-0.64926400	-0.08367500
H	0.96696800	1.47923000	0.43583000
H	2.89819000	1.64048100	1.27719000
H	5.28627300	2.23494900	1.15556000
H	6.85659600	0.84571300	-0.17644400

H	6.00750700	-1.15983300	-1.37572700
H	3.58780100	-1.75641000	-1.22409600

Dihedral = 60

SCF = -1105.577

XYZ =

28

060-thiazole-Ph.com

C	-5.20004500	1.11665100	-0.56873200
C	-3.90626900	1.61800600	-0.62225000
C	-2.83463800	0.81089400	-0.21494300
C	-3.10214600	-0.49861500	0.24570600
C	-4.39859400	-1.00682100	0.28466100
C	-5.44860600	-0.18656000	-0.11815300
N	-1.50852700	1.18606800	-0.23807500
C	-0.71281400	0.22121700	0.16000800
C	0.67667900	0.35819200	0.44745800
C	1.63039900	-0.54918100	-0.05992500
C	3.08182900	-0.10804300	-0.02960400
O	1.38785300	-1.68851200	-0.52337600
S	-1.59679900	-1.25738000	0.68552700
C	3.56823700	0.87863500	0.83481600
C	4.91445700	1.23495300	0.82148600
C	5.79572900	0.61213000	-0.06081400
C	5.32298200	-0.37941800	-0.91953700

C 3.97898600 -0.74025200 -0.89432400

H -6.03091700 1.74228700 -0.88144000

H -3.70429600 2.62501300 -0.97466000

H -4.58748900 -2.01843900 0.63156400

H -6.46661100 -0.56194400 -0.08174800

H 0.98318800 1.39670300 0.53368200

H 2.89248900 1.35499700 1.53908500

H 5.27828400 1.99631700 1.50570000

H 6.84508400 0.89246800 -0.07350300

H 6.00494600 -0.87328700 -1.60635000

H 3.59385900 -1.52114500 -1.54234900

Dihedral = 70

SCF = -1105.5725

XYZ =

28

070-thiazole-Ph.com

C -5.18078800 1.11195000 -0.62005500

C -3.88215700 1.60057900 -0.66304700

C -2.82721500 0.79644900 -0.21109800

C -3.11314600 -0.49620400 0.28339500

C -4.41564700 -0.99165500 0.31409300

C -5.44882500 -0.17544800 -0.13510000

N -1.49472700 1.15791000 -0.22803100

C	-0.71537500	0.20126000	0.20631300
C	0.68470300	0.31298100	0.49461500
C	1.62343800	-0.51110500	-0.15440100
C	3.08217600	-0.09830900	-0.06200600
O	1.36710300	-1.56040900	-0.79343800
S	-1.62254600	-1.24460600	0.78176100
C	3.58148500	0.76961300	0.91498300
C	4.93396900	1.10030500	0.94914700
C	5.81018800	0.56825900	0.00401500
C	5.32493600	-0.30619600	-0.96732800
C	3.97406800	-0.64018500	-0.99144500
H	-5.99994700	1.73405400	-0.96838400
H	-3.66393500	2.59421400	-1.04242700
H	-4.62034500	-1.99051700	0.68756500
H	-6.47065400	-0.54117100	-0.10849000
H	0.99008900	1.33383900	0.70764800
H	2.91021000	1.17178500	1.66793600
H	5.30658200	1.77071000	1.71860400
H	6.86459100	0.82812100	0.02945500
H	6.00299600	-0.72988500	-1.70311500
H	3.57653700	-1.33330900	-1.72572500

Dihedral = 80

SCF = -1105.56825

XYZ =

28

080-thiazole-Ph.com

C	-5.13463100	1.23209100	-0.51091200
C	-3.82244700	1.68242900	-0.48544200
C	-2.80030700	0.80186200	-0.10839800
C	-3.12890700	-0.52712200	0.24106000
C	-4.44672700	-0.98272000	0.20396700
C	-5.44680700	-0.09160300	-0.16849000
N	-1.45610100	1.12379000	-0.06878600
C	-0.71294700	0.10884000	0.27399600
C	0.69701200	0.13756000	0.57876100
C	1.62077500	-0.47300600	-0.28386700
C	3.08022500	-0.09686500	-0.09680600
O	1.35604100	-1.30175500	-1.19118800
S	-1.66797700	-1.36086600	0.68260000
C	3.59727600	0.41974100	1.09618500
C	4.94759200	0.74186700	1.20711700
C	5.80431100	0.55397700	0.12331000
C	5.30131300	0.03071800	-1.06710200
C	3.95270400	-0.29833700	-1.16950500
H	-5.93034900	1.91109700	-0.80228700
H	-3.56814300	2.70335200	-0.75320800
H	-4.68713200	-2.00856500	0.46611500

H	-6.47931000	-0.42624400	-0.19427900
H	1.00823400	1.05951900	1.06287000
H	2.93913100	0.54904100	1.95061100
H	5.33409200	1.13414600	2.14372300
H	6.85728000	0.80685300	0.20873700
H	5.96406400	-0.12366900	-1.91438600
H	3.54352800	-0.72076400	-2.08172500

Dihedral = 90

SCF = -1105.5649

XYZ =

28

090-thiazole-Ph.com

C	-5.12787500	1.28121000	0.27727200
C	-3.81625600	1.62729100	0.56459500
C	-2.80046800	0.68170700	0.37516000
C	-3.12956900	-0.60461000	-0.10454500
C	-4.44989500	-0.94993900	-0.39970800
C	-5.44345900	0.00050700	-0.20145100
N	-1.45458100	0.91279000	0.61360900
C	-0.71923500	-0.11909300	0.33398900
C	0.71087200	-0.27981800	0.54955300
C	1.59904900	-0.02347500	-0.49278500
C	3.07642300	0.02997400	-0.13925100

O	1.29388600	0.17376200	-1.70170100
S	-1.67151000	-1.54346700	-0.21236700
C	3.62682400	-0.56153700	1.00312300
C	4.99105600	-0.46866400	1.26813200
C	5.83111700	0.21756100	0.39209300
C	5.29582300	0.79936400	-0.75605300
C	3.93271700	0.69688800	-1.01913500
H	-5.91990700	2.00952400	0.42179400
H	-3.55688200	2.61552300	0.93163300
H	-4.69492100	-1.93979500	-0.77192600
H	-6.47624300	-0.25216100	-0.42130000
H	1.03914500	-0.17080500	1.57819200
H	2.98451500	-1.11954100	1.67844900
H	5.40192200	-0.93929700	2.15722500
H	6.89497700	0.29093300	0.59916400
H	5.94392600	1.33099600	-1.44785600
H	3.49587100	1.12626500	-1.91508900

Dihedral = 100

SCF = -1105.56625

XYZ =

28

100-thiazole-Ph.com

C	-5.14141600	1.10885000	0.13530000
C	-3.87741700	1.45644600	0.59054500

C	-2.81331700	0.56283000	0.41629900
C	-3.05030600	-0.67593000	-0.22071500
C	-4.32331400	-1.03007200	-0.66729900
C	-5.36478000	-0.12617000	-0.48992900
N	-1.51869900	0.78469800	0.85161300
C	-0.72625800	-0.21061900	0.58993700
C	0.70248800	-0.27517500	0.76749800
C	1.53259800	0.50016600	-0.05207100
C	3.02588500	0.19062300	-0.02492500
O	1.16957800	1.45196600	-0.78747000
S	-1.55011900	-1.55008700	-0.31327100
C	3.58293300	-1.01470000	0.41717500
C	4.96211000	-1.21205500	0.40885900
C	5.81403100	-0.20549600	-0.04193500
C	5.27178300	0.99607500	-0.49485300
C	3.89288000	1.18399900	-0.49190100
H	-5.96764400	1.80123600	0.26527300
H	-3.69330800	2.40869300	1.07835400
H	-4.49520900	-1.98668600	-1.15095500
H	-6.35998500	-0.38106100	-0.84112100
H	1.08763800	-1.19610800	1.19125800
H	2.94008500	-1.82115300	0.75576200
H	5.37168600	-2.15816500	0.75202100
H	6.88928200	-0.35933500	-0.04560800

H	5.92622700	1.78637600	-0.85278500
H	3.44999000	2.10635000	-0.85330400

Dihedral = 110

SCF = -1105.5695

XYZ =

28

110-thiazole-Ph.com

C	-5.15426200	1.10060800	0.01854600
C	-3.88390400	1.50429300	0.40708600
C	-2.81647500	0.60111800	0.32154400
C	-3.06115900	-0.70525800	-0.16087000
C	-4.33874400	-1.11442900	-0.53821200
C	-5.38324700	-0.19955200	-0.45150400
N	-1.51869800	0.87423900	0.70992600
C	-0.72672700	-0.14875400	0.55848900
C	0.69351900	-0.21111700	0.73677500
C	1.56046200	0.59908100	-0.01748200
C	3.03630000	0.21419100	-0.01818400
O	1.25140000	1.62392400	-0.66858700
S	-1.55932400	-1.58415400	-0.17954700
C	3.52273900	-1.07119500	0.24368100
C	4.89001300	-1.33856400	0.21388500
C	5.79855500	-0.32339700	-0.07801800
C	5.32620200	0.95930500	-0.35188900

C	3.95923300	1.21800000	-0.32969100
H	-5.98191900	1.80076300	0.08185000
H	-3.69833900	2.50781100	0.77761200
H	-4.51302900	-2.12253400	-0.90192300
H	-6.38295000	-0.49790200	-0.75176700
H	1.07125900	-1.16253600	1.09601800
H	2.83175800	-1.88248100	0.45032500
H	5.24584900	-2.34580300	0.41241400
H	6.86433300	-0.53201900	-0.09872700
H	6.02559900	1.75768900	-0.58503000
H	3.57128200	2.20642200	-0.55374400

Dihedral = 120

SCF = -1105.5729

XYZ =

28

120-thiazole-Ph.com

C	-5.14180900	1.11214700	-0.10050100
C	-3.86534800	1.53684800	0.24506800
C	-2.80692000	0.61887300	0.24381900
C	-3.06857500	-0.72392100	-0.11716600
C	-4.35092800	-1.15282800	-0.44970100
C	-5.38717400	-0.22326700	-0.44443000
N	-1.50782700	0.91280600	0.60369000
C	-0.72487900	-0.12987800	0.54422800

C	0.68625400	-0.20090300	0.72776500
C	1.57772100	0.66361000	0.05979300
C	3.03951600	0.23179500	0.00405200
O	1.29957900	1.75907300	-0.47572900
S	-1.57447500	-1.61865100	-0.06362800
C	3.48024000	-1.09028400	0.12690800
C	4.83556200	-1.40318800	0.04481200
C	5.77720800	-0.39744900	-0.16202500
C	5.35051100	0.92284600	-0.29723200
C	3.99514100	1.22825200	-0.22234200
H	-5.96151900	1.82456500	-0.10186100
H	-3.66735000	2.56928100	0.51657900
H	-4.53783700	-2.18897400	-0.71538800
H	-6.39131800	-0.53818400	-0.71097800
H	1.05867200	-1.17533500	1.02359800
H	2.76249800	-1.89286500	0.26282600
H	5.15563000	-2.43751300	0.13482400
H	6.83380100	-0.64152400	-0.22468300
H	6.07682900	1.71358700	-0.46407800
H	3.64124700	2.24723300	-0.34107200

Dihedral = 130

SCF = -1105.57613

XYZ =

28

130-thiazole-Ph.com

C	-5.13904500	1.14659500	-0.10942600
C	-3.84450100	1.56739800	0.16834000
C	-2.80153400	0.63169500	0.19176600
C	-3.10065200	-0.72629200	-0.07301500
C	-4.39933000	-1.14990200	-0.33960900
C	-5.41933500	-0.20195100	-0.36166800
N	-1.48654100	0.92403600	0.48398100
C	-0.72286900	-0.13749400	0.45849200
C	0.68500700	-0.23839000	0.61221100
C	1.59714400	0.66400800	0.02317300
C	3.05707200	0.22784200	-0.01484700
O	1.34008300	1.79120200	-0.45057800
S	-1.62234200	-1.64834100	-0.00710200
C	3.48479700	-1.10385500	0.00280500
C	4.84109700	-1.41947200	-0.04871100
C	5.79549800	-0.40695600	-0.11708900
C	5.38163000	0.92414200	-0.14694000
C	4.02568400	1.23308900	-0.10517400
H	-5.94542300	1.87393200	-0.13038200
H	-3.62052800	2.61084800	0.36801100
H	-4.61208400	-2.19665900	-0.53507500
H	-6.43666400	-0.51315800	-0.57796300
H	1.05090900	-1.22306300	0.87973900

H	2.75658200	-1.90840500	0.03246000
H	5.15212600	-2.46042300	-0.04254900
H	6.85272800	-0.65327400	-0.15475900
H	6.11802300	1.72090800	-0.20739300
H	3.68294600	2.26215400	-0.14354300

Dihedral = 140

SCF = -1105.578835

XYZ =

28

140-thiazole-Ph.com

C	-5.12576900	1.17859500	-0.14103600
C	-3.81706400	1.58980500	0.08027800
C	-2.79029900	0.63734000	0.13834800
C	-3.12225600	-0.72805700	-0.03542800
C	-4.43385700	-1.14110500	-0.24623800
C	-5.43756600	-0.17634300	-0.30294900
N	-1.46412500	0.92216600	0.37604400
C	-0.71922400	-0.15524700	0.39126300
C	0.68381000	-0.28092700	0.52188300
C	1.60806400	0.66513500	0.02321900
C	3.06681300	0.22786700	-0.02186200
O	1.35878100	1.82558500	-0.36323100
S	-1.65989900	-1.67579200	0.05380000
C	3.48842100	-1.10361800	-0.10059000

C 4.84433900 -1.42159200 -0.14438900

C 5.80355300 -0.41189800 -0.10883800

C 5.39558400 0.91977800 -0.04407600

C 4.04013100 1.23196000 -0.01076300

H -5.91820200 1.91996500 -0.18937200

H -3.56966400 2.63914000 0.20912500

H -4.66995500 -2.19354200 -0.37258300

H -6.46532800 -0.48024900 -0.47594300

H 1.04652500 -1.27528000 0.75341800

H 2.75566400 -1.90284400 -0.15420400

H 5.15159000 -2.46139600 -0.21311200

H 6.86050000 -0.66041300 -0.13917300

H 6.13611800 1.71470500 -0.02161500

H 3.70167900 2.26257200 0.02326400

Dihedral = 150

SCF = -1105.5810

XYZ =

28

150-thiazole-Ph.com

C -5.12727900 1.19010300 -0.16995400

C -3.80811900 1.59977700 -0.01722500

C -2.78802900 0.64211900 0.07188900

C -3.13952500 -0.72806600 -0.00221900

C -4.46015300 -1.13871800 -0.14754700

C	-5.45713700	-0.16845200	-0.23397900
N	-1.45334500	0.92761200	0.24836200
C	-0.71882900	-0.15753100	0.31516200
C	0.67978200	-0.30413100	0.43471300
C	1.62231800	0.67080700	0.03279500
C	3.07857800	0.22744300	-0.01690300
O	1.38817300	1.85931500	-0.26352200
S	-1.68519000	-1.68631300	0.11205600
C	3.49232400	-1.09742600	-0.18995300
C	4.84675000	-1.42132400	-0.23707400
C	5.81158700	-0.42410100	-0.11026000
C	5.41129500	0.90182300	0.05009600
C	4.05772300	1.22078300	0.08640900
H	-5.91362000	1.93617600	-0.24050900
H	-3.54730600	2.65243300	0.03582800
H	-4.70995300	-2.19445100	-0.19767600
H	-6.49265000	-0.47177500	-0.35310000
H	1.03468100	-1.30692000	0.63926800
H	2.75434400	-1.88351800	-0.31613600
H	5.14865500	-2.45490700	-0.38134800
H	6.86730800	-0.67734200	-0.14393300
H	6.15666600	1.68664600	0.14507200
H	3.72521700	2.24822000	0.19358600

Dihedral = 160

SCF = -1105.5827

XYZ =

28

160-thiazole-Ph.com

C	-5.12854700	1.20881700	-0.14089600
C	-3.79949100	1.60714500	-0.05976700
C	-2.78713100	0.64110000	0.03031700
C	-3.15775900	-0.72628300	0.03407300
C	-4.48746100	-1.12526400	-0.04203200
C	-5.47634500	-0.14662900	-0.13179600
N	-1.44288500	0.91672500	0.12862300
C	-0.71753300	-0.17523100	0.20509800
C	0.67980700	-0.34079900	0.27929100
C	1.63060400	0.66047200	-0.02926900
C	3.08981700	0.22618200	-0.03729600
O	1.40183600	1.86189600	-0.27138100
S	-1.71147700	-1.69875400	0.13632600
C	3.51850600	-1.08299200	-0.27952400
C	4.87525300	-1.40058100	-0.27879700
C	5.82690500	-0.41327900	-0.03190300
C	5.41200500	0.89788400	0.19834700
C	4.05679600	1.21185400	0.18484400
H	-5.90828600	1.96162000	-0.21355800
H	-3.52451200	2.65753300	-0.06558000

H -4.75138200 -2.17874400 -0.03598300

H -6.51917100 -0.44134100 -0.19636500

H 1.03273200 -1.34721100 0.46775400

H 2.79144800 -1.85985300 -0.49561300

H 5.18941300 -2.42124900 -0.47830700

H 6.88420600 -0.66201600 -0.02741000

H 6.14762200 1.67555100 0.38444100

H 3.71451700 2.22915100 0.34594600

Dihedral = 170

SCF = -1105.58361

XYZ =

28

170-thiazole-Ph.com

C -5.12035100 1.22591900 -0.15235300

C -3.78487100 1.61088300 -0.12977600

C -2.78051600 0.63794800 -0.02208500

C -3.16736100 -0.72259200 0.05956700

C -4.50282100 -1.10814700 0.04042100

C -5.48334200 -0.12257800 -0.06719700

N -1.43094600 0.90090100 0.01689400

C -0.71670400 -0.19706700 0.12436900

C 0.67763500 -0.37980800 0.17407500

C 1.63177700 0.64634100 -0.02440700

C 3.09410300 0.22291300 -0.03203500

O	1.39769800	1.86135600	-0.17139200
S	-1.73063300	-1.70830400	0.17262000
C	3.53581400	-1.07008900	-0.33150600
C	4.89483100	-1.37765900	-0.32838200
C	5.83581900	-0.39632900	-0.02399000
C	5.40790400	0.89921000	0.26382600
C	4.05047000	1.20319000	0.25036900
H	-5.89307800	1.98447200	-0.23892800
H	-3.49873400	2.65626200	-0.19537600
H	-4.77805000	-2.15671800	0.10624500
H	-6.53090800	-0.40681400	-0.08583100
H	1.02631800	-1.39219200	0.33468600
H	2.81824200	-1.84119100	-0.59478900
H	5.21908600	-2.38559400	-0.57148400
H	6.89492500	-0.63721600	-0.01917500
H	6.13510000	1.67299000	0.49426900
H	3.69793900	2.20889100	0.45594600

Dihedral = 180

SCF = -1105.584

XYZ =

28

180-thiazole-Ph.com

C	-5.12183900	1.22436700	-0.11809900

C	-3.78567600	1.60322100	-0.17698600
C	-2.78004000	0.63135300	-0.07038300
C	-3.16741300	-0.72117600	0.09723000
C	-4.50325000	-1.10092200	0.15604300
C	-5.48488800	-0.11659700	0.04708800
N	-1.42944800	0.88740600	-0.11636900
C	-0.71496200	-0.20936700	0.00640200
C	0.67918200	-0.39748500	0.00965900
C	1.63262500	0.64316100	-0.09717800
C	3.09541000	0.22731500	-0.05472200
O	1.39640400	1.86249900	-0.19341600
S	-1.73011200	-1.70773000	0.19793600
C	3.56016400	-1.03741300	-0.42980400
C	4.91843700	-1.34265700	-0.37238700
C	5.83399200	-0.38786000	0.06568200
C	5.38272000	0.87978000	0.43166000
C	4.02696300	1.18334200	0.36120300
H	-5.89540600	1.98213400	-0.20429000
H	-3.50018600	2.64278400	-0.30665500
H	-4.77770700	-2.14388300	0.28448400
H	-6.53307500	-0.39585100	0.08994900
H	1.03063900	-1.41060700	0.16057000
H	2.86095900	-1.78389500	-0.79459500
H	5.26299400	-2.32669700	-0.67734400

H	6.89232900	-0.62728100	0.11404900
H	6.09020500	1.63173100	0.77024300
H	3.65739700	2.16904100	0.62608600

S•••O vs. O•••O vs. C–H•••O Interaction Model Systems:

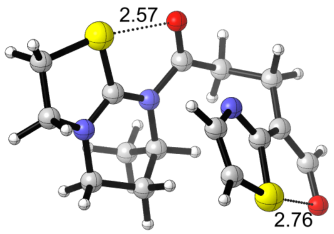

Dual S•••O
ΔΔG = 0.0

Supporting Information: Model-Thiazole-S-O-5.log

Using Gaussian 09: AM64L-G09RevD.01 24-Apr-2013

==

#m062X/6-31G(d) scf=(maxcycle=300,direct,tight) density=current

SCRF=(PCM,SOLVENT=THF) opt=(maxcycle=250,gdiis) iop(1/8=18) freq=noraman

#N Geom=AllCheck Guess=TCheck SCRF=Check Test GenChk RM062X/6-31G(d) Freq

----

Pointgroup= C1 Stoichiometry= C15H19N3O2S2 C1[X(C15H19N3O2S2)] #Atoms= 41

Charge = 0 Multiplicity = 1

SCF Energy= -1693.67041650 Predicted Change= -5.280747D-10

==

Optimization completed. {Found 2 times}

Item	Max Val.	Criteria	Pass?	RMS Val.	Criteria	Pass?
Force	0.00000 ||	0.00045	[YES]	0.00000 ||	0.00030	[YES]
Displ	0.00039 ||	0.00180	[YES]	0.00039 ||	0.00180	[YES]

Atomic Type	Coordinates (Angstroms) X	Y	Z
C	1.978031	0.247640	0.095412
S	2.984794	0.613395	-1.293715
C	4.280184	-0.512840	-0.656393
C	3.562474	-1.496256	0.249505
N	2.421202	-0.768570	0.817259
C	1.689368	-1.379630	1.930811
C	0.232427	-0.975778	1.838445
C	0.105530	0.528861	1.641795
C	0.594509	1.345606	2.833649
N	0.862793	0.925349	0.420889
C	0.357863	1.928946	-0.463212
C	-0.901926	2.666915	-0.091383
C	-2.151650	2.119137	-0.840760
C	-2.706808	0.808488	-0.363466
C	-2.045087	-0.390434	-0.743927
C	-3.883910	0.762660	0.398716

O	-4.461047	-0.258497	0.831997
O	0.962047	2.185003	-1.478897
N	-0.917454	-0.433961	-1.439318
C	-0.501060	-1.727766	-1.637450
C	-1.281634	-2.707241	-1.109859
S	-2.650954	-1.999857	-0.301675
H	5.015894	0.078384	-0.108784
H	4.761439	-1.007130	-1.499412
H	4.200034	-1.845894	1.063664
H	3.174446	-2.360495	-0.302799
H	2.158849	-1.071254	2.870290
H	1.804946	-2.462224	1.832738
H	-0.294738	-1.262046	2.751285
H	-0.234573	-1.493201	0.995674
H	0.454033	2.416072	2.660217
H	1.659221	1.175454	3.022362
H	0.032750	1.063206	3.728090
H	-0.943318	0.737252	1.426677
H	-1.086939	2.698555	0.982895
H	-1.889494	2.074280	-1.905266
H	-2.924624	2.888235	-0.730677
H	-4.326719	1.760006	0.621792
H	-0.718418	3.690239	-0.430913
H	0.412570	-1.900146	-2.199295

H -1.140440 -3.777525 -1.160215

Statistical Thermodynamic Analysis

Temperature= 298.150 Kelvin Pressure= 1.00000 Atm

===

SCF Energy= -1693.67041650 Predicted Change= -5.280747D-10

Zero-point correction (ZPE)= -1693.3342 0.33612

Internal Energy (U)= -1693.3143 0.35611

Enthalpy (H)= -1693.3133 0.35705

Gibbs Free Energy (G)= -1693.3825 0.28789

Frequencies -- 25.5290 51.6458 74.4340

===

#m062X/6-31+G(d,p) scf=(maxcycle=300,direct,tight) density=current

SCRF=(PCM,SOLVENT=THF)

Pointgroup= C1 Stoichiometry= C15H19N3O2S2 C1[X(C15H19N3O2S2)] #Atoms= 41

Charge = 0 Multiplicity = 1

SCF Energy= -1693.72116434

===

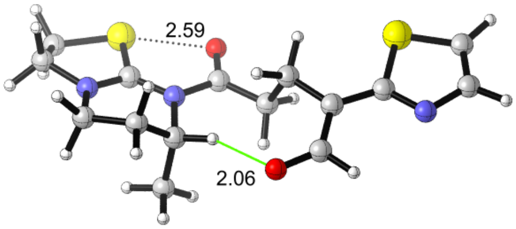

C–H•••O + S•••O
ΔΔG = 3.7

Supporting Information: Model-Thiazole-CH-O.log

Using Gaussian 09: AM64L-G09RevD.01 24-Apr-2013

==

#m062X/6-31G(d) scf=(maxcycle=300,direct,tight) density=current

SCRF=(PCM,SOLVENT=THF) opt=(maxcycle=250,gdiis) iop(1/8=18) freq=noraman

#N Geom=AllCheck Guess=TCheck SCRF=Check Test GenChk RM062X/6-31G(d) Freq

----

Pointgroup= C1 Stoichiometry= C15H19N3O2S2 C1[X(C15H19N3O2S2)] #Atoms= 41

Charge = 0 Multiplicity = 1

SCF Energy= -1693.66057232 Predicted Change= -6.594856D-09

==

Optimization completed. {Found 2 times}

Item	Max Val.	Criteria	Pass?	RMS Val.	Criteria	Pass?
Force	0.00001 ||	0.00045	[YES]	0.00000 ||	0.00030	[YES]
Displ	0.00136 ||	0.00180	[YES]	0.00136 ||	0.00180	[YES]

Atomic Coordinates (Angstroms)

Type	X	Y	Z
C	-2.908395	-0.424276	-0.013861
S	-3.423515	-2.087746	0.180780
C	-5.168721	-1.574389	-0.031454
C	-5.125663	-0.270003	-0.808326
N	-3.861789	0.382066	-0.444134
C	-3.671848	1.786178	-0.828921
C	-2.189602	2.085158	-0.938363
C	-1.439468	1.502485	0.252108
C	-1.860349	2.089778	1.595383
N	-1.664610	0.025930	0.258447
C	-0.633543	-0.891227	0.630745
C	0.723043	-0.314608	0.901432
C	1.481884	-0.068408	-0.433395
C	2.725241	0.739862	-0.211101
C	4.024899	0.157152	-0.188456
C	2.543628	2.098383	0.073257
O	1.430039	2.680230	0.149644
O	-0.869842	-2.079033	0.631963
S	4.230296	-1.606283	-0.386895
C	5.951357	-1.408690	-0.228965
C	6.231760	-0.093983	-0.044374
N	5.175617	0.780848	-0.019198

H	-5.612274	-1.442753	0.956481
H	-5.700996	-2.356597	-0.571215
H	-5.953199	0.391371	-0.544442
H	-5.124854	-0.431579	-1.892264
H	-4.169436	2.413740	-0.082842
H	-4.181459	1.926777	-1.786065
H	-2.030903	3.165140	-0.970334
H	-1.786925	1.658216	-1.862184
H	-1.255603	1.669888	2.403942
H	-2.912874	1.888895	1.821368
H	-1.705026	3.171908	1.580311
H	-0.373512	1.686663	0.098616
H	0.671328	0.625826	1.453678
H	0.819935	0.477356	-1.119910
H	1.684171	-1.038131	-0.902287
H	3.463990	2.685157	0.250056
H	1.262719	-1.051523	1.499392
H	6.629038	-2.247163	-0.286505
H	7.235913	0.299338	0.079145

Statistical Thermodynamic Analysis

Temperature= 298.150 Kelvin Pressure= 1.00000 Atm

===

SCF Energy= -1693.66057232 Predicted Change= -6.594856D-09

Zero-point correction (ZPE)= -1693.3249 0.33558

Internal Energy (U)= -1693.3044 0.35611

Enthalpy (H)= -1693.3035 0.35706

Gibbs Free Energy (G)= -1693.3758 0.28476

Frequencies -- 23.5339 34.2560 44.1627

==

#m062X/6-31+G(d,p) scf=(maxcycle=300,direct,tight) density=current

SCRF=(PCM,SOLVENT=THF)

Pointgroup= C1 Stoichiometry= C15H19N3O2S2 C1[X(C15H19N3O2S2)] #Atoms= 41

Charge = 0 Multiplicity = 1

SCF Energy= -1693.71218317

==

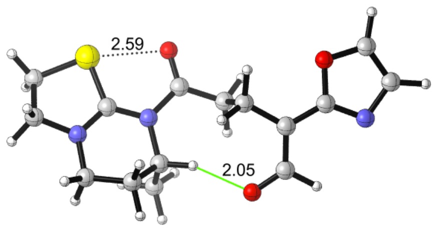

C–H···O + S···O
ΔΔG = 0.0

Supporting Information: Model-Oxazole-CH-O-1.log

Using Gaussian 09: AM64L-G09RevD.01 24-Apr-2013

==

#m062X/6-31G(d) scf=(maxcycle=300,direct,tight) density=current

SCRF=(PCM,SOLVENT=THF) opt=(maxcycle=250,gdiis) iop(1/8=18) freq=noraman

#N Geom=AllCheck Guess=TCheck SCRF=Check Test GenChk RM062X/6-31G(d) Freq

----

Pointgroup= C1 Stoichiometry= C15H19N3O3S C1[X(C15H19N3O3S)] #Atoms= 41

Charge = 0 Multiplicity = 1

SCF Energy= -1370.69489759 Predicted Change= -1.090137D-08

==

Optimization completed. {Found 1 times}

Item	Max Val.	Criteria	Pass?	RMS Val.	Criteria	Pass?
Force	0.00001 ||	0.00045	[YES]	0.00000 ||	0.00030	[YES]
Displ	0.00299 ||	0.00180	[NO]	0.00299 ||	0.00180	[YES]

Atomic Type	Coordinates (Angstroms) X	Y	Z
C	-2.694427	-0.399751	-0.024057
S	-3.283950	-2.038827	0.172746
C	-5.004071	-1.447141	-0.039997
C	-4.902404	-0.148633	-0.820373
N	-3.610450	0.447307	-0.458073
C	-3.355494	1.839823	-0.848835

C	-1.860647	2.073098	-0.949587
C	-1.143697	1.460439	0.246173
C	-1.545788	2.068009	1.586062
N	-1.433351	-0.004651	0.251451
C	-0.443847	-0.966151	0.626831
C	0.933068	-0.446852	0.906498
C	1.710043	-0.225125	-0.423403
C	2.969552	0.553899	-0.188044
C	4.252954	-0.072312	-0.221493
C	2.840934	1.900677	0.141528
O	1.750121	2.526023	0.251043
O	-0.732209	-2.142744	0.624071
O	4.294607	-1.418622	-0.459944
C	5.626196	-1.755739	-0.439172
C	6.320740	-0.626765	-0.192726
N	5.452224	0.444682	-0.054911
H	-5.440910	-1.293360	0.947757
H	-5.571623	-2.205552	-0.577836
H	-5.699542	0.549989	-0.558909
H	-4.909149	-0.313599	-1.903755
H	-3.829247	2.493152	-0.109427
H	-3.852611	1.997625	-1.809895
H	-1.654795	3.145053	-0.982523
H	-1.471413	1.627166	-1.870166

H	-0.972363	1.614833	2.399462
H	-2.609838	1.925390	1.802288
H	-1.329940	3.139739	1.573636
H	-0.068953	1.599220	0.103816
H	0.915681	0.492511	1.462883
H	1.064362	0.330916	-1.118339
H	1.906940	-1.199686	-0.877995
H	3.786303	2.447246	0.324873
H	1.439523	-1.208293	1.502113
H	5.871368	-2.790246	-0.613526
H	7.391628	-0.504348	-0.105450

Statistical Thermodynamic Analysis

Temperature= 298.150 Kelvin Pressure= 1.00000 Atm

==

SCF Energy= -1370.69489759 Predicted Change= -1.090137D-08

Zero-point correction (ZPE)= -1370.3558 0.33907

Internal Energy (U)= -1370.3357 0.35915

Enthalpy (H)= -1370.3347 0.36010

Gibbs Free Energy (G)= -1370.4064 0.28846

Frequencies -- 18.3608 30.2943 49.2790

==

#m062X/6-31+G(d,p) scf=(maxcycle=300,direct,tight) density=current

SCRF=(PCM,SOLVENT=THF)

Pointgroup= C1 Stoichiometry= C15H19N3O3S C1[X(C15H19N3O3S)] #Atoms= 41

Charge = 0 Multiplicity = 1

SCF Energy= -1370.74913825

==

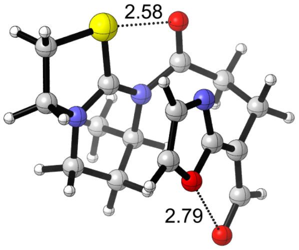

S···O + O···O
ΔΔG = 2.5

Supporting Information: Model-Oxazole-O-O-1.log

Using Gaussian 09: AM64L-G09RevD.01 24-Apr-2013

==

#m062X/6-31G(d) scf=(maxcycle=300,direct,tight) density=current

SCRF=(PCM,SOLVENT=THF) opt=(maxcycle=250,gdiis) iop(1/8=18) freq=noraman

#N Geom=AllCheck Guess=TCheck SCRF=Check Test GenChk RM062X/6-31G(d) Freq

----

Pointgroup= C1 Stoichiometry= C15H19N3O3S C1[X(C15H19N3O3S)] #Atoms= 41

Charge = 0 Multiplicity = 1

SCF Energy= -1370.69724354 Predicted Change= -1.756837D-08

==

Optimization completed. {Found 2 times}

Item	Max Val.	Criteria	Pass?	RMS Val.	Criteria	Pass?
Force	0.00006 ||	0.00045	[YES]	0.00000 ||	0.00030	[YES]
Displ	0.00137 ||	0.00180	[YES]	0.00137 ||	0.00180	[YES]

Atomic Type	Coordinates (Angstroms) X	Y	Z
C	-1.387279	-0.479784	0.106625
S	-2.523547	-0.502097	-1.225938
C	-3.724821	0.467055	-0.239149
C	-2.921547	1.146640	0.857881
N	-1.754890	0.293392	1.109842
C	-0.987008	0.528669	2.336960
C	0.426270	-0.007326	2.202280
C	0.439314	-1.340054	1.464285
C	-0.219112	-2.481248	2.235260
N	-0.244098	-1.196729	0.147067
C	0.235208	-1.807639	-1.054966
C	1.689824	-2.176444	-1.142776
C	2.532744	-0.925588	-1.543302
C	2.469817	0.239335	-0.591810

C	1.435721	1.197649	-0.804708
C	3.373886	0.306134	0.480823
O	3.427299	1.118132	1.426773
O	-0.516222	-1.931655	-1.994588
N	0.485600	1.157194	-1.722357
C	-0.294262	2.275110	-1.477294
C	0.201550	2.955781	-0.421620
O	1.315797	2.284894	0.015481
H	-4.469523	-0.218330	0.167747
H	-4.212902	1.190299	-0.891769
H	-3.492729	1.248725	1.783017
H	-2.548241	2.129286	0.547453
H	-1.537303	0.061212	3.160758
H	-0.978539	1.609817	2.505125
H	0.847218	-0.158168	3.199712
H	1.069894	0.706545	1.684815
H	-0.145855	-3.419406	1.678235
H	-1.279669	-2.277459	2.417880
H	0.278728	-2.609738	3.200111
H	1.478424	-1.573055	1.250793
H	2.080301	-2.615219	-0.223436
H	2.209646	-0.623696	-2.544994
H	3.566471	-1.279918	-1.629623
H	4.125687	-0.521871	0.453309

H 1.753446 -2.931760 -1.929748

H -1.158754 2.511320 -2.083333

H -0.052657 3.867253 0.094833

Statistical Thermodynamic Analysis

Temperature= 298.150 Kelvin Pressure= 1.00000 Atm

==

SCF Energy= -1370.69724354 Predicted Change= -1.756837D-08

Zero-point correction (ZPE)= -1370.3575 0.33973

Internal Energy (U)= -1370.3380 0.35919

Enthalpy (H)= -1370.3371 0.36013

Gibbs Free Energy (G)= -1370.4042 0.29303

Frequencies -- 53.7049 65.1210 74.1677

==

#m062X/6-31+G(d,p) scf=(maxcycle=300,direct,tight) density=current

SCRF=(PCM,SOLVENT=THF)

Pointgroup= C1 Stoichiometry= C15H19N3O3S C1[X(C15H19N3O3S)] #Atoms= 41

Charge = 0 Multiplicity = 1

SCF Energy= -1370.74968136

==

Regioselective Structures sans Ph:

We postulate that lactamization using benzothiazole nucleophile occurs to the *Re* face of the acylated HyperBTM due to unfavorable aromatic interactions that occur if cyclization occurred to the *Si* face.

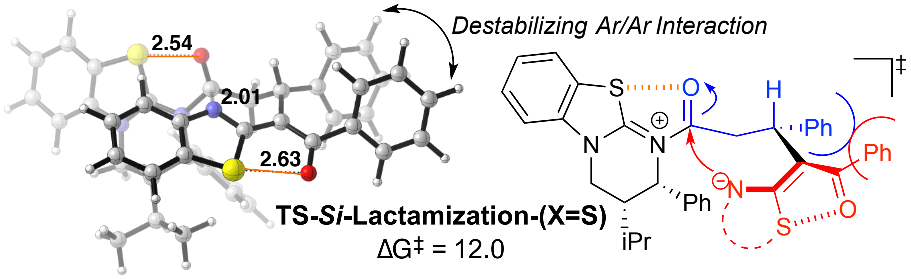

We computed truncated versions of the regioselective transition structures in which the stereogenic Ph group was removed. In these systems, *Si* face lactamization is now the favored cyclization for benzothiazole nucleophile. This suggests destabilizing Ar/Ar interactions within the *si*-face transition structure with the full system in place.

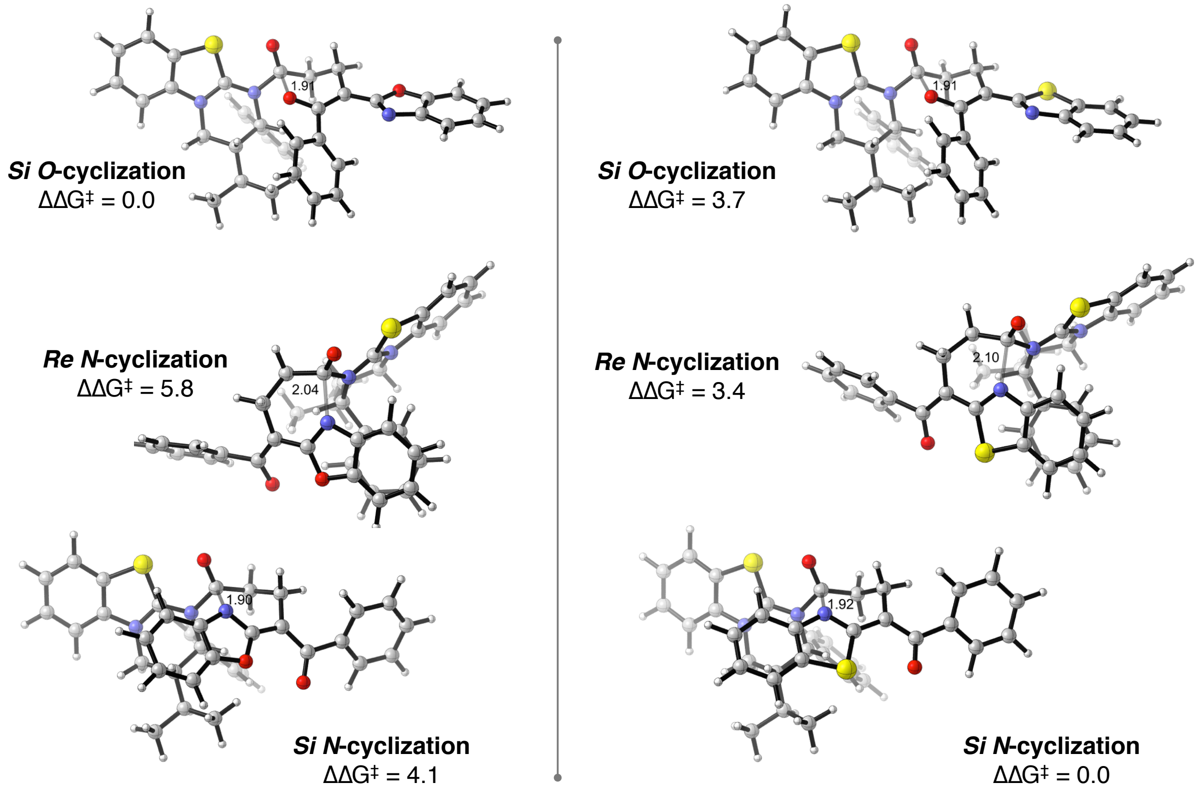

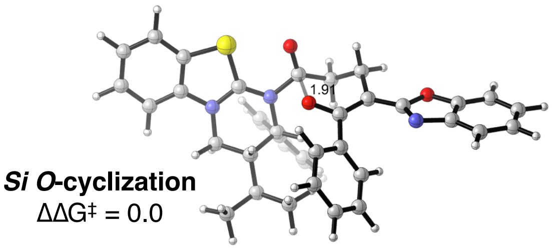

Supporting Information: Si-Lactonization_X-equals-O-sans-Ph.log

----

Using Gaussian 09: AM64L-G09RevD.01 24-Apr-2013

==

#m062X/6-31G(d) scf=(maxcycle=300,direct,tight) density=current

SCRF=(PCM,SOLVENT=THF) opt=(maxcycle=250,ts,calcfc,noeigentest,gdiis)

iop(1/8=18) freq=noraman

#N Geom=AllCheck Guess=TCheck SCRF=Check Test GenChk RM062X/6-31G(d) Freq

----

Pointgroup= C1 Stoichiometry= C37H33N3O3S C1[X(C37H33N3O3S)] #Atoms= 77

Charge = 0 Multiplicity = 1

SCF Energy= -2217.17775634 Predicted Change= -7.673662D-10

==

Optimization completed. {Found 2 times}

Item	Max Val.	Criteria	Pass?	RMS Val.	Criteria	Pass?
Force	0.00001 ||	0.00045	[YES]	0.00000 ||	0.00030	[YES]
Displ	0.00083 ||	0.00180	[YES]	0.00083 ||	0.00180	[YES]

Atomic Type	Coordinates (Angstroms) X	Y	Z
N	-3.728984	0.057770	-0.345750
C	-2.764503	-0.834848	-0.632189
N	-1.582360	-0.813955	-0.032468
C	-0.501707	-1.701027	-0.526953
C	0.531926	-2.110153	0.500662
O	-0.814028	-2.460292	-1.437959
C	-1.281527	0.241691	0.954932
C	-1.591411	-0.214554	2.369237
C	-1.994963	1.537384	0.521255
C	-1.659687	2.751196	1.406271
C	-0.179357	3.121031	1.277226
C	-2.520570	3.963363	1.034558
C	-3.491116	1.271367	0.443280
C	-4.959541	-0.203527	-0.962641
C	-4.897040	-1.323231	-1.788789
S	-3.285357	-2.034616	-1.795725
C	-6.136194	0.524308	-0.809335
C	-7.252893	0.099208	-1.520988
C	-7.194526	-1.018275	-2.360038
C	-6.014552	-1.740896	-2.504084

C	-0.745339	0.180009	3.409926
C	-1.014703	-0.188033	4.724988
C	-2.133480	-0.965212	5.016335
C	-2.974217	-1.375842	3.985256
C	-2.703568	-1.005857	2.669845
C	1.887091	-2.303701	-0.185688
C	2.516790	-0.978360	-0.550281
C	3.949640	-0.861884	-0.504115
C	1.678455	0.021094	-1.001599
C	2.051357	1.465821	-1.144263
O	0.423203	-0.203117	-1.257634
O	4.602628	-1.806460	0.270472
C	5.928087	-1.519497	0.136524
C	6.029393	-0.413506	-0.713261
N	4.756252	-0.035425	-1.113831
C	7.013462	-2.162342	0.703789
C	8.266661	-1.637417	0.383617
C	8.398515	-0.529838	-0.466535
C	7.288817	0.098353	-1.027701
C	2.846624	2.120729	-0.199152
C	3.064433	3.491345	-0.287818
C	2.494785	4.226076	-1.327706
C	1.694908	3.582927	-2.269968
C	1.463917	2.213023	-2.168627

H	0.628434	-1.399686	1.324243
H	0.172189	-3.057534	0.916712
H	-0.212200	0.422524	0.854572
H	-1.629219	1.745702	-0.495261
H	0.047968	3.995141	1.895150
H	0.068225	3.366696	0.236913
H	0.494073	2.317036	1.587196
H	-1.874349	2.486538	2.450575
H	-3.580015	3.809289	1.260037
H	-2.424603	4.193635	-0.033491
H	-2.191425	4.841705	1.596976
H	-4.004028	2.084317	-0.074022
H	-3.934143	1.147881	1.439274
H	-6.186678	1.386333	-0.153325
H	-8.184286	0.645682	-1.419162
H	-8.079563	-1.327903	-2.905232
H	-5.966261	-2.608907	-3.153166
H	0.138729	0.772933	3.187304
H	-0.344842	0.125366	5.519491
H	-2.343755	-1.256759	6.040473
H	-3.841194	-1.991938	4.202037
H	-3.362106	-1.355783	1.878921
H	1.727475	-2.929978	-1.074132
H	2.546981	-2.860367	0.482583

H	6.894997	-3.018427	1.358776
H	9.156255	-2.096988	0.802061
H	9.391832	-0.153738	-0.691387
H	7.391016	0.953804	-1.687308
H	3.285382	1.551367	0.614838
H	3.675308	3.989534	0.459203
H	2.668558	5.295746	-1.398276
H	1.243572	4.148526	-3.079899
H	0.820790	1.706778	-2.881587

Statistical Thermodynamic Analysis

Temperature= 298.150 Kelvin Pressure= 1.00000 Atm

==

SCF Energy= -2217.17775634 Predicted Change= -7.673662D-10

Zero-point correction (ZPE)= -2216.5473 0.63037

Internal Energy (U)= -2216.5119 0.66579

Enthalpy (H)= -2216.5110 0.66674

Gibbs Free Energy (G)= -2216.6160 0.56170

Frequencies -- -124.4810 13.0337 19.1972

==

#m062X/6-31+G(d,p) scf=(maxcycle=300,direct,tight) density=current

SCRF=(PCM,SOLVENT=THF)

Pointgroup= C1 Stoichiometry= C37H33N3O3S C1[X(C37H33N3O3S)] #Atoms= 77

Charge = 0 Multiplicity = 1

SCF Energy= -2217.26547066

==

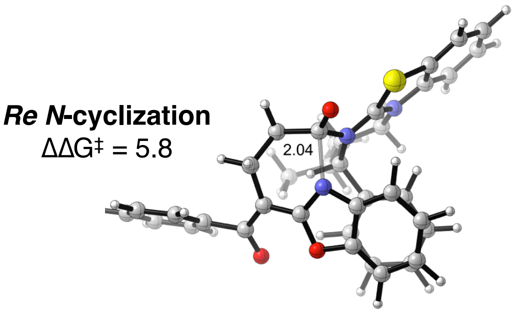

Supporting Information: Re-Lactamization_X-equals-O-sans-Ph.log

----

Using Gaussian 09: AM64L-G09RevD.01 24-Apr-2013

==

#m062X/6-31G(d) scf=(maxcycle=300,direct,tight) density=current

SCRF=(PCM,SOLVENT=THF) opt=(maxcycle=250,ts,calcfc,noeigentest,gdiis)

iop(1/8=18) freq=noraman

#N Geom=AllCheck Guess=TCheck SCRF=Check Test GenChk RM062X/6-31G(d) Freq

----

Pointgroup= C1 Stoichiometry= C37H33N3O3S C1[X(C37H33N3O3S)] #Atoms= 77

Charge = 0 Multiplicity = 1

SCF Energy= -2217.17047877 Predicted Change= -1.853743D-08

==

Optimization completed. {Found 1 times}

Item	Max Val.	Criteria	Pass?	RMS Val.	Criteria	Pass?
Force	0.00002 ||	0.00045	[YES]	0.00000 ||	0.00030	[YES]
Displ	0.00226 ||	0.00180	[NO]	0.00226 ||	0.00180	[YES]

Atomic Type	Coordinates (Angstroms) X	Y	Z
N	2.839434	-1.377937	0.099968
C	2.192196	-0.755173	-0.900872
N	0.899296	-0.466526	-0.827101
C	0.142745	-0.158165	-2.078370
C	-1.148739	-0.976868	-2.201623
O	0.824529	0.142309	-3.047029
C	0.213826	-0.658431	0.465224
C	0.421603	0.495135	1.435302
C	0.660146	-2.024315	1.029018
C	-0.115716	-2.463059	2.282335
C	-1.603617	-2.646554	1.962542
C	0.454277	-3.764506	2.854816
C	2.164559	-1.967860	1.266354
C	4.218091	-1.516339	-0.107401
C	4.610892	-1.013089	-1.346229

S	3.245321	-0.343617	-2.233706
C	5.136105	-2.083576	0.771959
C	6.465236	-2.139016	0.366960
C	6.863724	-1.645649	-0.879716
C	5.939616	-1.079051	-1.751769
C	1.620341	1.208275	1.545375
C	1.764790	2.205426	2.505712
C	0.714813	2.504045	3.370472
C	-0.490751	1.820222	3.248207
C	-0.638745	0.826367	2.283384
C	-2.496690	-0.224618	-2.171438
C	-2.771044	0.537805	-0.894307
C	-1.801408	1.522972	-0.670497
C	-3.774836	0.233463	0.073555
C	-4.931380	-0.651490	-0.331424
O	-3.763178	0.636653	1.253334
O	-1.908976	2.557544	0.216425
C	-0.752691	3.283265	0.072671
C	0.026716	2.674080	-0.917372
N	-0.670840	1.570997	-1.373363
C	-0.360258	4.411724	0.761478
C	0.893041	4.932977	0.422588
C	1.691829	4.333332	-0.557347
C	1.274120	3.194293	-1.248737

C	-5.601103	-0.484684	-1.546555
C	-6.713266	-1.264652	-1.854708
C	-7.160244	-2.227860	-0.952363
C	-6.503232	-2.394354	0.266649
C	-5.404725	-1.599357	0.579861
H	-1.043218	-1.462885	-3.176272
H	-1.159831	-1.772948	-1.450090
H	-0.850743	-0.702569	0.233783
H	0.469282	-2.767549	0.239859
H	-1.735586	-3.400022	1.176123
H	-2.091403	-1.723654	1.630992
H	-2.137968	-2.994399	2.851759
H	-0.011094	-1.676993	3.041436
H	1.474037	-3.642116	3.232038
H	0.461362	-4.553704	2.093117
H	-0.163303	-4.109173	3.689067
H	2.579277	-2.970089	1.393366
H	2.406542	-1.366241	2.150887
H	4.829212	-2.462336	1.740623
H	7.202633	-2.571983	1.034099
H	7.906880	-1.700646	-1.171466
H	6.246081	-0.693759	-2.718488
H	2.446947	1.021073	0.865997
H	2.697410	2.757765	2.565828

H	0.828919	3.284595	4.116619
H	-1.331172	2.071401	3.887956
H	-1.611566	0.356997	2.150024
H	-3.268940	-0.976684	-2.342848
H	-2.507108	0.454914	-3.034601
H	-0.984017	4.855683	1.529464
H	1.251313	5.821073	0.933420
H	2.659494	4.768747	-0.788032
H	1.885934	2.726163	-2.013491
H	-5.257465	0.274629	-2.244671
H	-7.232282	-1.117786	-2.797144
H	-8.023200	-2.840917	-1.194489
H	-6.855389	-3.137775	0.975790
H	-4.901745	-1.697011	1.537876

Statistical Thermodynamic Analysis

Temperature= 298.150 Kelvin Pressure= 1.00000 Atm

==

SCF Energy= -2217.17047877 Predicted Change= -1.853743D-08

Zero-point correction (ZPE)= -2216.5398 0.63061

Internal Energy (U)= -2216.5046 0.66583

Enthalpy (H)= -2216.5036 0.66678

Gibbs Free Energy (G)= -2216.6058 0.56464

Frequencies -- -136.0052 19.0856 26.5849

==

#m062X/6-31+G(d,p) scf=(maxcycle=300,direct,tight) density=current

SCRF=(PCM,SOLVENT=THF)

Pointgroup= C1 Stoichiometry= C37H33N3O3S C1[X(C37H33N3O3S)] #Atoms= 77

Charge = 0 Multiplicity = 1

SCF Energy= -2217.25911334

==

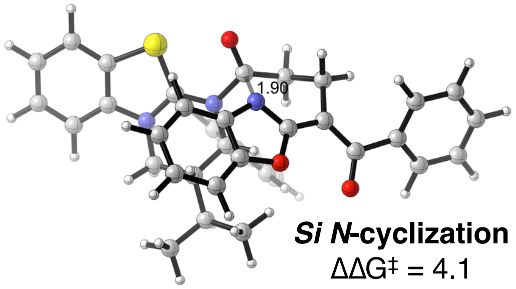

***Si N*-cyclization**
ΔΔG‡ = 4.1

Supporting Information: Si-Lactamization_X-equals-O-OO-Syn-sans-Ph.log

----

Using Gaussian 09: AM64L-G09RevD.01 24-Apr-2013

==

#m062X/6-31G(d) scf=(maxcycle=300,direct,tight) density=current

SCRF=(PCM,SOLVENT=THF) opt=(maxcycle=250,ts,calcfc,noeigentest)

freq=noraman

#N Geom=AllCheck Guess=TCheck SCRF=Check GenChk RM062X/6-31G(d) Freq

----

Pointgroup= C1 Stoichiometry= C37H33N3O3S C1[X(C37H33N3O3S)] #Atoms= 77

Charge = 0 Multiplicity = 1

SCF Energy= -2217.17303207 Predicted Change= -8.952741D-09

==

Optimization completed. {Found 2 times}

Item	Max Val.	Criteria	Pass?	RMS Val.	Criteria	Pass?
Force	0.00002 ||	0.00045	[YES]	0.00000 ||	0.00030	[YES]
Displ	0.00090 ||	0.00180	[YES]	0.00090 ||	0.00180	[YES]

Atomic Type	Coordinates (Angstroms) X	Y	Z
N	3.064891	0.472331	0.060699
C	2.196138	0.217468	-0.936455
N	0.884770	0.282056	-0.766877
C	-0.041346	-0.185793	-1.871775
C	-1.338562	0.628248	-1.932761
O	0.537996	-0.541882	-2.899674
C	0.354125	0.697118	0.546618
C	0.163735	2.203794	0.632704
C	1.251472	0.114360	1.659767
C	0.716046	0.379056	3.078597
C	-0.611642	-0.349028	3.313025

C	1.725900	-0.075748	4.137718
C	2.666271	0.638769	1.463616
C	4.409341	0.488321	-0.334210
C	4.556582	0.193925	-1.687558
S	3.001852	-0.105411	-2.460490
C	5.508951	0.759140	0.475248
C	6.768619	0.715625	-0.113301
C	6.923902	0.410172	-1.468867
C	5.818039	0.144304	-2.270449
C	1.055066	3.111314	0.054839
C	0.851924	4.483232	0.189623
C	-0.245922	4.964797	0.897077
C	-1.148395	4.066892	1.463279
C	-0.945981	2.696851	1.326553
C	-2.631297	-0.208596	-1.952665
C	-2.877163	-0.889121	-0.624577
C	-1.795698	-1.652981	-0.230178
C	-4.002277	-0.669813	0.248488
C	-5.284210	-0.130834	-0.339008
O	-3.986502	-0.907530	1.463231
O	-1.721933	-2.413580	0.906897
C	-0.470447	-2.977917	0.896640
C	0.194191	-2.563456	-0.262091
N	-0.663506	-1.730503	-0.961690

C	0.106331	-3.775700	1.862520
C	1.432395	-4.164173	1.631068
C	2.112210	-3.769589	0.475230
C	1.504086	-2.970176	-0.497797
C	-5.747568	-0.521029	-1.598344
C	-6.977004	-0.065969	-2.069251
C	-7.748132	0.792492	-1.288176
C	-7.295786	1.179671	-0.026860
C	-6.076595	0.708309	0.448866
H	-1.401527	1.355227	-1.120679
H	-1.246660	1.191280	-2.866516
H	-0.628479	0.230630	0.627342
H	1.277904	-0.974116	1.508905
H	-0.449186	-1.432804	3.290683
H	-1.388709	-0.126289	2.574895
H	-1.009773	-0.089326	4.298515
H	0.561175	1.460196	3.193326
H	1.995437	-1.128154	3.982584
H	1.286977	0.013956	5.135572
H	2.642977	0.520868	4.128092
H	3.376514	0.063474	2.061024
H	2.749714	1.698591	1.736224
H	5.391264	1.003760	1.525109
H	7.642598	0.926124	0.493599

H	7.917197	0.381497	-1.903594
H	5.933919	-0.091618	-3.323075
H	1.908814	2.762438	-0.520164
H	1.552815	5.175493	-0.266118
H	-0.403725	6.033906	0.997861
H	-2.017269	4.430492	2.002741
H	-1.665333	1.999754	1.751025
H	-2.551288	-0.938982	-2.770464
H	-3.454337	0.466145	-2.195233
H	-0.437593	-4.073670	2.752253
H	1.934691	-4.790040	2.361134
H	3.135557	-4.098568	0.324234
H	2.022679	-2.678688	-1.405278
H	-5.151412	-1.200566	-2.201883
H	-7.334459	-0.385230	-3.043494
H	-8.703065	1.153548	-1.658030
H	-7.898999	1.841835	0.587027
H	-5.721433	0.978543	1.438745

Statistical Thermodynamic Analysis

Temperature= 298.150 Kelvin Pressure= 1.00000 Atm

===

SCF Energy= -2217.17303207 Predicted Change= -8.952741D-09

Zero-point correction (ZPE)= -2216.5422 0.63083

Internal Energy (U)= -2216.5070 0.66596

Enthalpy (H)= -2216.5061 0.66690

Gibbs Free Energy (G)= -2216.6082 0.56477

Frequencies -- -192.8753 23.8575 30.0107

==

#m062X/6-31+G(d,p) scf=(maxcycle=300,direct,tight) density=current

SCRF=(PCM,SOLVENT=THF)

Pointgroup= C1 Stoichiometry= C37H33N3O3S C1[X(C37H33N3O3S)] #Atoms= 77

Charge = 0 Multiplicity = 1

SCF Energy= -2217.26194638

==

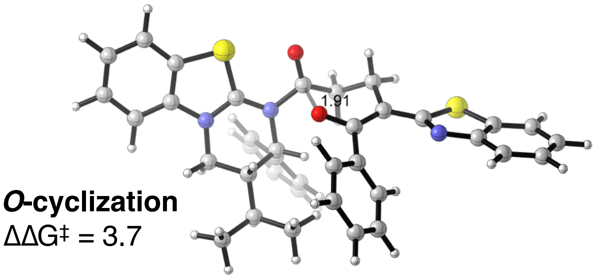

***Si O*-cyclization**
$\Delta\Delta G^{\ddagger}$ = 3.7

Supporting Information: Si-Lactonization_X-equals-S-sans-Ph.log

----

Using Gaussian 09: AM64L-G09RevD.01 24-Apr-2013

==

#m062X/6-31G(d) scf=(maxcycle=300,direct,tight) density=current

SCRF=(PCM,SOLVENT=THF) opt=(maxcycle=250,ts,calcfc,noeigentest,gdiis)

iop(1/8=18) freq=noraman

#N Geom=AllCheck Guess=TCheck SCRF=Check Test GenChk RM062X/6-31G(d) Freq

----

Pointgroup= C1 Stoichiometry= C37H33N3O2S2 C1[X(C37H33N3O2S2)] #Atoms= 77

Charge = 0 Multiplicity = 1

SCF Energy= -2540.14063115 Predicted Change= -7.318370D-10

==

Optimization completed. {Found 2 times}

Item	Max Val.		Criteria	Pass?	RMS Val.		Criteria	Pass?
Force	0.00000	||	0.00045	[YES]	0.00000	||	0.00030	[YES]
Displ	0.00090	||	0.00180	[YES]	0.00090	||	0.00180	[YES]

Atomic Type	Coordinates (Angstroms) X	Y	Z
N	-3.885589	0.108613	-0.387895
C	-2.932103	-0.801920	-0.653342
N	-1.754906	-0.790020	-0.043040
C	-0.687708	-1.707799	-0.507451
C	0.318781	-2.127337	0.541926
O	-1.001302	-2.470947	-1.414579
C	-1.447772	0.271650	0.936303

C	-1.776241	-0.165531	2.352464
C	-2.140099	1.572595	0.483710
C	-1.799268	2.789583	1.362262
C	-0.311960	3.136407	1.251874
C	-2.636654	4.010603	0.966732
C	-3.638774	1.325817	0.392559
C	-5.114495	-0.142853	-1.012130
C	-5.061654	-1.274824	-1.822096
S	-3.460830	-2.010079	-1.804566
C	-6.281433	0.604316	-0.879290
C	-7.398232	0.185746	-1.594654
C	-7.349459	-0.944414	-2.417251
C	-6.179167	-1.686235	-2.541030
C	-0.931857	0.224827	3.396136
C	-1.218223	-0.125108	4.712571
C	-2.352529	-0.879912	5.002500
C	-3.191646	-1.286883	3.968647
C	-2.904022	-0.934873	2.651832
C	1.676344	-2.368693	-0.124261
C	2.359457	-1.071361	-0.492813
C	3.796318	-0.976546	-0.422534
C	1.549124	-0.051327	-0.957351
C	1.955554	1.386303	-1.082578
O	0.293658	-0.244338	-1.236019

S	4.697190	-2.129391	0.629435
C	6.185428	-1.358963	0.156624
C	5.896266	-0.312535	-0.744864
N	4.565088	-0.143543	-1.062518
C	7.492130	-1.654258	0.539764
C	8.522171	-0.878549	0.019645
C	8.249655	0.168611	-0.872419
C	6.948825	0.455593	-1.259936
C	2.736823	2.017710	-0.109667
C	2.986789	3.383378	-0.181756
C	2.465068	4.137062	-1.233610
C	1.679705	3.517719	-2.203200
C	1.414899	2.152303	-2.117772
H	0.424993	-1.405630	1.354416
H	-0.071240	-3.057763	0.968664
H	-0.375174	0.437059	0.842524
H	-1.761479	1.768313	-0.530415
H	-0.081649	4.014718	1.862722
H	-0.044216	3.365699	0.212848
H	0.344779	2.326978	1.582153
H	-2.032775	2.537443	2.405565
H	-3.701428	3.874852	1.178322
H	-2.521844	4.228866	-0.101953
H	-2.302336	4.889428	1.525325

H	-4.135747	2.140982	-0.136687
H	-4.093087	1.217075	1.385264
H	-6.324465	1.476360	-0.236226
H	-8.322055	0.747640	-1.509052
H	-8.234347	-1.248726	-2.965640
H	-6.138340	-2.564112	-3.177184
H	-0.035701	0.799893	3.175354
H	-0.549521	0.184859	5.509383
H	-2.576076	-1.157209	6.027772
H	-4.070628	-1.886070	4.184376
H	-3.561793	-1.281857	1.858977
H	1.513552	-3.003516	-1.005966
H	2.294572	-2.950694	0.566187
H	7.700726	-2.465363	1.230297
H	9.547293	-1.088083	0.308321
H	9.069270	0.761863	-1.266175
H	6.726123	1.262147	-1.951205
H	3.140144	1.431724	0.711384
H	3.586012	3.863319	0.586416
H	2.664882	5.202913	-1.291918
H	1.266092	4.098351	-3.022651
H	0.782451	1.665175	-2.853426

Statistical Thermodynamic Analysis

Temperature= 298.150 Kelvin Pressure= 1.00000 Atm

==

SCF Energy= -2540.14063115 Predicted Change= -7.318370D-10

Zero-point correction (ZPE)= -2539.5137 0.62692

Internal Energy (U)= -2539.4776 0.66300

Enthalpy (H)= -2539.4766 0.66395

Gibbs Free Energy (G)= -2539.5836 0.55701

Frequencies -- -130.9206 10.7746 16.4782

==

#m062X/6-31+G(d,p) scf=(maxcycle=300,direct,tight) density=current

SCRF=(PCM,SOLVENT=THF)

Pointgroup= C1 Stoichiometry= C37H33N3O2S2 C1[X(C37H33N3O2S2)] #Atoms= 77

Charge = 0 Multiplicity = 1

SCF Energy= -2540.22722309

==

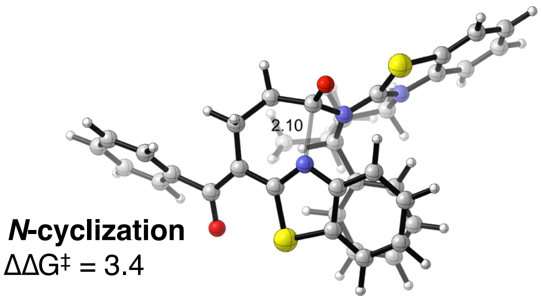

***Re N*-cyclization**
ΔΔG‡ = 3.4

Supporting Information: Re-Lactamization_X-equals-S-sans-Ph.log

----

Using Gaussian 09: AM64L-G09RevD.01 24-Apr-2013

==

#m062X/6-31G(d) scf=(maxcycle=300,direct,tight) density=current

SCRF=(PCM,SOLVENT=THF) opt=(maxcycle=250,ts,calcfc,noeigentest,gdiis)

iop(1/8=18) freq=noraman

#N Geom=AllCheck Guess=TCheck SCRF=Check Test GenChk RM062X/6-31G(d) Freq

----

Pointgroup= C1 Stoichiometry= C37H33N3O2S2 C1[X(C37H33N3O2S2)] #Atoms= 77

Charge = 0 Multiplicity = 1

SCF Energy= -2540.14252771 Predicted Change= -1.841882D-09

==

Optimization completed. {Found 2 times}

Item	Max Val.	Criteria	Pass?	RMS Val.	Criteria	Pass?
Force	0.00000 ||	0.00045	[YES]	0.00000 ||	0.00030	[YES]
Displ	0.00104 ||	0.00180	[YES]	0.00104 ||	0.00180	[YES]

Atomic Type	Coordinates (Angstroms) X	Y	Z
N	2.940891	-1.382029	0.137879
C	2.224423	-0.849958	-0.865833

N	0.930614	-0.572499	-0.732380
C	0.090231	-0.426505	-1.950361
C	-1.179206	-1.277258	-1.918076
O	0.686335	-0.179047	-2.985387
C	0.337478	-0.675652	0.614119
C	0.614830	0.530186	1.499530
C	0.830399	-2.008411	1.224235
C	0.134948	-2.405245	2.537039
C	-1.353069	-2.679438	2.296968
C	0.788678	-3.644379	3.156998
C	2.344359	-1.913853	1.373358
C	4.306947	-1.516990	-0.141765
C	4.615692	-1.105946	-1.436995
S	3.185402	-0.535438	-2.289446
C	5.285863	-2.003756	0.720214
C	6.589075	-2.069962	0.240346
C	6.903859	-1.665368	-1.061321
C	5.919236	-1.181891	-1.916661
C	1.789452	1.289055	1.452409
C	1.990197	2.333537	2.349475
C	1.026818	2.632934	3.310414
C	-0.150115	1.893351	3.355963
C	-0.354560	0.855675	2.450370
C	-2.522218	-0.540266	-2.068790

C	-2.880960	0.370480	-0.921105
C	-1.937350	1.381899	-0.683698
C	-4.054677	0.313452	-0.119862
C	-5.131834	-0.703665	-0.407634
O	-4.250884	1.074325	0.852853
S	-2.149695	2.767867	0.407851
C	-0.564964	3.353760	-0.067134
C	0.013390	2.474204	-1.003650
N	-0.771228	1.388558	-1.324741
C	0.122740	4.476500	0.381837
C	1.398662	4.723215	-0.120105
C	1.973216	3.859043	-1.060101
C	1.290020	2.737099	-1.514122
C	-5.685345	-0.879700	-1.678955
C	-6.733810	-1.776790	-1.874770
C	-7.232764	-2.513457	-0.803027
C	-6.693332	-2.335031	0.470823
C	-5.660256	-1.424378	0.667025
H	-1.049414	-1.945020	-2.776146
H	-1.208498	-1.911123	-1.027425
H	-0.740974	-0.718234	0.461002
H	0.611841	-2.791660	0.482272
H	-1.476474	-3.521272	1.604940
H	-1.890735	-1.822745	1.877111

H	-1.844310	-2.944654	3.237555
H	0.236282	-1.573563	3.246007
H	1.813715	-3.453077	3.488006
H	0.806857	-4.474328	2.440164
H	0.218040	-3.969609	4.031459
H	2.785403	-2.900217	1.531420
H	2.623757	-1.258591	2.206995
H	5.044739	-2.312945	1.731364
H	7.372970	-2.441336	0.891389
H	7.928841	-1.726817	-1.410332
H	6.159428	-0.868000	-2.926938
H	2.546002	1.105903	0.695465
H	2.899396	2.923119	2.286173
H	1.187143	3.448688	4.008640
H	-0.922515	2.131994	4.080428
H	-1.295776	0.310208	2.465881
H	-3.283000	-1.311690	-2.199668
H	-2.472262	0.030768	-3.007201
H	-0.323323	5.145138	1.112434
H	1.948392	5.596238	0.218002
H	2.965151	4.072537	-1.448004
H	1.716899	2.070594	-2.257279
H	-5.308276	-0.295352	-2.514486
H	-7.163821	-1.896890	-2.864678

H -8.043789 -3.218775 -0.957294

H -7.085118 -2.900146 1.311544

H -5.249741 -1.257379 1.658720

Statistical Thermodynamic Analysis

Temperature= 298.150 Kelvin Pressure= 1.00000 Atm

==

SCF Energy= -2540.14252771 Predicted Change= -1.841882D-09

Zero-point correction (ZPE)= -2539.5155 0.62702

Internal Energy (U)= -2539.4795 0.66295

Enthalpy (H)= -2539.4786 0.66390

Gibbs Free Energy (G)= -2539.5831 0.55939

Frequencies -- -103.9199 11.7108 21.6219

==

#m062X/6-31+G(d,p) scf=(maxcycle=300,direct,tight) density=current

SCRF=(PCM,SOLVENT=THF)

Pointgroup= C1 Stoichiometry= C37H33N3O2S2 C1[X(C37H33N3O2S2)] #Atoms= 77

Charge = 0 Multiplicity = 1

SCF Energy= -2540.23012609

==

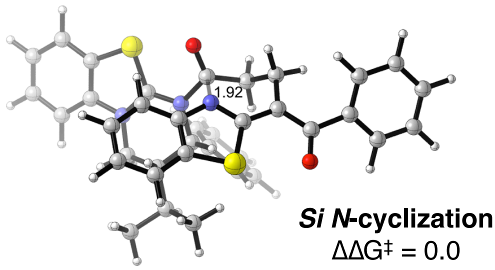

Supporting Information: Si-Lactamization_X-equals-S-sans-Ph.log

----

Using Gaussian 09: AM64L-G09RevD.01 24-Apr-2013

==

#m062X/6-31G(d) scf=(maxcycle=300,direct,tight) density=current

SCRF=(PCM,SOLVENT=THF) opt=(maxcycle=250,ts,calcfc,noeigentest)

freq=noraman

#N Geom=AllCheck Guess=TCheck SCRF=Check GenChk RM062X/6-31G(d) Freq

----

Pointgroup= C1 Stoichiometry= C37H33N3O2S2 C1[X(C37H33N3O2S2)] #Atoms= 77

Charge = 0 Multiplicity = 1

SCF Energy= -2540.14932998 Predicted Change= -2.081473D-09

==

Optimization completed. {Found 2 times}

Item	Max Val.	Criteria	Pass?	RMS Val.	Criteria	Pass?
Force	0.00001 ||	0.00045	[YES]	0.00000 ||	0.00030	[YES]
Displ	0.00140 ||	0.00180	[YES]	0.00140 ||	0.00180	[YES]

Atomic Coordinates (Angstroms)

Type	X	Y	Z
N	-3.223501	-0.445721	-0.023388
C	-2.226698	-0.444949	-0.926062
N	-0.948851	-0.434291	-0.572403
C	0.115799	-0.311266	-1.637857
C	1.331470	-1.188050	-1.362343
O	-0.323022	-0.179279	-2.782167
C	-0.597253	-0.493752	0.862938
C	-0.433223	-1.928760	1.341982
C	-1.632263	0.319294	1.674561
C	-1.327559	0.387247	3.182395
C	-0.022411	1.139508	3.450817
C	-2.466140	1.081018	3.938903
C	-3.018690	-0.250689	1.414482
C	-4.504845	-0.591826	-0.571385
C	-4.468120	-0.664632	-1.961442
S	-2.821796	-0.541772	-2.573368
C	-5.705547	-0.661921	0.129710
C	-6.875611	-0.796216	-0.610306
C	-6.846453	-0.858215	-2.007112
C	-5.640778	-0.791503	-2.698175
C	-1.273220	-2.961821	0.918005
C	-1.106622	-4.255315	1.408801

C	-0.095469	-4.534612	2.324106
C	0.756438	-3.514311	2.740388
C	0.589674	-2.223127	2.248734
C	2.650924	-0.517216	-1.772530
C	3.045317	0.534665	-0.764437
C	2.026463	1.418808	-0.435834
C	4.301562	0.641534	-0.086689
C	5.485235	-0.171553	-0.540627
O	4.474396	1.409946	0.878610
S	2.123352	2.693295	0.801011
C	0.473800	3.167105	0.412776
C	-0.034511	2.362280	-0.621936
N	0.827892	1.372103	-1.048649
C	-0.309170	4.154280	1.004216
C	-1.610274	4.344485	0.540455
C	-2.101894	3.581329	-0.525195
C	-1.320002	2.599312	-1.123202
C	5.791501	-0.364713	-1.890571
C	6.940407	-1.061074	-2.259799
C	7.787838	-1.577855	-1.282214
C	7.494629	-1.378804	0.066791
C	6.357053	-0.667428	0.433042
H	1.399973	-1.495247	-0.317258
H	1.152716	-2.088148	-1.960114

H	0.365646	0.016712	0.943820
H	-1.608423	1.344597	1.279597
H	-0.108817	2.176749	3.105882
H	0.843874	0.700362	2.948911
H	0.189532	1.153577	4.524049
H	-1.235415	-0.638985	3.562569
H	-2.670229	2.067079	3.502922
H	-2.183061	1.229845	4.984892
H	-3.393168	0.500346	3.930923
H	-3.788692	0.448531	1.746487
H	-3.166399	-1.209542	1.927922
H	-5.732170	-0.621529	1.213003
H	-7.825535	-0.854835	-0.089994
H	-7.773853	-0.961352	-2.559979
H	-5.612973	-0.839809	-3.781656
H	-2.056006	-2.777423	0.186910
H	-1.766633	-5.046163	1.066509
H	0.034801	-5.543340	2.702707
H	1.559079	-3.722916	3.440592
H	1.273019	-1.437286	2.561365
H	2.520271	-0.090856	-2.777878
H	3.417769	-1.290914	-1.841722
H	0.085939	4.764582	1.811237
H	-2.235138	5.106771	0.994478

H	-3.104054	3.765405	-0.900433
H	-1.676852	2.027363	-1.973942
H	5.140811	0.052318	-2.654415
H	7.174946	-1.196533	-3.311283
H	8.678715	-2.127919	-1.569974
H	8.158489	-1.772332	0.830704
H	6.129034	-0.480977	1.478105

Statistical Thermodynamic Analysis

Temperature= 298.150 Kelvin Pressure= 1.00000 Atm

===

SCF Energy= -2540.14932998 Predicted Change= -2.081473D-09

Zero-point correction (ZPE)= -2539.5219 0.62739

Internal Energy (U)= -2539.4861 0.66313

Enthalpy (H)= -2539.4852 0.66407

Gibbs Free Energy (G)= -2539.5892 0.56008

Frequencies -- -178.2754 19.6035 23.8722

===

#m062X/6-31+G(d,p) scf=(maxcycle=300,direct,tight) density=current

SCRF=(PCM,SOLVENT=THF)

Pointgroup= C1 Stoichiometry= C37H33N3O2S2 C1[X(C37H33N3O2S2)] #Atoms= 77

Charge = 0 Multiplicity = 1

SCF Energy= -2540.23622293

==

Aromatic interactions

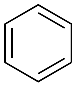

Supporting Information: Benzene.log

Using Gaussian 09: AM64L-G09RevD.01 24-Apr-2013

==

#m062X/6-31G(d) scf=(maxcycle=300,direct,tight) density=current

SCRF=(PCM,SOLVENT=THF) opt=(maxcycle=250,gdiis) iop(1/8=18) freq=noraman

#N Geom=AllCheck Guess=TCheck SCRF=Check Test GenChk RM062X/6-31G(d) Freq

----

Pointgroup= D2H Stoichiometry= C6H6 D2H[C2"(HC.CH),SG(C4H4)] #Atoms= 12

Charge = 0 Multiplicity = 1

SCF Energy= -232.138898015 Predicted Change= -7.255816D-07

==

Optimization completed. {Found 2 times}

Item Max Val. Criteria Pass? RMS Val. Criteria Pass?

Force 0.00036 || 0.00045 [YES] 0.00012 || 0.00030 [YES]

Displ 0.00077 || 0.00180 [YES] 0.00077 || 0.00180 [YES]

Atomic Type	Coordinates (Angstroms) X	Y	Z
C	0.000000	1.207438	0.697094
C	0.000000	0.000000	1.393987
C	0.000000	-1.207438	0.697094
C	0.000000	-1.207438	-0.697094
C	0.000000	0.000000	-1.393987
C	0.000000	1.207438	-0.697094
H	0.000000	2.147745	1.240405
H	0.000000	0.000000	2.479957
H	0.000000	-2.147745	1.240405
H	0.000000	-2.147745	-1.240405
H	0.000000	0.000000	-2.479957
H	0.000000	2.147745	-1.240405

Statistical Thermodynamic Analysis

Temperature= 298.150 Kelvin Pressure= 1.00000 Atm

===

SCF Energy= -232.138898015 Predicted Change= -7.255816D-07

Zero-point correction (ZPE)= -232.0373 0.10154

Internal Energy (U)= -232.0329 0.10591

Enthalpy (H)= -232.0320 0.10685

Gibbs Free Energy (G)= -232.0634 0.07539

Frequencies -- 413.7060 414.1124 615.5951

==

#m062X/6-31+G(d,p) scf=(maxcycle=300,direct,tight) density=current

SCRF=(PCM,SOLVENT=THF)

Pointgroup= D2H Stoichiometry= C6H6 D2H[C2"(HC.CH),SG(C4H4)] #Atoms= 12

Charge = 0 Multiplicity = 1

SCF Energy= -232.154583839

==

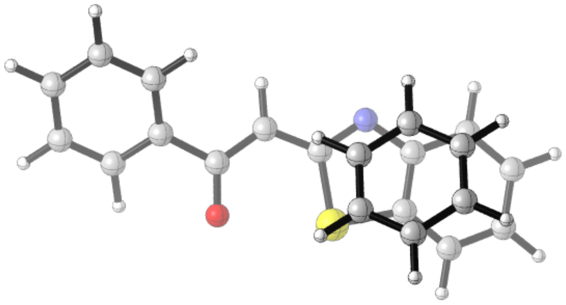

Supporting Information: Thiazole-Benzene-Direct-overlap-Stack.log

----

Using Gaussian 09: AM64L-G09RevD.01 24-Apr-2013

==

#m062X/6-31G(d) scf=(maxcycle=300,direct,tight) density=current

SCRF=(PCM,SOLVENT=THF) opt=(maxcycle=250,gdiis) iop(1/8=18) freq=noraman

#N Geom=AllCheck Guess=TCheck SCRF=Check Test GenChk RM062X/6-31G(d) Freq

----

Pointgroup= C1 Stoichiometry= C21H16NOS(1-) C1[X(C21H16NOS)] #Atoms= 40

Charge = -1 Multiplicity = 1

SCF Energy= -1337.73974902 Predicted Change= -1.887339D-08

==

Optimization completed. {Found 1 times}

Item	Max Val.	Criteria	Pass?	RMS Val.	Criteria	Pass?
Force	0.00000 ||	0.00045	[YES]	0.00000 ||	0.00030	[YES]
Displ	0.00193 ||	0.00180	[NO]	0.00193 ||	0.00180	[YES]

Atomic Type	Coordinates (Angstroms) X	Y	Z
C	3.200870	-1.497572	-1.663500
C	4.421806	-1.678108	-1.026466
C	4.509630	-1.697382	0.371805
C	3.364170	-1.540784	1.149604
C	2.138835	-1.366289	0.512663
C	2.037444	-1.332553	-0.897038
S	0.550628	-1.144299	1.212506
C	-0.115798	-0.973291	-0.447252

C	-1.479362	-0.678974	-0.654115
C	-2.400302	-0.522806	0.395200
O	-2.137328	-0.694604	1.611777
C	-3.819827	-0.140113	0.037466
N	0.779625	-1.117354	-1.406689
C	-4.836292	-0.458155	0.942810
C	-6.161242	-0.129255	0.673868
C	-6.488914	0.541622	-0.503820
C	-5.481493	0.881494	-1.404796
C	-4.157727	0.542072	-1.136093
H	3.128438	-1.471642	-2.747121
H	5.323035	-1.802044	-1.620822
H	5.473139	-1.834976	0.853011
H	3.427101	-1.556087	2.234227
H	-1.791077	-0.594647	-1.687855
H	-4.559429	-0.968027	1.860170
H	-6.940639	-0.392726	1.383516
H	-7.521460	0.804578	-0.714606
H	-5.725925	1.419935	-2.315987
H	-3.377614	0.833405	-1.833126
C	3.519922	2.179146	-0.892601
C	3.886369	2.578302	0.390790
C	2.913214	2.726762	1.378922
C	1.575315	2.476489	1.081907

C	1.208666	2.070456	-0.200395
C	2.181897	1.919891	-1.185740
H	4.278406	2.048815	-1.659177
H	4.930168	2.769078	0.623627
H	3.199847	3.032725	2.381015
H	0.817872	2.579843	1.853396
H	0.169689	1.841942	-0.423317
H	1.896556	1.571226	-2.173757

Statistical Thermodynamic Analysis

Temperature= 298.150 Kelvin Pressure= 1.00000 Atm

==

SCF Energy= -1337.73974902 Predicted Change= -1.887339D-08

Zero-point correction (ZPE)= -1337.4256 0.31411

Internal Energy (U)= -1337.4055 0.33418

Enthalpy (H)= -1337.4046 0.33512

Gibbs Free Energy (G)= -1337.4775 0.26221

Frequencies -- 24.5810 30.0592 36.8865

Reaction coordinates for 16A and 16B

Compounds **16A** (lactam) and **16B** (lactone) were unusual examples given that each had significantly different enantioselectivity (81% ee for **16A**, 31% ee for **16B**). We investigated the origin of this discrepancy but could not identify its origin. We computed TSs needed to computed enantioselectivity and chemoselectivity using the benzothiazole nucleophile. We show good agreement for the the enantioselectivity of **16A** ($\Delta\Delta G^{\ddagger}$ = 1.7 kcal/mol, 89% ee) in comparing **TS-*(Re,Si)*-Major** and **TS-*(Re,Re)*-minor.** The chemoselective transition states actually favor formation of the lactone over the lactam, in disagreement with experiments. None of these computed energetics can account for the different enantioselectivity between **16A** and **16B.**

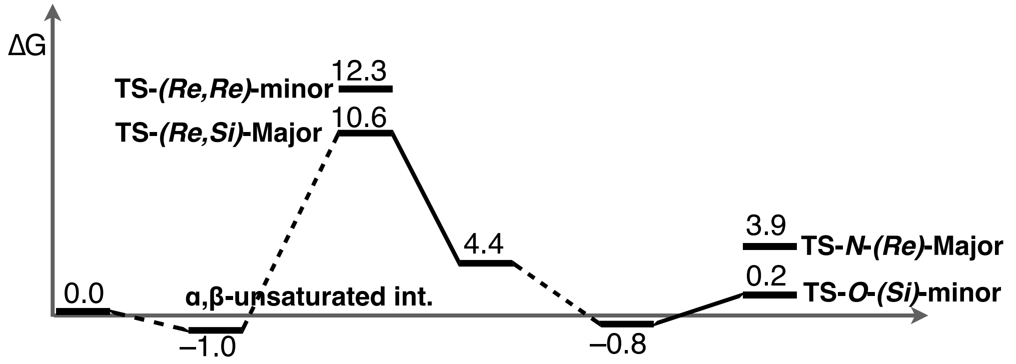

TS-*(x,y)*
x *= prochirality of nucleophile*
y *= prochirality of substrate*

(x,Si) *leads to Major enantiomer*
(x,Re) *leads to minor enantiomer*

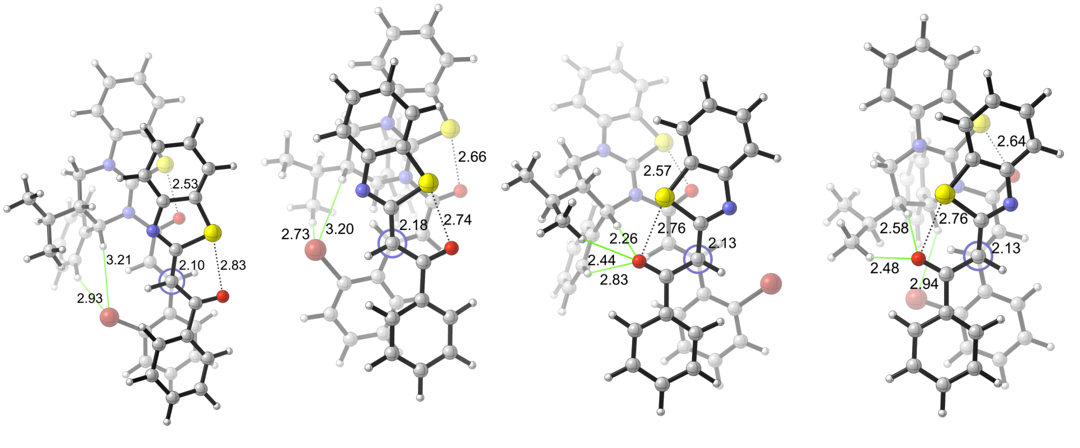

**TS-*(Re,Si)*-Major**	**TS-*(Re,Re)*-minor**	**TS-*(Si,Si)***	**TS-*(Si,Re)***
$\Delta G^{\ddagger}$ = 10.6 ($\Delta\Delta G^{\ddagger}$ = 0.0)	$\Delta G^{\ddagger}$ = 12.3 ($\Delta\Delta G^{\ddagger}$ = 1.7)<br>$\Delta\Delta G^{\ddagger}_{exp}$ = 1.3 (lactam)<br>$\Delta\Delta G^{\ddagger}_{exp}$ = 0.4 (lactone)	$\Delta G^{\ddagger}$ = 11.6	$\Delta G^{\ddagger}$ = 15.4

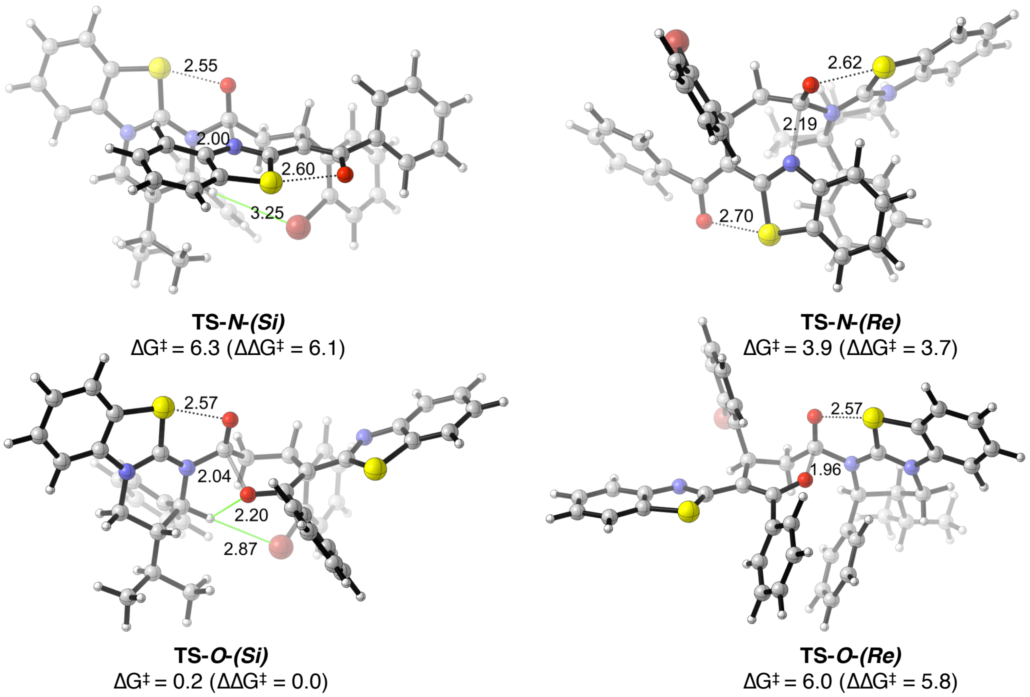

TS-*N*-(*Si*)
ΔG‡ = 6.3 (ΔΔG‡ = 6.1)

TS-*N*-(*Re*)
ΔG‡ = 3.9 (ΔΔG‡ = 3.7)

TS-*O*-(*Si*)
ΔG‡ = 0.2 (ΔΔG‡ = 0.0)

TS-*O*-(*Re*)
ΔG‡ = 6.0 (ΔΔG‡ = 5.8)

A possible mechanism involving HBTM-catalyzed ketene formation was also considered. Since cyclization would then off-cycle, this was hypothesized to account for the difference in enantioselectivity. While ketene formation is possible (**TS-Ketene-formation**, $\Delta G^{\ddagger}$ = 15.2 kcal/mol), the annulation TSs with catalyst still attached are highly favored ($\Delta G^{\ddagger}$ = 0.2 kcal/mol vs. $\Delta G^{\ddagger}$ = 15.2 kcal/mol), and the ketene formation pathway is not viable.

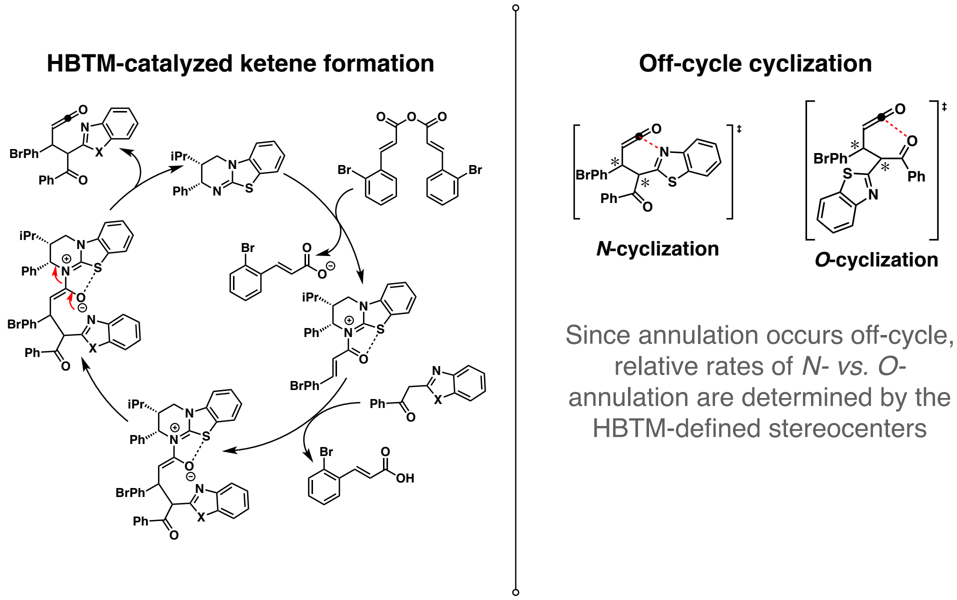

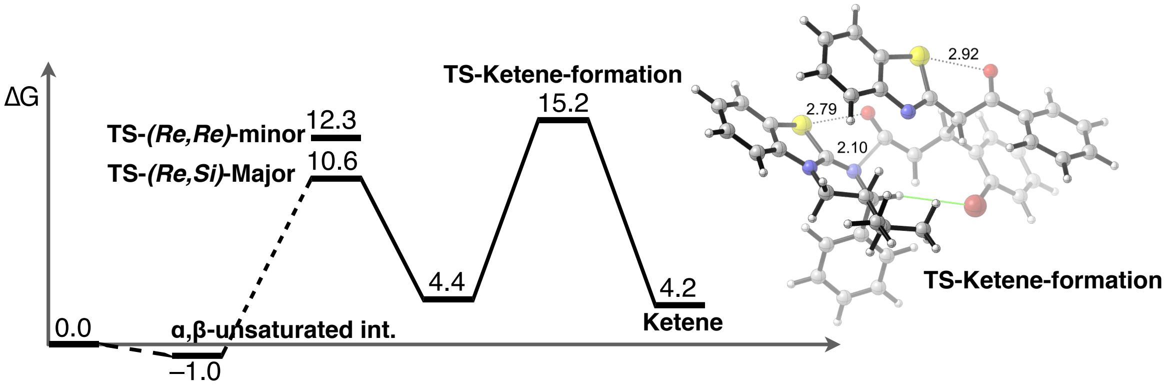

Supporting Information: 000-DiBrPh-Anhydride-004.log

Using Gaussian 09: AM64L-G09RevD.01 24-Apr-2013

==

#M062X/6-31G(d) scf=(maxcycle=300,direct,tight) density=current

SCRF=(PCM,SOLVENT=THF) opt=(maxcycle=250,gdiis) iop(1/8=18) freq=noraman

#N Geom=AllCheck Guess=TCheck SCRF=Check Test GenChk RM062X/6-31G(d) Freq

----

Pointgroup= C1 Stoichiometry= C18H12Br2O3 C1[X(C18H12Br2O3)] #Atoms= 35

Charge = 0 Multiplicity = 1

SCF Energy= -6061.99828031 Predicted Change= -1.073474D-09

==

Optimization completed. {Found 2 times}

Item	Max Val.	Criteria	Pass?	RMS Val.	Criteria	Pass?
Force	0.00000 ||	0.00045	[YES]	0.00000 ||	0.00030	[YES]

Displ 0.00100 || 0.00180 [YES] 0.00100 || 0.00180 [YES]

Atomic Type	Coordinates (Angstroms) X	Y	Z
O	-0.026762	0.273427	0.323101
C	1.195966	-0.258478	0.685075
C	2.282329	0.495037	0.032871
C	3.554074	0.179298	0.311913
C	4.735345	0.844924	-0.247534
O	1.314052	-1.168870	1.458632
C	-1.178683	-0.484454	0.392328
C	-2.357195	0.399241	0.462721
C	-3.582429	-0.136229	0.385920
C	-4.834177	0.623719	0.479721
O	-1.182250	-1.683960	0.343968
C	5.998649	0.234708	-0.250907
Br	6.234583	-1.508788	0.461381
C	7.117631	0.865726	-0.781905
C	6.993827	2.141893	-1.320912
C	5.754761	2.779725	-1.322960
C	4.647104	2.137144	-0.790289
C	-6.021127	0.176989	-0.118198
Br	-6.038993	-1.423637	-1.136219

C	-7.207826	0.893138	-0.017910
C	-7.228961	2.085817	0.697912
C	-6.066952	2.553702	1.308389
C	-4.889721	1.826945	1.199886
H	1.992936	1.269744	-0.668516
H	3.726167	-0.643032	1.002509
H	-2.176183	1.465046	0.551874
H	-3.658911	-1.211459	0.238903
H	8.075066	0.357252	-0.771238
H	7.868223	2.635389	-1.732303
H	5.654033	3.779216	-1.732060
H	3.690716	2.649986	-0.770273
H	-8.103643	0.518677	-0.500261
H	-8.156530	2.642914	0.779342
H	-6.080865	3.477185	1.877129
H	-3.992655	2.176096	1.701891

Statistical Thermodynamic Analysis

Temperature= 298.150 Kelvin   Pressure= 1.00000 Atm

===

SCF Energy=   -6061.99828031   Predicted Change= -1.073474D-09

Zero-point correction (ZPE)=   -6061.7421 0.25611

Internal Energy (U)=   -6061.7213 0.27693

Enthalpy (H)=   -6061.7204 0.27788

Gibbs Free Energy (G)= -6061.7974 0.20085

Frequencies -- 12.9242 22.0654 26.1257

==

#M062X/6-31+G(d,p) scf=(maxcycle=300,direct,tight) density=current

SCRF=(PCM,SOLVENT=THF)

Pointgroup= C1 Stoichiometry= C18H12Br2O3 C1[X(C18H12Br2O3)] #Atoms= 35

Charge = 0 Multiplicity = 1

SCF Energy= -6062.08926671

==

Supporting Information: 020-Br-alpha-beta-unsaturated-HBTM_Br-Cinnamyl-acetate-complex-002.log

----

Using Gaussian 09: AM64L-G09RevD.01 24-Apr-2013

==

#M062X/6-31G(d) scf=(maxcycle=300,direct,tight) density=current

SCRF=(PCM,SOLVENT=THF) opt=(maxcycle=250,gdiis) iop(1/8=18) freq=noraman

#N Geom=AllCheck Guess=TCheck SCRF=Check Test GenChk RM062X/6-31G(d) Freq

----

Pointgroup= C1 Stoichiometry= C37H32Br2N2O3S C1[X(C37H32Br2N2O3S)] #Atoms= 77

Charge = 0 Multiplicity = 1

SCF Energy= -7305.40889463 Predicted Change= -4.704944D-08

==

Optimization completed. {Found 1 times}

Item	Max Val.	Criteria	Pass?	RMS Val.	Criteria	Pass?
Force	0.00001 ||	0.00045	[YES]	0.00000 ||	0.00030	[YES]
Displ	0.00587 ||	0.00180	[NO]	0.00587 ||	0.00180	[NO]

Atomic Type	Coordinates (Angstroms) X	Y	Z
C	8.406652	-0.964165	-0.070402
C	7.284350	-0.173260	0.137175
C	6.041233	-0.803034	0.110545
C	5.923553	-2.169975	-0.125562
C	7.051427	-2.960443	-0.330397
C	8.294336	-2.341264	-0.299751
N	4.791196	-0.198350	0.294174
C	3.752388	-1.031627	0.177077
S	4.244602	-2.679506	-0.106152
C	4.655482	1.217699	0.653753
C	3.316683	1.434052	1.338611
C	3.097149	2.915256	1.705563
C	1.915266	3.065860	2.666170

C	4.349405	3.531809	2.339080
C	2.190408	0.850313	0.462150
C	1.955468	1.579787	-0.843807
N	2.478666	-0.595807	0.227006
C	1.428285	-1.533055	-0.011751
C	0.101002	-0.999367	-0.363863
C	-0.812097	-1.899266	-0.770064
C	-2.208800	-1.750442	-1.193548
O	1.706777	-2.717985	-0.003672
C	0.969714	2.572019	-0.873233
C	0.727880	3.286135	-2.044010
C	1.459504	3.010120	-3.197839
C	2.432589	2.013993	-3.177603
C	2.678969	1.301339	-2.005814
C	-2.964139	-2.937161	-1.191802
C	-4.302910	-2.967674	-1.554703
C	-4.923458	-1.795660	-1.974008
C	-4.201955	-0.606468	-2.015578
C	-2.872653	-0.582097	-1.609828
Br	-2.002769	1.097111	-1.658840
H	9.387589	-0.501697	-0.056891
H	7.377628	0.894280	0.301213
H	6.959112	-4.026116	-0.510367
H	9.189208	-2.932995	-0.458963

H	4.755586	1.827963	-0.252479
H	5.481174	1.443503	1.329595
H	0.996118	2.599769	2.297789
H	1.722267	4.127276	2.852920
H	2.160771	2.597734	3.627892
H	2.880201	3.464776	0.780227
H	4.679550	2.940399	3.202037
H	4.120898	4.540013	2.696609
H	5.185247	3.616687	1.637679
H	3.312532	0.849740	2.270334
H	1.281160	0.868580	1.067508
H	-0.097854	0.056913	-0.264203
H	-0.480524	-2.936555	-0.763698
H	0.368735	2.739993	0.018547
H	-0.046232	4.048292	-2.059058
H	1.263487	3.560681	-4.112636
H	2.996811	1.783217	-4.075771
H	3.427431	0.510766	-2.013698
H	-2.472946	-3.854829	-0.879694
H	-4.855597	-3.900370	-1.514528
H	-5.967583	-1.798088	-2.270350
H	-4.678426	0.315642	-2.327948
O	0.354801	-0.384403	2.586807
C	-0.670833	0.228584	2.196304

C	-1.978511	-0.531710	2.287552
C	-3.051855	-0.103142	1.615835
C	-4.352524	-0.777097	1.498274
O	-0.709947	1.371069	1.665057
C	-4.533755	-2.110947	1.899866
C	-5.757305	-2.755525	1.784029
C	-6.851837	-2.081464	1.245111
C	-6.707747	-0.764764	0.822493
C	-5.474060	-0.136713	0.951852
Br	-5.339664	1.643581	0.294243
H	-1.962028	-1.456941	2.860022
H	-2.929233	0.834757	1.081829
H	-3.680084	-2.652115	2.296676
H	-5.855106	-3.787726	2.105226
H	-7.812795	-2.575853	1.144662
H	-7.541474	-0.224041	0.387818

Statistical Thermodynamic Analysis

Temperature= 298.150 Kelvin Pressure= 1.00000 Atm

===

SCF Energy= -7305.40889463 Predicted Change= -4.704944D-08

Zero-point correction (ZPE)= -7304.7950 0.61384

Internal Energy (U)= -7304.7548 0.65405

Enthalpy (H)= -7304.7539 0.65499

Gibbs Free Energy (G)= -7304.8706 0.53823

Frequencies -- 11.9344 18.9714 24.6060

==

#M062X/6-31+G(d,p) scf=(maxcycle=300,direct,tight) density=current

SCRF=(PCM,SOLVENT=THF)

Pointgroup= C1 Stoichiometry= C37H32Br2N2O3S C1[X(C37H32Br2N2O3S)] #Atoms= 77

Charge = 0 Multiplicity = 1

SCF Energy= -7305.55389316

==

Supporting Information: 020-Br-Cinnamyl-acetate-001.log

Using Gaussian 09: AM64L-G09RevD.01 24-Apr-2013

==

#M062X/6-31G(d) scf=(maxcycle=300,direct,tight) density=current

SCRF=(PCM,SOLVENT=THF) opt=(maxcycle=250,gdiis) iop(1/8=18) freq=noraman

#N Geom=AllCheck Guess=TCheck SCRF=Check Test GenChk RM062X/6-31G(d) Freq

----

Pointgroup= C1 Stoichiometry= C9H6BrO2(1-) C1[X(C9H6BrO2)] #Atoms= 18

Charge = -1 Multiplicity = 1

SCF Energy= -3068.71107688 Predicted Change= -2.972151D-09

===

Optimization completed. {Found 2 times}

Item	Max Val.		Criteria	Pass?	RMS Val.		Criteria	Pass?
Force	0.00001	||	0.00045	[YES]	0.00000	||	0.00030	[YES]
Displ	0.00045	||	0.00180	[YES]	0.00045	||	0.00180	[YES]

Atomic Type	Coordinates (Angstroms) X	Y	Z
O	3.556777	-1.157619	0.430389
C	3.595884	0.010987	-0.020877
C	2.258707	0.741677	-0.155525
C	1.098245	0.173558	0.185288
C	-0.225844	0.811913	0.108272
O	4.596326	0.674783	-0.381939
C	-1.414694	0.075757	0.007538
Br	-1.364073	-1.826879	-0.059534
C	-2.666637	0.677901	-0.061595
C	-2.762471	2.064362	-0.030118
C	-1.603152	2.830999	0.076788
C	-0.364193	2.209763	0.150875
H	2.304157	1.753277	-0.558132

H	1.143811	-0.854169	0.536959
H	-3.554795	0.060473	-0.140512
H	-3.737629	2.537666	-0.083021
H	-1.666003	3.913933	0.114584
H	0.533103	2.810777	0.262345

Statistical Thermodynamic Analysis

Temperature= 298.150 Kelvin Pressure= 1.00000 Atm

==

SCF Energy= -3068.71107688 Predicted Change= -2.972151D-09

Zero-point correction (ZPE)= -3068.5833 0.12770

Internal Energy (U)= -3068.5728 0.13822

Enthalpy (H)= -3068.5719 0.13916

Gibbs Free Energy (G)= -3068.6223 0.08871

Frequencies -- 27.0339 77.7702 82.3677

==

#M062X/6-31+G(d,p) scf=(maxcycle=300,direct,tight) density=current

SCRF=(PCM,SOLVENT=THF)

Pointgroup= C1 Stoichiometry= C9H6BrO2(1-) C1[X(C9H6BrO2)] #Atoms= 18

Charge = -1 Multiplicity = 1

SCF Energy= -3068.78006420

==

Supporting Information: 020-Br-Cinnamyl-acid-001.log

Using Gaussian 09: AM64L-G09RevD.01 24-Apr-2013

==

#M062X/6-31G(d) scf=(maxcycle=300,direct,tight) density=current

SCRF=(PCM,SOLVENT=THF) opt=(maxcycle=250,gdiis) iop(1/8=18) freq=noraman

#N Geom=AllCheck Guess=TCheck SCRF=Check Test GenChk RM062X/6-31G(d) Freq

----

Pointgroup= C1 Stoichiometry= C9H7BrO2 C1[X(C9H7BrO2)] #Atoms= 19

Charge = 0 Multiplicity = 1

SCF Energy= -3069.20020909 Predicted Change= -1.153453D-08

==

Optimization completed. {Found 2 times}

Item	Max Val.	Criteria	Pass?	RMS Val.	Criteria	Pass?
Force	0.00003 ||	0.00045	[YES]	0.00000 ||	0.00030	[YES]
Displ	0.00110 ||	0.00180	[YES]	0.00110 ||	0.00180	[YES]

Atomic Type	Coordinates (Angstroms) X	Y	Z
O	-4.532739	0.682513	-0.365262

C	-3.460976	-0.034972	0.012792
C	-2.208852	0.736798	-0.142405
C	-1.042435	0.162966	0.175284
C	0.271713	0.812476	0.098998
O	-3.545485	-1.171128	0.424558
C	1.460274	0.073558	0.006743
Br	1.417188	-1.823703	-0.059645
C	2.705724	0.687477	-0.056599
C	2.787548	2.075315	-0.023762
C	1.625682	2.838667	0.075484
C	0.390116	2.210763	0.138861
H	-5.318027	0.120631	-0.237856
H	-2.295252	1.746259	-0.530499
H	-1.069105	-0.870721	0.512188
H	3.600549	0.079847	-0.131136
H	3.759744	2.554997	-0.070408
H	1.683728	3.921221	0.113704
H	-0.510174	2.808005	0.244848

Statistical Thermodynamic Analysis

Temperature= 298.150 Kelvin Pressure= 1.00000 Atm

===

SCF Energy= -3069.20020909 Predicted Change= -1.153453D-08

Zero-point correction (ZPE)= -3069.0590 0.14113

Internal Energy (U)= -3069.0484 0.15178

Enthalpy (H)= -3069.0474 0.15272

Gibbs Free Energy (G)= -3069.0977 0.10249

Frequencies -- 42.9080 77.5294 80.1365

==

#M062X/6-31+G(d,p) scf=(maxcycle=300,direct,tight) density=current

SCRF=(PCM,SOLVENT=THF)

Pointgroup= C1 Stoichiometry= C9H7BrO2 C1[X(C9H7BrO2)] #Atoms= 19

Charge = 0 Multiplicity = 1

SCF Energy= -3069.25526569

==

Supporting Information: Re-Re-Nuc-attack_X-equals-S-PhBr_substrate-006.log

----

Using Gaussian 09: AM64L-G09RevD.01 24-Apr-2013

==

#M062X/6-31G(d) scf=(maxcycle=300,direct,tight) density=current

SCRF=(PCM,SOLVENT=THF) opt=(maxcycle=250,ts,calcfc,noeigentest)

freq=noraman

#N Geom=AllCheck Guess=TCheck SCRF=Check GenChk RM062X/6-31G(d) Freq

----

Pointgroup= C1 Stoichiometry= C43H36BrN3O2S2 C1[X(C43H36BrN3O2S2)] #Atoms= 87

Charge = 0 Multiplicity = 1

SCF Energy= -5342.27066443 Predicted Change= -5.916728D-09

==

Optimization completed. {Found 1 times}

Item	Max Val.	Criteria	Pass?	RMS Val.	Criteria	Pass?
Force	0.00003 ||	0.00045	[YES]	0.00000 ||	0.00030	[YES]
Displ	0.00230 ||	0.00180	[NO]	0.00230 ||	0.00180	[YES]

Atomic Type	Coordinates (Angstroms) X	Y	Z
C	6.555931	1.131697	-0.523944
C	5.427946	0.382126	-0.835920
C	4.372306	0.402651	0.070956
C	4.440287	1.134557	1.254708
C	5.568696	1.888604	1.560865
C	6.626012	1.879053	0.658013
N	3.133454	-0.231108	-0.077420
C	2.282416	-0.015261	0.934944
S	2.970024	0.964030	2.199757
C	2.806737	-0.988727	-1.291014

C	1.396824	-1.590401	-1.250279
C	1.323595	-2.851346	-2.131863
C	-0.012903	-3.582932	-1.982820
C	1.554707	-2.471432	-3.597159
C	0.917315	-1.827821	0.185829
C	1.521620	-2.971194	0.985966
N	1.062026	-0.550161	0.941796
C	0.121800	-0.199999	2.030830
C	-1.255630	-0.293076	1.718114
C	-1.778202	-0.280153	0.422455
C	-3.104149	-0.851016	0.088873
O	0.628700	0.098240	3.107879
C	0.640473	-3.752418	1.742028
C	1.106709	-4.797415	2.533674
C	2.469981	-5.076332	2.582556
C	3.358049	-4.303124	1.839669
C	2.890196	-3.256760	1.047835
C	-3.489003	-1.097870	-1.237206
Br	-2.324495	-0.656044	-2.676909
C	-4.726662	-1.638178	-1.566901
C	-5.629037	-1.944777	-0.554666
C	-5.279105	-1.715189	0.774451
C	-4.033111	-1.188781	1.084741
H	7.393338	1.139946	-1.213308

H	5.368174	-0.179836	-1.761614
H	5.618121	2.467366	2.477099
H	7.517127	2.457819	0.875784
H	3.565671	-1.772507	-1.398969
H	2.911127	-0.295683	-2.130800
H	-0.849864	-2.902190	-2.169915
H	-0.134389	-4.019008	-0.986551
H	-0.074594	-4.398899	-2.709294
H	2.125861	-3.530781	-1.809727
H	1.520522	-3.359939	-4.234087
H	2.524341	-1.987828	-3.755198
H	0.770432	-1.781840	-3.933842
H	0.701980	-0.843585	-1.664497
H	-0.150591	-2.021065	0.140046
H	-1.901730	-0.212537	2.585129
H	-1.069381	-0.346390	-0.396794
H	-0.424319	-3.530744	1.710545
H	0.405562	-5.391575	3.111061
H	2.838853	-5.891442	3.197109
H	4.422477	-4.512962	1.872433
H	3.612624	-2.673938	0.486965
H	-4.973668	-1.813576	-2.608051
H	-6.599347	-2.360031	-0.807583
H	-5.977617	-1.946277	1.572138

H	-3.772720	-1.021732	2.124432
C	-2.111780	1.763701	-0.264815
C	-0.750363	2.131037	-0.536025
C	-2.795844	2.222014	0.911976
O	-2.185239	2.658588	1.897828
C	-4.278575	1.998131	1.005210
S	0.252872	2.957594	0.675256
C	1.572150	2.937973	-0.464558
C	1.167360	2.274740	-1.642439
N	-0.137091	1.829277	-1.654117
C	2.857390	3.468880	-0.335666
C	3.745204	3.322501	-1.393730
C	3.360198	2.647685	-2.563121
C	2.080205	2.129061	-2.699284
C	-4.839922	1.919477	2.283550
C	-6.191171	1.634294	2.446883
C	-6.999799	1.433900	1.329239
C	-6.452253	1.528419	0.050780
C	-5.099452	1.809392	-0.111466
H	-2.677678	1.520631	-1.153550
H	3.157188	3.985037	0.572117
H	4.753269	3.715774	-1.304163
H	4.073740	2.536901	-3.374303
H	1.765141	1.620545	-3.606532

H	-4.189676	2.071343	3.139575
H	-6.614309	1.563377	3.444534
H	-8.054760	1.208014	1.453849
H	-7.079563	1.378191	-0.822848
H	-4.689627	1.880143	-1.114674

Statistical Thermodynamic Analysis

Temperature= 298.150 Kelvin Pressure= 1.00000 Atm

===

SCF Energy= -5342.27066443 Predicted Change= -5.916728D-09

Zero-point correction (ZPE)= -5341.5742 0.69646

Internal Energy (U)= -5341.5314 0.73926

Enthalpy (H)= -5341.5304 0.74020

Gibbs Free Energy (G)= -5341.6512 0.61943

Frequencies -- -285.3069 12.9168 21.4666

===

#M062X/6-31+G(d,p) scf=(maxcycle=300,direct,tight) density=current

SCRF=(PCM,SOLVENT=THF)

Pointgroup= C1 Stoichiometry= C43H36BrN3O2S2 C1[X(C43H36BrN3O2S2)]
#Atoms= 87

Charge = 0 Multiplicity = 1

SCF Energy= -5342.39602497

==

Supporting Information: Re-Si-Nuc-attack_X-equals-S-PhBr_substrate-001.log

----

Using Gaussian 09: AM64L-G09RevD.01 24-Apr-2013

==

#M062X/6-31G(d) scf=(maxcycle=300,direct,tight) density=current

SCRF=(PCM,SOLVENT=THF) opt=(maxcycle=250,ts,calcfc,noeigentest)

freq=noraman

#N Geom=AllCheck Guess=TCheck SCRF=Check GenChk RM062X/6-31G(d) Freq

----

Pointgroup= C1 Stoichiometry= C43H36BrN3O2S2 C1[X(C43H36BrN3O2S2)] #Atoms= 87

Charge = 0 Multiplicity = 1

SCF Energy= -5342.27556081 Predicted Change= -1.694350D-09

==

Optimization completed. {Found 2 times}

Item	Max Val.	Criteria	Pass?	RMS Val.	Criteria	Pass?
Force	0.00000 ||	0.00045	[YES]	0.00000 ||	0.00030	[YES]
Displ	0.00156 ||	0.00180	[YES]	0.00156 ||	0.00180	[YES]

Atomic Coordinates (Angstroms)

Type	X	Y	Z
C	7.403033	-0.523221	0.156186
C	6.284847	0.233173	0.491177
C	5.083847	-0.076161	-0.142895
C	5.001897	-1.094040	-1.090313
C	6.121463	-1.853331	-1.416188
C	7.324179	-1.555739	-0.784503
N	3.843548	0.540052	0.065255
C	2.842523	0.027332	-0.674060
S	3.364940	-1.290786	-1.691705
C	3.651691	1.555524	1.104013
C	2.181915	1.648362	1.483715
C	1.906177	2.825197	2.437254
C	0.460397	2.789479	2.941400
C	2.865506	2.803700	3.631496
C	1.297250	1.681121	0.227852
C	1.345372	2.968884	-0.574619
N	1.605297	0.518571	-0.634452
C	0.517343	-0.213185	-1.291752
C	-0.768794	0.354706	-1.181255
C	-1.893765	-0.473467	-1.277132
C	-3.240549	0.126003	-1.473653
O	0.836342	-1.261020	-1.857387

C	0.256967	3.844416	-0.545515
C	0.293236	5.046551	-1.248904
C	1.416846	5.384024	-1.997851
C	2.496413	4.504869	-2.055389
C	2.459396	3.304445	-1.352530
C	-3.804185	1.146072	-0.696514
Br	-2.900847	1.882386	0.818561
C	-5.085667	1.631718	-0.929780
C	-5.839661	1.113433	-1.977294
C	-5.309145	0.102405	-2.772395
C	-4.033811	-0.383463	-2.511318
H	8.351263	-0.304719	0.635407
H	6.354251	1.036606	1.216333
H	6.053769	-2.655170	-2.144151
H	8.210882	-2.131663	-1.026305
H	4.041904	2.516320	0.746505
H	4.246313	1.238578	1.963579
H	0.251942	1.833617	3.436968
H	-0.272710	2.916644	2.139934
H	0.293994	3.590868	3.666893
H	2.065643	3.759261	1.880999
H	3.905000	2.975404	3.336590
H	2.809969	1.842151	4.156408
H	2.594755	3.588999	4.342989

H	1.904872	0.722246	2.005524
H	0.280681	1.505822	0.580051
H	-0.894466	1.385893	-0.898903
H	-1.764950	-1.419949	-1.803243
H	-0.646024	3.568801	-0.005230
H	-0.564344	5.711183	-1.220103
H	1.445555	6.318601	-2.549004
H	3.367346	4.749697	-2.655069
H	3.300329	2.620243	-1.436566
H	-5.488072	2.405934	-0.285826
H	-6.839917	1.494244	-2.156442
H	-5.890083	-0.318089	-3.587026
H	-3.625562	-1.185714	-3.119504
C	2.227944	-4.395735	0.098167
C	1.151559	-3.543767	0.344775
C	1.306481	-2.367589	1.107695
C	2.561370	-2.072861	1.663969
C	3.634206	-2.915624	1.411313
C	3.471839	-4.064383	0.622665
S	-0.510413	-3.700944	-0.157570
C	-0.852671	-2.153426	0.642322
C	-2.129709	-1.505774	0.538624
C	-3.326350	-2.260588	0.227760
C	-4.650291	-1.712718	0.680561

O	-3.294299	-3.274149	-0.480102
N	0.168867	-1.594633	1.245521
C	-5.786992	-2.046936	-0.061766
C	-7.034057	-1.546554	0.292760
C	-7.159944	-0.715439	1.405604
C	-6.035468	-0.393848	2.163148
C	-4.785175	-0.888802	1.802573
H	2.099109	-5.295785	-0.495390
H	2.676580	-1.199501	2.299837
H	4.610862	-2.684600	1.827831
H	4.324133	-4.708302	0.428261
H	-2.212730	-0.655326	1.202828
H	-5.666764	-2.692104	-0.926649
H	-7.908521	-1.800526	-0.298933
H	-8.133523	-0.323419	1.684935
H	-6.131034	0.241664	3.038469
H	-3.918340	-0.638912	2.405985

Statistical Thermodynamic Analysis

Temperature= 298.150 Kelvin Pressure= 1.00000 Atm

===

SCF Energy= -5342.27556081 Predicted Change= -1.694350D-09

Zero-point correction (ZPE)= -5341.5788 0.69675

Internal Energy (U)= -5341.5362 0.73935

Enthalpy (H)= -5341.5352 0.74030

Gibbs Free Energy (G)= -5341.6545 0.62105

Frequencies -- -374.8074 15.2373 20.0053

==

#M062X/6-31+G(d,p) scf=(maxcycle=300,direct,tight) density=current

SCRF=(PCM,SOLVENT=THF)

Pointgroup= C1 Stoichiometry= C43H36BrN3O2S2 C1[X(C43H36BrN3O2S2)] #Atoms= 87

Charge = 0 Multiplicity = 1

SCF Energy= -5342.40032743

==

Supporting Information: Si-Re-Nuc-attack_X-equals-S-PhBr_substrate-004.log

----

Using Gaussian 09: AM64L-G09RevD.01 24-Apr-2013

==

#M062X/6-31G(d) scf=(maxcycle=300,direct,tight) density=current

SCRF=(PCM,SOLVENT=THF) opt=(maxcycle=250,ts,calcfc,noeigentest)

freq=noraman

#N Geom=AllCheck Guess=TCheck SCRF=Check GenChk RM062X/6-31G(d) Freq

----

Pointgroup= C1 Stoichiometry= C43H36BrN3O2S2 C1[X(C43H36BrN3O2S2)] #Atoms= 87

Charge = 0 Multiplicity = 1

SCF Energy= -5342.26748352 Predicted Change= 2.310521D-10

==

Optimization completed. {Found 2 times}

Item	Max Val.	Criteria	Pass?	RMS Val.	Criteria	Pass?
Force	0.00000 ||	0.00045	[YES]	0.00000 ||	0.00030	[YES]
Displ	0.00018 ||	0.00180	[YES]	0.00018 ||	0.00180	[YES]

Atomic Type	Coordinates (Angstroms) X	Y	Z
C	6.208349	0.018198	2.200783
C	4.918457	0.525870	2.102245
C	4.214835	0.272487	0.926636
C	4.780801	-0.453072	-0.119551
C	6.070963	-0.963749	-0.014245
C	6.779217	-0.718967	1.156225
N	2.899918	0.659163	0.640014
C	2.463297	0.264922	-0.563406
S	3.668630	-0.615544	-1.466206
C	2.124923	1.495411	1.565264

C	0.662885	1.595918	1.143948
C	-0.104696	2.700075	1.918299
C	-0.798878	2.087682	3.137624
C	0.731972	3.917482	2.323630
C	0.567886	1.741060	-0.386011
C	1.054980	3.069374	-0.935021
N	1.240116	0.570667	-0.999873
C	0.720118	-0.142228	-2.183869
C	-0.667626	-0.414111	-2.236903
C	-1.587352	-0.379521	-1.182454
C	-3.033845	-0.293676	-1.515415
O	1.558105	-0.431842	-3.035459
C	2.399934	3.389182	-1.138014
C	2.762626	4.665603	-1.562027
C	1.788661	5.636153	-1.785472
C	0.444854	5.320752	-1.599214
C	0.081974	4.043284	-1.183314
C	-3.890998	0.610868	-0.883283
Br	-3.206047	1.807293	0.429664
C	-5.249580	0.678325	-1.170776
C	-5.783898	-0.176203	-2.128608
C	-4.955396	-1.084168	-2.785659
C	-3.602313	-1.138098	-2.478334
H	6.778485	0.197906	3.105808

H	4.478694	1.093149	2.914911
H	6.504928	-1.542375	-0.823137
H	7.787086	-1.105872	1.260483
H	2.612081	2.476455	1.579055
H	2.207275	1.050325	2.561092
H	-0.062972	1.686304	3.845118
H	-1.456591	1.266145	2.834976
H	-1.390012	2.843684	3.664926
H	-0.886829	3.065085	1.241736
H	1.464109	3.661132	3.098349
H	0.072703	4.683417	2.743042
H	1.263879	4.361203	1.475297
H	0.180650	0.634370	1.369505
H	-0.479788	1.674547	-0.661047
H	-0.983260	-0.786378	-3.205838
H	-1.315023	0.135072	-0.270262
H	3.181516	2.650845	-0.981585
H	3.810847	4.899265	-1.719512
H	2.076232	6.630172	-2.112941
H	-0.322446	6.065866	-1.783957
H	-0.968327	3.790509	-1.047416
H	-5.876364	1.391347	-0.646434
H	-6.844499	-0.133159	-2.354095
H	-5.365665	-1.758072	-3.530751

H	-2.962363	-1.864328	-2.972989
C	1.655022	-3.421750	-0.726466
C	2.116479	-2.986051	0.534260
C	3.434170	-3.199064	0.937056
C	4.294896	-3.847998	0.058295
C	3.849454	-4.274605	-1.201444
C	2.535295	-4.072166	-1.598505
N	0.326025	-3.157575	-0.996584
C	-0.252616	-2.547828	0.000536
C	-1.637183	-2.166923	-0.034007
C	-2.279237	-1.654896	1.157295
C	-3.773142	-1.717918	1.283865
O	-1.624143	-1.078199	2.040309
S	0.820055	-2.180719	1.382776
C	-4.570999	-2.563988	0.506948
C	-5.956143	-2.545586	0.643382
C	-6.557437	-1.679566	1.553538
C	-5.767860	-0.841392	2.341461
C	-4.384943	-0.865610	2.210308
H	3.786915	-2.855153	1.905581
H	5.326759	-4.017355	0.351285
H	4.541451	-4.775940	-1.871342
H	2.176762	-4.400528	-2.568746
H	-2.214475	-2.756744	-0.735721

H	-4.120358	-3.247979	-0.204571
H	-6.565976	-3.204853	0.033237
H	-7.638648	-1.660609	1.653573
H	-6.232660	-0.168653	3.055996
H	-3.752976	-0.220356	2.811672

Statistical Thermodynamic Analysis

Temperature= 298.150 Kelvin Pressure= 1.00000 Atm

==

SCF Energy= -5342.26748352 Predicted Change= 2.310521D-10

Zero-point correction (ZPE)= -5341.5697 0.69772

Internal Energy (U)= -5341.5272 0.74025

Enthalpy (H)= -5341.5262 0.74119

Gibbs Free Energy (G)= -5341.6442 0.62320

Frequencies -- -342.7320 21.7194 29.6918

==

#M062X/6-31+G(d,p) scf=(maxcycle=300,direct,tight) density=current

SCRF=(PCM,SOLVENT=THF)

Pointgroup= C1 Stoichiometry= C43H36BrN3O2S2 C1[X(C43H36BrN3O2S2)] #Atoms= 87

Charge = 0 Multiplicity = 1

SCF Energy= -5342.39480393

==

Supporting Information: Si-Si-Nuc-attack_X-equals-S-PhBr_substrate-006.log

----

Using Gaussian 09: AM64L-G09RevD.01 24-Apr-2013

==

#M062X/6-31G(d) scf=(maxcycle=300,direct,tight) density=current

SCRF=(PCM,SOLVENT=THF) opt=(maxcycle=250,ts,calcfc,noeigentest,gdiis)

iop(1/8=18) freq=noraman

#N Geom=AllCheck Guess=TCheck SCRF=Check Test GenChk RM062X/6-31G(d) Freq

----

Pointgroup= C1 Stoichiometry= C43H36BrN3O2S2 C1[X(C43H36BrN3O2S2)] #Atoms= 87

Charge = 0 Multiplicity = 1

SCF Energy= -5342.27159546 Predicted Change= -3.504154D-09

==

Optimization completed. {Found 1 times}

Item	Max Val.	Criteria	Pass?	RMS Val.	Criteria	Pass?
Force	0.00000 ||	0.00045	[YES]	0.00000 ||	0.00030	[YES]
Displ	0.00189 ||	0.00180	[NO]	0.00189 ||	0.00180	[YES]

Atomic Coordinates (Angstroms)

Type	X	Y	Z
C	-7.633566	-0.134846	-1.182696
C	-6.539997	-0.763532	-0.597309
C	-5.280857	-0.217979	-0.832984
C	-5.113746	0.910689	-1.632499
C	-6.211145	1.539733	-2.211378
C	-7.473593	1.003636	-1.979116
N	-4.057987	-0.694325	-0.342394
C	-2.985820	-0.000137	-0.763400
S	-3.421683	1.387350	-1.734075
C	-3.970770	-1.790713	0.627910
C	-2.656500	-1.680630	1.386997
C	-2.448554	-2.833929	2.384666
C	-1.264757	-2.536819	3.310718
C	-3.702875	-3.085423	3.227210
C	-1.494208	-1.545814	0.384210
C	-1.209719	-2.797514	-0.423700
N	-1.742089	-0.377538	-0.491776
C	-0.611017	0.448440	-0.949665
C	0.640455	-0.192673	-0.937589
C	1.809810	0.577241	-1.001370
C	3.086902	0.005403	-1.496122
O	-0.895230	1.591248	-1.316912

C	-1.954488	-3.144754	-1.553057
C	-1.667365	-4.314349	-2.253750
C	-0.628770	-5.143452	-1.837316
C	0.130438	-4.794457	-0.721831
C	-0.157084	-3.625776	-0.021965
C	4.197072	0.804715	-1.802200
Br	4.133430	2.684666	-1.533261
C	5.394202	0.274275	-2.270415
C	5.513704	-1.099997	-2.441332
C	4.432183	-1.926175	-2.144475
C	3.241686	-1.376515	-1.687998
H	-8.625797	-0.541343	-1.019461
H	-6.666835	-1.652771	0.010492
H	-6.082803	2.422645	-2.828884
H	-8.342985	1.474755	-2.424661
H	-4.064051	-2.750926	0.105962
H	-4.819256	-1.675092	1.304763
H	-1.494813	-1.669441	3.941738
H	-0.336593	-2.312941	2.776935
H	-1.080738	-3.390093	3.970656
H	-2.234706	-3.745418	1.811110
H	-3.489389	-3.831018	3.998624
H	-4.540790	-3.461556	2.632417
H	-4.023252	-2.165811	3.732198

H	-2.682986	-0.739445	1.957920
H	-0.596328	-1.282182	0.948232
H	0.693214	-1.252911	-0.746971
H	1.666779	1.631620	-1.229010
H	-2.749179	-2.492811	-1.909848
H	-2.251052	-4.571851	-3.131850
H	-0.402941	-6.051144	-2.387850
H	0.953921	-5.425511	-0.402385
H	0.453914	-3.330863	0.829477
H	6.222734	0.937823	-2.492516
H	6.447765	-1.517492	-2.803087
H	4.513884	-3.000868	-2.271552
H	2.405540	-2.035041	-1.474060
C	2.346921	1.076844	0.995856
C	1.231858	1.934406	1.322745
C	2.436517	-0.257426	1.525407
O	1.419493	-0.881237	1.878260
C	3.762978	-0.962599	1.541277
C	4.971716	-0.344205	1.200836
C	6.158301	-1.071386	1.200694
C	6.156988	-2.419493	1.552023
C	4.959500	-3.040559	1.905142
C	3.773802	-2.315707	1.898979
N	1.203626	3.203193	1.020831

C	-0.011955	3.768418	1.344314
C	-0.951591	2.894644	1.929031
S	-0.251873	1.307900	2.078125
C	-2.225881	3.332476	2.293415
C	-2.556271	4.661744	2.066434
C	-1.627336	5.543113	1.488366
C	-0.361812	5.109480	1.128116
H	3.254667	1.638783	0.819331
H	5.002115	0.699917	0.905539
H	7.086347	-0.581592	0.921598
H	7.084780	-2.983628	1.551839
H	4.951946	-4.090317	2.183052
H	2.832508	-2.783177	2.169774
H	-2.942325	2.649255	2.740134
H	-3.542780	5.022946	2.339584
H	-1.907228	6.578611	1.320489
H	0.362468	5.781793	0.679103

Statistical Thermodynamic Analysis

Temperature= 298.150 Kelvin   Pressure= 1.00000 Atm

==

SCF Energy=   -5342.27159546   Predicted Change= -3.504154D-09

Zero-point correction (ZPE)=   -5341.5741 0.69741

Internal Energy (U)=   -5341.5314 0.74013

Enthalpy (H)= -5341.5305 0.74107

Gibbs Free Energy (G)= -5341.6510 0.62050

Frequencies -- -349.0185 15.6683 23.0845

==

#M062X/6-31+G(d,p) scf=(maxcycle=300,direct,tight) density=current

SCRF=(PCM,SOLVENT=THF)

Pointgroup= C1 Stoichiometry= C43H36BrN3O2S2 C1[X(C43H36BrN3O2S2)] #Atoms= 87

Charge = 0 Multiplicity = 1

SCF Energy= -5342.39814120

==

Supporting Information: 040_X-equals-S-014.log

Using Gaussian 09: AM64L-G09RevD.01 24-Apr-2013

==

#M062X/6-31G(d) scf=(maxcycle=300,direct,tight) density=current

SCRF=(PCM,SOLVENT=THF) opt=(maxcycle=250,gdiis) iop(1/8=18) freq=noraman

#N Geom=AllCheck Guess=TCheck SCRF=Check Test GenChk RM062X/6-31G(d) Freq

----

Pointgroup= C1 Stoichiometry= C43H36BrN3O2S2 C1[X(C43H36BrN3O2S2)] #Atoms= 87

Charge = 0 Multiplicity = 1

SCF Energy= -5342.29549737 Predicted Change= -1.999697D-09

==

Optimization completed on the basis of negligible forces. {Found 2 times}

Item	Max Val.	Criteria	Pass?	RMS Val.	Criteria	Pass?
Force	0.00000 ||	0.00045	[YES]	0.00000 ||	0.00030	[YES]
Displ	0.00260 ||	0.00180	[NO]	0.00260 ||	0.00180	[YES]

Atomic Type	Coordinates (Angstroms) X	Y	Z
N	-4.215660	1.107984	-0.219944
C	-3.063291	1.115714	-0.903646
N	-2.133696	0.164680	-0.742075
C	-0.857320	0.304039	-1.390698
C	0.034574	-0.901647	-1.430264
O	-0.633604	1.312347	-2.021214
C	-2.355888	-0.919164	0.251415
C	-2.927467	-2.159816	-0.406518
C	-3.155693	-0.340540	1.428910
C	-3.354815	-1.350617	2.571650
C	-1.998939	-1.849667	3.086503
C	-4.153970	-0.723642	3.718439

C	-4.471104	0.238303	0.936596
C	-5.125744	2.095386	-0.619135
C	-4.603639	2.908881	-1.621431
S	-2.964921	2.433652	-2.041787
C	-6.410063	2.288760	-0.116441
C	-7.154052	3.335327	-0.646002
C	-6.631060	4.164020	-1.645859
C	-5.349250	3.961706	-2.144158
C	-4.057711	-2.114309	-1.230413
C	-4.572497	-3.279358	-1.791952
C	-3.959613	-4.505843	-1.544329
C	-2.816329	-4.557333	-0.751862
C	-2.299747	-3.391080	-0.192423
C	1.540410	-0.579629	-1.307932
C	2.000899	0.361171	-0.200106
C	3.312921	0.882737	-0.435657
C	1.167101	0.741944	0.865608
C	1.742597	1.317603	2.131966
O	-0.084584	0.580345	0.886144
S	4.089607	2.183589	0.552890
C	5.435666	2.197644	-0.558324
C	5.213821	1.238536	-1.568014
N	4.034470	0.535901	-1.478773
C	6.590017	2.974776	-0.527682

C	7.538003	2.795675	-1.532312
C	7.331939	1.847227	-2.542340
C	6.181485	1.068919	-2.567343
C	1.275846	2.538331	2.624287
C	1.758351	3.038263	3.830109
C	2.680237	2.299447	4.572023
C	3.116067	1.060347	4.105862
C	2.653337	0.573419	2.886253
C	2.269847	-1.924113	-1.258800
C	2.191639	-2.830088	-0.197559
Br	1.160245	-2.440577	1.363871
C	2.842592	-4.060714	-0.211466
C	3.609539	-4.416691	-1.314843
C	3.713232	-3.537860	-2.390268
C	3.052158	-2.316222	-2.351641
H	-0.259347	-1.653141	-0.708556
H	-0.124150	-1.349090	-2.421349
H	-1.367832	-1.125834	0.657366
H	-2.537990	0.484519	1.811398
H	-1.538424	-2.564052	2.399855
H	-2.120568	-2.354713	4.049035
H	-1.300030	-1.014235	3.219126
H	-3.922542	-2.206830	2.180497
H	-5.176072	-0.464482	3.426014

H	-3.659408	0.184654	4.083187
H	-4.223396	-1.426240	4.553491
H	-4.928443	0.871213	1.699497
H	-5.184876	-0.543661	0.650754
H	-6.819851	1.641880	0.651120
H	-8.158757	3.508775	-0.276359
H	-7.233728	4.974938	-2.040102
H	-4.941642	4.601955	-2.918994
H	-4.533956	-1.165046	-1.463558
H	-5.449818	-3.227192	-2.428864
H	-4.361625	-5.414196	-1.981734
H	-2.315004	-5.504074	-0.577621
H	-1.375659	-3.432938	0.380843
H	1.839927	-0.120347	-2.255474
H	6.746612	3.707730	0.257966
H	8.442095	3.396302	-1.529473
H	8.082794	1.717504	-3.316053
H	6.015472	0.329195	-3.344498
H	0.544448	3.096540	2.046320
H	1.411350	3.999743	4.196992
H	3.051638	2.686303	5.516354
H	3.821345	0.476547	4.689925
H	2.986427	-0.388921	2.506566
H	2.745714	-4.727599	0.638395

H	4.122439	-5.373100	-1.327746
H	4.312867	-3.801219	-3.255833
H	3.151635	-1.617124	-3.176726

Statistical Thermodynamic Analysis

Temperature= 298.150 Kelvin Pressure= 1.00000 Atm

===

SCF Energy= -5342.29549737 Predicted Change= -1.999697D-09

Zero-point correction (ZPE)= -5341.5951 0.70039

Internal Energy (U)= -5341.5521 0.74335

Enthalpy (H)= -5341.5511 0.74430

Gibbs Free Energy (G)= -5341.6737 0.62171

Frequencies -- 8.2426 15.9101 20.9831

===

#M062X/6-31+G(d,p) scf=(maxcycle=300,direct,tight) density=current

SCRF=(PCM,SOLVENT=THF)

Pointgroup= C1 Stoichiometry= C43H36BrN3O2S2 C1[X(C43H36BrN3O2S2)] #Atoms= 87

Charge = 0 Multiplicity = 1

SCF Energy= -5342.41915559

===

Supporting Information: 045-Re-Br_Lactamization-X-equals-S-002.log

----

Using Gaussian 09: AM64L-G09RevD.01 24-Apr-2013

===

#M062X/6-31G(d) scf=(maxcycle=300,direct,tight) density=current

SCRF=(PCM,SOLVENT=THF) opt=(maxcycle=250,ts,calcfc,noeigentest,gdiis)

iop(1/8=18) freq=noraman

#N Geom=AllCheck Guess=TCheck SCRF=Check Test GenChk RM062X/6-31G(d) Freq

----

Pointgroup= C1 Stoichiometry= C43H36BrN3O2S2 C1[X(C43H36BrN3O2S2)] #Atoms= 87

Charge = 0 Multiplicity = 1

SCF Energy= -5342.28816262 Predicted Change= -5.639281D-09

===

Optimization completed. {Found 2 times}

Item	Max Val.	Criteria	Pass?	RMS Val.	Criteria	Pass?
Force	0.00001 ||	0.00045	[YES]	0.00000 ||	0.00030	[YES]
Displ	0.00154 ||	0.00180	[YES]	0.00154 ||	0.00180	[YES]

Atomic Coordinates (Angstroms)

Type X Y Z

N	3.561438	-1.069755	1.133491
C	2.594919	-1.305388	0.233328
N	1.449975	-0.622491	0.238156
C	0.267572	-1.205088	-0.431523
C	-0.990976	-1.188754	0.424341
O	0.480066	-2.038630	-1.288802
C	1.352781	0.558235	1.121622
C	2.013609	1.804364	0.553517
C	1.915379	0.139489	2.499703
C	1.695967	1.176297	3.614189
C	0.201748	1.343989	3.907246
C	2.425435	0.774567	4.900078
C	3.387674	-0.205868	2.312100
C	4.729061	-1.816760	0.928388
C	4.607344	-2.685954	-0.153869
S	3.030597	-2.538270	-0.921428
C	5.895918	-1.754699	1.685342
C	6.940047	-2.598540	1.325687
C	6.820943	-3.479321	0.245036
C	5.651546	-3.535502	-0.505675
C	3.165839	1.788746	-0.239187
C	3.752802	2.979011	-0.657015
C	3.200835	4.203444	-0.287349
C	2.045991	4.229941	0.487257

C	1.455051	3.037326	0.896834
C	-2.274522	-0.485617	-0.089676
C	-2.863377	-1.247874	-1.277043
C	-2.159035	1.010143	-0.270248
C	-1.081065	1.478506	-1.042767
C	-3.081974	1.950571	0.274295
C	-4.289047	1.485696	1.056385
O	-2.931542	3.185622	0.159899
C	-2.737958	-0.803217	-2.598665
C	-3.285653	-1.507585	-3.665392
C	-3.995237	-2.684122	-3.440222
C	-4.161813	-3.142849	-2.139091
C	-3.600857	-2.422483	-1.089267
S	-0.854104	3.165852	-1.561366
C	0.562208	2.666718	-2.471423
C	0.764849	1.280263	-2.321038
N	-0.153241	0.654262	-1.511110
C	1.431161	3.438541	-3.235566
C	2.500475	2.810435	-3.870775
C	2.694814	1.429479	-3.741410
C	1.833783	0.654364	-2.974149
C	-5.128911	0.449096	0.638317
C	-6.245856	0.094286	1.392272
C	-6.539190	0.776858	2.570211

C	-5.720424	1.828172	2.982848
C	-4.610743	2.185246	2.224034
Br	-3.922404	-3.084165	0.672177
H	-2.195984	0.115185	-2.788087
H	-1.220279	-2.253142	0.548131
H	-0.776777	-0.804897	1.424392
H	0.289029	0.776077	1.209052
H	1.384976	-0.779977	2.790379
H	-0.221290	0.399702	4.270622
H	-0.379792	1.654991	3.033275
H	0.052071	2.098752	4.684412
H	2.104372	2.136800	3.274905
H	3.513367	0.786900	4.784185
H	2.124016	-0.229919	5.221158
H	2.174771	1.471723	5.704626
H	3.770418	-0.766791	3.167320
H	3.995288	0.696717	2.174177
H	5.991256	-1.069586	2.520520
H	7.862212	-2.570227	1.895722
H	7.651768	-4.126225	-0.014401
H	5.556467	-4.217206	-1.344047
H	3.599462	0.852675	-0.577444
H	4.634571	2.945889	-1.289038
H	3.658908	5.130588	-0.618107

H	1.588207	5.175776	0.759820
H	0.533472	3.068419	1.473872
H	-2.968760	-0.644715	0.738088
H	-3.160784	-1.130449	-4.675401
H	-4.429640	-3.237789	-4.266436
H	-4.728062	-4.043793	-1.930293
H	1.279067	4.509387	-3.336814
H	3.184273	3.398660	-4.474935
H	3.527685	0.955546	-4.252843
H	1.965275	-0.419759	-2.874764
H	-4.922921	-0.075187	-0.291366
H	-6.884508	-0.716908	1.055252
H	-7.406233	0.496982	3.160970
H	-5.951104	2.371239	3.894577
H	-3.976604	3.014008	2.524436

Statistical Thermodynamic Analysis

Temperature= 298.150 Kelvin Pressure= 1.00000 Atm

==

SCF Energy= -5342.28816262 Predicted Change= -5.639281D-09

Zero-point correction (ZPE)= -5341.5888 0.69930

Internal Energy (U)= -5341.5469 0.74123

Enthalpy (H)= -5341.5459 0.74218

Gibbs Free Energy (G)= -5341.6641 0.62401

Frequencies -- -80.7980 17.6689 19.3951

==

#M062X/6-31+G(d,p) scf=(maxcycle=300,direct,tight) density=current

SCRF=(PCM,SOLVENT=THF)

Pointgroup= C1 Stoichiometry= C43H36BrN3O2S2 C1[X(C43H36BrN3O2S2)] #Atoms= 87

Charge = 0 Multiplicity = 1

SCF Energy= -5342.41389693

==

Supporting Information: 045-Si-Br_Lactamization-X-equals-S-004.log

----

Using Gaussian 09: AM64L-G09RevD.01 24-Apr-2013

==

#M062X/6-31G(d) scf=(maxcycle=300,direct,tight) density=current

SCRF=(PCM,SOLVENT=THF) opt=(maxcycle=250,ts,calcfc,noeigentest,gdiis)

iop(1/8=18) freq=noraman

#N Geom=AllCheck Guess=TCheck SCRF=Check Test GenChk RM062X/6-31G(d) Freq

----

Pointgroup= C1 Stoichiometry= C43H36BrN3O2S2 C1[X(C43H36BrN3O2S2)] #Atoms= 87

Charge = 0 Multiplicity = 1

SCF Energy= -5342.28464243 Predicted Change= -2.814938D-09

==

Optimization completed. {Found 2 times}

Item	Max Val.	Criteria	Pass?	RMS Val.	Criteria	Pass?
Force	0.00000 ||	0.00045	[YES]	0.00000 ||	0.00030	[YES]
Displ	0.00163 ||	0.00180	[YES]	0.00163 ||	0.00180	[YES]

Atomic Type	Coordinates (Angstroms) X	Y	Z
N	-4.060052	0.308417	0.447096
C	-2.919922	-0.001969	1.091502
N	-1.720823	0.161193	0.543827
C	-0.500595	-0.297685	1.282052
C	0.689090	0.580647	0.967288
O	-0.690028	-0.787492	2.387723
C	-1.605589	0.812198	-0.787515
C	-1.412935	2.315225	-0.642787
C	-2.808125	0.372039	-1.642519
C	-2.783663	0.789017	-3.128684
C	-3.135743	2.249511	-3.432454
C	-1.452782	0.396292	-3.776170
C	-4.107292	0.749035	-0.953043

C	-5.221348	0.147531	1.216379
C	-4.936952	-0.358106	2.482048
S	-3.210791	-0.622397	2.703104
C	-6.525572	0.435762	0.823091
C	-7.541833	0.189655	1.739825
C	-7.263505	-0.327422	3.009382
C	-5.956882	-0.607731	3.394541
C	-2.267296	3.095572	0.143001
C	-2.105643	4.477378	0.210979
C	-1.078283	5.098974	-0.493645
C	-0.195996	4.326206	-1.244557
C	-0.360089	2.945829	-1.313517
C	2.079767	-0.016502	1.226483
C	3.051788	1.086527	0.801382
C	2.349003	-1.315486	0.478982
C	1.312907	-1.983158	-0.176770
C	3.689908	-1.767888	0.266102
C	4.785681	-1.232808	1.152037
O	4.016822	-2.599096	-0.601385
C	3.288362	1.454044	-0.528917
Br	2.441947	0.564501	-1.998126
C	4.170742	2.474303	-0.870565
C	4.848045	3.163235	0.129785
C	4.625885	2.830687	1.463098

C	3.734899	1.812906	1.781881
S	1.622321	-3.252038	-1.388413
C	-0.102275	-3.528958	-1.517157
C	-0.803053	-2.661924	-0.655793
N	0.005561	-1.761662	0.013023
C	-0.755655	-4.485579	-2.287033
C	-2.138726	-4.602875	-2.182183
C	-2.840130	-3.794847	-1.282758
C	-2.187648	-2.838765	-0.511604
C	5.928288	-0.688631	0.562948
C	6.948537	-0.174729	1.356330
C	6.847621	-0.232699	2.746711
C	5.725152	-0.807845	3.338997
C	4.692688	-1.299127	2.542914
H	3.576719	1.540114	2.822292
H	0.662628	0.913082	-0.063229
H	0.566435	1.472969	1.594769
H	-0.718272	0.368189	-1.247066
H	-2.756844	-0.723510	-1.645828
H	-3.165696	2.388092	-4.517860
H	-2.398592	2.947856	-3.028071
H	-4.120643	2.527146	-3.044060
H	-3.564279	0.171964	-3.594855
H	-0.641632	1.062541	-3.461750

H	-1.527784	0.465621	-4.865141
H	-1.168287	-0.631252	-3.518537
H	-4.947883	0.236153	-1.428681
H	-4.297957	1.827039	-0.973251
H	-6.745297	0.844276	-0.157058
H	-8.567566	0.406404	1.461815
H	-8.074986	-0.510847	3.705134
H	-5.736757	-1.007713	4.378709
H	-3.058897	2.636151	0.728736
H	-2.781019	5.066166	0.823549
H	-0.952719	6.175667	-0.439127
H	0.631264	4.794375	-1.768765
H	0.366367	2.352256	-1.862920
H	2.192131	-0.143842	2.310201
H	4.323930	2.717488	-1.916412
H	5.542873	3.952917	-0.137424
H	5.149389	3.357175	2.254841
H	-0.190911	-5.137406	-2.947280
H	-2.666238	-5.341966	-2.776135
H	-3.911957	-3.924209	-1.166111
H	-2.747282	-2.270407	0.221632
H	5.992315	-0.650311	-0.521136
H	7.824449	0.270311	0.893448
H	7.646506	0.162968	3.366903

H	5.652118	-0.871064	4.420846
H	3.809624	-1.740940	2.998463

Statistical Thermodynamic Analysis

Temperature= 298.150 Kelvin Pressure= 1.00000 Atm

==

SCF Energy= -5342.28464243 Predicted Change= -2.814938D-09

Zero-point correction (ZPE)= -5341.5851 0.69950

Internal Energy (U)= -5341.5433 0.74131

Enthalpy (H)= -5341.5423 0.74226

Gibbs Free Energy (G)= -5341.6601 0.62450

Frequencies -- -134.2652 17.3455 18.0938

==

#M062X/6-31+G(d,p) scf=(maxcycle=300,direct,tight) density=current

SCRF=(PCM,SOLVENT=THF)

Pointgroup= C1 Stoichiometry= C43H36BrN3O2S2 C1[X(C43H36BrN3O2S2)]
#Atoms= 87

Charge = 0 Multiplicity = 1

SCF Energy= -5342.41052233

==

Supporting Information: 045-Re-Br_Lactonization-X-equals-S-001.log

----

Using Gaussian 09: AM64L-G09RevD.01 24-Apr-2013

==

#M062X/6-31G(d) scf=(maxcycle=300,direct,tight) density=current

SCRF=(PCM,SOLVENT=THF) opt=(maxcycle=250,ts,calcfc,noeigentest,gdiis)

iop(1/8=18) freq=noraman

#N Geom=AllCheck Guess=TCheck SCRF=Check Test GenChk RM062X/6-31G(d) Freq

----

Pointgroup= C1 Stoichiometry= C43H36BrN3O2S2 C1[X(C43H36BrN3O2S2)] #Atoms= 87

Charge = 0 Multiplicity = 1

SCF Energy= -5342.28390438 Predicted Change= -2.065737D-10

==

Optimization completed. {Found 2 times}

Item	Max Val.	Criteria	Pass?	RMS Val.	Criteria	Pass?
Force	0.00000 ||	0.00045	[YES]	0.00000 ||	0.00030	[YES]
Displ	0.00033 ||	0.00180	[YES]	0.00033 ||	0.00180	[YES]

Atomic Type	Coordinates (Angstroms) X	Y	Z
N	4.317346	-0.366158	-0.449996

C	3.072128	-0.782077	-0.736824
N	2.146291	-0.969869	0.196908
C	0.772198	-1.312355	-0.258690
C	-0.267695	-1.413388	0.830955
O	0.708486	-1.933122	-1.307576
C	2.453901	-0.631687	1.602152
C	2.095663	0.802962	1.954789
C	3.932593	-0.989369	1.877436
C	4.348321	-0.776674	3.344636
C	3.595339	-1.731356	4.275126
C	5.856631	-0.975355	3.531180
C	4.821617	-0.212983	0.916903
C	5.115313	-0.115478	-1.573518
C	4.441013	-0.397297	-2.759604
S	2.813861	-0.992884	-2.452319
C	6.420105	0.369712	-1.574113
C	7.036770	0.554707	-2.806752
C	6.369521	0.263579	-4.001254
C	5.064385	-0.217213	-3.990275
C	2.377171	1.873262	1.099107
C	2.097982	3.178479	1.487681
C	1.530582	3.434768	2.734579
C	1.228265	2.376287	3.585138
C	1.504611	1.067665	3.191914

C	-1.690778	-1.089199	0.336707
C	-2.192742	-2.044167	-0.739389
C	-1.837615	0.375239	-0.018945
C	-3.201141	0.844045	-0.013056
C	-0.721194	1.102123	-0.404519
C	-0.690989	2.600453	-0.447677
O	0.414582	0.566279	-0.686386
C	-2.639309	-3.331365	-0.441667
C	-3.110923	-4.211169	-1.411690
C	-3.147562	-3.798276	-2.738105
C	-2.712944	-2.516783	-3.072157
C	-2.245260	-1.660863	-2.083152
S	-3.748620	2.332118	-0.858841
C	-5.398509	1.921233	-0.466200
C	-5.413865	0.709830	0.254218
N	-4.181573	0.133225	0.480621
C	-6.571174	2.618357	-0.748312
C	-7.778728	2.081600	-0.313118
C	-7.809634	0.874564	0.399003
C	-6.638963	0.186467	0.686583
C	-1.152417	3.358228	0.631172
C	-1.006195	4.742359	0.633192
C	-0.401477	5.381283	-0.447474
C	0.069761	4.629971	-1.524581

C	-0.062695	3.244919	-1.517191
Br	-2.634920	-3.951170	1.365473
H	-0.238079	-2.438982	1.219051
H	-0.056691	-0.722538	1.649500
H	1.846601	-1.308438	2.204698
H	4.044429	-2.058280	1.644806
H	3.823957	-2.772734	4.019870
H	2.510178	-1.606055	4.232767
H	3.902313	-1.564519	5.311576
H	4.102091	0.256371	3.622513
H	6.446060	-0.206316	3.023568
H	6.171529	-1.956285	3.154770
H	6.108818	-0.929592	4.594397
H	5.837609	-0.612440	0.924014
H	4.857103	0.853977	1.169977
H	6.936130	0.606965	-0.650406
H	8.052202	0.934533	-2.837755
H	6.872690	0.416725	-4.949756
H	4.542240	-0.441111	-4.914532
H	2.771658	1.692733	0.103485
H	2.289681	3.995806	0.798592
H	1.300209	4.454777	3.026718
H	0.766925	2.561123	4.550161
H	1.252617	0.244023	3.856324

H	-2.335791	-1.262322	1.204754
H	-3.449684	-5.200296	-1.123353
H	-3.518389	-4.475038	-3.501338
H	-2.738350	-2.184848	-4.105409
H	-1.901065	-0.662753	-2.339013
H	-6.545136	3.553479	-1.299013
H	-8.704862	2.604868	-0.528999
H	-8.762268	0.474071	0.731937
H	-6.650057	-0.747111	1.240117
H	-1.607949	2.851605	1.477894
H	-1.356770	5.321700	1.482361
H	-0.290600	6.461653	-0.448661
H	0.546064	5.124697	-2.365814
H	0.321583	2.647870	-2.339061

Statistical Thermodynamic Analysis

Temperature= 298.150 Kelvin Pressure= 1.00000 Atm

===

SCF Energy= -5342.28390438 Predicted Change= -2.065737D-10

Zero-point correction (ZPE)= -5341.5852 0.69861

Internal Energy (U)= -5341.5431 0.74078

Enthalpy (H)= -5341.5421 0.74173

Gibbs Free Energy (G)= -5341.6625 0.62139

Frequencies -- -138.9287 13.8919 20.0855

==

#M062X/6-31+G(d,p) scf=(maxcycle=300,direct,tight) density=current

SCRF=(PCM,SOLVENT=THF)

Pointgroup= C1 Stoichiometry= C43H36BrN3O2S2 C1[X(C43H36BrN3O2S2)] #Atoms= 87

Charge = 0 Multiplicity = 1

SCF Energy= -5342.40793253

==

Supporting Information: 045-Si-Br_Lactonization-X-equals-S-002.log

----

Using Gaussian 09: AM64L-G09RevD.01 24-Apr-2013

==

#M062X/6-31G(d) scf=(maxcycle=300,direct,tight) density=current

SCRF=(PCM,SOLVENT=THF) opt=(maxcycle=250,ts,calcfc,noeigentest,gdiis)

iop(1/8=18) freq=noraman

#N Geom=AllCheck Guess=TCheck SCRF=Check Test GenChk RM062X/6-31G(d) Freq

----

Pointgroup= C1 Stoichiometry= C43H36BrN3O2S2 C1[X(C43H36BrN3O2S2)] #Atoms= 87

Charge = 0 Multiplicity = 1

SCF Energy= -5342.29387553 Predicted Change= -5.623579D-09

==

Optimization completed. {Found 1 times}

Item	Max Val.	Criteria	Pass?	RMS Val.	Criteria	Pass?
Force	0.00000 ||	0.00045	[YES]	0.00000 ||	0.00030	[YES]
Displ	0.00656 ||	0.00180	[NO]	0.00656 ||	0.00180	[NO]

Atomic Type	Coordinates (Angstroms) X	Y	Z
N	4.244266	-1.019359	-0.297529
C	3.052072	-1.045077	-0.916447
N	2.116268	-0.129655	-0.689144
C	0.771036	-0.317939	-1.243513
C	-0.059709	0.930070	-1.380006
O	0.581486	-1.304159	-1.933997
C	2.344352	0.929034	0.322691
C	2.842528	2.209141	-0.322829
C	3.232816	0.361225	1.443012
C	3.477770	1.362137	2.585729
C	2.153402	1.786380	3.228233
C	4.404377	0.764954	3.649750
C	4.534930	-0.166755	0.860700
C	5.154001	-1.979293	-0.760015

C	4.595351	-2.792651	-1.742813
S	2.922529	-2.353298	-2.070203
C	6.467820	-2.149543	-0.332373
C	7.205679	-3.173127	-0.916128
C	6.647084	-4.001015	-1.895705
C	5.334442	-3.820798	-2.318929
C	3.922534	2.218831	-1.212089
C	4.380269	3.415444	-1.756931
C	3.759533	4.618075	-1.426811
C	2.664325	4.614767	-0.567265
C	2.204937	3.417299	-0.024160
C	-1.578629	0.658116	-1.292401
C	-2.035043	-0.368380	-0.264623
C	-3.388541	-0.816508	-0.469026
C	-1.156157	-0.888120	0.675965
C	-1.618527	-1.668807	1.868781
O	0.111719	-0.711028	0.641512
S	-4.180527	-2.136328	0.467972
C	-5.578986	-1.999807	-0.565256
C	-5.357543	-0.985092	-1.517466
N	-4.135209	-0.353318	-1.440325
C	-6.771421	-2.718155	-0.524601
C	-7.755044	-2.419186	-1.461900
C	-7.547869	-1.415073	-2.417840

C	-6.360216	-0.696496	-2.452343
C	-1.176293	-2.979240	2.062950
C	-1.566600	-3.687226	3.195280
C	-2.370123	-3.075034	4.157673
C	-2.778798	-1.753722	3.987099
C	-2.407608	-1.052294	2.842898
C	-2.269388	2.008977	-1.109003
C	-2.166691	2.798277	0.039593
Br	-1.149146	2.230688	1.554650
C	-2.776897	4.044992	0.150890
C	-3.523455	4.539396	-0.911749
C	-3.648468	3.780514	-2.072759
C	-3.030237	2.538700	-2.157600
H	0.233197	1.688484	-0.661650
H	0.158056	1.340623	-2.374552
H	1.364677	1.092222	0.770627
H	2.673966	-0.493066	1.851690
H	1.546066	2.403723	2.561954
H	2.343408	2.371874	4.132541
H	1.558708	0.908237	3.509262
H	3.964656	2.251793	2.162298
H	5.413183	0.578216	3.269883
H	3.999229	-0.180247	4.030629
H	4.497218	1.454156	4.493748

H	5.054030	-0.797526	1.585248
H	5.206610	0.645278	0.556970
H	6.905892	-1.502800	0.419808
H	8.233101	-3.327679	-0.604784
H	7.244053	-4.793336	-2.334146
H	4.897949	-4.461218	-3.077976
H	4.403762	1.289333	-1.507354
H	5.219748	3.405556	-2.444940
H	4.117566	5.550597	-1.851480
H	2.154526	5.542647	-0.328049
H	1.312572	3.415382	0.598554
H	-1.900196	0.291385	-2.272396
H	-6.928657	-3.494257	0.218076
H	-8.690001	-2.970450	-1.451152
H	-8.327451	-1.197155	-3.141592
H	-6.191716	0.084094	-3.187833
H	-0.539805	-3.439811	1.312325
H	-1.240838	-4.714061	3.332271
H	-2.669302	-3.626591	5.043977
H	-3.391306	-1.271473	4.743052
H	-2.720891	-0.022706	2.690511
H	-2.662604	4.616193	1.065851
H	-4.001672	5.509953	-0.827160
H	-4.230974	4.152047	-2.909969

H -3.143674 1.936409 -3.054384

Statistical Thermodynamic Analysis

Temperature= 298.150 Kelvin Pressure= 1.00000 Atm

===

SCF Energy= -5342.29387553 Predicted Change= -5.623579D-09

Zero-point correction (ZPE)= -5341.5943 0.69954

Internal Energy (U)= -5341.5521 0.74175

Enthalpy (H)= -5341.5511 0.74269

Gibbs Free Energy (G)= -5341.6722 0.62162

Frequencies -- -126.5909 7.2360 17.7401

===

#M062X/6-31+G(d,p) scf=(maxcycle=300,direct,tight) density=current

SCRF=(PCM,SOLVENT=THF)

Pointgroup= C1 Stoichiometry= C43H36BrN3O2S2 C1[X(C43H36BrN3O2S2)] #Atoms= 87

Charge = 0 Multiplicity = 1

SCF Energy= -5342.41741899

===

Supporting Information: Re-Si-Ketene-formation_X-equals-S-PhBr_substrate-001.log

----

Using Gaussian 09: AM64L-G09RevD.01 24-Apr-2013

==

#M062X/6-31G(d) scf=(maxcycle=300,direct,tight) density=current

SCRF=(PCM,SOLVENT=THF) opt=(maxcycle=250,ts,calcfc,noeigentest,gdiis)

iop(1/8=18) freq=noraman

#N Geom=AllCheck Guess=TCheck SCRF=Check Test GenChk RM062X/6-31G(d) Freq

----

Pointgroup= C1 Stoichiometry= C43H36BrN3O2S2 C1[X(C43H36BrN3O2S2)]
#Atoms= 87

Charge = 0 Multiplicity = 1

SCF Energy= -5342.27076755 Predicted Change= -1.830376D-10

==

Optimization completed. {Found 2 times}

Item	Max Val.	Criteria	Pass?	RMS Val.	Criteria	Pass?
Force	0.00000 ||	0.00045	[YES]	0.00000 ||	0.00030	[YES]
Displ	0.00040 ||	0.00180	[YES]	0.00040 ||	0.00180	[YES]

Atomic Type	Coordinates (Angstroms) X	Y	Z
C	7.200753	-0.664072	0.863969
C	6.054395	0.101744	1.071964

C	5.009767	-0.017710	0.159639
C	5.116666	-0.870894	-0.943234
C	6.256709	-1.635884	-1.144110
C	7.303876	-1.524626	-0.229317
N	3.780716	0.633325	0.213861
C	2.898999	0.308470	-0.789903
S	3.646540	-0.860847	-1.902788
C	3.385903	1.478084	1.333023
C	1.865531	1.568211	1.415555
C	1.428456	2.609001	2.460519
C	-0.095935	2.653242	2.596435
C	2.062699	2.319506	3.825308
C	1.242844	1.779371	0.017491
C	1.400874	3.176363	-0.562561
N	1.716223	0.784763	-0.951979
C	0.299890	-0.511843	-1.808317
C	-0.858821	0.099502	-1.545948
C	-2.050218	-0.787931	-1.297612
C	-3.378815	-0.095457	-1.547185
O	0.932215	-1.431520	-2.202011
C	0.281474	3.997966	-0.716996
C	0.405335	5.285366	-1.237270
C	1.653657	5.766331	-1.619794
C	2.773645	4.945267	-1.497483

C	2.647079	3.659707	-0.979534
C	-3.846256	1.015243	-0.835941
Br	-2.789225	1.853266	0.514786
C	-5.118209	1.542090	-1.030879
C	-5.960622	0.968811	-1.977289
C	-5.520930	-0.123167	-2.718656
C	-4.249514	-0.642111	-2.497297
C	2.230271	-4.560815	-0.089195
C	1.178576	-3.669261	0.133204
C	1.370268	-2.483139	0.866187
C	2.631546	-2.191316	1.403191
C	3.679599	-3.066836	1.169033
C	3.479523	-4.241155	0.424173
S	-0.482405	-3.771992	-0.383387
C	-0.764154	-2.202212	0.365770
C	-2.031773	-1.434707	0.166816
C	-3.300174	-2.263892	0.271241
C	-4.489425	-1.660624	0.942283
O	-3.370920	-3.332743	-0.310128
N	0.248383	-1.675244	0.975701
C	-5.753060	-1.967623	0.426065
C	-6.887541	-1.369796	0.959286
C	-6.767875	-0.481227	2.028387
C	-5.514429	-0.193790	2.563866

C	-4.373860	-0.773966	2.016951
H	8.023532	-0.583668	1.566741
H	5.983632	0.774594	1.919841
H	6.327454	-2.307638	-1.993492
H	8.204576	-2.111873	-0.373358
H	3.841649	2.472460	1.231931
H	3.783692	1.018795	2.243032
H	-0.505807	1.645110	2.741392
H	-0.565458	3.091159	1.712100
H	-0.384566	3.263956	3.457294
H	1.774067	3.596205	2.121602
H	3.151604	2.422698	3.810774
H	1.819420	1.301741	4.155056
H	1.678989	3.016321	4.576258
H	1.489031	0.586833	1.739127
H	0.174078	1.587946	0.145471
H	-0.896022	1.153620	-1.337298
H	-2.012894	-1.651747	-1.972411
H	-0.703638	3.620029	-0.451194
H	-0.477979	5.906654	-1.350398
H	1.753558	6.767919	-2.026345
H	3.747936	5.303218	-1.816035
H	3.525456	3.021053	-0.927922
H	-5.441187	2.392960	-0.441128

H	-6.954051	1.378427	-2.128072
H	-6.168143	-0.578667	-3.461071
H	-3.917919	-1.509581	-3.061522
H	2.078301	-5.472996	-0.657099
H	2.770495	-1.288294	1.991528
H	4.668191	-2.840351	1.559068
H	4.314945	-4.911307	0.247199
H	-2.007593	-0.618122	0.885547
H	-5.822812	-2.654547	-0.411793
H	-7.864674	-1.590253	0.540989
H	-7.654660	-0.013533	2.445275
H	-5.423418	0.488371	3.403113
H	-3.401025	-0.543542	2.440114

Statistical Thermodynamic Analysis

Temperature= 298.150 Kelvin Pressure= 1.00000 Atm

===

SCF Energy= -5342.27076755 Predicted Change= -1.830376D-10

Zero-point correction (ZPE)= -5341.5741 0.69662

Internal Energy (U)= -5341.5312 0.73953

Enthalpy (H)= -5341.5302 0.74047

Gibbs Free Energy (G)= -5341.6503 0.62044

Frequencies -- -156.8697 17.3060 25.8278

==

#M062X/6-31+G(d,p) scf=(maxcycle=300,direct,tight) density=current

SCRF=(PCM,SOLVENT=THF)

Pointgroup= C1 Stoichiometry= C43H36BrN3O2S2 C1[X(C43H36BrN3O2S2)] #Atoms= 87

Charge = 0 Multiplicity = 1

SCF Energy= -5342.39234760

==

Supporting Information: 040-Ketene-PhBr_X-equals-S-Re-Si-Nuc-attack-009.log

----

Using Gaussian 09: AM64L-G09RevD.01 24-Apr-2013

==

#M062X/6-31G(d) scf=(maxcycle=300,direct,tight) density=current

SCRF=(PCM,SOLVENT=THF) opt=(maxcycle=250,gdiis) iop(1/8=18) freq=noraman

#N Geom=AllCheck Guess=TCheck SCRF=Check Test GenChk RM062X/6-31G(d) Freq

----

Pointgroup= C1 Stoichiometry= C24H16BrNO2S C1[X(C24H16BrNO2S)] #Atoms= 45

Charge = 0 Multiplicity = 1

SCF Energy= -4098.86539007 Predicted Change= -5.250579D-09

==

Optimization completed. {Found 2 times}

Item	Max Val.	Criteria	Pass?	RMS Val.	Criteria	Pass?
Force	0.00001 ||	0.00045	[YES]	0.00000 ||	0.00030	[YES]
Displ	0.00073 ||	0.00180	[YES]	0.00073 ||	0.00180	[YES]

Atomic Type	Coordinates (Angstroms) X	Y	Z
C	-1.545583	-3.006114	0.130166
C	-0.415415	-2.379581	0.376919
C	0.067258	-1.242267	-0.498111
C	1.555026	-1.314344	-0.810351
O	-2.537065	-3.584916	-0.075142
C	2.580180	-1.241958	0.138336
Br	2.201022	-1.132386	1.998732
C	3.923436	-1.209330	-0.219581
C	4.276005	-1.256318	-1.563823
C	3.282453	-1.340452	-2.534233
C	1.945199	-1.368835	-2.153568
C	-5.381122	1.601820	0.717516
C	-4.113796	1.092326	0.431921
C	-3.939979	0.006569	-0.447312
C	-5.053934	-0.587445	-1.050920
C	-6.312842	-0.082857	-0.768667

C	-6.474382	1.003082	0.107029
S	-2.556933	1.596588	1.032274
C	-1.815283	0.326372	0.059151
C	-0.324209	0.163042	0.065064
C	0.329183	1.198837	-0.867687
C	1.610660	1.837365	-0.447755
O	-0.141722	1.379962	-1.972588
N	-2.624147	-0.393235	-0.637422
C	1.929319	2.084602	0.890436
C	3.157795	2.650757	1.218890
C	4.077035	2.951706	0.216752
C	3.762446	2.707050	-1.120867
C	2.529242	2.162464	-1.452940
H	0.136469	-2.697386	1.252775
H	-0.453570	-1.306011	-1.457062
H	4.680522	-1.138779	0.553579
H	5.323650	-1.226217	-1.844849
H	3.544590	-1.377947	-3.586458
H	1.171265	-1.416706	-2.914771
H	-5.508648	2.438239	1.396501
H	-4.910595	-1.426601	-1.723815
H	-7.187091	-0.531427	-1.228991
H	-7.470561	1.381897	0.311962
H	0.049408	0.257298	1.086156

H	1.220227	1.857701	1.680552
H	3.397914	2.851817	2.257977
H	5.040792	3.378645	0.477223
H	4.480938	2.938795	-1.900753
H	2.266990	1.958090	-2.486413

Statistical Thermodynamic Analysis

Temperature= 298.150 Kelvin Pressure= 1.00000 Atm

===

SCF Energy= -4098.86539007 Predicted Change= -5.250579D-09

Zero-point correction (ZPE)= -4098.5254 0.33995

Internal Energy (U)= -4098.5012 0.36418

Enthalpy (H)= -4098.5002 0.36512

Gibbs Free Energy (G)= -4098.5817 0.28364

Frequencies -- 24.8487 28.4834 34.0731

===

#M062X/6-31+G(d,p) scf=(maxcycle=300,direct,tight) density=current

SCRF=(PCM,SOLVENT=THF)

Pointgroup= C1 Stoichiometry= C24H16BrNO2S C1[X(C24H16BrNO2S)] #Atoms= 45

Charge = 0 Multiplicity = 1

SCF Energy= -4098.94609139

==

Supporting Information: 040-Ketene-PhBr_X-equals-S_Re-Si_Acid-catalysis-017.log

----

Using Gaussian 09: AM64L-G09RevD.01 24-Apr-2013

==

#M062X/6-31G(d) scf=(maxcycle=300,direct,tight) density=current

SCRF=(PCM,SOLVENT=THF) opt=(maxcycle=250,gdiis) iop(1/8=18) freq=noraman

#N Geom=AllCheck Guess=TCheck SCRF=Check Test GenChk RM062X/6-31G(d) Freq

----

Pointgroup= C1 Stoichiometry= C33H23Br2NO4S C1[X(C33H23Br2NO4S)] #Atoms= 64

Charge = 0 Multiplicity = 1

SCF Energy= -7168.09272004 Predicted Change= -2.020645D-08

==

Optimization completed. {Found 2 times}

Item	Max Val.	Criteria	Pass?	RMS Val.	Criteria	Pass?
Force	0.00001 ||	0.00045	[YES]	0.00000 ||	0.00030	[YES]
Displ	0.00140 ||	0.00180	[YES]	0.00140 ||	0.00180	[YES]

Atomic Coordinates (Angstroms)

Type X Y Z

C	-0.007766	-2.453918	1.471431
C	-1.147259	-2.060700	0.942876
C	-1.847963	-0.770149	1.309239
C	-3.361075	-0.929517	1.287268
O	0.990746	-2.868519	1.911767
C	-4.121827	-1.230013	0.152741
Br	-3.303248	-1.512509	-1.544651
C	-5.511200	-1.280567	0.179535
C	-6.182559	-1.030172	1.371362
C	-5.457582	-0.733680	2.521437
C	-4.068722	-0.687141	2.470073
C	3.455597	2.552695	0.842371
C	2.233937	1.878761	0.871574
C	2.003153	0.815353	1.764412
C	3.016674	0.403149	2.636641
C	4.233230	1.064131	2.601415
C	4.447568	2.133782	1.716777
S	0.813073	2.124935	-0.106986
C	0.029896	0.810815	0.770702
C	-1.394291	0.459305	0.459180
C	-2.319965	1.617407	0.863182
C	-3.454430	1.977939	-0.034788
O	-2.180647	2.135655	1.954077
N	0.739090	0.245511	1.684600

C	-3.387075	1.831217	-1.422919
C	-4.490580	2.154298	-2.208285
C	-5.664601	2.604118	-1.609181
C	-5.734168	2.754597	-0.223016
C	-4.629208	2.452007	0.561215
H	-1.574485	-2.743910	0.217864
H	-1.579519	-0.509696	2.336086
H	-6.056553	-1.505384	-0.730313
H	-7.266811	-1.064519	1.393315
H	-5.969538	-0.533400	3.456852
H	-3.506187	-0.438377	3.365518
H	3.630199	3.365803	0.146196
H	2.835516	-0.430728	3.307516
H	5.037398	0.745446	3.256983
H	5.411572	2.633287	1.704379
H	-1.477475	0.221320	-0.602081
H	-2.475011	1.482536	-1.899128
H	-4.434135	2.048333	-3.286885
H	-6.528289	2.841754	-2.222674
H	-6.650745	3.105113	0.240896
H	-4.661756	2.555625	1.641457
C	0.434622	-1.860288	-1.800253
C	1.748164	-2.048699	-1.153936
C	2.580821	-1.011165	-1.006968

C	3.890747	-1.087624	-0.349014
O	-0.192396	-3.037176	-1.980079
O	-0.047225	-0.801812	-2.148332
C	4.929790	-0.198799	-0.649068
Br	4.685407	1.148886	-1.959529
C	6.168851	-0.275191	-0.022947
C	6.391472	-1.260568	0.932634
C	5.372066	-2.151986	1.263881
C	4.139761	-2.059126	0.633642
H	1.996366	-3.055676	-0.833143
H	2.272367	-0.048789	-1.411646
H	-1.069798	-2.845488	-2.357312
H	6.947613	0.431496	-0.287817
H	7.357920	-1.322497	1.422635
H	5.533898	-2.910392	2.022725
H	3.328770	-2.719411	0.925724

Statistical Thermodynamic Analysis

Temperature= 298.150 Kelvin Pressure= 1.00000 Atm

===

SCF Energy= -7168.09272004 Predicted Change= -2.020645D-08

Zero-point correction (ZPE)= -7167.6099 0.48275

Internal Energy (U)= -7167.5737 0.51892

Enthalpy (H)= -7167.5728 0.51987

Gibbs Free Energy (G)= -7167.6805 0.41219

Frequencies -- 18.5045 24.3759 28.4463

==

#M062X/6-31+G(d,p) scf=(maxcycle=300,direct,tight) density=current

SCRF=(PCM,SOLVENT=THF)

Pointgroup= C1 Stoichiometry= C33H23Br2NO4S C1[X(C33H23Br2NO4S)] #Atoms= 64

Charge = 0 Multiplicity = 1

SCF Energy= -7168.22884747

==

References

[i] a) Y. Zhao, D. G. Truhlar, *Theor. Chem. Acc.* **2008**, *120*, 215–241. b) P. C. Hariharan, J. A. Pople, *Theor. Chim. Acta.* **1973**, *28*, 213–222. c) W. J. Hehre, R. Ditchfield, J. A. Pople, *J. Chem. Phys.* **1972**, *56*, 2257–2261.
[ii] S. Miertuš, E. Scrocco, J. Tomasi, *Chem. Phys.* **1981**, *55*, 117–129.
[iii] CYLview, 1.0b; Legault, C. Y., Université de Sherbrooke, **2009**.
[iv] H. Eyring, *J. Chem. Phys.* **1935**, *3*, 107–115.
